# Supplementary material for: Steric control of signaling bias in the immunometabolic receptor GPR84
Source: Nat Commun. 2026 Sep 5;17:10562. doi: 10.1038/s41467-026-77312-7 (PMC13642346; doi:10.1038/s41467-026-77312-7)
Supplement: Supplementary file 1 [file 41467_2026_77312_MOESM1_ESM.pdf]

Supporting information for

Steric control of signaling bias in the immunometabolic receptor GPR84

Authors and affiliations:

Pinqi Wang[1,2,¶,§], Xuan Zhang[3,§], Abdul-Akim Guseinov[4,5,§], Laura Jenkins[5,§], Carl von Hallerstein[6,§] Jonathan D. Colburn[6], Rowan Ives[1,2,7], Vincent B. Luscombe[7], Sara Marsango[5], Listiana Oktavia[1,2], Arun Raja[1,2], David R. Greaves[7], Philip C. Biggin[6], Graeme Milligan[5*], Cheng Zhang[3*], Irina G. Tikhonova[4*] & Angela J. Russell[1,2*]

[1] Department of Chemistry, Chemistry Research Laboratory, University of Oxford, Mansfield Road, Oxford OX1 3TA, U.K.

[2] Department of Pharmacology, University of Oxford, Mansfield Road, Oxford OX1 3QT, U.K.

[3] Department of Pharmacology and Chemical Biology, School of Medicine, University of Pittsburgh, Pittsburgh, PA 15213, U.S.A.

[4] School of Pharmacy, Queen's University Belfast, Belfast BT9 7BL, Northern Ireland, U.K.

[5] School of Molecular Biosciences, College of Medical, Veterinary and Life Sciences, University of Glasgow, Glasgow, G12 8QQ, U.K.

[6] Department of Biochemistry, University of Oxford, South Parks Road, Oxford, OX1 3QU, U.K.

[7] Sir William Dunn School of Pathology, University of Oxford, South Parks Road, Oxford OX1 3RE, U.K.

¶ Current affiliation: Shanghai Academy of Artificial Intelligence for Science, Shanghai, China.

§ These authors contributed equally: Pinqi Wang, Xuan Zhang, Abdul-Akim Guseinov, Laura Jenkins, Carl von Hallerstein.

*email: Graeme.Milligan@glasgow.ac.uk; chengzh@pitt.edu; i.tikhonova@qub.ac.uk; angela.russell@chem.ox.ac.uk

Supplementary Tables

Supplementary Table 1: Potency and relative efficacy of compounds at orthosteric site mutations in the [^{35}S]GTPγS binding assay

	PSB-16671		OX04539		OX04954		OX04529	
	pEC_{50} ± SEM	Emax ± SEM	pEC_{50} ± SEM	Emax ± SEM	pEC_{50} ± SEM	Emax ± SEM	pEC_{50} ± SEM	Emax ± SEM
GPR84-WT	6.73 ± 0.15	100	9.61 ± 0.05	150 ± 14	9.18 ± 0.20	129 ± 12	8.93 ± 0.03	119 ± 13
Y69A	6.96 ± 0.07	100	9.04 ± 0.02**	101 ± 14	8.29 ± 0.11***	106 ± 4	8.34 ± 0.11**	94 ± 9
Y81A	6.49 ± 0.11	100	8.27 ± 0.16****	134 ± 5	7.79 ± 0.19****	133 ± 18	7.67 ± 0.07****	85 ± 4
N104A	6.14 ± 0.08****	100	8.12 ± 0.10****	150 ± 23	7.93 ± 0.07****	130 ± 20	8.40 ± 0.02*	68 ± 8
T167A	6.32 ± 0.05**	100	8.23 ± 0.06****	139 ± 7	7.82 ± 0.10****	129 ± 15	7.75 ± 0.10****	82 ± 10
S169A	6.18 ± 0.04***	100	6.32 ± 0.07****	84 ± 4	6.42 ± 0.14****	47 ± 11*	6.38 ± 0.04****	30 ± 2**
R172A	6.09 ± 0.06****	100	5.67 ± 0.05****	42 ± 4	NR	NR	NR	NR
W360A	7.09 ± 0.02*	100	8.23 ± 0.03****	94 ± 13	7.87 ± 0.15****	75 ± 8	8.06 ± 0.07****	75 ± 10

*p<0.05, **p<0.01, ***p<0.001 ****p<0.0001 compared to wild type. One-way ANOVA followed by Dunnett's multiple comparison test. Emax calculated as % of PSB-16671 response, NR, no detectable response (n=4 for PSB-16671, n=3 for all other experiments). Data represent means ± S.E.M.

Source data are provided within the Source Data file.

Supplementary Table 2: Cryo-EM data collection, refinement and validation statistics

	OX04529-GPR84-G_i complex (EMDB-71830) (PDB 9PT4)
**Data collection and processing**	
Magnification	105,000
Voltage (kV)	300
Electron exposure (e–/Å^2)	58
Defocus range (μm)	-1.0 to -1.8
Pixel size (Å)	0.414
Symmetry imposed	C1
Initial particle images (no.)	6,052,532
Final particle images (no.)	218,137
Map resolution (Å)	3.03
FSC threshold	0.143
Map resolution range (Å)	2.0-4.0
**Refinement**	
Initial model used (PDB code)	8G05
Model resolution (Å)	3.3
FSC threshold	0.5
Map sharpening *B* factor (Å^2)	-116.7
Model composition	
Non-hydrogen atoms	8513
Protein residues	1136
Ligand	1
B factors (Å^2)	
Protein	95.58
Ligand	104.49
R.m.s. deviations	
Bond lengths (Å)	0.004
Bond angles (°)	0.551
Validation	
MolProbity score	1.55
Clashscore	7.50
Poor rotamers (%)	0.35
Ramachandran plot	
Favored (%)	97.23
Allowed (%)	2.77
Disallowed (%)	0

Supplementary Table 3: Potency of compounds in [^{35}S]GTPγS binding assay at Phe^{187}Ala and Leu^{336}Ala GPR84

	OX04539 pEC$_{50}$ ± SEM	OX04954 pEC$_{50}$ ± SEM	OX04529 pEC$_{50}$ ± SEM	OX04959 pEC$_{50}$ ± SEM	OX04960 pEC$_{50}$ ± SEM	PSB-16671 pEC$_{50}$ ± SEM	6-OAU pEC$_{50}$ ± SEM
GPR84-WT	9.49 ± 0.12	9.18 ± 0.20	8.90 ± 0.04	8.40 ± 0.05	8.20 ± 0.09	6.73 ± 0.15	6.48 ± 0.03
F187A	10.08 ± 0.17**	9.97 ± 0.10**	9.99 ± 0.01****	nd	nd	7.08 ± 0.09*	7.50 ± 0.18***
L336A	8.64 ± 0.07****	10.37 ± 0.16****	10.16 ± 0.09****	nd	nd	6.48 ± 0.07	6.32 ± 0.10

*p<0.05, **p<0.01, ***p<0.001 ****p<0.0001 compared to wild type. One-way ANOVA followed by Dunnett's multiple comparison test (n=4 for PSB-16671, n=3 for all other experiments). nd = not determined Data represent mean ± S.E.M.

Source data are provided within the Source Data file.

Supplementary Table 4: The potency of OX04529 to recruit β-arrestins isoforms is enhanced at Phe^{187}Ala and Leu^{336}Ala GPR84

	OX04539		OX04954		OX04529	
	β-arrestin-1 pEC$_{50}$ ± SEM	β-arrestin-2 pEC$_{50}$ ± SEM	β-arrestin-1 pEC$_{50}$ ± SEM	β-arrestin-2 pEC$_{50}$ ± SEM	β-arrestin-1 pEC$_{50}$ ± SEM	β-arrestin-2 pEC$_{50}$ ± SEM
GPR84-WT	8.17 ± 0.05	8.42 ± 0.08	7.40 ± 0.05	7.55 ± 0.16	7.31 ± 0.09	7.23 ± 0.14
F187A	8.63 ± 0.08**	8.69 ± 0.06	8.24 ± 0.04****	8.39 ± 0.05**	8.42 ± 0.17***	8.51 ± 0.22**
L336A	7.51 ± 0.08***	7.56 ± 0.11***	8.67 ± 0.05****	8.88 ± 0.07***	8.73 ± 0.08***	8.91 ± 0.06***

*p<0.05, **p<0.01, ***p<0.001 ****p<0.0001 compared to wild type. One-way ANOVA followed by Dunnett's multiple comparison test (n=3). Data represent mean ± S.E.M.

Source data are provided within the Source Data file.

Supplementary Table 5: Potency and efficacy of compounds in [^{35}S]GTPγS binding assays at various mutants of GPR84

	PSB-16671		OX04539		OX04954		OX04529	
	pEC_{50} ± SEM	Emax ± SEM	pEC_{50} ± SEM	Emax ± SEM	pEC_{50} ± SEM	Emax ± SEM	pEC_{50} ± SEM	Emax ± SEM
GPR84-WT	6.73 ± 0.15	100	9.61 ± 0.05	150 ± 14	9.18 ± 0.20	129 ± 12	8.93 ± 0.03	119 ± 13
Y332A	6.23 ± 0.03***	100	8.14 ± 0.17****	57 ± 10	9.37 ± 0.21	60 ± 11	9.46 ± 0.27*	50 ± 12
Y332F	6.46 ± 0.07	100	8.95 ± 0.09***	167 ± 2*	8.38 ± 0.03**	151 ± 10	8.59 ± 0.07	134 ± 12
N362S	6.23 ± 0.08***	100	7.91 ± 0.09****	196 ± 32***	7.62 ± 0.02****	124 ± 19	7.78 ± 0.08****	126 ± 30

*p<0.05, **p<0.01, ***p<0.001 ****p<0.0001 compared to wild type. One-way ANOVA followed by Dunnett's multiple comparison test. Emax calculated as % of PSB-16671 response (n=4 for PSB-16671, n=3 for all other experiments). Data represent mean ± S.E.M.

Source data are provided within the Source Data file.

Supplementary Table 6: The potency and efficacy of OX04529, OX04539 and OX04954 to recruit β-arrestin-1 to various mutants of GPR84

	OX04539		OX04954		OX04529	
	pEC_{50} ± SEM	Emax ± SEM	pEC_{50} ± SEM	Emax ± SEM	pEC_{50} ± SEM	Emax ± SEM
GPR84-WT	8.25 ± 0.11	100	7.50 ± 0.11	71 ± 4**	7.10 ± 0.11	18 ± 4****
N104A	7.08 ± 0.09***	100	NR	NR	NR	NR
S331A	8.25 ± 0.21	100	7.51 ± 0.04	66 ± 7**	7.54 ± 0.22	24 ± 6****
Y332A	NR	NR	NR	NR	NR	NR
Y332F	7.83 ± 0.11	100	7.30 ± 0.06	40 ± 4***	NR	NR
N362S	7.11 ± 0.08***	100	NR	NR	NR	NR

**p<0.01, ***p<0.001 ****p<0.0001 compared to wild type. One-way ANOVA followed by Dunnett's multiple comparison test (n=3). Emax calculated as % of OX04539 response. NR: no response. Data represents mean ± S.E.M.

Source data are provided within the Source Data file.

Supplementary Table 7: Summary of cAMP inhibition and β-arrestin-2 recruitment of OX04529 analogs at wild type GPR84

	cAMP pEC50 ± SEM	cAMP %Emax ± SEM	β-arrestin-2 pEC50 ± SEM	β-arrestin-2 %Emax ± SEM
**OX04529**	10.92 ± 0.18	97 ± 0.2	ND	10 ± 1.5
**OX04539**	11.25 ± 0.10<br>ns	100 ± 0.8<br>ns	8.11 ± 0.25	100<br>****
**OX04540**	10.69 ± 0.09<br>ns	97 ± 0.7<br>ns	6.96 ± 0.17<br>*	81 ± 5.3<br>****
**OX04954**	10.90 ± 0.23<br>ns	98 ± 1.8<br>ns	7.03 ± 0.24<br>*	71 ± 1.3<br>****
**OX04955**	10.45 ± 0.20<br>ns	101 ± 1.5<br>ns	6.58 ± 0.23<br>**	21 ± 2.9<br>ns
**OX04956**	11.04 ± 0.12<br>ns	98 ± 2.1<br>ns	6.86 ± 0.39<br>*	34 ± 6.0<br>****
**OX04957**	10.41 ± 0.11<br>ns	102 ± 3.0<br>ns	6.68 ± 0.23<br>**	46 ± 5.8<br>****
**OX04958**	10.39 ± 0.13<br>ns	99 ± 0.1<br>ns	7.15 ± 0.01<br>ns	72 ± 1.3<br>****
**OX04959**	11.14 ± 0.25<br>ns	95 ± 11.5<br>ns	6.82 ± 0.14<br>**	24 ± 2.3<br>*
**OX04960**	10.99 ± 0.15<br>ns	107 ± 2.1<br>ns	6.35 ± 0.31<br>***	45 ± 1.2<br>****
**OX04961**	10.95 ± 0.26<br>ns	103 ± 3.6<br>ns	7.40 ± 0.04<br>ns	21 ± 0.3<br>ns
**OX04962**	11.03 ± 0.32<br>ns	97 ± 3.4<br>ns	6.75 ± 0.36<br>**	18 ± 2.2<br>ns
**OX04963**	11.19 ± 0.12<br>ns	106 ± 3.8<br>ns	7.43 ± 0.18<br>ns	15 ± 1.1<br>ns
**OX04964**	10.84 ± 0.31<br>ns	94 ± 7.4<br>ns	ND	9 ± 1.4<br>ns
**OX04965**	11.40 ± 0.08<br>ns	103 ± 3.7<br>ns	ND	9 ± 3.0<br>ns
**OX04966**	9.77 ± 0.21<br>**	107 ± 5.6<br>ns	ND	5 ± 1.5<br>ns

*p<0.05, **p<0.01, ***p<0.001 ****p<0.0001, ns not significant. One-way ANOVA followed by Dunnett's multiple comparison test (n=3). ND = not determinable. All compounds are compared to OX04529, β-arrestin-2 pEC_{50} is compared to OX04539.

Source data are provided within the Source Data file.

Gene	TM5, 5.47	TM6, 6.52	Gene	TM5, 5.47	TM6, 6.52	Gene	TM5, 5.47	TM6, 6.52
ACKR1	L	G	GPR15	F	N	MLNR	F	H
ACKR2	F	N	GPR150	F	F	MRGPRD	V	S
ACKR3	F	H	GPR151	F	W	MRGPRE	L	G
ACKR4	F	N	GPR152	G	Q	MRGPRF	L	G
ADORA1	V	H	GPR153	G	L	MRGPRG	V	V
ADORA2A	V	H	GPR160	L	V	MRGPRX1	L	G
ADORA2B	V	H	GPR161	A	M	MRGPRX2	L	G
ADORA3	I	S	GPR162	L	L	MRGPRX3	L	G
ADRA1A	F	F	GPR17	F	H	MRGPRX4	L	G
ADRA1B	F	F	GPR171	F	H	MTNR1A	F	N
ADRA1D	F	F	GPR173	F	I	MTNR1B	F	N
ADRA2A	F	F	GPR174	F	H	NMBR	F	H
ADRA2B	F	F	GPR176	V	A	NMUR1	F	H
ADRA2C	F	F	GPR18	F	H	NMUR2	Y	H
ADRB1	F	F	GPR182	F	H	NPBWR1	F	H
ADRB2	F	F	GPR183	Y	H	NPBWR2	F	H
ADRB3	F	F	GPR19	F	H	NPFFR1	Y	W
AGTR1	F	Q	GPR20	F	H	NPFFR2	Y	W
AGTR2	F	H	GPR21	L	I	NPSR1	Y	F
APLNR	F	H	GPR22	F	S	NPY1R	Y	T
AVPR1A	F	F	GPR25	F	S	NPY2R	Y	H
AVPR1B	F	F	GPR26	F	V	NPY4R	Y	H
AVPR2	F	F	GPR27	F	V	NPY5R	Y	H
BDKRB1	F	H	GPR3	F	T	NPY6R	Y	I
BDKRB2	F	Q	GPR31	F	F	NTSR1	F	H
BRS3	Y	H	GPR32	F	N	NTSR2	F	H
C3AR1	F	H	GPR33	F	H	OPN1LW	C	T
C5AR1	F	Q	GPR34	F	H	OPN1MW	C	A
C5AR2	F	H	GPR35	F	H	OPN1SW	F	A
CCKAR	F	F	GPR37	F	N	OPN3	L	I
CCKBR	F	Y	GPR37L1	F	N	OPN4	F	S
CCR1	L	N	GPR39	F	Q	OPN5	L	A
CCR10	F	S	GPR4	F	H	OPRD1	F	H
CCR2	L	N	GPR42	F	N	OPRK1	F	H
CCR3	L	N	GPR45	F	S	OPRL1	F	Q
CCR4	L	N	GPR50	F	N	OPRM1	F	H
CCR5	L	N	GPR52	L	I	OXGR1	F	H
CCR6	F	N	GPR55	F	H	OXTR	Y	F
CCR7	F	N	GPR6	F	A	P2RY1	F	H
CCR8	L	N	GPR61	F	F	P2RY10	F	H
CCR9	F	N	GPR62	F	G	P2RY11	C	H
CCRL2	L	N	GPR63	F	T	P2RY12	F	H
CHRM1	F	N	GPR65	Y	H	P2RY13	F	H
CHRM2	F	N	GPR68	F	H	P2RY14	F	H
CHRM3	F	N	GPR75	F	G	P2RY2	F	H
CHRM4	F	N	GPR78	F	V	P2RY4	F	H
CHRM5	F	N	GPR82	I	S	P2RY6	F	H
CMKLR1	F	H	GPR83	Y	N	P2RY8	F	N
CMKLR2	Y	H	GPR85	F	L	PARD3	F	N
CNR1	T	L	GPR87	F	H	PAWR	C	N
CNR2	I	L	GPR88	L	V	PRLHR	Y	H
CX3CR1	F	N	GRPR	Y	H	PROKR1	F	Y
CXCR1	F	N	HCAR1	F	V	PROKR2	F	Y
CXCR2	F	N	HCAR2	F	V	PTAFR	F	H
CXCR3	F	H	HCAR3	F	V	PTGDR	L	I
CXCR4	L	Y	HCRTR1	Y	S	PTGDR2	F	H
CXCR5	F	H	HCRTR2	Y	S	PTGER1	V	L
CXCR6	F	N	HRH1	F	F	PTGER2	L	T
CYSLTR1	F	H	HRH2	F	F	PTGER3	G	L
CYSLTR2	C	H	HRH3	F	T	PTGER4	S	V
DRD1	F	F	HRH4	F	S	PTGFR	L	L
DRD2	F	F	HTR1A	F	F	PTGIR	L	T
DRD3	F	F	HTR1B	F	F	QRFPR	F	H
DRD4	F	F	HTR1D	F	F	RHO	F	A
DRD5	F	F	HTR1E	F	F	RRH	F	S
EDNRA	F	H	HTR1F	F	F	RXFP1	N	F
EDNRB	F	H	HTR2A	F	F	RXFP2	N	F
F2R	F	N	HTR2B	F	F	RXFP3	F	Q
F2RL1	F	N	HTR2C	F	F	RXFP4	F	H
FFAR1	F	N	HTR4	F	F	S1PR1	F	F
FFAR2	F	N	HTR5A	F	F	S1PR2	F	F
FFAR3	F	N	HTR6	F	F	S1PR3	F	F
FFAR4	F	I	HTR7	F	F	S1PR4	F	F
FPR1	F	Q	KISS1R	Y	Q	S1PR5	F	F
FPR2	F	Q	LGR4	N	A	SSTR1	F	Y
FPR3	F	E	LGR5	N	A	SSTR2	F	Y
FSHR	N	S	LGR6	N	A	SSTR3	F	Y
GALR1	Y	H	LHCGR	N	S	SSTR4	F	Y
GALR2	Y	H	LPAR1	N	L	SSTR5	F	F
GALR3	Y	H	LPAR2	S	Q	SUCNR1	F	H
GHSR	F	H	LPAR3	N	L	TAAR1	F	F
GNRHR	F	Y	LPAR4	F	N	TAAR2	F	F
GPBAR1	L	V	LPAR5	F	N	TAAR3P	F	F
GPER	F	N	LPAR6	F	N	TAAR5	F	T
GPR101	I	C	LTB4R	F	H	TAAR6	F	S
GPR119	G	L	LTB4R2	F	H	TAAR8	F	T
GPR12	F	T	MAS1	V	R	TAAR9	F	L
GPR132	F	H	MAS1L	I	S	TACR1	Y	H
GPR135	Y	C	MC1R	F	F	TACR2	Y	H
GPR139	Y	I	MC2R	F	V	TACR3	Y	H
GPR141	A	Q	MC3R	F	F	TBXA2R	G	L
GPR142	Y	V	MC4R	F	F	TRHR	Y	R
GPR146	Y	Y	MC5R	F	F	TSHR	N	S
GPR148	G	S	MCHR1	F	Y	UTS2R	I	W
GPR149	F	M	MCHR2	F	H	XCR1	F	N

Supplementary Table 8: Sequence alignment of 285 non-olfactory class A GPCRs of 2 residues, 5.47 and 6.52. Boxes highlighted in orange or brown represent identical or similar residues at 5.47 respectively, boxes highlighted in sky blue or dark blue mean identical or similar residues at 6.52 respectively. Sequence alignment was performed on GPCRdb (https://gpcrdb.org/).

Supplementary Figures

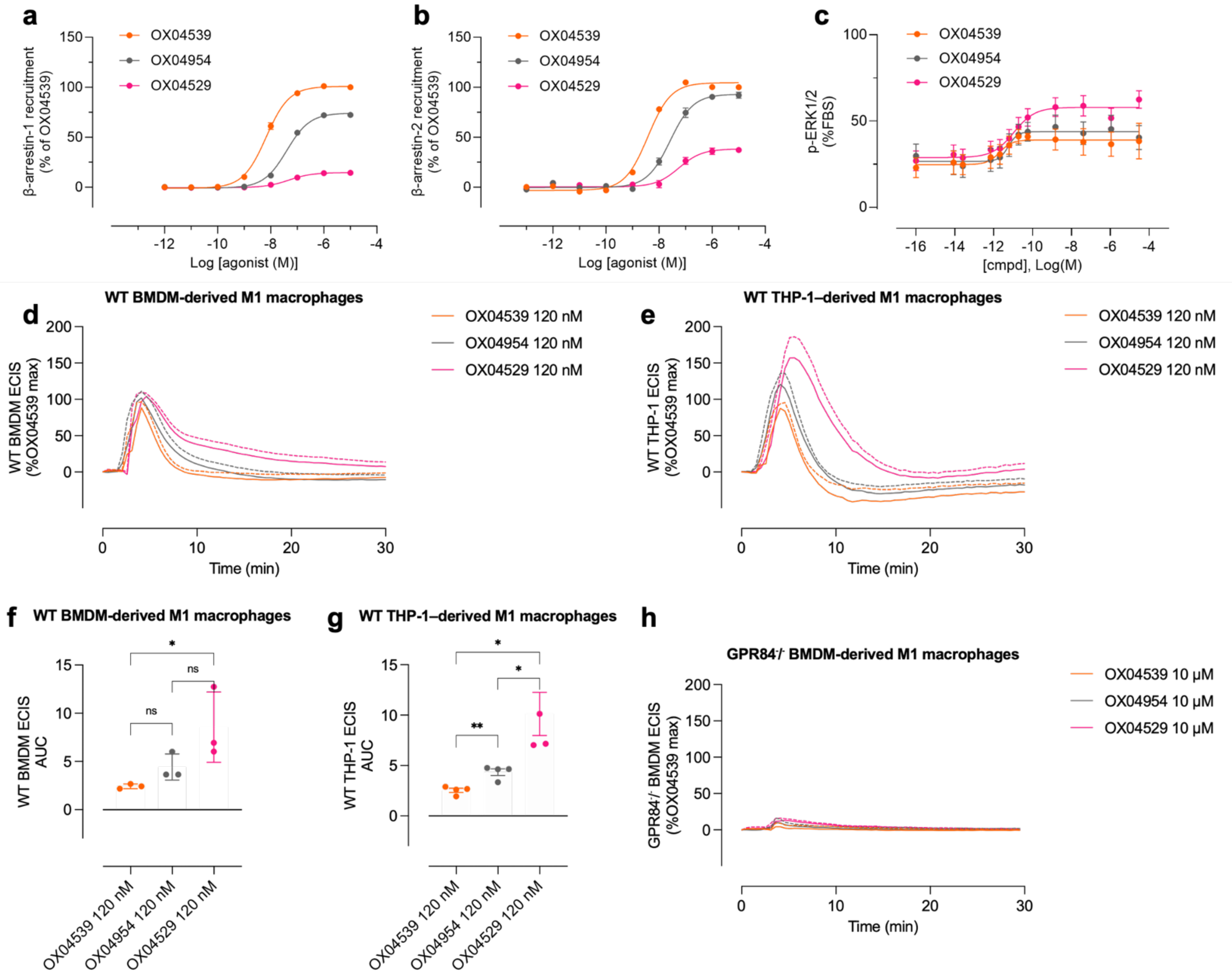

Supplementary Fig. 1: Additional functional characterization of OX04529, OX04954 and OX04539 across β-arrestin recruitment, ERK1/2 phosphorylation and impedance-based macrophage assays. Interactions between GPR84 and either (a), β-arrestin-2 or (b), β-arrestin-1 produced by various concentrations of the indicated ligands are shown. Data represent means ± S.E.M. from at least three biologically independent experiments. (c), Phosphorylation of ERK1/2 detected after 5 min incubation of the indicated agonists. Data represent means ± S.E.M. from three biologically independent experiments. Electric cell-substrate impedance sensing assays at tested at 120 nM (~EC_{90}) (d), WT BMDM-derived M1 macrophages, (e), THP-1 derived M1 macrophages or (h), $GPR84^{-/-}$ BMDM-derived M1 macrophages (tested up to 10 μM). (f) and (g) compare the area under the curve between each compound at corresponding cell lines. $*p < 0.05$, $**p < 0.01$. One-way ANOVA followed by Tukey's multiple comparison test.

Source data are provided within the Source Data file.

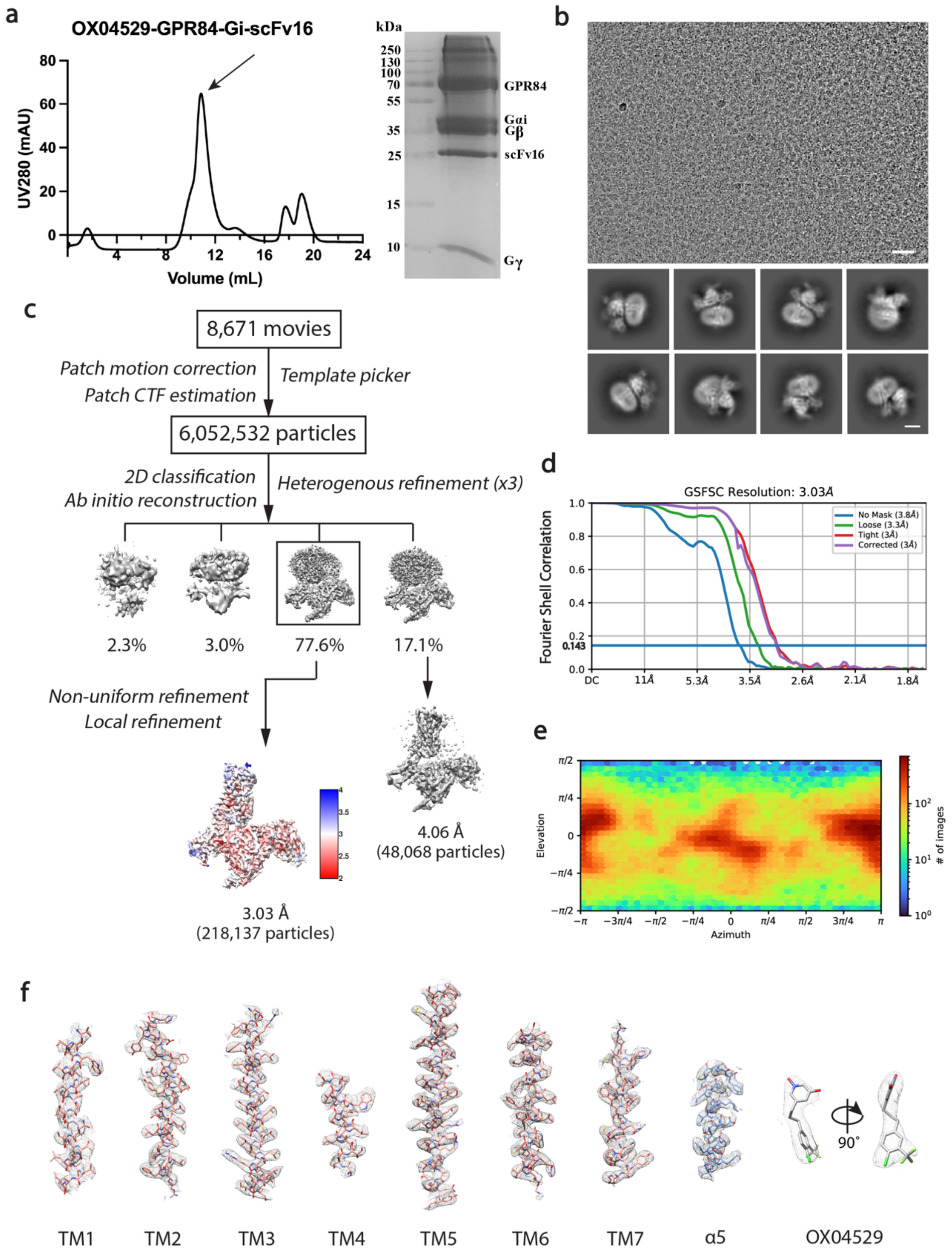

Supplementary Fig. 2: Purification of the OX04529-GPR84-G$_i$ complex and cryo-EM data processing. **(a)** Size-exclusion chromatography profile and SDS-PAGE analysis of the purified OX04529-GPR84-G$_i$ complex. **(b)** Representative cryo-EM micrograph (scale bar: 50 nm) and 2D class averages (scale bar: 5 nm). **(c)** Cryo-EM image processing workflow for the OX04529-GPR84-G$_i$ complex. Density map according to local resolution estimation. **(d)** Gold-standard Fourier shell correlation (FSC) curve showing an overall resolution is 3.03 Å at FSC=0.143. **(e)** Angular distribution of the particles used in the final reconstruction. **(f)** Cryo-EM density maps and models of the seven transmembrane helices (TM1-7), α5 helix of G$_i$ and the ligand of OX04529 bound GPR84-G$_i$ complex are shown.

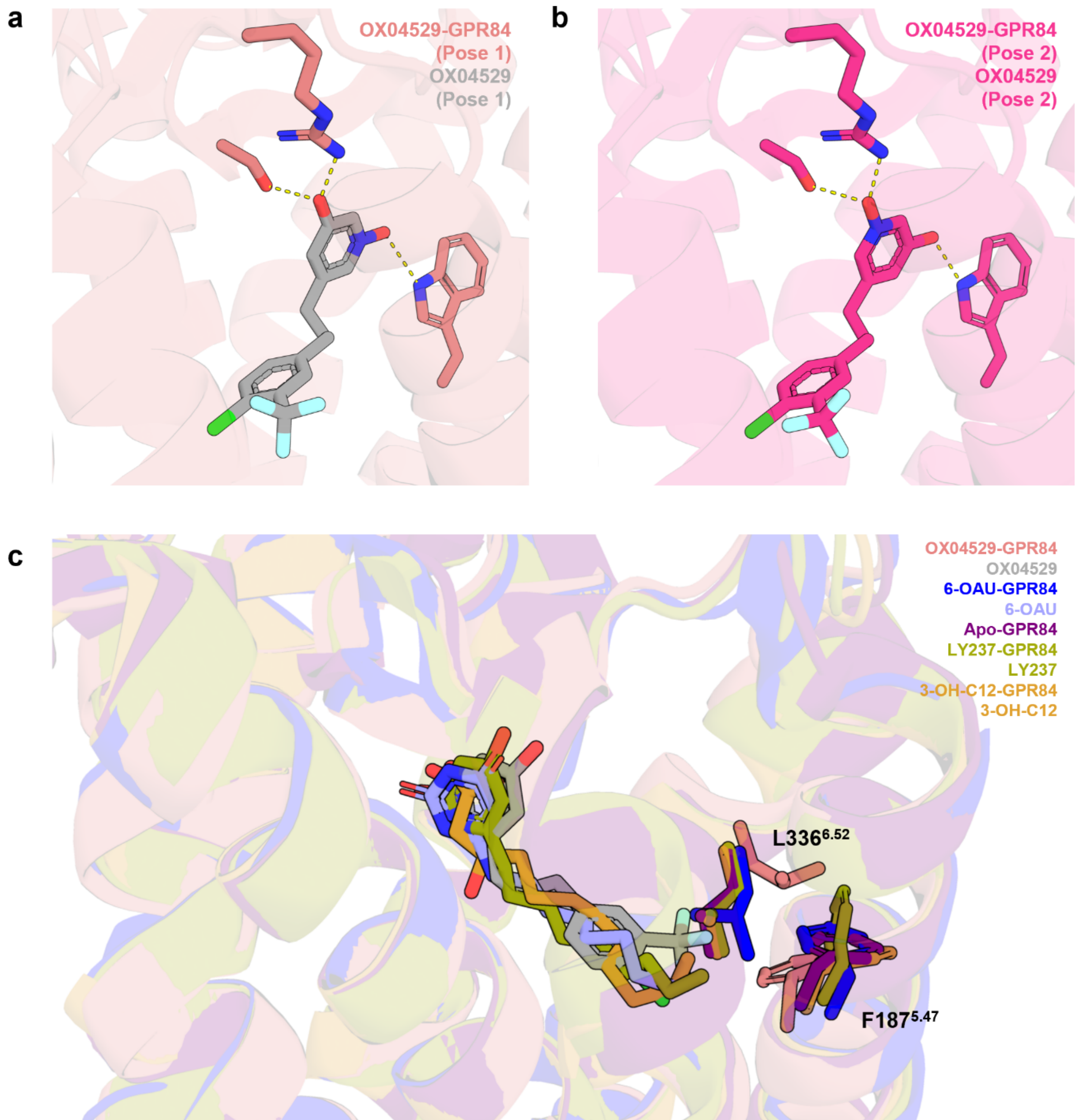

Supplementary Fig. 3: Alternative binding poses of the OX04529 *N*-oxide and overlay of reported cryo-EM structures at L336 and F187.

a, b, Two alternative orientations of the N-oxide moiety in the binding pocket. In Pose 1 **(a)**, the *N*-oxide group is oriented toward W360, with the hydroxyl group directed toward R172 and S169. In Pose 2 **(b)**, the orientation is reversed. **c,** Overlay of OX04529-GPR84, 6-OAU-GPR84, apo-GPR84, LY237-GPR84 and 3-OH-C12-GPR84 structures zooming at L336 and F187.

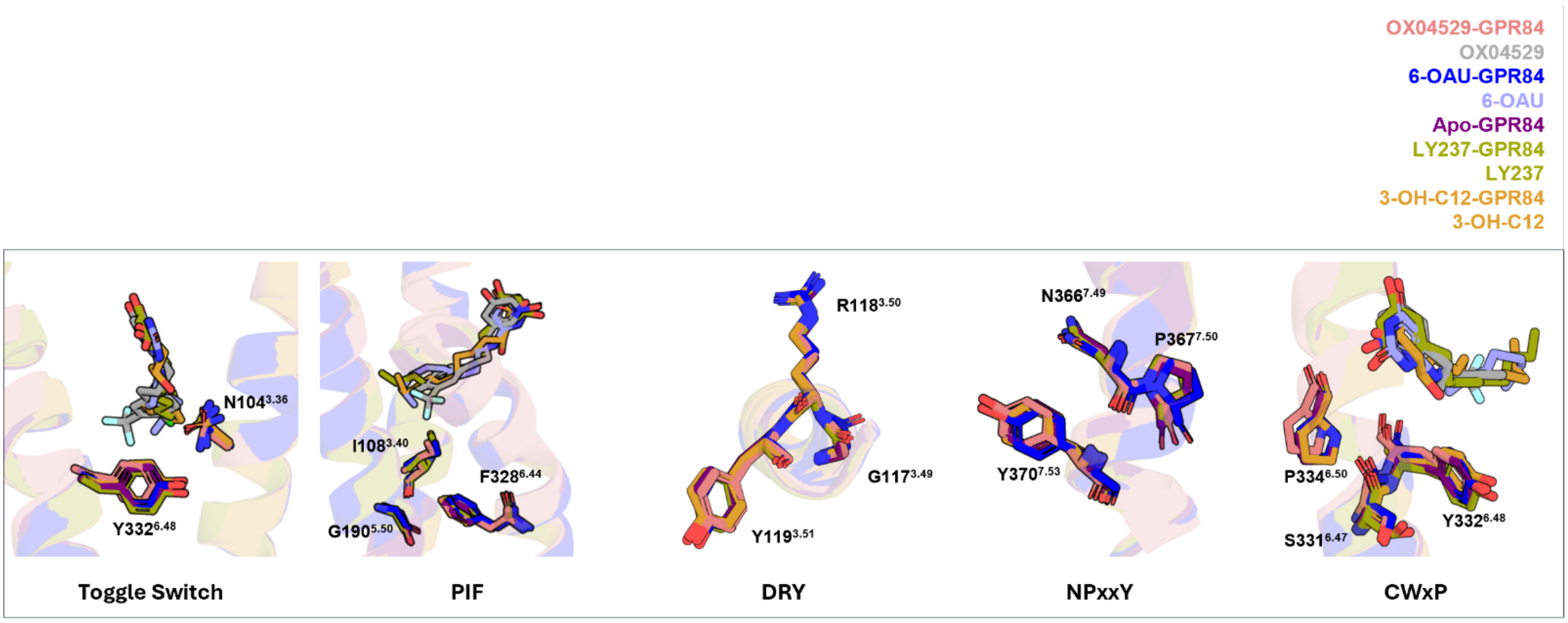

Supplementary Fig. 4: Structural comparison with other GPR84 cryo-EM co-structures.

Overlay of OX04529-GPR84, 6-OAU-GPR84, apo-GPR84, LY237-GPR84 and 3-OH-C12-GPR84 structures revealed that key conserved motifs in class A GPCRs, involved in G protein activation including the toggle switch, PIF (GIF) motif, DRY (GRY) motif, NPxxY motif and CWxP (SYxP) motif remain unchanged.

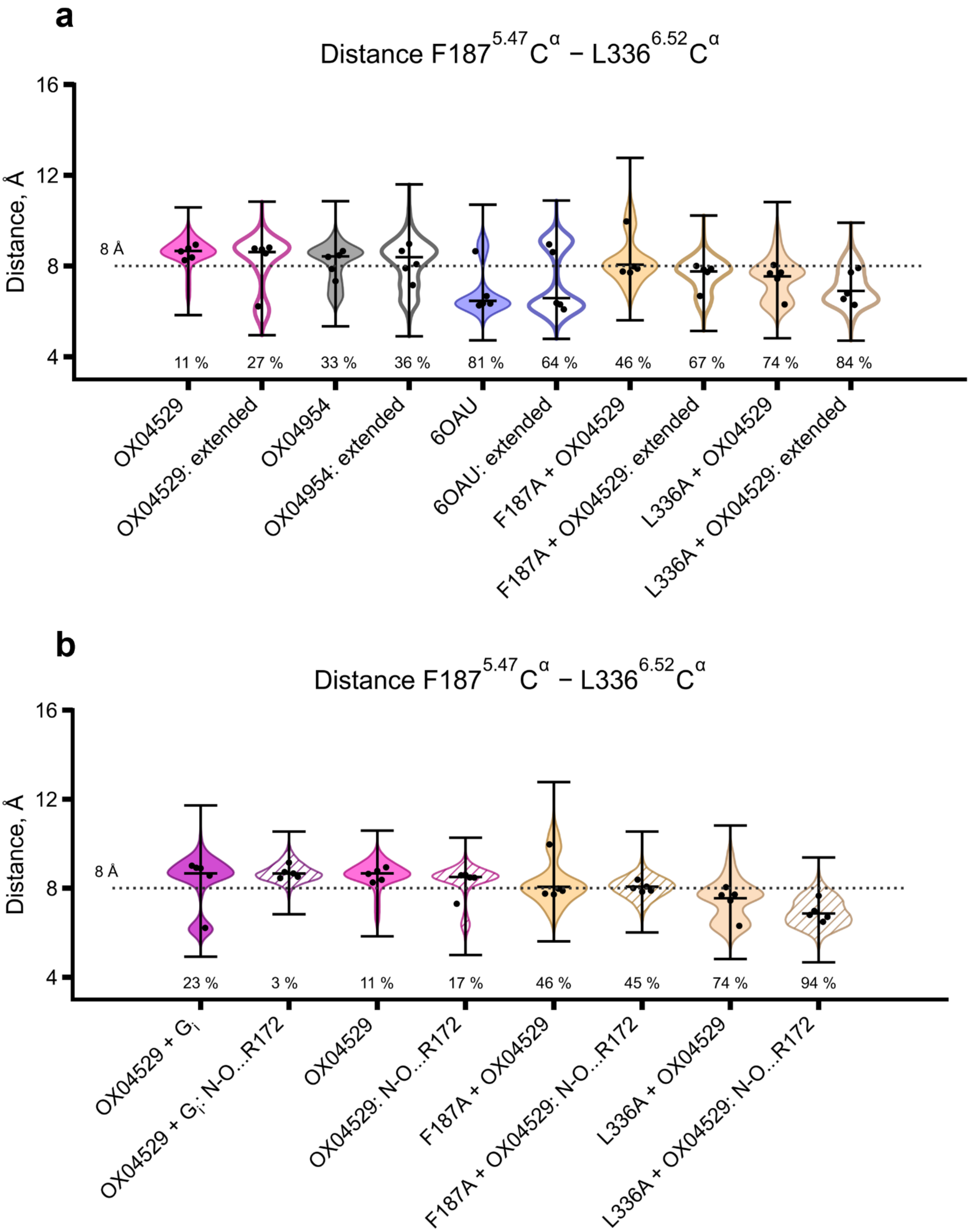

Supplementary Fig. 5: Validation of Phe187$^{5.47}$-Leu336$^{6.52}$ separation mechanism across simulation conditions and ligand orientations

a. Violin plots comparing Cα-Cα distance distributions from 1-µs (5 replicates) and 2-µs (5 replicates) MD simulations. The dashed line at 8 Å separates compact and extended conformational states. Percentages indicate frames below threshold. Extended sampling confirms that OX04529 maintains the separated state (11-27% compact) while β-arrestin recruiting agonists (6-OAU) favor compaction

(64-81% compact). Mutants F187A and L336A consistently restore the compact state (67-84% compact) across both timescales, validating the robustness of the steric mechanism independent of simulation length. This graph shows that both setups (forcefield, protonation, specific receptor model) give same results within the 1st microsecond, and then their results start showing deeper differences.

b. Violin plots comparing Cα-Cα distance distributions between two possible orientations of the OX04529 3-hydroxypyridine *N*-oxide ring system from 1-μs MD simulations (5 replicates). System names with '+G_i' indicates simulations with G protein, system names containing 'N-O…R172' indicate simulations with the pyridine *N*-oxide oxygen interacting with Arg172^{ECL2}, and systems labeled with a just ligand name indicates simulations with the hydroxyl group interacting with Arg172^{ECL2}. The dashed line at 8 Å separates compact and extended states. Percentages indicate frames below threshold. Both orientations of OX04529 maintain the separated conformation (3-23% compact), while mutants Phe187Ala and Leu336Ala restore compaction (74-94% compact) regardless of head group orientation, demonstrating that the steric mechanism is independent of ligand binding pose ambiguity.

Source data are provided within the Source Data file.

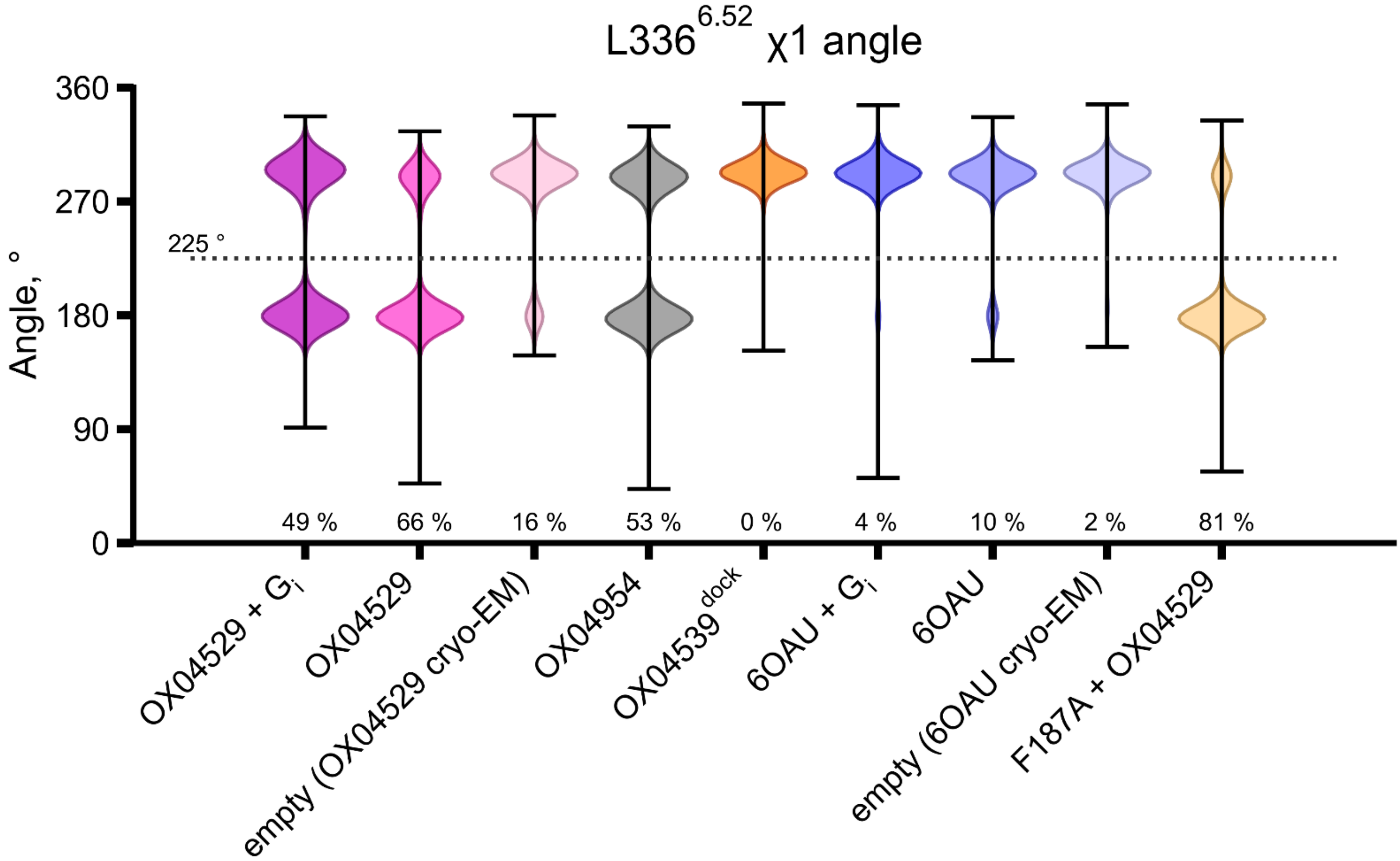

Supplementary Fig. 6: Violin plots illustrate the distribution of χ1 dihedral angles of Leu336$^{6.52}$ obtained from five replicate 1-μs MD simulation trajectories. The analysis compares distinct rotameric states in GPR84 complexes with G protein-biased ligand OX04529, partially biased OX04954 and reference agonists (OX04539 and 6-OAU), two ligand-free receptor conformations derived from cryo-EM structures, and the Phe187$^{5.47}$Ala mutant.

Source data are provided within the Source Data file.

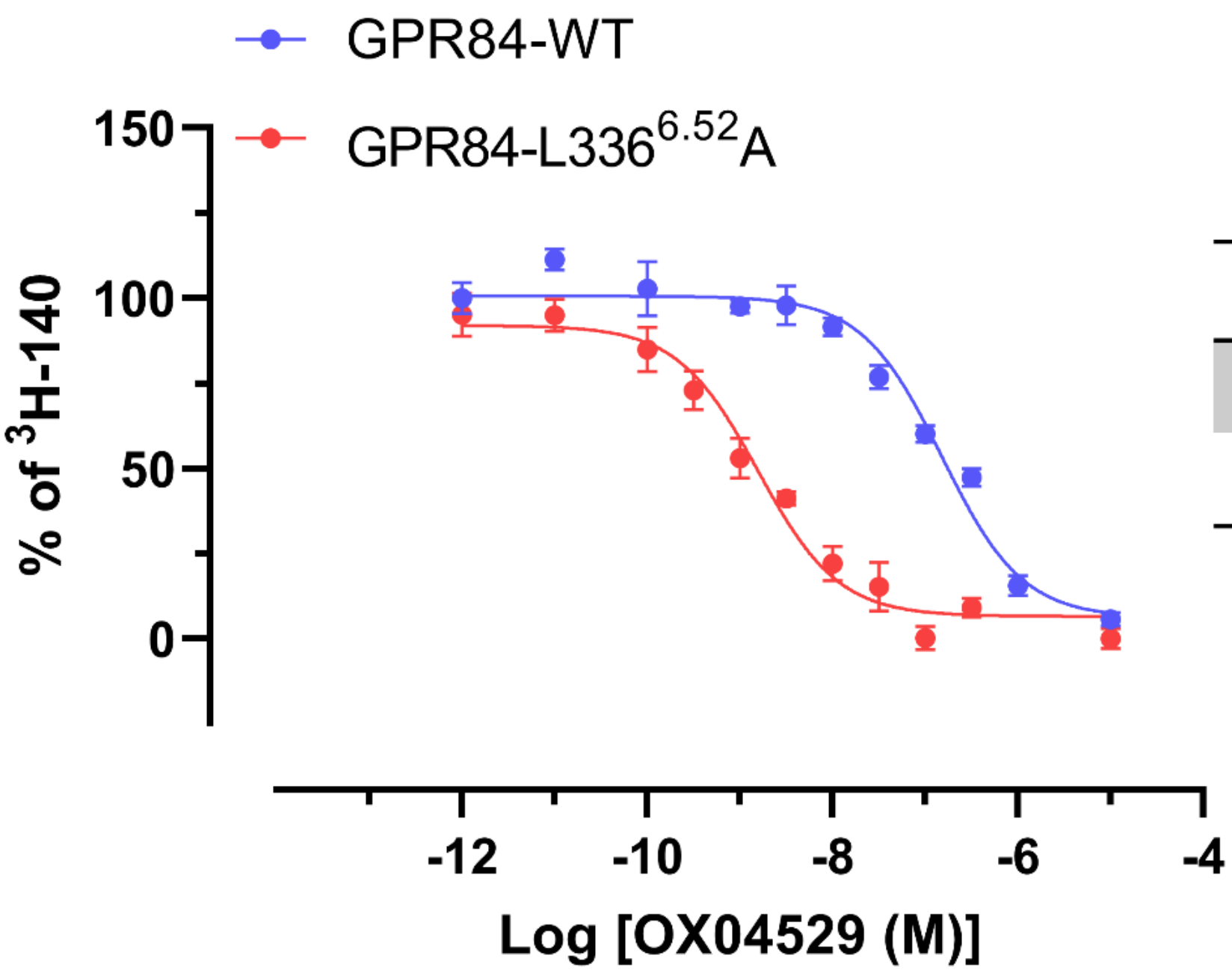

	pKi
WT	7.29 ± 0.05
L336A	8.82 ± 0.19

Supplementary Fig. 7: The affinity of OX04529 is markedly increased at Leu^{336}Ala GPR84 in competition binding studies using stable cell lines expressing the indicated GPR84-G$_{i2}$ fusion protein

Left, data from a representative experiment, right pKi represents means +/- S.E.M. from three independent biological replicates

Source data are provided within the Source Data file.

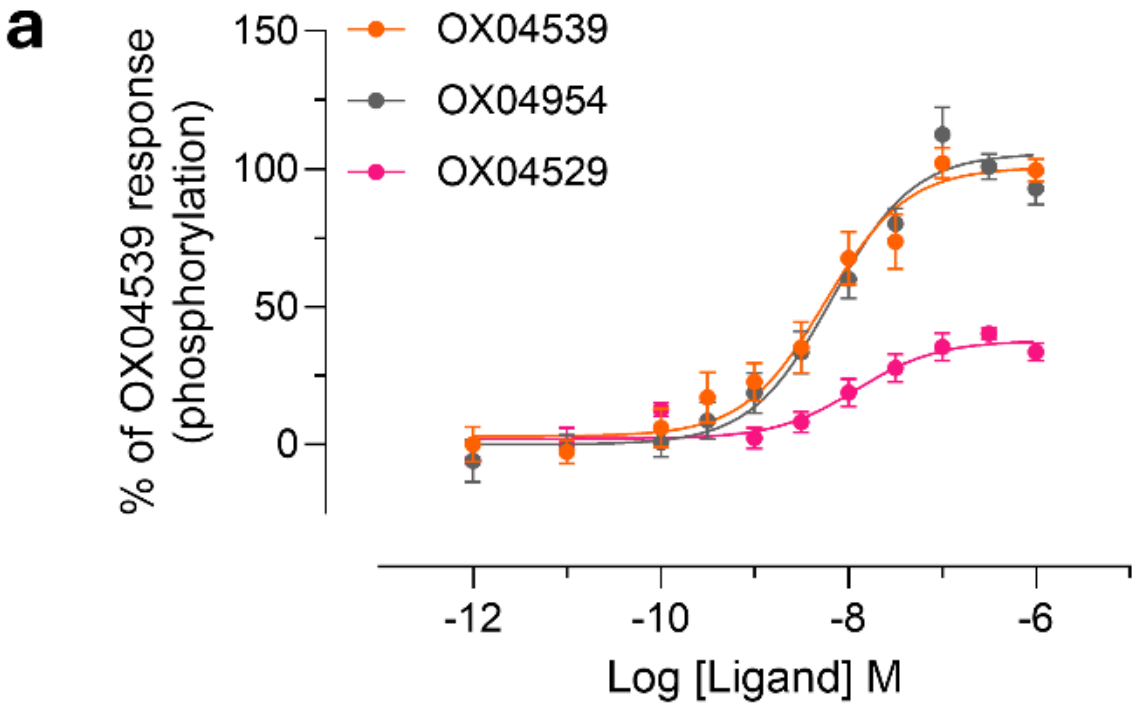

	pEC50 ± SEM
**OX04539**	8.30 ± 0.24
**OX04954**	8.12 ± 0.04
**OX04529**	7.91 ± 0.26

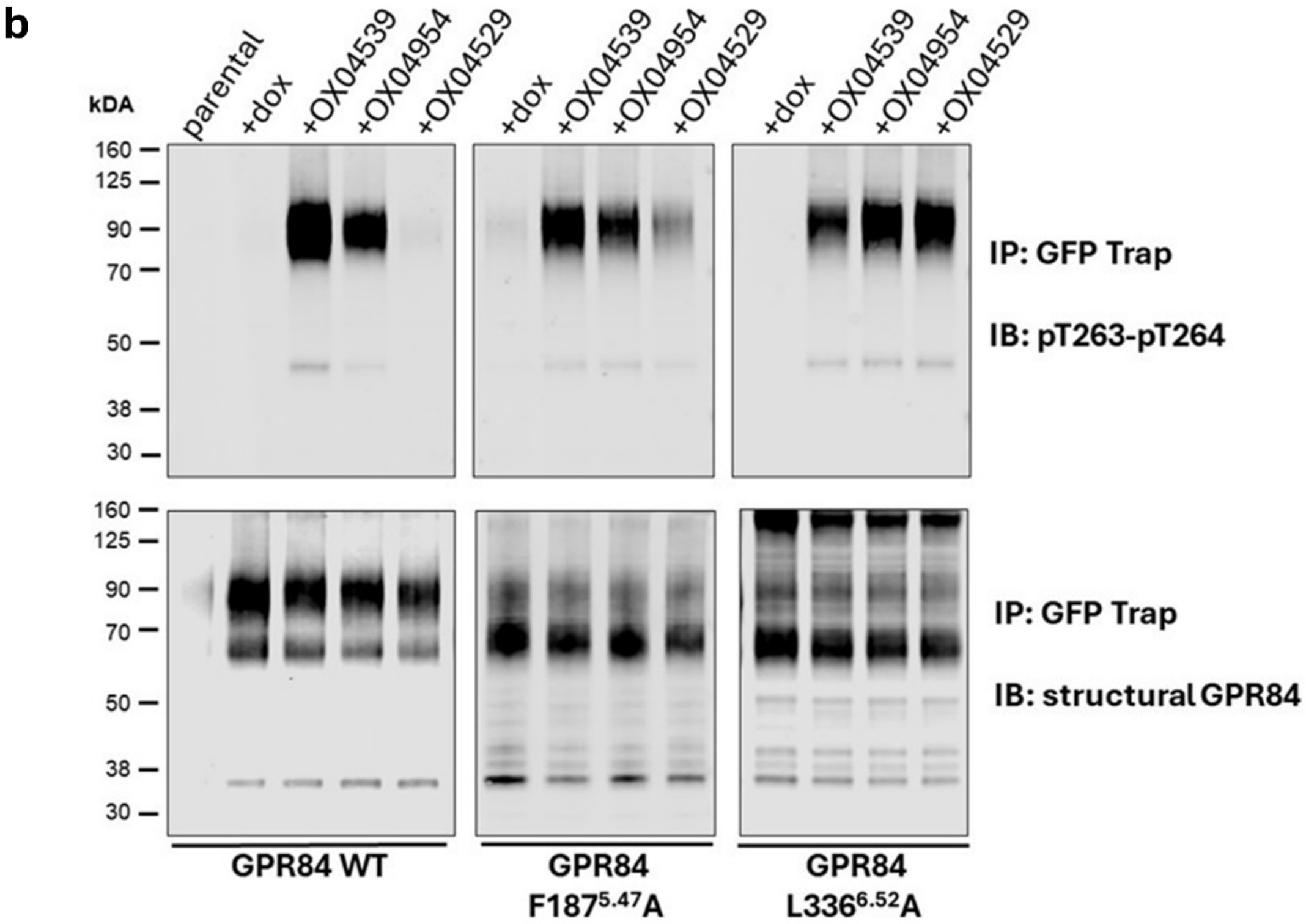

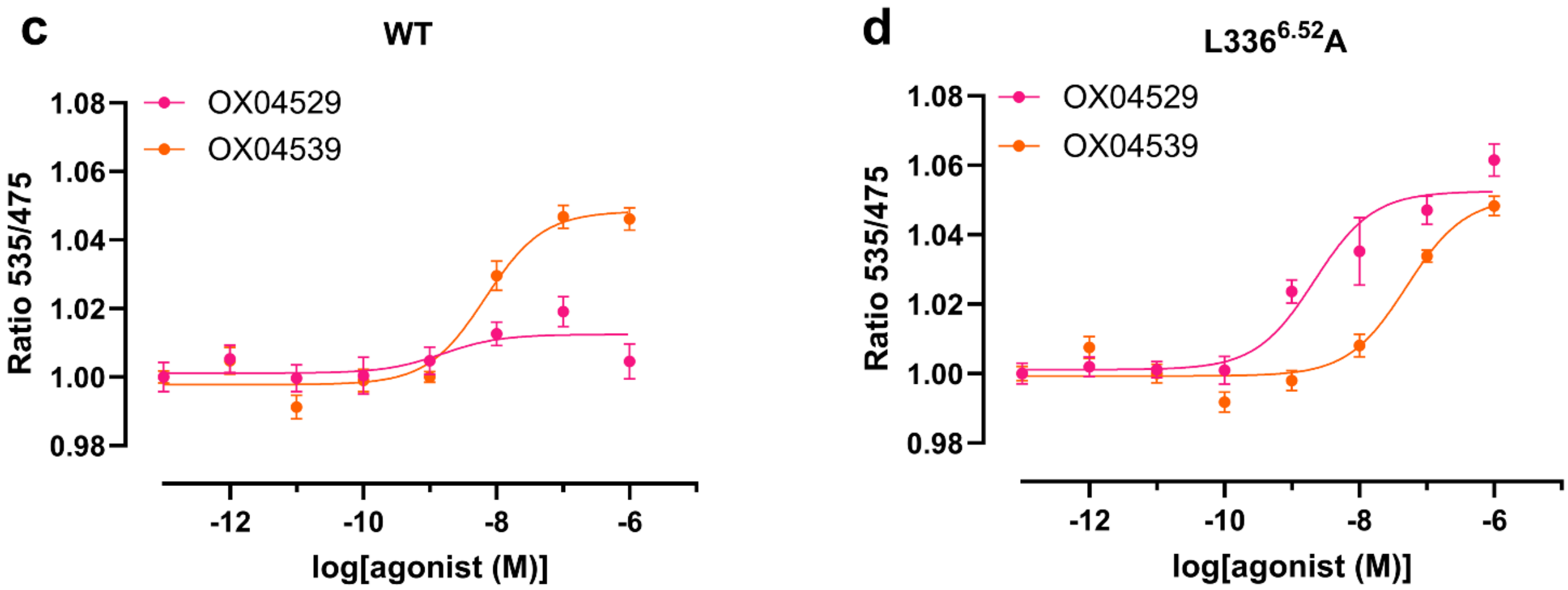

	WT pEC50 ± SEM	L336$^{6.52}$A pEC50 ± SEM
**OX04539**	8.22 ± 0.10	7.41 ± 0.07
**OX04529**	8.28 ± 0.54	8.95 ± 0.14

Supplementary Fig. 8: Biased agonist-induced phosphorylation of Thr263^{ICL3} and Thr264^{ICL3} in GPR84 is modulated by mutation of Phe187$^{5.47}$ and Leu336$^{6.52}$

a, The ability of varying concentrations of OX04529, OX04539 and OX04954 to promote phosphorylation of residues Thr263^{ICL3} and Thr264^{ICL3} of wild type GPR84 was assessed in a magnetic bead capture assay.

b, Immunoblots show the lack of basal phosphorylation of residues Thr263^{ICL3} and Thr264^{ICL3} in each of wild type, Phe187$^{5.47}$Ala and Leu336$^{6.52}$Ala GPR84. The ability of OX04529, OX04539 and OX04954 (1 μM) to promote phosphorylation at these sites is illustrated. A representative example of 4 independent experiments is displayed.

c, OX04539 can induce internalization of wild type GPR84 receptor, while OX04529 is much less effective.

d, OX04529 and OX04539 induce equivalent receptor internalization at Leu336$^{6.52}$Ala GPR84.

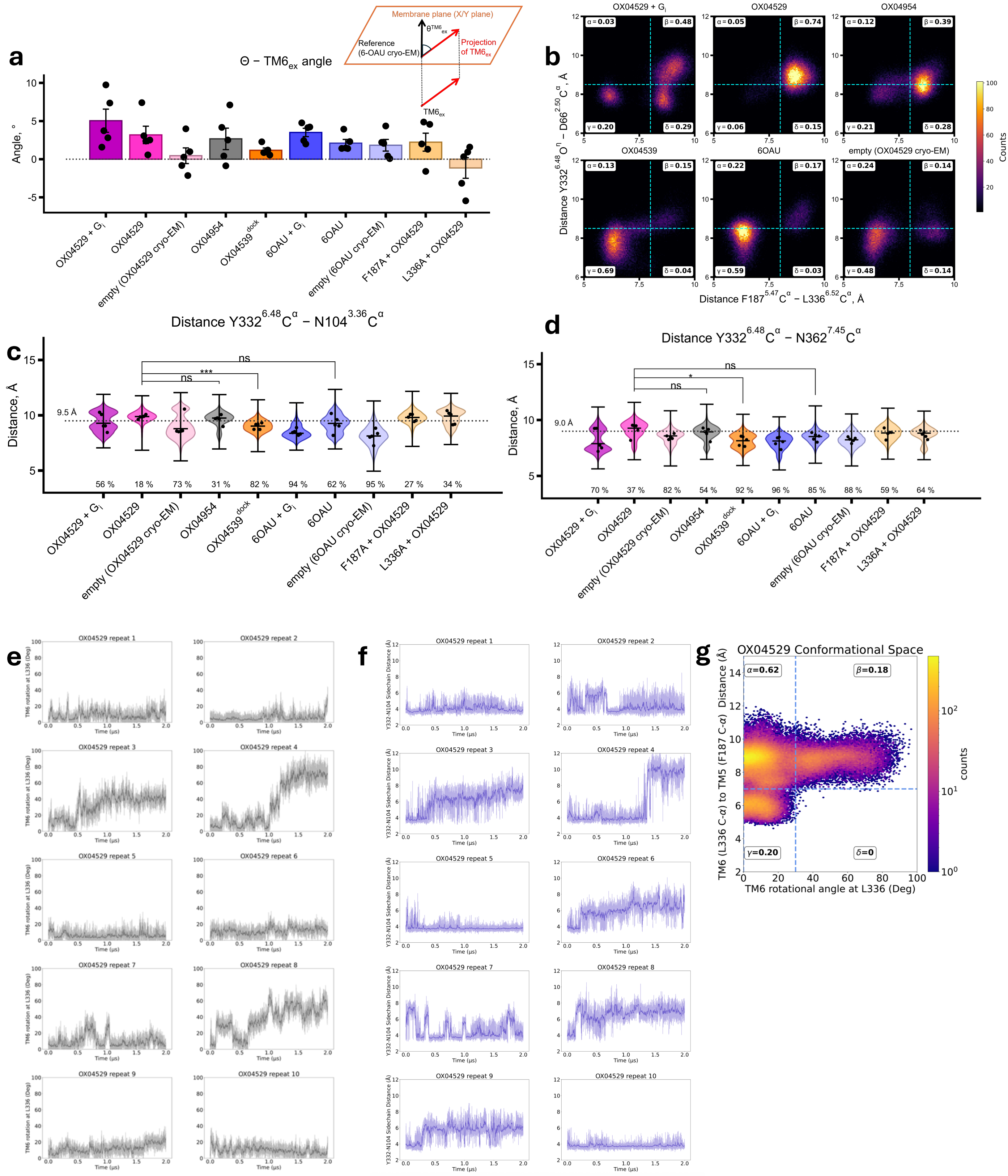

Supplementary Fig. 9: Comprehensive analysis of TM6 conformational dynamics and polar network disruption in OX04529-bound GPR84

a. Bar graph showing the angular deviation of the TM6 extracellular segment across different GPR84 systems from five replicate 1-μs MD simulation trajectories, measured relative to the 6-OAU cryo-EM reference structure. The inset diagram illustrates the measurement method: angles represent the projection of vectors (connecting first-to-last helical turn Cα centers of mass in TM6) onto the membrane plane. Note the significant positive angle in OX04529-bound systems, indicating outward TM6 movement away from TM5 toward the

lipid interface, contrasting with the more neutral or negative angles observed with β-arrestin recruiting agonists. This conformational difference is enhanced when OX04529 is co-bound with G protein and is disrupted in the Phe187Ala and Leu336Ala mutants.

b. Two-dimensional density plots showing the correlation between TM6 extracellular rotation angle and Phe187$^{5.47}$-Leu336$^{6.52}$ Cα distance across five replicate 1-μs MD simulation trajectories. Each panel represents a different system as labelled, with the proportion of simulation frames in each quadrant indicated by percentages. The dashed lines at 7 Å (vertical) and 30° (horizontal) demarcate threshold values separating distinct conformational states. OX04529-bound systems show preferential occupancy of the high-distance, high-rotation quadrant, while reference agonists (OX04539, 6-OAU) predominantly occupy the low-distance, low-rotation region.

c. Distribution of Tyr332$^{6.48}$-Asn104$^{3.36}$ hydrogen bond distances across five replicate 1-μs MD simulations. The dashed line at ~9.5 Å indicates the hydrogen bonding threshold. Percentages represent the proportion of frames maintaining hydrogen bonding. OX04529 shows the lowest maintenance (56%), demonstrating disruption of this critical toggle-switch interaction. ns = not significant, *** $p < 0.001$, 2-tailed equal-variance t-test.

d. Distribution of Tyr332$^{6.48}$-Asn362$^{7.45}$ hydrogen bond distances across the same simulation conditions. The dashed line at ~9.0 Å indicates the hydrogen bonding threshold. OX04529 shows reduced stability (70%) compared to most other systems (54-96%), contributing to polar network disruption. ns = not significant, * $p < 0.05$, 2-tailed equal-variance t-test.

e. Individual time-series traces of TM6 rotation angles from ten replicate 2-μs simulations of OX04529, demonstrating the temporal evolution and variability of TM6 conformational changes.

f. Individual time-series traces of Tyr332$^{6.48}$-Asn104$^{3.36}$ distances from ten replicate 2-μs simulations of OX04529, showing the dynamic disruption of this key hydrogen bond over extended timescales. The measured distance is between the oxygen of Tyr332$^{6.48}$ and the C-gamma of Asn104$^{3.36}$. The quadrant labels in panels **e.** and **f.** represent the fraction of the total simulation frames in a given state of the receptor.

g. Two-dimensional correlation plot of TM6 rotation angle versus TM5-TM6 separation distance, demonstrating that significant TM6 rotation (>30°) occurs only when the TM5-TM6 separation exceeds 7 Å, establishing the coupling between these conformational changes.

Source data are provided within the Source Data file.

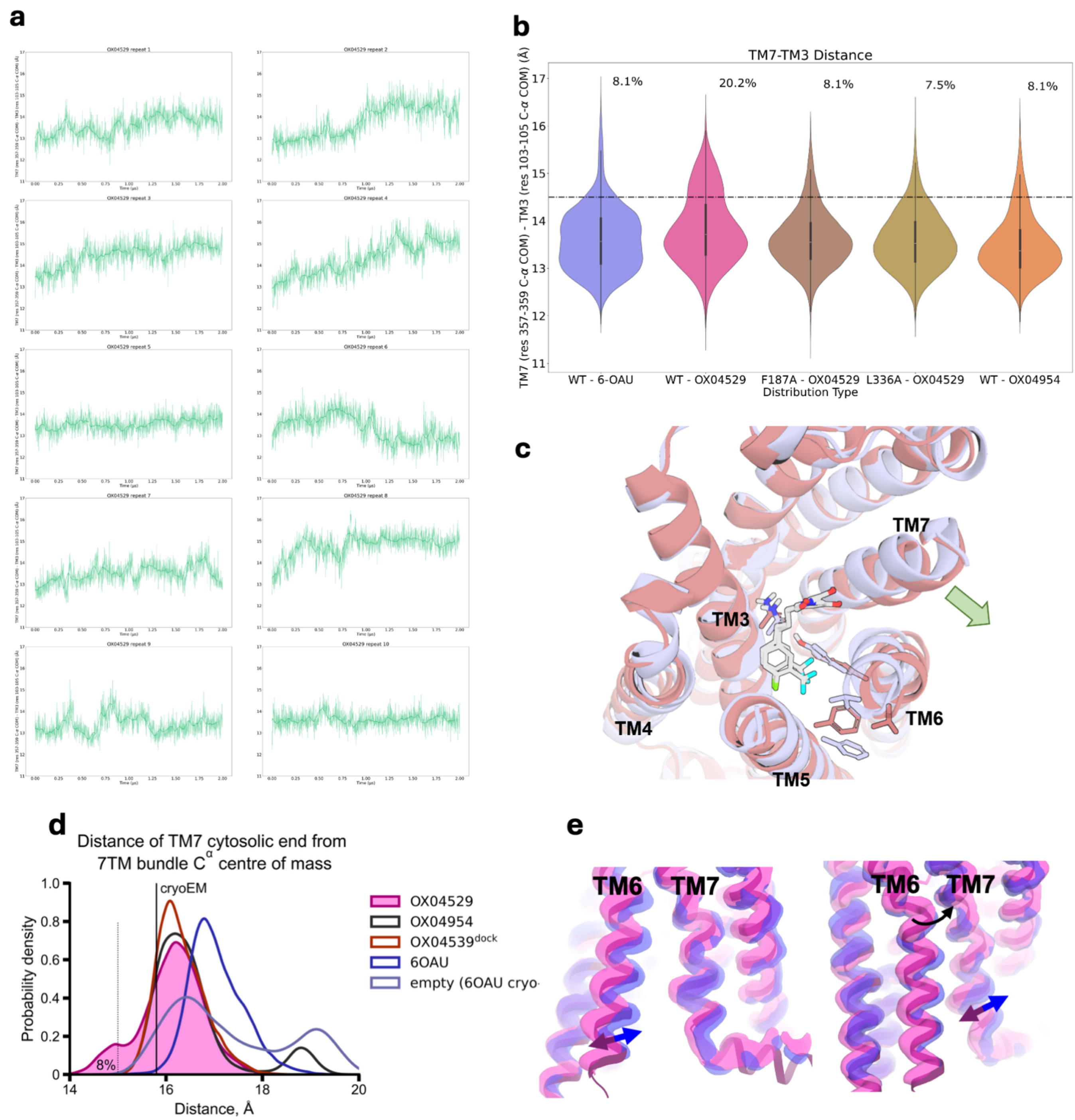

Supplementary Fig. 10: Distribution of TM7-TM3 distances obtained from 10 x 2 µs simulations.

a, Individual time-series traces of TM7-TM3 distances from ten replicate 2-µs simulations of OX04529.

b, Distribution of TM7-TM3 distances across ten replicate 2-µs MD simulations. Note OX04529 has a larger occupancy of higher distances.

c, Two overlaid representative snapshots from molecular dynamics simulations showing the binding poses of G protein-biased OX04529 (gray) in the GPR84 orthosteric pocket, and the outward movement of TM7.

d, Distance distribution of the TM7 cytosolic end from the 7TM bundle Cα centre of mass. OX04529 (magenta) stabilizes TM7 in a more inward position compared to 6-OAU (blue) and $OX04539_{dock}$ (orange),

with 8% of conformations showing reduced distance (~15 Å). Dashed line indicates the cryo-EM reference distance.

e, MD simulation density visualization of TM6 and TM7 movements. Semi-transparent surfaces show backbone occupancy (>75% of frames) for OX04529 (magenta) and 6-OAU (blue). OX04529 stabilizes an outward TM6 and inward TM7 conformation compared to 6-OAU. Arrows indicate helix displacement directions. Two views are shown to illustrate the coupled TM6-TM7 rearrangement.

a

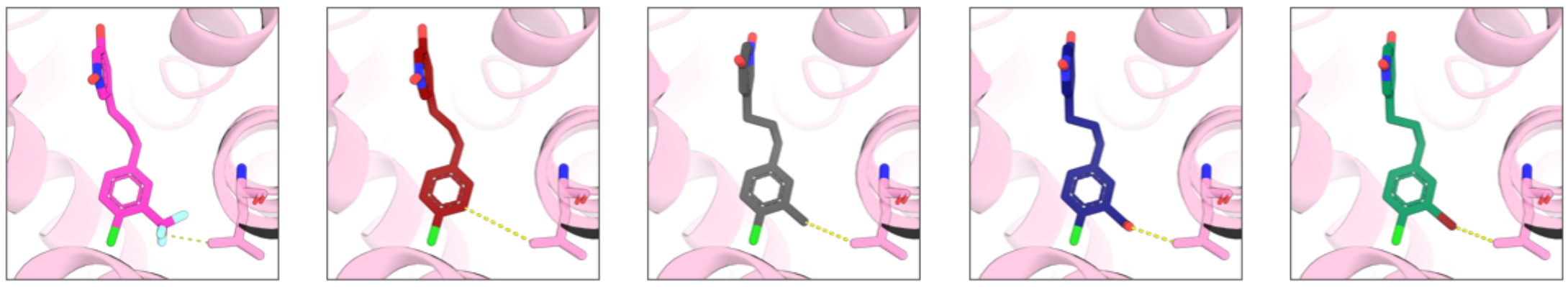

Agonist	OX04529	OX04540	OX04954	OX04955	OX04956
Aryl 3-substituent	CF_3	H	Me	MeOH	Br
Distance between L336$^{6.52}$ (Å)	3.7 (dock)	5.8	4.6	3.7	4.3
pEC_{50} ± SEM<br>%E_{max} ± SEM	10.92 ± 0.18<br>97 ± 0.2	10.69 ± 0.09<br>97 ± 0.7	10.90 ± 0.23<br>98 ± 1.8	10.45 ± 0.20<br>101 ± 1.5	11.04 ± 0.12<br>98 ± 2.1
pEC_{50} ± SEM<br>%E_{max}	ND<br>10 ± 1.5	6.96 ± 0.17<br>81 ± 5.3	7.03 ± 0.24<br>71 ± 1.3	6.58 ± 0.23<br>21 ± 2.9	6.86 ± 0.39<br>34 ± 6.0
$\Delta\Delta Log(E_{max}/EC_{50})$ ± SEM	ND	0.67 ± 0.09	0.86 ± 0.17	1.42 ± 0.15	1.5 ± 0.22
Bias Factor	ND	4.63	7.32	26.05	31.85
VDM-V (docking)	269.736	239.72	256.784	264.84	257.872
VDM-V (elements)	316.74	267.55	287.46	310.9	302.7
cLogP	3.9476	3.0993	3.4432	2.502	3.8245
LLE	6.9757	7.5907	7.4535	7.9447	7.2155

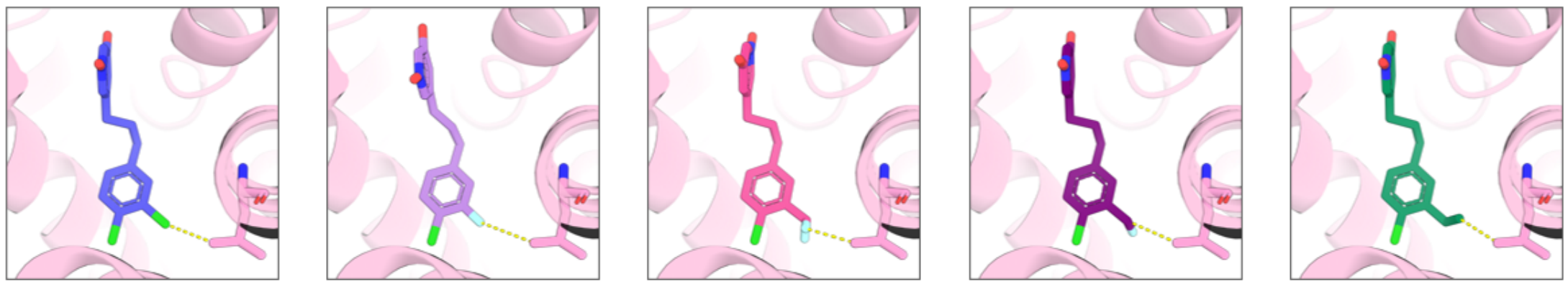

Agonist	OX04957	OX04958	OX04959	OX04960	OX04961
Aryl 3-substituent	Cl	F	CF2H	CH2F	Ethyl
Distance between L336$^{6.52}$ (Å)	4.4	4.7	3.6	4.4	3.7
pEC_{50} ± SEM<br>%E_{max} ± SEM	10.41 ± 0.11<br>102 ± 3.0	10.39 ± 0.13<br>99 ± 0.1	11.14 ± 0.25<br>95 ± 11.5	10.99 ± 0.15<br>107 ± 2.1	10.95 ± 0.26<br>103 ± 3.6
pEC_{50} ± SEM<br>%E_{max} ± SEM	6.68 ± 0.23<br>46 ± 5.8	7.15 ± 0.01<br>72 ± 1.3	6.82 ± 0.14<br>24 ± 2.3	6.35 ± 0.31<br>45 ± 1.2	7.40 ± 0.04<br>21 ± 0.3
$\Delta\Delta Log(E_{max}/EC_{50})$ ± SEM	0.95 ± 0.10	0.24 ± 0.06	1.78 ± 0.11	1.88 ± 0.17	1.1 ± 0.13
Bias Factor	9	1.74	60.32	75.55	12.56
VDM-V (docking)	255.256	244.152	265.728	261.04	273.56
VDM-V (elements)	284.54	273.15	311.45	307.34	306.79
cLogP	3.7053	3.2001	3.797	3.2031	3.8588
LLE	6.708	7.1899	7.3463	7.7902	7.0912

a, cont.

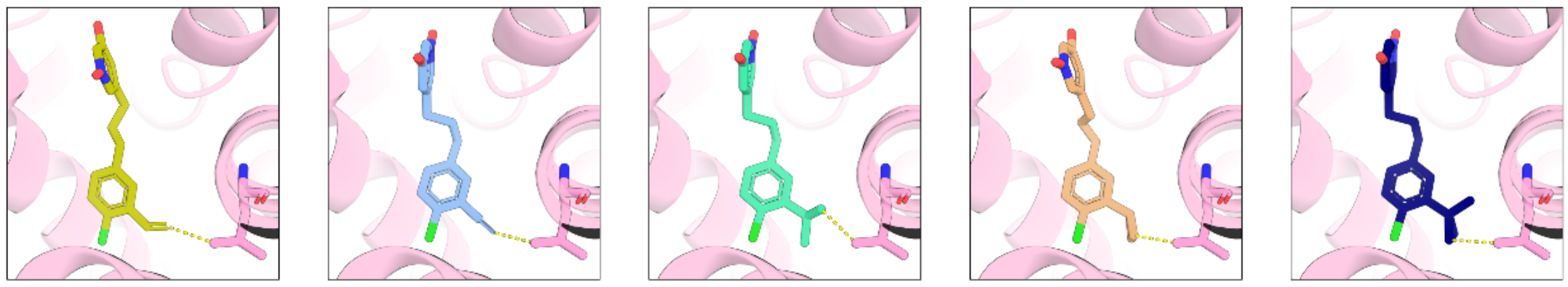

Agonist	OX04962	OX04963	OX04964	OX04965	OX04966
Aryl 3-substituent	Vinyl	Ethynyl	isopropyl	cyclopropyl	1-methyl cyclopropyl
Distance between L336$^{6.52}$ (Å)	3.8	3.7	3.7	3.8	3.6
pEC_{50} ± SEM	11.03 ± 0.32	11.19 ± 0.12	10.84 ± 0.31	11.40 ± 0.08	9.77 ± 0.21
$\%E_{max}$ ± SEM	97 ± 3.4	106 ± 3.8	94 ± 7.4	103 ± 3.7	107 ± 5.6
pEC_{50} ± SEM	6.75 ± 0.36	7.43 ± 0.18	ND	ND	ND
$\%E_{max}$	18 ± 2.2	15 ± 1.1	9 ± 1.4	9 ± 3.0	5 ± 1.5
$\Delta\Delta Log(E_{max}/EC_{50})$ ± SEM	1.89 ± 0.24	1.48 ± 0.12	ND	ND	ND
Bias Factor	76.96	30.24	ND	ND	ND
VDM-V (docking)	268.224	264.04	290.344	282.832	299.608
VDM-V (elements)	313.66	309.42	345.69	335.36	352.78
cLogP	3.8022	3.2005	4.2863	4.2101	3.9821
LLE	7.2311	7.9862	6.5504	7.1899	5.7836

b

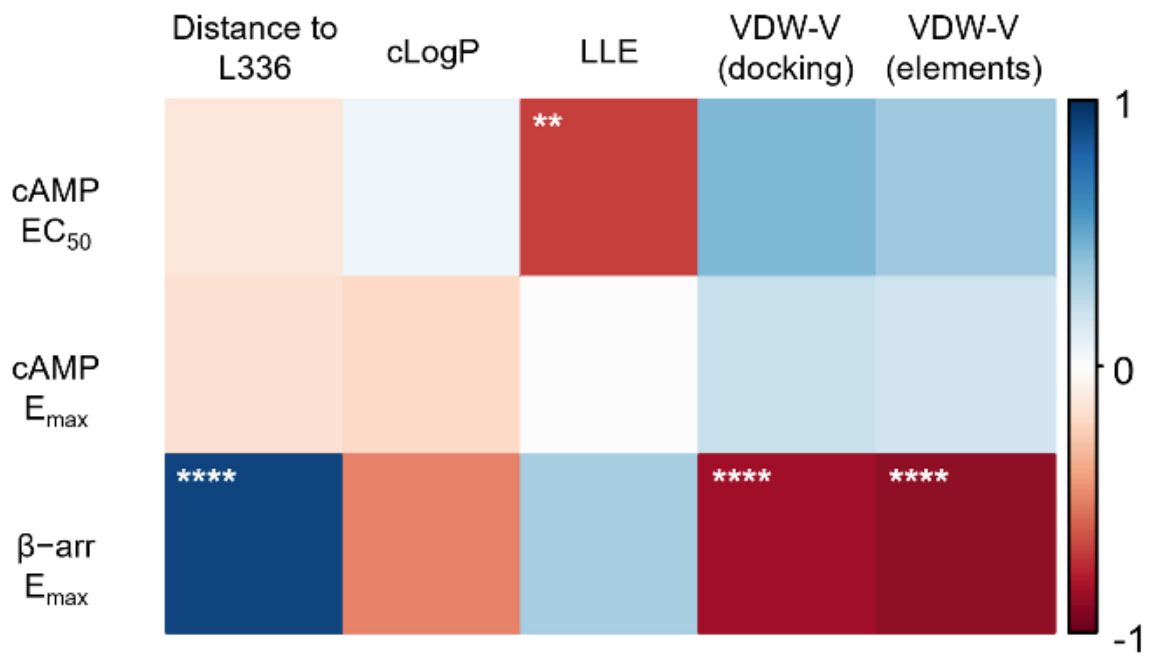

c

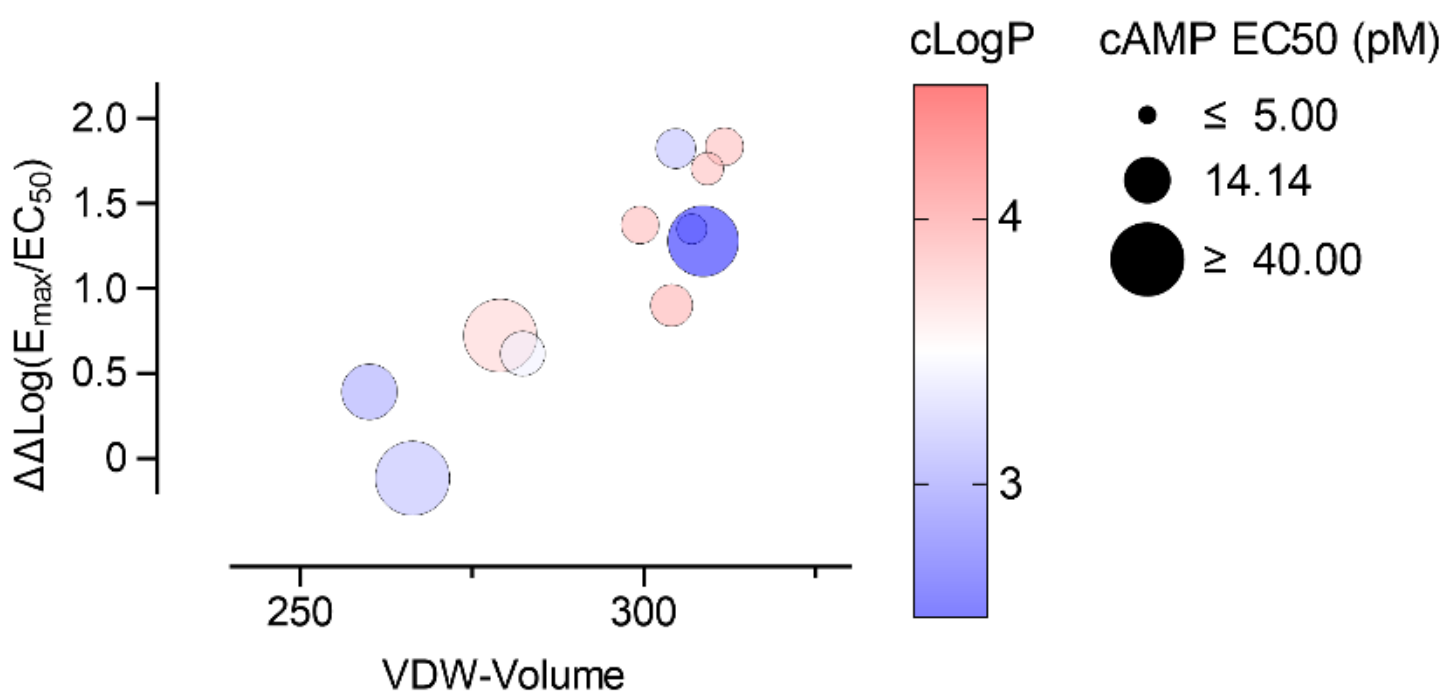

Correlation coefficient: 0.87
p-value: 0.00044***

d **e** **f**

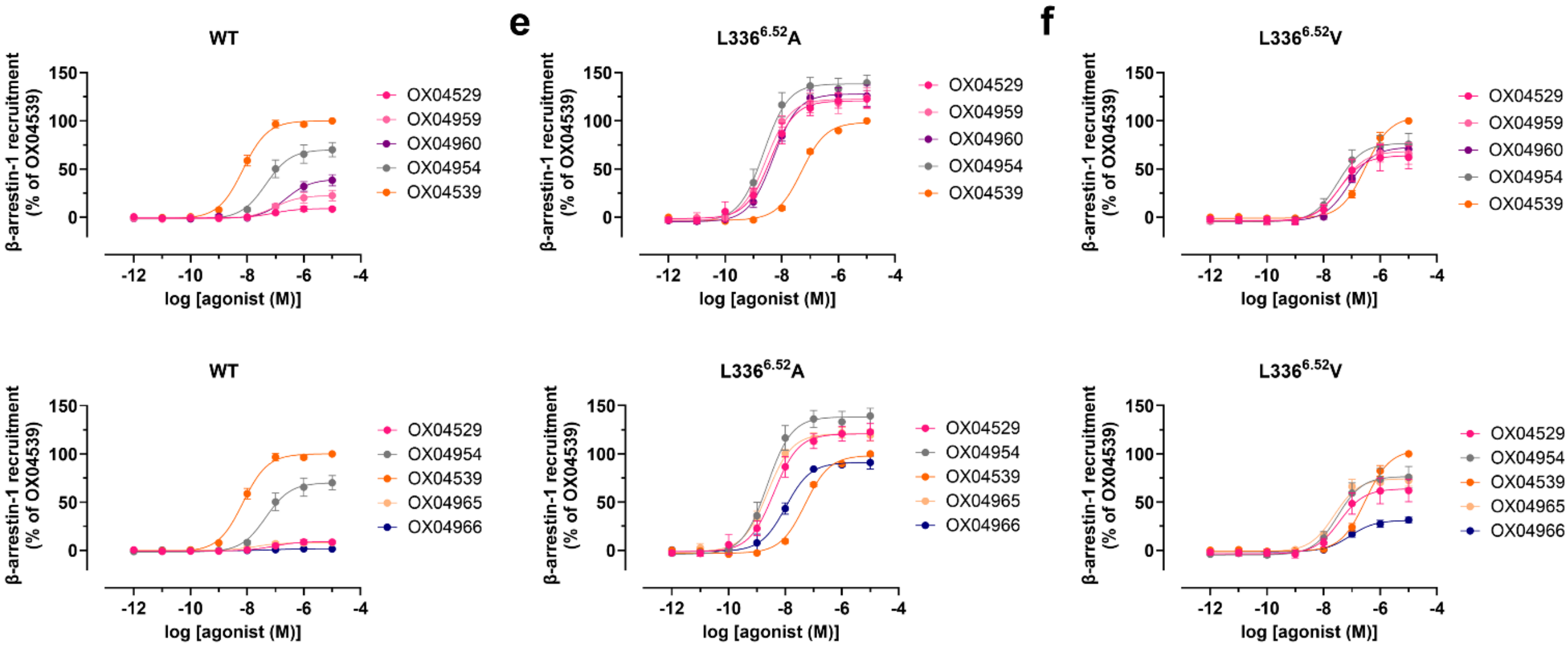

Supplementary Fig. 11: Correlation analyses, docking-based interatomic distance measurements, bias quantification and β-arrestin-1 recruitment profiles for OX04529 analogs varying the aryl 3-substituent. a, Details of measurements of interatomic distance between the most distal heavy atoms of the

ligand aryl 3-substituent and the side chain of Leu336$^{6.52}$ taken from docking models using the cryo-EM structure of the OX04529-GPR84 complex in MOE. For details of docking studies see Methods. Comparison of interatomic distance, cAMP inhibition and β-arrestin-2 recruitment potency and efficacy for each ligand. Data are means ± S.E.M. from at least three independent biological experiments, each performed in technical triplicate. **b**, Correlation map of β-arrestin-2 maximum efficacy, van der Waals volume (VDW-V), distance from the most distal heavy (non-H) atom of the ligand arene 3-substituent to Leu336$^{6.52}$, cLogP and cAMP activity. VDW-volume was calculated either from the ligand elements alone (elements) or the 3D structure of the docked pose (docking). Asterisks denote statistical significance (** $p < 0.01$, **** $p < 0.0001$) of the correlation. For details of the methodology used for correlation analysis see Methods. **c,** van der Waals volume (VDW-V) and $\Delta\Delta$Log(Emax/EC_{50}) showed strong, positive and significant correlation. BRET-based β-arrestin-1 recruitment to **d**, GPR84 wild type (WT), **e**, Leu336$^{6.52}$Ala and **f**, Leu336$^{6.52}$Val mutants in response to OX04529 analogs.

Source data are provided within the Source Data file.

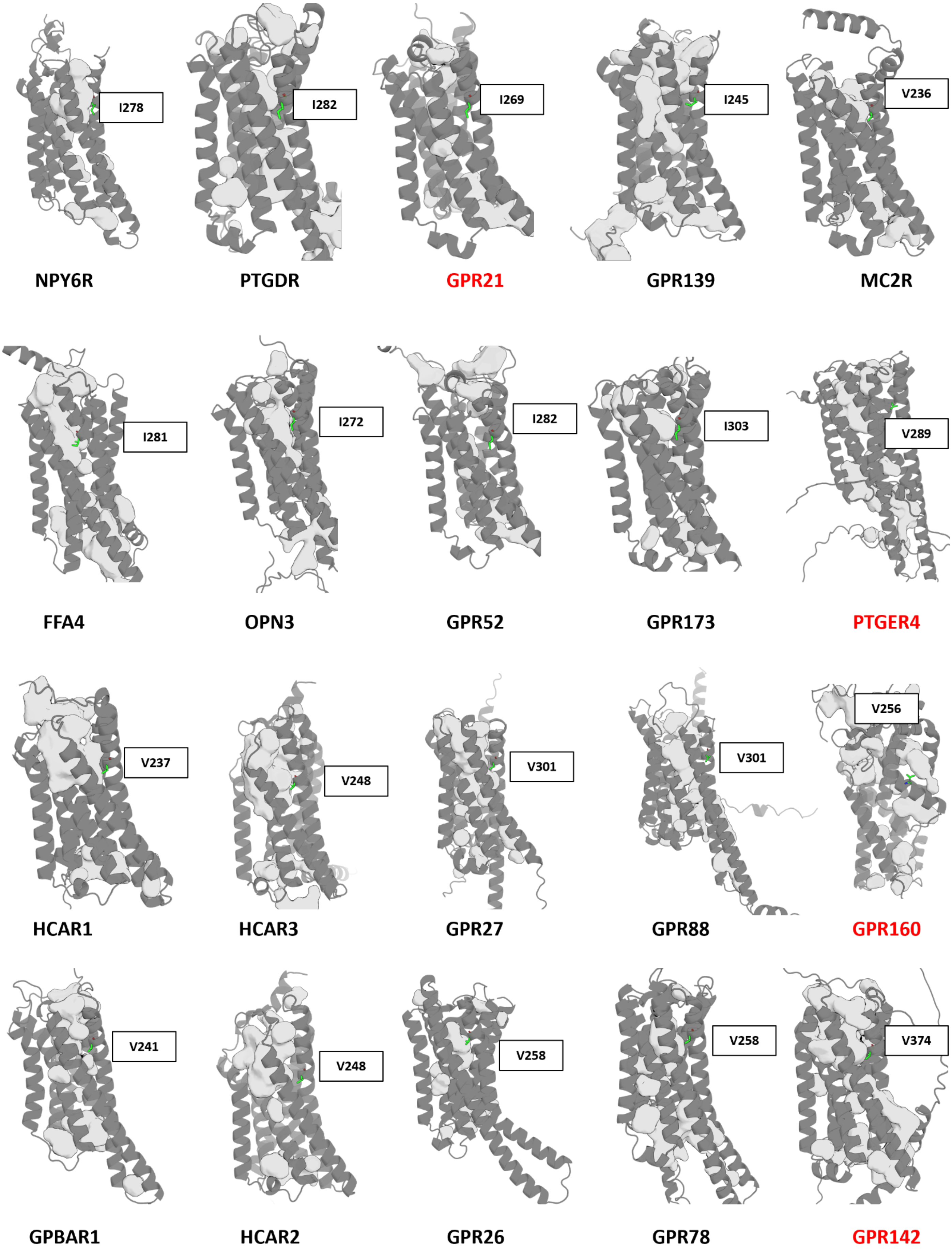
I278
I282
I269
I245
V236
NPY6R
PTGDR
GPR21
GPR139
MC2R
I281
I272
I282
I303
V289
FFA4
OPN3
GPR52
GPR173
PTGER4
V237
V248
V301
V301
V256
HCAR1
HCAR3
GPR27
GPR88
GPR160
V241
V248
V258
V258
V374
GPBAR1
HCAR2
GPR26
GPR78
GPR142

cont.

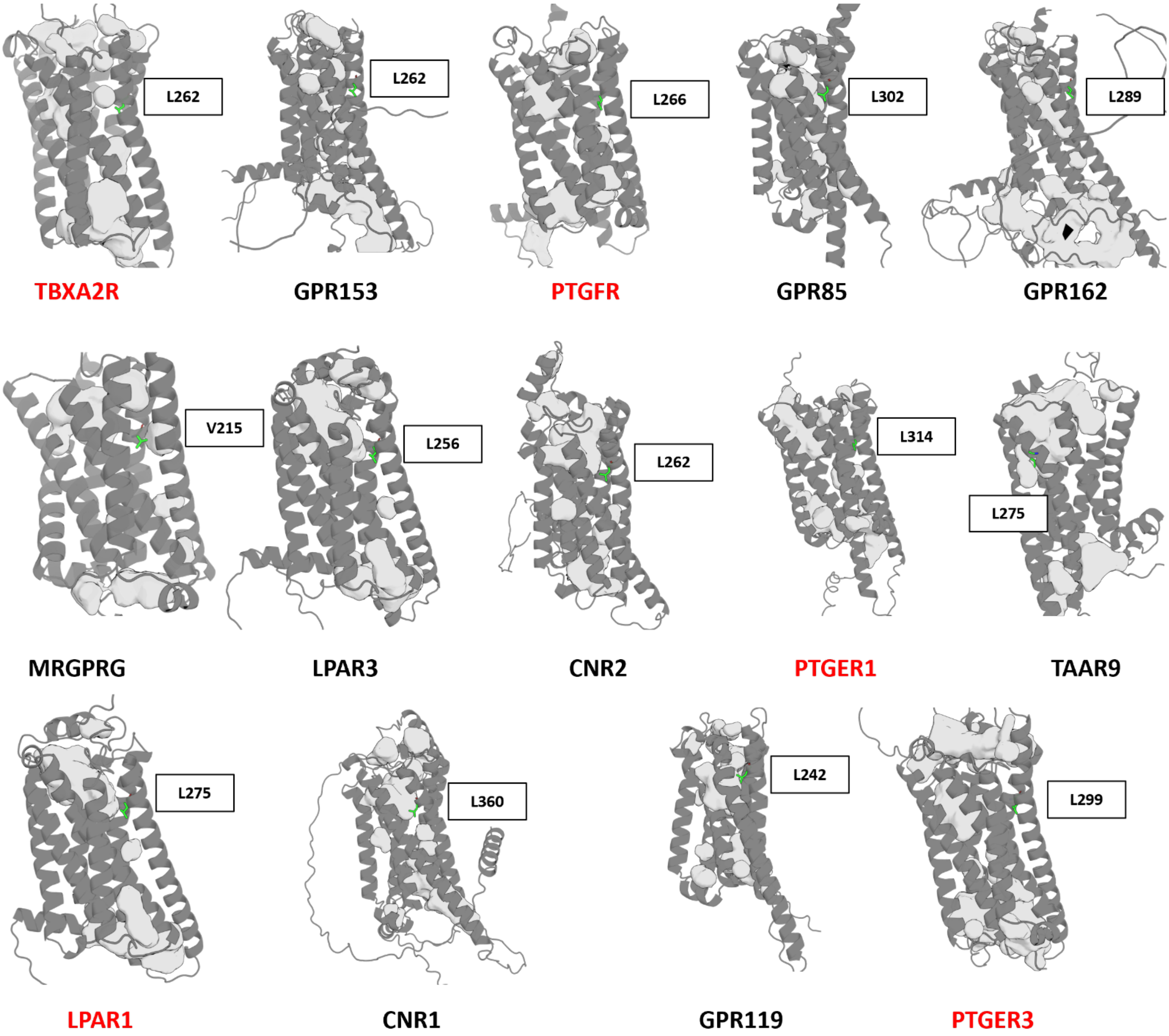

Supplementary Fig. 12: AlphaFold2 models of class A GPCRs bearing identical or similar residues at position 6.52. In 73.5% of these receptors, the 6.52 residue (green stick) is predicted to project into the orthosteric binding pocket. Grey surfaces denote predicted binding pockets.

Supplementary Fig. 13: Uncropped blots for the 4 independent biological replicates supporting **Supplementary Figure 8b** showing the lack of basal phosphorylation of residues Thr263^{ICL3} and Thr264^{ICL3} in each of wild type, Phe187$^{5.47}$Ala and Leu336$^{6.52}$Ala GPR84.

n=1

pT263-pT264

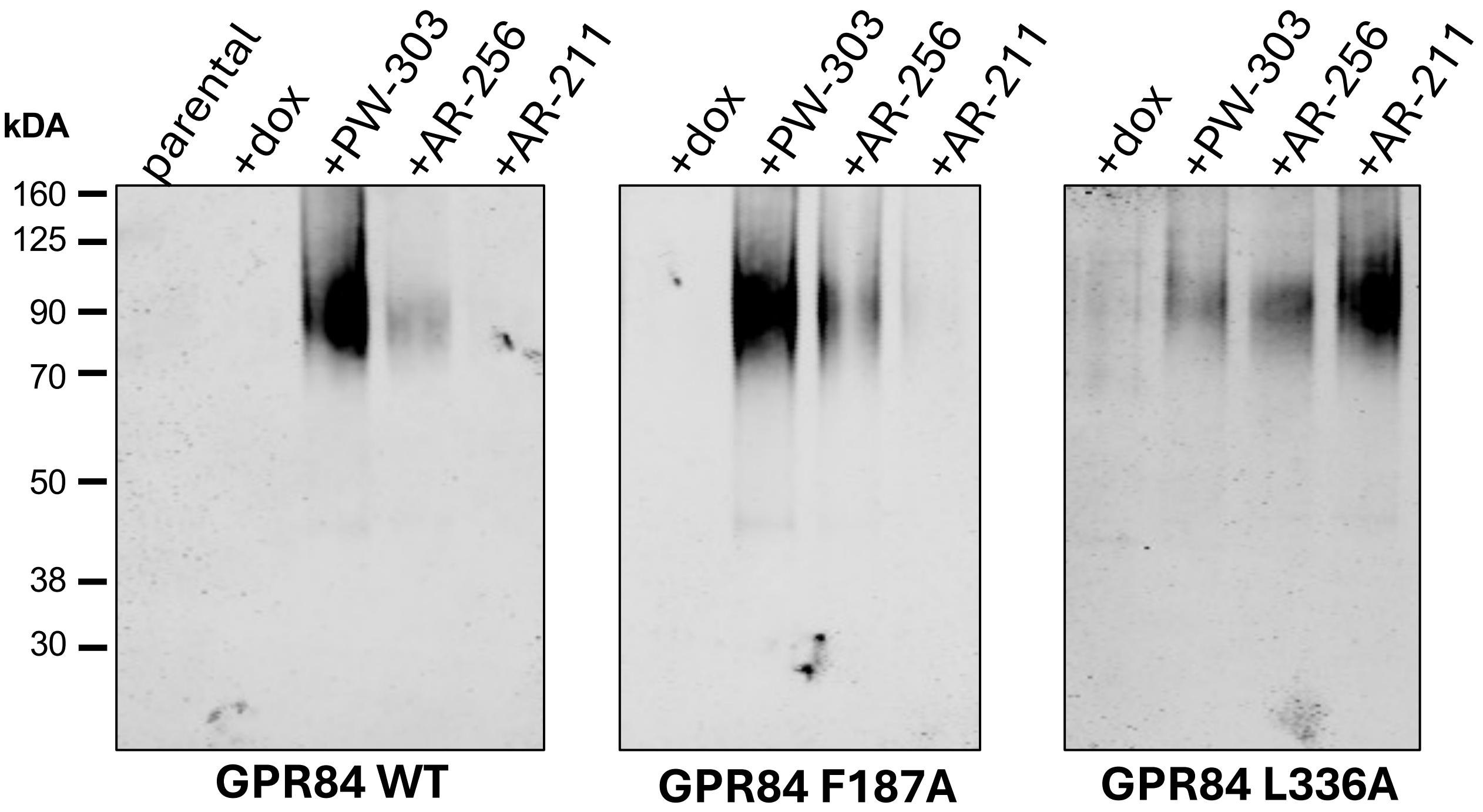

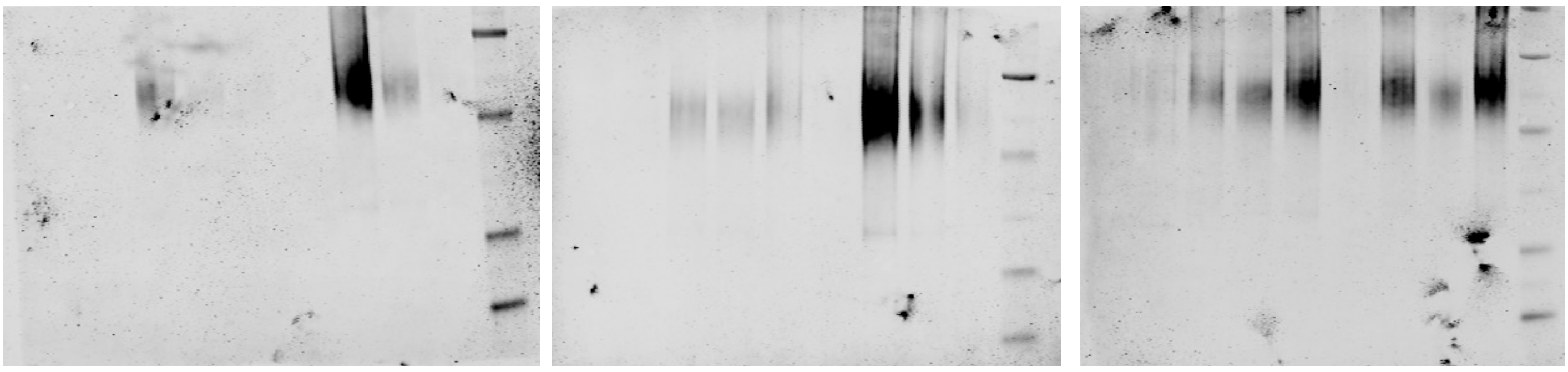

structural GPR84

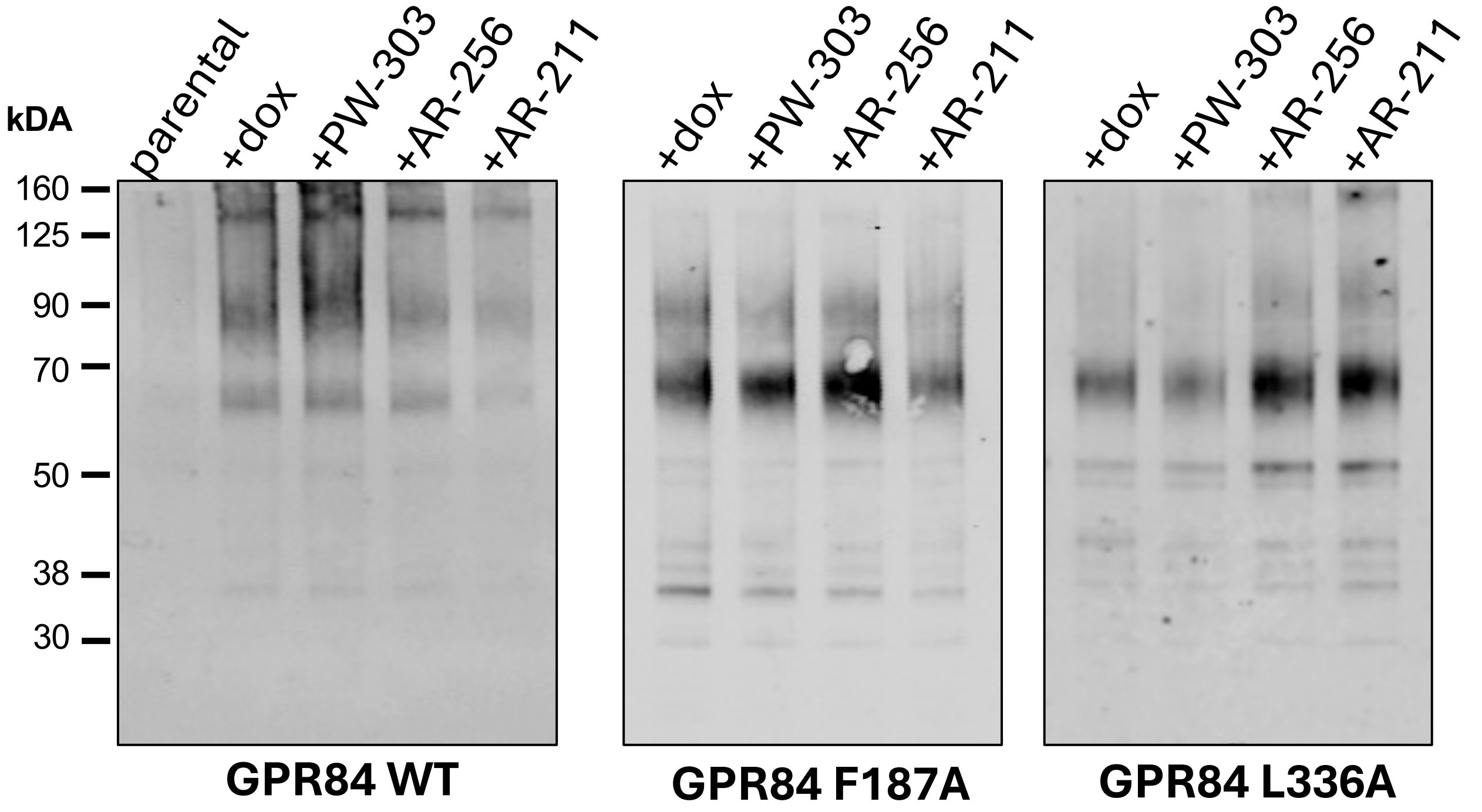

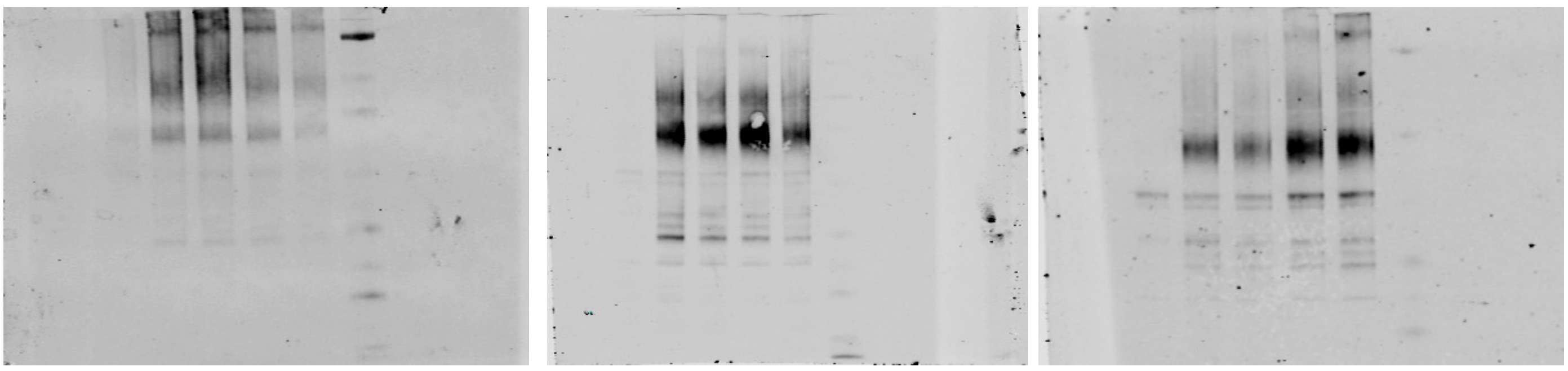

n=2

pT263-pT264

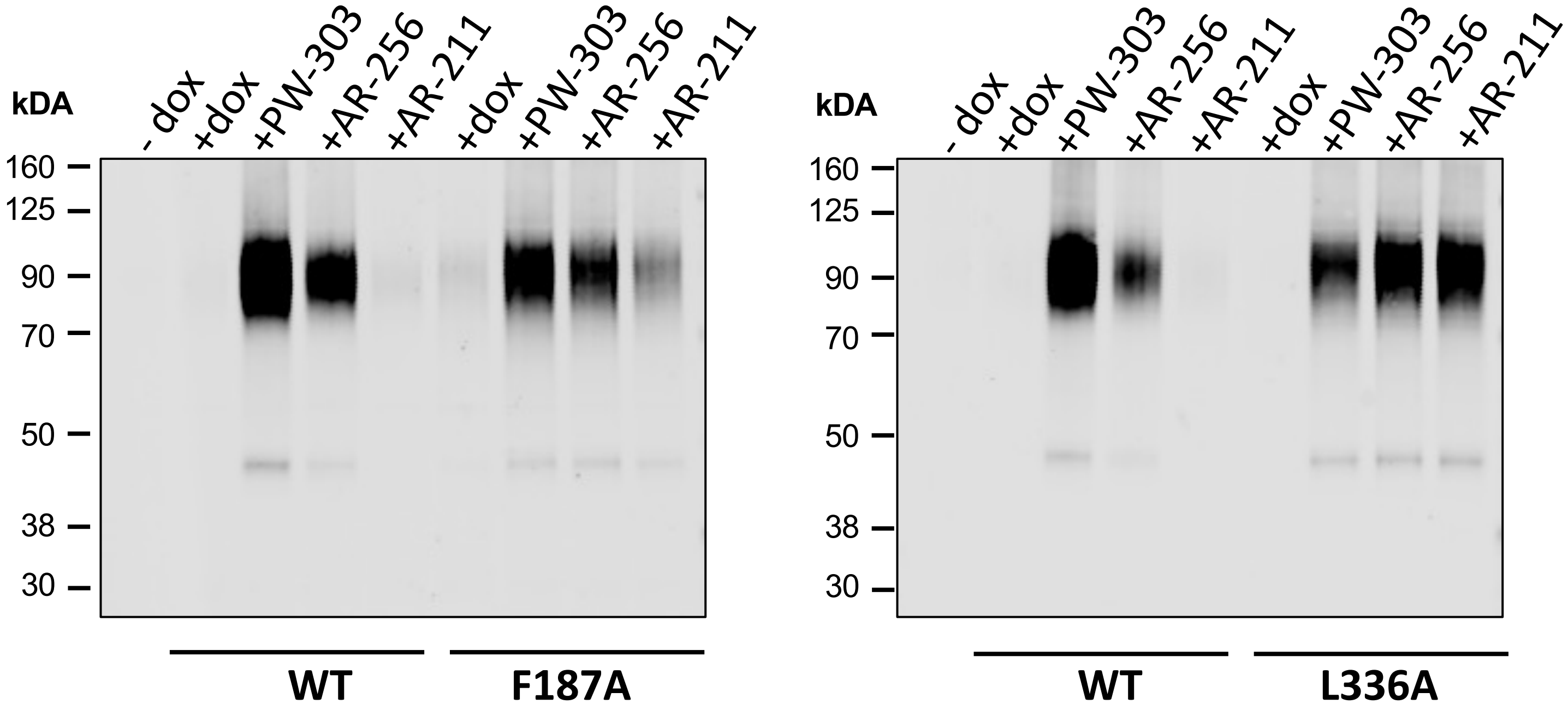

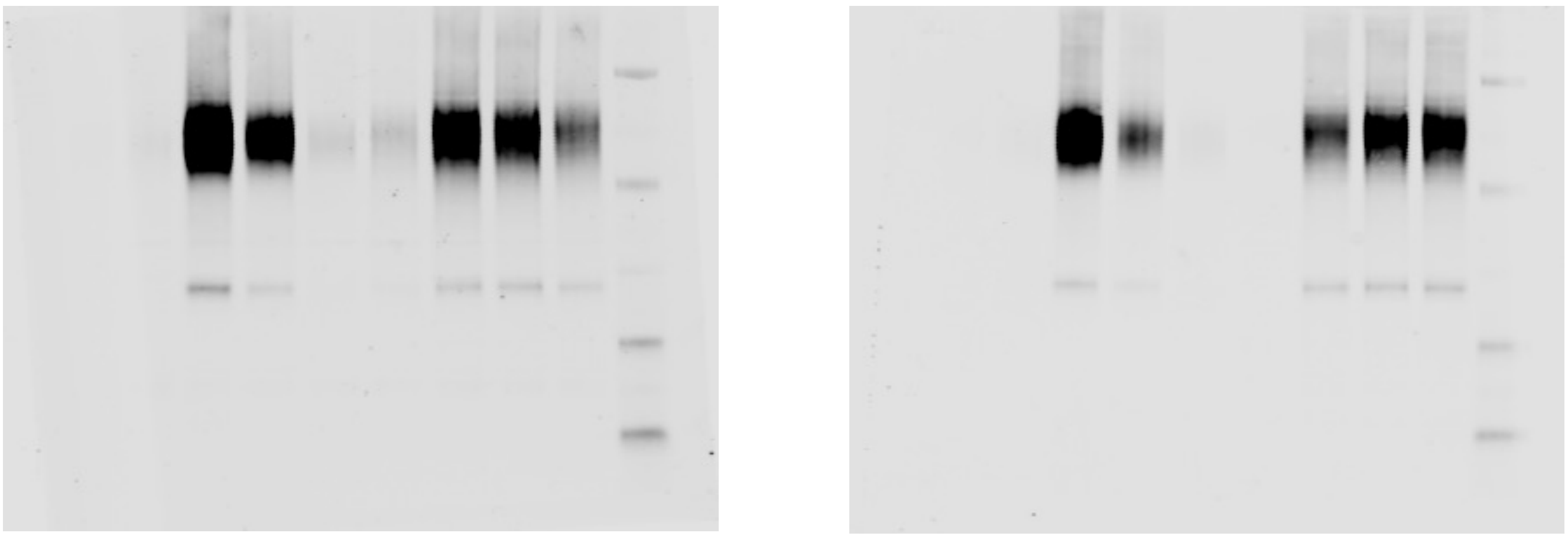

structural GPR84

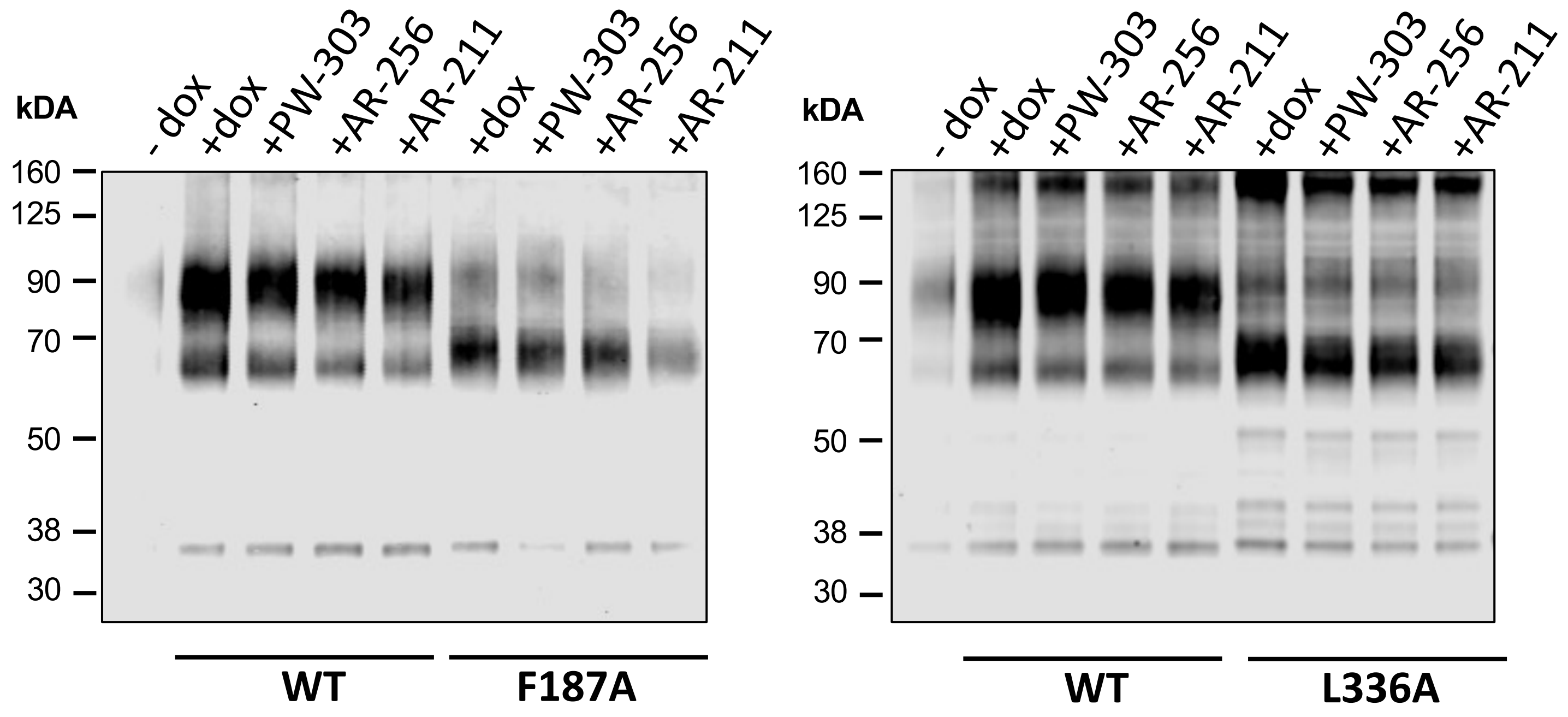

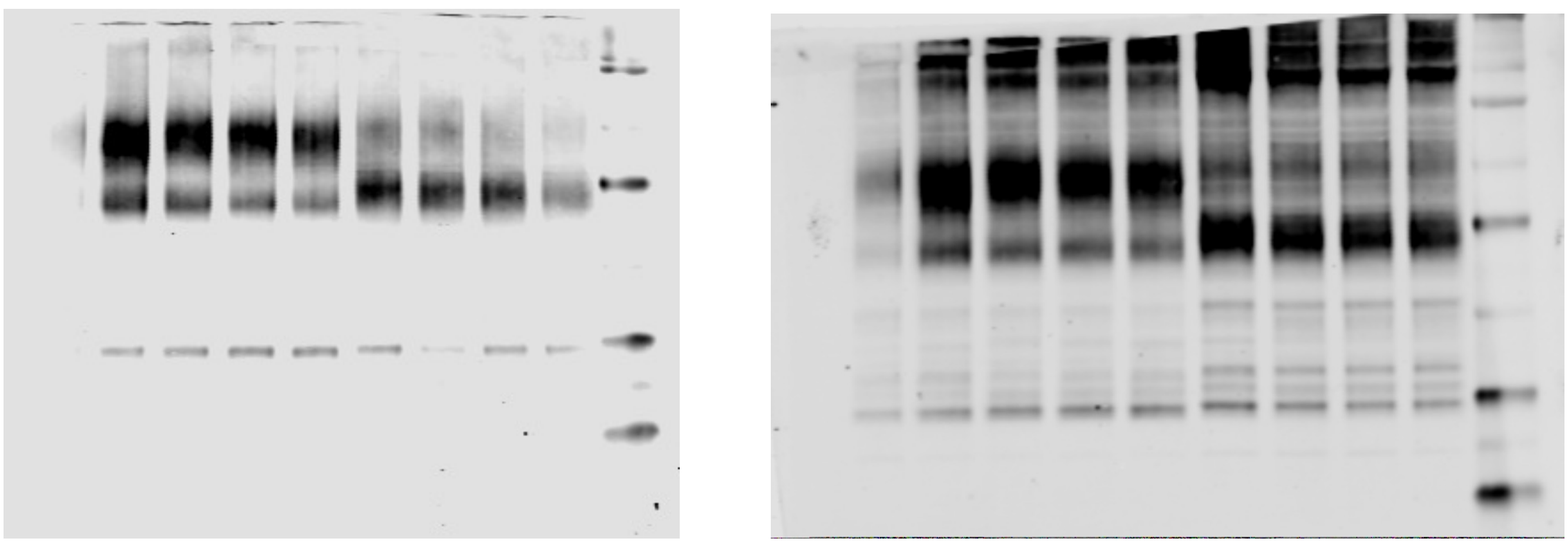

n=3

pT263-pT264

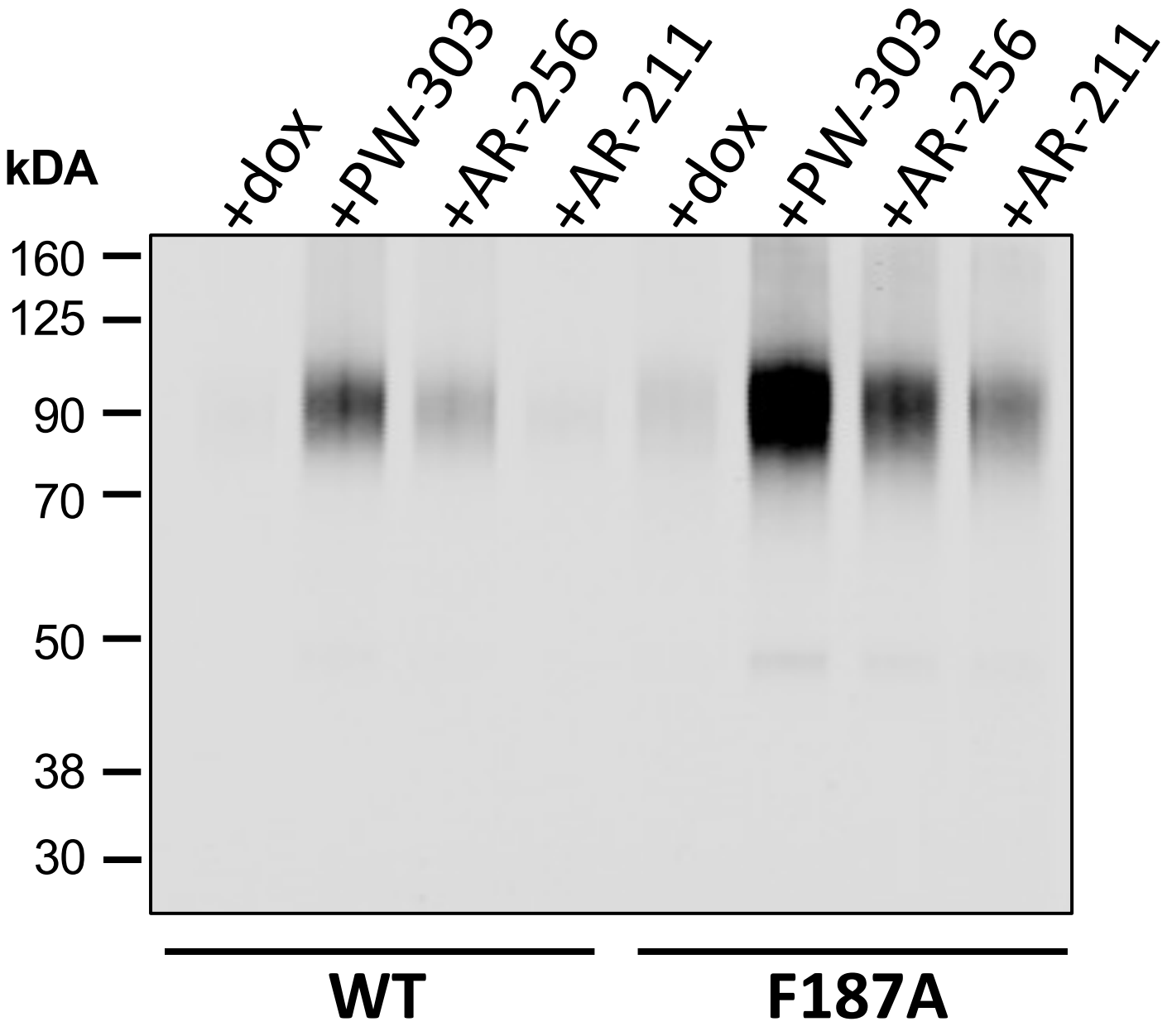

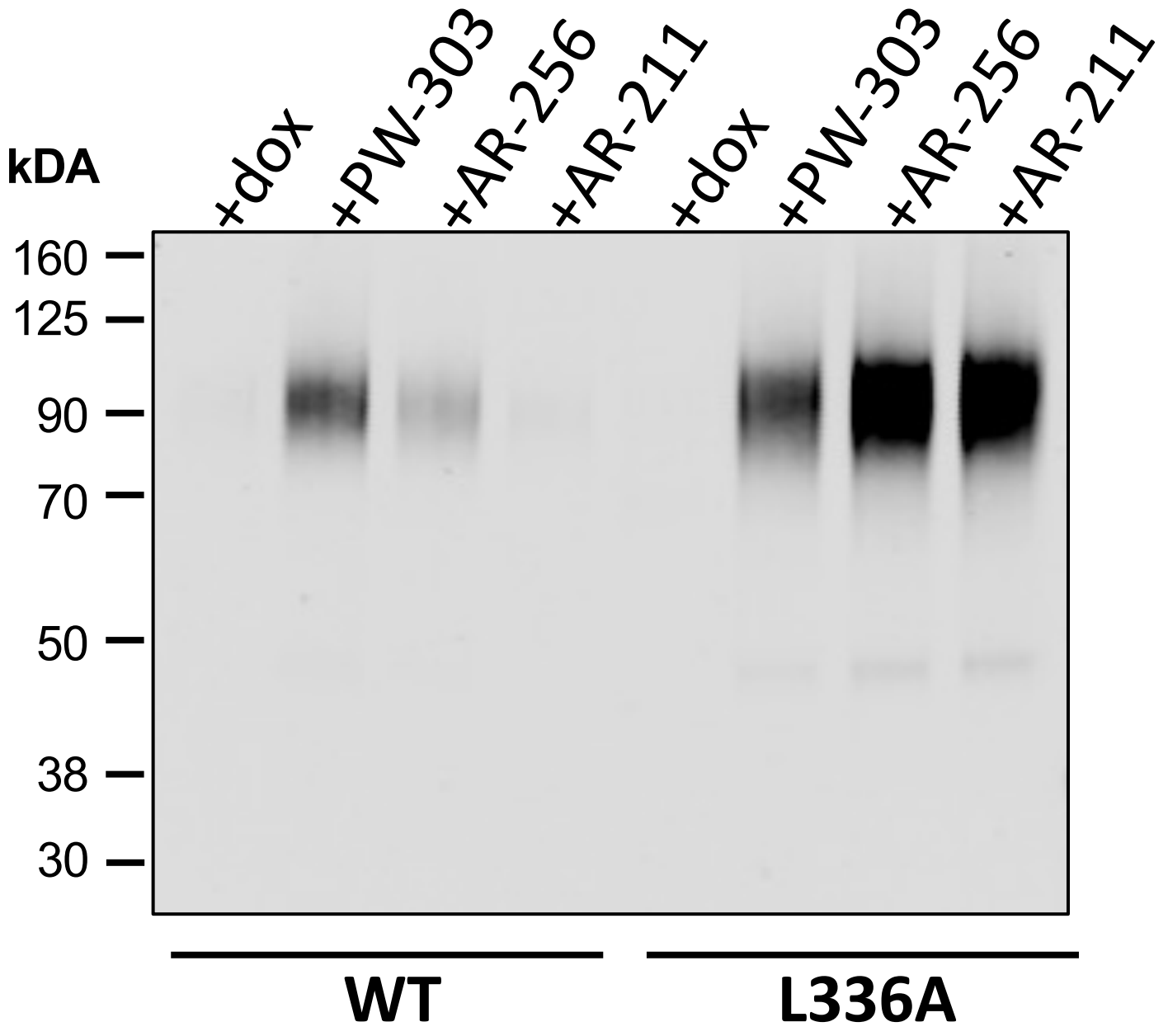

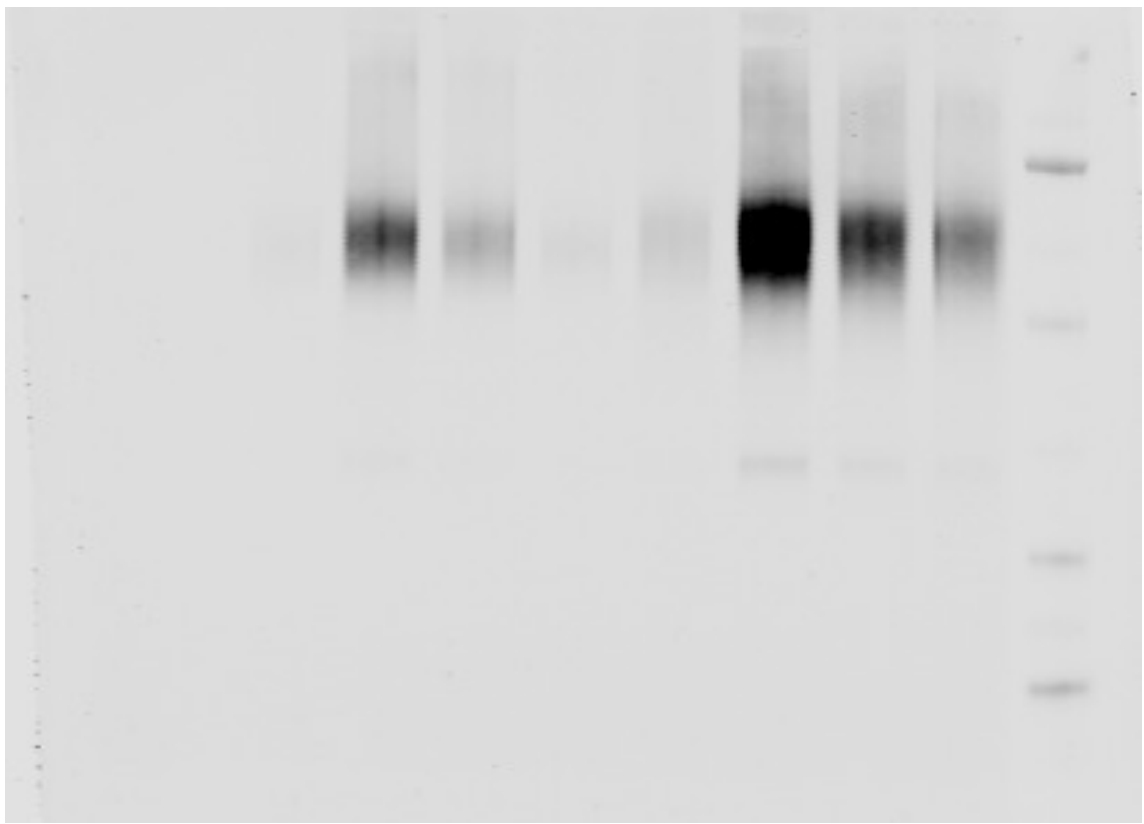

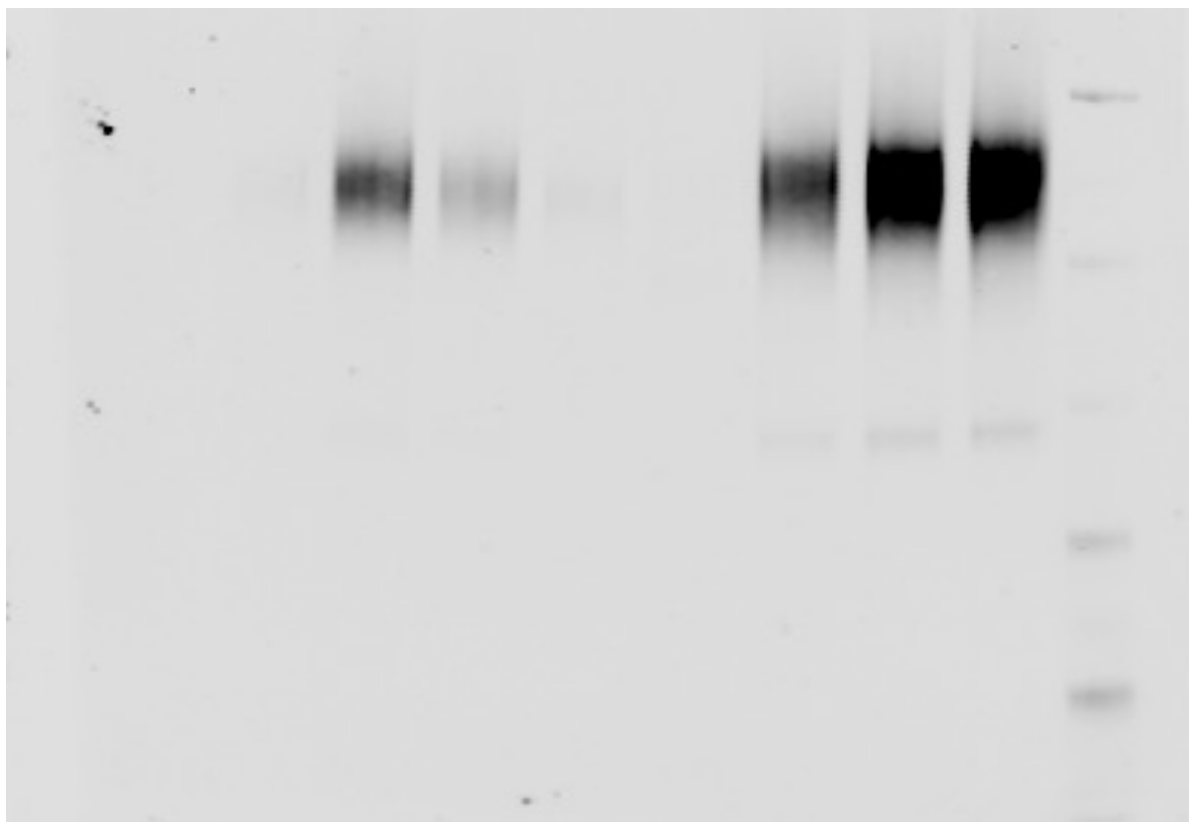

structural GPR84

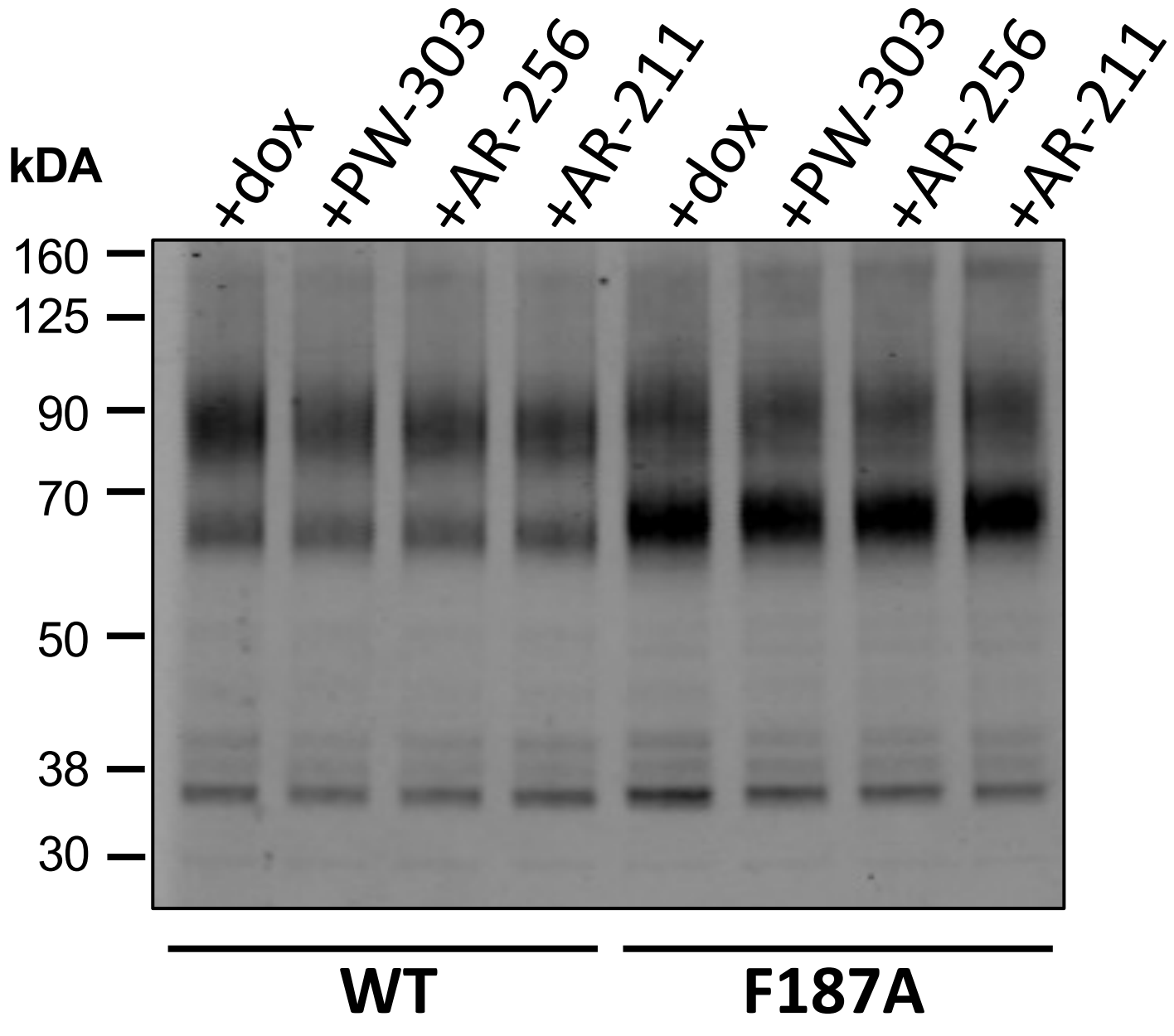

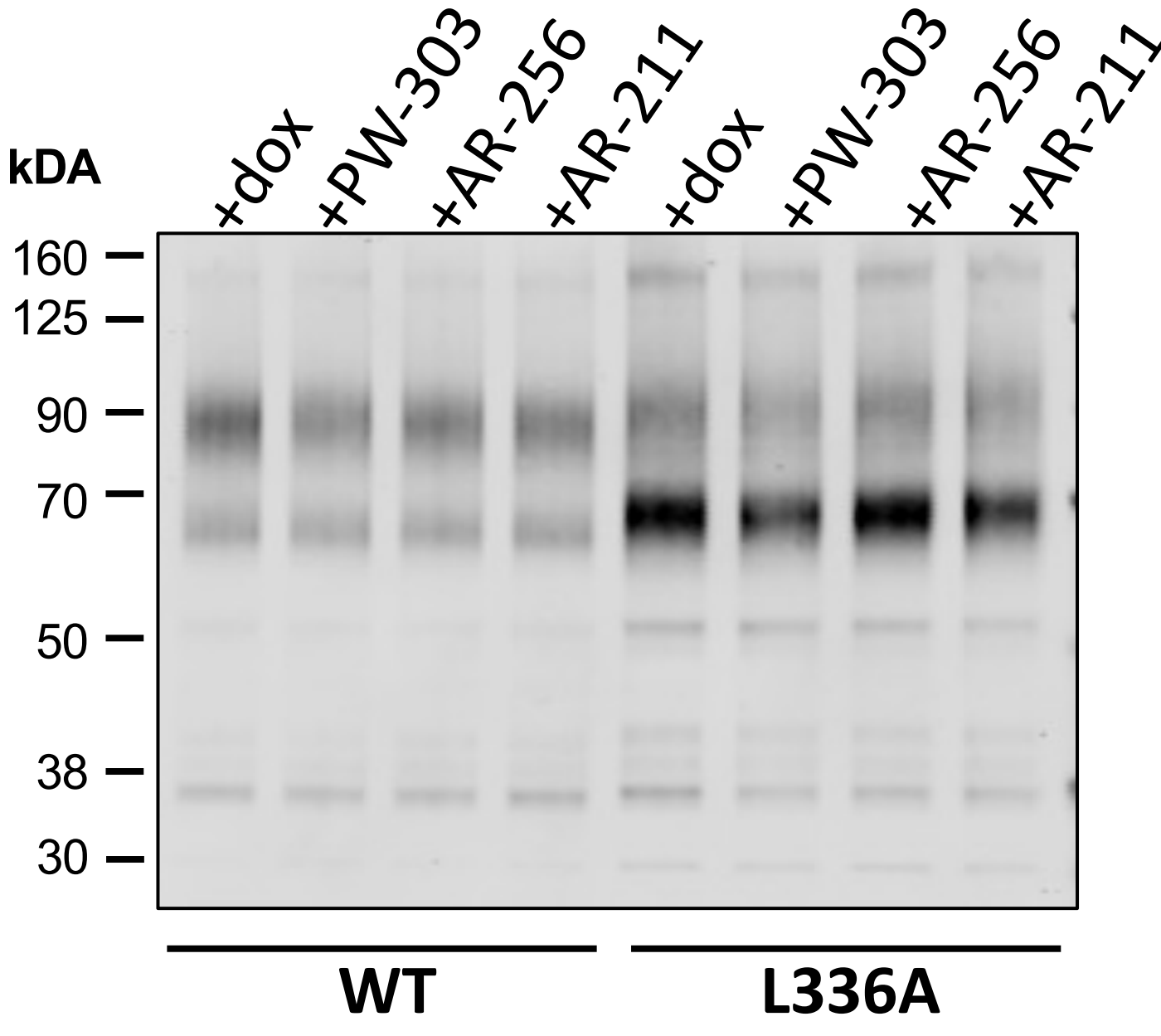

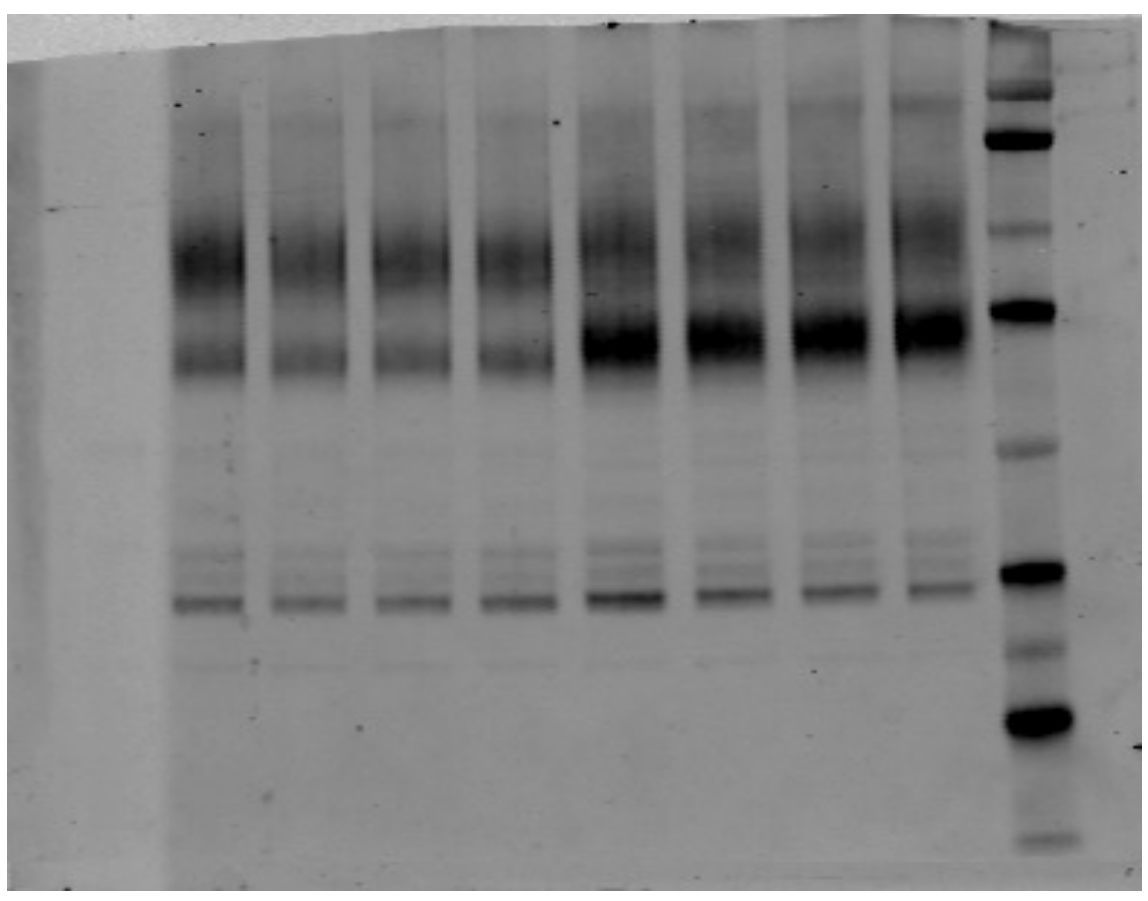

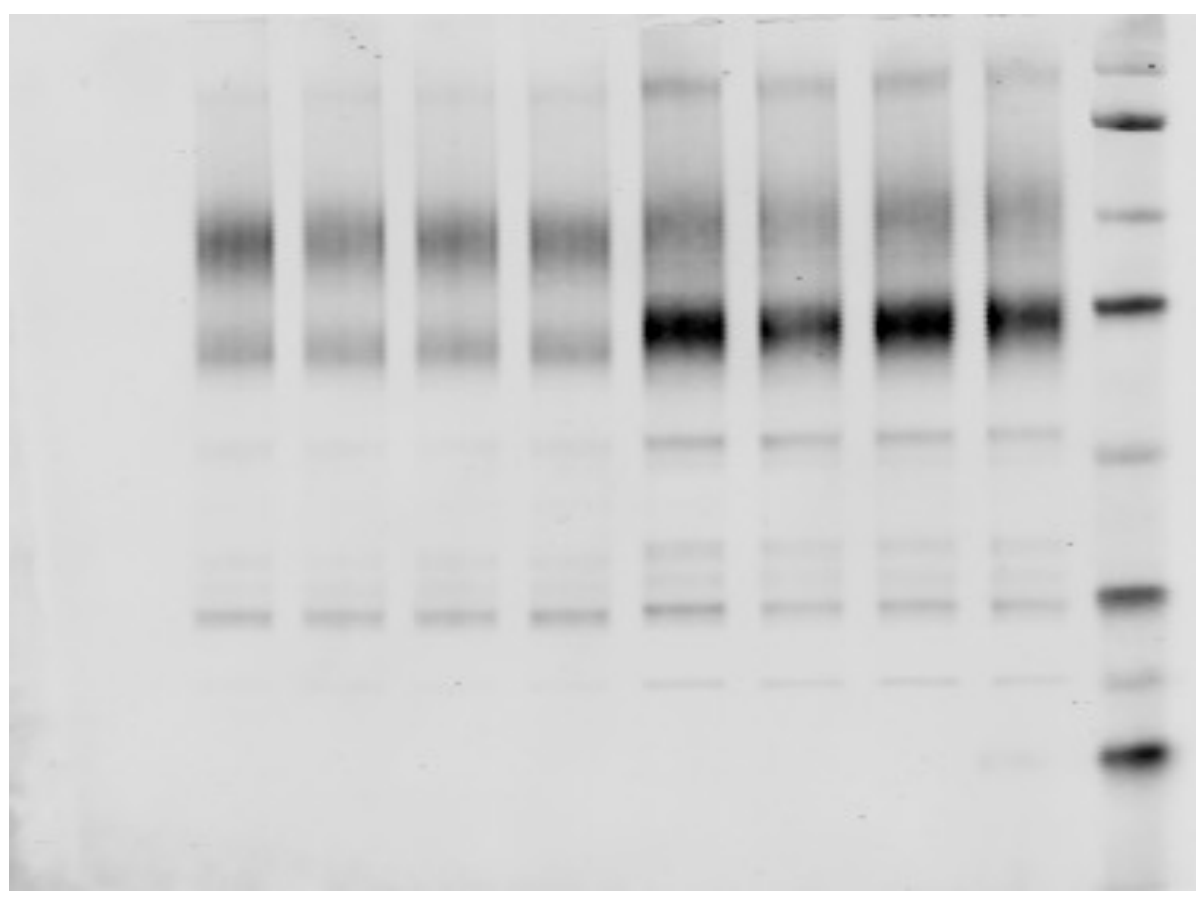

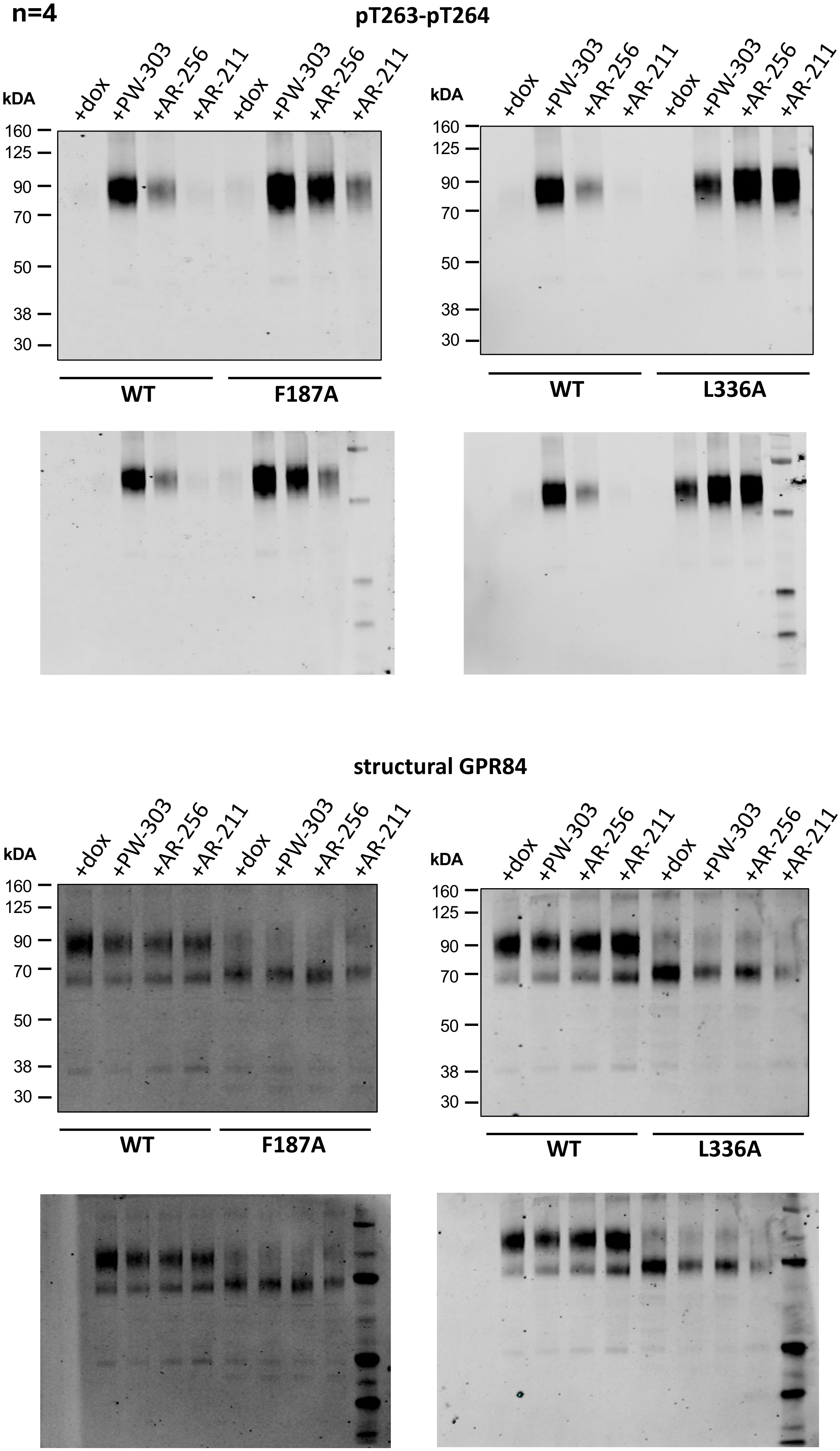
n=4
pT263-pT264
kDA
+dox
+PW-303
+AR-256
+AR-211
160
125
90
70
50
38
30
WT
F187A
L336A
structural GPR84

Supplementary Methods

General information

All reactions involving moisture sensitive reagents were carried out under a nitrogen atmosphere. Anhydrous solvents were dried by passing over an activated alumina column, under an inert atmosphere, using a solvent purification system. All other solvents and reagents were used as supplied (analytical or HPLC grade) without prior purification. Flash column chromatography was performed on Kieselgel 60 silica gel (230-400 mesh particle size) on a glass column or on a Biotage SP4 automated flash column chromatography platform. NMR spectra were recorded on Bruker Advance spectrometers at 400, 500 or 600 MHz in the deuterated solvent stated at room temperature. The field was locked by external referencing to the relevant deuteron resonance. Chemical shifts (δ) are reported in parts per million (ppm) and coupling constants (*J*) are quoted in Hz. Data are reported as follows: chemical shift, multiplicity (s = singlet, d = doublet, t = triplet, q = quartet, sext = sextuplet, hept = heptet, and m = multiplet), coupling constant and integration. Low-resolution mass spectra (*m/z*) were recorded on an Agilent 1260 Infinity II with Diode Array and Single Quadrupole Detectors in solutions of MeOH. A selected peak is reported in Daltons and its intensity given as percentage of the base peak. High resolution mass spectra (HRMS) were run on a Bruker microTOF (ESI and APCI) or on a Waters GCT (EI). Experiments conducted in CROs used their standard equipment. The purity for test compounds was determined by HPLC on a Shimadzu SIL-20AC HT instrument. HPLC conditions were as follows: Atlantis dC18, 100 Å, 5 μm, Column 4.6 × 150 mm, 35−100% MECN in water, 15 min run, flow rate 1.5 mL/min, UV detection (λ = 220, 254, 280 nm). The mass spectra were obtained using Agilent 6120 bench-top single quadrupole instrument using electrospray ionization (ESI).

Reaction Schemes

Scheme 1. Synthesis of **OX04539**[a]

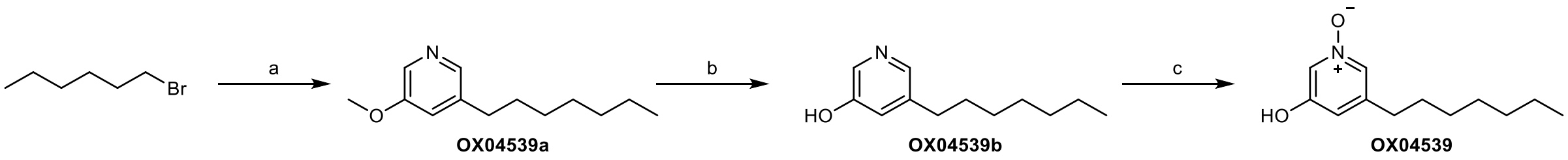

[a]Reagents and conditions: (a) (1) PPh_3, Toluene, reflux, 16 h, 63%; (2) 5-methoxynicotinaldehyde, NaOtBu, dichloromethane, 0ºC–rt, 91%; (3) Pd/C, H_2, MeOH, rt, 1 h, 99%; (b) L-Selectride, THF, 70ºC, 72 h, 39%; (c) *m*-CPBA, CH_2Cl_2, rt, 2 h, 55%.

Scheme 2. Synthesis of **OX04540**[a]

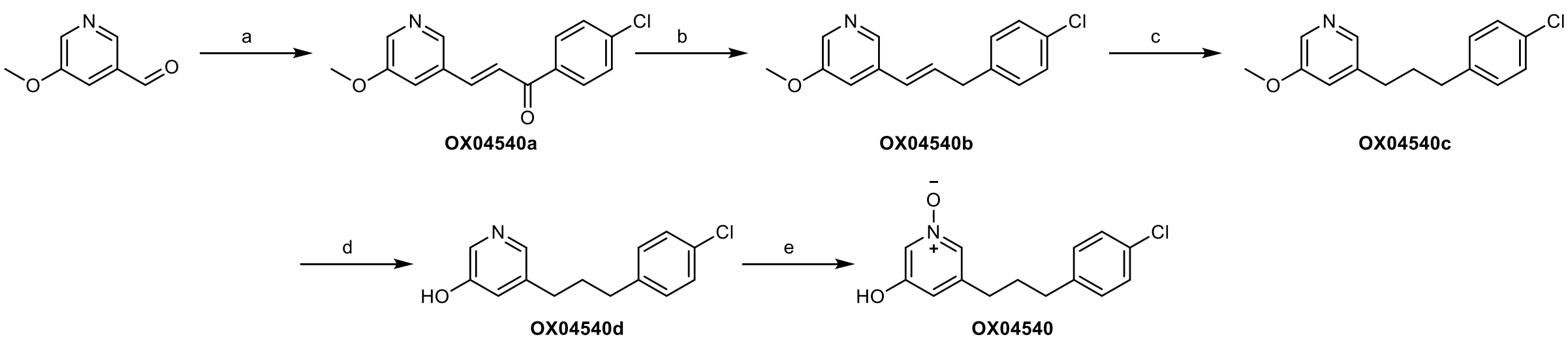

[a]Reagents and conditions: (a) 1-(4-chlorophenyl)ethan-1-one, $BF_3 \cdot Et_2O$, 1,4-dioxane, 105ºC, 16 h, 70%; (b) Et_3SiH, TFA, rt, 48 h, 59%; (c) Pd/C, H_2, MeOH, rt, 1 h, 79%; (d) 48% HBr, 120ºC, 48 h, 23%; (e) *m*-CPBA, CH_2Cl_2, rt, 2 h, 28%.

Scheme 3. Synthesis of **OX04954**[a]

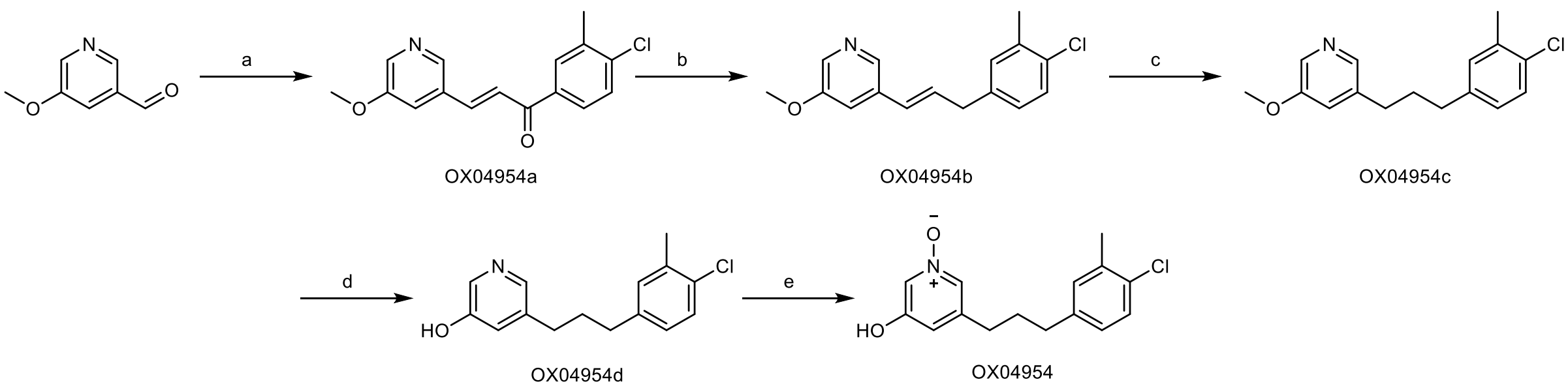

[a]Reagents and conditions: (a) 1-(4-chloro-3-methylphenyl)ethan-1-one, $BF_3 \cdot Et_2O$, 1,4-dioxane, 105ºC, 16 h, 85%; (b) Et_3SiH, TFA, rt, 48 h, 82%; (c) Pd/C, H_2, MeOH, rt, 30 min, 85%; (d) 48% HBr, 120ºC, 48 h, 79%; (e) *m*-CPBA, CH_2Cl_2, rt, 2 h, 87%.

Scheme 4. Synthesis of **OX04956**[a]

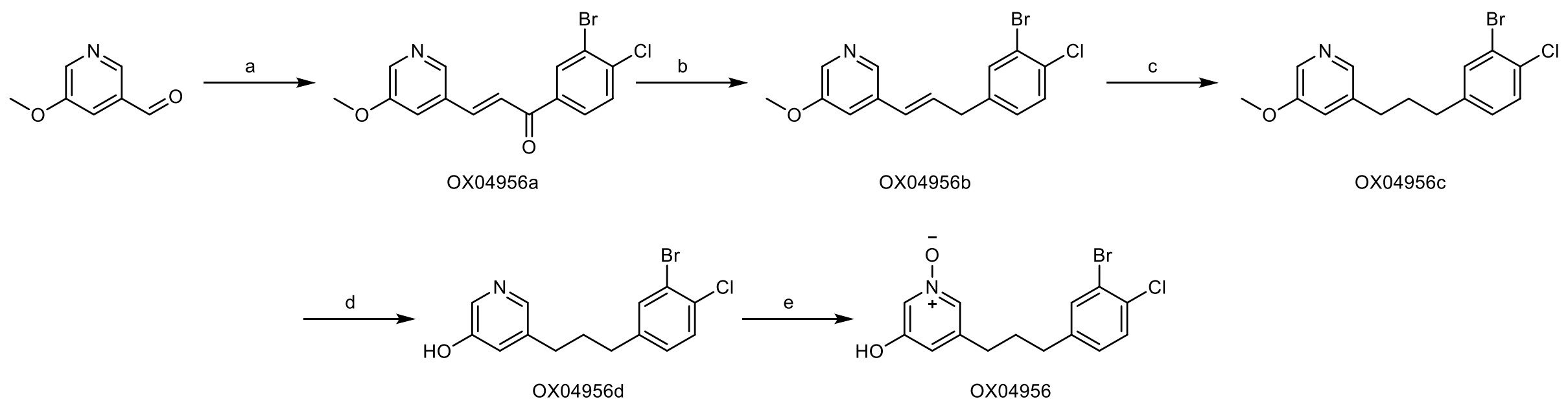

[a]Reagents and conditions: (a) 1-(3-bromo-4-chlorophenyl)ethan-1-one, $BF_3 \cdot Et_2O$, 1,4-dioxane, 105ºC, 16 h, 29%; (b) Et_3SiH, TFA, rt, 48 h, 59%; (c) PtO_2, H_2, MeOH, rt, 1 h, 85%; (d) 48% HBr, 120ºC, 48 h, 28%; (e) *m*-CPBA, CH_2Cl_2, rt, 2 h, 52%.

Scheme 5. Synthesis of **OX04955**[a]

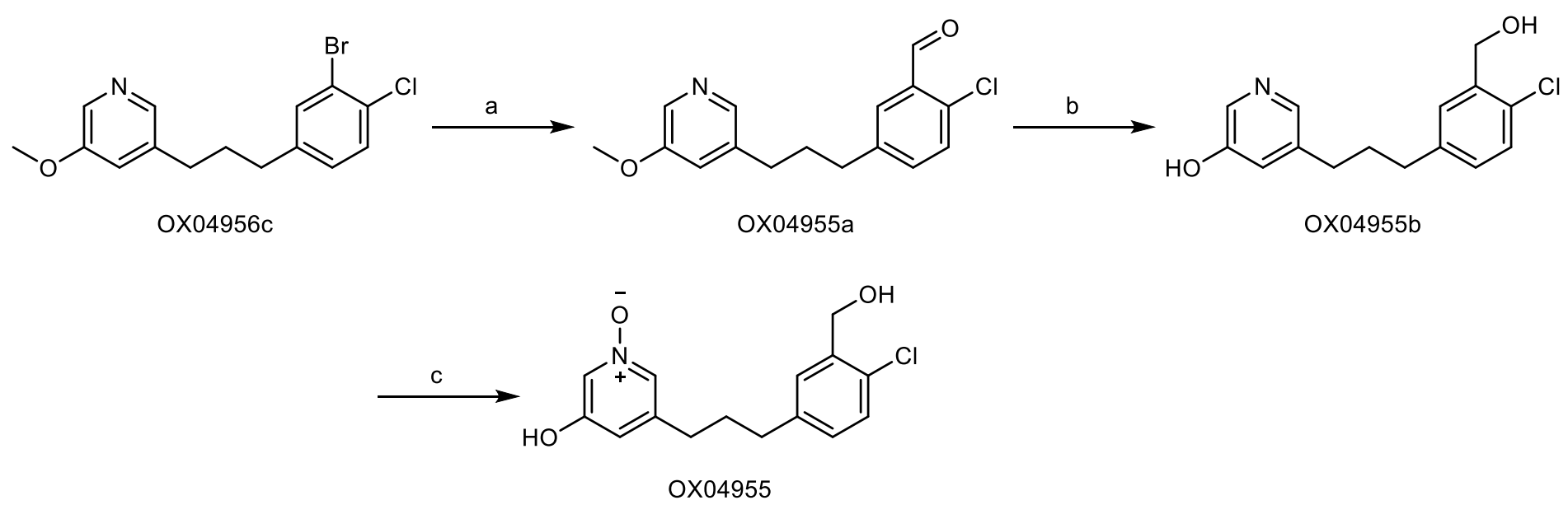

[a]Reagents and conditions: (a) (1) n-BuLi, THF, -78ºC, 5 min; (2) DMF, THF, -78ºC, 15 min, 45%; (b) L-Selectride, THF, 70ºC, 48 h, 39%; (c) *m*-CPBA, CH_2Cl_2, rt, 2 h, 64%.

Scheme 6. Synthesis of **OX04957**[a]

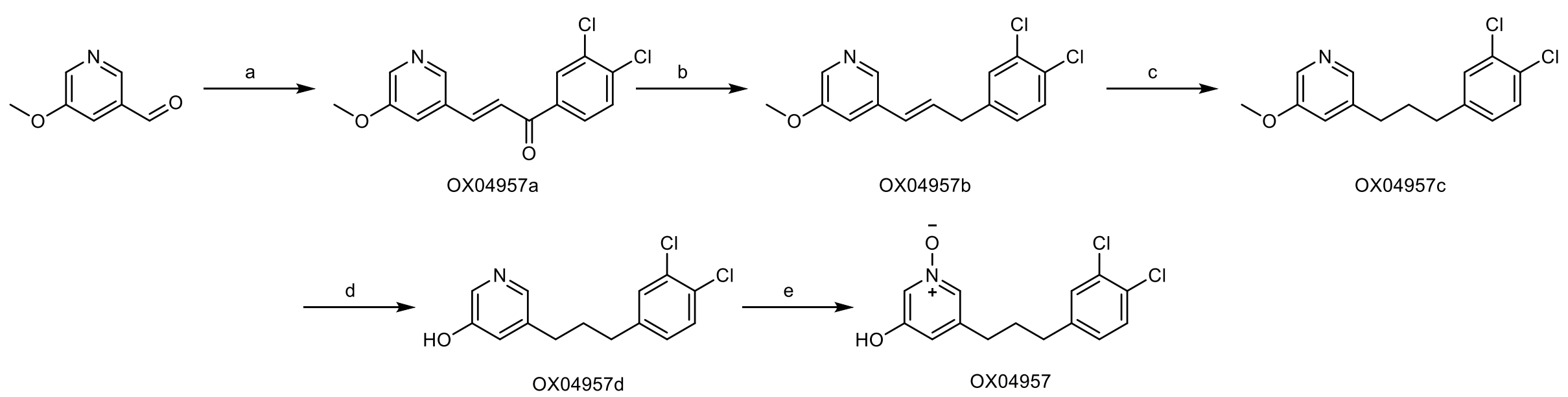

[a]Reagents and conditions: (a) 1-(3,4-dichlorophenyl)ethan-1-one, $BF_3 \cdot Et_2O$, 1,4-dioxane, 105ºC, 16 h, 67%; (b) Et_3SiH, TFA, rt, 48 h, 38%; (c) PtO_2, H_2, MeOH, rt, 1 h, 76%; (d) 48% HBr, 120ºC, 48 h, 46%; (e) *m*-CPBA, CH_2Cl_2, rt, 2.5 h, 23%.

Scheme 7. Synthesis of **OX04958**[a]

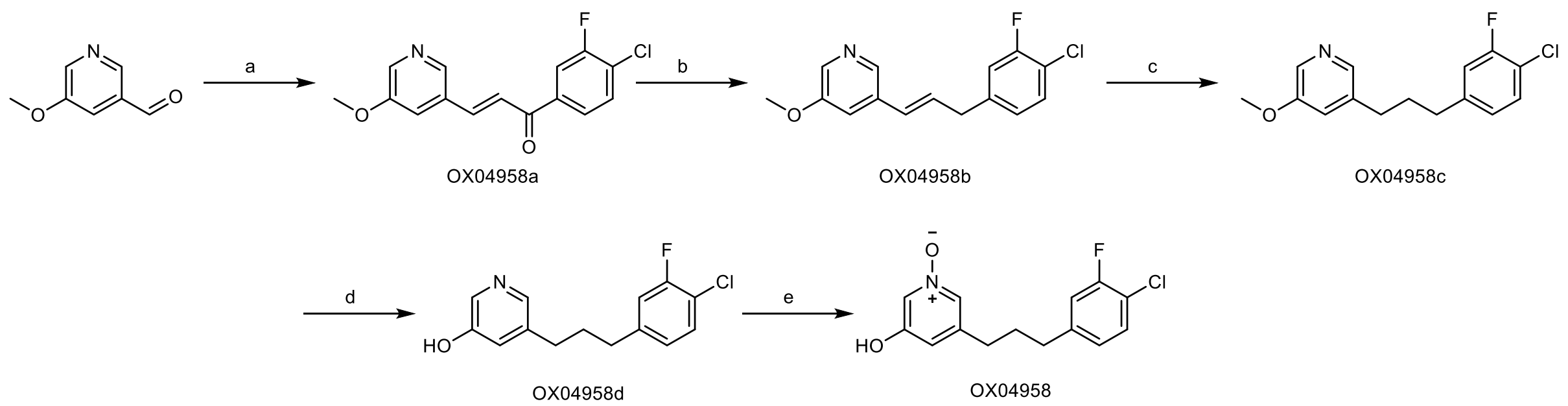

[a]Reagents and conditions: (a) 1-(4-chloro-3-fluorophenyl)ethan-1-one, $BF_3 \cdot Et_2O$, 1,4-dioxane, 105ºC, 16 h, 64%; (b) Et_3SiH, TFA, rt, 48 h, 38%; (c) PtO2, H_2, MeOH, rt, 1 h, 93%; (d) 48% HBr, 120ºC, 48 h, 18%; (e) *m*-CPBA, CH_2Cl_2, rt, 2.5 h, 65%.

Scheme 8. Synthesis of **OX04959**[a]

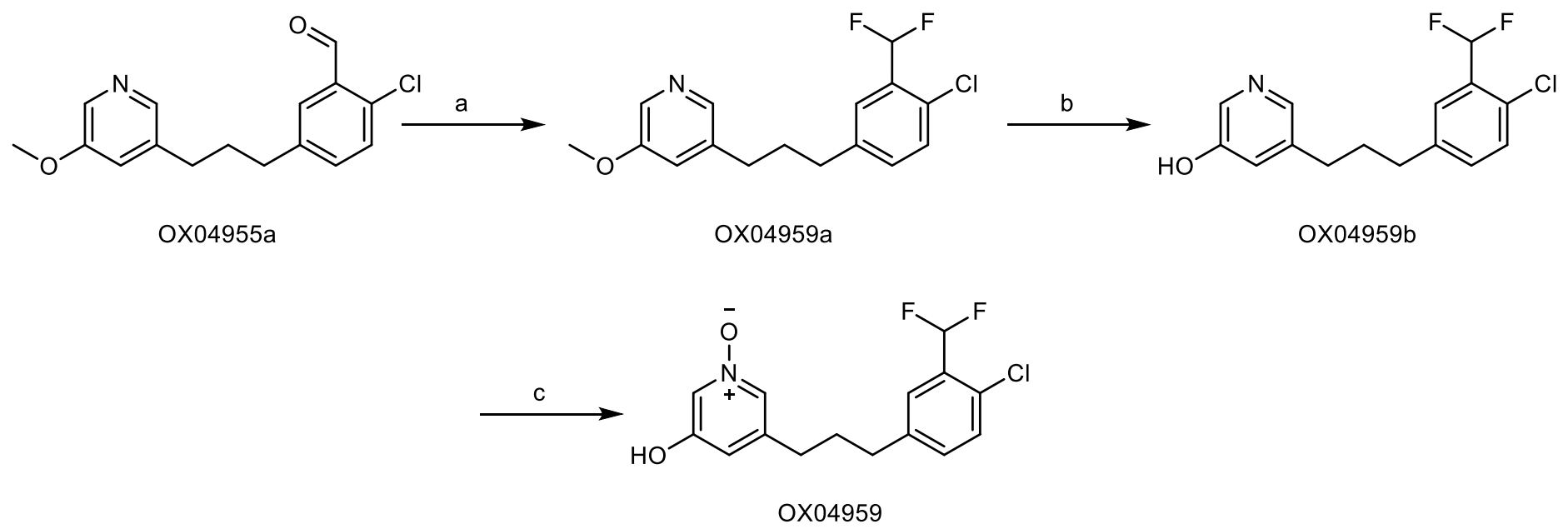

[a]Reagents and conditions: (a) DAST, CH_2Cl_2, rt, 16 h, 37%; (b) L-Selectride, THF, 70ºC, 48 h, 92%; (c) *m*-CPBA, CH_2Cl_2, rt, 2 h, 43%.

Scheme 9. Synthesis of **OX04960**[a]

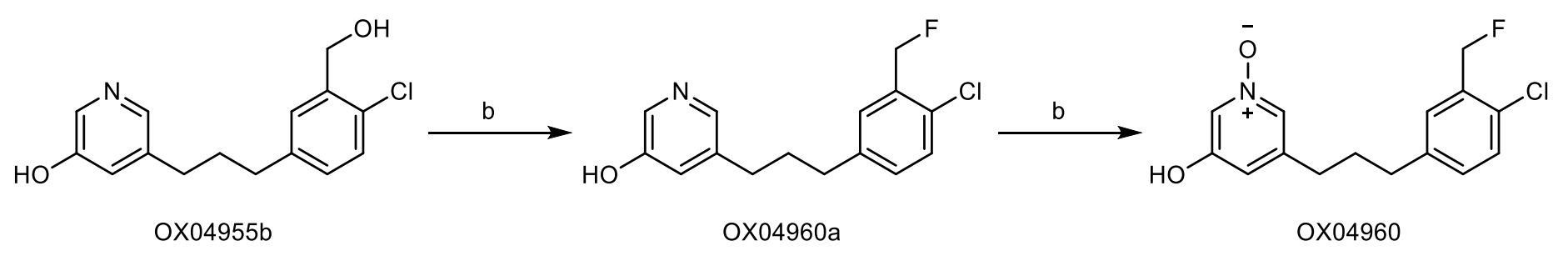

[a]Reagents and conditions: (a) DAST, CH_2Cl_2, rt, 24 h, 11%; (b) *m*-CPBA, CH_2Cl_2, rt, 2 h, 44%.

Scheme 10. Synthesis of **OX04961**[a]

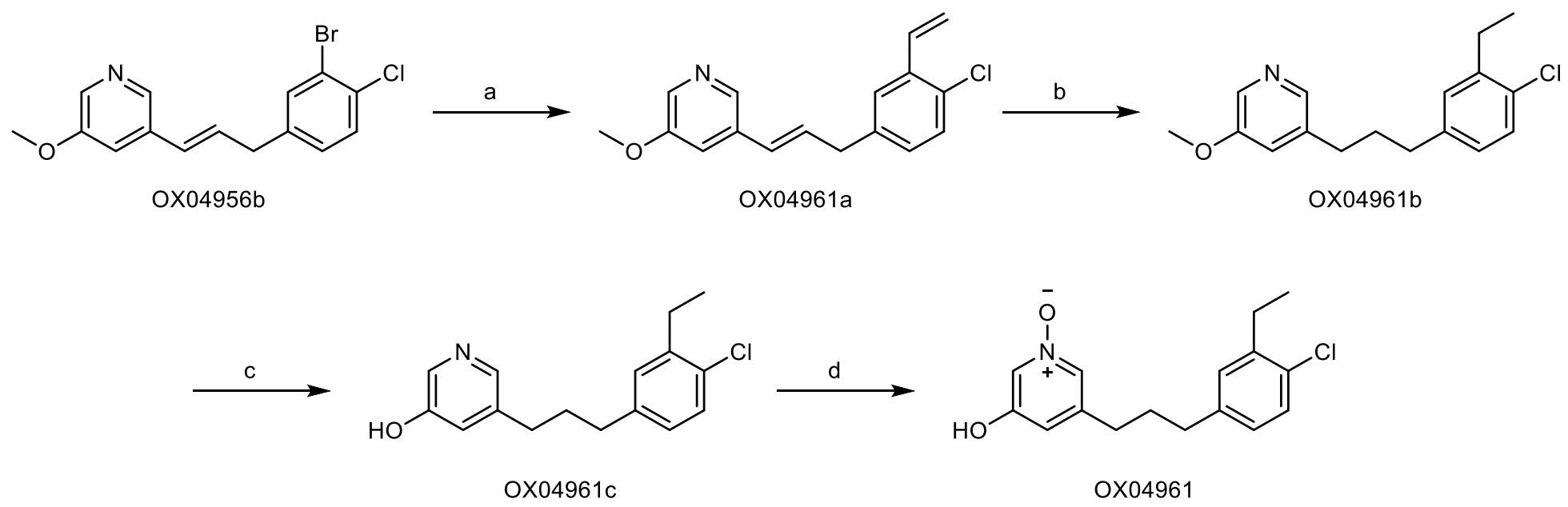

[a]Reagents and conditions: (a) vinyl boronic ester, Cs_2CO_3, PPh_3, $Pd(OAc)_2$, THF:H_2O 9:1, 70ºC, 25 h, 56%; (b) Pd/C, H_2, MeOH, rt, 1 h, 39%; (c) 48% HBr, 120ºC, 48 h, 55%; (d) *m*-CPBA, CH_2Cl_2, rt, 2 h, 41%.

Scheme 11. Synthesis of **OX04962**[a]

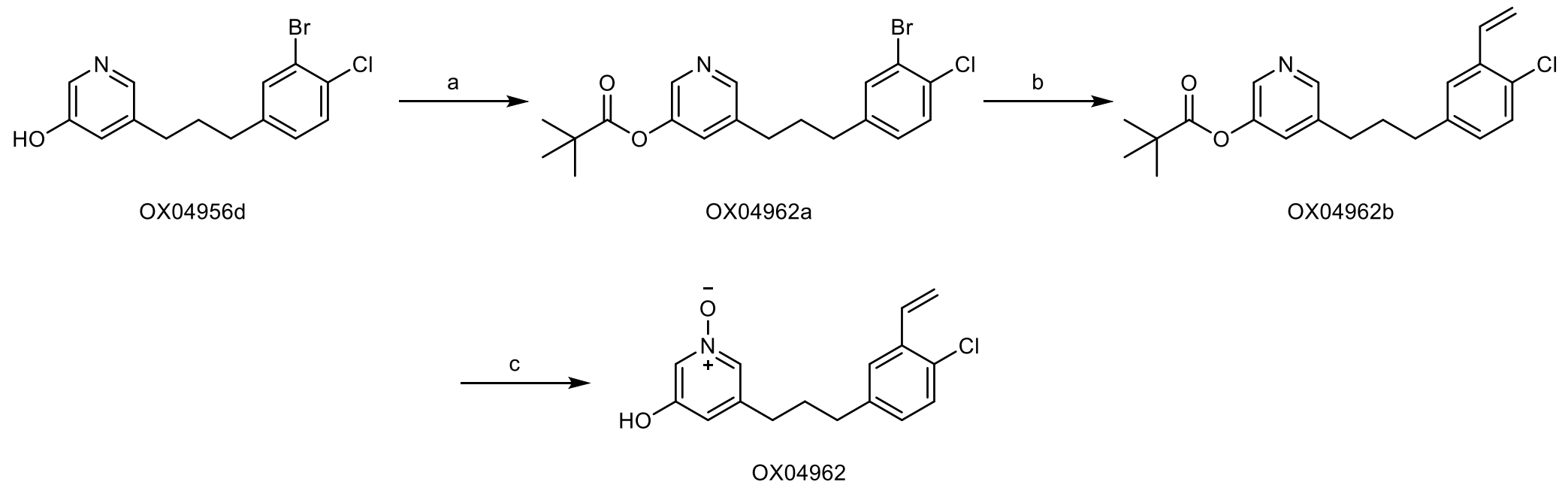

[a]Reagents and conditions: (a) trimethylacetyl chloride, NEt_3, CH_2Cl_2, rt, 1 h, 98%; (b) tributyl(vinyl)stannane, $Pd(PPh_3)_4$, DMF, 90ºC, 24 h, 34%; (c) (1) *m*-CPBA, CH_2Cl_2, rt, 2 h; (2) NaOH, MeOH, rt, 16 h, 34%.

Scheme 12. Synthesis of **OX04963**[a]

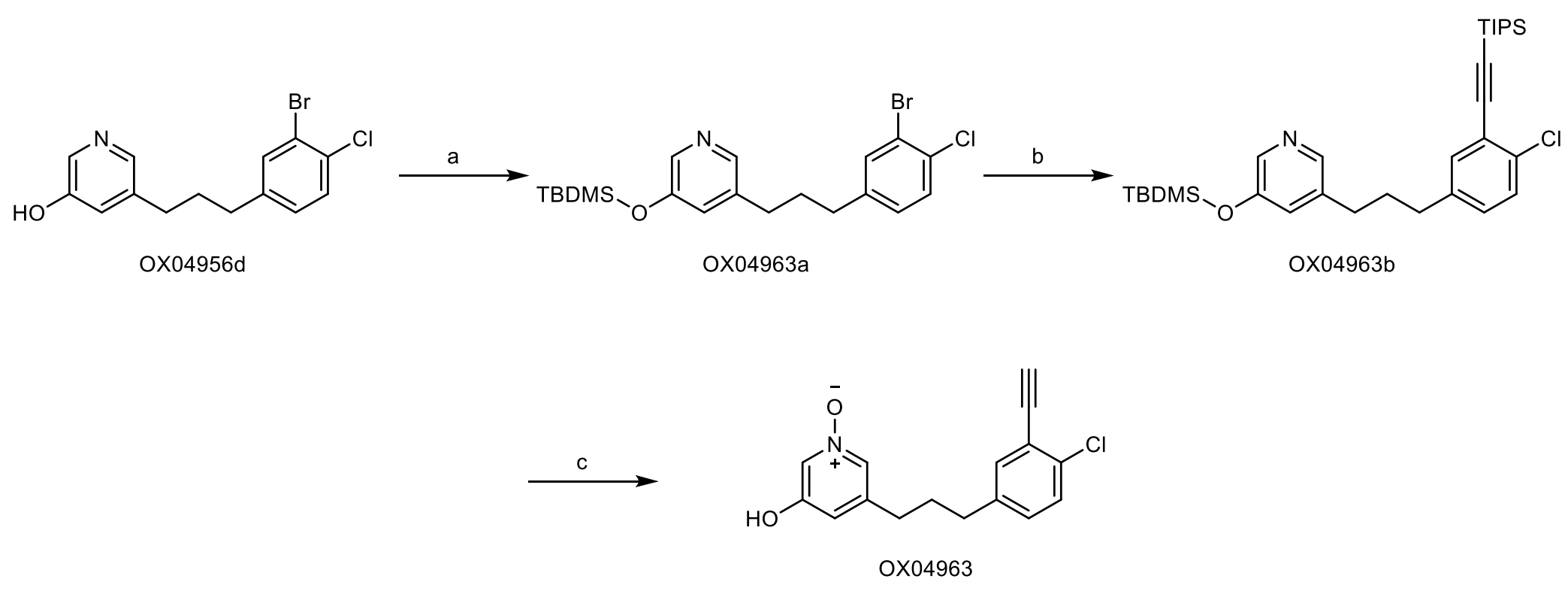

[a]Reagents and conditions: (a) TBDMSCl, imidazole, $CHCl_3$, 60ºC, 16 h, 26%; (b) ethynyltriisopropylsilane, PPh_3, $Pd_2(PPh_3)_2Cl_2$, CuI, THF:NEt_3 3:1, 60ºC, 16 h, 69%; (c) (1) *m*-CPBA, CH_2Cl_2, rt, 2 h; (2) TBAF, THF, rt, 16 h, 22%.

Scheme 13. Synthesis of **OX04964**[a]

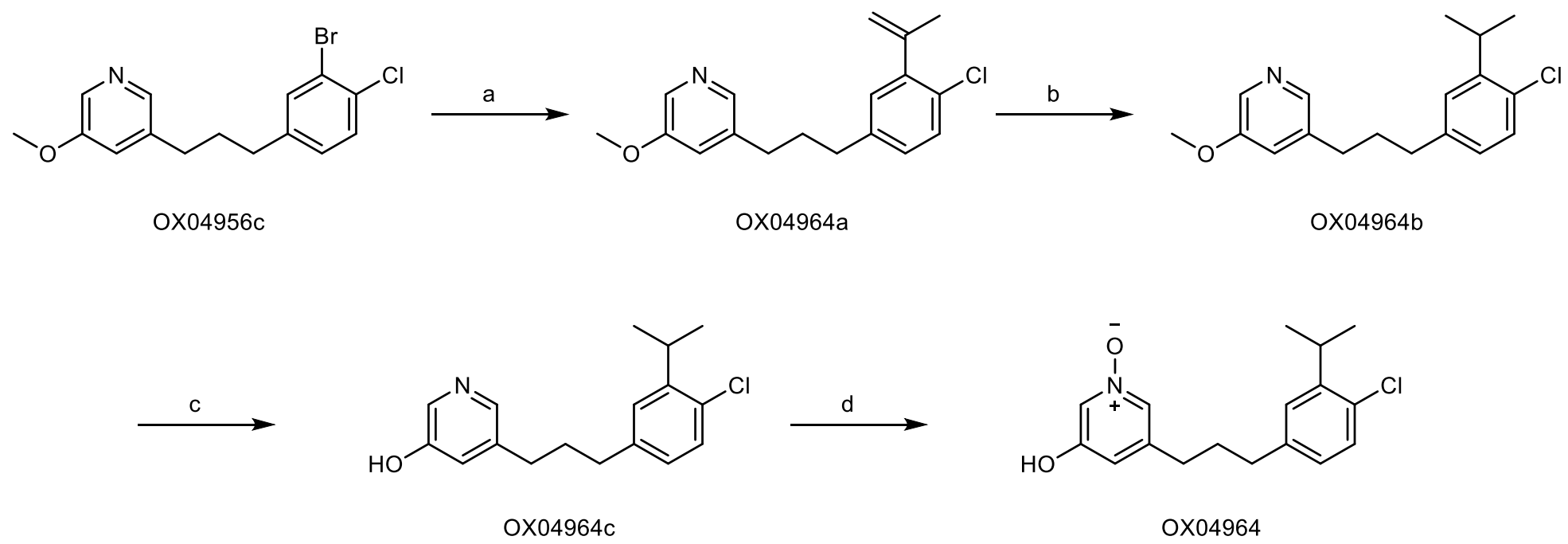

[a]Reagents and conditions: (a) tributyl(prop-1-en-2-yl)stannane, $Pd(PPh_3)_4$, DMF, 90ºC, 24 h, 39%; (b) PtO_2, H_2, MeOH, rt, 1 h, 64%; (c) 48% HBr, 120ºC, 48 h, 60%; (d) *m*-CPBA, CH_2Cl_2, rt, 2 h, 60%.

Scheme 14. Synthesis of **OX04965**[a]

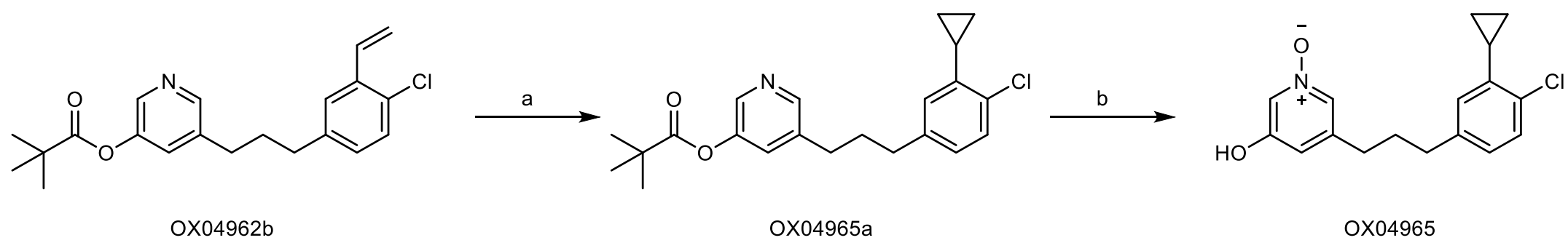

[a]Reagents and conditions: (a) (1) Et_2Zn, TFA, CH_2Cl_2, 0ºC, 30 min; (2) diiodomethane, CH_2Cl_2, 0ºC–rt, 16 h, 92%; (b) (1) *m*-CPBA, CH_2Cl_2, rt, 2 h; (2) NaOH, MeOH, rt, 16 h, 86%.

Scheme 15. Synthesis of **OX04966**[a]

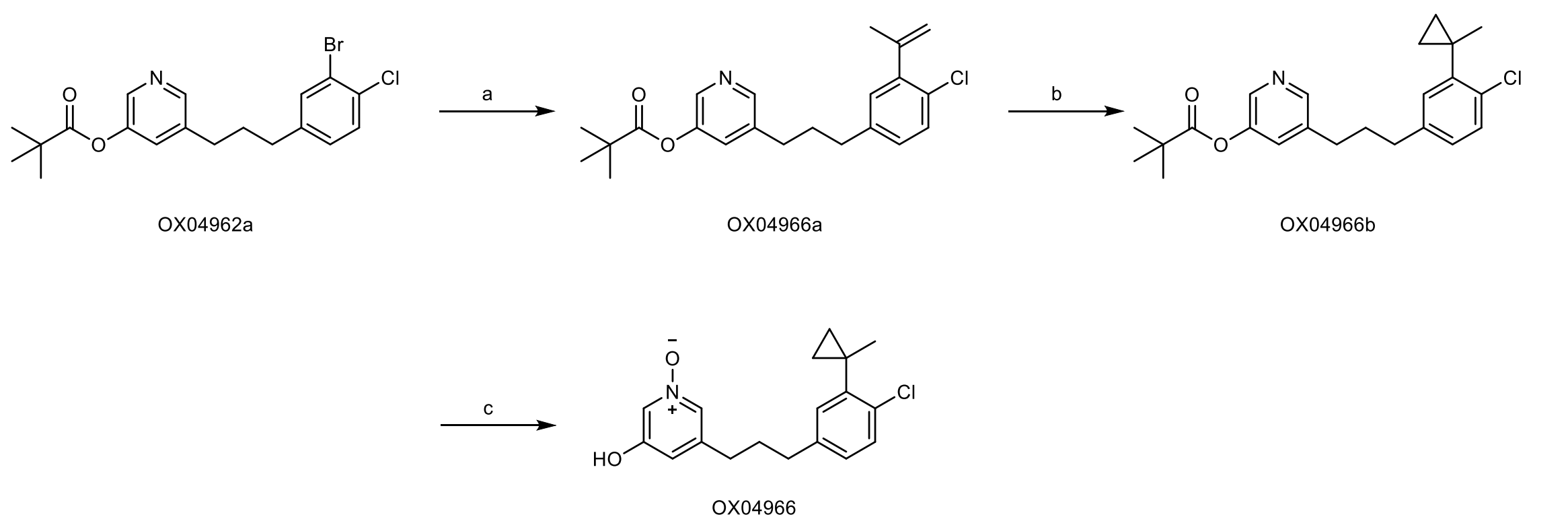

[a]Reagents and conditions: (a) tributyl(vinyl)stannane, $Pd(PPh_3)_4$, DMF, 90ºC, 24 h, 89%; (b) (1) Et_2Zn, TFA, CH_2Cl_2, 0ºC, 30 min; (2) diiodomethane, CH_2Cl_2, 0ºC–rt, 16 h, 31%; (c) (1) *m*-CPBA, CH_2Cl_2, rt, 2 h; (2) NaOH, MeOH, rt, 16 h, 99%.

OX04539a

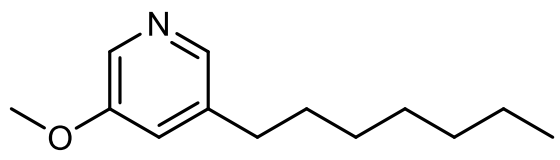

OX04539a

(1) To a solution of 1-bromohexane (825 mg, 5.0 mmol) in 20 mL toluene was added PPh_3 (1443 mg, 5.5 mmol), and the mixture was refluxed for 16 h. After completion, the mixture was concentrated under vacuum, redissolved in CH_2Cl_2 and purified by flash column chromatography (0–10% MeOH in CH_2Cl_2) to afford hexyltriphenylphosphonium bromide as a white solid (1351 mg, 63%).

(2) The solid (1042 mg, 3.0 mmol) was then dissolved in anhydrous CH_2Cl_2 (30 mL), NaOtBu (577 mg, 6.0 mmol) was added at 0ºC in one portion and the mixture was stirred for 15 minutes before adding 5-methoxynicotinaldehyde (274 mg, 2.0 mmol). The mixture was allowed to ward to rt and stirred for 16 h. After completion, the reaction mixture was diluted with CH_2Cl_2, washed with aq. sat. NH_4Cl solution, brine, dried (Na_2SO_4) and concentrated *in vacuo*. Purification by flash column chromatography (0–30% EtOAc in Pentane) afforded 3-heptyl-5-methoxypyridine as a transparent oil (400 mg, 91%).

(3) The oil (400 mg) was then dissolved in MeOH and the solution was degassed with N_2. 40 mg Pd/C (10 wt.%) was added and the atmosphere was replaced with H_2. The reaction was stirred for 1 h, filtered through Celite®, and dried under vacuum to afford **OX04539a** as a transparent oil (400 mg, 99%). ^{1}H NMR (400 MHz, $CDCl_3$) δ 8.12 (d, J = 2.8 Hz, 1H), 8.05 (d, J = 1.8 Hz, 1H), 6.99 (dd, J = 2.8, 1.8 Hz, 1H), 3.83 (s, 3H), 2.57 (dd, J = 8.7, 6.8 Hz, 2H), 1.66–1.54 (m, 2H), 1.37–1.19 (m, 8H), 0.90–0.83 (m, 3H). ^{13}C NMR (101 MHz, $CDCl_3$) δ 155.7, 142.6, 138.9, 134.7, 120.8, 55.6, 32.0, 31.9, 31.2, 29.3, 29.2, 22.7, 14.2. HRMS (ESI +ve) $C_{13}H_{21}NO$ $[M+H]^+$ calc. 208.1696, found 208.1693.

OX04539b

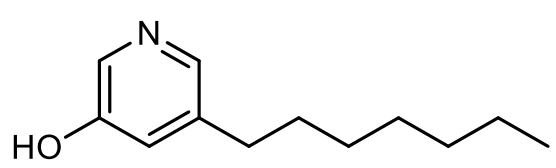

OX04539b

The demethylation using L-Selectride was adapted from a reported route.[1] L-Selectride (6 mL, 1 M in THF) was added to **OX04539a** (400 mg, 1.90 mmol) in THF (10 mL) and then the mixture was stirred at 70ºC for 48 h. After completion, the mixture was cooled to rt, quenched with MeOH, stirred for 30 min and concentrated *in vacuo*. The residue was then purified by flash column chromatography (0–8% MeOH in CH_2Cl_2) to afford **OX04539b** as a yellow oil (146 mg, 39%). ^{1}H NMR (400 MHz, $CDCl_3$) δ 8.73 (d, J = 2.5 Hz, 1H), 8.04 (d, J = 1.6 Hz, 1H), 7.89 (t, J = 2.0 Hz, 1H), 2.72 (t, J = 7.8 Hz, 2H), 1.71–1.58 (m, 2H), 1.50–1.02 (m, 8H), 0.90–0.79 (m, 3H). ^{13}C NMR (101 MHz, $CDCl_3$) δ 157.0, 144.1, 133.4, 130.9, 126.9, 32.9, 31.7, 30.4, 29.0, 29.0, 22.6, 14.1. HRMS (ESI +ve) $C_{12}H_{19}NO$ $[M+H]^+$ calc. 194.1539, found 194.1534.

OX04539

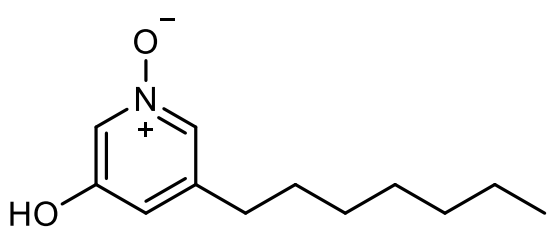

OX04539

To a solution of **OX04539b** (146 mg, 0.76 mmol) in CH_2Cl_2 (20 mL) at rt was added *m*-CPBA (1.5 eq.) and the resulting mixture stirred for 2 h. After completion, the reaction was quenched with $NaHCO_3$ (sat, aq.). The organic phase was washed with brine, dried (Na_2SO_4), filtered and concentrated *in vacuo*. Purification by flash column chromatography (0–6% MeOH in CH_2Cl_2) afforded **OX04539** as a yellow oil (87 mg, 55%). ^1H NMR (400 MHz, MeOD) δ 7.67 (q, *J* = 1.7 Hz, 2H), 6.87 (t, *J* = 1.6 Hz, 1H), 2.53–2.45 (m, 2H), 1.58–1.46 (m, 2H), 1.25–1.20 (m, 8H), 0.84–0.76 (m, 3H). ^{13}C NMR (101 MHz, MeOD) δ 157.8, 144.1, 131.8, 126.9, 119.5, 33.5, 32.9, 31.6, 30.1, 30.0, 23.7, 14.4. HRMS (ESI +ve) $C_{12}H_{19}NO_2$ [M+H$^+$] calc. 210.1489, found 210.1487. HPLC 98% (AUC), t_R = 5.1 min.

OX04540a

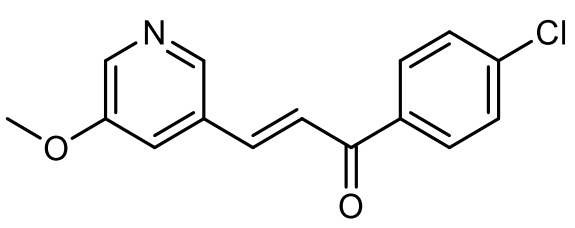

OX04540a

To a solution of 5-methoxynicotinaldehyde (500 mg, 3.65 mmol) and 1-(4-chlorophenyl)ethan-1-one (562 mg, 3.65 mmol) in 1,4-dioxane (10 mL) was added $BF_3{\cdot}Et_2O$ (1.9 mL) under N_2 and then the mixture was stirred at 105ºC for 16 h. After completion of the reaction, cooled to rt, the mixture was diluted with sat. aq. $NaHCO_3$ (50 mL) and extracted with EtOAc (3 × 30 mL). The combined organic extracts were washed with brine (100 mL), dried over Na_2SO_4, filtered, and concentrated under reduced pressure. The resulting residue was chromatographed over silica gel (0 − 60% EtOAc in pentane) to give **OX04540a** as a white solid (700 mg, 70%). ^1H NMR (400 MHz, $CDCl_3$) δ 8.46 (d, *J* = 1.7 Hz, 1H), 8.35 (d, *J* = 2.8 Hz, 1H), 8.00–7.93 (m, 2H), 7.77 (d, *J* = 15.8 Hz, 1H), 7.56–7.46 (m, 3H), 7.39 (dd, *J* = 2.8, 1.8 Hz, 1H), 3.92 (s, 3H). ^{13}C NMR (101 MHz, $CDCl_3$) δ 188.7, 155.9, 142.6, 141.6, 139.8, 139.7, 136.2, 131.2, 130.1, 129.2, 123.6, 118.4, 77.5, 77.2, 76.8, 55.9. HRMS (ESI+ve) $C_{15}H_{12}ClNO_2$ [M+H$^+$] calc. 274.0629, found 274.0623.

OX04540b

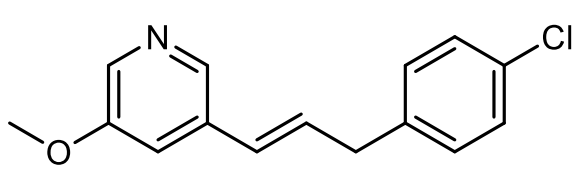

OX04540b

OX04540a (273 mg, 1.0 mmol) was dissolved in TFA (6 mL) using sonication. Triethylsilane (12 mL) was added and the reaction was vigorously stirred for 48 h. Upon completion, the reaction mixture was poured into sat. aq. $NaHCO_3$ and stirred until bubbling ceased. This solution was taken up into a separatory funnel and extracted with CH_2Cl_2 (3 × 40 mL), washed with brine, and dried under Na_2SO_4. Organic extracts were

dried under vacuum. Purification by flash column chromatography (30% EtOAc in Pentane) afforded **OX04540b** as a transparent oil (627 mg, 59%). ^{1}H NMR (400 MHz, $CDCl_3$) δ 8.10 (dd, *J* = 8.6, 2.3 Hz, 2H), 7.25–7.17 (m, 2H), 7.11–7.06 (m, 3H), 6.34–6.29 (m, 2H), 3.77 (s, 3H), 3.48–3.43 (m, 2H). ^{13}C NMR (101 MHz, $CDCl_3$) δ 155.8, 140.8, 138.0, 136.5, 133.5, 132.3, 131.4, 130.1, 129.8, 128.8, 128.6, 127.9, 116.8, 55.6, 38.7.

OX04540c

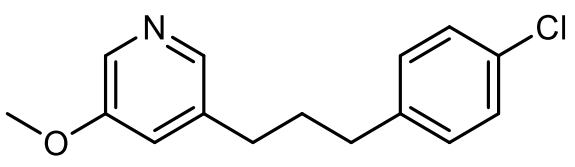

OX04540c

40 mg Pd/C (10% M/w) was added in one portion to a N_2 degassed solution of the **OX04540b** (400 mg, 1.5 mmol) in MeOH (50 mL) at rt, the atmosphere was replaced with H_2, and the reaction was stirred for 1 h. After completion, the reaction was filtered through Celite® using MeOH as an eluent, concentrated *in vacuo* and purified by flash column chromatography (30% EtOAc in Pentane) to afford the **OX04540c** as a yellow oil (320 mg, 79%). ^{1}H NMR (400 MHz, $CDCl_3$) δ 8.15 (d, *J* = 2.8 Hz, 1H), 8.06 (d, *J* = 1.8 Hz, 1H), 7.28–7.21 (m, 2H), 7.13–7.05 (m, 2H), 6.98 (dd, *J* = 2.7, 1.8 Hz, 1H), 3.84 (s, 3H), 2.61 (td, *J* = 7.7, 3.0 Hz, 4H), 1.99–1.87 (m, 2H). ^{13}C NMR (101 MHz, $CDCl_3$) δ 155.7, 142.5, 140.2, 138.0, 135.0, 131.8, 129.9, 128.6, 120.8, 55.6, 34.7, 32.5, 32.3. HRMS (ESI+ve) $C_{15}H_{16}ClNO$ [M+H$^+$] calc. 262.0993, found 262.0990.

OX04540d

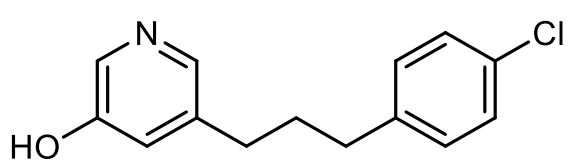

OX04540d

3 mL 48% HBr in H_2O was added to **OX04540c** (100 mg, 0.4 mmol) and then the mixture was stirred at 120ºC for 48 h. After completion of the reaction, cooled to rt, the mixture was neutralized with sat. aq. $NaHCO_3$ and extracted with EtOAc (3 × 30 mL). The combined organic extracts were washed with brine (100 mL), dried over Na_2SO_4, filtered, and concentrated under reduced pressure. The resulting residue was purified by flash column chromatography (5% MeOH in CH_2Cl_2) afforded the **OX04540d** as a transparent oil (22 mg, 23%). ^{1}H NMR (400 MHz, $CDCl_3$) δ 8.27–8.21 (m, 1H), 8.02 (s, 1H), 7.88 (s, 1H), 7.39 (t, *J* = 2.0 Hz, 1H), 7.26–7.22 (m, 2H), 7.11–7.06 (m, 2H), 2.63 (q, *J* = 7.1 Hz, 4H), 1.92 (dq, *J* = 9.3, 7.7 Hz, 2H). ^{13}C NMR (101 MHz, $CDCl_3$) δ 155.2, 140.1, 139.7, 139.6, 134.1, 131.8, 129.9, 128.7, 125.1, 34.7, 32.3, 32.2. HRMS (ESI+ve) $C_{14}H_{14}ClNO$ [M+H$^+$] calc. 248.0837, found 248.0835.

OX04540

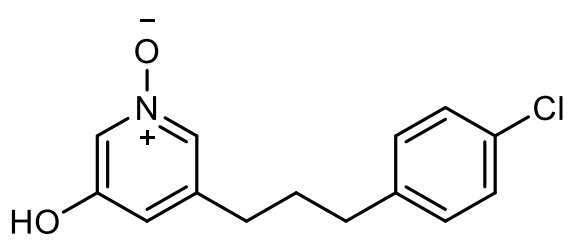

OX04540

To a solution of **OX04540d** (20 mg, 0.08 mmol) in CH_2Cl_2 (10 mL) at rt was added *m*-CPBA (1.5 eq.) and the resulting mixture stirred for 2 h. After completion, the reaction was quenched with $NaHCO_3$ (sat, aq.). The organic phase was washed with brine, dried (Na_2SO_4), filtered and concentrated *in vacuo*. Purification by flash column chromatography (0–100% EtOAc in Pentane then 0–6% MeOH in CH_2Cl_2) afforded **OX04540** as a yellow oil (6 mg, 28%). ^{1}H NMR (600 MHz, $CDCl_3$) δ 8.06 (t, *J* = 1.9 Hz, 1H), 7.63 (t, *J* = 1.5 Hz, 1H), 7.26–7.23 (m, 2H), 7.10–7.05 (m, 2H), 6.98 (t, *J* = 1.7 Hz, 1H), 2.60 (t, *J* = 7.6 Hz, 2H), 2.56–2.50 (m, 2H), 1.89 (tt, *J* = 9.2, 6.8 Hz, 2H). ^{13}C NMR (151 MHz, $CDCl_3$) δ 157.6, 141.7, 139.6, 132.1, 129.8, 129.7, 128.8, 126.6, 120.7, 34.5, 32.2, 31.7. HRMS (ESI +ve) $C_{14}H_{14}ClNO_2$ [M+H$^+$] calc. 264.0786, found 264.0784. HPLC 98% (AUC), t_R = 6.3 min.

OX04954a

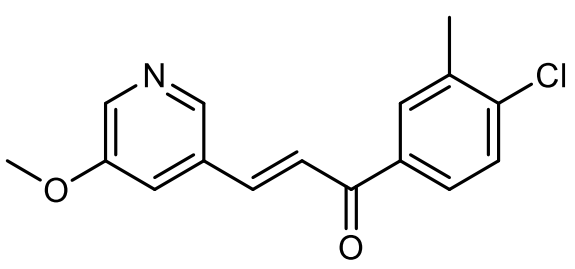

OX04954a

To a solution of 5-methoxynicotinaldehyde (609 mg, 4.45 mmol) and 1-(4-chloro-3-methylphenyl)ethan-1-one (500 mg, 2.96 mmol) in 1,4-dioxane (8 mL) was added $BF_3·Et_2O$ (1.5 mL) under N_2 and then the mixture was stirred at 105ºC for 16 h. After completion of the reaction, cooled to rt, the mixture was diluted with sat. aq. $NaHCO_3$ (50 mL) and extracted with EtOAc (3 × 30 mL). The combined organic extracts were washed with brine (100 mL), dried over Na_2SO_4, filtered, and concentrated under reduced pressure. The resulting residue was chromatographed over silica gel (0 − 60% EtOAc in pentane) to give **OX04954a** as a white solid (720 mg, 85%). ^{1}H NMR (400 MHz, $CDCl_3$) δ 8.47 (d, J = 1.7 Hz, 1H), 8.35 (d, J = 2.8 Hz, 1H), 7.89 (dd, J = 2.2, 0.9 Hz, 1H), 7.81–7.73 (m, 2H), 7.52 (d, J = 15.7 Hz, 1H), 7.48 (d, J = 8.3 Hz, 1H), 7.43–7.37 (m, 1H), 3.93 (s, 3H), 2.47 (s, 3H). ^{13}C NMR (101 MHz, $CDCl_3$) δ 189.0, 155.9, 142.5, 141.4, 139.9, 139.5, 137.0, 136.3, 131.3, 131.1, 129.6, 127.4, 123.8, 118.6, 55.9, 20.3. LRMS (ESI +ve) 288 [M+H]+. HRMS (ESI +ve) $C_{16}H_{14}ClNO_2$ [M+H$^+$] calc. 288.0822, found 288.0786.

OX04954b

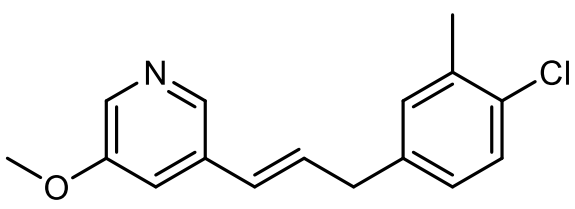

OX04954b

To a solution of **OX04954a** (700 mg, 2.43 mmol) in TFA (3.0 mL) was added Triethylsilane (3.1 mL) under N_2 and then the mixture was stirred at room temperature for 48 h. After completion of the reaction, the mixture was diluted with CH_2Cl_2 (50 mL), quenched with sat. aq. 1N NaOH (30 mL) and extracted with CH_2Cl_2 (3 × 30 mL). The combined organic extracts were washed with brine (100 mL), dried over Na_2SO_4, filtered, and concentrated under reduced pressure. The resulting residue was chromatographed over silica gel (0 − 60% EtOAc in pentane) to give **OX04954b** as a colourless oil (540 mg, 82%). ^1H NMR (400 MHz, $CDCl_3$) δ 8.17 (dd, J = 10.9, 2.3 Hz, 2H), 7.27 (d, J = 8.1 Hz, 1H), 7.15 (dd, J = 2.8, 1.8 Hz, 1H), 7.08 (d, J = 2.2 Hz, 1H), 6.99 (dd, J = 8.1, 2.3 Hz, 1H), 6.41–6.36 (m, 2H), 3.84 (s, 3H), 3.55–3.44 (m, 2H), 2.36 (s, 3H). ^{13}C NMR (101 MHz, $CDCl_3$) δ 155.8, 140.8, 138.1, 136.5, 136.2, 133.6, 132.4, 131.6, 131.4, 129.2, 127.7, 127.5, 116.8, 55.6, 38.8, 20.1. LRMS (ESI +ve) 274 [M+H]+. HRMS (ESI +ve) $C_{16}H_{16}ClNO$ $[M+H^+]$ calc. 274.0993, found 274.1040.

OX04954c

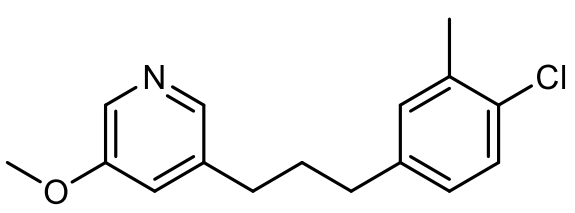

OX04954c

A mixture of **OX04954b** (500 mg, 1.82 mmol) and 50 mg PtO_2 (10% w/w) in MeOH (10 mL) was stirred at rt under an atmosphere of H_2 gas via balloon (1 atm of pressure) for 30 min. The mixture was filtered through a pad of Celite® with repeat rinsing using anhydrous MeOH. The filtrate was concentrated under reduced pressure, and the resulting crude residue was chromatographed over silica gel (0 − 40% EtOAc in pentane) to give **OX04954c** as a white solid (430 mg, 85%). ^1H NMR (400 MHz, $CDCl_3$) δ 8.14 (d, J = 2.8 Hz, 1H), 8.05 (d, J = 1.8 Hz, 1H), 7.22 (d, J = 8.1 Hz, 1H), 7.03–6.96 (m, 2H), 6.92 (dd, J = 8.2, 2.2 Hz, 1H), 3.83 (s, 3H), 2.59 (dt, J = 10.4, 7.6 Hz, 4H), 2.33 (s, 3H), 2.01–1.85 (m, 2H). ^{13}C NMR (101 MHz, $CDCl_3$) δ 155.7, 142.4, 140.3, 138.0, 135.9, 134.9, 131.8, 131.1, 129.0, 127.2, 120.7, 55.5, 34.6, 32.5, 32.3, 20.1. LRMS (ESI +ve) 276 [M+H]+. HRMS (ESI +ve) $C_{16}H_{18}ClNO$ $[M+H^+]$ calc. 276.1150, found 276.1181.

OX04954d

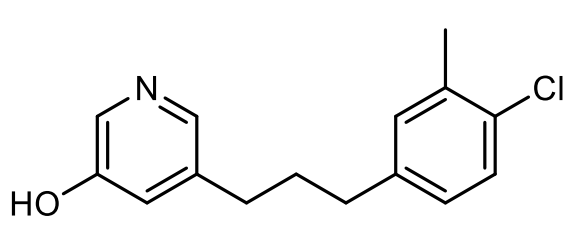

OX04954d

To 3-(3-(4-chloro-3-methylphenyl)propyl)-5-methoxypyridine **OX04954c (**250 mg, 0.906 mmol) in a vial was added 48% HBr in water (5 mL) and then the vial was sealed and the mixture was stirred at 120ºC for 48 h. After completion of the reaction, cooled to rt, the mixture was neutralized with sat. aq. $NaHCO_3$ and extracted with EtOAc (3 × 30 mL). The combined organic extracts were washed with brine (100 mL), dried over Na_2SO_4, filtered, and concentrated under reduced pressure. The resulting residue was chromatographed

over silica gel (0 − 10% MeOH in CH_2Cl_2) to give **OX04954d** as a white solid (187 mg, 79%). ^{1}H NMR (400 MHz, Acetone) δ 8.84 (s, 1H), 8.00 (dd, J = 34.7, 2.3 Hz, 2H), 7.31–7.13 (m, 2H), 7.11–7.00 (m, 2H), 2.61 (td, J = 7.8, 3.8 Hz, 4H), 2.32 (s, 3H), 1.97–1.88 (m, 2H). ^{13}C NMR (101 MHz, Acetone) δ 154.4, 142.0, 142.0, 139.1, 136.6, 136.3, 132.1, 132.0, 129.6, 128.3, 122.7, 35.2, 33.5, 32.7, 20.0. LRMS (ESI +ve) 262 [M+H]+. HRMS (ESI +ve) $C_{15}H_{16}ClNO$ [M+H$^+$] calc. 262.0993, found 262.1028.

OX04954

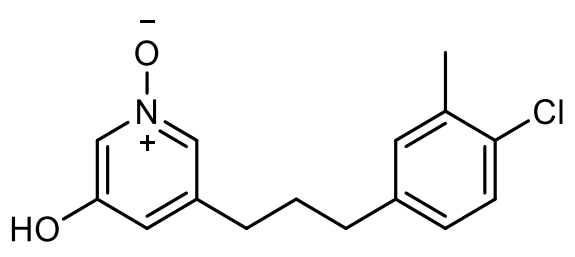

OX04954

To a solution of **OX04954a** (150 mg, 0.573 mmol) in CH_2Cl_2 (10 mL) at rt was added *m*-CPBA (1.5 eq.) and the resulting mixture stirred for 2 h. After completion, the reaction was quenched with $NaHCO_3$ (sat, aq.). The organic phase was washed with brine, dried (Na_2SO_4), filtered and concentrated *in vacuo*. Purification by flash column chromatography (0–6% MeOH in CH_2Cl_2) afforded **OX04954** as a white solid (138 mg, 87%). ^{1}H NMR (400 MHz, $CDCl_3$) δ 7.99 (t, J = 1.9 Hz, 1H), 7.62 (t, J = 1.5 Hz, 1H), 7.22 (d, J = 8.1 Hz, 1H), 7.00 (d, J = 2.2 Hz, 1H), 6.94–6.85 (m, 2H), 2.53 (dt, J = 16.2, 7.7 Hz, 4H), 2.37 (s, 3H), 1.95–1.81 (m, 2H). ^{13}C NMR (101 MHz, $CDCl_3$) δ 157.5, 141.6, 139.7, 136.1, 132.1, 131.1, 129.8, 129.1, 127.2, 126.6, 119.8, 34.4, 32.2, 31.8, 20.1. LRMS (ESI +ve) 278 [M+H]+. HRMS (ESI +ve) $C_{15}H_{16}ClNO_2$ [M+H$^+$] calc. 278.0942, found 278.0992. HPLC 97% (AUC), t_R = 7.4 min.

OX04956a

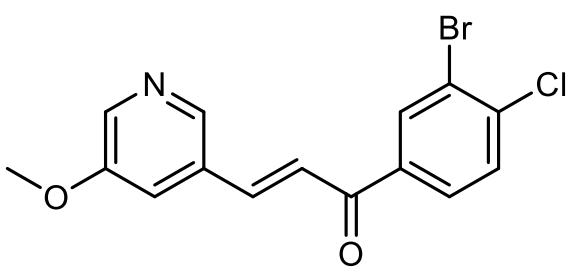

OX04956a

To a solution of 5-methoxynicotinaldehyde (1181 mg, 8.62 mmol) and 1-(3-bromo-4-chlorophenyl)ethan-1-one (1000 mg, 4.31 mmol) in 1,4-dioxane (10 mL) was added $BF_3\cdot Et_2O$ (2.2 mL) under N_2 and then the mixture was stirred at 105ºC for 16 h. After completion of the reaction, cooled to rt, the mixture was diluted with sat. aq. $NaHCO_3$ (50 mL) and extracted with EtOAc (3 × 30 mL). The combined organic extracts were washed with brine (100 mL), dried over Na_2SO_4, filtered, and concentrated under reduced pressure. The resulting residue was chromatographed over silica gel (0 − 60% EtOAc in pentane) to give **OX04956a** as a white solid (1168 mg, 29%). ^{1}H NMR (400 MHz, DMSO) δ 8.79 (d, *J* = 1.7 Hz, 1H), 8.52 (dd, *J* = 5.8, 2.4 Hz, 2H), 8.27 (dd, *J* = 2.7, 1.7 Hz, 1H), 8.22–8.14 (m, 2H), 7.90–7.80 (m, 2H), 3.97 (s, 3H). ^{13}C NMR (101 MHz, DMSO) δ 186.7, 156.4, 140.2, 140.2, 138.5, 137.2, 136.4, 133.7, 132.4, 131.1, 129.3, 124.8, 122.4, 122.3, 56.5, 39.9, 39.7, 39.5, 39.3, 39.1.

OX04956b

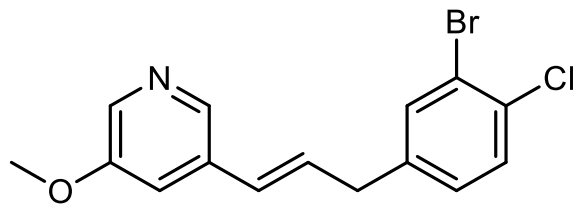

OX04956b

OX04956a (1100 mg, 3.1 mmol) was dissolved in TFA (10 mL) using sonication. Triethylsilane (20 mL) was added and the reaction was vigorously stirred for 48 h. Upon completion, the reaction mixture was poured into sat. aq. $NaHCO_3$ and stirred until bubbling ceased. This solution was taken up into a separatory funnel and extracted with CH_2Cl_2 (3 × 70 mL), washed with brine, and dried under Na_2SO_4. Organic extracts were dried under vacuum. Purification by flash column chromatography (30% EtOAc in Pentane) afforded **OX04956b** as a transparent oil (627 mg, 59%). ^{1}H NMR (400 MHz, $CDCl_3$) δ 8.17 (dd, *J* = 6.7, 2.3 Hz, 2H), 7.48 (d, *J* = 2.0 Hz, 1H), 7.37 (d, *J* = 8.2 Hz, 1H), 7.15 (t, *J* = 2.3 Hz, 1H), 7.10 (dd, *J* = 8.2, 2.1 Hz, 1H), 6.43–6.29 (m, 2H), 3.84 (s, 3H), 3.50 (d, *J* = 6.1 Hz, 2H). ^{13}C NMR (101 MHz, $CDCl_3$) δ 155.8, 140.8, 139.9, 136.6, 133.9, 133.3, 132.5, 130.5, 130.4, 128.9, 128.4, 122.6, 116.9, 55.6, 38.4. HRMS (ESI+ve) $C_{15}H_{13}BrClNO$ [M+H$^+$] calc. 337.9942, found 337.9936.

OX04956c

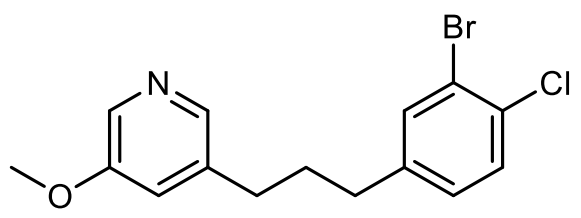

OX04956c

30 mg PtO2 was added in one portion to a N_2 degassed solution of the **OX04956b** (600 mg, 1.8 mmol) in MeOH (80 mL) at rt, the atmosphere was replaced with H_2, and the reaction stirred for 1 h. After completion, the reaction was filtered through Celite® using MeOH as an eluent, concentrated *in vacuo* and purified by flash column chromatography (30% EtOAc in Pentane) to afford **OX04956c** as a transparent oil (514 mg, 85%). ^{1}H NMR (400 MHz, $CDCl_3$) δ 8.12 (d, *J* = 2.8 Hz, 1H), 8.02 (d, *J* = 1.8 Hz, 1H), 7.40 (d, *J* = 2.1 Hz, 1H), 7.31 (d, *J* = 8.2 Hz, 1H), 7.02 (dd, *J* = 8.2, 2.1 Hz, 1H), 6.96 (dd, *J* = 2.7, 1.8 Hz, 1H), 3.82 (s, 3H), 2.58 (q, *J* = 7.3 Hz, 4H), 1.95–1.85 (m, 2H). ^{13}C NMR (101 MHz, $CDCl_3$) δ 155.7, 142.2, 142.1, 137.7, 134.0, 133.6, 131.8, 130.2, 128.6, 122.3, 120.7, 55.5, 34.3, 32.2. HRMS (ESI+ve) $C_{15}H_{15}BrClNO$ [M+H$^+$] calc. 340.0098, found 340.0094.

OX04956d

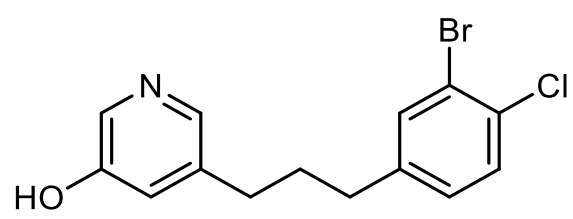

OX04956d

6 mL 48% HBr in H_2O was added to **OX04956c** (440 mg, 1.3 mmol) and then the mixture was stirred at 120ºC for 48 h. After completion of the reaction, cooled to rt, the mixture was neutralised with sat. aq. $NaHCO_3$ and extracted with EtOAc (3 × 40 mL). The combined organic extracts were washed with brine (100 mL), dried over Na_2SO_4, filtered, and concentrated under reduced pressure. The resulting residue was purified by flash column chromatography (5% MeOH in CH_2Cl_2) afforded **OX04956d** as a transparent oil (120 mg, 28%). ^{1}H NMR (400 MHz, MeOD) δ 7.92 (d, J = 2.7 Hz, 1H), 7.84 (d, J = 1.8 Hz, 1H), 7.48 (d, J = 2.0 Hz, 1H), 7.37 (d, J = 8.2 Hz, 1H), 7.13 (dd, J = 8.2, 2.1 Hz, 1H), 7.07 (dd, J = 2.6, 1.8 Hz, 1H), 2.59 (td, J = 7.7, 5.1 Hz, 4H), 1.96–1.82 (m, 2H). ^{13}C NMR (101 MHz, MeOD) δ 155.7, 144.1, 140.9, 140.3, 135.9, 134.7, 132.6, 131.3, 130.1, 124.4, 122.9, 35.2, 33.4, 32.9. HRMS (ESI+ve) $C_{14}H_{13}BrClNO$ [M+H$^+$] calc. 325.9942, found 325.9936.

OX04956

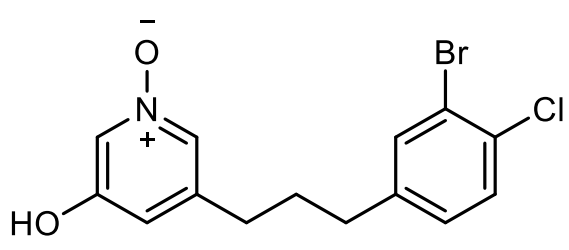

OX04956

To a solution of **OX04956d** (20 mg, 0.035 mmol) in CH_2Cl_2 (10 mL) at rt was added *m*-CPBA (1.5 eq.) and the resulting mixture stirred for 2 h. After completion, the reaction was quenched with $NaHCO_3$ (sat., aq.). The organic phase was washed with brine, dried (Na_2SO_4), filtered and concentrated *in vacuo*. Purification by flash column chromatography (0–100% EtOAc in Pentane then 0–6% MeOH in CH_2Cl_2) afforded **OX04956** as a yellow oil (11 mg, 52%). ^{1}H NMR (400 MHz, MeOD) δ 7.77 (d, J = 1.8 Hz, 2H), 7.53 (d, J = 2.1 Hz, 1H), 7.40 (d, J = 8.2 Hz, 1H), 7.17 (dd, J = 8.2, 2.1 Hz, 1H), 6.96 (t, J = 1.8 Hz, 1H), 2.62 (dt, J = 13.4, 7.7 Hz, 4H), 1.97–1.86 (m, 2H). ^{13}C NMR (101 MHz, MeOD) δ 157.8, 143.9, 143.4, 134.8, 132.8, 131.8, 131.3, 130.1, 127.1, 123.0, 119.4, 49.6, 49.4, 49.2, 49.0, 48.8, 48.6, 48.4, 35.1, 33.0, 32.8. HRMS (ESI +ve) $C_{14}H_{13}BrClNO_2$ [M+H$^+$] calc. 339.9745, found 339.9745. HPLC 98% (AUC), t_R = 7.5 min.

OX04955a

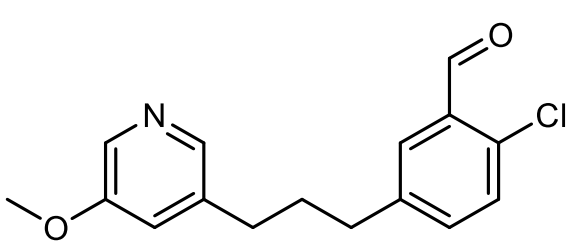

OX04955a

To a solution of **OX4956c** (555 mg, 1.6 mmol) in THF (4 mL) was added *n*-BuLi (1.4 mL, 2.5 M in hexane) dropwise over 10 min at -78ºC. The reaction was stirred for 5 min before adding DMF:THF mixture (1:9, 4 mL) in one portion at -78ºC. The reaction was stirred for 15 min then quenched with brine (5 mL) at -78ºC, and the mixture was allowed to warm to room temperature. The organic layer was washed with brine (8 mL

× 2), dried (Na_2SO_4), concentrated *in vacuo* and purified with flash column chromatography (10–30% EtOAc in petroleum ether) to afford the **OX04955a** as a transparent oil (212 mg, 45%). ^{1}H NMR (400 MHz, $CDCl_3$) δ 10.46 (s, 1H), 8.15 (d, *J* = 2.8 Hz, 1H), 8.05 (s, 1H), 7.73 (dd, *J* = 2.1, 0.7 Hz, 1H), 7.38–7.31 (m, 2H), 6.98 (dd, *J* = 2.7, 1.8 Hz, 1H), 3.84 (s, 3H), 2.65 (dt, *J* = 20.7, 7.7 Hz, 4H), 2.01–1.90 (m, 2H). ^{13}C NMR (101 MHz, $CDCl_3$) δ 190.1, 155.8, 142.4, 141.4, 137.7, 135.7, 135.5, 135.2, 132.4, 130.7, 129.0, 120.7, 55.6, 34.6, 32.3, 32.3. LRMS (ESI +ve) 290 $[M+H]^+$. HRMS (ESI +ve) $C_{16}H_{16}ClNO_2$ $[M+H]^+$ calc. 290.0942, found 290.0941.

OX04955b

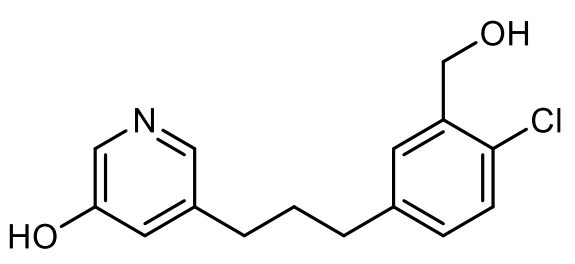

OX04955b

The demethylation using L-Selectride was adapted from a reported route.[1] L-Selectride (1.2 mL, 1 M in THF) was added to **OX04955a** (120 mg, 0.4 mmol) in THF (4 mL) and then the mixture was stirred at 70ºC for 48 h. After completion, the mixture was cooled to rt, quenched with MeOH, stirred for 30 min and concentrated *in vacuo*. The residue was then purified by flash column chromatography (0–8% MeOH in CH_2Cl_2) to afford **OX04955b** as a transparent oil (45 mg, 39%). ^{1}H NMR (400 MHz, MeOD) δ 7.91 (d, *J* = 2.6 Hz, 1H), 7.84 (d, *J* = 1.8 Hz, 1H), 7.37 (d, *J* = 2.2 Hz, 1H), 7.25 (d, *J* = 8.1 Hz, 1H), 7.11–7.03 (m, 2H), 4.67 (s, 2H), 2.62 (dt, *J* = 17.9, 7.8 Hz, 4H), 1.99–1.85 (m, 2H). ^{13}C NMR (101 MHz, MeOD) δ 155.7, 142.2, 140.9, 140.6, 139.8, 135.8, 130.8, 130.0, 129.6, 129.5, 124.3, 62.3, 35.7, 33.7, 33.0. LRMS (ESI +ve) 278 $[M+H]^+$. HRMS (ESI +ve) $C_{15}H_{16}ClNO_2$ $[M+H]^+$ calc. 278.0942, found 278.0932.

OX04955

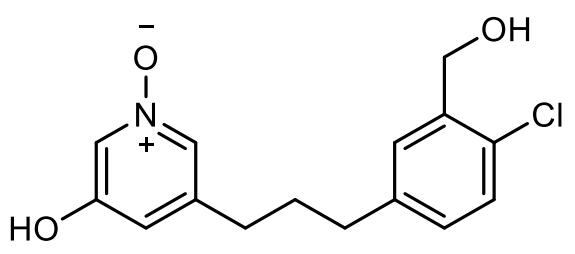

OX04955

To a solution of **OX04955b** (25 mg, 0.1 mmol) in CH_2Cl_2 (15 mL) at rt was added *m*-CPBA (1.5 eq.) and the resulting mixture stirred for 2 h. After completion, the reaction was quenched with $NaHCO_3$ (sat., aq.). The organic phase was washed with brine, dried (Na_2SO_4), filtered and concentrated *in vacuo*. Purification by flash column chromatography (0–10% MeOH in CH_2Cl_2) afforded **OX04955** as a yellow solid (17 mg, 64%). ^{1}H NMR (400 MHz, MeOD) δ 7.76 (p, *J* = 1.7 Hz, 2H), 7.38 (d, *J* = 2.2 Hz, 1H), 7.26 (d, *J* = 8.1 Hz, 1H), 7.10 (dd, *J* = 8.1, 2.3 Hz, 1H), 6.95 (dd, *J* = 2.1, 1.4 Hz, 1H), 4.67 (s, 2H), 2.68 (t, *J* = 7.6 Hz, 2H), 2.65–2.59 (m, 2H), 2.01–1.91 (m, 2H). ^{13}C NMR (151 MHz, MeOD) δ 157.8, 143.6, 141.9, 139.9, 131.9, 130.9, 130.1, 129.6, 129.5, 127.0, 119.4, 62.3, 35.5, 33.0, 33.0. LRMS (ESI +ve) 294 $[M+H]^+$. HRMS (ESI +ve) $C_{15}H_{16}ClNO_3$ $[M+H]^+$ calc. 294.0892, found 294.0890. HPLC 96% (AUC), t_R = 3.2 min.

OX04957a

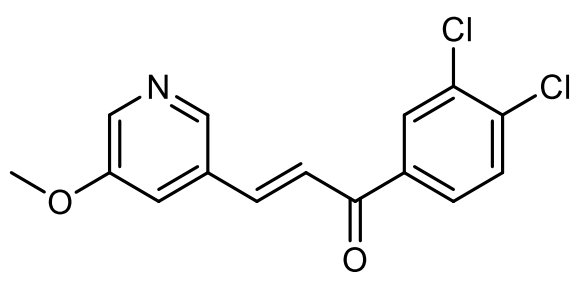

OX04957a

To a solution of 5-methoxynicotinaldehyde (1.00 g, 7.3 mmol) in 10 mL 1,4-dioxane was added 1-(3,4-dichlorophenyl)ethan-1-one (1.15 g, 6.1 mmol). $BF_3 \cdot OEt_2$ (6.0 mL) was added, and the mixture was heated at 105ºC for 16 h. After completion, the reaction was cooled to room temperature and slowly poured into aq. sat. $NaHCO_3$ and stirred until bubbling ceased. This solution was taken up into a separatory funnel and extracted with CH_2Cl_2. The organic layer was dried over Na_2SO_4 then under vacuum. To the crude residue, 5 mL MeOH was added. This mixture was diluted with pentane and allowed to stir for 20 minutes. The resulting suspension was filtered and washed with pentane to afford **OX04957a** as a yellow-brown solid (1.52 g, 67%). ^{1}H NMR (400 MHz, $CDCl_3$) δ 8.47 (d, *J* = 1.8 Hz, 1H), 8.36 (d, *J* = 2.8 Hz, 1H), 8.10 (d, *J* = 2.0 Hz, 1H), 7.85 (dd, *J* = 8.3, 2.1 Hz, 1H), 7.80 (d, *J* = 15.7 Hz, 1H), 7.60 (d, *J* = 8.4 Hz, 1H), 7.48 (d, *J* = 15.7 Hz, 1H), 7.41 (dd, *J* = 2.7, 2.0 Hz, 1H), 3.93 (s, 3H). ^{13}C NMR (101 MHz, $CDCl_3$) δ 187.6, 156.0, 142.7, 142.3, 139.9, 137.9, 137.4, 133.6, 131.0, 131.0, 130.7, 127.7, 123.1, 118.5, 55.9. HRMS (ESI +ve) $C_{15}H_{11}Cl_2NO_2$ $[M+H]^+$ calc. 308.0240, found 308.0255.

OX04957b

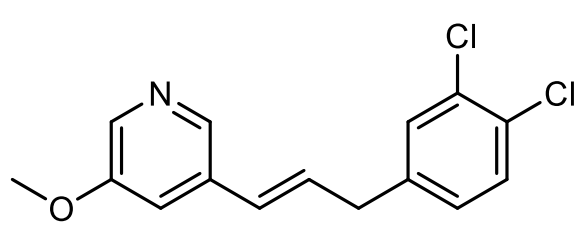

OX04957b

OX04957a (1.52 g, 4.9 mmol) was dissolved in TFA (10 mL) using sonication. Triethylsilane (20 mL) was added and the reaction was vigorously stirred for 48 h. Upon completion, the reaction mixture was poured into sat. aq. $NaHCO_3$ and stirred until bubbling ceased. This solution was taken up into a separatory funnel and extracted with CH_2Cl_2, washed with brine, and dried under Na_2SO_4. Organic extracts were dried under vacuum. Purification by flash column chromatography (0–50% EtOAc in pentane) afforded **OX04957b** as a pale yellow solid (547 mg, 38%). ^{1}H NMR (400 MHz, $CDCl_3$) δ 8.19–8.16 (m, 2H), 7.37 (d, *J* = 8.2 Hz, 1H), 7.31 (d, *J* = 2.1 Hz, 1H), 7.15 (dd, *J* = 2.8, 1.8 Hz, 1H), 7.08–7.03 (m, 1H), 6.41 (d, *J* = 15.9 Hz, 1H), 6.34 (dt, *J* = 15.8, 6.1 Hz, 1H), 3.85 (s, 3H), 3.51 (d, *J* = 6.0 Hz, 2H). ^{13}C NMR (101 MHz, $CDCl_3$) δ 155.5, 140.4, 139.5, 136.3, 133.0, 132.3, 130.4, 130.2, 130.2, 130.2, 128.1, 127.9, 116.6, 55.2, 38.1. HRMS (ESI +ve) $C_{15}H_{13}Cl_2NO$ $[M+H]^+$ calc. 294.0447, found 294.0449.

OX04957c

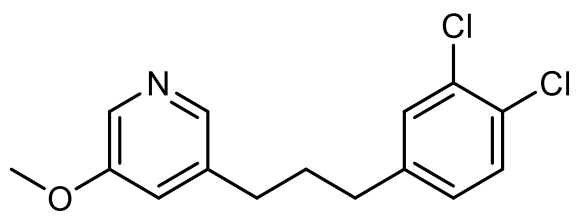

OX04957c

OX04957b (547 mg, 1.83 mmol) was dissolved in MeOH and the solution was degassed with N_2. PtO_2 (27 mg, 5 mol%) was added and the atmosphere was replaced with H_2. The reaction was stirred for 1h, filtered through Celite®, and dried under vacuum. Purification by flash column chromatography (0–40% EtOAc in pentane) afforded **OX04957c** as a pale yellow oil (411 mg, 76%). ^{1}H NMR (400 MHz, $CDCl_3$) δ 8.16 (d, *J* = 2.8 Hz, 1H), 8.06 (d, *J* = 1.8 Hz, 1H), 7.34 (d, *J* = 8.2 Hz, 1H), 7.26 (s, 1H), 7.00 (dd, *J* = 8.1, 2.1 Hz, 2H), 3.85 (s, 3H), 2.62 (d, *J* = 4.9 Hz, 4H), 1.99–1.89 (m, 2H). ^{13}C NMR (101 MHz, $CDCl_3$) δ 155.8, 142.3, 142.1, 137.8, 135.0, 132.4, 130.5, 130.5, 130.1, 128.0, 120.9, 55.7, 34.5, 32.3, 32.3. HRMS (ESI +ve) $C_{15}H_{15}Cl_2NO$ [M+H$^+$] calc. 296.0604, found 296.0609.

OX04957d

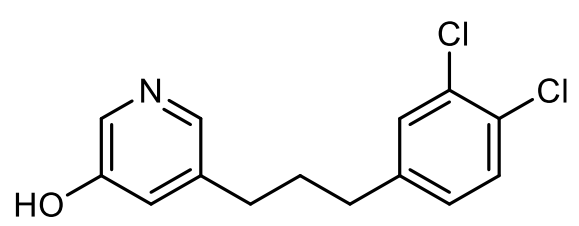

OX04957d

6 mL 48% HBr in H_2O was added to **OX04957c** (411 mg, 1.39 mmol), and the reaction was heated to 120℃ and stirred for 48 h. Upon completion, the reaction mixture was poured into aq. sat. $NaHCO_3$ and stirred until bubbling ceased. This solution was taken up into a separatory funnel and extracted with EtOAc, washed with brine, and dried under Na_2SO_4. Organic extracts were dried under vacuum. Purification by flash column chromatography (0–100% EtOAc in pentane) afforded **OX04957d** as a pale orange solid (180 mg, 46%). ^{1}H NMR (400 MHz, $CDCl_3$) δ 8.12 (d, *J* = 2.6 Hz, 1H), 7.91 (d, *J* = 1.8 Hz, 1H), 7.33 (d, *J* = 8.2 Hz, 1H), 7.25 (d, *J* = 2.1 Hz, 1H), 7.14 (t, *J* = 2.1 Hz, 1H), 6.99 (dd, *J* = 8.2, 2.1 Hz, 1H), 2.61 (td, *J* = 8.3, 3.4 Hz, 4H), 1.98–1.86 (m, 2H). ^{13}C NMR (101 MHz, $CDCl_3$) δ 155.4, 141.9, 139.6, 139.2, 133.9, 132.4, 130.5, 130.4, 130.1, 128.0, 125.3, 34.5, 32.2, 32.1. HRMS (ESI +ve) $C_{14}H_{13}Cl_2NO$ [M+H$^+$] calc. 282.0447, found 282.0459.

OX04957

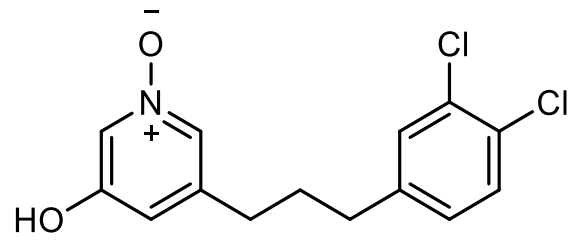

OX04957

To a solution of **OX04957d** (50 mg, 0.18 mmol) in CH_2Cl_2 (10 mL) was added 77% *meta*-chloroperoxybenzoic acid (60 mg, 0.27 mmol) and the reaction was stirred at room temperature for 2 h. Another portion of 77% *meta*-chloroperoxybenzoic acid (40 mg, 0.18 mmol) was added and the reaction was further stirred for 30 mins. Upon completion, aq. 2M NaOH (5 mL) was added to the reaction mixture. The

pH was adjusted to 3 – 4 using aq. 1M HCl, and a precipitate formed in the organic phase. The precipitate was filtered and washed with CH_2Cl_2 to afford **OX04957** as a white solid (12 mg, 23%). ^{1}H NMR (400 MHz, DMSO) δ 10.38 (s, 1H), 7.68 (t, *J* = 1.5 Hz, 1H), 7.61 (t, *J* = 1.9 Hz, 1H), 7.53 (d, *J* = 8.3 Hz, 1H), 7.51 (d, *J* = 2.1 Hz, 1H), 7.22 (dd, *J* = 8.2, 2.1 Hz, 1H), 6.67 (t, *J* = 1.7 Hz, 1H), 2.63–2.56 (m, 2H), 2.47 (t, *J* = 6.9 Hz, 2H), 1.84 (tt, *J* = 9.5, 6.6 Hz, 2H). ^{13}C NMR (101 MHz, $CDCl_3$) δ 157.6, 141.4, 141.3, 132.5, 130.6, 130.4, 130.3, 129.8, 128.0, 126.7, 119.8, 34.3, 32.2, 31.5. HRMS (ESI +ve) $C_{14}H_{13}Cl_2NO_2$ [M+H$^+$] calc. 298.0396, found 298.0408. HPLC 98% (AUC), t_R = 7.3 min.

OX04958a

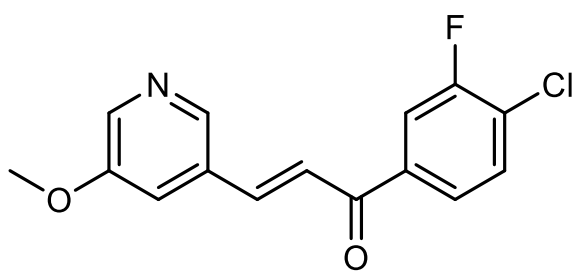

OX04958a

To a solution of 5-methoxynicotinaldehyde (1.00 g, 7.3 mmol) in 10 mL 1,4-dioxane was added 1-(4-chloro-3-fluorophenyl)ethan-1-one (1.05 g, 6.1 mmol). $BF_3{\cdot}OEt_2$ (6.0 mL) was added, and the mixture was heated to 105ºC for 16 h. After completion, the reaction was cooled to room temperature and slowly poured into aq. sat. $NaHCO_3$ and stirred until bubbling ceased. This solution was taken up into a separatory funnel and extracted with CH_2Cl_2. Organic extracts were dried under vacuum. To the crude residue, a small amount of MeOH was added. This mixture was diluted witpentane and allowed to stir for 20 minutes. The resulting suspension was filtered and washed with pentane to afford **OX04958a** as a yellow-brown solid (1.13 g, 64%). ^{1}H NMR (400 MHz, $CDCl_3$) δ 8.47 (d, *J* = 1.8 Hz, 1H), 8.36 (d, *J* = 2.8 Hz, 1H), 7.82–7.73 (m, 3H), 7.55 (dd, *J* = 8.2, 7.1 Hz, 1H), 7.48 (d, *J* = 15.7 Hz, 1H), 7.42–7.38 (m, 1H), 3.93 (s, 3H). ^{13}C NMR (101 MHz, $CDCl_3$) δ 187.5, 158.4 (d, *J* = 251.3 Hz), 156.0, 142.6, 142.3, 139.9, 138.1 (d, *J* = 5.4 Hz), 131.2, 131.0, 126.7 (d, *J* = 17.9 Hz), 125.0 (d, *J* = 3.7 Hz), 118.5, 116.7 (d, *J* = 22.1 Hz), 116.6, 55.9. ^{19}F NMR (376 MHz, $CDCl_3$) δ -113.42 (dd, *J* = 9.4, 7.1 Hz). HRMS (ESI +ve) $C_{15}H_{11}ClFNO_2$ [M+H]$^+$ calc. 292.0535, found 292.0532.

OX04958b

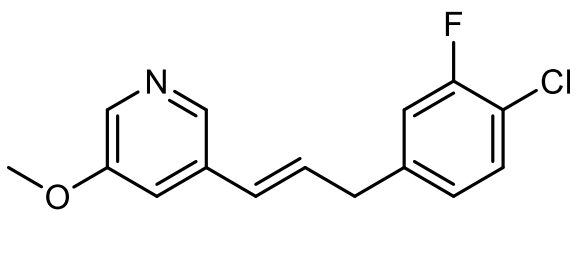

OX04958b

OX04958a (1.13 g, 3.9 mmol) was dissolved in TFA (8 mL) using sonication. Triethylsilane (16 mL) was added, and the reaction was vigorously stirred for 48 h. Upon completion, the reaction mixture was poured into aq. sat. $NaHCO_3$ and stirred until bubbling ceased. This solution was taken up into a separatory funnel and extracted with CH_2Cl_2, washed with brine, and dried under Na_2SO_4. Organic extracts were dried under vacuum. Purification by flash column chromatography (0–40% EtOAc in pentane) afforded **OX04958b** as a pale yellow solid (406 mg, 38%). ^{1}H NMR (400 MHz, $CDCl_3$) δ 8.19 (d, *J* = 1.8 Hz, 1H), 8.17 (d, *J* = 2.8 Hz,

1H), 7.33 (t, J = 7.9 Hz, 1H), 7.16 (dd, J = 2.8, 1.8 Hz, 1H), 7.02 (dd, J = 9.9, 2.0 Hz, 1H), 6.96 (ddd, J = 8.2, 2.0, 0.8 Hz, 1H), 6.42 (d, J = 16.0 Hz, 1H), 6.35 (dt, J = 15.8, 6.0 Hz, 1H), 3.86 (s, 3H), 3.54 (d, J = 5.9 Hz, 2H). ^{13}C NMR (101 MHz, $CDCl_3$) δ 158.1 (d, J = 248.8 Hz), 155.8, 140.6, 140.4 (d, J = 6.3 Hz), 136.5, 133.3, 130.6, 130.5, 128.3, 125.1 (d, J = 3.5 Hz), 118.9 (d, J = 8.1 Hz), 116.9, 116.9 (d, J = 21.2 Hz), 55.6, 38.6 (d, J = 1.6 Hz). ^{19}F NMR (376 MHz, $CDCl_3$) δ -115.44 (dd, J = 10.0, 7.6 Hz). HRMS (ESI +ve) $C_{15}H_{13}ClFNO$ $[M+H]^+$ calc. 278.0743, found 278.0736.

OX04958c

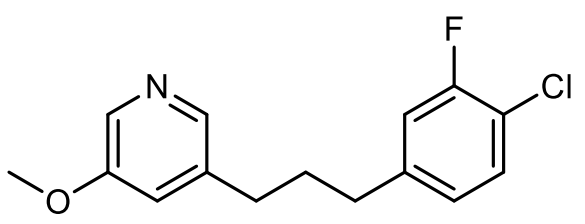

OX04958c

OX04958b (406 mg, 1.46 mmol) was dissolved in MeOH, and the solution was degassed with N_2. PtO_2 (20 mg, 5 mol%) was added and the atmosphere was replaced with H_2. The reaction was stirred for 1h, filtered through Celite®, and dried under vacuum to afford **OX04958c** as pale yellow oil (380 mg, 93%). ^{1}H NMR (400 MHz, $CDCl_3$) δ 8.16 (d, J = 2.8 Hz, 1H), 8.06 (d, J = 1.7 Hz, 1H), 7.29 (t, J = 7.9 Hz, 1H), 7.00–6.98 (m, 1H), 6.96 (dd, J = 10.1, 2.0 Hz, 1H), 6.89 (ddd, J = 8.2, 2.1, 0.8 Hz, 1H), 3.85 (s, 3H), 2.63 (dd, J = 7.4, 1.7 Hz, 4H), 1.99–1.88 (m, 2H). ^{13}C NMR (151 MHz, $CDCl_3$) δ 158.2 (d, J = 248.5 Hz), 155.8, 142.8 (d, J = 6.5 Hz), 142.4, 137.8, 135.1, 130.5, 125.0 (d, J = 3.4 Hz), 120.9, 118.4 (d, J = 17.7 Hz), 116.6 (d, J = 20.4 Hz), 55.7, 34.7, 32.3, 32.3. ^{19}F NMR (376 MHz, $CDCl_3$) δ -115.94 (dd, J = 10.1, 7.6 Hz). HRMS (ESI +ve) $C_{15}H_{15}ClFNO$ $[M+H^+]$ calc. 280.0899, found 280.0913.

OX04958d

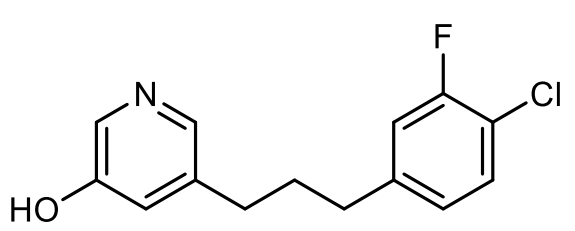

OX04958d

5 mL 48% HBr in H_2O was added to **OX04958c** (380 mg, 1.36 mmol), and the reaction was heated to 120℃ and stirred for 48 h. Upon completion, the reaction mixture was poured into aq. sat. $NaHCO_3$ and stirred until bubbling ceased. This solution was taken up into a separatory funnel and extracted with EtOAc, washed with brine, and dried under Na_2SO_4. Organic extracts were dried under vacuum. Purification by flash column chromatography (0–100% EtOAc in pentane) afforded **OX04958d** as a pale orange oil (63.8 mg, 18%). ^{1}H NMR (400 MHz, $CDCl_3$) δ 8.15–8.11 (m, 1H), 7.92 (s, 1H), 7.28 (t, J = 8.0 Hz, 1H), 7.15 (t, J = 2.1 Hz, 1H), 6.95 (dd, J = 10.1, 2.0 Hz, 1H), 6.88 (ddd, J = 8.1, 2.0, 0.8 Hz, 1H), 2.64 (d, J = 2.8 Hz, 2H), 2.60 (d, J = 2.9 Hz, 2H), 1.99–1.87 (m, 2H). ^{13}C NMR (101 MHz, $CDCl_3$) δ 158.1 (d, J = 248.6 Hz), 155.4, 142.6 (d, J = 6.5 Hz), 139.6, 139.1, 133.9, 130.6, 125.4, 125.0 (d, J = 3.4 Hz), 118.5 (d, J = 17.5 Hz), 116.6 (d, J = 20.5 Hz),

34.6, 32.2, 32.1. ^{19}F NMR (376 MHz, $CDCl_3$) δ -115.85 (dd, *J* = 10.1, 7.6 Hz). HRMS (ESI +ve) $C_{14}H_{13}ClFNO$ [M+H$^+$] calc. 266.0743, found 266.0751.

OX04958

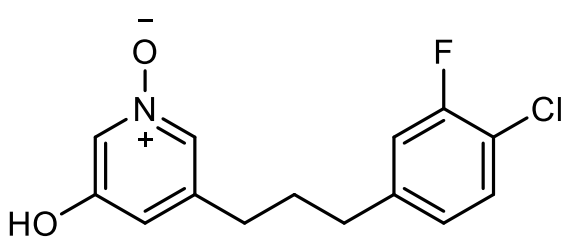

OX04958

To a solution of **OX04958c** (60 mg, 0.23 mmol) in CH_2Cl_2 (10 mL). 77% *meta*-chloroperoxybenzoic acid (83 mg, 0.36 mmol) was added, and the reaction was stirred at room temperature for 2 h. Upon completion, aq. 2M NaOH (5 mL) was added to the reaction mixture. The pH was adjusted to 3-4 using aq. 1M HCl, and the aqueous phase was extracted with 2:1 $CHCl_3$:EtOH. The organic extract was dried and purified by flash column chromatography (0–5% MeOH in CH_2Cl_2) to afford **OX04958** as a pale yellow solid (40.3 mg, 65%). ^{1}H NMR (400 MHz, $CDCl_3$) δ 8.00 (d, *J* = 2.1 Hz, 1H), 7.64 (s, 1H), 7.28 (t, *J* = 7.2 Hz, 1H), 6.94–6.90 (m, 2H), 6.86 (dd, *J* = 8.2, 2.0 Hz, 1H), 2.60 (t, *J* = 7.6 Hz, 2H), 2.53 (t, *J* = 7.7 Hz, 2H), 1.93–1.84 (m, 2H). ^{13}C NMR (101 MHz, $CDCl_3$) δ 158.1 (d, *J* = 249.5 Hz), 157.5, 142.1 (d, *J* = 6.4 Hz), 141.3, 130.6, 129.8, 126.7, 124.9 (d, *J* = 3.5 Hz), 119.8, 118.6 (d, *J* = 17.7 Hz), 116.6 (d, *J* = 20.6 Hz), 34.4, 32.1, 31.4. ^{19}F NMR (377 MHz, $CDCl_3$) δ -115.60 (dd, *J* = 10.1, 7.8 Hz). HRMS (ESI +ve) $C_{14}H_{13}ClFNO_2$ [M+H$^+$] calc. 282.0692, found 282.0701. HPLC 96% (AUC), t_R = 6.5 min.

OX04959a

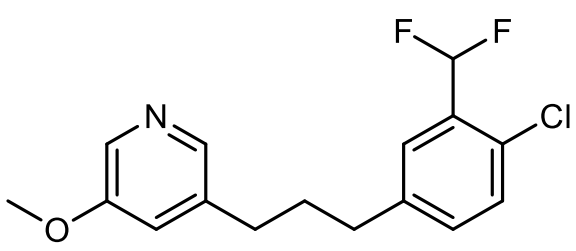

OX04959a

To a solution of **OX04955a** (100 mg, 0.35 mmol) in CH_2Cl_2 (8 mL) was added DAST (0.11 mL, 0.84 mmol) dropwise. The reaction was stirred for 16 h at rt before quenched with $NaHCO_3$ (10 mL, sat., aq.) and the two phases were separated. The organic layer was washed with $NaHCO_3$ (10 mL, sat., aq.), brine (15 mL), dried (Na_2SO_4), filtered and concentrated *in vacuo*. The residue was purified by flash column chromatography (10–30% EtOAc in petroleum ether) to afford **OX04959a** as a yellow oil (40 mg, 37%). ^{1}H NMR (400 MHz, $CDCl_3$) δ 8.16 (d, *J* = 2.8 Hz, 1H), 8.06 (d, *J* = 1.8 Hz, 1H), 7.47 (d, *J* = 2.2 Hz, 1H), 7.33 (dt, *J* = 8.3, 1.2 Hz, 1H), 7.21 (ddt, *J* = 8.2, 1.9, 0.9 Hz, 1H), 7.09–6.77 (m, 2H), 3.85 (s, 3H), 2.65 (dt, *J* = 17.4, 7.7 Hz, 4H), 2.03–1.91 (m, 2H). ^{13}C NMR (101 MHz, $CDCl_3$) δ 155.8, 142.4, 141.3, 137.7, 135.2, 132.1 (t, *J* = 2.0 Hz), 131.7, 130.3, 130.0, 126.8 (t, *J* = 5.8 Hz), 120.8, 112.2 (t, *J* = 238.0 Hz), 55.6, 34.7, 32.4, 32.3. ^{19}F NMR (376 MHz, $CDCl_3$) δ -115.0. LRMS (ESI +ve) 312 [M+H]$^+$. HRMS (ESI +ve) $C_{16}H_{16}ClF_2NO$ [M+H]$^+$ calc. 312.0961, found 312.0967.

OX04959b

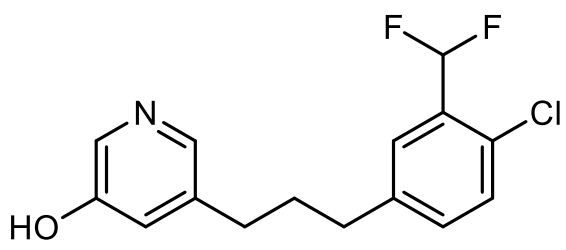

OX04959b

The demethylation using L-Selectride was adapted from a reported route.[1] L-Selectride (0.4 mL, 1 M in THF) was added to **OX04959a** (40 mg, 0.13 mmol) in THF (3 mL) and then the mixture was stirred at 70ºC for 48 h. After completion, the mixture was cooled to rt, quenched with MeOH, stirred for 30 min and concentrated *in vacuo*. The residue was then purified by flash column chromatography (0–8% MeOH in CH_2Cl_2) to afford **OX04959b** as a white solid (40 mg, 92%). ^{1}H NMR (400 MHz, $CDCl_3$) δ 8.12 (d, J = 2.7 Hz, 1H), 7.91 (d, J = 1.8 Hz, 1H), 7.45 (d, J = 2.1 Hz, 1H), 7.32 (dt, J = 8.2, 1.2 Hz, 1H), 7.22–7.18 (m, 1H), 7.14 (t, J = 2.2 Hz, 1H), 6.92 (t, J = 55.0 Hz, 1H), 2.64 (dt, J = 19.9, 7.7 Hz, 4H), 2.00–1.90 (m, 2H). ^{13}C NMR (101 MHz, $CDCl_3$) δ 155.4, 141.2, 139.5, 139.3, 134.1, 132.1 (t, J = 1.8 Hz), 130.0, 129.1, 127.2, 126.8 (t, J = 5.9 Hz), 125.2, 112.2 (t, J = 237.9 Hz), 34.8, 32.3, 32.2. ^{19}F NMR (376 MHz, $CDCl_3$) δ -115.0. HRMS (ESI +ve) $C_{15}H_{14}ClF_2NO$ $[M+H]^+$ calc. 298.0805, found 298.0814.

OX04959

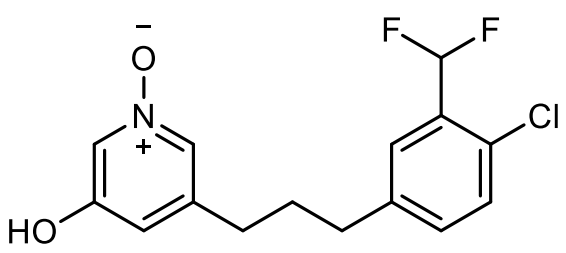

OX04959

To a solution of **OX04959b** (20 mg, 0.07 mmol) in CH_2Cl_2 (10 mL) at rt was added *m*-CPBA (1.5 eq.) and the resulting mixture stirred for 2 h. After completion, the reaction was quenched with $NaHCO_3$ (sat, aq.). The organic phase was washed with brine, dried (Na_2SO_4), filtered and concentrated *in vacuo*. Purification by flash column chromatography (0–6% MeOH in CH_2Cl_2) afforded **OX04959** as a white solid (9 mg, 43%). ^{1}H NMR (400 MHz, $CDCl_3$) δ 8.01 (s, 1H), 7.63 (s, 1H), 7.44 (d, J = 2.2 Hz, 1H), 7.33 (d, J = 8.2 Hz, 1H), 7.19 (dd, J = 8.2, 2.1 Hz, 1H), 7.08–6.77 (m, 2H), 2.65 (t, J = 7.7 Hz, 2H), 2.55 (t, J = 7.7 Hz, 2H), 1.97–1.85 (m, 2H). ^{13}C NMR (151 MHz, CDCl3) δ 157.5, 141.3, 140.7, 132.0, 131.9, 130.5, 130.1, 130.1, 126.8 (t, J = 5.9 Hz), 126.7, 119.8, 112.2 (t, J = 237.1 Hz), 34.6, 32.3, 31.6. ^{19}F NMR (376 MHz, $CDCl_3$) δ -115.0. LRMS (ESI +ve) 314 $[M+H]^+$. HRMS (ESI +ve) $C_{15}H_{14}ClF_2NO_2$ $[M+H]^+$ calc. 314.0754, found 314.0768. HPLC 98% (AUC), t_R = 6.8 min.

OX04960a

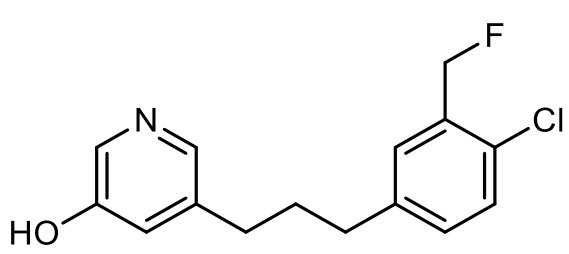

OX04960a

OX04955b (110 mg, 0.4 mmol) was dissolved in anhydrous CH_2Cl_2 (CH_2Cl_2) (4 mL) at room temperature under inert conditions. The solution was then cooled to 0°C and stirred for 5 minutes. Diethylaminosulfur trifluoride (DAST) (0.2 mL, 1.2 mmol) was added dropwise, and the mixture was allowed to warm gradually to room temperature. The reaction was stirred for 24 h. Upon completion, $NaHCO_3$ solution (4 mL) was added, followed by EtOAc. The organic layer was separated, dried over Na_2SO_4, and concentrated. Purification by flash chromatography (0–10% MeOH in CH_2Cl_2) afforded **OX04960a** as a brown solid (12 mg, 11% yield). ^{1}H NMR (400 MHz, $CDCl_3$) δ 8.14 (d, J = 2.7 Hz, 1H), 7.93 (d, J = 1.8 Hz, 1H), 7.28 (dd, J = 8.2, 1.3 Hz, 2H), 7.14–7.11 (m, 1H), 7.09 (dt, J = 7.9, 1.6 Hz, 1H), 5.54 (s, 1H), 5.42 (s, 1H), 2.63 (dt, J = 13.3, 7.7 Hz, 4H), 2.01–1.89 (m, 2H). ^{13}C NMR (101 MHz, $CDCl_3$) δ 154.8, 140.7, 139.7, 139.4, 134.1, 129.7, 129.7, 129.4, 128.6, 128.5, 124.8, 81.7 (d, J = 168.5 Hz), 34.6, 32.2, 32.1. ^{19}F NMR (377 MHz, $CDCl_3$) δ -216.85. HRMS (ESI +ve) $C_{15}H_{15}ClFNO$ [M+H$^+$] calc. 280.0899, found 280.0964.

OX04960

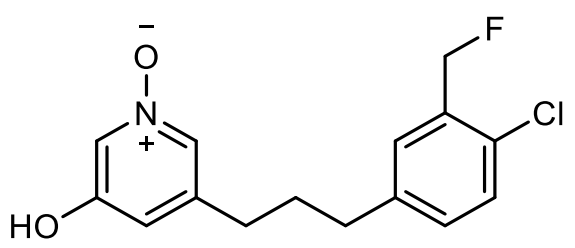

OX04960

To a solution of **OX04960a** (11.7 mg, 0.04 mmol, 1.0 eq.) in CH_2Cl_2 (7 mL), *meta*-chloroperoxybenzoic acid (1.5 eq.) was added at room temperature. The reaction progress was monitored by TLC. After 2 hours, $NaHCO_3$ solution (10 mL) was added. The mixture was diluted with CH_2Cl_2, and the organic layer was separated, washed with brine, and dried over Na_2SO_4. Purification by flash chromatography (0–10% MeOH in CH_2Cl_2) yielded **OX04960** (5 mg, 44% yield) as a light brown solid. ^{1}H NMR (600 MHz, $CDCl_3$) δ 7.99 (s, 1H), 7.63 (s, 1H), 7.29 (d, J = 8.1 Hz, 1H), 7.27 (s, 1H), 7.07 (d, J = 7.5 Hz, 1H), 6.92 (s, 1H), 5.48 (d, J = 47.2 Hz, 2H), 2.58 (dt, J = 54.0, 7.7 Hz, 4H), 1.91 (p, J = 7.7 Hz, 2H). ^{13}C NMR (151 MHz, $CDCl_3$) δ 157.3, 141.3, 140.1, 134.1 (d, J = 17.7 Hz), 129.9 (d, J = 5.3 Hz), 129.7, 129.6 (d, J = 3.0 Hz), 129.4, 128.3 (d, J = 8.7 Hz), 126.4, 119.8, 81.6 (d, J = 168.3 Hz), 34.3, 32.0, 31.4. ^{19}F NMR (377 MHz, CDCl3) δ -217.37. HRMS (ESI +ve) $C_{15}H_{16}ClFNO_2$ [M+H$^+$] calc. 296.0848, found 296.0829. HPLC 95% (AUC), t_R = 7.3 min.

OX04961a

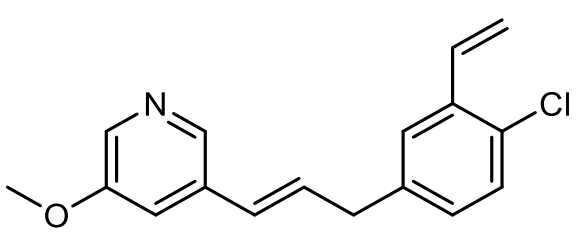

OX04961a

To a nitrogen-degassed microwave vial with **OX04956b** (1150 mg, 3.4 mmol), vinyl boronic ester (787 mg, 5.1 mmol) and Cs_2CO_3 (1111 mg, 10.2 mmol) in THF:H_2O mixture (9:1, 10 mL) was added PPh_3 (89 mg, 0.3 mmol) and $Pd(OAc)_2$ (38 mg, 0.2 mmol). After degassing 5 min with nitrogen, the vial was sealed and the mixture was placed on a pre-heated heating plate and stirred at 70ºC for 25 h and then the mixture was diluted

with EtOAc and filtered through Celite®. The organic layer was washed with brine (30 mL × 2), dried over Na_2SO_4 and concentrated *in vacuo*. **OX04961a** was purified via column chromatography (0–30% EtOAc in petroleum) as a yellow oil (543 mg, 56%). ^{1}H NMR (400 MHz, $CDCl_3$) δ 8.19 (d, *J* = 1.8 Hz, 1H), 8.16 (d, *J* = 2.8 Hz, 1H), 7.42 (d, *J* = 2.2 Hz, 1H), 7.30 (d, *J* = 8.2 Hz, 1H), 7.15 (dd, *J* = 2.8, 1.8 Hz, 1H), 7.13–7.05 (m, 2H), 6.42–6.38 (m, 2H), 5.74 (dd, *J* = 17.6, 1.1 Hz, 1H), 5.38 (dd, *J* = 11.0, 1.1 Hz, 1H), 3.85 (s, 3H), 3.56–3.52 (m, 2H). ^{13}C NMR (101 MHz, $CDCl_3$) δ 155.8, 140.8, 138.3, 136.5, 135.9, 133.6, 133.3, 131.4, 131.3, 129.9, 129.4, 127.9, 126.9, 116.9, 116.8, 55.7, 38.9. LRMS (ESI +ve) 286 $[M+H]^+$. HRMS (ESI +ve) $C_{17}H_{16}ClNO$ $[M+H]^+$ calc. 286.0993, found 286.0971.

OX04961b

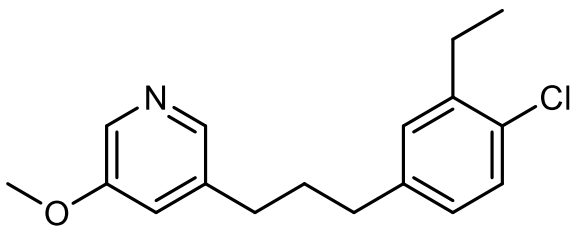

OX04961b

54 mg Pd/C (10% M/w) was added in one portion to a N_2 degassed solution of the **OX04961a** (540 mg, 1.9 mmol) in MeOH (60 mL) at rt, the atmosphere was replaced with H_2, and the reaction stirred for 1 h. After completion, the reaction was filtered through Celite® using MeOH as an eluent, concentrated *in vacuo* and purified by flash column chromatography (30% EtOAc in Pentane) to afford the **OX04961b** as a yellow oil (215 mg, 39%). ^{1}H NMR (400 MHz, $CDCl_3$) δ 8.17 (d, *J* = 2.8 Hz, 1H), 8.09 (d, *J* = 1.8 Hz, 1H), 7.30–7.23 (m, 1H), 7.06–7.01 (m, 2H), 6.96 (dd, *J* = 8.1, 2.3 Hz, 1H), 3.87 (s, 3H), 2.74 (q, *J* = 7.5 Hz, 2H), 2.64 (q, *J* = 7.7 Hz, 4H), 2.01–1.90 (m, 2H), 1.24 (t, *J* = 7.5 Hz, 3H). ^{13}C NMR (101 MHz, $CDCl_3$) δ 155.8, 142.2, 141.6, 140.5, 138.3, 134.6, 131.4, 129.8, 129.4, 127.2, 121.2, 55.7, 34.8, 32.6, 32.4, 26.9, 14.3. LRMS (ESI +ve) 290 $[M+H]^+$. HRMS (ESI +ve) $C_{17}H_{20}ClNO$ $[M+H]^+$ calc. 290.1306, found 290.1323.

OX04961c

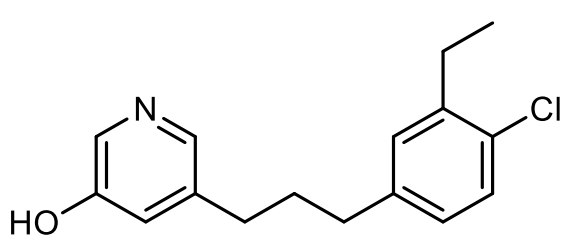

OX04961c

5 mL 48% HBr in H_2O was added to **OX04961b** (215 mg, 0.7 mmol) and then the mixture was stirred at 120ºC for 48 h. After completion of the reaction, cooled to rt, the mixture was neutralized with sat. aq. $NaHCO_3$ and extracted with EtOAc (3 × 40 mL). The combined organic extracts were washed with brine (100 mL), dried over Na_2SO_4, filtered, and concentrated under reduced pressure. The resulting residue was purified by flash column chromatography (0–6% MeOH in CH_2Cl_2) afforded **OX04961c** as a yellow oil (113 mg, 55%). ^{1}H NMR (400 MHz, $CDCl_3$) δ 11.27 (s, 1H), 8.10 (d, *J* = 2.6 Hz, 1H), 7.90 (d, *J* = 1.8 Hz, 1H), 7.22 (d, *J* = 8.1 Hz, 1H), 7.14 (t, *J* = 2.2 Hz, 1H), 7.01 (d, *J* = 2.2 Hz, 1H), 6.91 (dd, *J* = 8.1, 2.2 Hz, 1H), 2.71 (q, *J* = 7.5 Hz, 2H), 2.59 (td, *J* = 7.8, 2.4 Hz, 4H), 1.97–1.86 (m, 2H), 1.21 (t, *J* = 7.5 Hz, 3H). ^{13}C NMR (101

MHz, $CDCl_3$) δ 155.5, 141.6, 140.4, 139.9, 139.1, 133.8, 131.4, 129.7, 129.4, 127.2, 125.3, 34.8, 32.4, 32.3, 26.9, 14.3. LRMS (ESI +ve) 276 $[M+H]^+$. HRMS (ESI +ve) $C_{16}H_{18}ClNO$ $[M+H^+]$ calc. 276.1150, found 276.1124.

OX04961

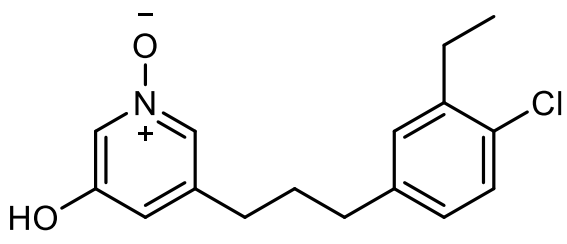

OX04961

3-(3-(4-chloro-3-ethylphenyl)propyl)-5-hydroxypyridine 1-oxide

To a solution of **OX04961c** (64 mg, 0.2 mmol) in CH_2Cl_2 (20 mL) at rt was added *m*-CPBA (1.5 eq.) and the resulting mixture stirred for 2 h. After completion, the reaction was quenched with $NaHCO_3$ (sat., aq.). The organic phase was washed with brine, dried (Na_2SO_4), filtered and concentrated *in vacuo*. Purification by flash column chromatography (0–6% MeOH in CH_2Cl_2) afforded **OX04961** as a yellow oil (28 mg, 41%). ^1H NMR (600 MHz, $CDCl_3$) δ 7.99 (s, 1H), 7.63 (s, 1H), 7.23 (d, J = 8.1 Hz, 1H), 7.00 (d, J = 2.2 Hz, 1H), 6.93–6.88 (m, 2H), 2.72 (q, J = 7.5 Hz, 2H), 2.58 (t, J = 7.6 Hz, 2H), 2.53 (t, J = 7.7 Hz, 2H), 1.89 (h, J = 6.9 Hz, 2H), 1.22 (t, J = 7.5 Hz, 3H). ^{13}C NMR (151 MHz, $CDCl_3$) δ 157.5, 141.8, 141.6, 139.9, 131.6, 130.0, 129.7, 129.5, 127.2, 126.6, 119.9, 34.6, 32.3, 31.8, 26.9, 14.3. LRMS (ESI +ve) 292 $[M+H]^+$. HRMS (ESI +ve) $C_{16}H_{18}ClNO_2$ $[M+H]^+$ calc. 292.1099, found 292.1100. HPLC 96% (AUC), t_R = 3.0 min.

OX04962a

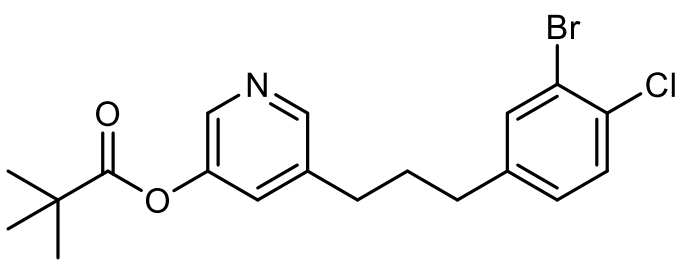

OX04962a

To a slurry of **OX04956d** (2.2 g, 6.8 mmol) and NEt_3 (1.43 mL, 10.2 mmol) in CH_2Cl_2 (80 mL) was added trimethylacetyl chloride (1.09 mL, 8.9 mmol) dropwise at 0ºC over 10 min. The mixture was then stirred at rt for 3 h before poured into H_2O (100 mL). The organic layer was separated, dried (Na_2SO_4) and concentrated *in vacuo* to afford **OX04962a** as an orange oil (2.7 g, 98%). ^1H NMR (400 MHz, $CDCl_3$) δ 8.32–8.28 (m, 1H), 8.23 (d, J = 2.4 Hz, 1H), 7.43 (d, J = 2.1 Hz, 1H), 7.35 (d, J = 8.2 Hz, 1H), 7.26–7.23 (m, 1H), 7.04 (dd, J = 8.2, 2.1 Hz, 1H), 2.63 (dt, J = 17.2, 7.7 Hz, 4H), 2.01–1.89 (m, 2H), 1.37 (s, 9H). ^{13}C NMR (101 MHz, $CDCl_3$) δ 176.8, 147.8, 147.0, 142.0, 141.1, 137.9, 133.7, 132.0, 130.3, 129.1, 128.7, 122.4, 39.3, 34.3, 32.2, 32.0, 27.2. LRMS (ESI +ve) 410 $[M+H]^+$. HRMS (ESI +ve) $C_{19}H_{21}BrClNO_2$ $[M+H]^+$ calc. 410.0517, found 410.0513.

OX04962b

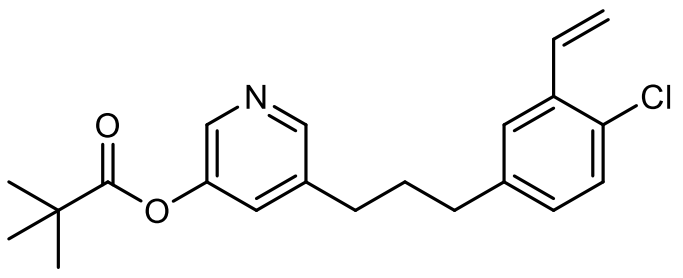

OX04962b

To a degassed microwave vial with **OX04962a** (701 mg, 1.7 mmol) and tributyl(vinyl)stannane (599 mg, 1.9 mmol) in DMF (4 mL) was added $Pd(PPh_3)4$ (109 mg, 0.1 mmol). The mixture was degassed for 5 min before the vial was sealed and placed on a pre-heated heating plate. The mixture was stirred for 24 h at 90ºC. After completion, the mixture was diluted with EtOAc (5 mL), poured into KF (5 mL, sat., aq.) then the mixture was stirred for 30 min before filtered through Celite®. The organic layer was washed with NH_4Cl (20 mL × 2, sat., aq.), dried (Na_2SO_4) and concentrated *in vacuo*. The crude product was purified with flash column chromatography (0–17% EtOAc in petroleum ether) to afford **OX04962b** as a colourless oil (207 mg, 34%). ^{1}H NMR (400 MHz, $CDCl_3$) δ 8.31 (d, *J* = 1.9 Hz, 1H), 8.24 (d, *J* = 2.5 Hz, 1H), 7.36 (d, *J* = 2.2 Hz, 1H), 7.29–7.24 (m, 2H), 7.09 (dd, *J* = 17.5, 11.0 Hz, 1H), 7.02 (dd, *J* = 8.2, 2.2 Hz, 1H), 5.74 (dd, *J* = 17.5, 1.1 Hz, 1H), 5.37 (dd, *J* = 11.0, 1.1 Hz, 1H), 2.66 (dt, *J* = 10.9, 7.6 Hz, 4H), 2.03–1.92 (m, 2H), 1.37 (s, 9H). ^{13}C NMR (101 MHz, $CDCl_3$) δ 176.8, 147.7, 147.0, 141.0, 140.4, 138.1, 135.6, 133.4, 130.9, 129.7, 129.1, 129.1, 126.6, 116.5, 39.3, 34.8, 32.4, 32.1, 27.2. LRMS (ESI +ve) 358 $[M+H]^+$. HRMS (ESI +ve) $C_{21}H_{24}ClNO_2$ $[M+H]^+$ calc. 358.1568, found 358.1576.

OX04962

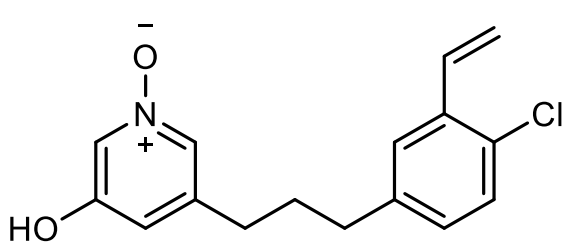

OX04962

To a mixture of **OX04962b** (30 mg, 0.1 mmol) in CH_2Cl_2 (10 mL) was added *m*-CPBA (1.5 eq.) in one portion at rt. The mixture was stirred at rt for 2 h then concentrated *in vacuo*, followed by purification with flash column chromatography (20–100% EtOAc in petroleum ether then 0–20% MeOH in EtOAc). The MeOH in EtOAc fractions were checked by thin layer chromatography, collected, concentrated *in vacuo* and dissolved in MeOH (10 mL). 4M aq. NaOH (2 mL) was added subsequently, and the mixture was stirred for 16 h at rt. After completion, the mixture was concentrated *in vacuo*, taken up with sat. aq. NH_4Cl to adjust pH < 8 and extracted with CH_2Cl_2 (3 × 20 mL). The organic layer was washed with brine, dried with anhydrous Na_2SO_4, concentrated *in vacuo* and purified with flash column chromatography (0–10% MeOH in CH_2Cl_2) to afford **OX04962** as a brown solid (8.3 mg, 34%). ^{1}H NMR (400 MHz, $CDCl_3$) δ 8.00 (s, 1H), 7.63 (s, 1H), 7.33 (d, *J* = 2.1 Hz, 1H), 7.27–7.25 (m, 2H), 7.07 (dd, *J* = 17.5, 11.0 Hz, 1H), 6.97 (dd, *J* = 8.2, 2.2 Hz, 1H), 6.91 (s, 1H), 5.73 (dd, *J* = 17.6, 1.1 Hz, 1H), 5.37 (dd, *J* = 11.0, 1.1 Hz, 1H), 2.60 (t, *J* = 7.6 Hz, 2H), 2.54 (t, *J* = 7.8 Hz, 2H), 1.95–1.85 (m, 2H). ^{13}C NMR (151 MHz, $CDCl_3$) δ 157.6, 141.5, 139.9, 135.8, 133.3, 131.0, 129.9, 129.8, 129.0, 126.7, 126.6, 119.9, 116.7, 34.6, 32.3, 31.7. LRMS (ESI +ve) 290 $[M+H]^+$. HRMS (ESI +ve) $C_{16}H_{16}ClNO_2$ $[M+H]^+$ calc. 290.0942, found 290.0947. HPLC 95% (AUC), t_R = 8.0 min.

OX04963a

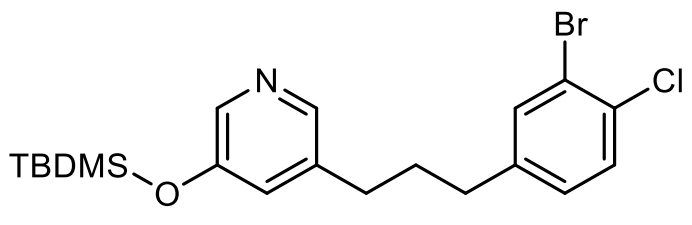

OX04963a

To a solution of **OX04956d** (4.8 g, 14.8 mmol) in chloroform (60 mL) was added TBDMSCl (3.3 g, 22.2 mmol) and imidazole (4.1 g, 59.1 mmol). The organic mixture was stirred at 60ºC for 16 h before washed with H_2O (50 mL × 2), brine (50 mL × 1), dried (Na_2SO_4), concentrated *in vacuo*. Purification with flash column chromatography (0–30% EtOAc in petroleum ether) afforded **OX04963a** as a yellow oil (1.7 g, 26%). ^{1}H NMR (400 MHz, $CDCl_3$) δ 8.07 (d, *J* = 2.7 Hz, 1H), 8.05 (d, *J* = 1.8 Hz, 1H), 7.43 (d, *J* = 2.0 Hz, 1H), 7.35 (d, *J* = 8.2 Hz, 1H), 7.04 (dd, *J* = 8.2, 2.1 Hz, 1H), 6.94 (dd, *J* = 2.6, 1.9 Hz, 1H), 2.59 (t, *J* = 7.7 Hz, 4H), 1.96–1.86 (m, 2H), 0.99 (s, 9H), 0.21 (s, 6H). ^{13}C NMR (101 MHz, $CDCl_3$) δ 157.6, 144.1, 141.6, 139.8, 133.5, 131.4, 129.9, 129.7, 127.5, 126.6, 119.9, 34.6, 32.4, 31.8, 25.2, 20.9, 14.3. LRMS (ESI +ve) 440 $[M+H]^+$. HRMS (ESI +ve) $C_{20}H_{27}BrClNOSi$ $[M+H]^+$ calc. 440.0807, found 440.0815.

OX04963b

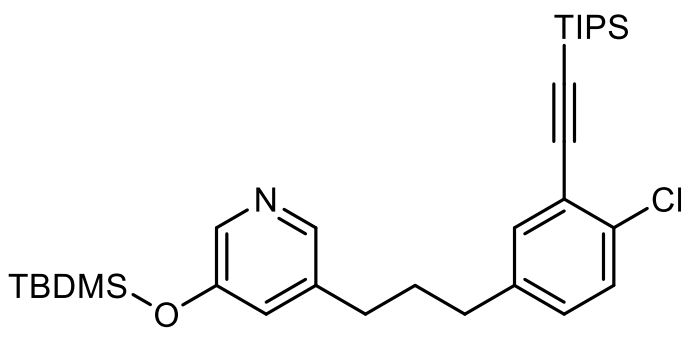

OX04963b

To a microwave vial was added **OX04963a** (773 mg, 1.8 mmol), PPh_3 (92 mg, 0.4 mmol) and ethynyltriisopropylsilane (352 mg, 1.9 mmol) sequentially before the addition of degassed THF/NEt_3- mixture (3:1, 8 mL). The mixture was degassed for 5 min before adding $Pd_2(PPh_3)_2Cl_2$ (123 mg, 0.2 mmol) and CuI (134 mg, 0.7 mmol). The mixture was degassed for another 5 min then the vial was sealed, placed on a 60°C pre-heated heating plate and stirred for 16 h. After completion, the mixture was diluted with EtOAc (40 mL), filtered through Celite® and concentrated *in vacuo*. The dried mixture was taken up with CH_2Cl_2 (50 mL), washed with brine (50 mL × 2), dried (Na_2SO_4) and concentrated *in vacuo*. The residue was then purified by flash column chromatography (0–100% CH_2Cl_2 in petroleum ether then 0–10% EtOAc in CH_2Cl_2) to afford **OX04963b** as a transparent oil (660 mg, 69%). ^{1}H NMR (400 MHz, $CDCl_3$) δ 8.07 (d, *J* = 2.7 Hz, 2H), 7.31 (d, *J* = 2.2 Hz, 1H), 7.29 (d, *J* = 8.3 Hz, 1H), 7.03 (dd, *J* = 8.2, 2.2 Hz, 1H), 6.95 (dd, *J* = 2.6, 1.9 Hz, 1H), 2.63–2.55 (m, 4H), 1.96–1.86 (m, 2H), 1.15 (s, 21H), 0.99 (s, 9H), 0.21 (s, 6H). ^{13}C NMR (101 MHz, $CDCl_3$) δ 152.2, 143.0, 140.3, 140.3, 138.0, 134.0, 133.6, 129.6, 129.3, 127.0, 123.4, 103.4, 96.6, 34.6, 32.5, 32.2, 25.8, 18.8, 18.4, 11.4, -4.3. LRMS (ESI +ve) 542 $[M+H]^+$. HRMS (ESI +ve) $C_{31}H_{48}ClNOSi_2$ $[M+H]^+$ calc. 542.3036, found 542.3040.

OX04963

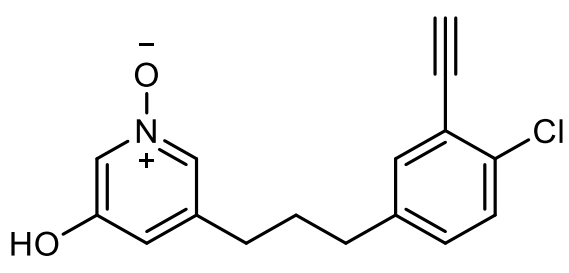

OX04963

To a solution of **OX04963b** (60 mg, 0.1 mmol) in CH_2Cl_2 (10 mL) was added *m*-CPBA (60 mg, 0.27 mmol) in one portion at rt. The mixture was stirred for 2 h before adding $NaHCO_3$ (20 mL, sat., aq.) to quench the reaction. The organic layer was separated and washed with $NaHCO_3$ (25 mL × 2), brine (25 mL), dried (Na_2SO_4) and concentrated *in vacuo*. The resulting while solid was dissolved in THF (10 mL) and TBAF (0.3 mL, 1M in THF) was added at 0ºC. The mixture was stirred for 1 h and MeOH (10 mL) was added, then the mixture was concentrated *in vacuo*. Flash column chromatography (20–100% EtOAc in petroleum ether, then 0–15% MeOH in EtOAc) afforded **OX04963** as a yellow oil (7.0 mg, 22%). ^{1}H NMR (400 MHz, $CDCl_3$) δ 7.93 (t, *J* = 1.9 Hz, 1H), 7.56 (d, *J* = 1.5 Hz, 1H), 7.26 (s, 1H), 7.22 (d, *J* = 14.1 Hz, 1H), 7.00 (dd, *J* = 8.3, 2.2 Hz, 1H), 6.85 (d, *J* = 1.6 Hz, 1H), 3.29 (s, 1H), 2.49 (dt, *J* = 20.6, 7.6 Hz, 4H), 1.82 (tt, *J* = 9.2, 6.7 Hz, 2H). ^{13}C NMR (101 MHz, $CDCl_3$) δ 157.6, 141.3, 139.8, 134.1, 133.9, 130.2, 129.8, 129.5, 126.7, 122.1, 119.8, 82.5, 80.4, 34.3, 32.2, 31.5. LRMS (ESI +ve) 288 $[M+H]^+$. HRMS (ESI +ve) $C_{16}H_{14}ClNO_2$ $[M+H]^+$ calc. 288.0786, found 288.0800. HPLC 99% (AUC), t_R = 6.6 min.

OX04964a

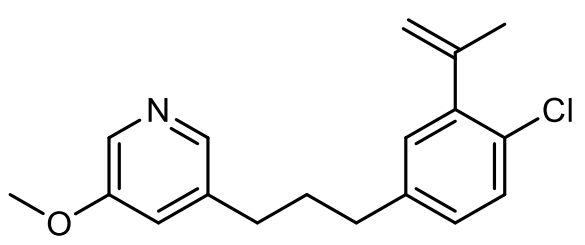

OX04964a

To a degassed microwave vial with **OX04956c** (339 mg, 1.45 mmol) and tributyl(prop-1-en-2-yl)stannane (660 mg, 2.0 mmol) in DMF (4 mL) was added $Pd(PPh_3)_4$ (173 mg, 0.15 mmol). The mixture was degassed for 5 min before the vial was sealed and placed on a pre-heated heating plate. The mixture was stirred for 24 h at 90ºC. After completion, the mixture was diluted with EtOAc (5 mL), poured into KF (5 mL, sat., aq.) then the mixture was stirred for 30 min before filtered through Celite®. The organic layer was washed with NH_4Cl (20 mL × 2, sat., aq.), dried (Na_2SO_4) and concentrated *in vacuo*. The crude product was purified with flash column chromatography (0–30% EtOAc in petroleum ether) to afford **OX04964a** as a colourless oil (170 mg, 39%). ^{1}H NMR (400 MHz, $CDCl_3$) δ 8.15 (d, *J* = 2.8 Hz, 1H), 8.07 (d, *J* = 1.8 Hz, 1H), 7.30–7.22 (m, 1H), 7.00 (td, *J* = 3.0, 1.2 Hz, 3H), 5.22 (p, *J* = 1.6 Hz, 1H), 4.96 (dq, *J* = 1.9, 1.0 Hz, 1H), 3.85 (s, 3H), 2.62 (q, *J* = 7.2 Hz, 4H), 2.10 (dd, *J* = 1.5, 0.9 Hz, 3H), 1.98–1.91 (m, 2H). ^{13}C NMR (101 MHz, $CDCl_3$) δ 155.7, 144.6, 142.7, 142.5, 140.5, 138.0, 135.0, 129.9, 129.6, 129.4, 128.3, 120.8, 116.2, 55.6, 34.7, 32.5, 32.4, 23.5. LRMS (ESI +ve) 302 $[M+H]^+$. HRMS (ESI +ve) $C_{18}H_{20}ClNO$ $[M+H]^+$ calc. 302.1306, found 302.1311.

OX04964b

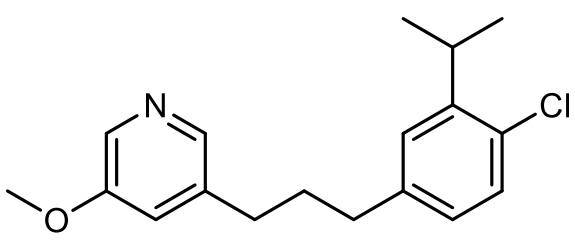

OX04964b

Into Seven a N_2 degassed solution of the **OX04964a** (130 mg, 0.43 mmol) in MeOH (180 mL), 7 mg PtO_2 was added in one portion to at rt, the atmosphere was then replaced with H_2, and the reaction stirred for 1 h. After completion, the reaction was filtered through Celite® using MeOH as an eluent, concentrated *in vacuo* and purified by flash column chromatography (30% EtOAc in Pentane) to afford **OX04964b** as a colourless oil (84 mg, 64%). ^{1}H NMR (400 MHz, $CDCl_3$) δ 8.15 (d, *J* = 2.8 Hz, 1H), 8.07 (d, *J* = 1.8 Hz, 1H), 7.24 (d, *J* = 8.1 Hz, 1H), 7.07 (d, *J* = 2.2 Hz, 1H), 7.03–6.99 (m, 1H), 6.92 (dd, *J* = 8.1, 2.2 Hz, 1H), 3.85 (s, 3H), 3.38 (p, *J* = 6.9 Hz, 1H), 2.62 (td, *J* = 7.7, 3.8 Hz, 4H), 2.00–1.88 (m, 2H), 1.23 (d, *J* = 6.9 Hz, 6H). ^{13}C NMR (101 MHz, $CDCl_3$) δ 155.8, 145.6, 142.3, 140.6, 138.2, 134.7, 131.0, 129.4, 127.0, 126.7, 121.0, 55.6, 35.0, 32.6, 32.4, 30.2, 22.8. LRMS (ESI +ve) 304 $[M+H]^+$. HRMS (ESI +ve) $C_{18}H_{22}ClNO$ $[M+H]^+$ calc. 304.1463, found 304.1467.

OX04964c

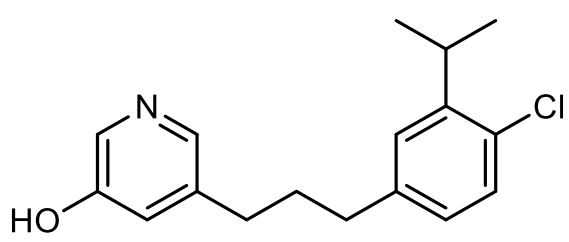

OX04964c

Into **OX04964b** (80 mg, 0.26 mmol) was added 3 mL 48% HBr in H_2O and then the mixture was stirred at 120ºC for 48 h. After completion of the reaction, cooled to rt, the mixture was neutralized with sat. aq. $NaHCO_3$ and extracted with EtOAc (3 × 30 mL). The combined organic extracts were washed with brine (100 mL), dried over Na_2SO_4, filtered, and concentrated under reduced pressure. The resulting residue was purified by flash column chromatography (5% MeOH in CH_2Cl_2) afforded **OX04964c** as a brown solid (46 mg, 60%). ^{1}H NMR (400 MHz, $CDCl_3$) δ 8.14 (d, *J* = 2.7 Hz, 1H), 7.92 (d, *J* = 1.8 Hz, 1H), 7.23 (d, *J* = 8.1 Hz, 1H), 7.16–7.13 (m, 1H), 7.06 (d, *J* = 2.2 Hz, 1H), 6.91 (dd, *J* = 8.1, 2.2 Hz, 1H), 3.37 (hept, *J* = 6.7 Hz, 1H), 2.66–2.57 (m, 4H), 1.99–1.87 (m, 2H), 1.23 (d, *J* = 6.9 Hz, 6H). ^{13}C NMR (101 MHz, $CDCl_3$) δ 155.2, 145.7, 140.5, 139.8, 139.6, 134.0, 131.0, 129.5, 127.0, 126.8, 125.2, 35.0, 32.4, 32.3, 30.2, 22.8. LRMS (ESI +ve) 290 $[M+H]^+$. HRMS (ESI +ve) $C_{17}H_{20}ClNO$ $[M+H]^+$ calc. 290.1306, found 290.1313.

OX04964

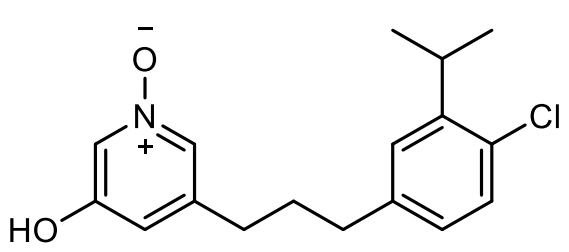

OX04964

To a solution of **OX04964c** (30 mg, 0.1 mmol) in CH_2Cl_2 (10 mL) at rt was added *m*-CPBA (1.5 eq.) and the resulting mixture stirred for 2 h. After completion, the reaction was quenched with $NaHCO_3$ (sat., aq.). The organic phase was washed with brine, dried (Na_2SO_4), filtered and concentrated *in vacuo*. Purification by flash column chromatography (0–8% MeOH in CH_2Cl_2) afforded **OX04964** as a yellow oil (19 mg, 60%). ^{1}H NMR (400 MHz, $CDCl_3$) δ 8.00 (s, 1H), 7.63 (s, 1H), 7.24 (d, *J* = 8.1 Hz, 1H), 7.05 (d, *J* = 2.2 Hz, 1H), 6.93–6.87 (m, 2H), 3.37 (p, *J* = 6.8 Hz, 1H), 2.56 (dt, *J* = 23.1, 7.7 Hz, 4H), 1.90 (h, *J* = 6.8 Hz, 2H), 1.23 (d, *J* = 6.9 Hz, 6H). ^{13}C NMR (151 MHz, $CDCl_3$) δ 157.5, 145.8, 141.6, 140.0, 131.3, 130.0, 129.6, 126.9, 126.7, 126.6, 119.9, 34.8, 32.3, 31.8, 30.2, 22.8. LRMS (ESI +ve) 306 $[M+H]^+$. HRMS (ESI +ve) $C_{17}H_{20}ClNO_2$ $[M+H^+]$ calc. 306.1255, found 306.1267. HPLC 99% (AUC), t_R = 6.5 min.

OX04965a

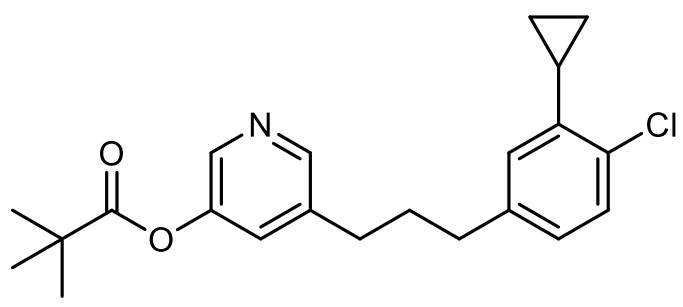

OX04965a

To dichloromethane (4 mL), it was added Et_2Zn (1.3 mL, 1.0 M in hexane). The solution was cooled in an ice bath and TFA (140 mg, 1.2 mmol) was then added dropwise into the reaction mixture. Upon stirring for 30 min, a solution of CH_2I_2 (330 mg, 1.2 mmol) was added at 0ºC. After an additional 20 min of stirring, a solution of **OX04962b** (44 mg, 0.1 mmol) in CH_2Cl_2 (4 mL) was added, and the ice bath was removed. The reaction mixture was stirred for 16 h before it was quenched with $NaHCO_3$ (10 mL, sat., aq.) and the phases were separated. The organic layer was washed with NH_4Cl (20 mL × 2, sat., aq.), brine (15 mL) and then dried (Na_2SO_4), filtered and concentrated *in vacuo*. The residue was purified by flash column chromatography (10–18% EtOAc in petroleum ether) to afford **OX04965a** as a beige oil (32 mg, 92%). ^{1}H NMR (400 MHz, $CDCl_3$) δ 8.30 (d, *J* = 1.8 Hz, 1H), 8.23 (d, *J* = 2.5 Hz, 1H), 7.27–7.20 (m, 2H), 6.91 (dd, *J* = 8.1, 2.2 Hz, 1H), 6.72 (d, *J* = 2.2 Hz, 1H), 2.64 (t, *J* = 7.7 Hz, 2H), 2.58 (t, *J* = 7.7 Hz, 2H), 2.22–2.12 (m, 1H), 1.98–1.87 (m, 2H), 1.37 (s, 9H), 1.03–0.96 (m, 2H), 0.70–0.63 (m, 2H). ^{13}C NMR (101 MHz, $CDCl_3$) δ 176.8, 147.7, 147.1, 141.0, 140.8, 140.2, 138.2, 132.9, 129.1, 129.1, 126.8, 126.3, 39.3, 34.9, 32.4, 32.1, 27.2, 13.4, 8.1. LRMS (ESI +ve) 372 $[M+H]^+$. HRMS (ESI +ve) $C_{22}H_{26}ClNO_2$ $[M+H]^+$ calc. 372.1725, found 372.1713.

OX04965

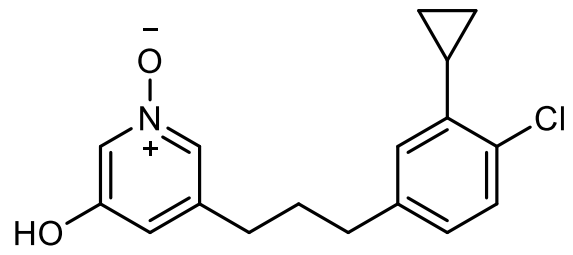

OX04965

To a mixture of **OX04965a** (20 mg, 0.05 mmol) in CH_2Cl_2 (10 mL) was added *m*-CPBA (1.5 eq.) in one portion at rt. The mixture was stirred at rt for 2 h then concentrated *in vacuo*, followed by purification with

flash column chromatography (20–100% EtOAc in petroleum ether then 0–20% MeOH in EtOAc). The MeOH in EtOAc fractions were checked by thin layer chromatography, collected, concentrated *in vacuo* and dissolved in MeOH (10 mL). 4M aq. NaOH (2 mL) was added subsequently, and the mixture was stirred for 16 h at rt. After completion, the mixture was concentrated *in vacuo*, taken up with sat. aq. NH_4Cl to adjust pH < 8 and extracted with CH_2Cl_2 (3 × 20 mL). The organic layer was washed with brine, dried with anhydrous Na_2SO_4, concentrated *in vacuo* and purified with flash column chromatography (0–10% MeOH in CH_2Cl_2) to afford **OX04965** as a white solid (14 mg, 86%). ^{1}H NMR (400 MHz, $CDCl_3$) δ 8.01 (t, *J* = 1.9 Hz, 1H), 7.63 (d, *J* = 1.5 Hz, 1H), 7.24 (d, *J* = 8.1 Hz, 1H), 6.93–6.83 (m, 2H), 6.69 (d, *J* = 2.1 Hz, 1H), 2.52 (dt, *J* = 14.7, 7.7 Hz, 4H), 2.16 (tt, *J* = 8.5, 5.3 Hz, 1H), 1.85 (tt, *J* = 9.3, 6.7 Hz, 2H), 1.05–0.93 (m, 2H), 0.72–0.62 (m, 2H). ^{13}C NMR (101 MHz, $CDCl_3$) δ 157.5, 141.6, 141.0, 139.8, 133.1, 129.9, 129.2, 126.7, 126.6, 126.3, 119.9, 34.7, 32.3, 31.8, 13.4, 8.1. LRMS (ESI +ve) 304 $[M+H]^+$. HRMS (ESI +ve) $C_{17}H_{18}ClNO_2$ $[M+H]^+$ calc. 304.1099, found 304.1097. HPLC 95% (AUC), t_R = 8.0 min.

OX04966a

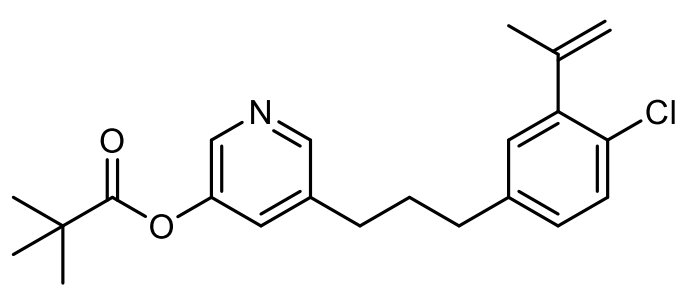

OX04966a

To an N_2 degassed mixture of **OX04962a** (839 mg, 2.05 mmol) and tributyl(1-methylethenyl)Stannane (1362 mg, 4.10 mmol) in DMF (4.5 mL) was added $Pd(PPh_3)_4$ (118 mg, 0.10 mmol). The mixture was subsequently degassed for another 5 min before the mixture was heated to 90ºC and stirred for 72 h. After completion, the mixture was poured into sat. aq. KF (10 mL), stirred for 30 min and EtOAc (2 × 30 mL) was added to extract the organic layer. The organic layer was then washed with sat. aq. KF (1 × 10 mL), water (3 × 30 mL), brine (1 × 50 mL), dried over Na_2SO_4 and saturated *in vacuo*. The resulting brown oil was purified with flash column chromatography (0–30% EtOAc in petroleum ether) to afford **OX04966a** as a yellow oil (677 mg, 89%). ^{1}H NMR (400 MHz, $CDCl_3$) δ 8.30 (s, 1H), 8.22 (s, 1H), 7.26–7.23 (m, 2H), 7.02–6.98 (m, 2H), 5.21 (p, *J* = 1.6 Hz, 1H), 4.95 (dq, *J* = 1.9, 0.9 Hz, 1H), 2.64 (dt, *J* = 17.2, 7.7 Hz, 4H), 2.09 (dd, *J* = 1.5, 0.9 Hz, 3H), 2.01–1.90 (m, 2H), 1.36 (s, 9H). ^{13}C NMR (101 MHz, $CDCl_3$) δ 176.8, 147.1, 144.6, 142.8, 141.0, 140.3, 129.9, 129.6, 129.4, 129.1, 128.3, 116.2, 39.3, 34.7, 32.4, 32.2, 27.2, 23.5. HRMS (ESI +ve) $C_{22}H_{26}ClNO_2$ $[M+H^+]$ calc. 372.1725, found 372.1742.

OX04966b

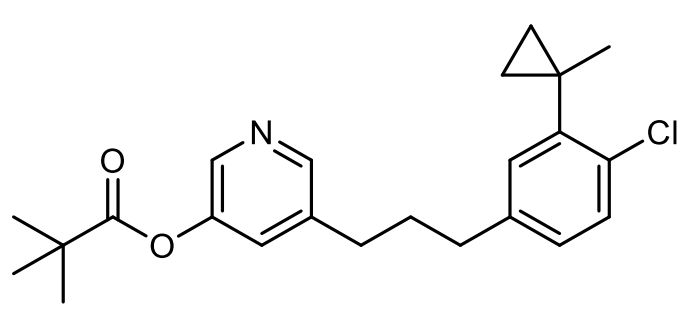

OX04966b

To a mixture of $ZnEt_2$ (8.1 mL, 1M in hexane) in CH_2Cl_2 (18 mL) was added TFA (0.62 mL) dropwise at 0ºC and the resulting white slurry was stirred vigorously for 30 min at 0ºC. Diiodomethane (0.65 mL) was added dropwise to the mixture at 0ºC to from a Clear solution, followed by stirring for 30 min. Then **OX04966a** (300 mg, 0.8 mmol) in CH_2Cl_2 (5 mL) was added to the mixture at 0ºC, the ice bath was removed, and the reaction mixture was stirred for 16 h. After completion, the mixture was poured into sat. $NaHCO_3$ (50 mL) and separated. The organic layer was washed with sat. aq. NH_4Cl (2 × 50 mL), brine (1 × 30 mL) and concentrated *in vacuo*. Purification with flash column chromatography (0–20% EtOAc in petroleum ether) afforded **OX04966b** as a colorless oil (98 mg, 31%). ^{1}H NMR (400 MHz, $CDCl_3$) δ 8.23 (d, J = 1.9 Hz, 1H), 8.15 (d, J = 2.5 Hz, 1H), 7.18 (d, J = 2.4 Hz, 1H), 7.14 (d, J = 8.1 Hz, 1H), 7.07 (d, J = 2.2 Hz, 1H), 6.86 (dd, J = 8.1, 2.3 Hz, 1H), 2.55 (dt, J = 20.9, 7.7 Hz, 4H), 1.92–1.83 (m, 2H), 1.29 (s, 9H), 1.26 (s, 3H), 0.76–0.70 (m, 2H), 0.70–0.63 (m, 2H). ^{13}C NMR (101 MHz, $CDCl_3$) δ 176.8, 147.7, 147.0, 143.9, 140.9, 140.2, 138.2, 133.2, 131.5, 129.6, 129.0, 127.6, 39.3, 34.7, 32.4, 32.2, 27.2, 25.2, 20.9, 14.2. HRMS (ESI +ve) $C_{23}H_{28}ClNO_2$ $[M+H]^+$ calc. 386.1881, found 386.1906.

OX04966

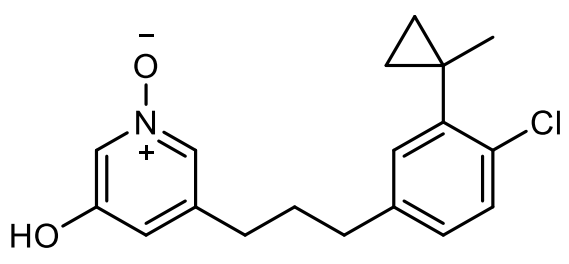

OX04966

To a mixture of **OX04966b** (98 mg, 0.3 mmol) in CH_2Cl_2 (30 mL) was added *m*-CPBA (1.5 eq.) in one portion at rt. The mixture was stirred at rt for 2 h then concentrated *in vacuo*, followed by purification with flash column chromatography (20–100% EtOAc in petroleum ether then 0–20% MeOH in EtOAc). The MeOH in EtOAc fractions were checked by thin layer chromatography, collected, concentrated *in vacuo* and dissolved in MeOH (10 mL). 4M aq. NaOH (5 mL) was added subsequently, and the mixture was stirred for 16 h at rt. After completion, the mixture was concentrated *in vacuo*, taken up with sat. aq. NH_4Cl to adjust pH < 8 and extracted with CH_2Cl_2 (3 × 20 mL). The organic layer was washed with brine, dried with anhydrous Na_2SO_4, concentrated *in vacuo* and purified with flash column chromatography (0–10% MeOH in CH_2Cl_2) to afford **OX04966** as a white solid (80 mg, 99%). ^{1}H NMR (400 MHz, $CDCl_3$) δ 8.01 (s, 1H), 7.63 (s, 1H), 7.22 (d, J = 8.1 Hz, 1H), 7.13 (d, J = 2.3 Hz, 1H), 6.94–6.89 (m, 2H), 2.55 (dt, J = 15.1, 7.7 Hz, 4H), 1.95–1.84 (m, 2H), 1.33 (s, 3H), 0.83–0.77 (m, 2H), 0.77–0.71 (m, 2H). ^{13}C NMR (101 MHz, $CDCl_3$) δ 157.6, 144.1, 141.6, 139.8, 133.5, 131.4, 129.9, 129.7, 127.5, 126.6, 119.9, 34.6, 32.4, 31.8, 25.2, 20.9, 14.3. LRMS (ESI +ve) 318 $[M+H]^+$. HRMS (ESI +ve) $C_{18}H_{20}ClNO_2$ $[M+H]^+$ calc. 318.1255, found 318.1253. HPLC 99% (AUC), t_R = 8.6 min.

NMR

OX04539a

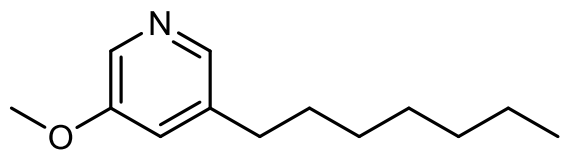

OX04539a

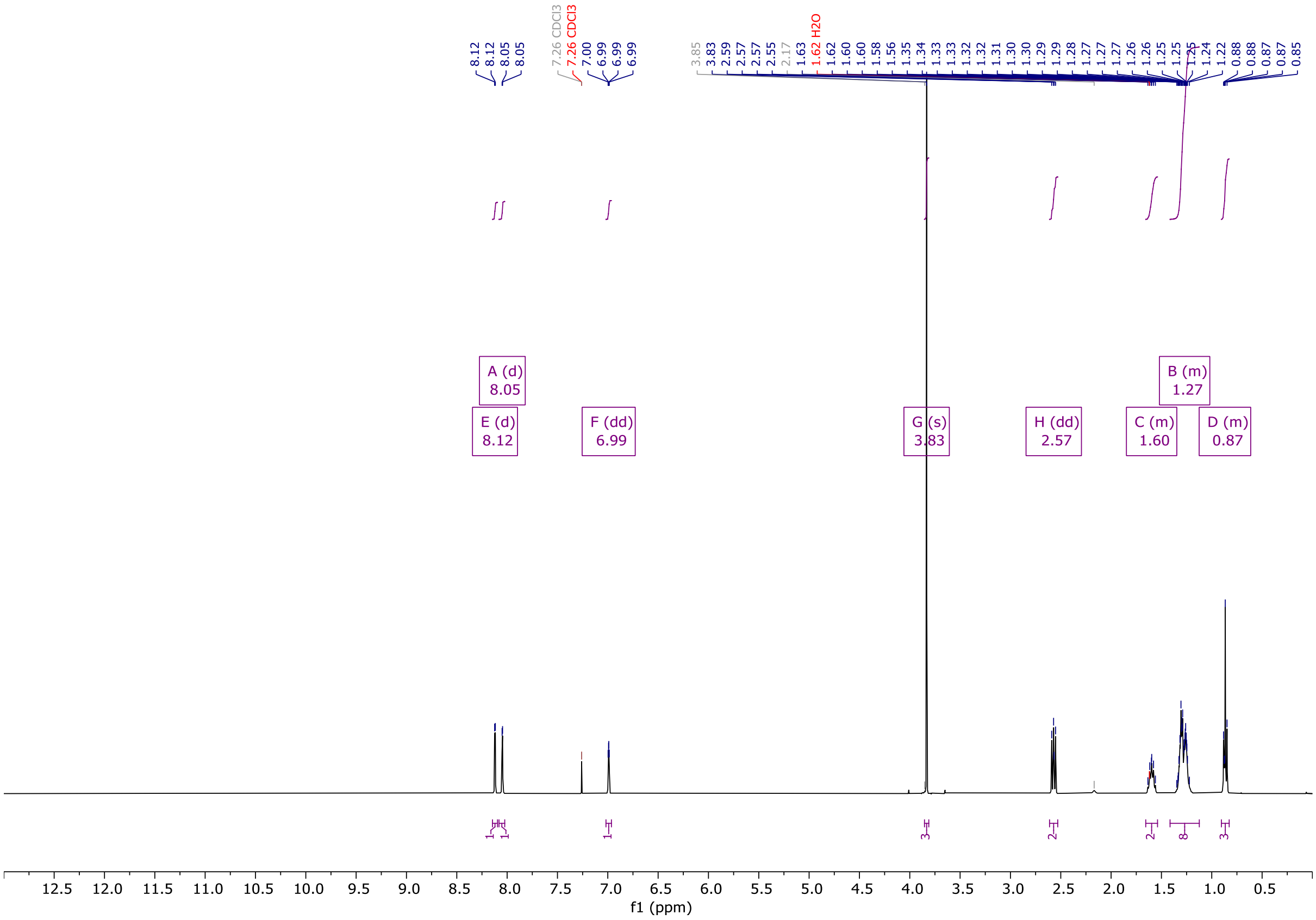

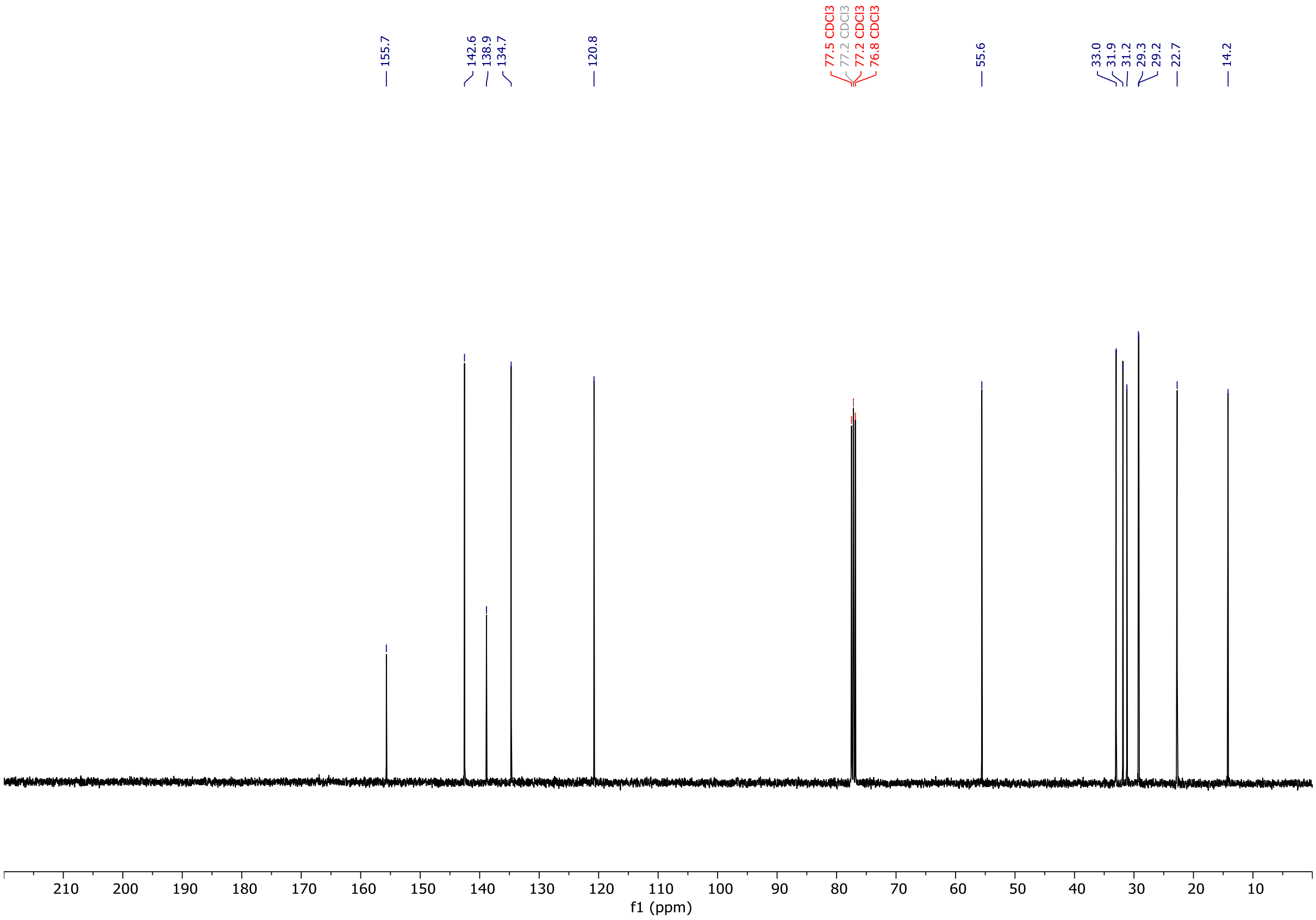

OX04539b

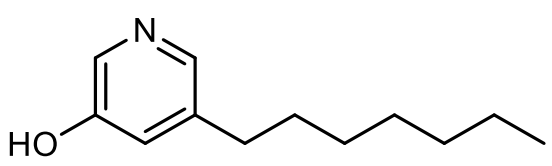

OX04539b

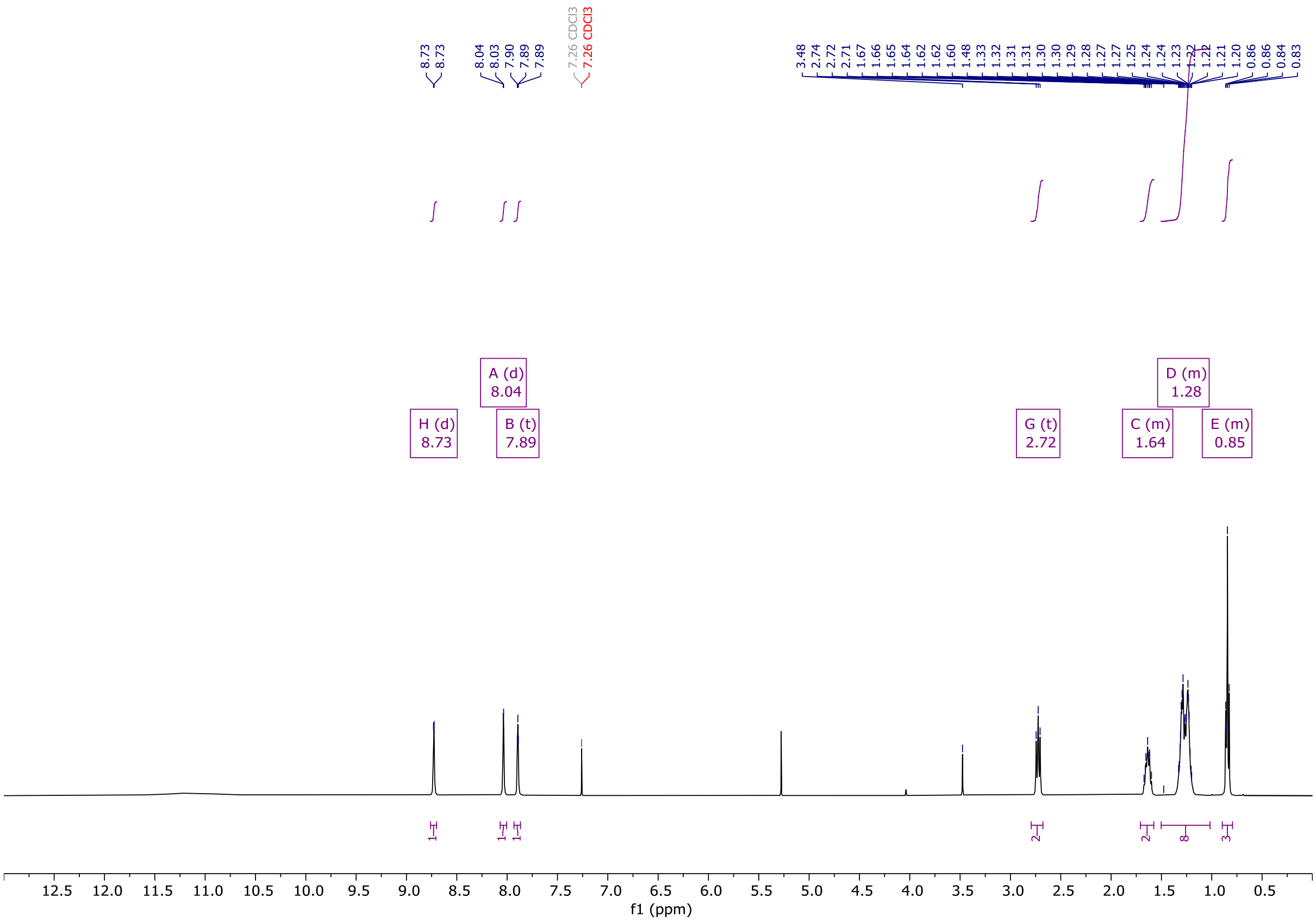

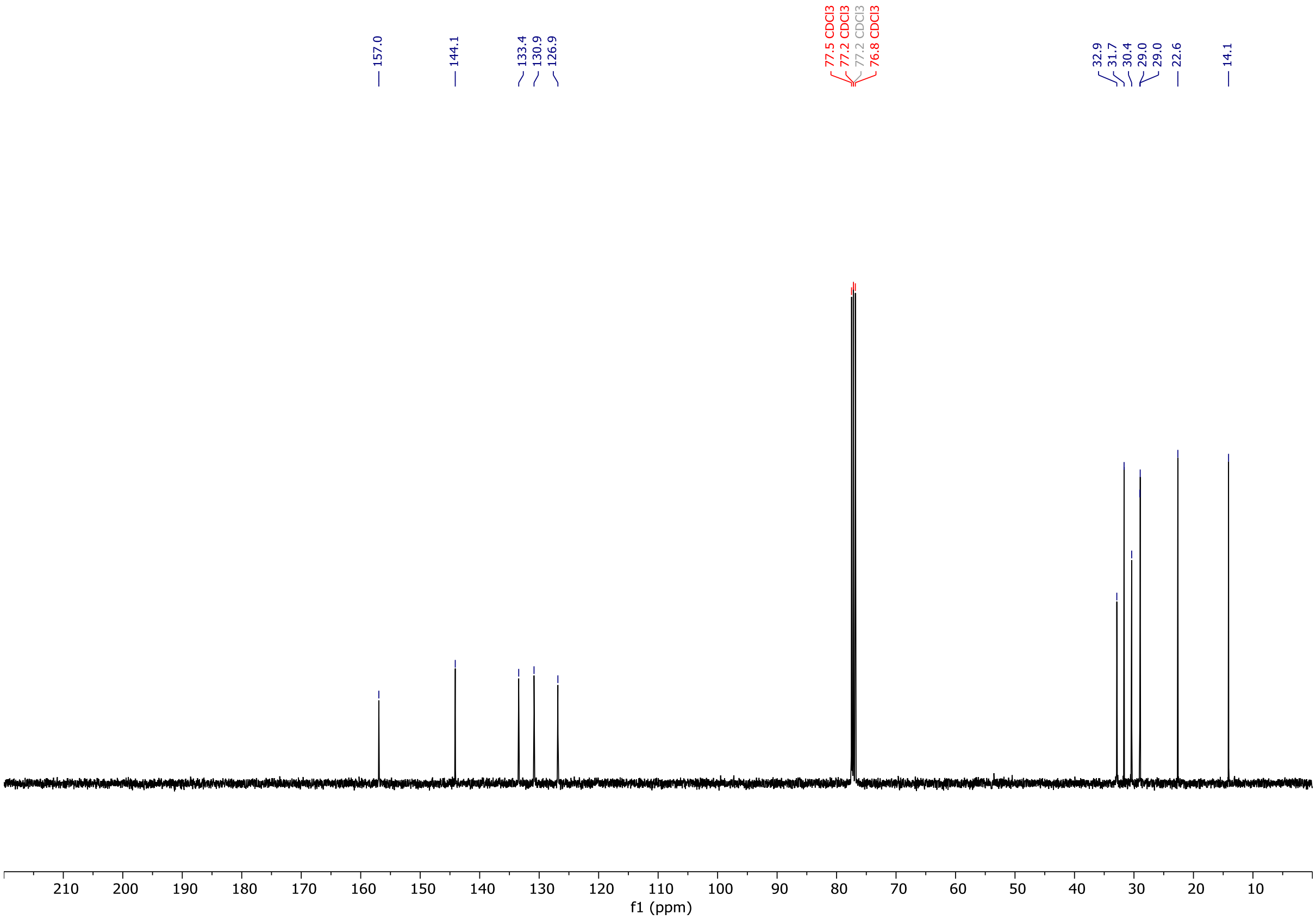
157.0
144.1
133.4
130.9
126.9
77.5 CDCl3
77.2 CDCl3
77.2 CDCl3
76.8 CDCl3
32.9
31.7
30.4
29.0
29.0
22.6
14.1
210 200 190 180 170 160 150 140 130 120 110 100 90 80 70 60 50 40 30 20 10
f1 (ppm)

OX04539

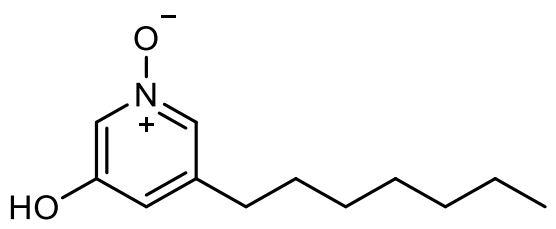

OX04539

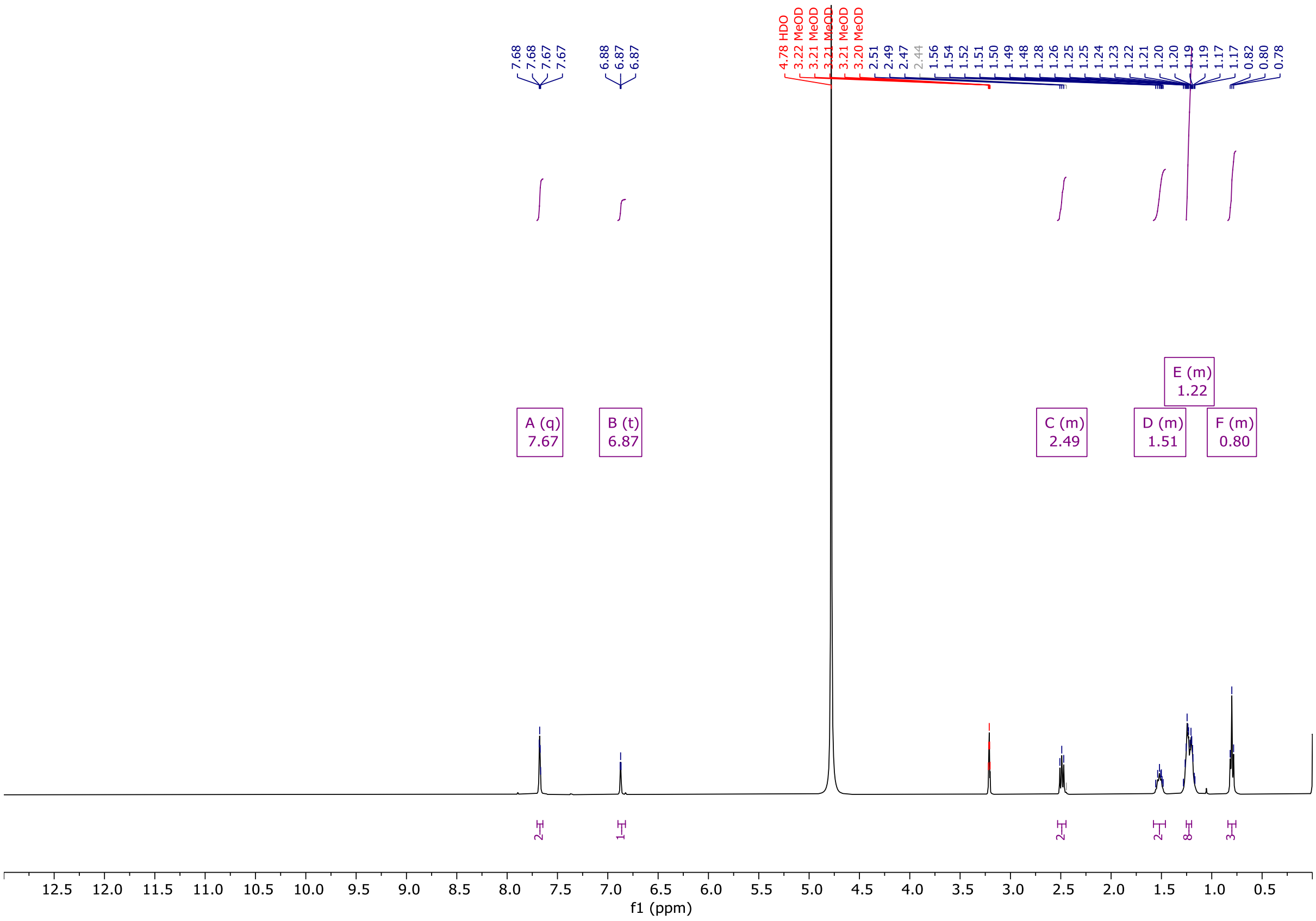

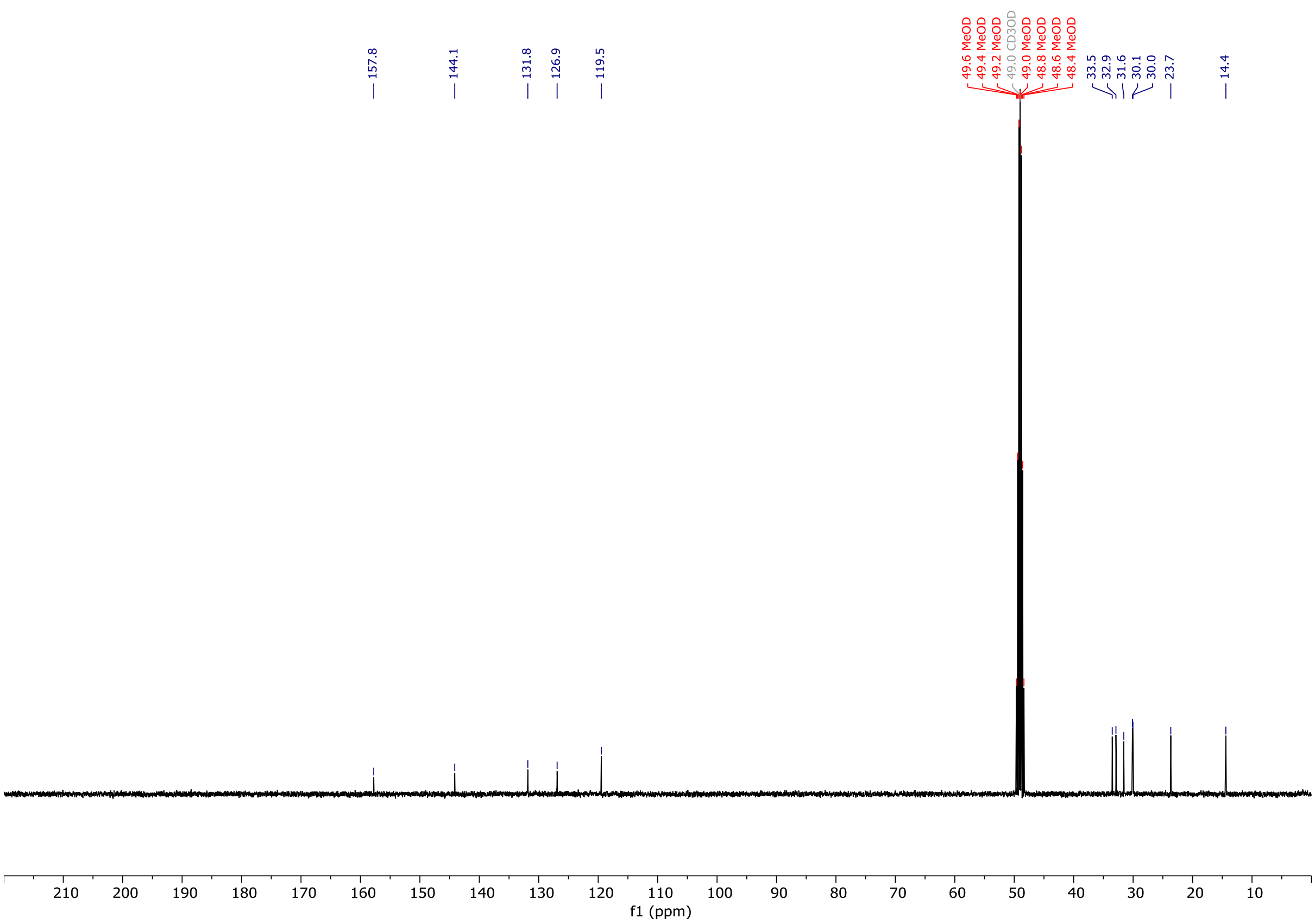

OX04540a

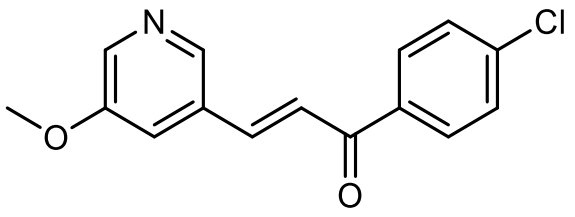

OX04540a

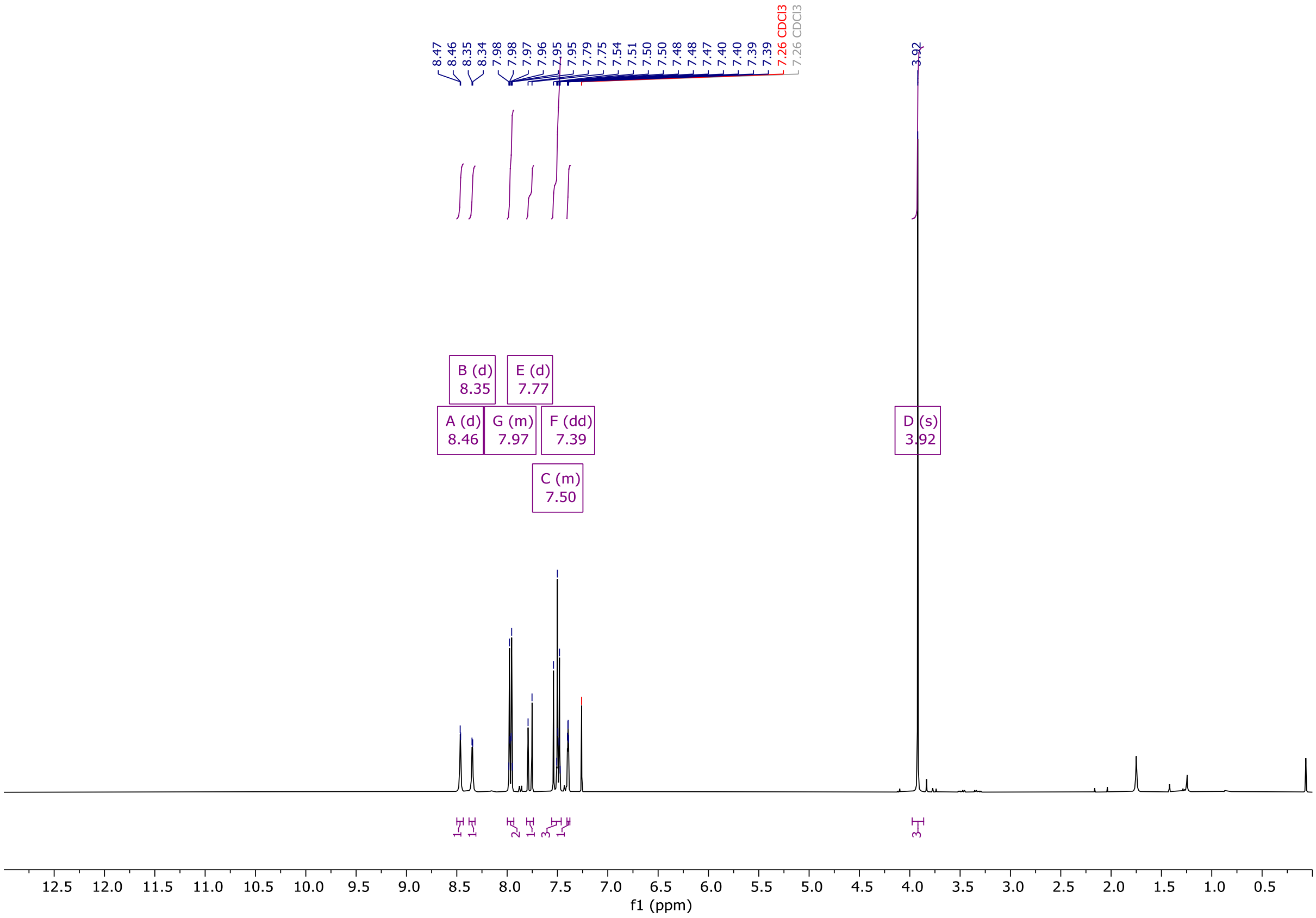

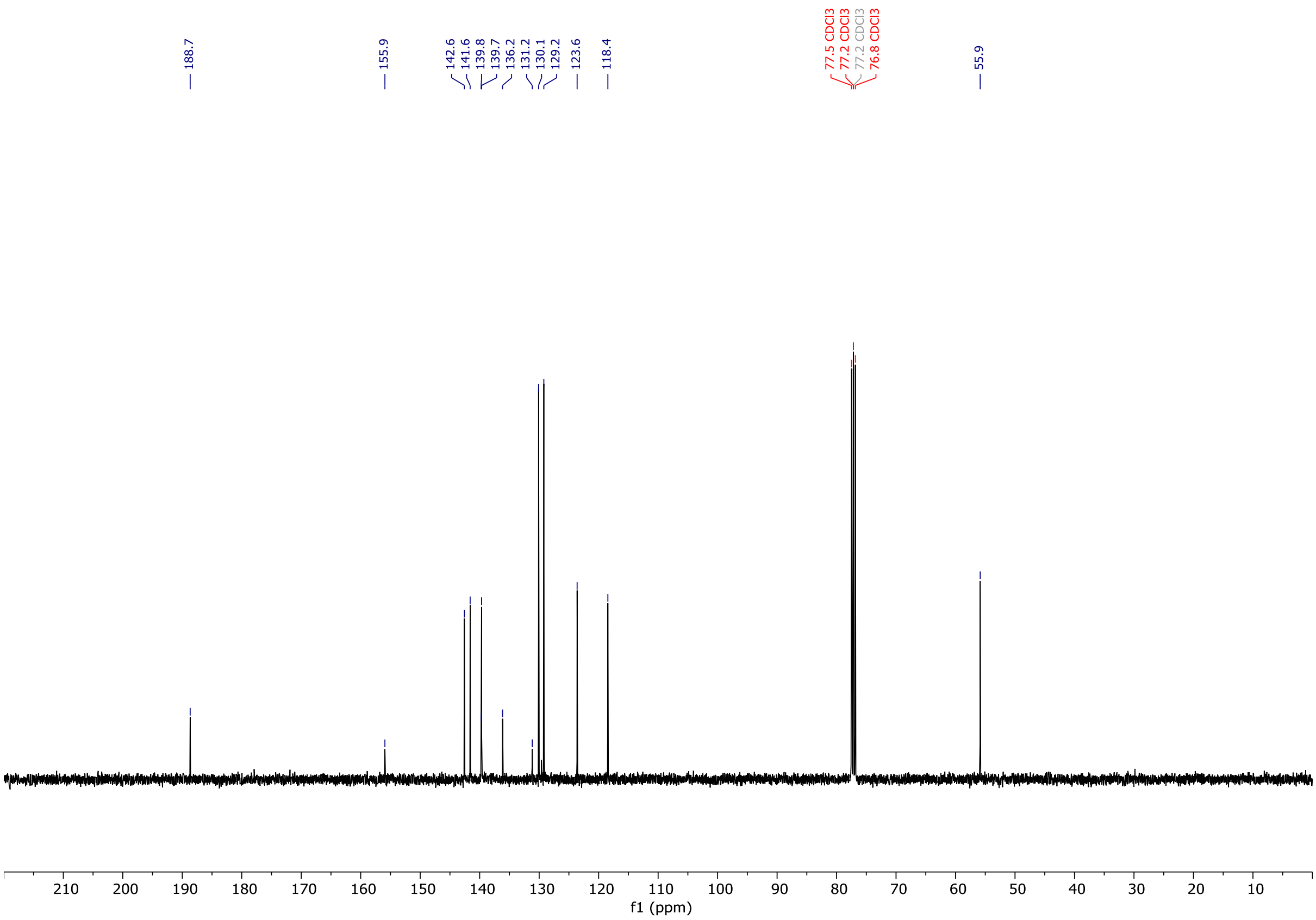

OX04540b

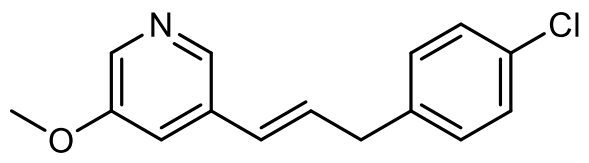

OX04540b

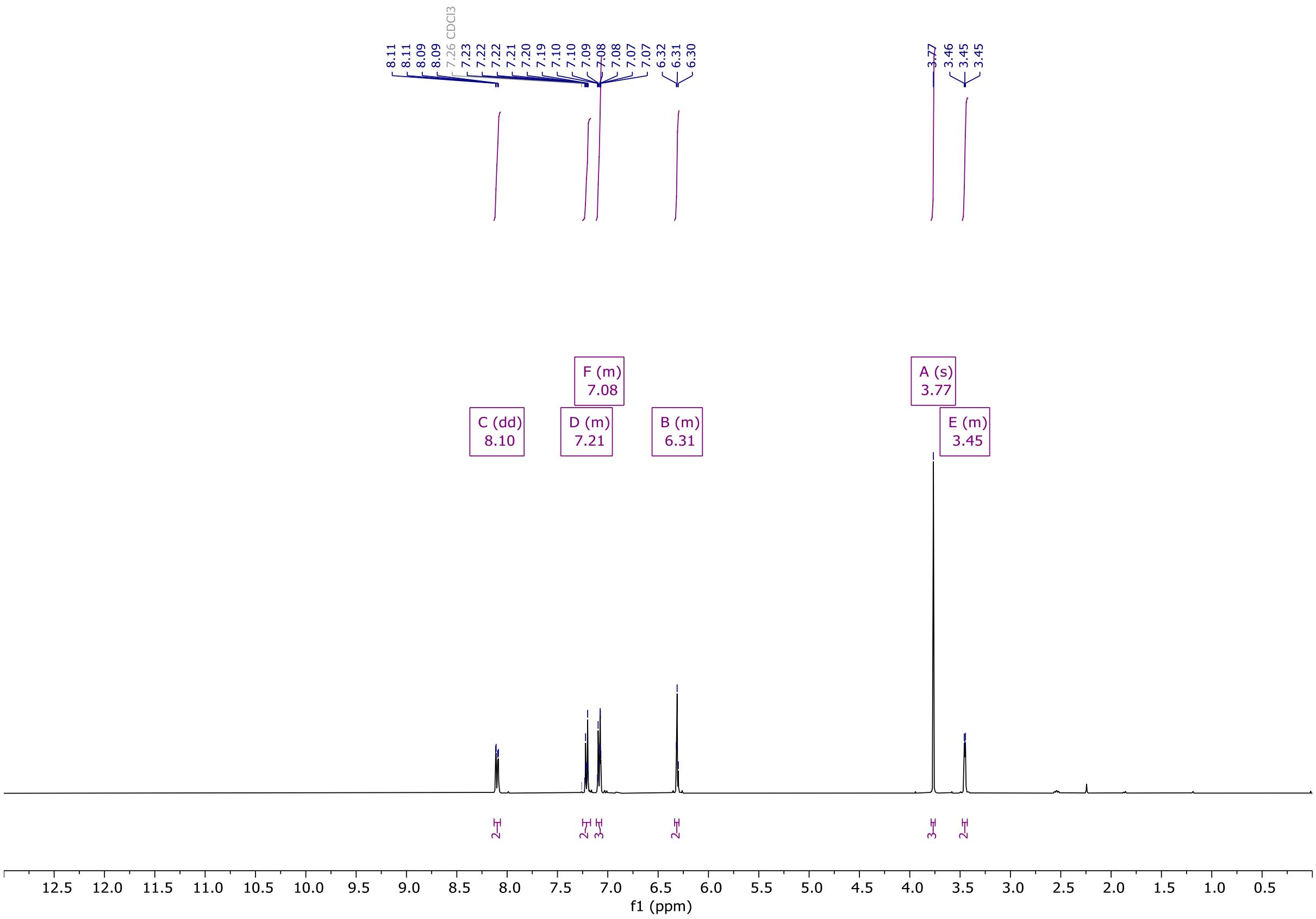

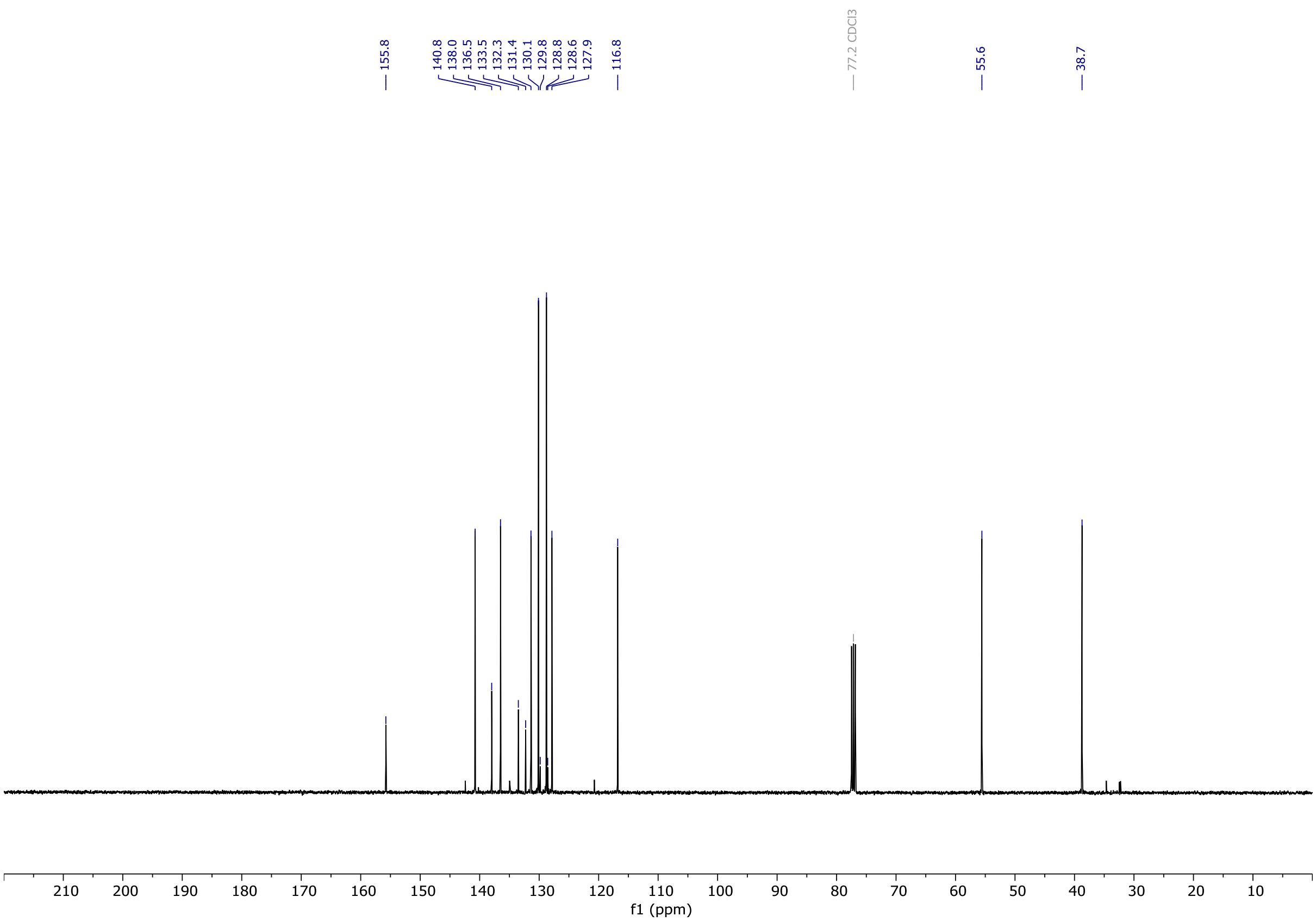

OX04540c

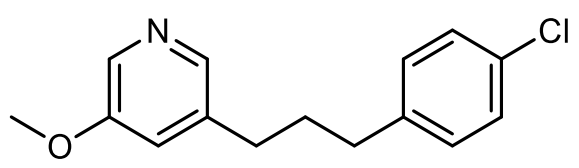

OX04540c

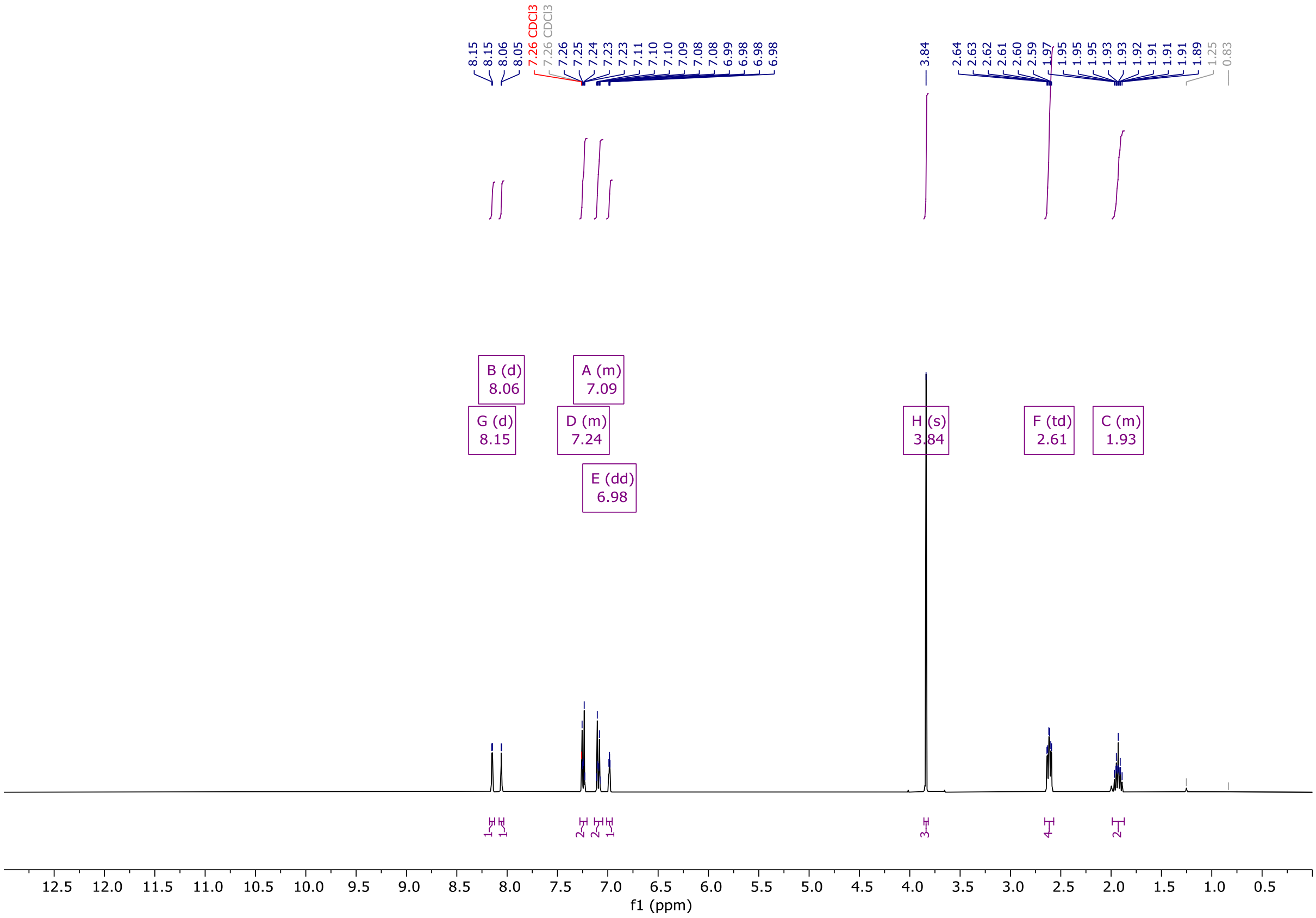

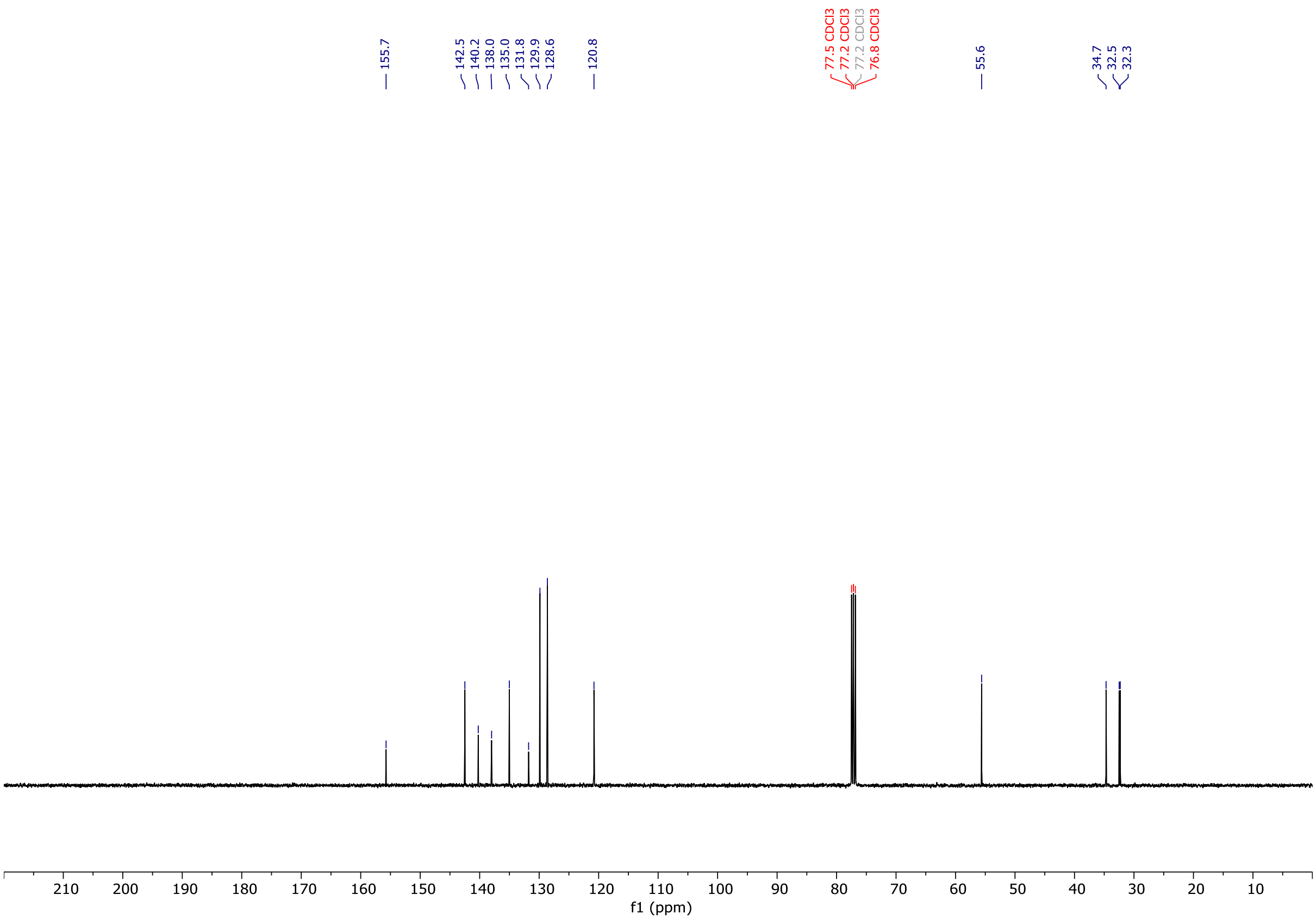

OX04540d

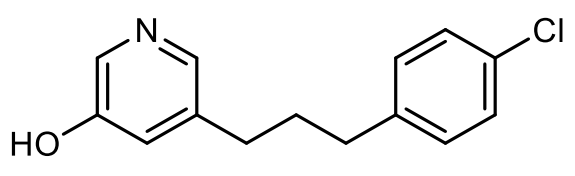

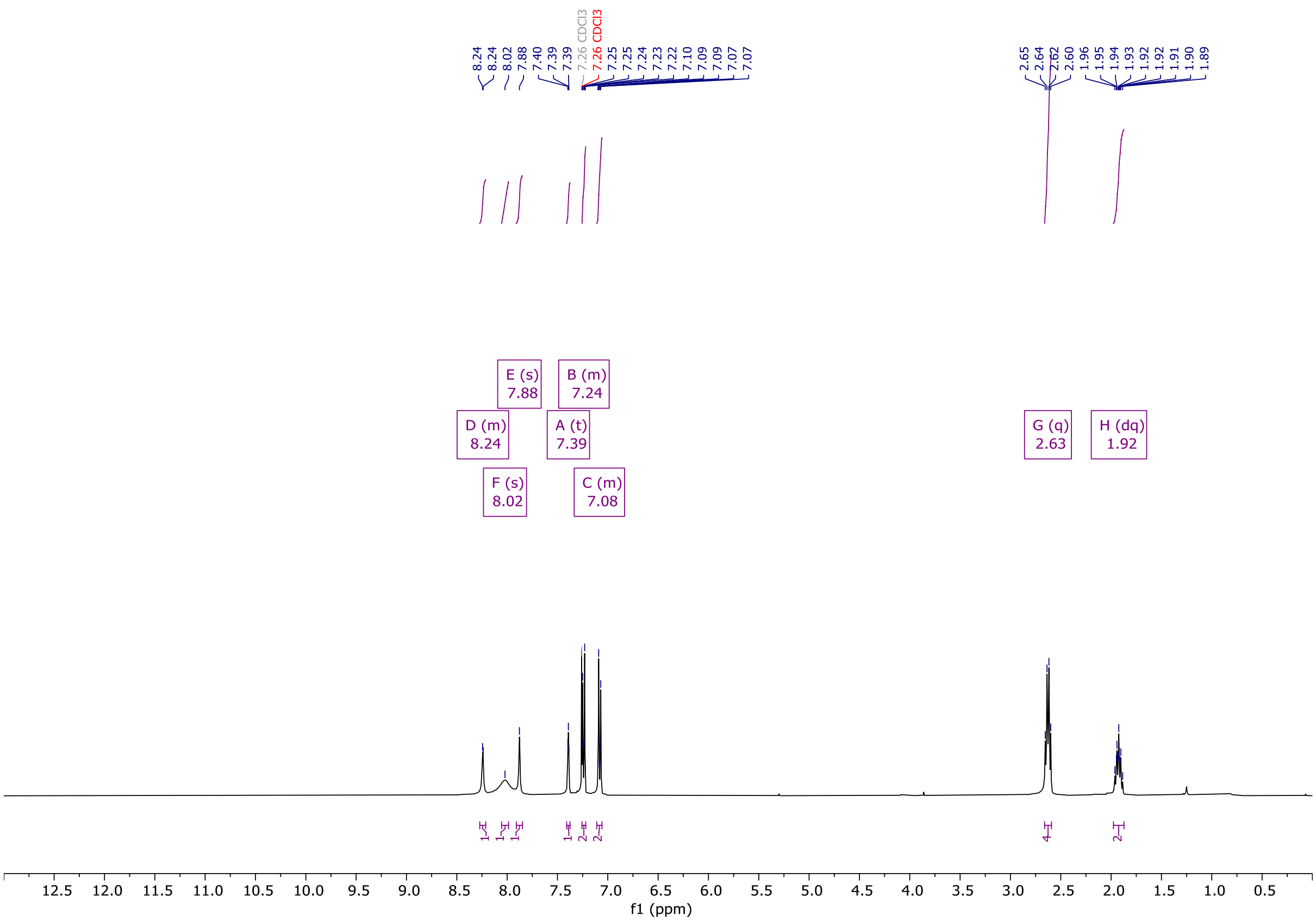

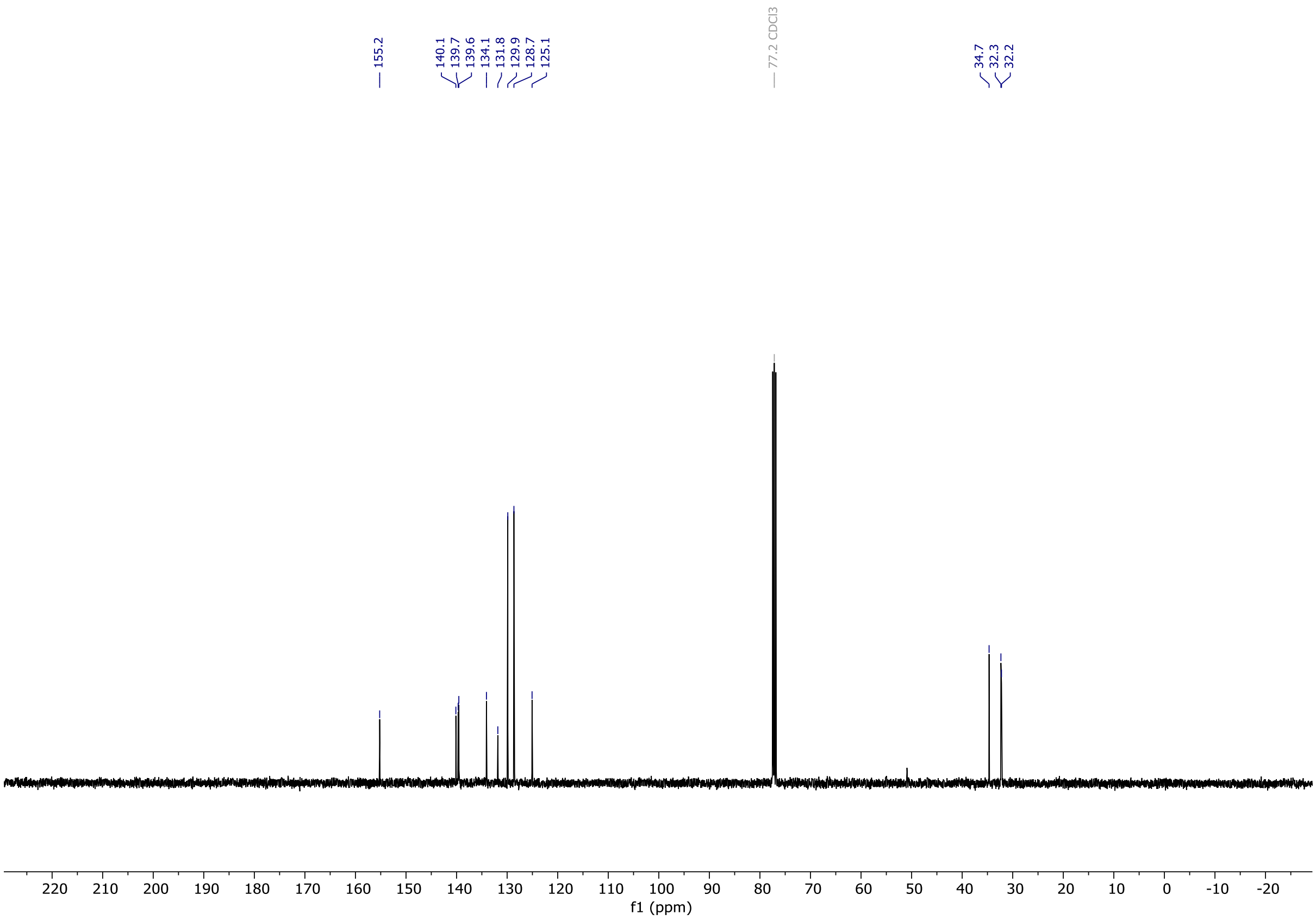

OX04540

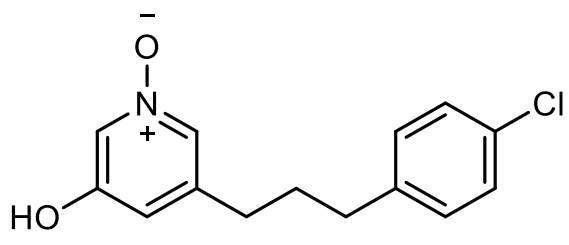

OX04540

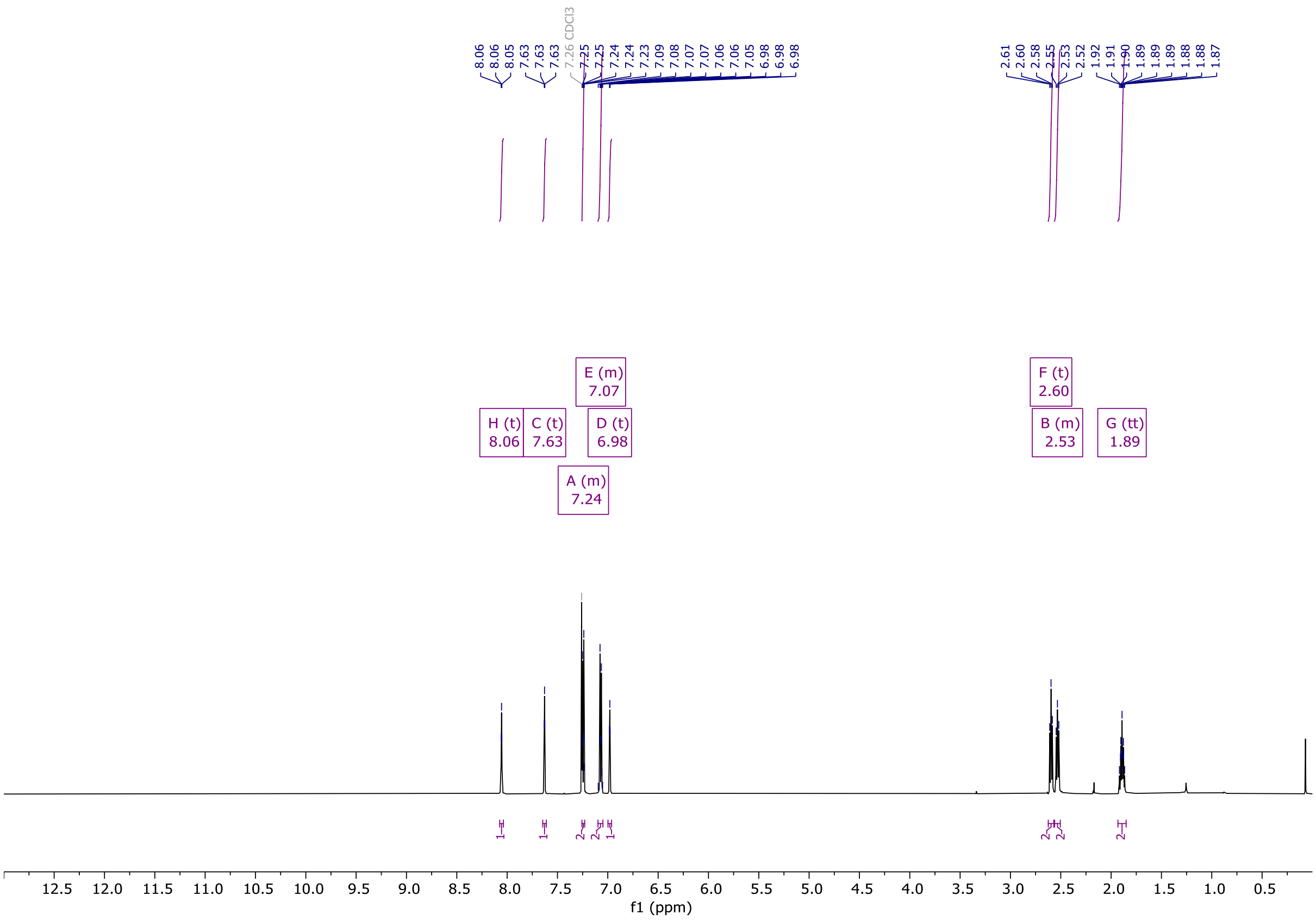

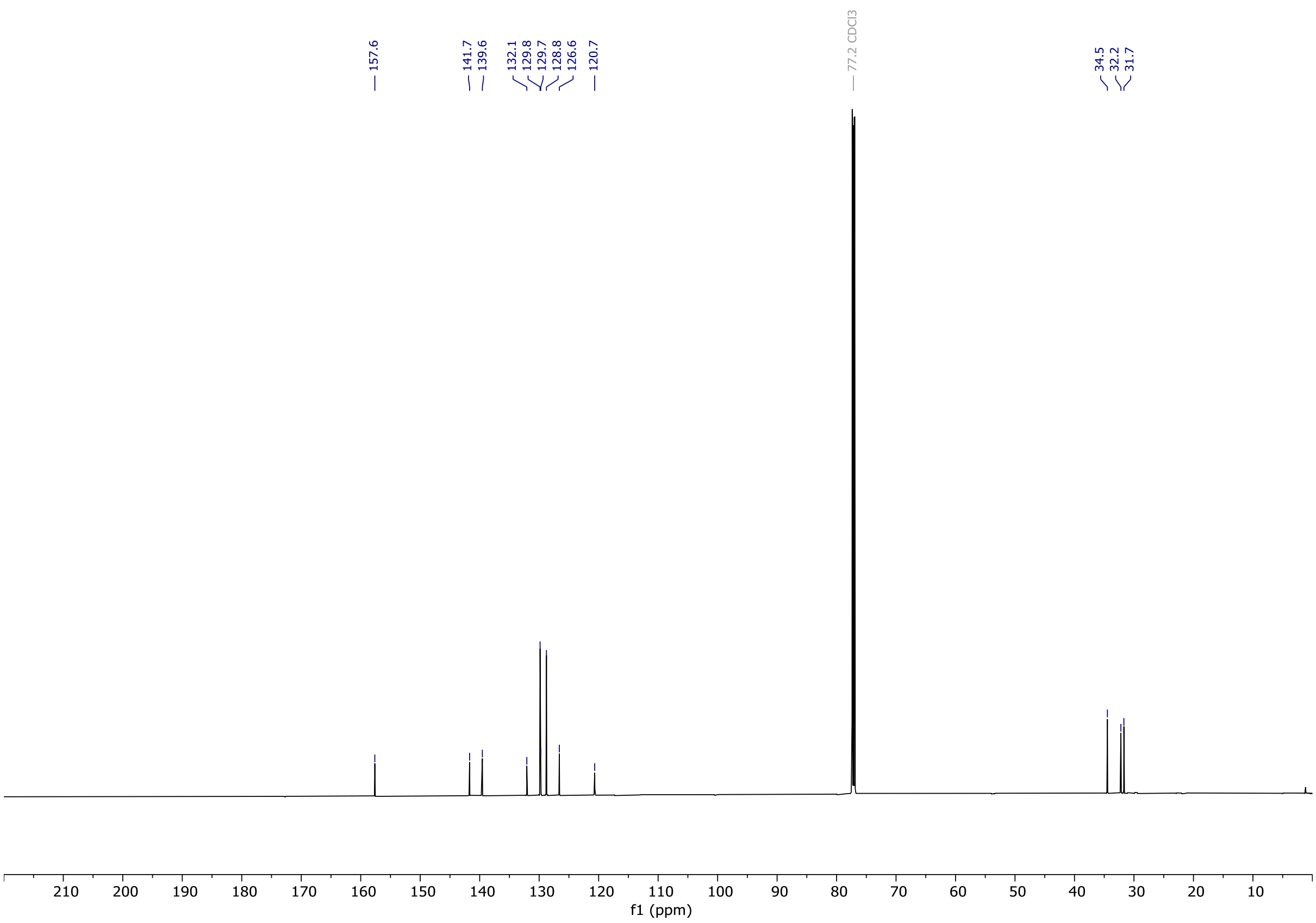

OX04954a

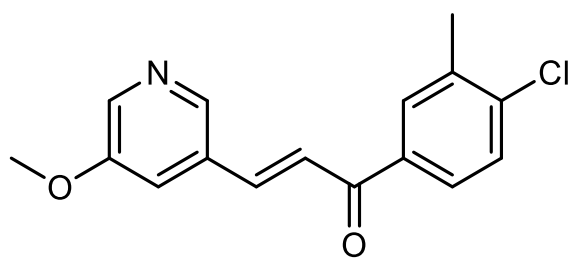

OX04954a

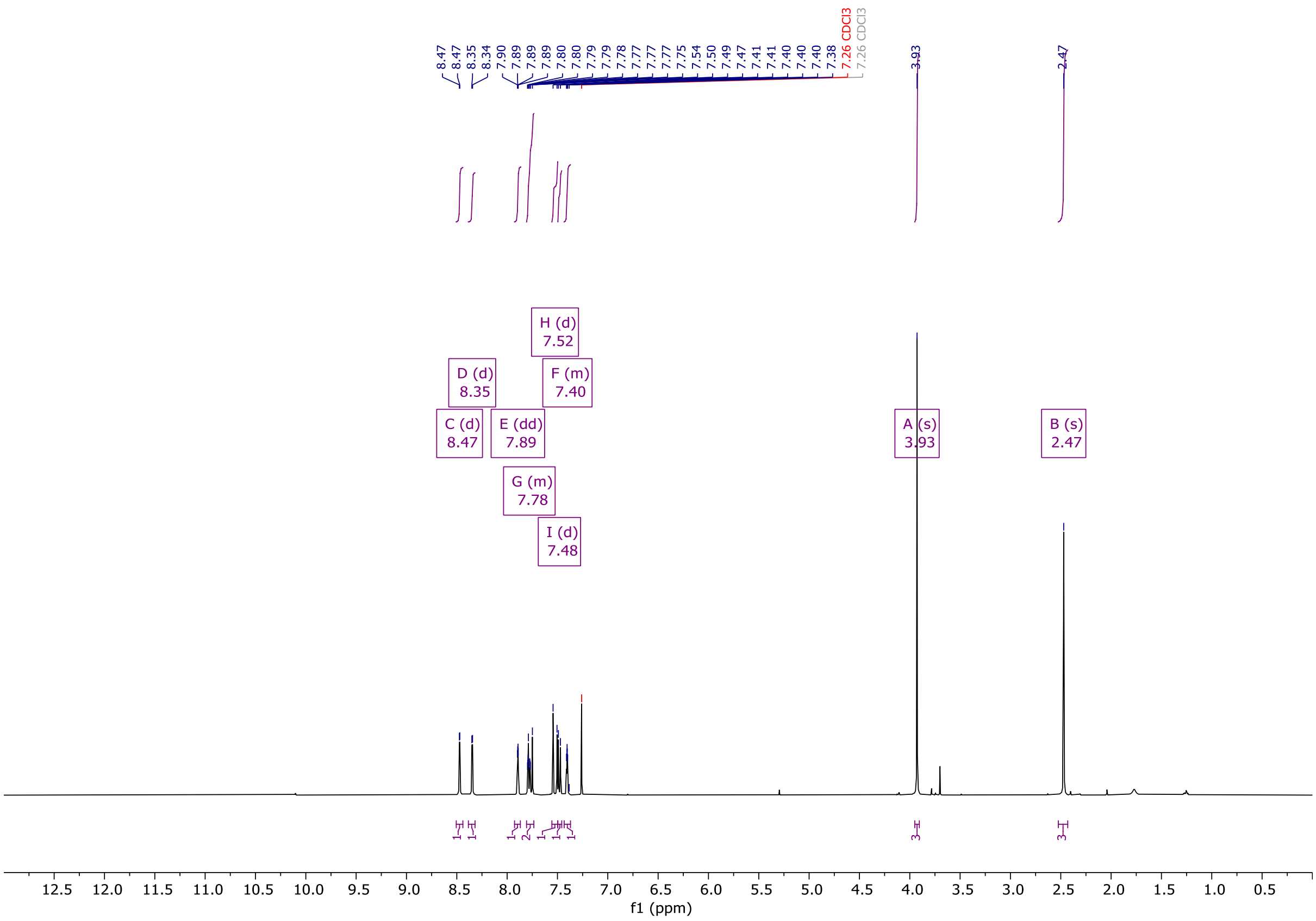

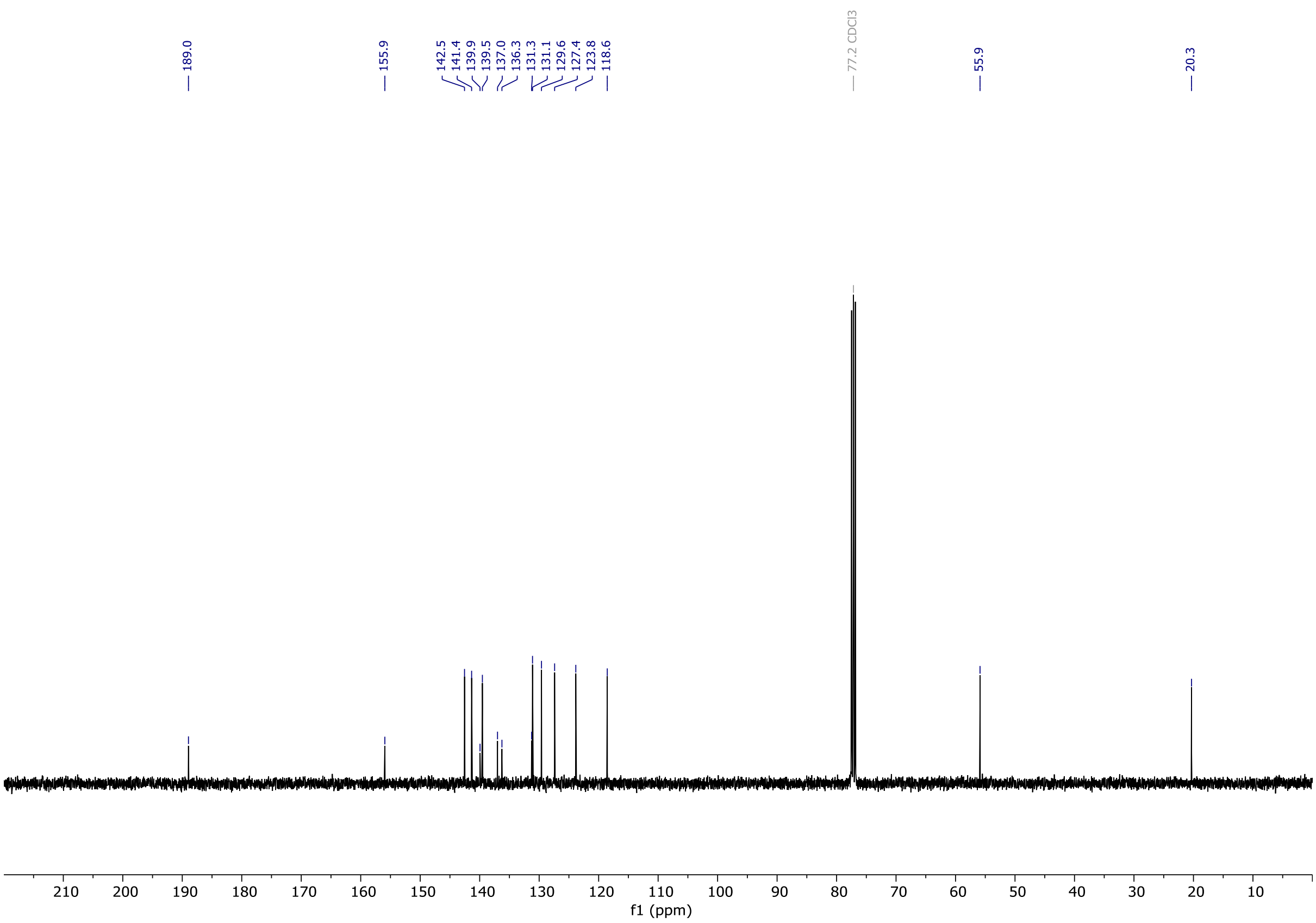

OX04954b

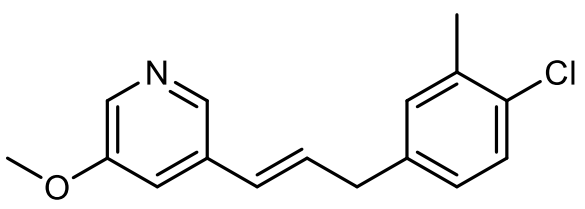

OX04954b

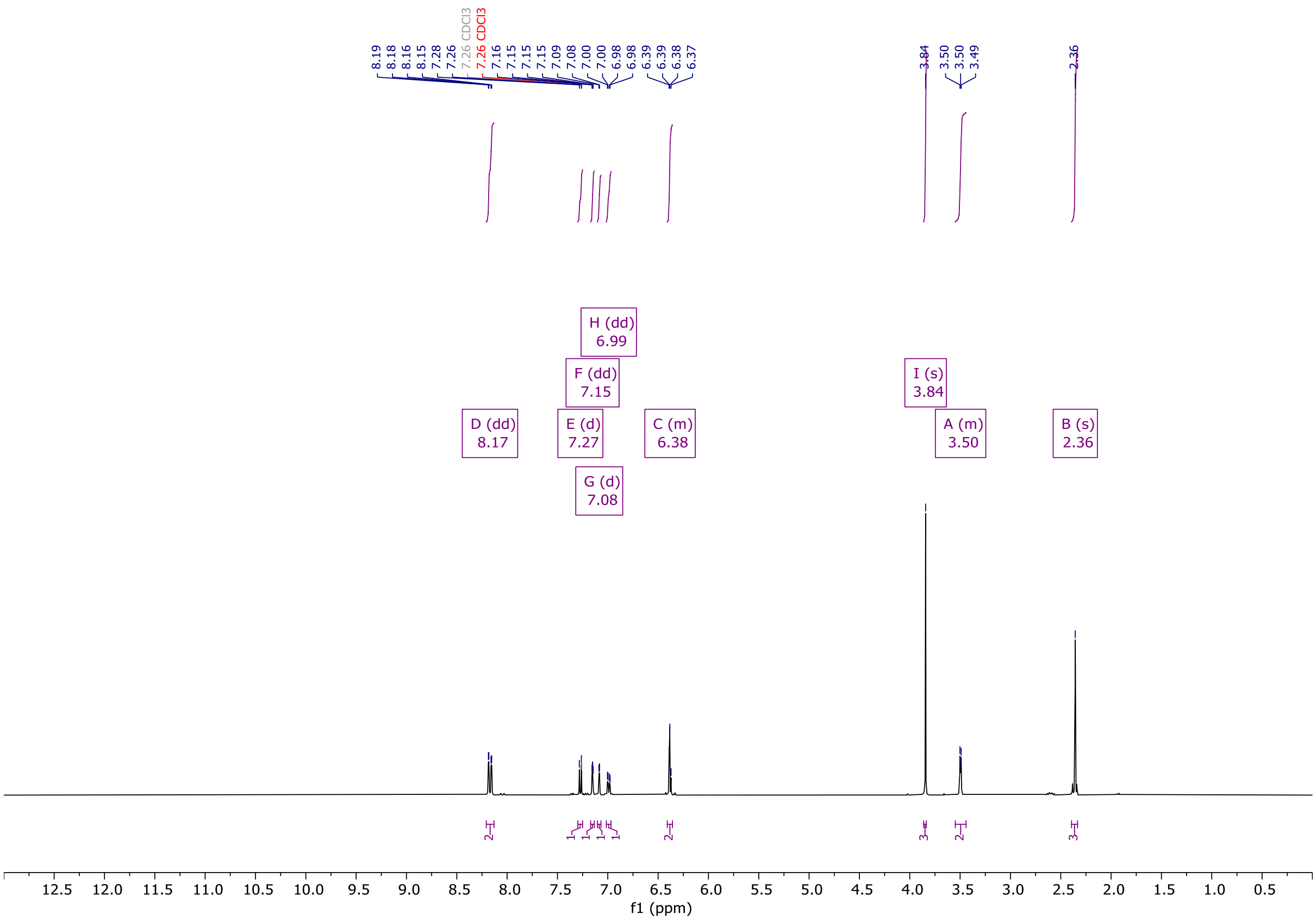

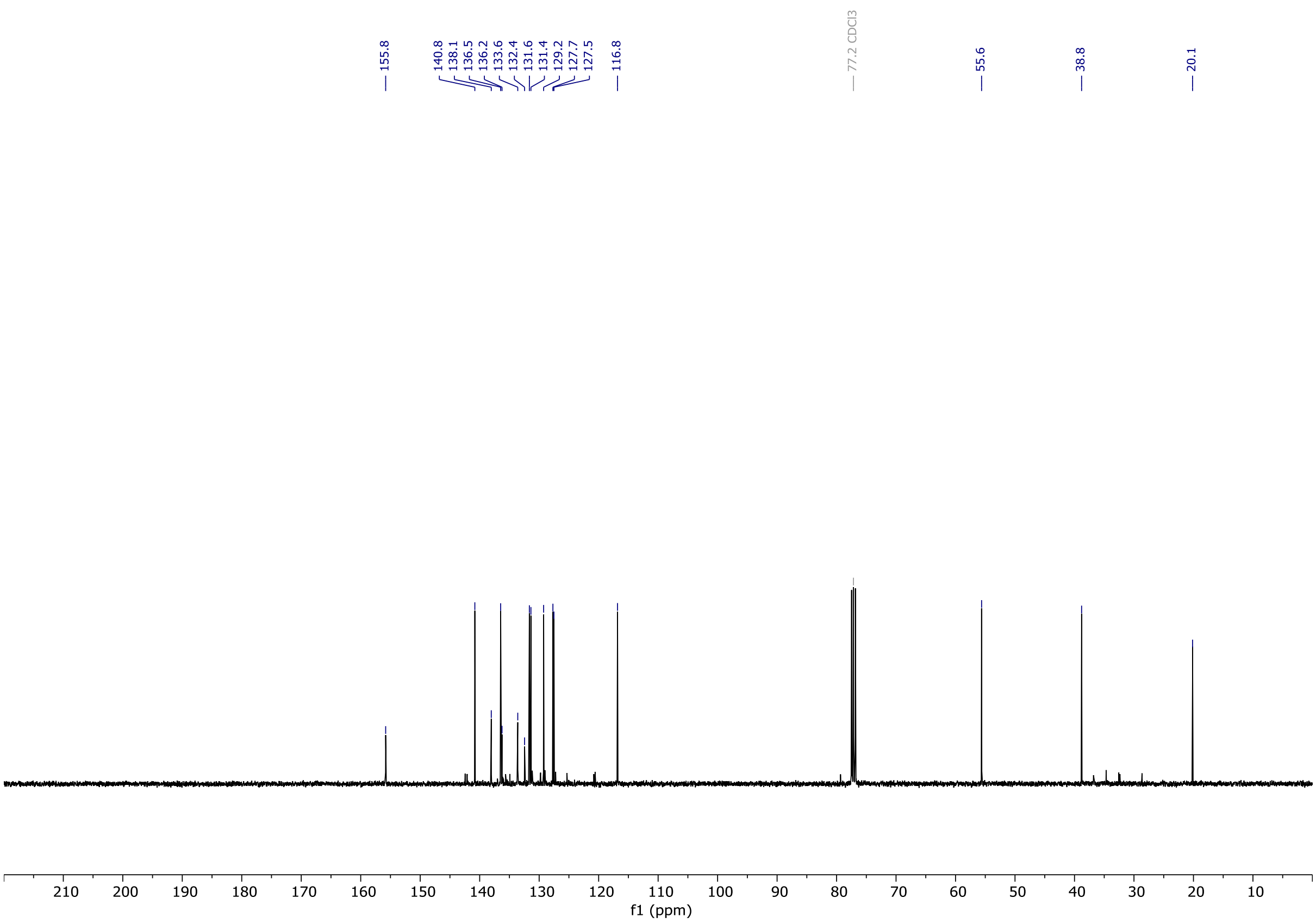

OX04954c

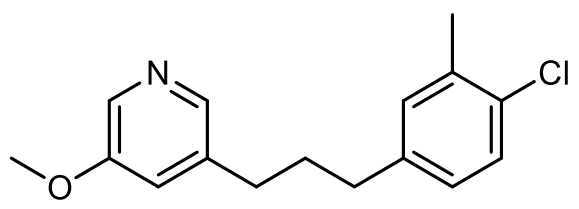

OX04954c

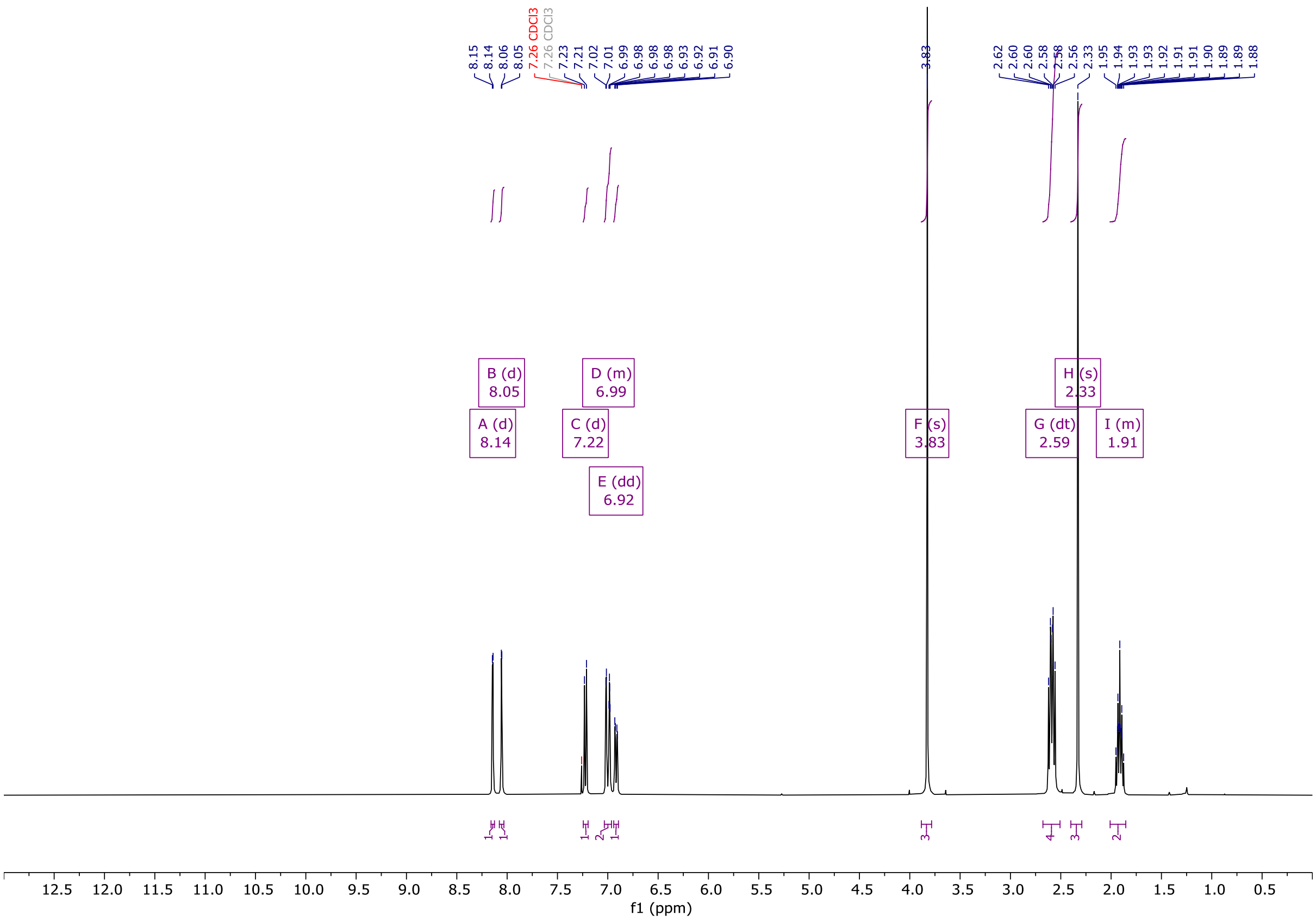

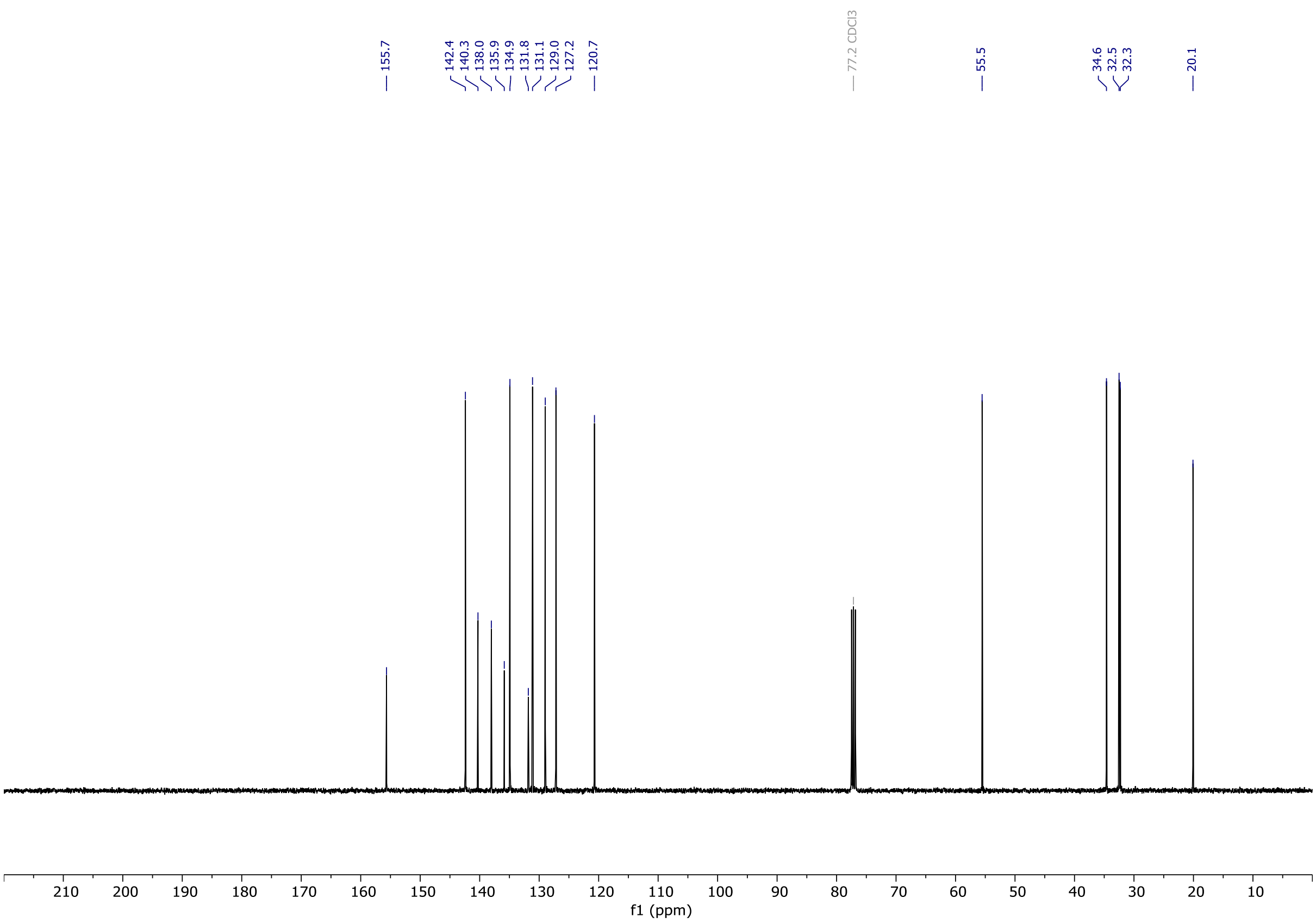

OX04954d

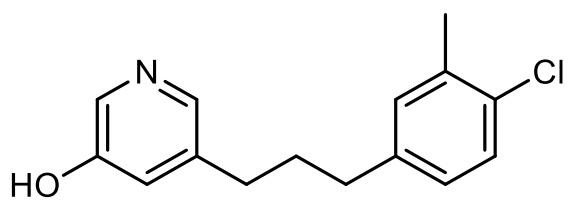

OX04954d

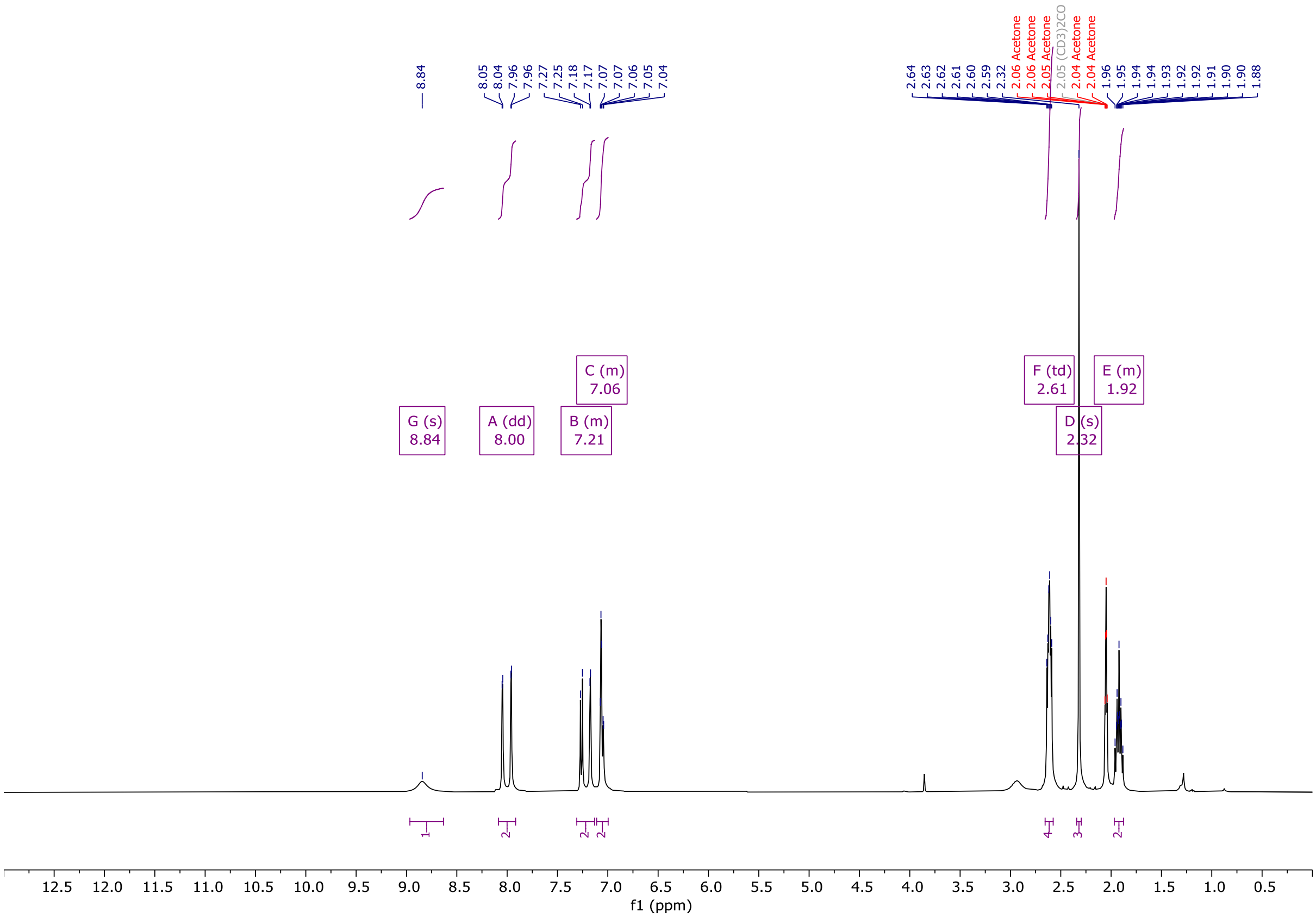

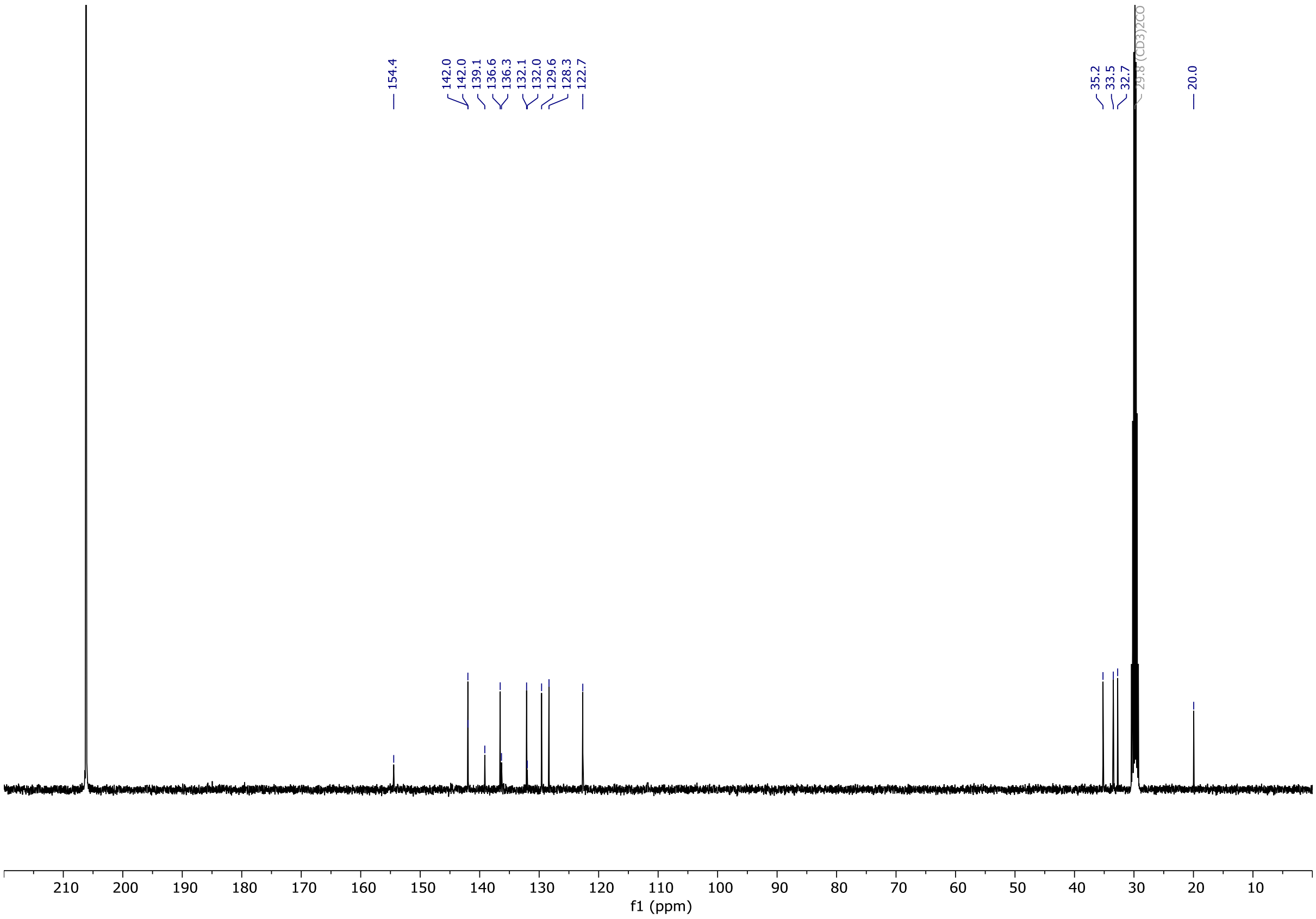

OX04954

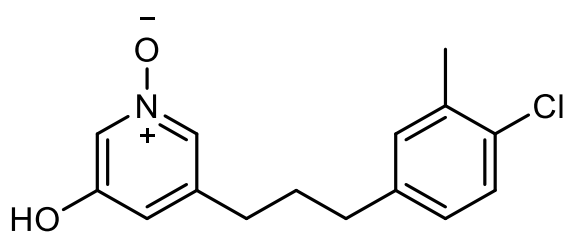

OX04954

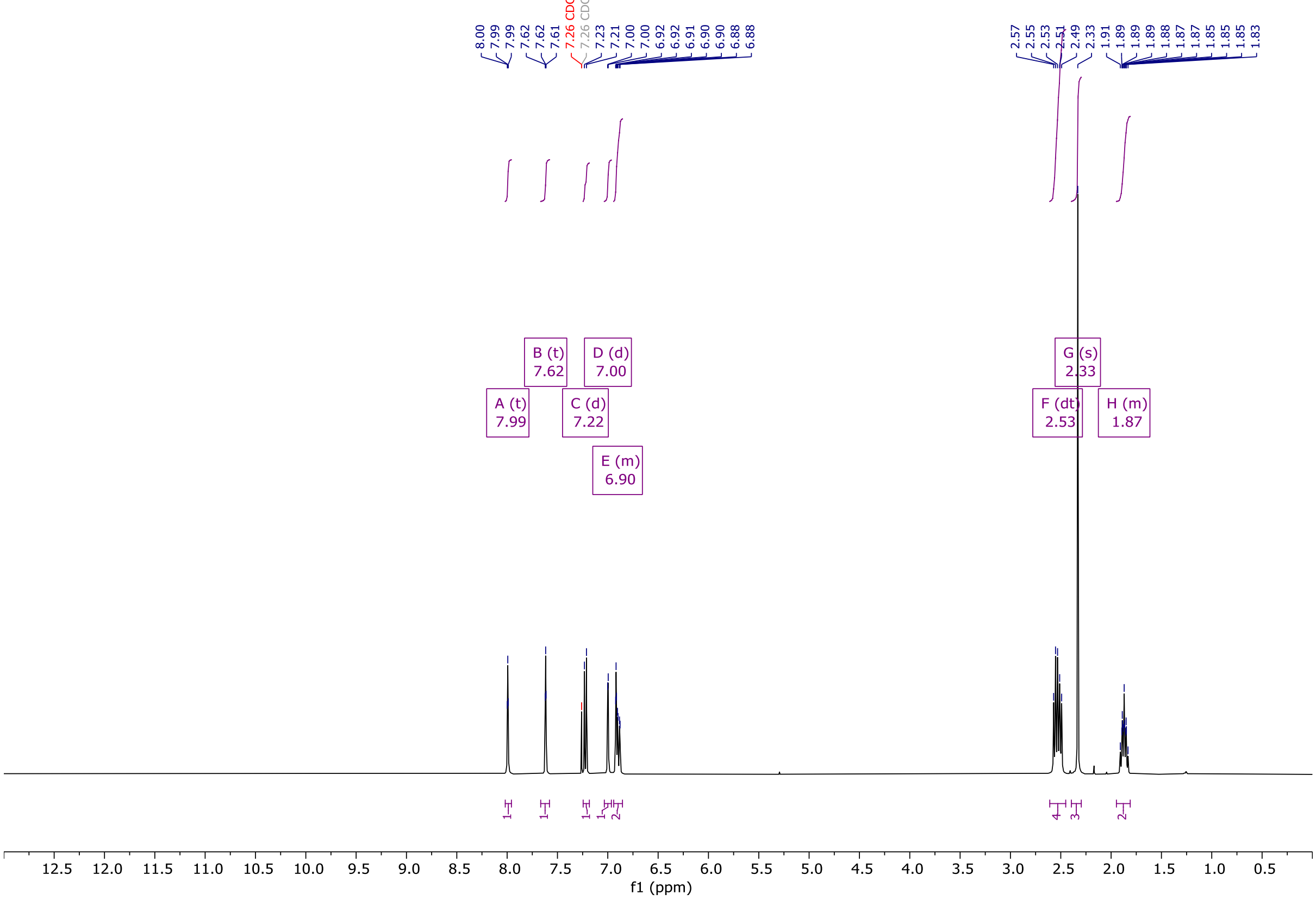

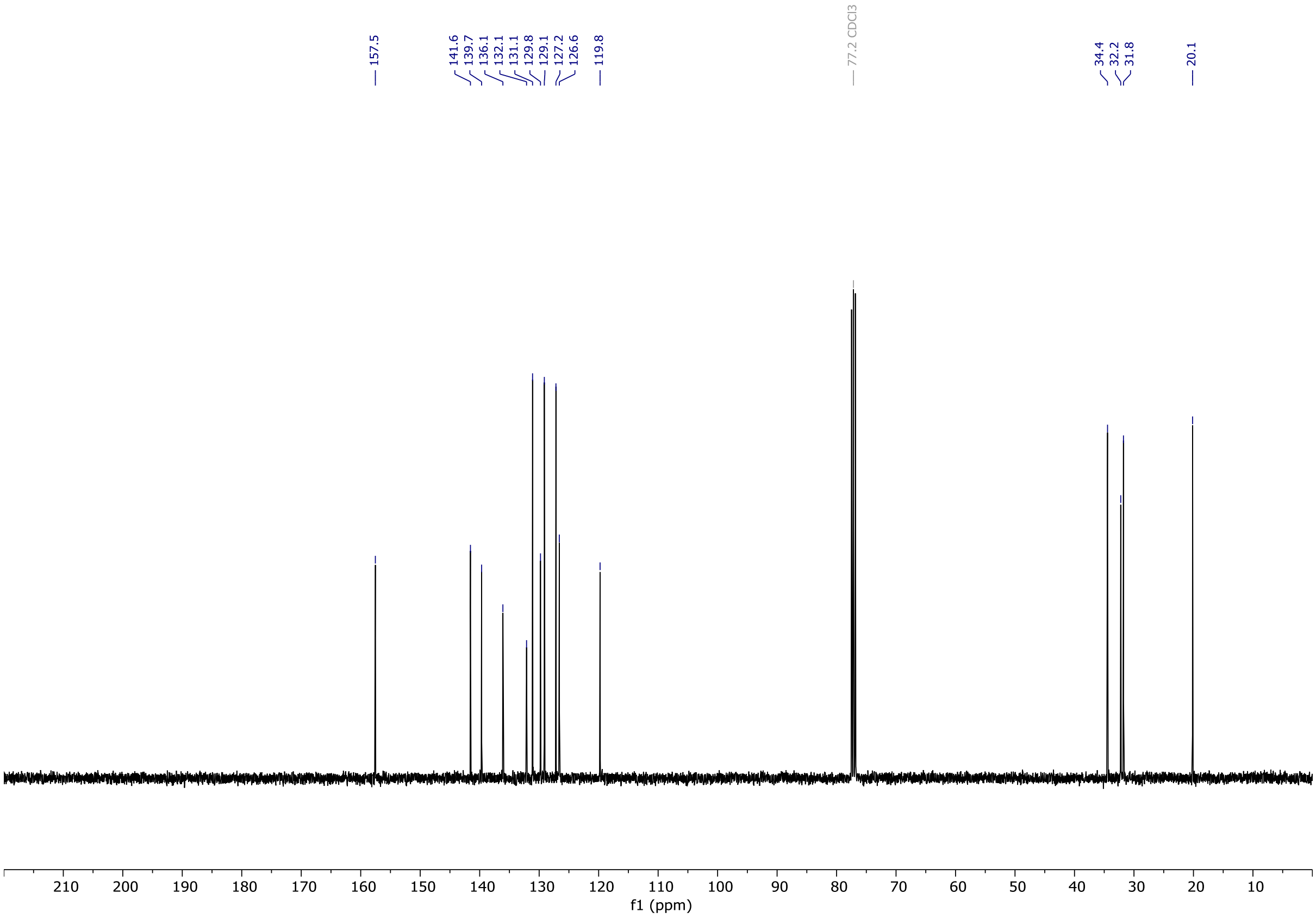

OX04956a

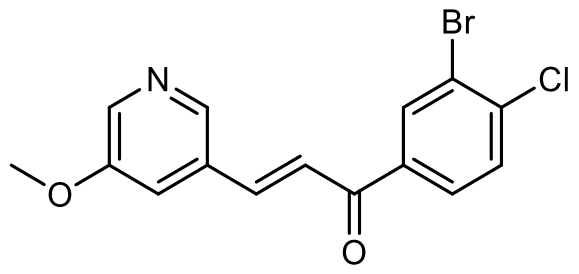

OX04956a

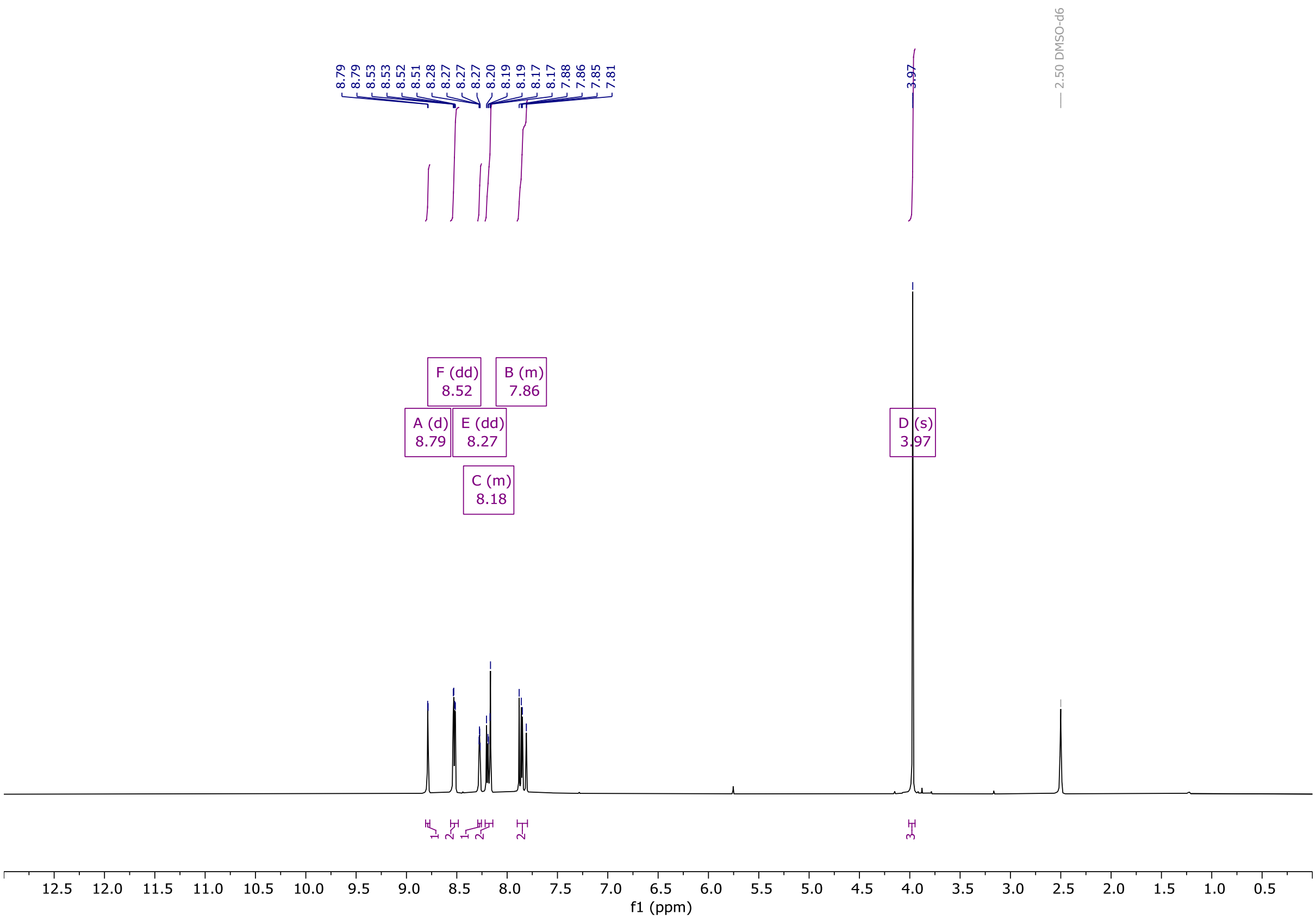

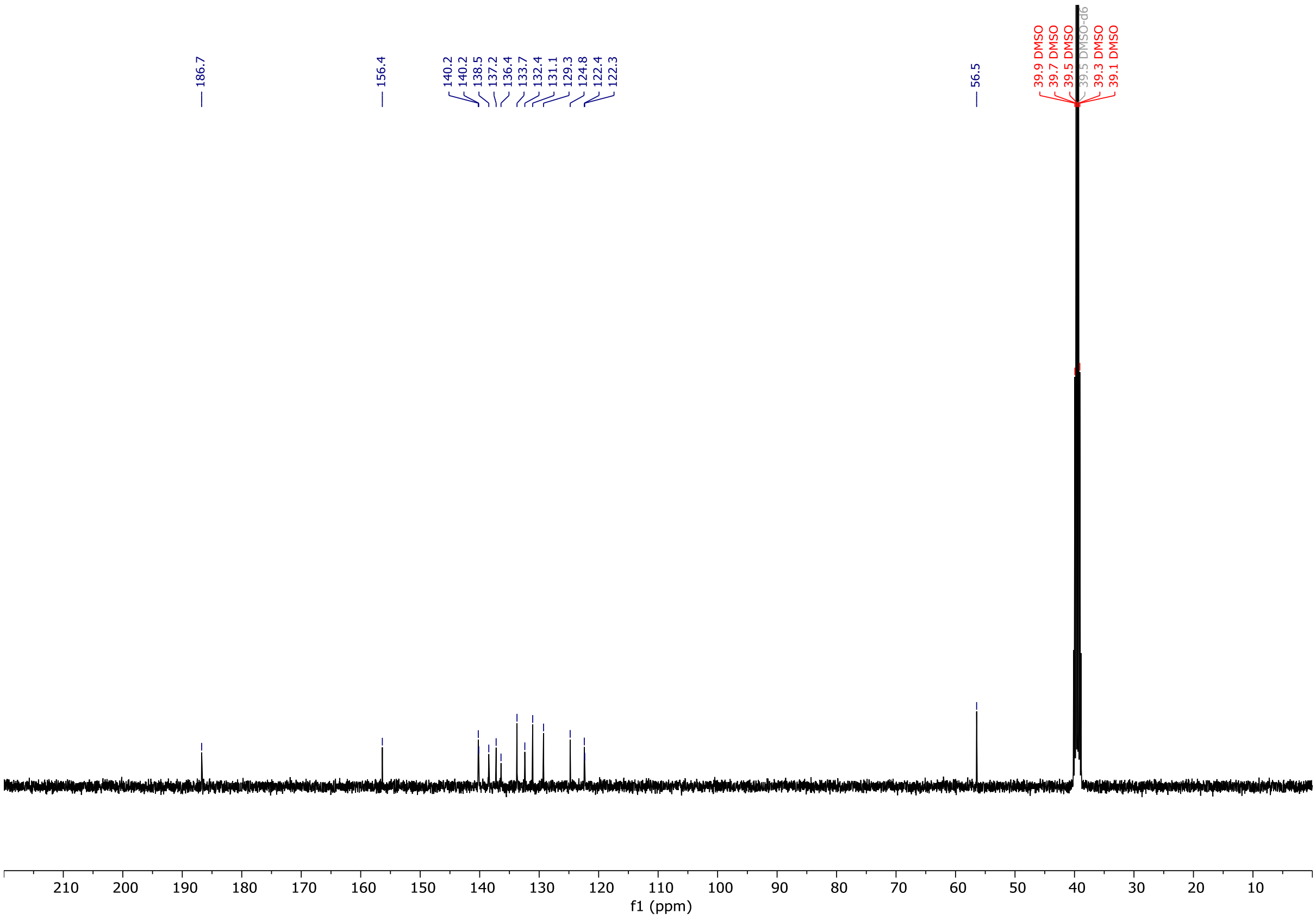

OX04956b

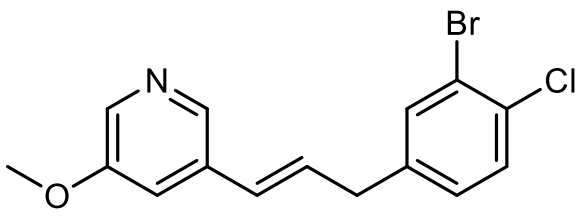

OX04956b

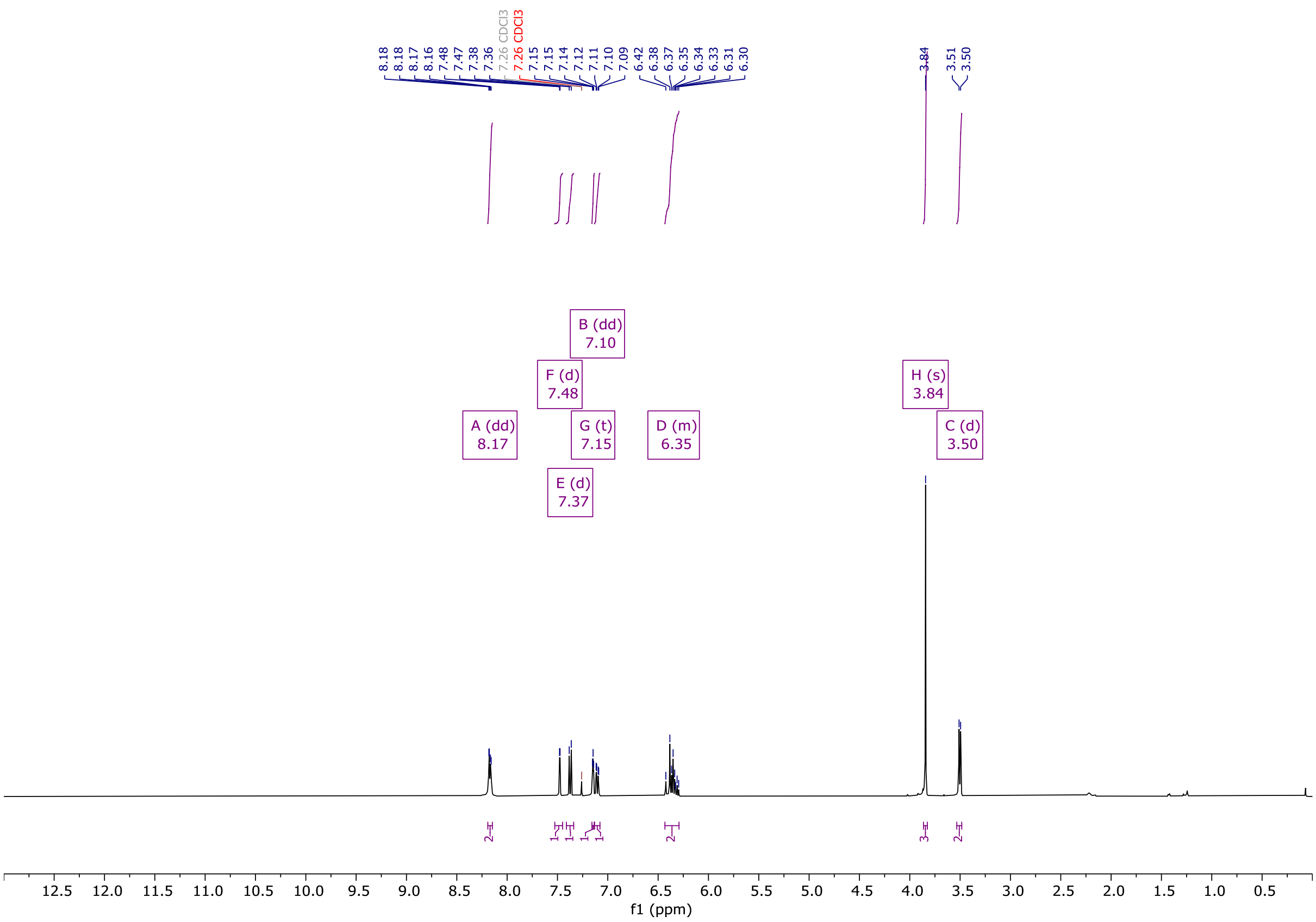

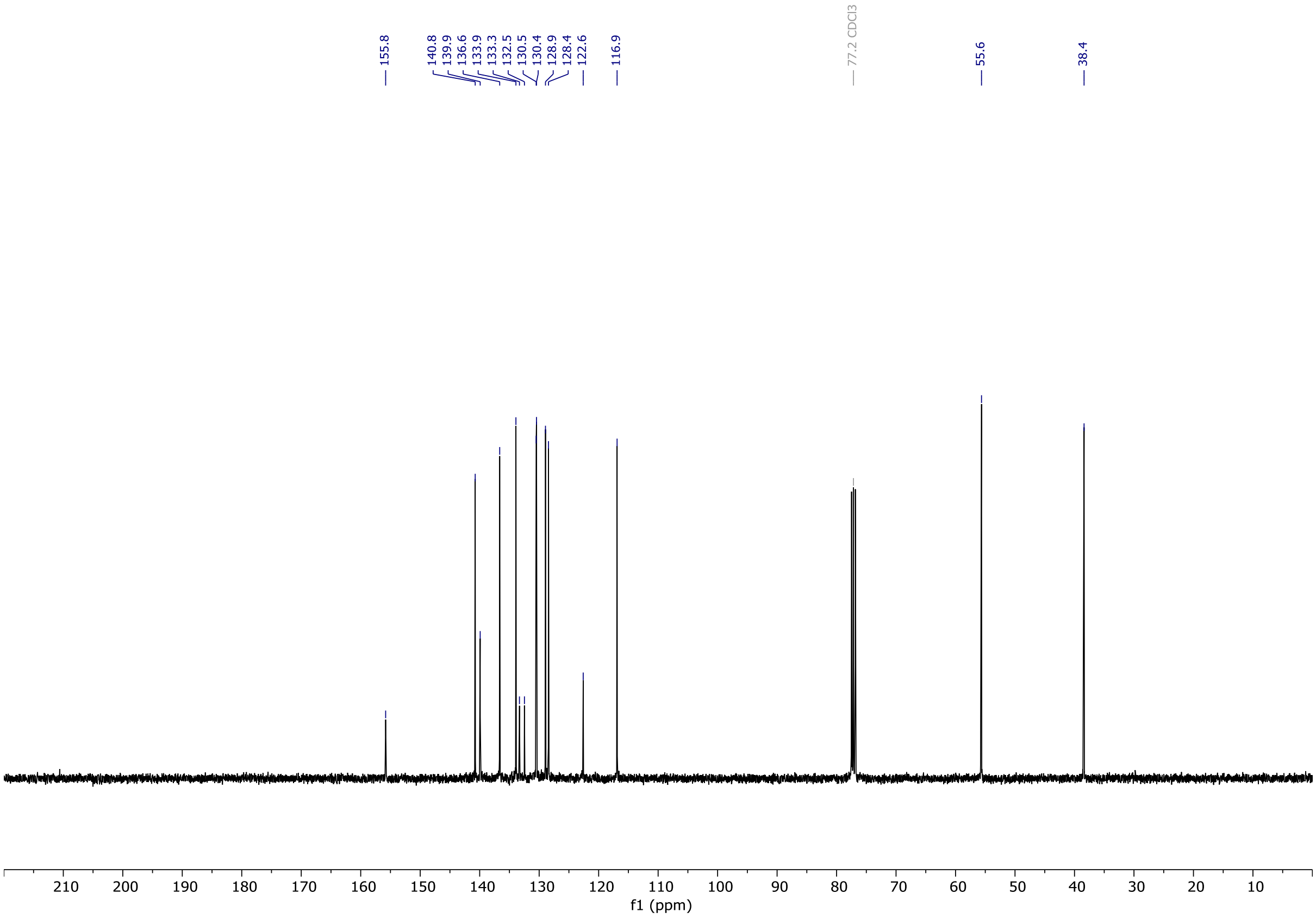

OX04956c

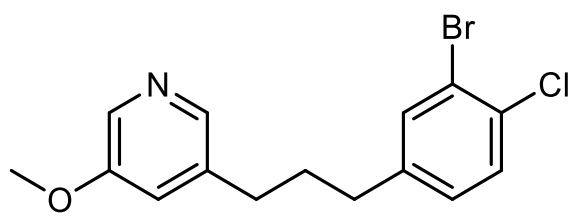

OX04956c

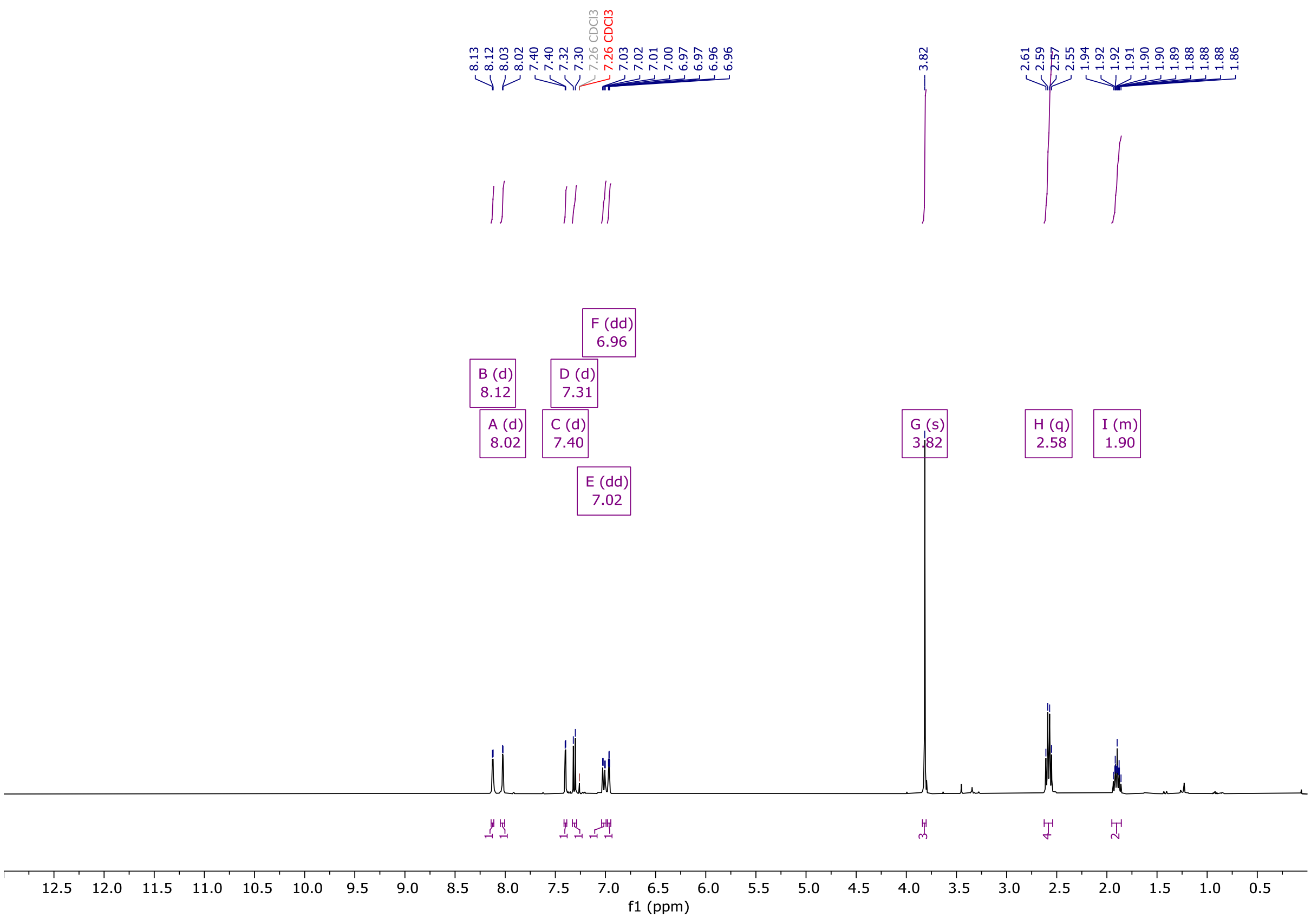

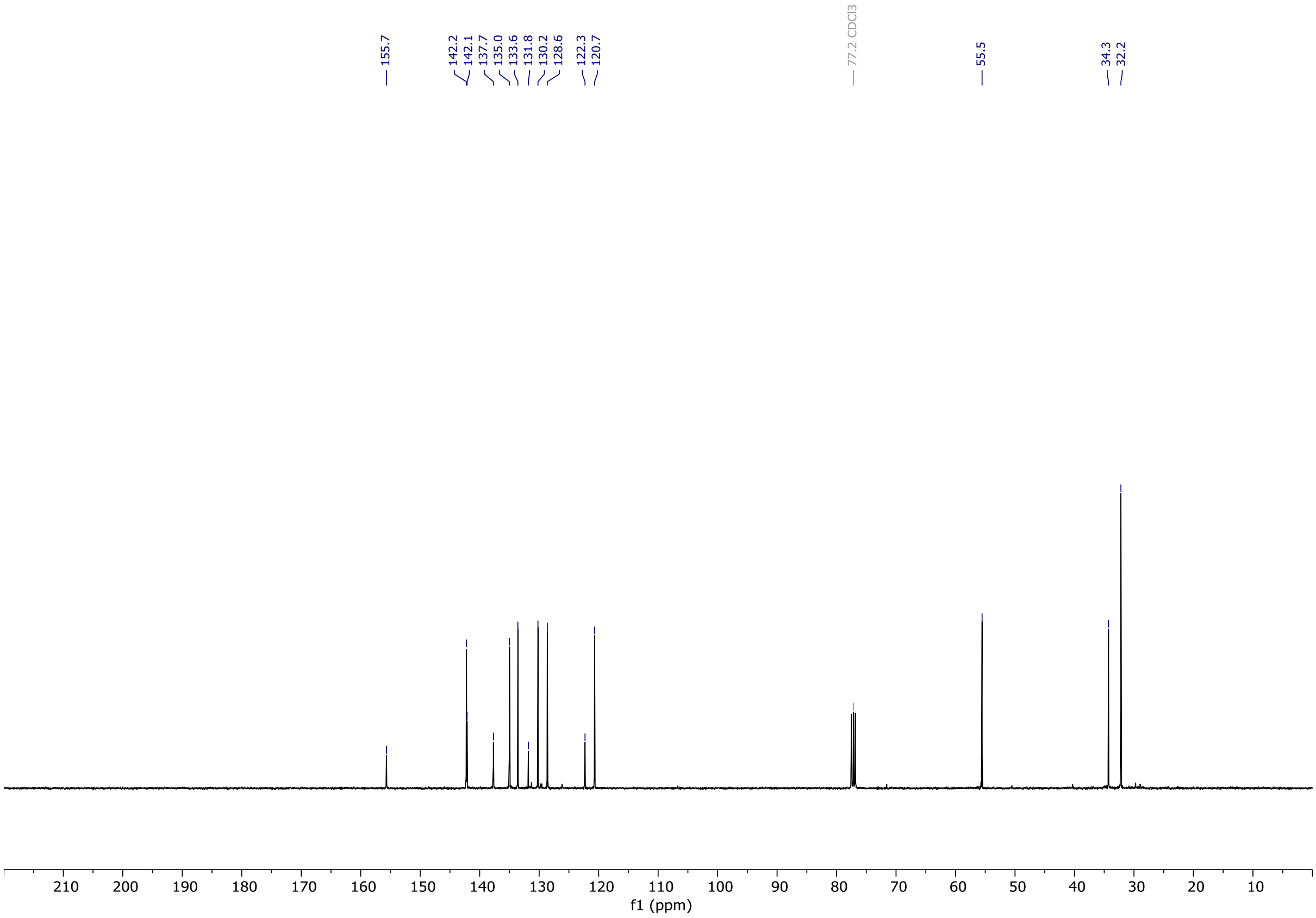

OX04956d

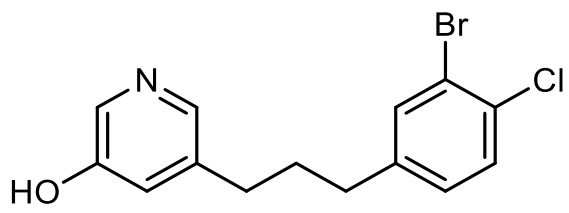

OX04956d

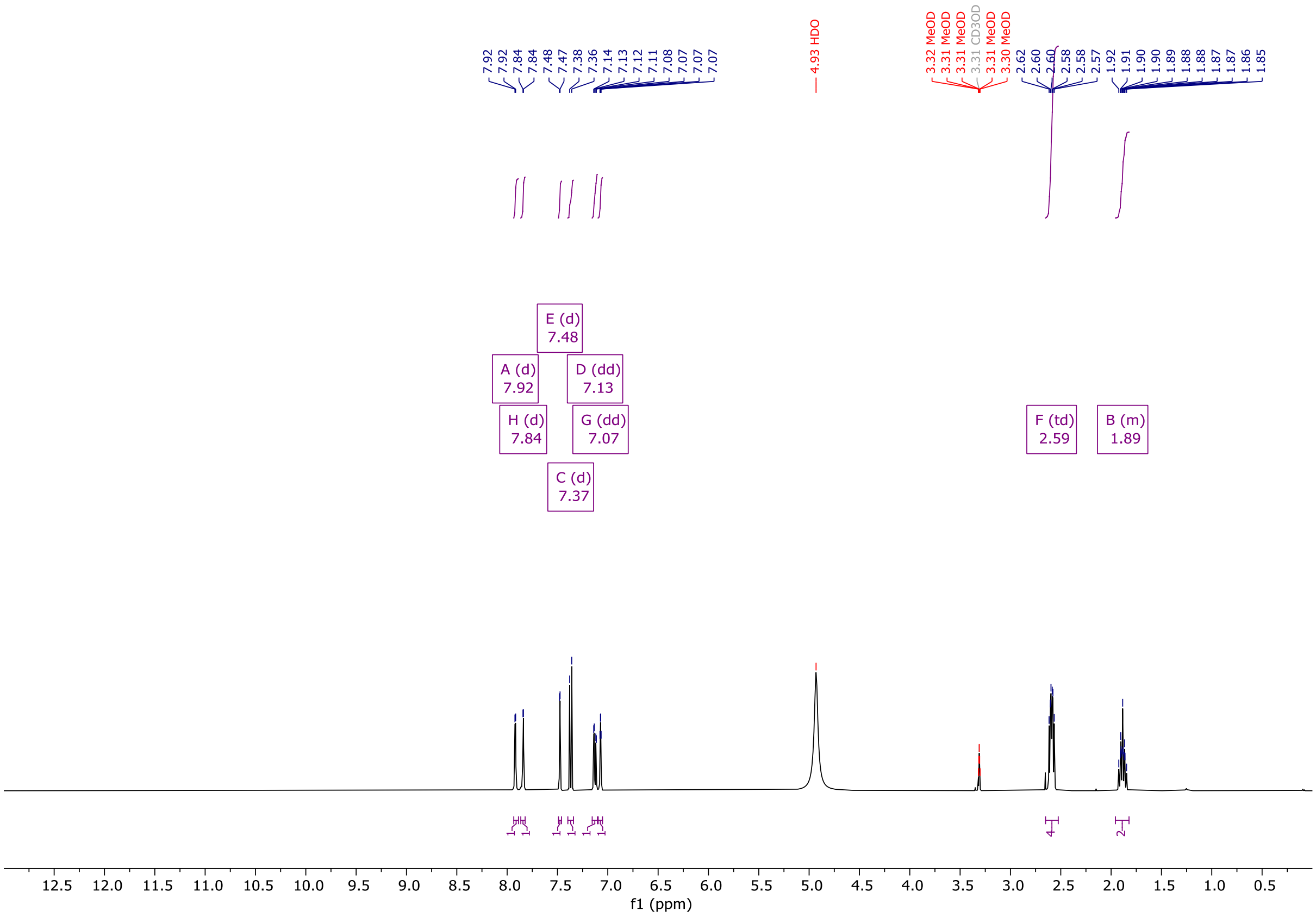

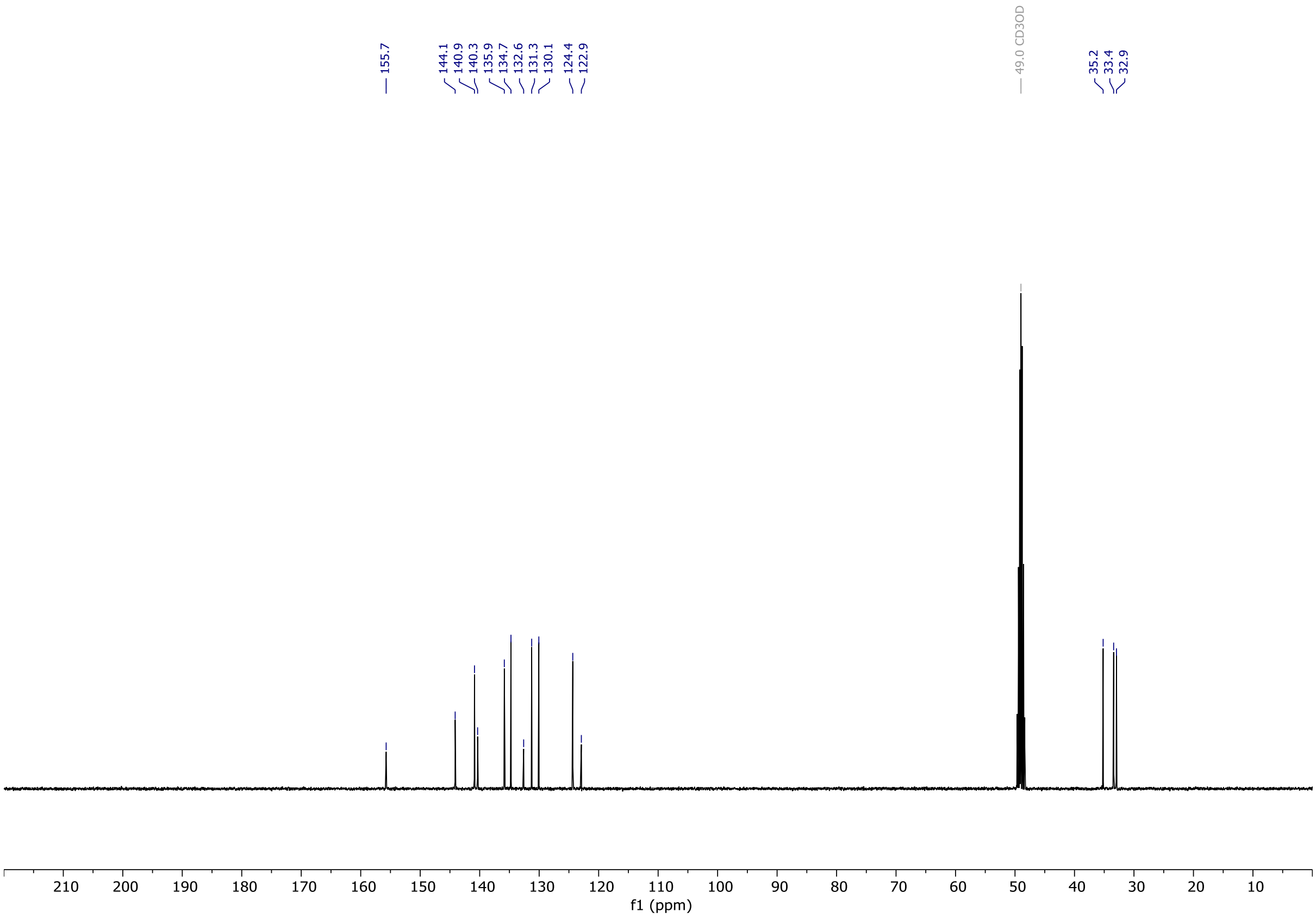

OX04956

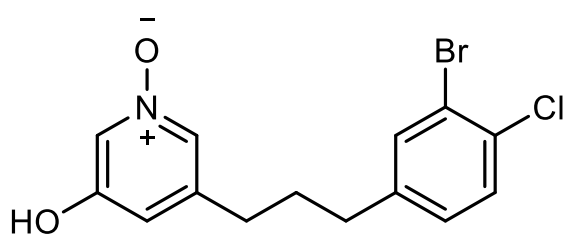

OX04956

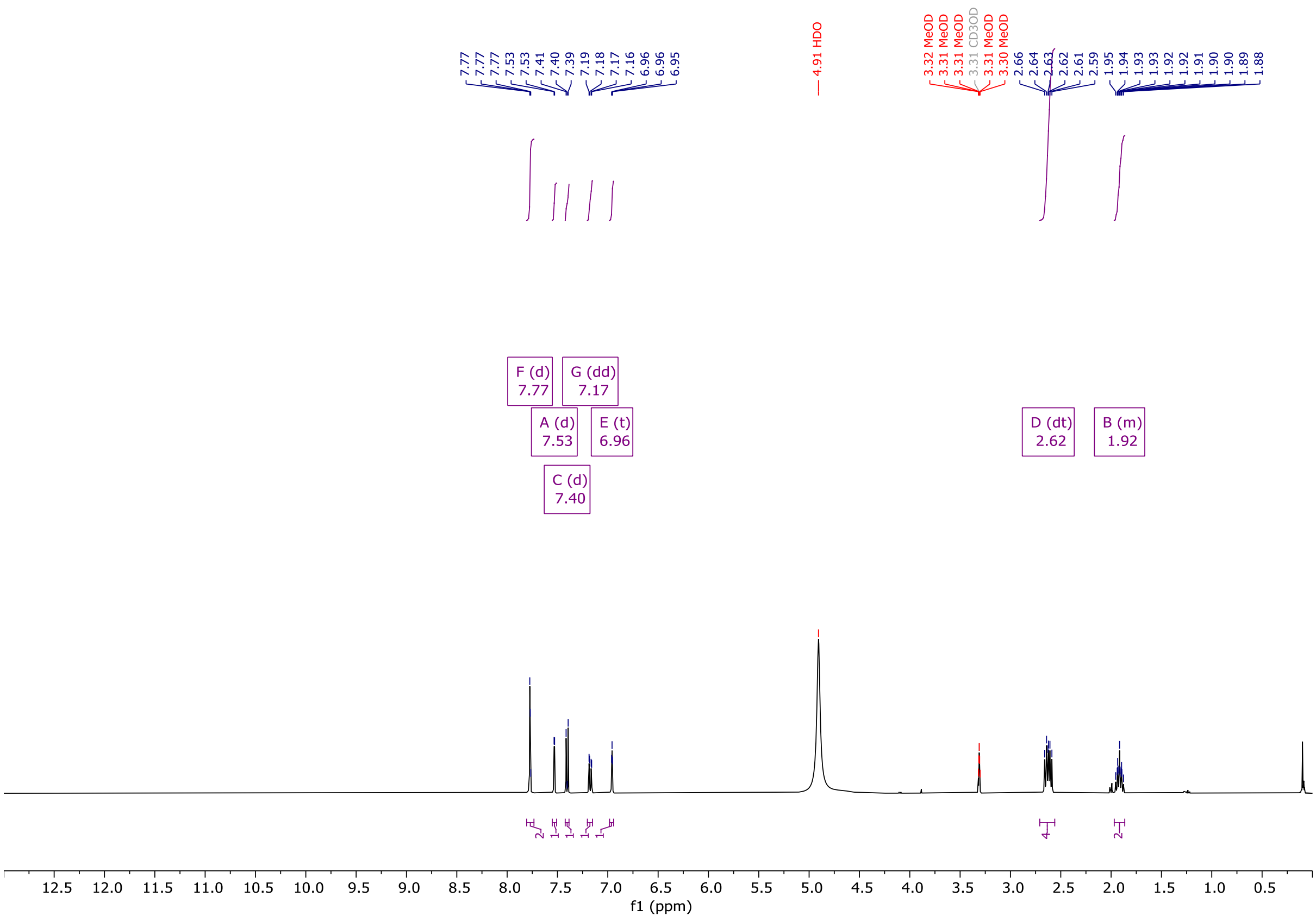

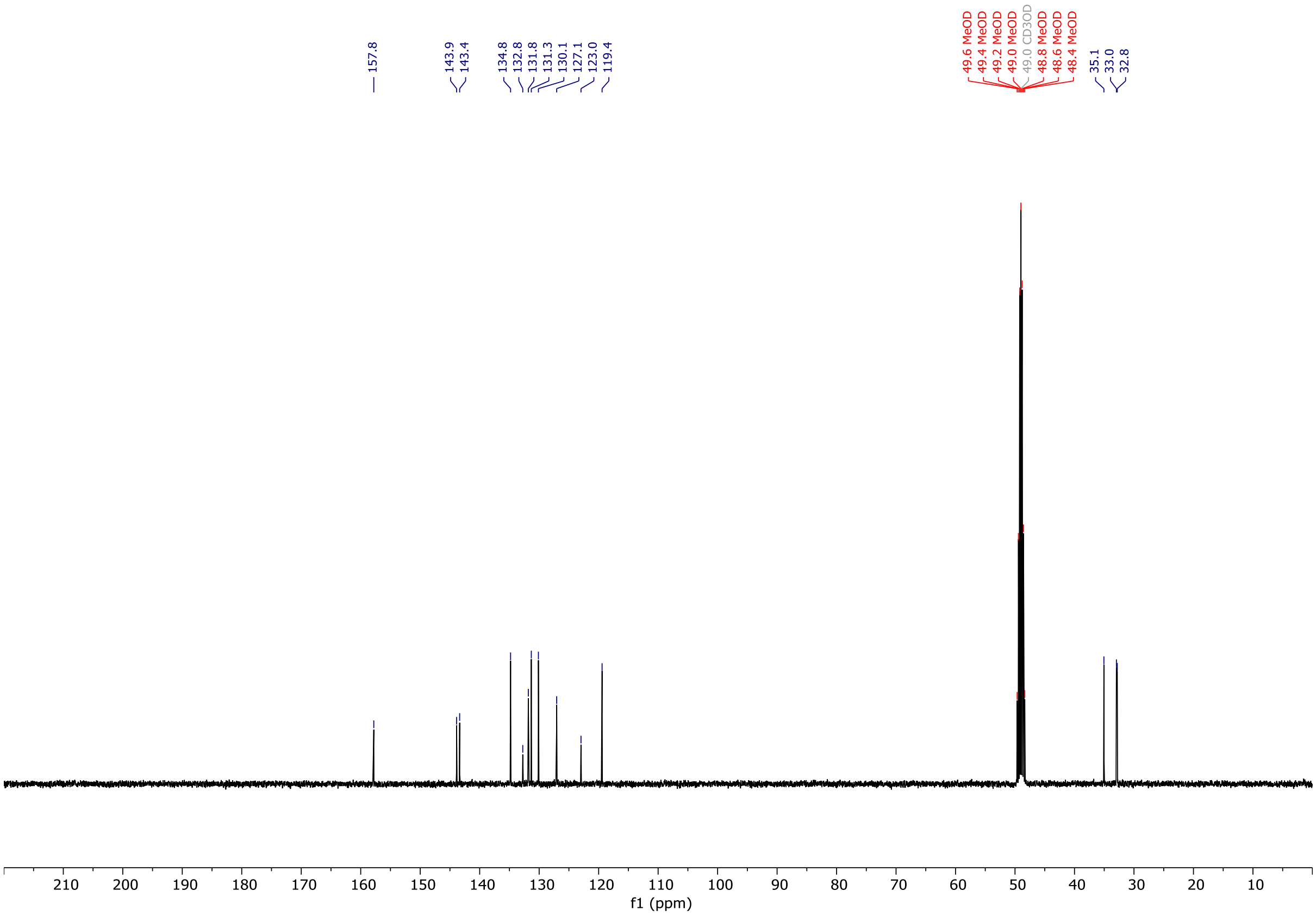

OX04955a

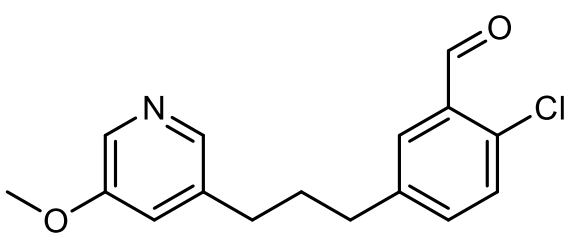

OX04955a

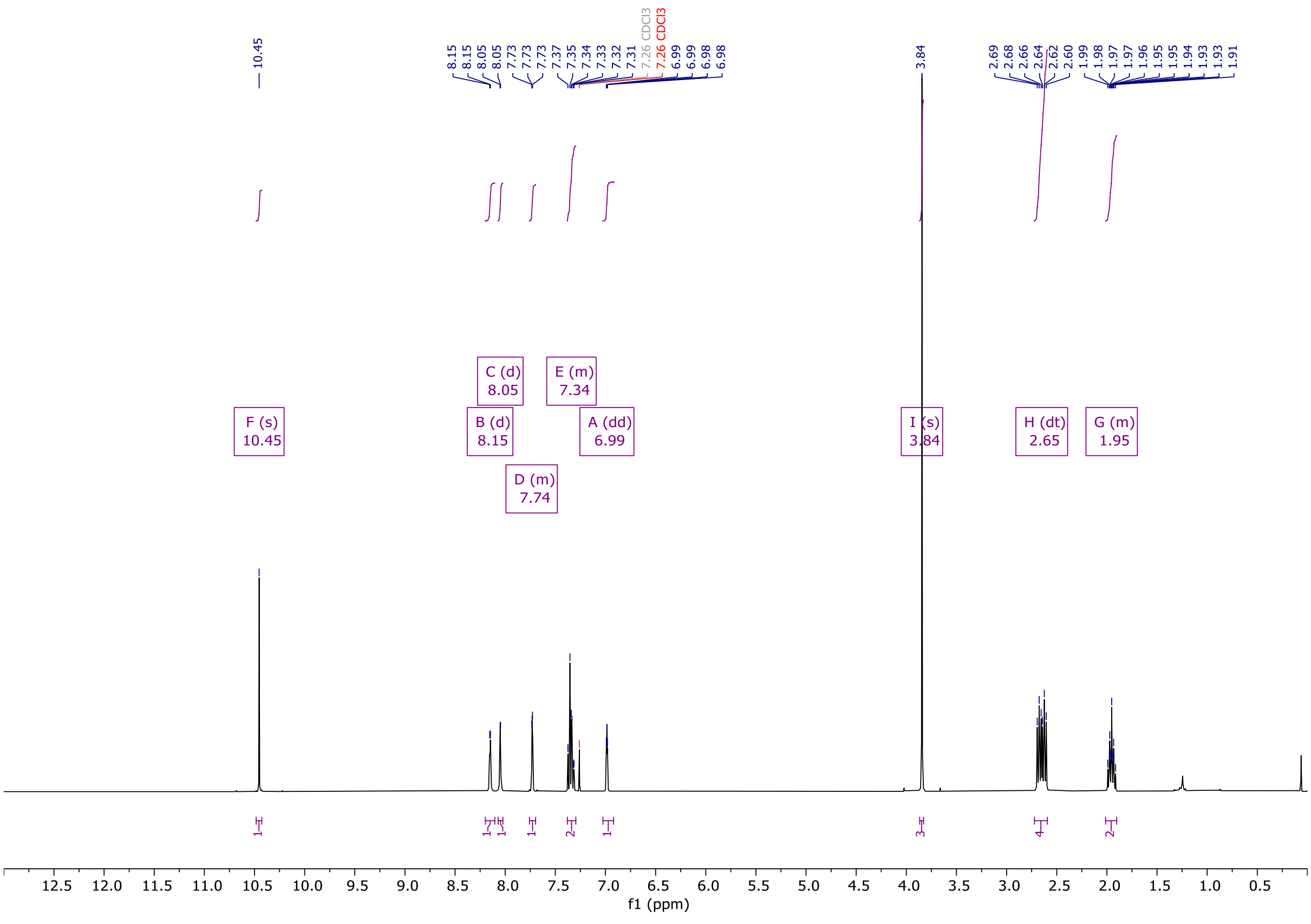

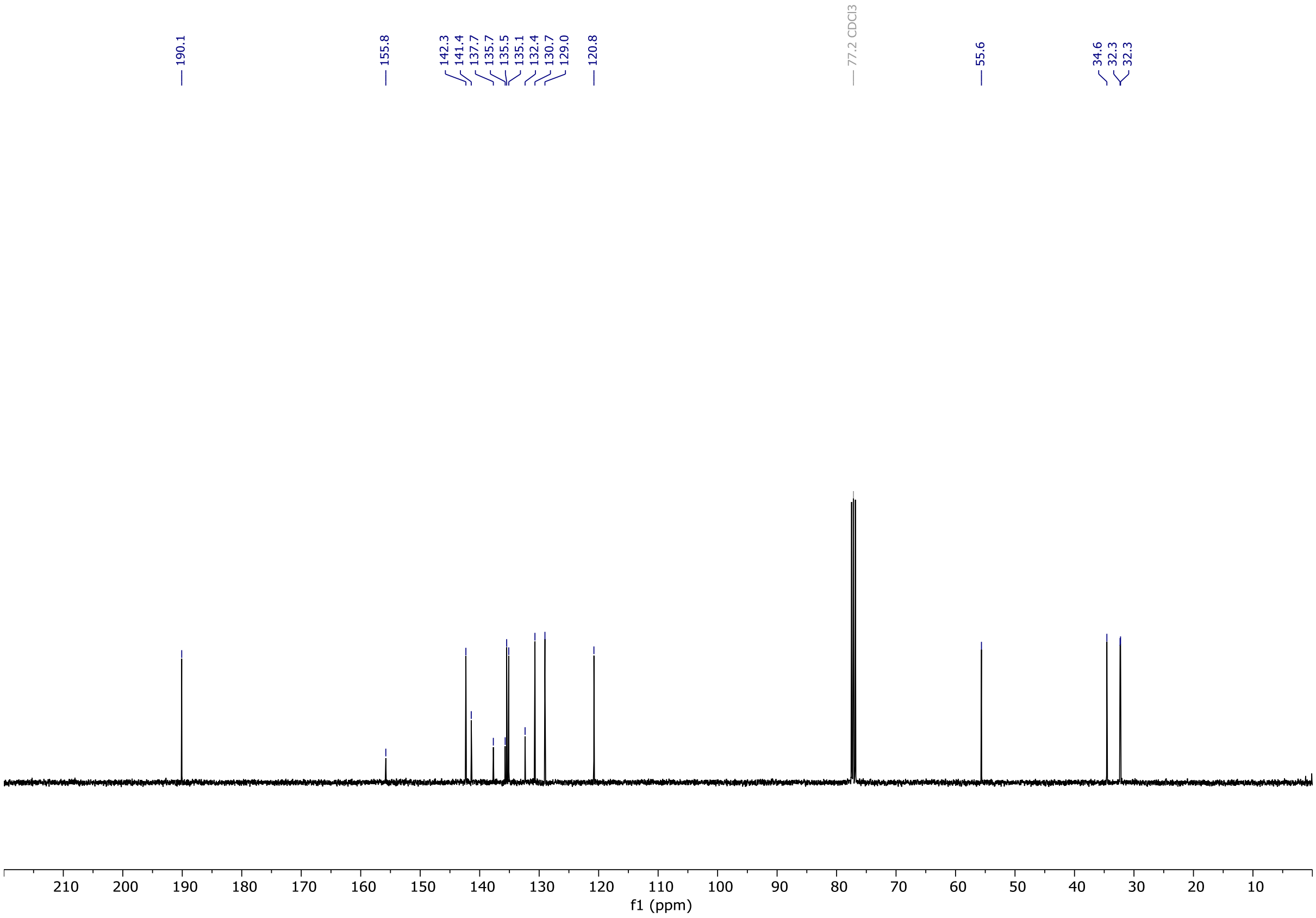

OX04955b

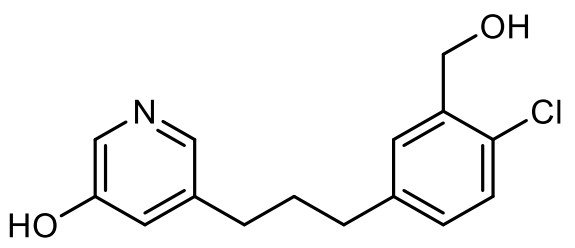

OX04955b

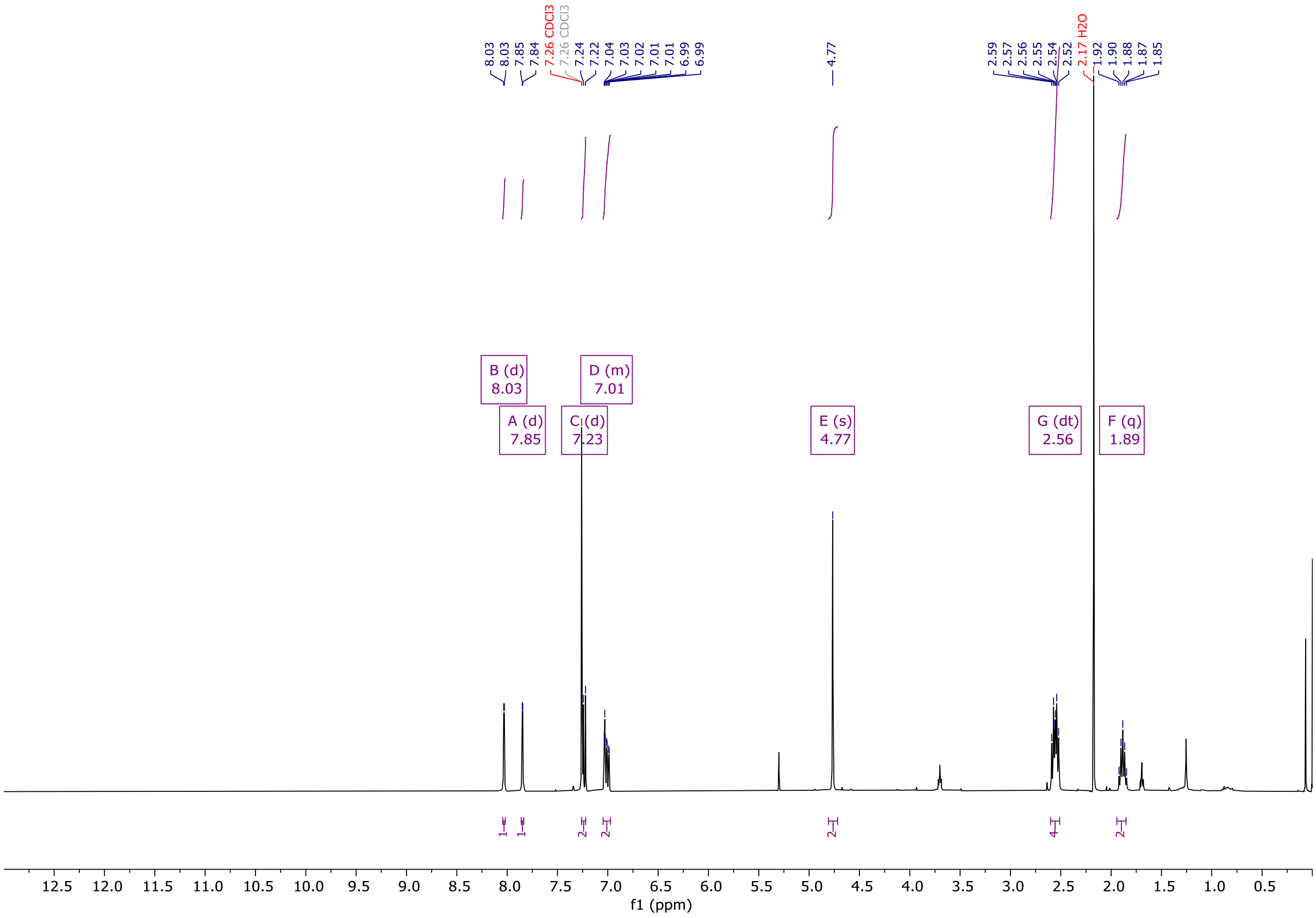

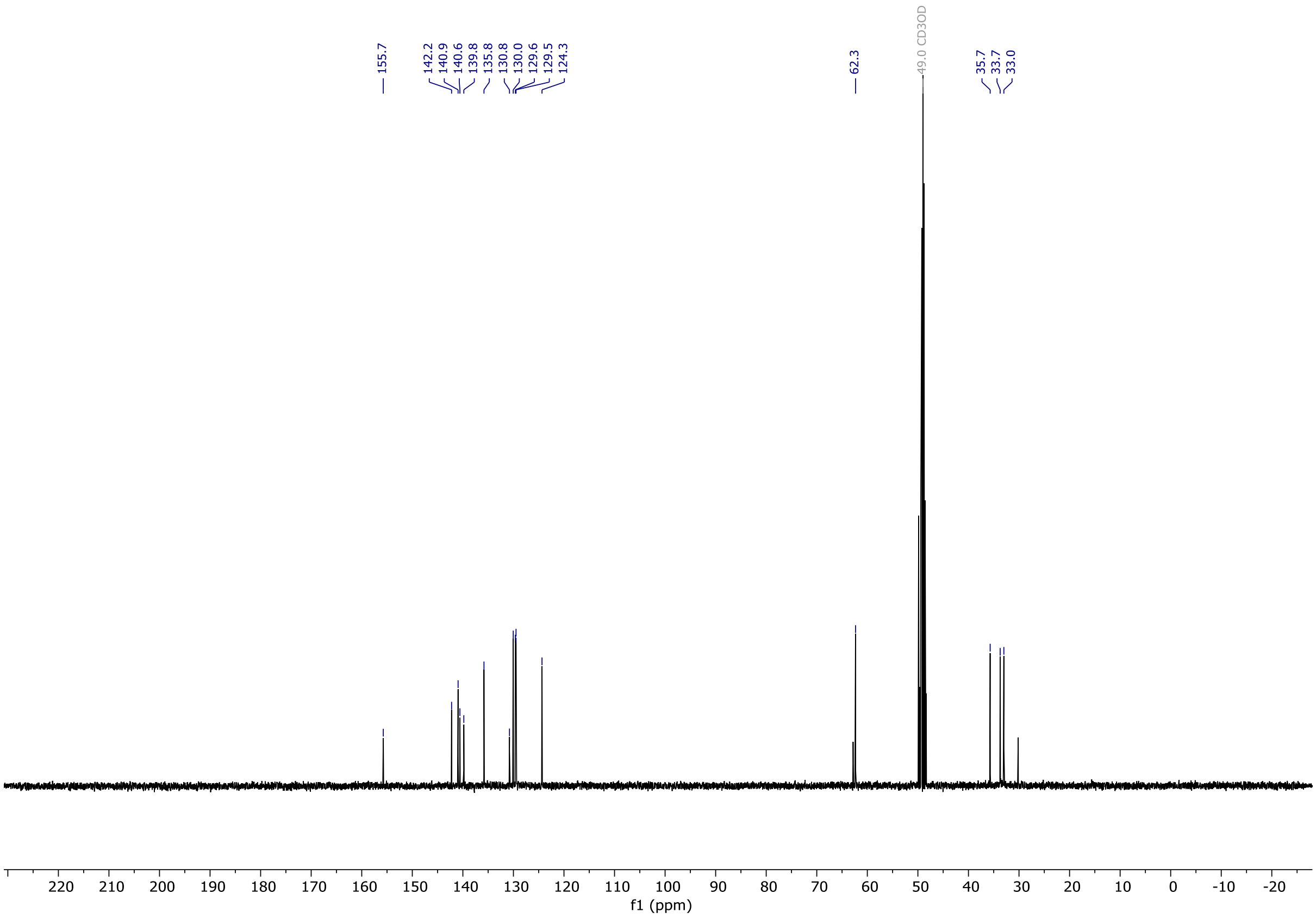

OX04955

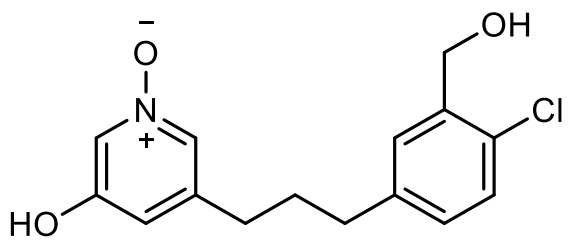

OX04955

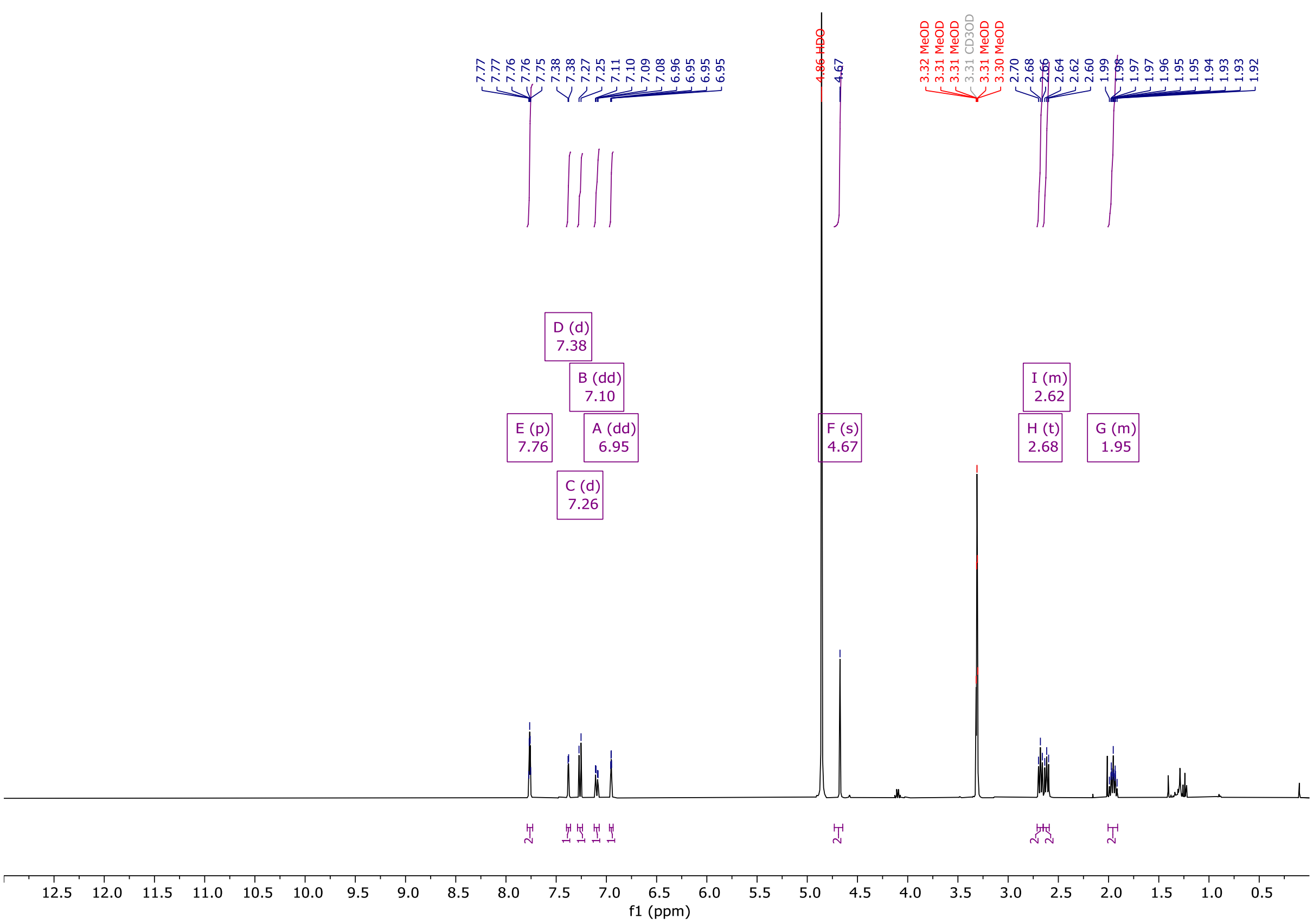

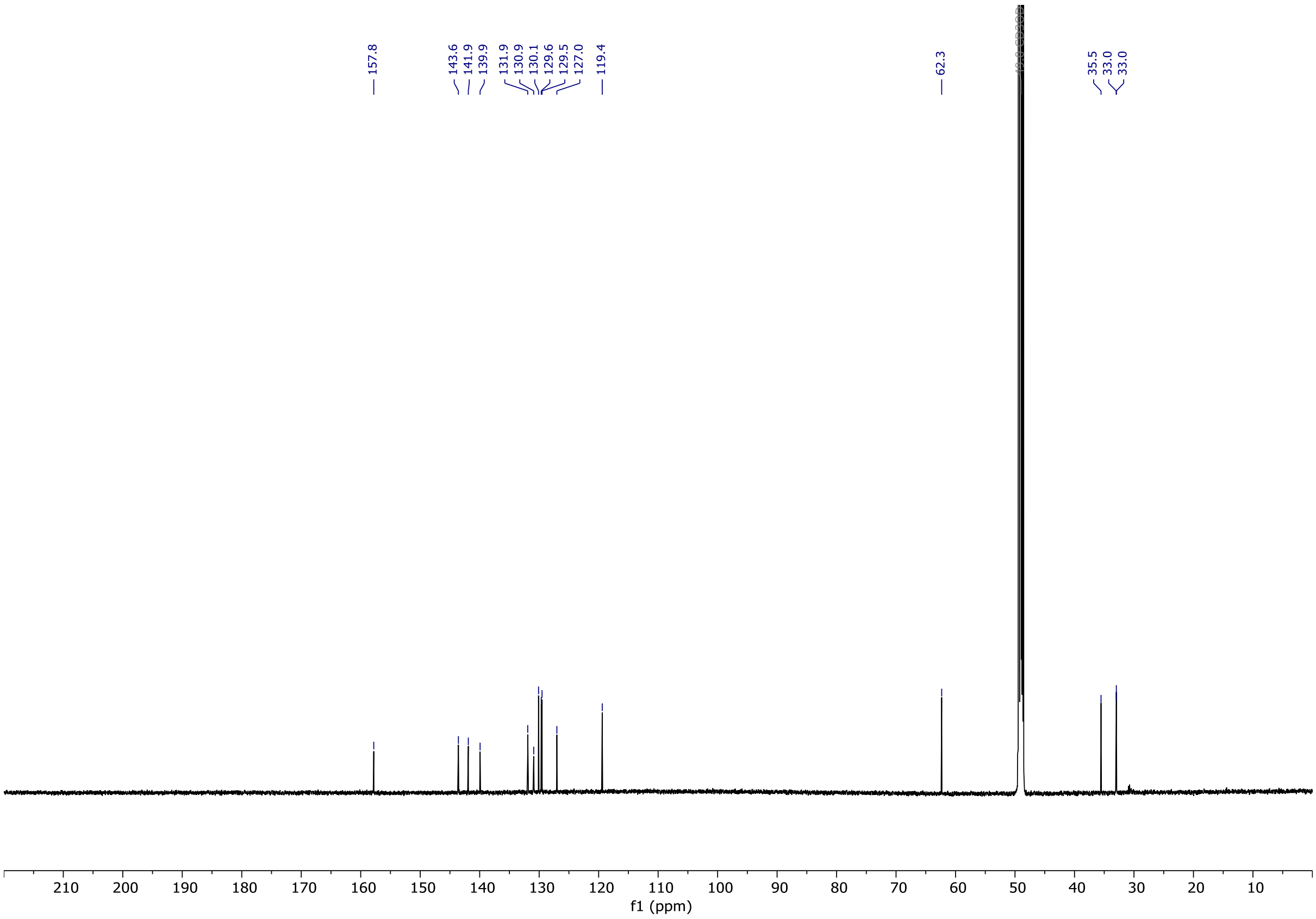

OX04957a

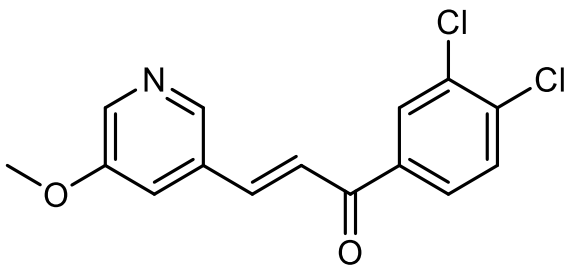

OX04957a

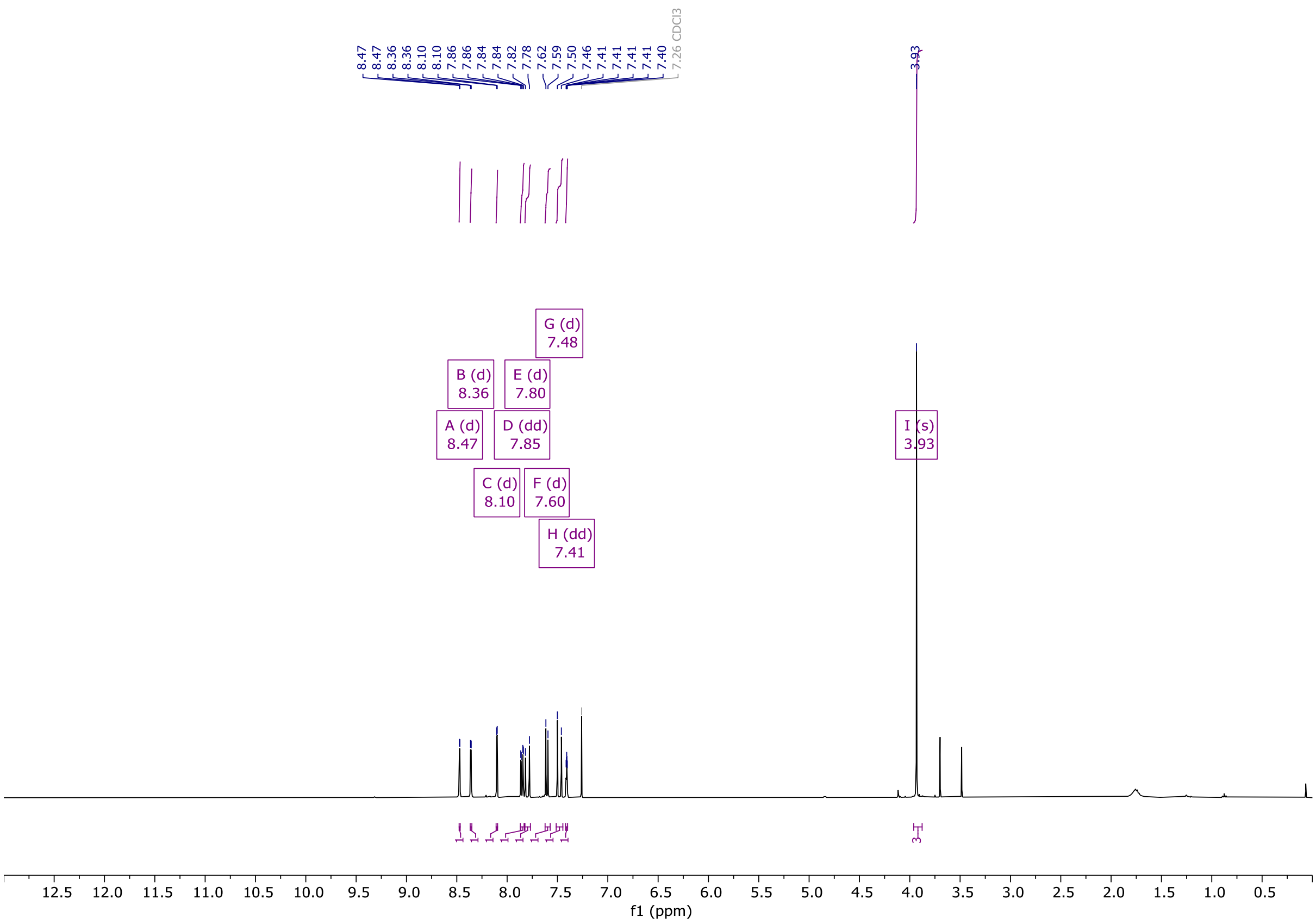

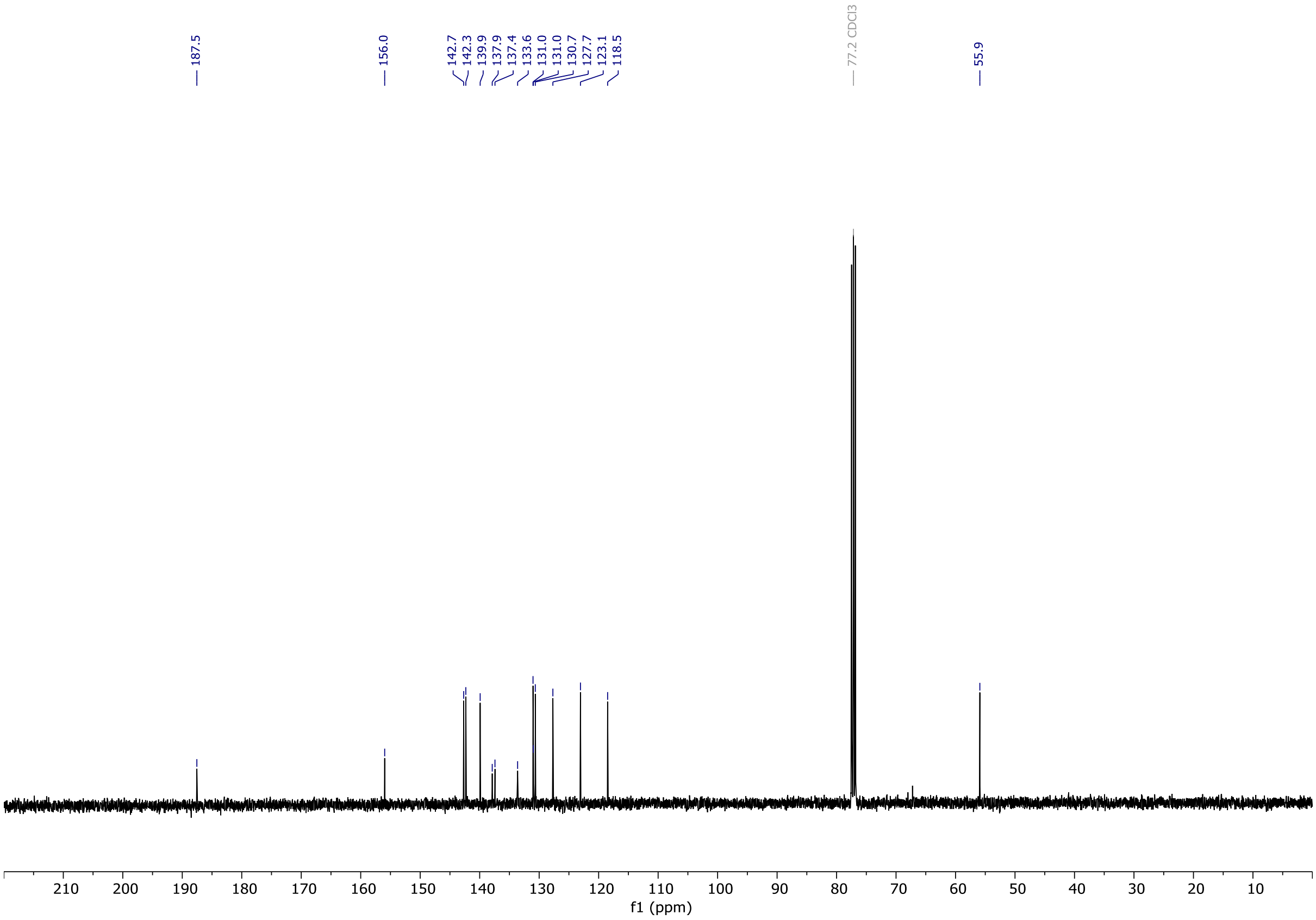

OX04957b

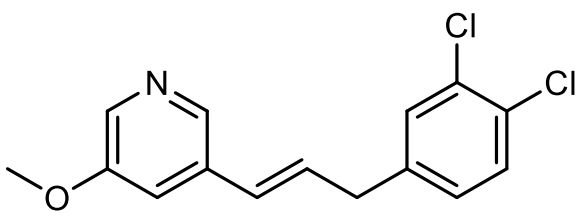

OX04957b

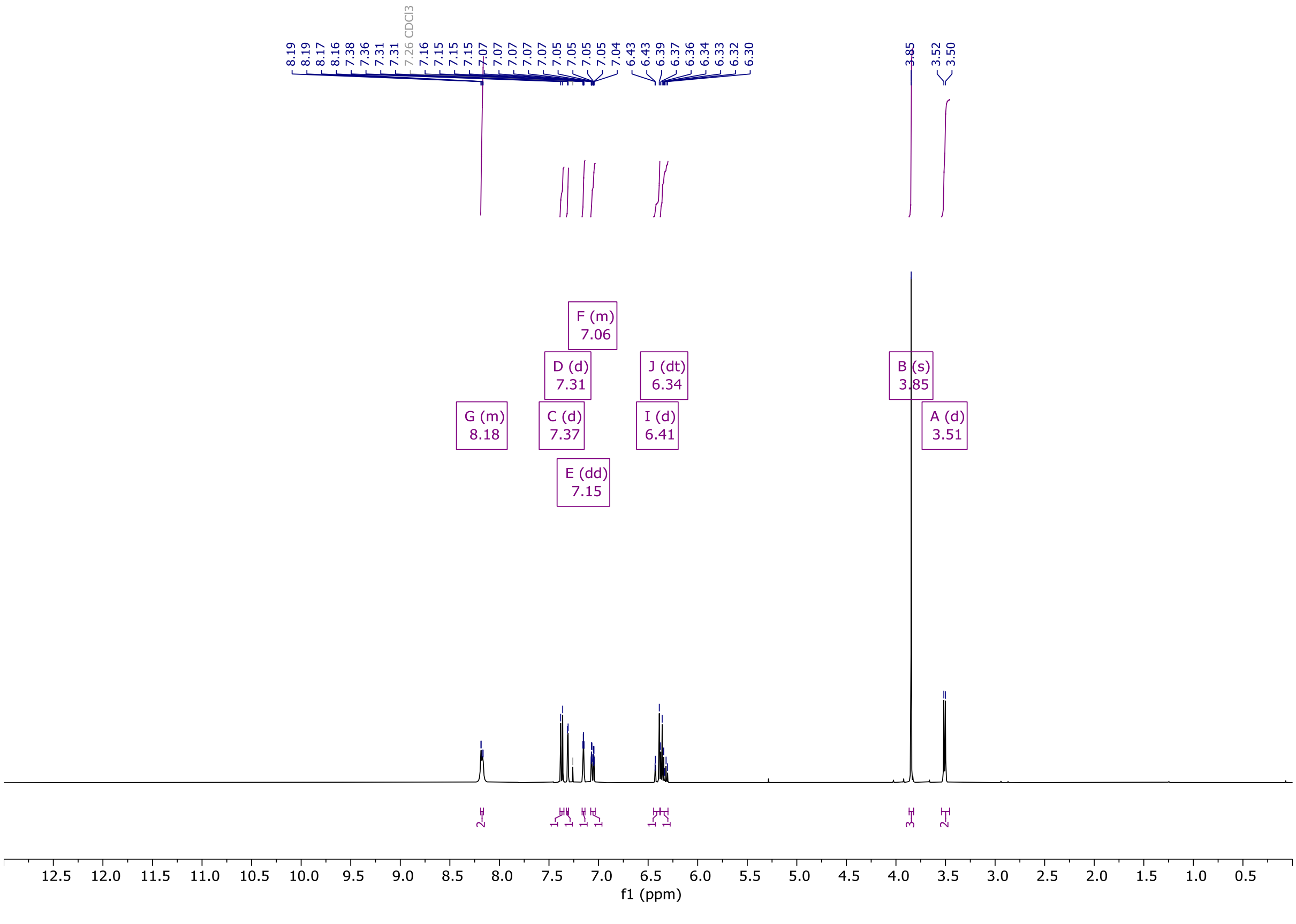

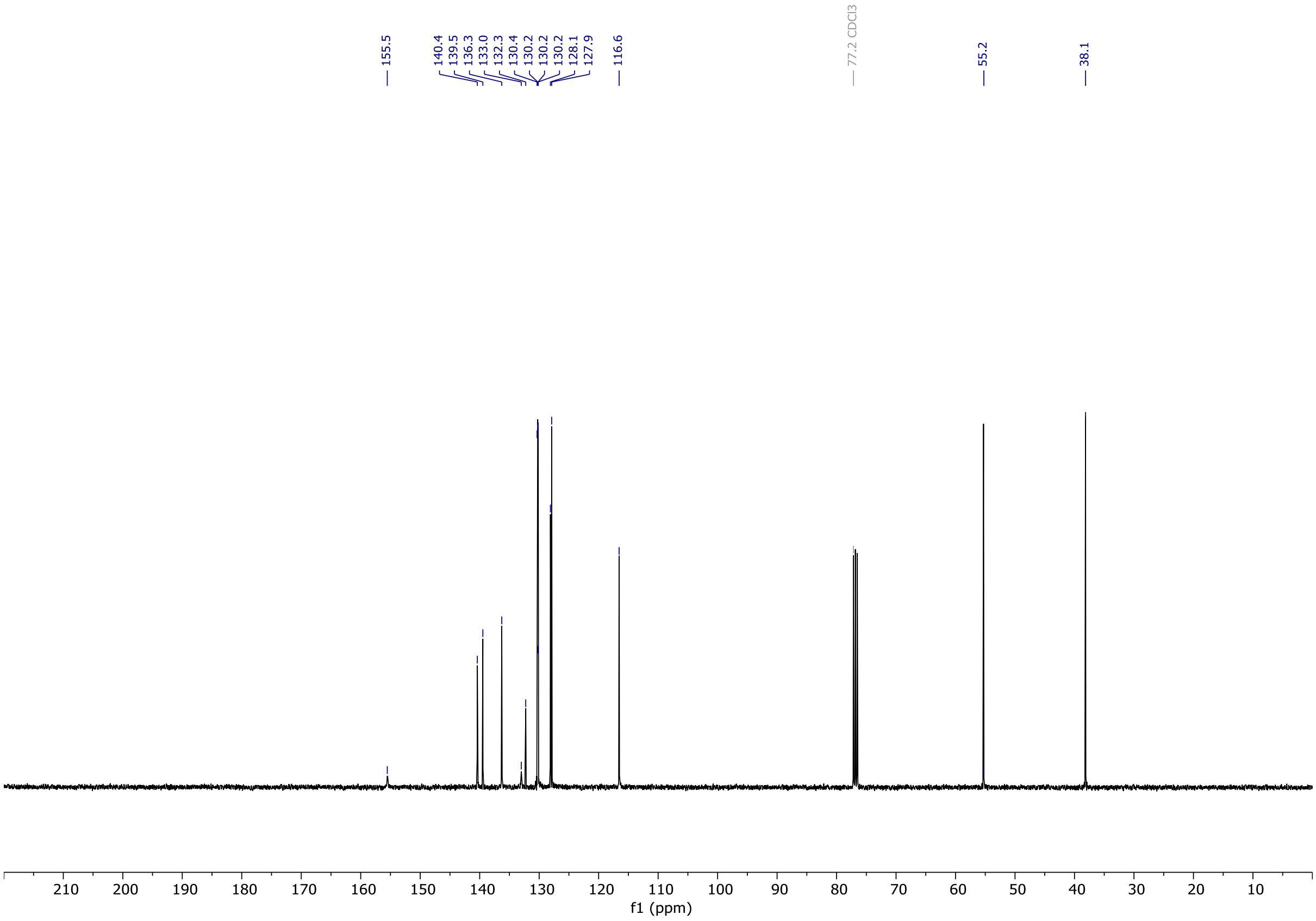

OX04957c

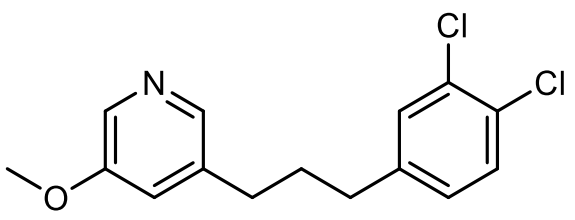

OX04957c

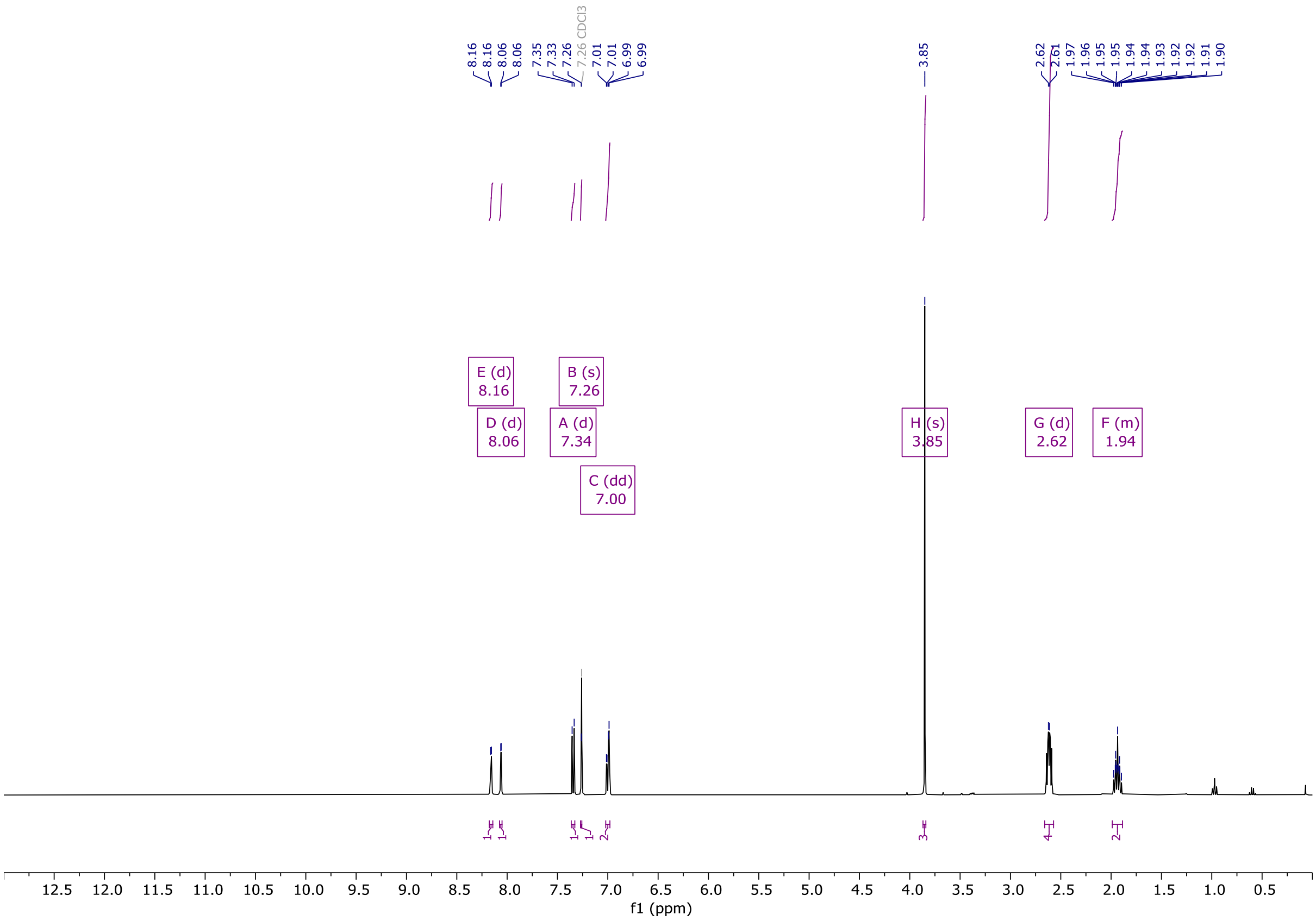

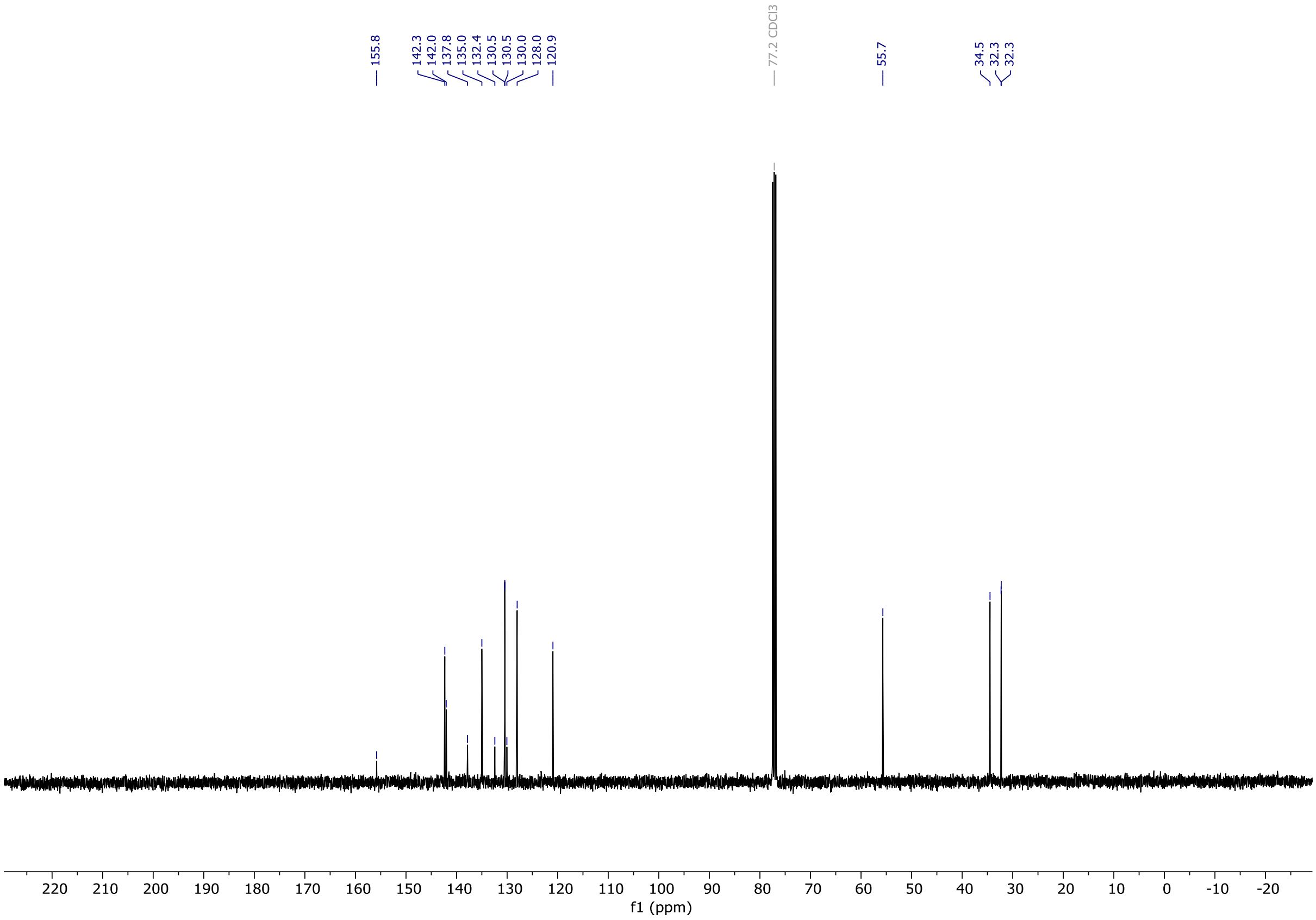

OX04957d

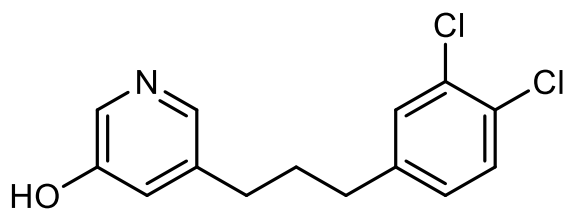

OX04957d

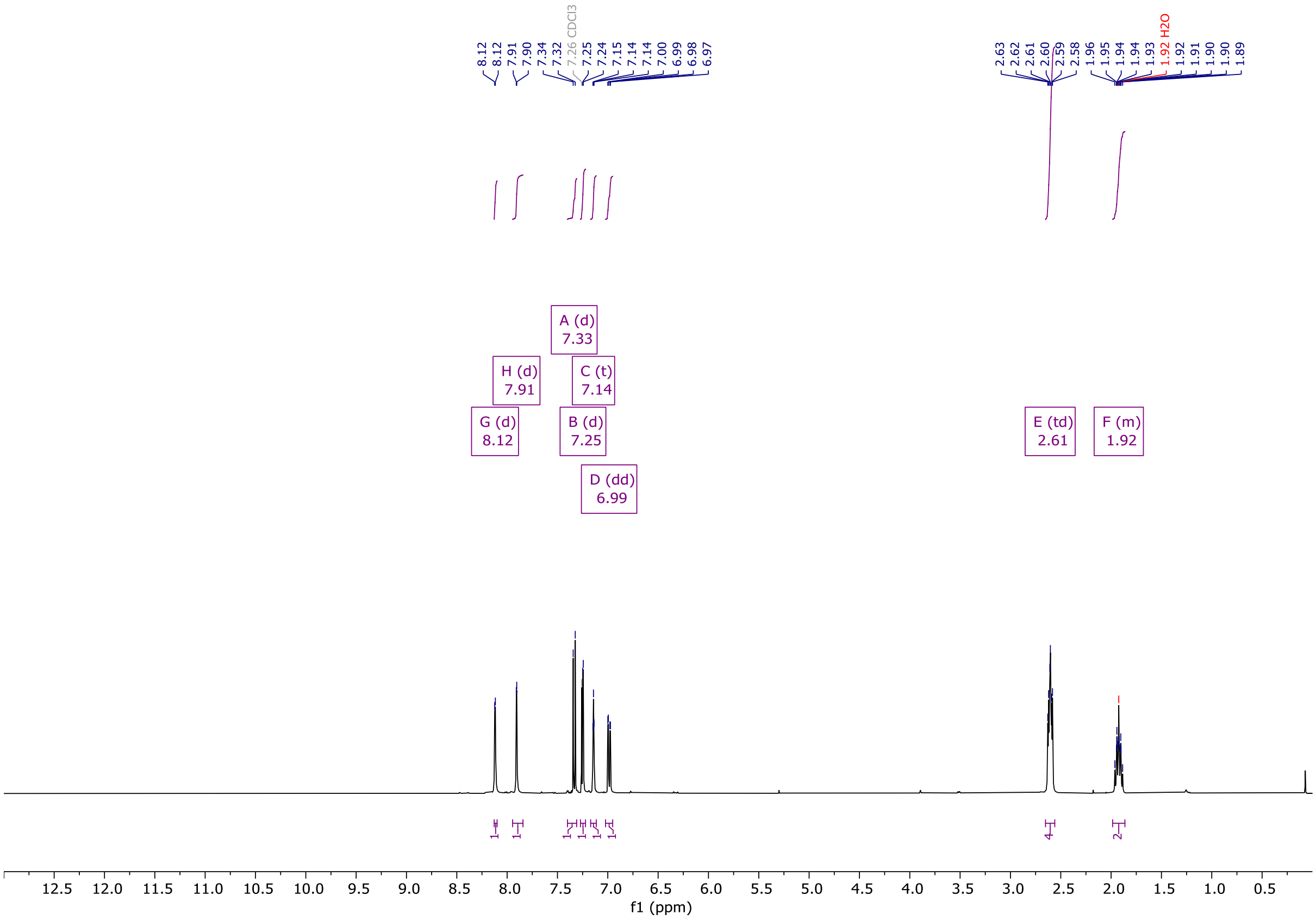

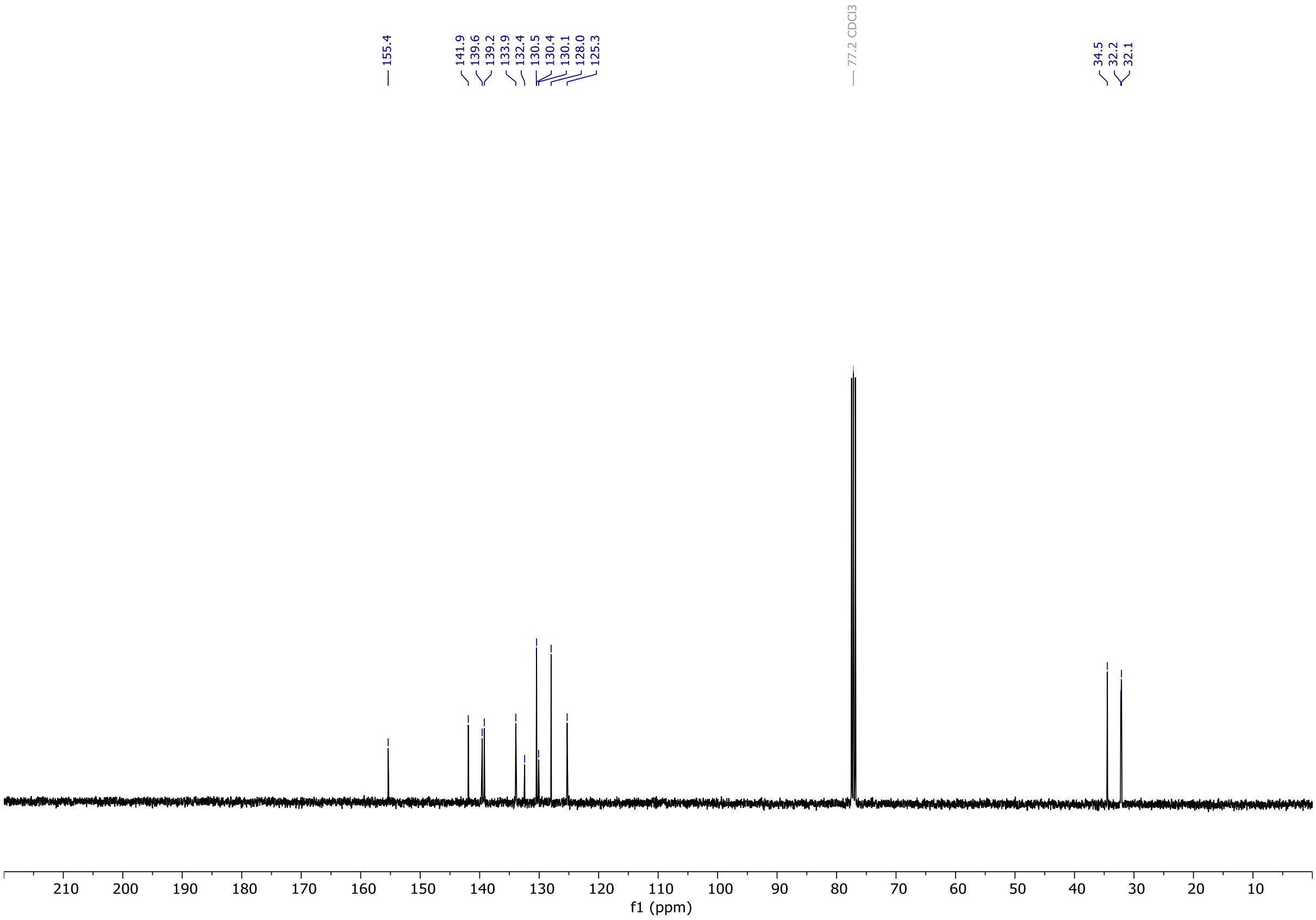

OX04957

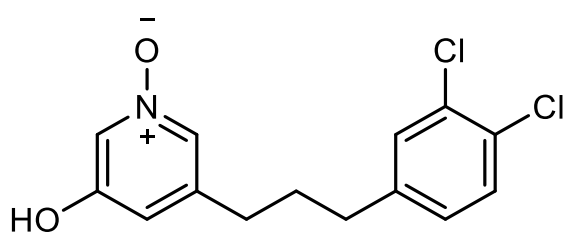

OX04957

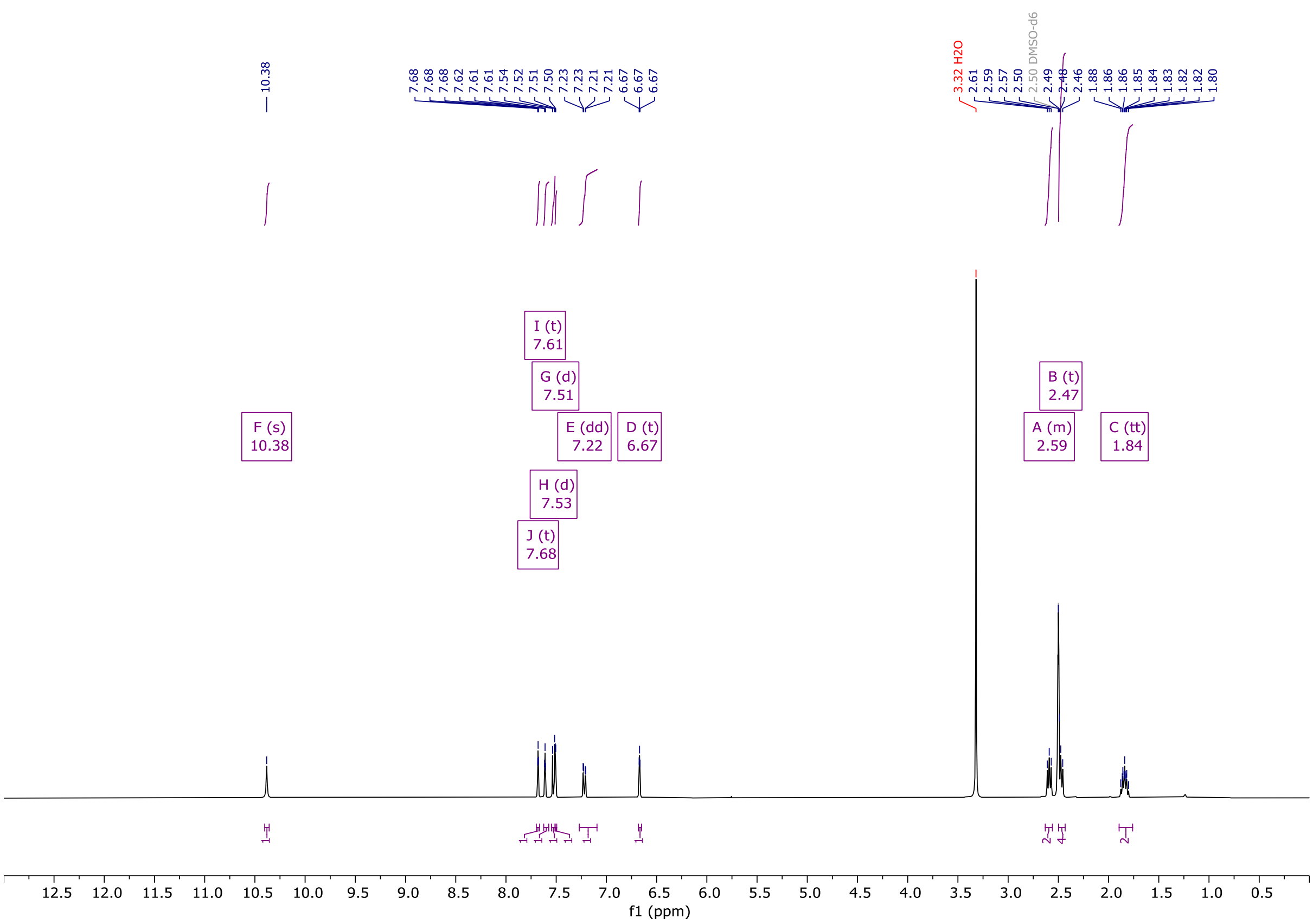

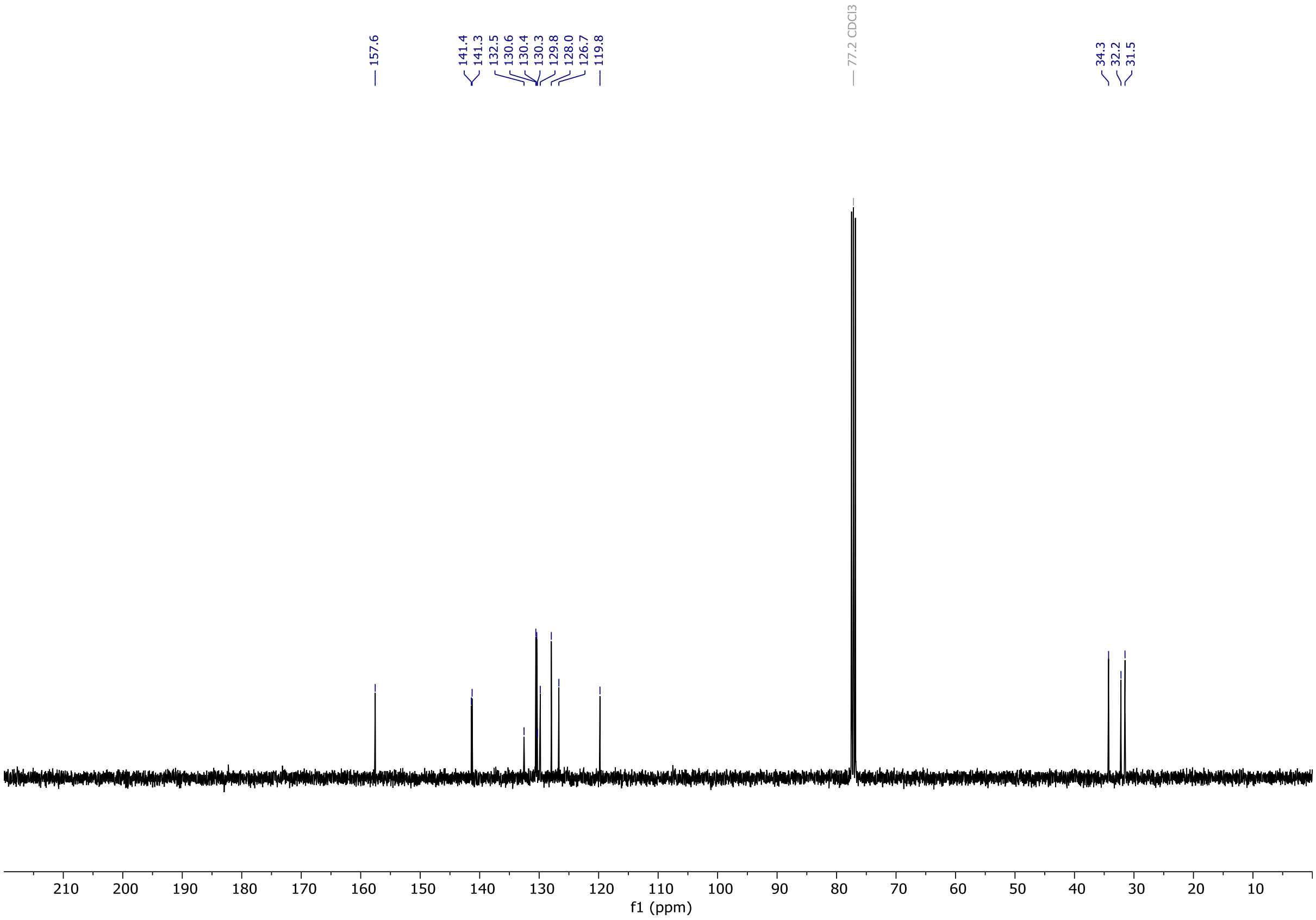

OX04958a

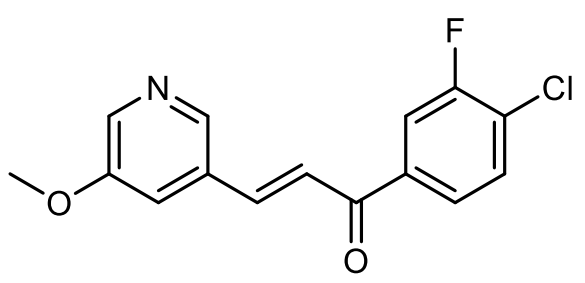

OX04958a

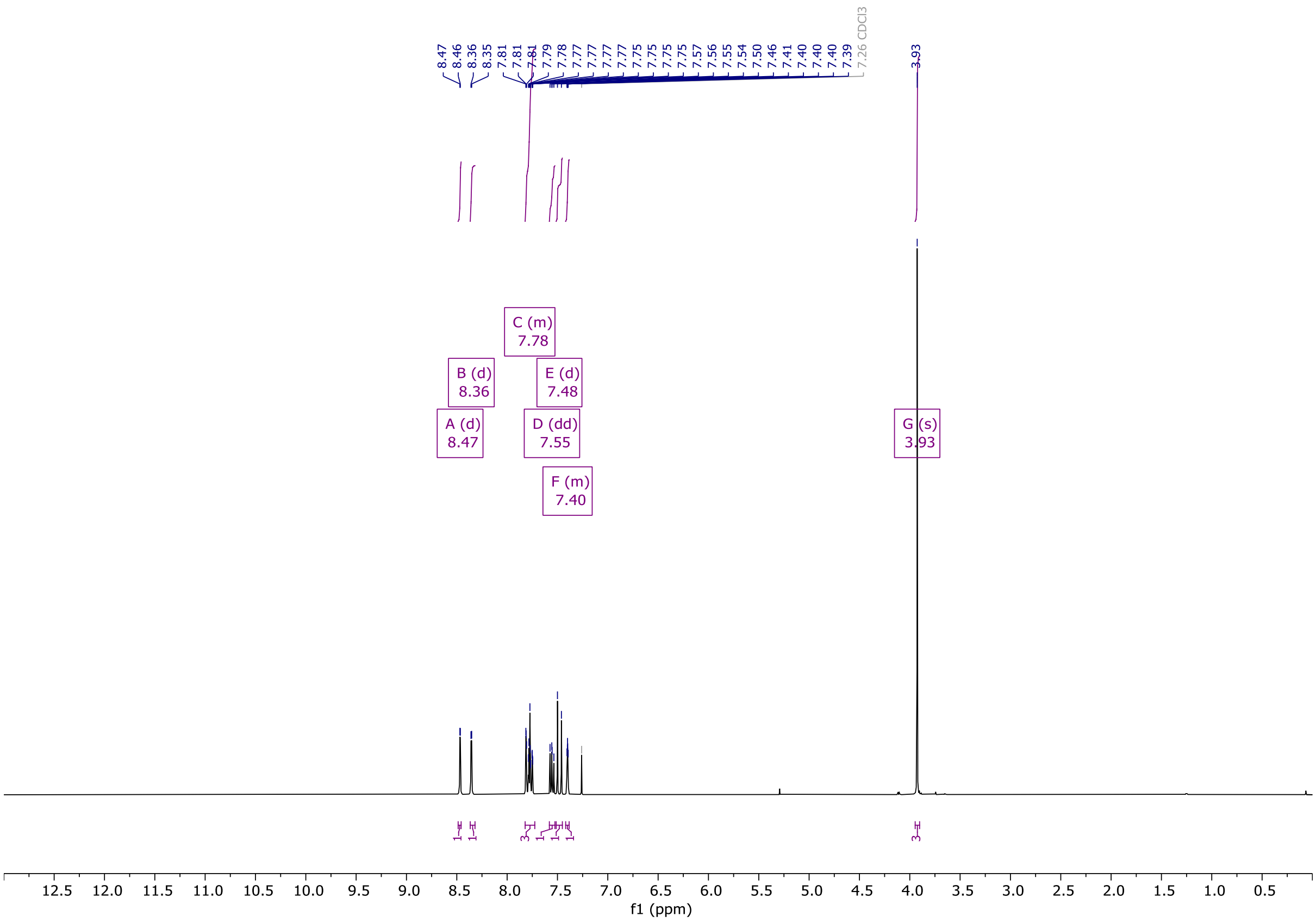

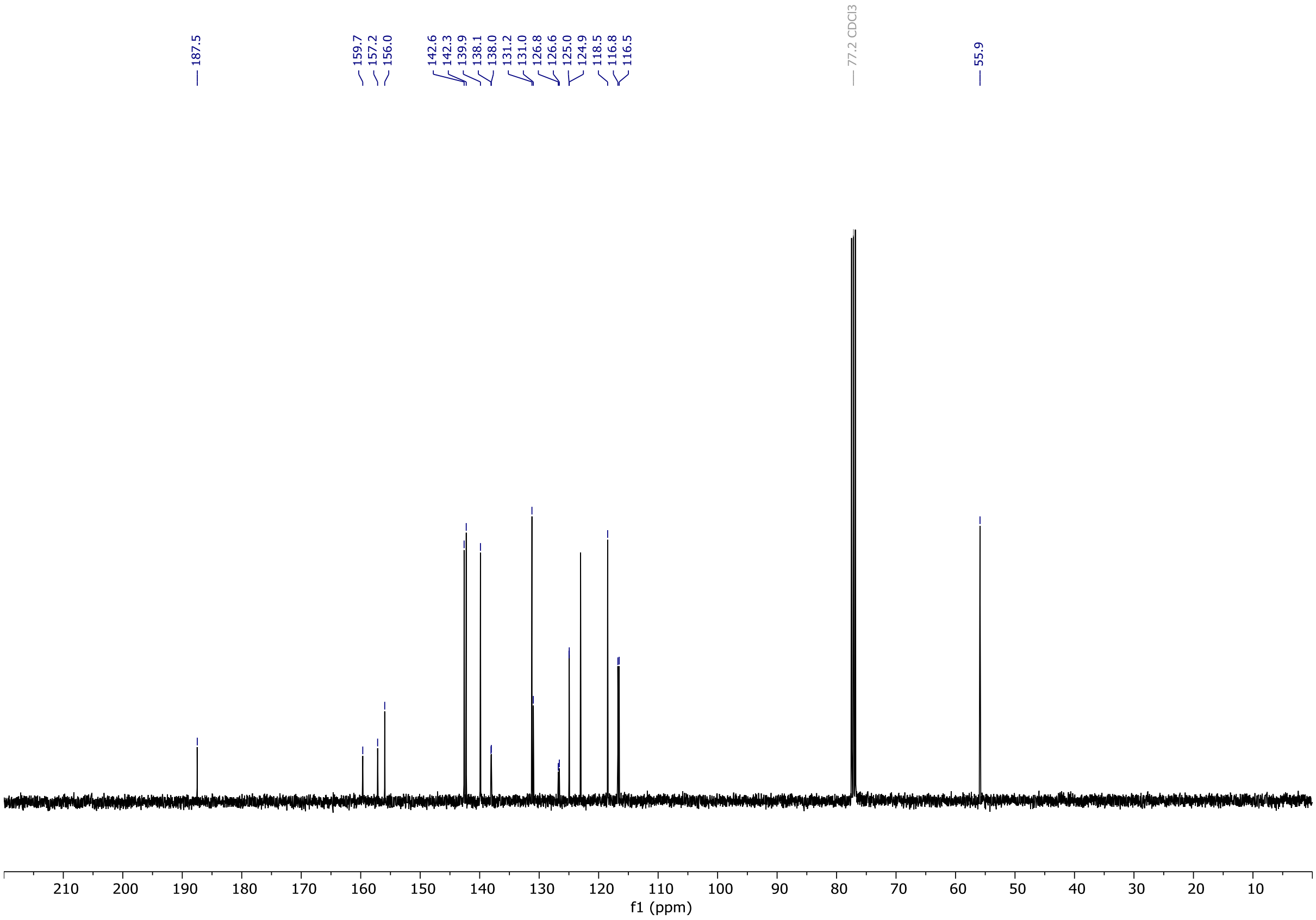

OX04958b

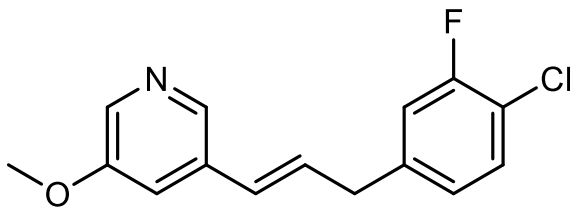

OX04958b

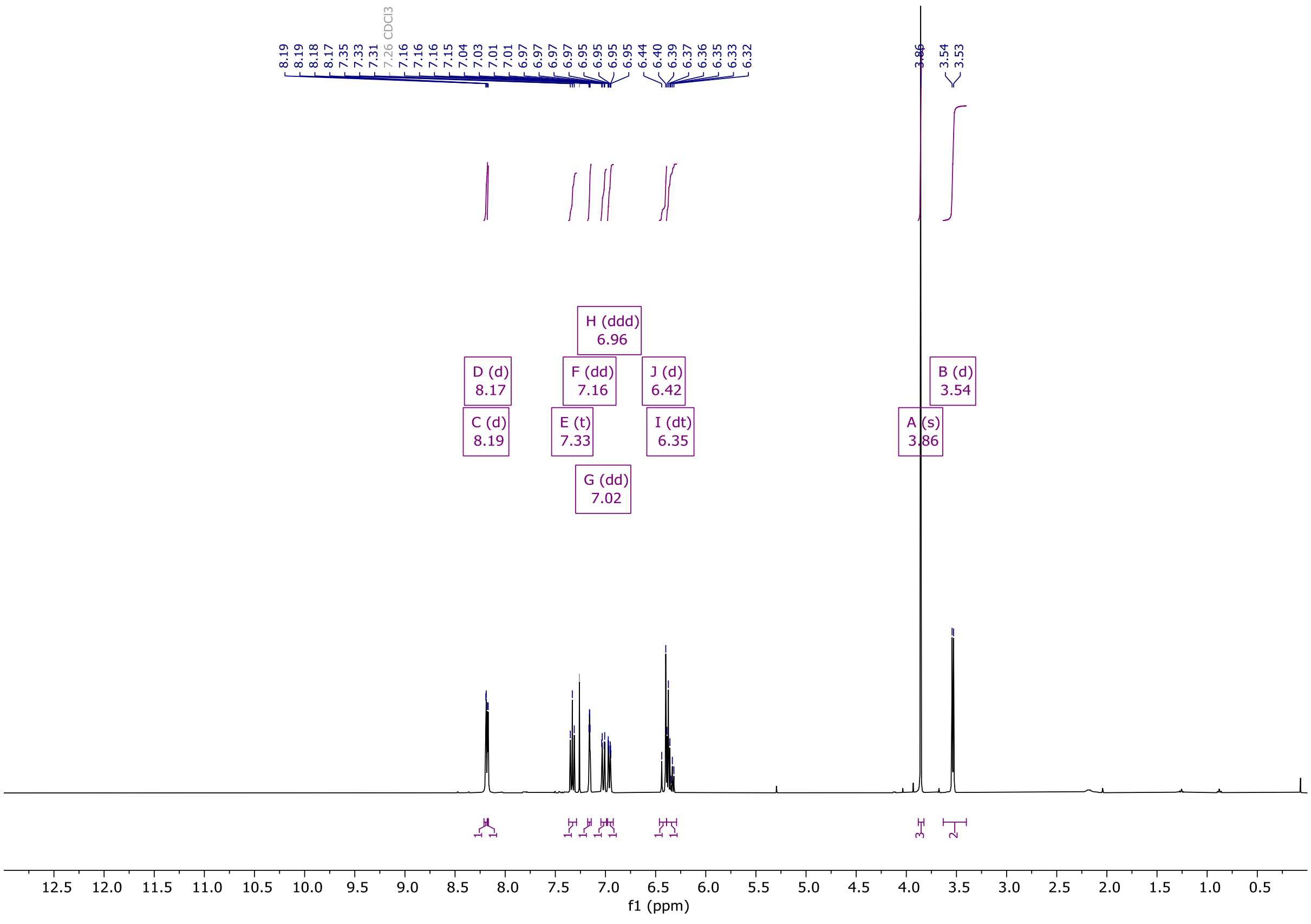

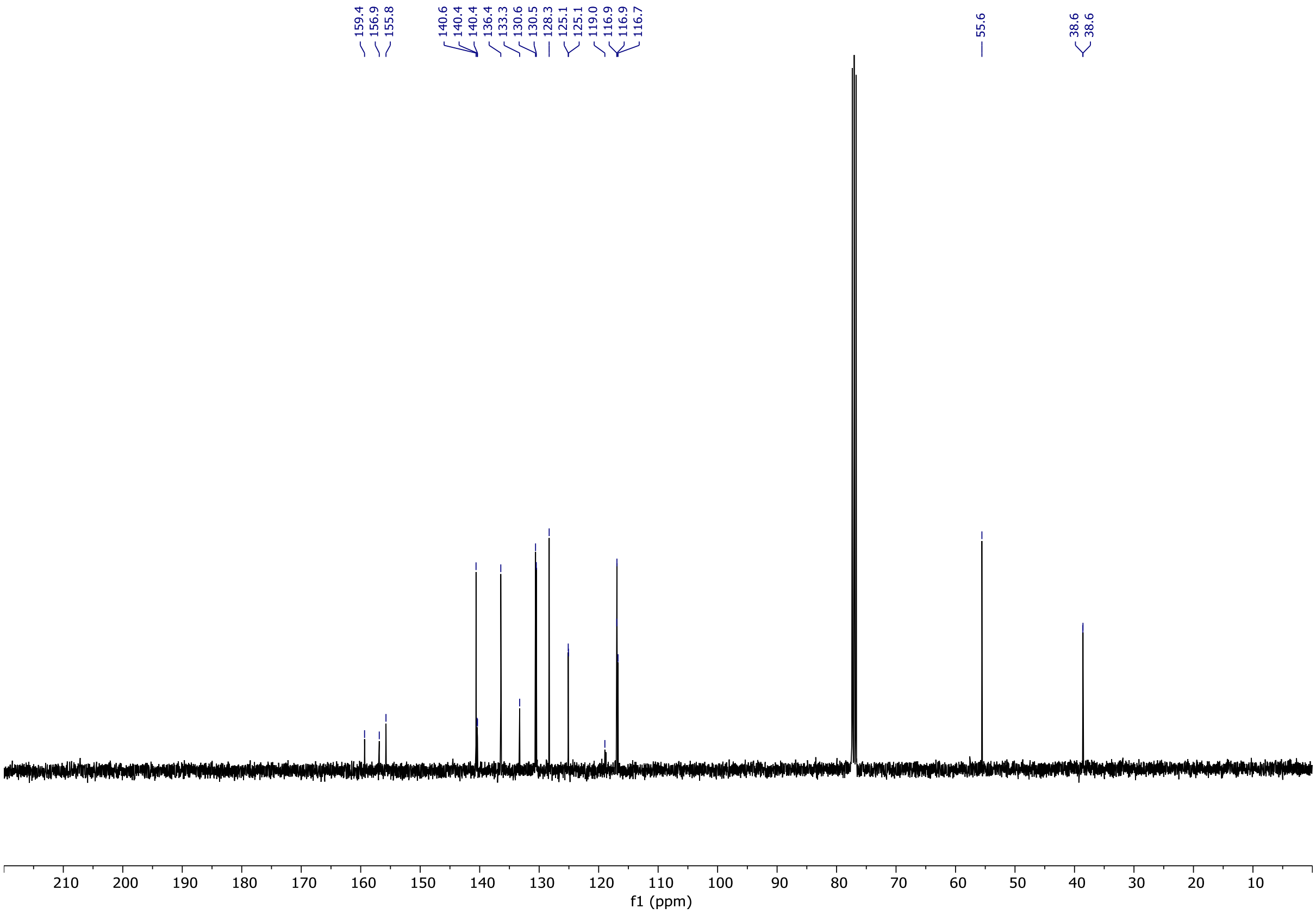
159.4
156.9
155.8
140.6
140.4
140.4
136.4
133.3
130.6
130.5
128.3
125.1
125.1
119.0
116.9
116.9
116.7
55.6
38.6
38.6
210 200 190 180 170 160 150 140 130 120 110 100 90 80 70 60 50 40 30 20 10
f1 (ppm)

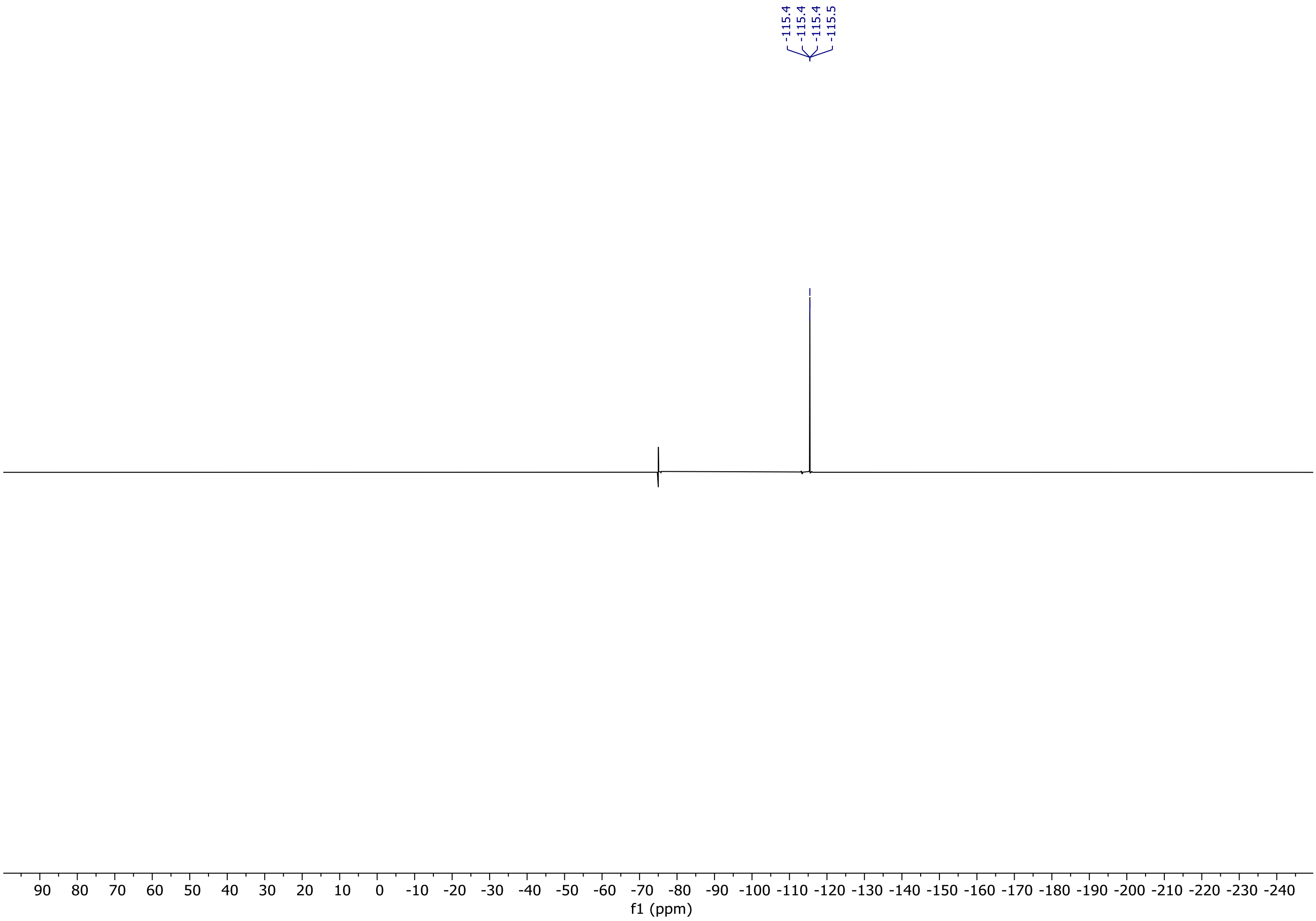
-115.4
-115.4
-115.4
-115.5
90 80 70 60 50 40 30 20 10 0 -10 -20 -30 -40 -50 -60 -70 -80 -90 -100 -110 -120 -130 -140 -150 -160 -170 -180 -190 -200 -210 -220 -230 -240
f1 (ppm)

OX04958c

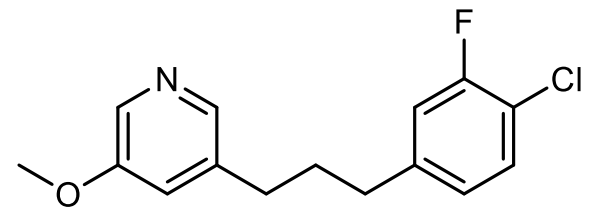

OX04958c

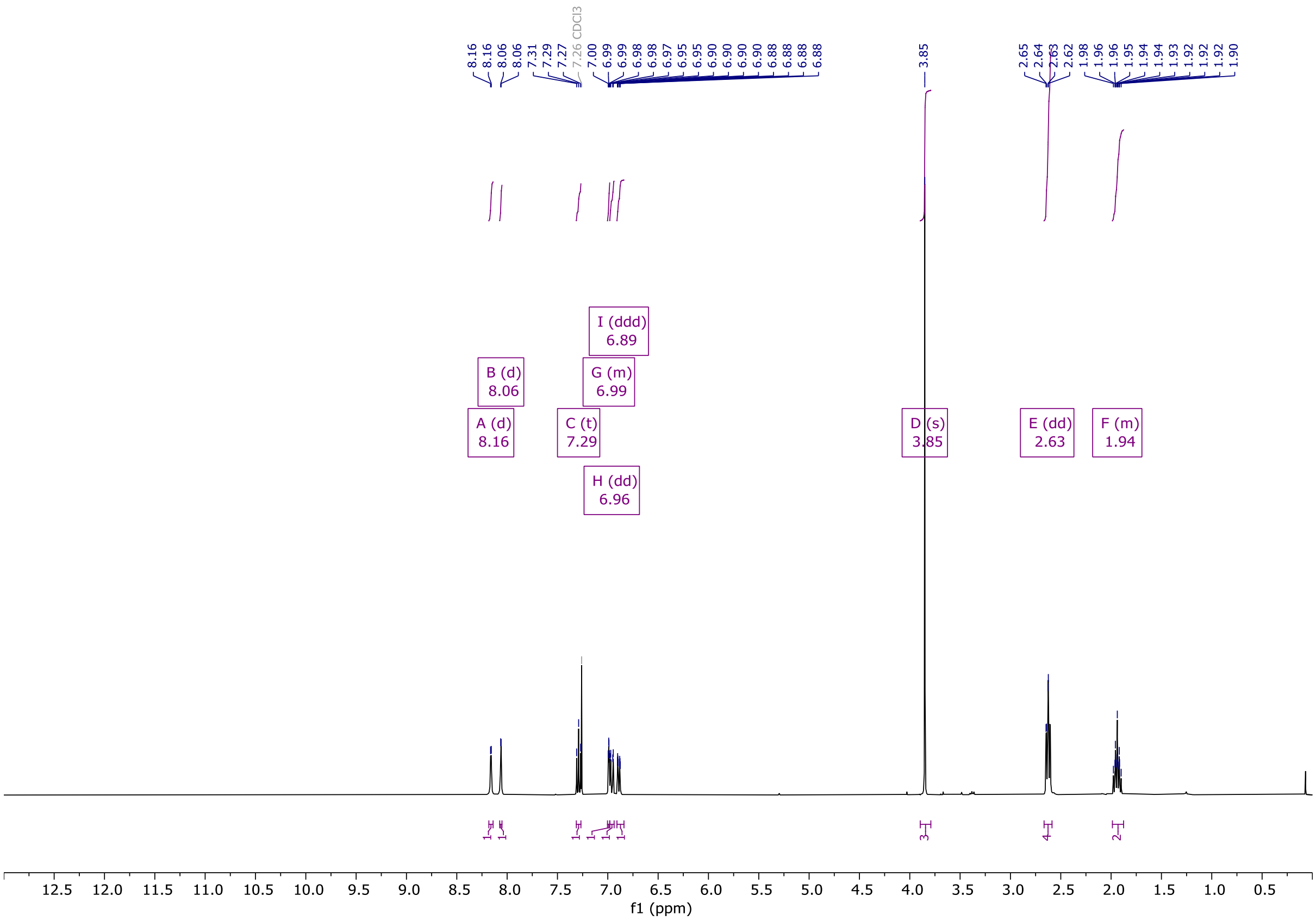

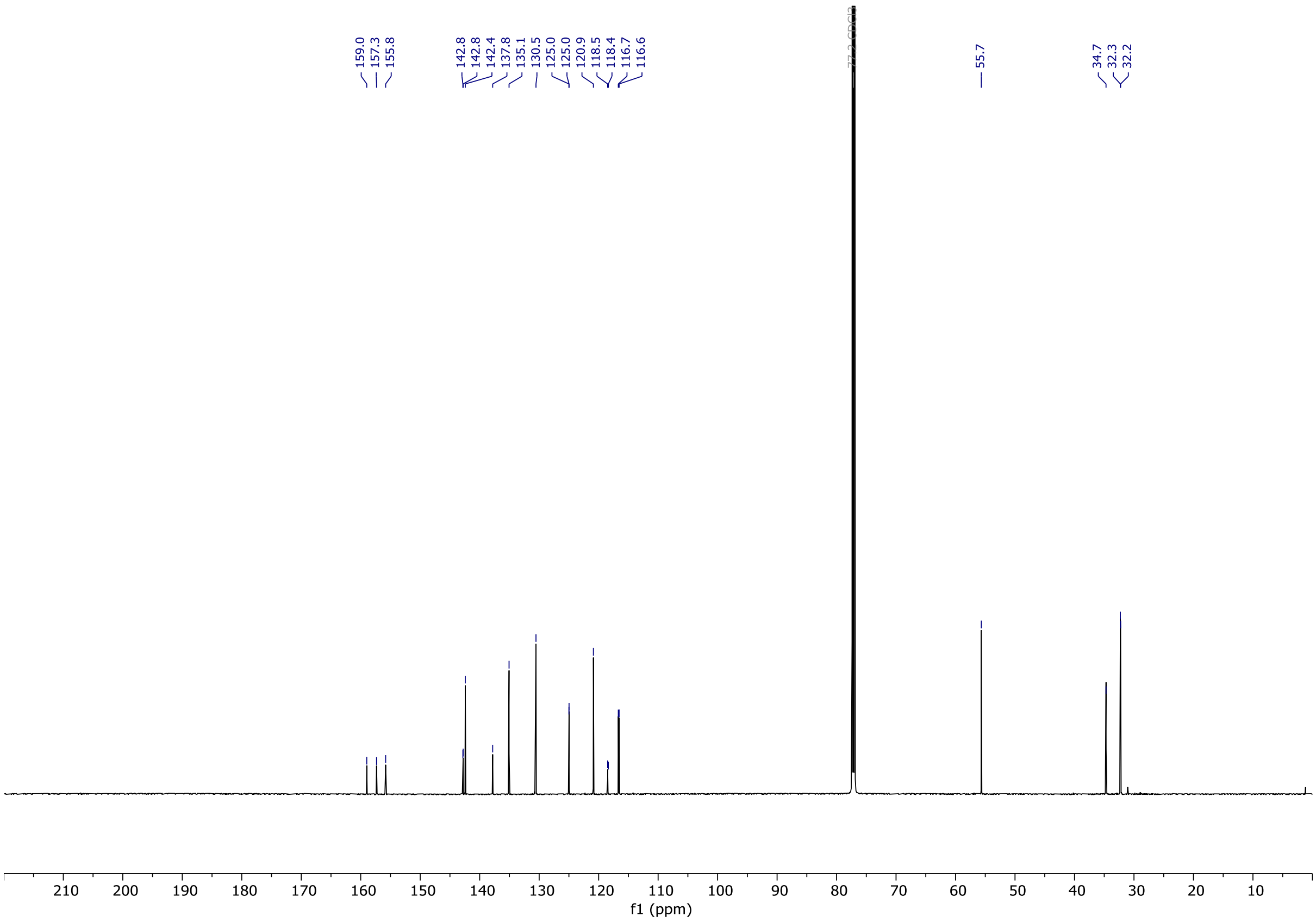

OX04958d

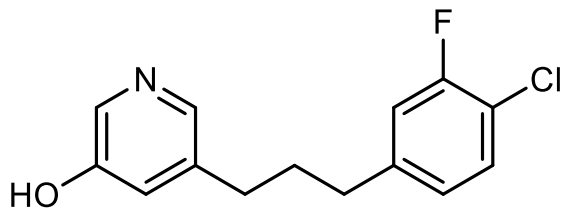

OX04958d

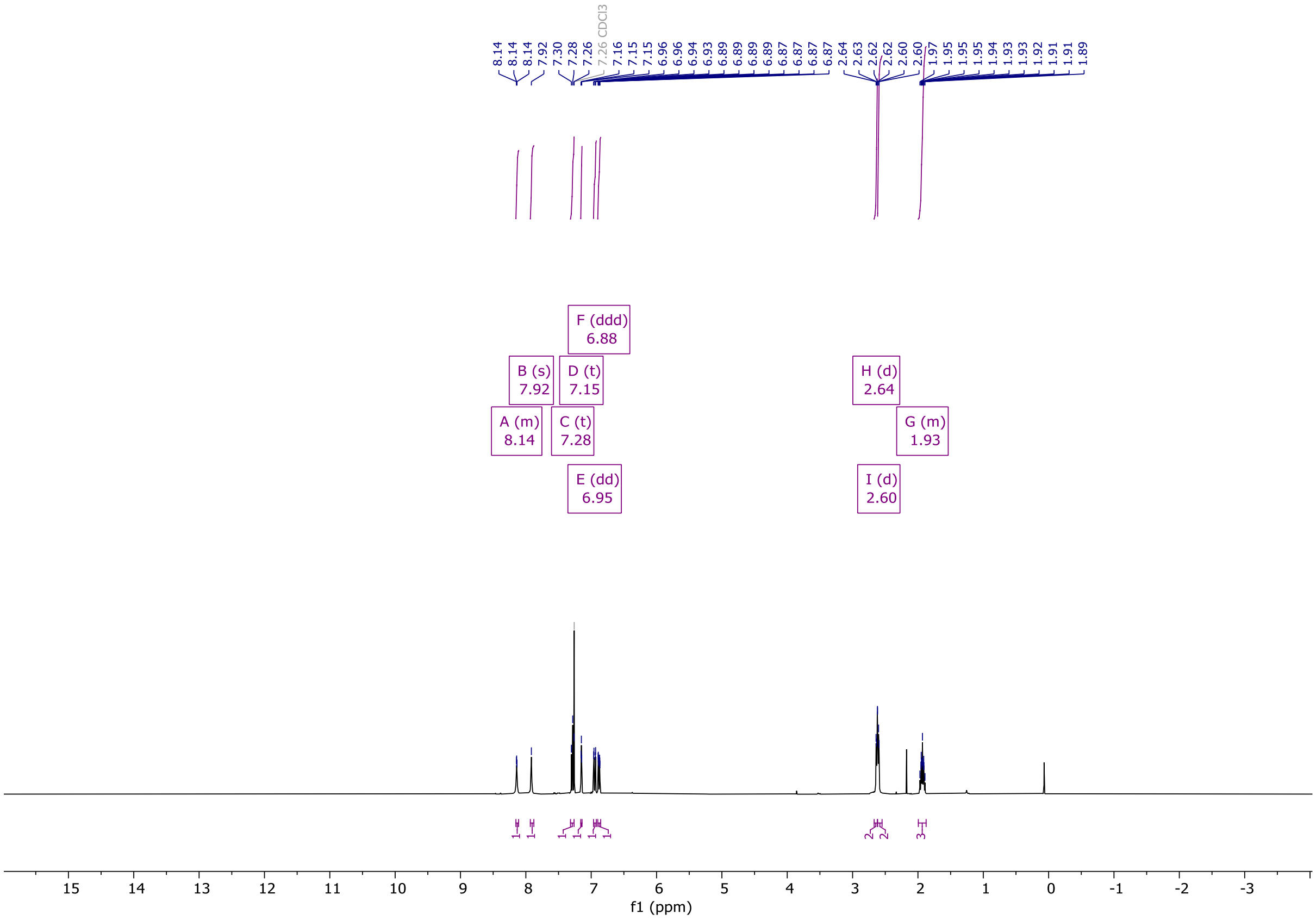

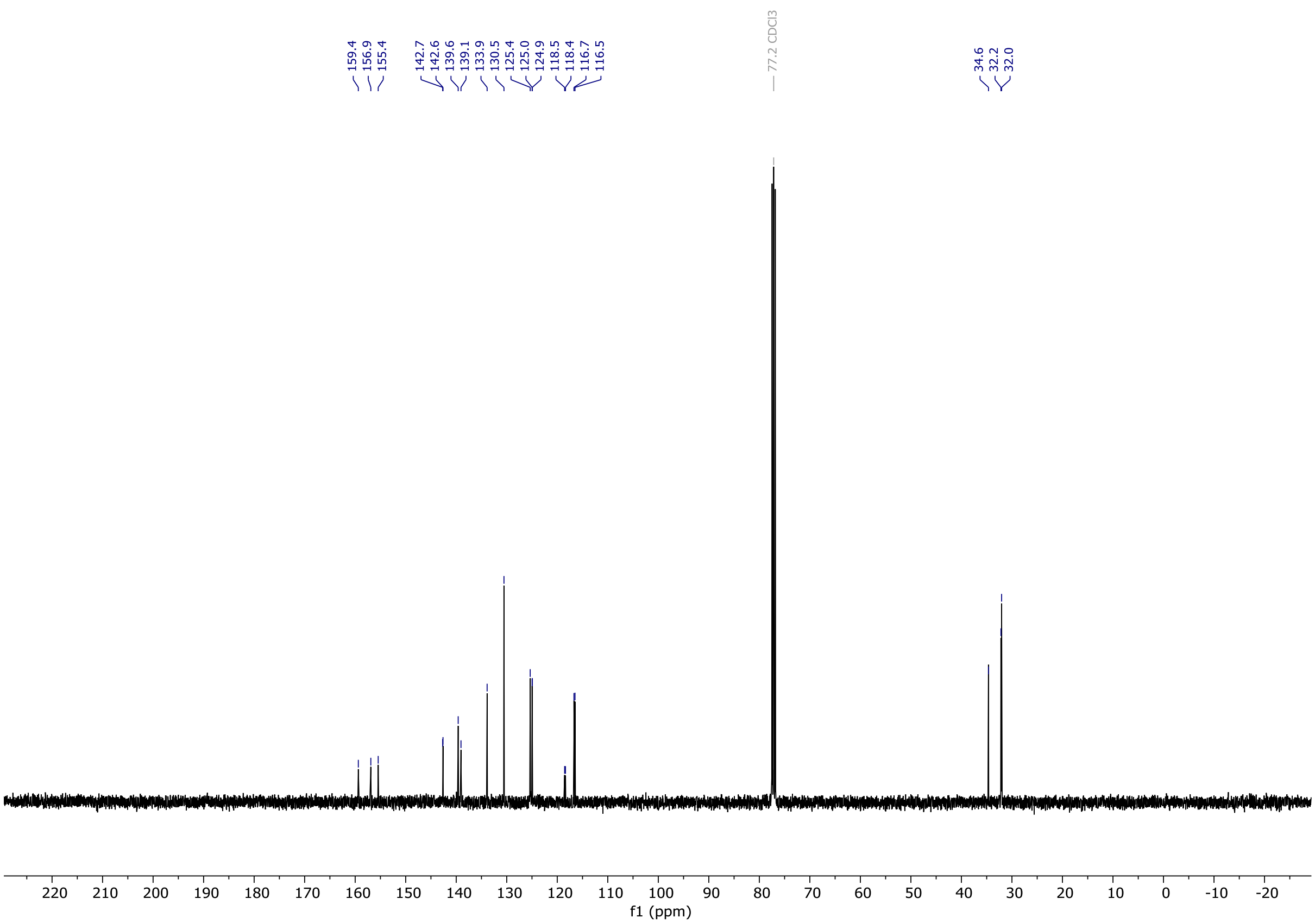

OX04958

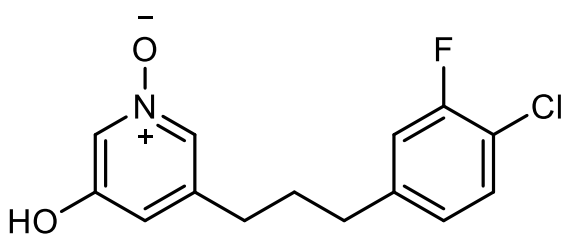

OX04958

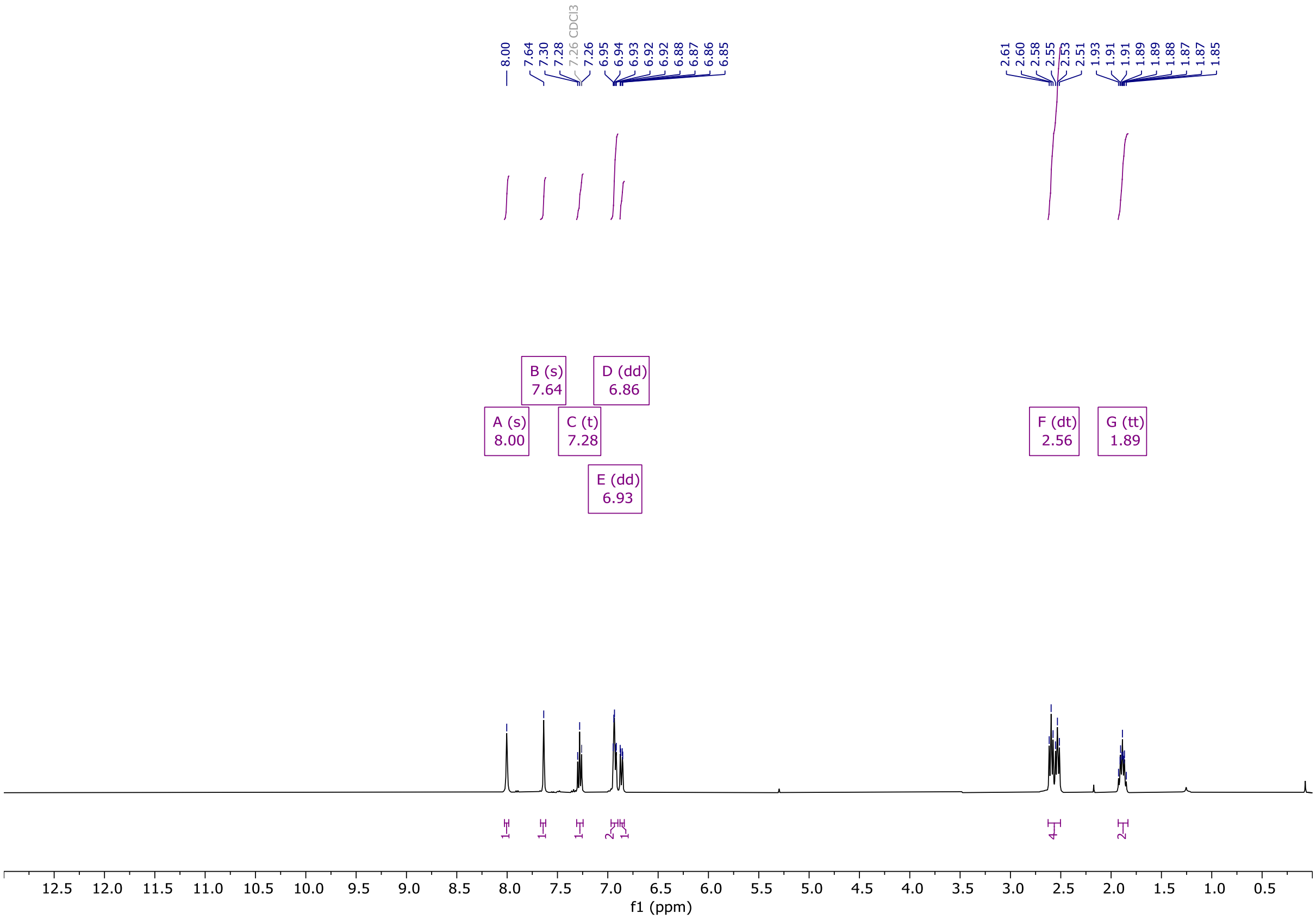

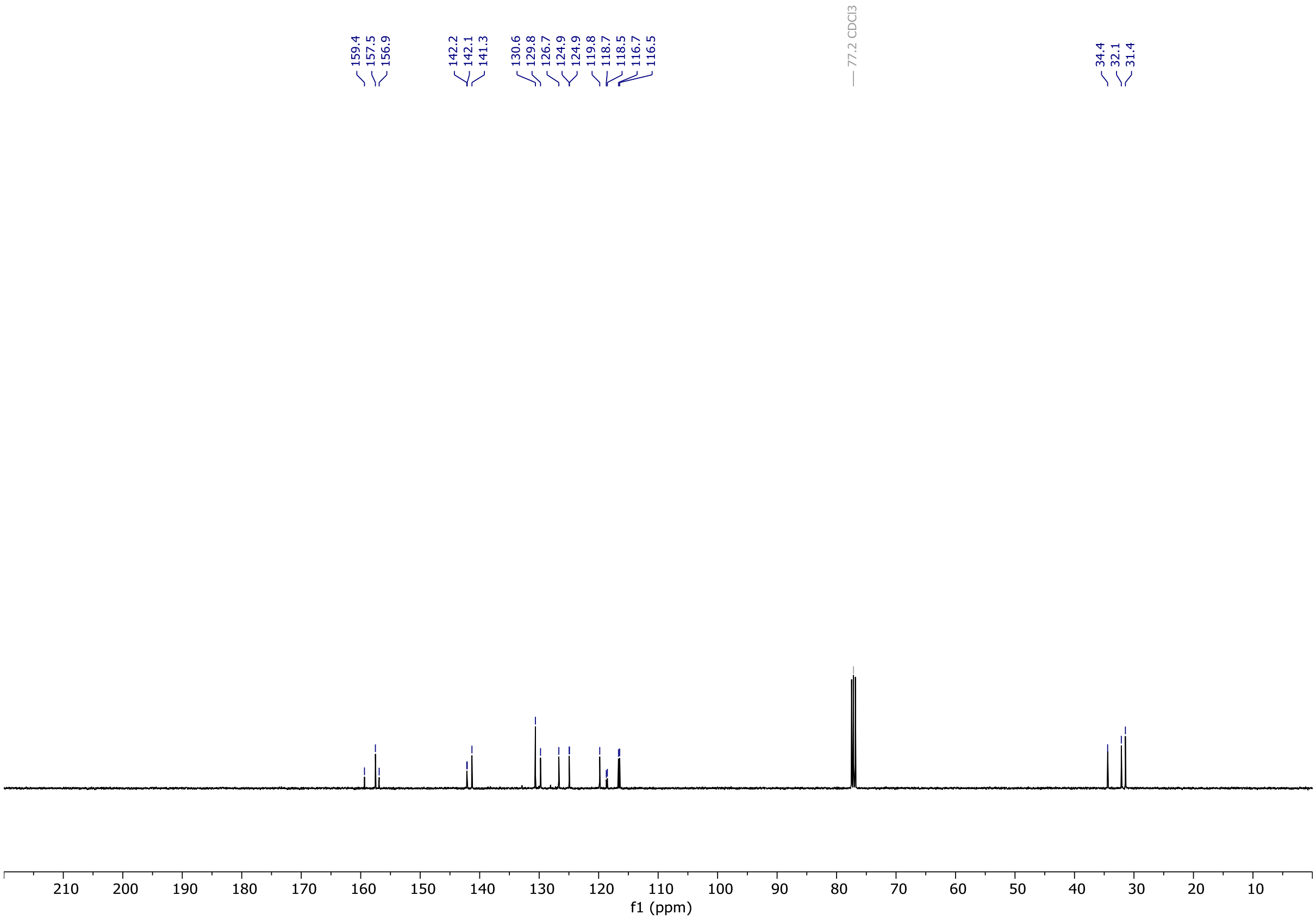

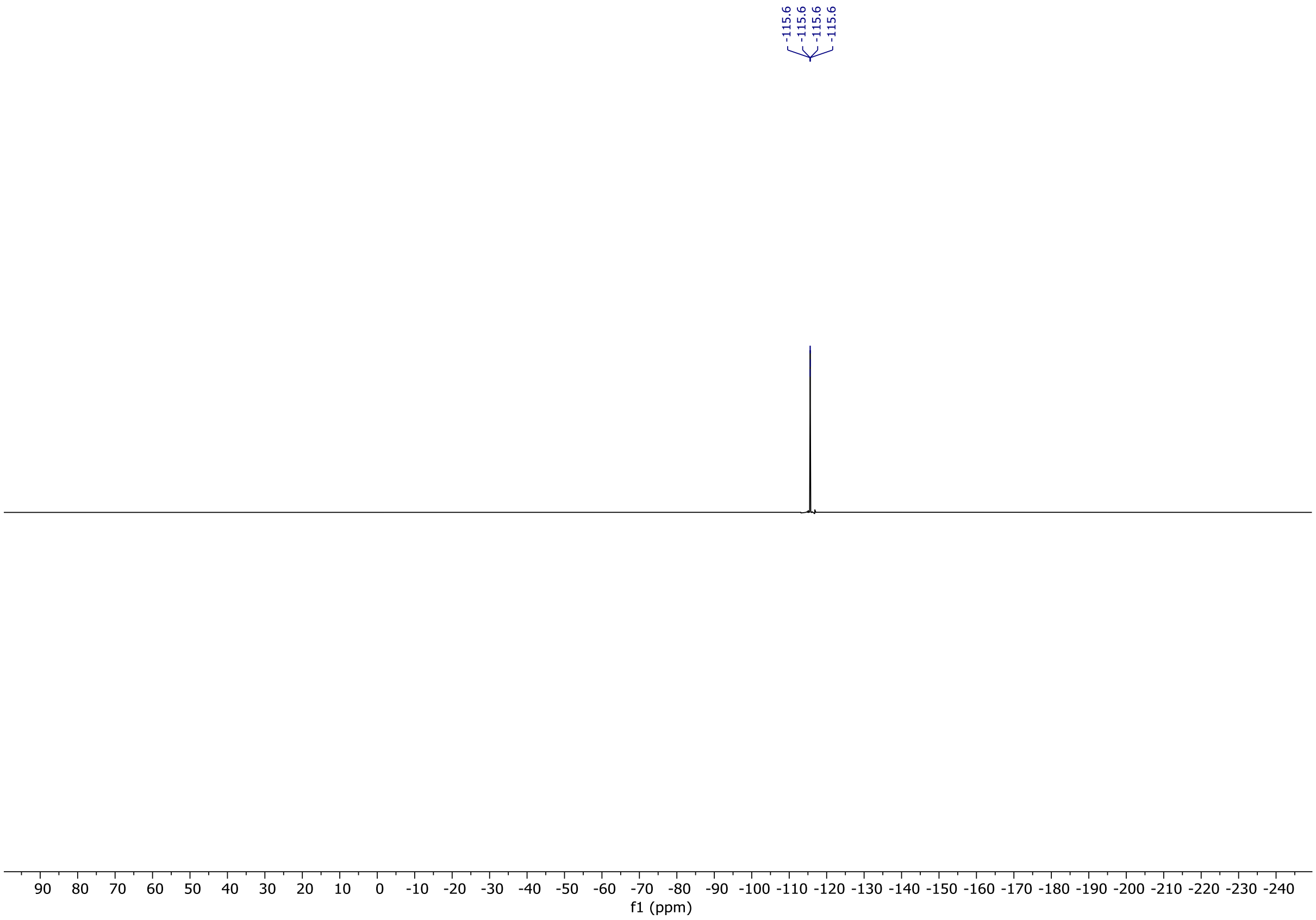
-115.6
-115.6
-115.6
-115.6
90 80 70 60 50 40 30 20 10 0 -10 -20 -30 -40 -50 -60 -70 -80 -90 -100 -110 -120 -130 -140 -150 -160 -170 -180 -190 -200 -210 -220 -230 -240
f1 (ppm)

OX04959a

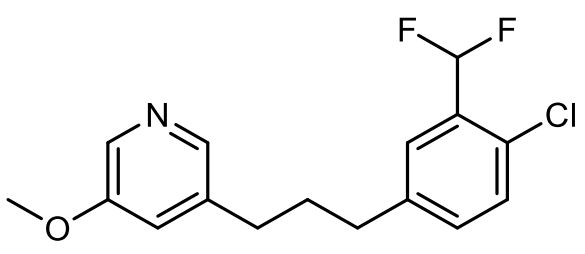

OX04959a

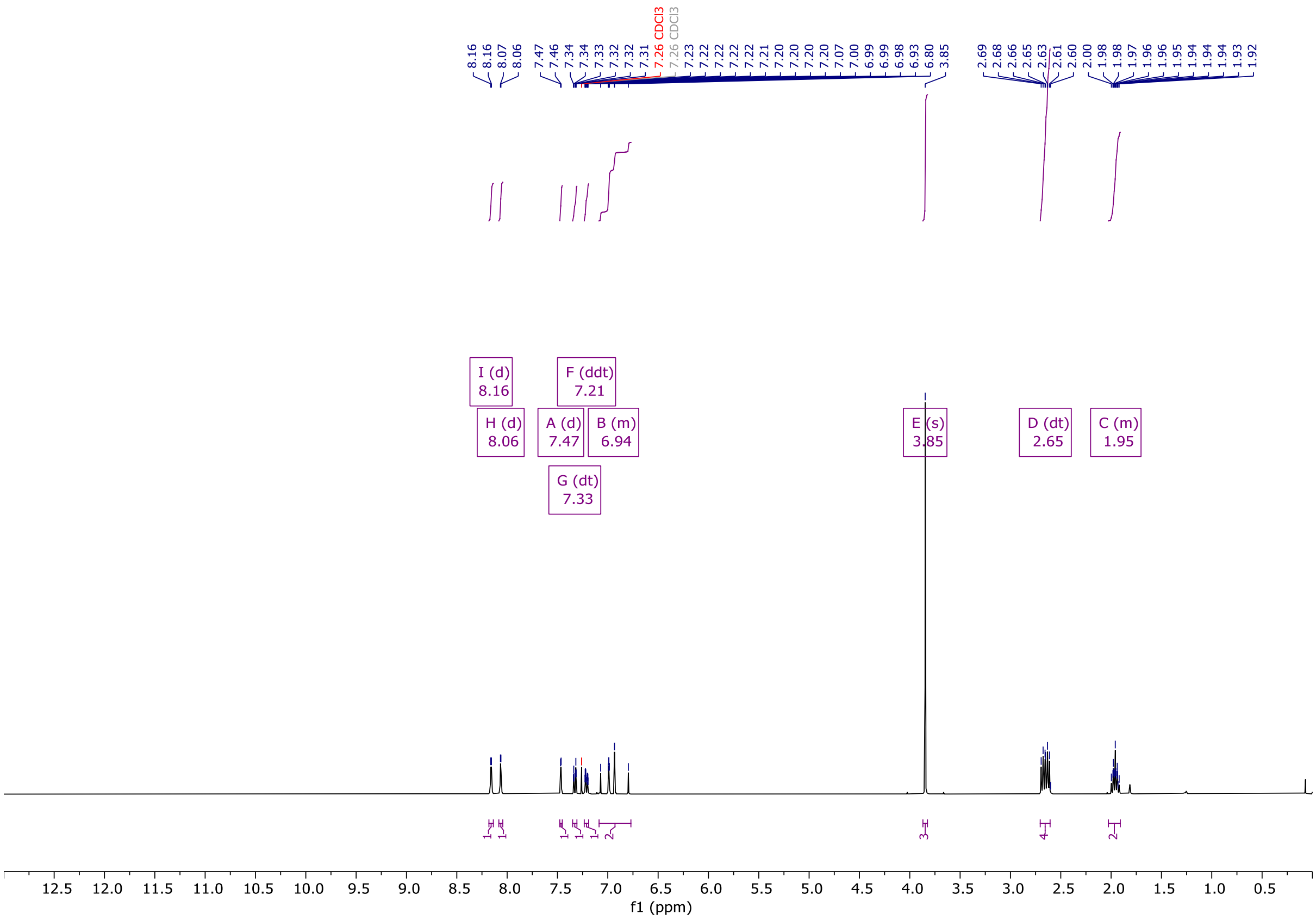

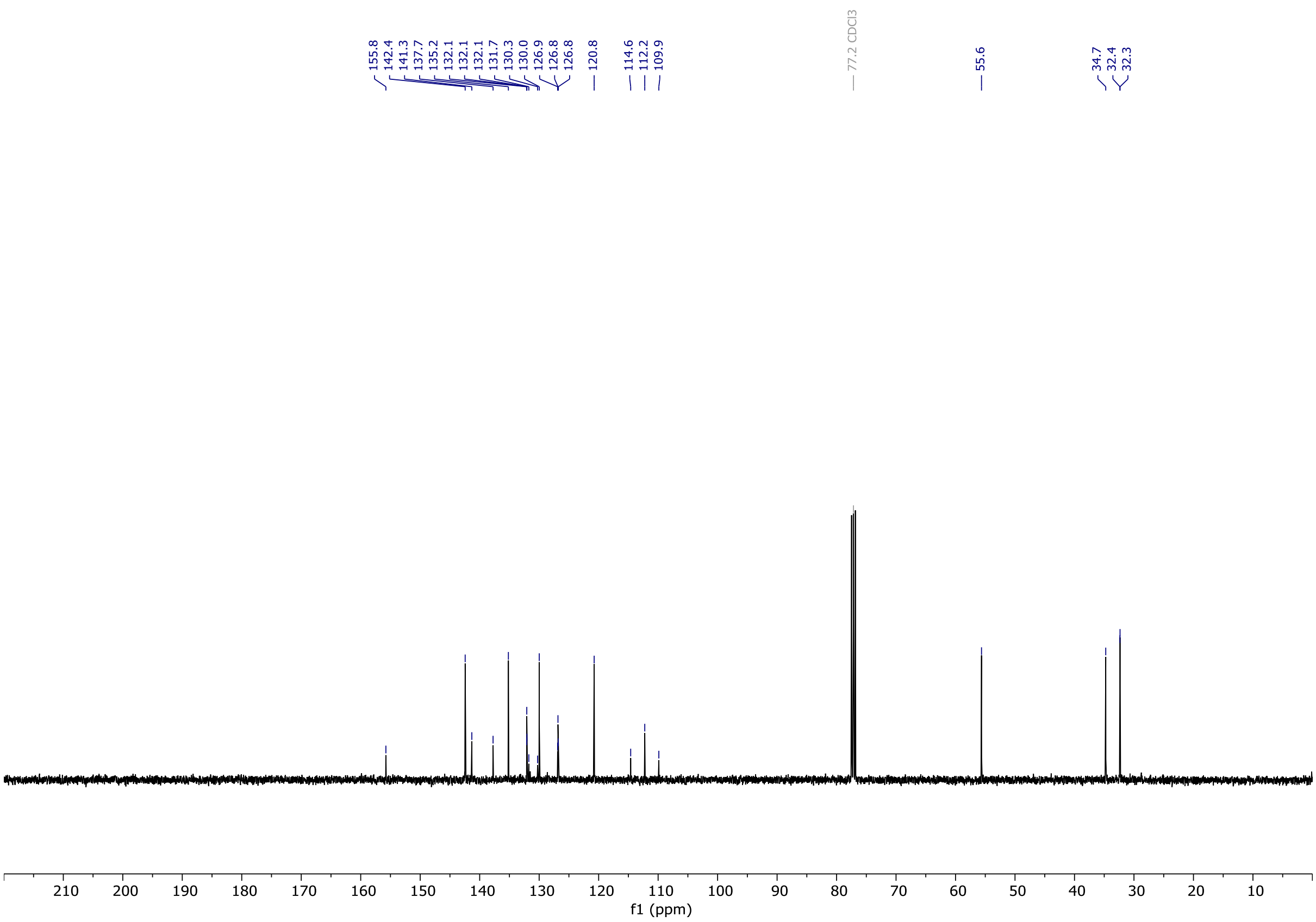

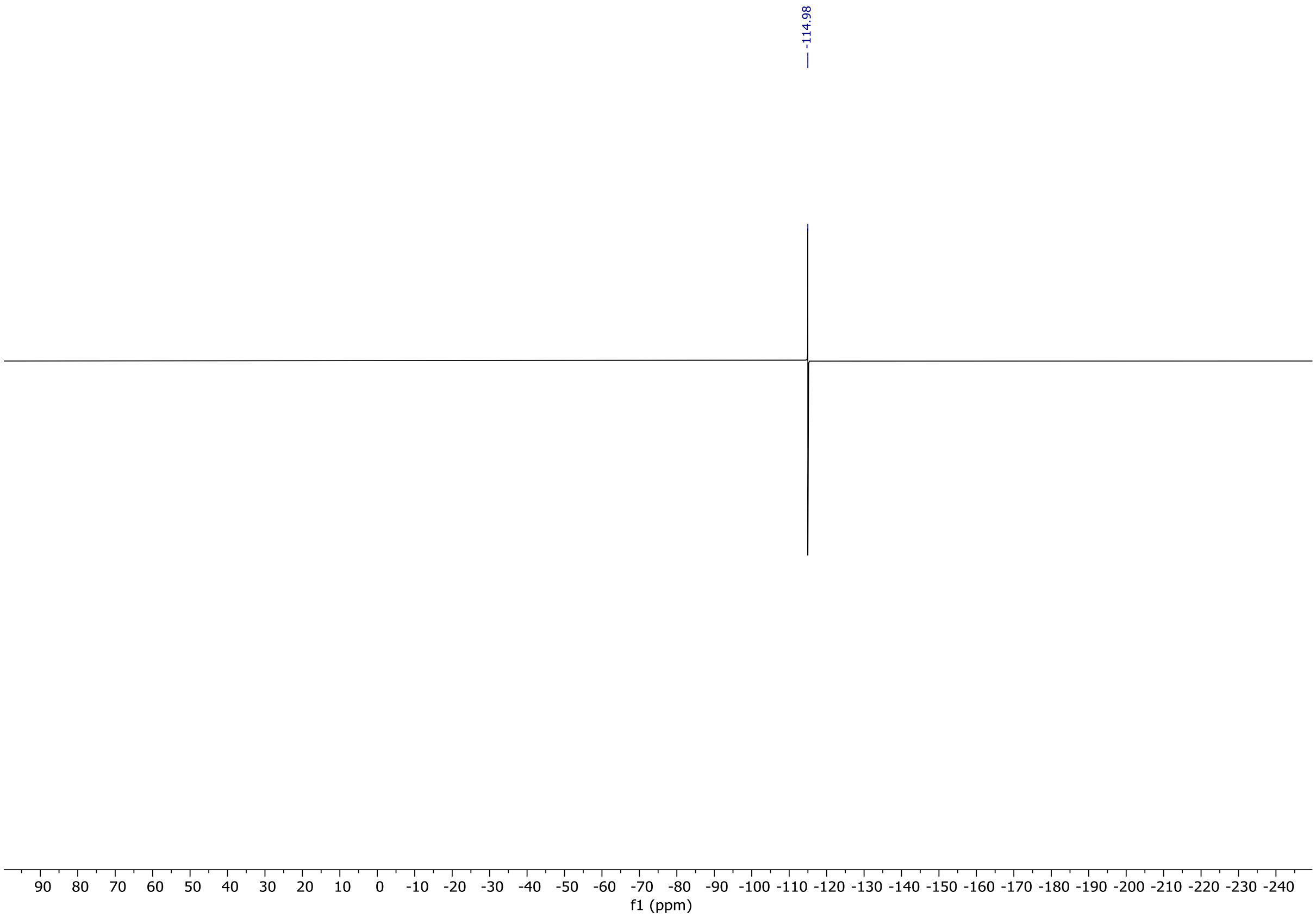
-114.98
90 80 70 60 50 40 30 20 10 0 -10 -20 -30 -40 -50 -60 -70 -80 -90 -100 -110 -120 -130 -140 -150 -160 -170 -180 -190 -200 -210 -220 -230 -240
f1 (ppm)

OX04959b

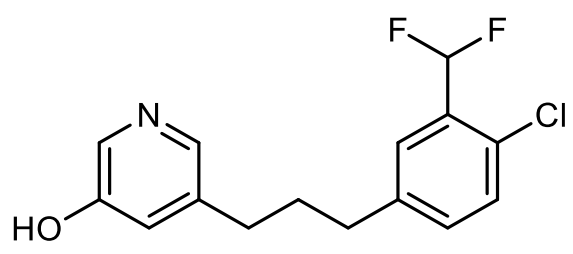

OX04959b

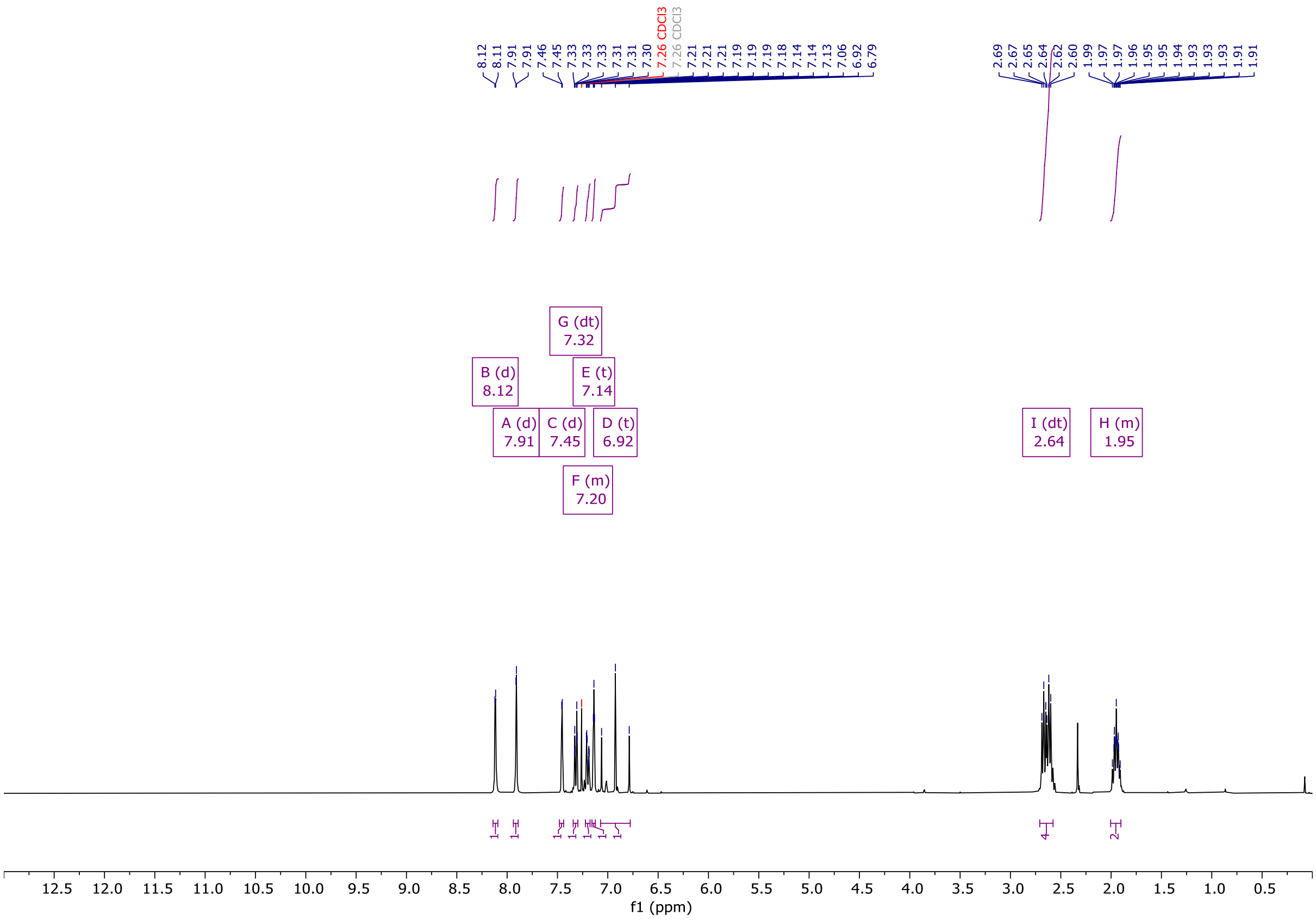

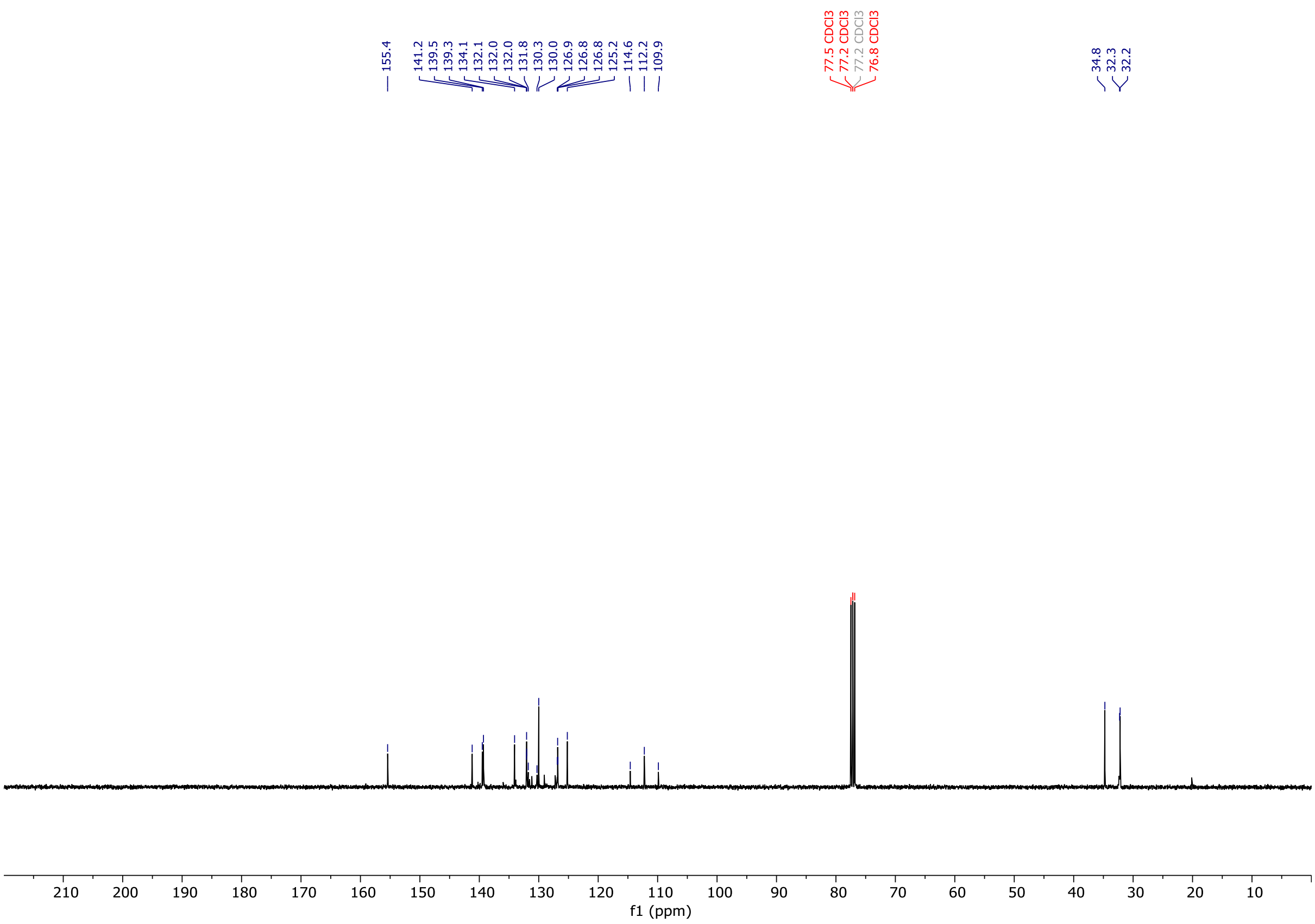

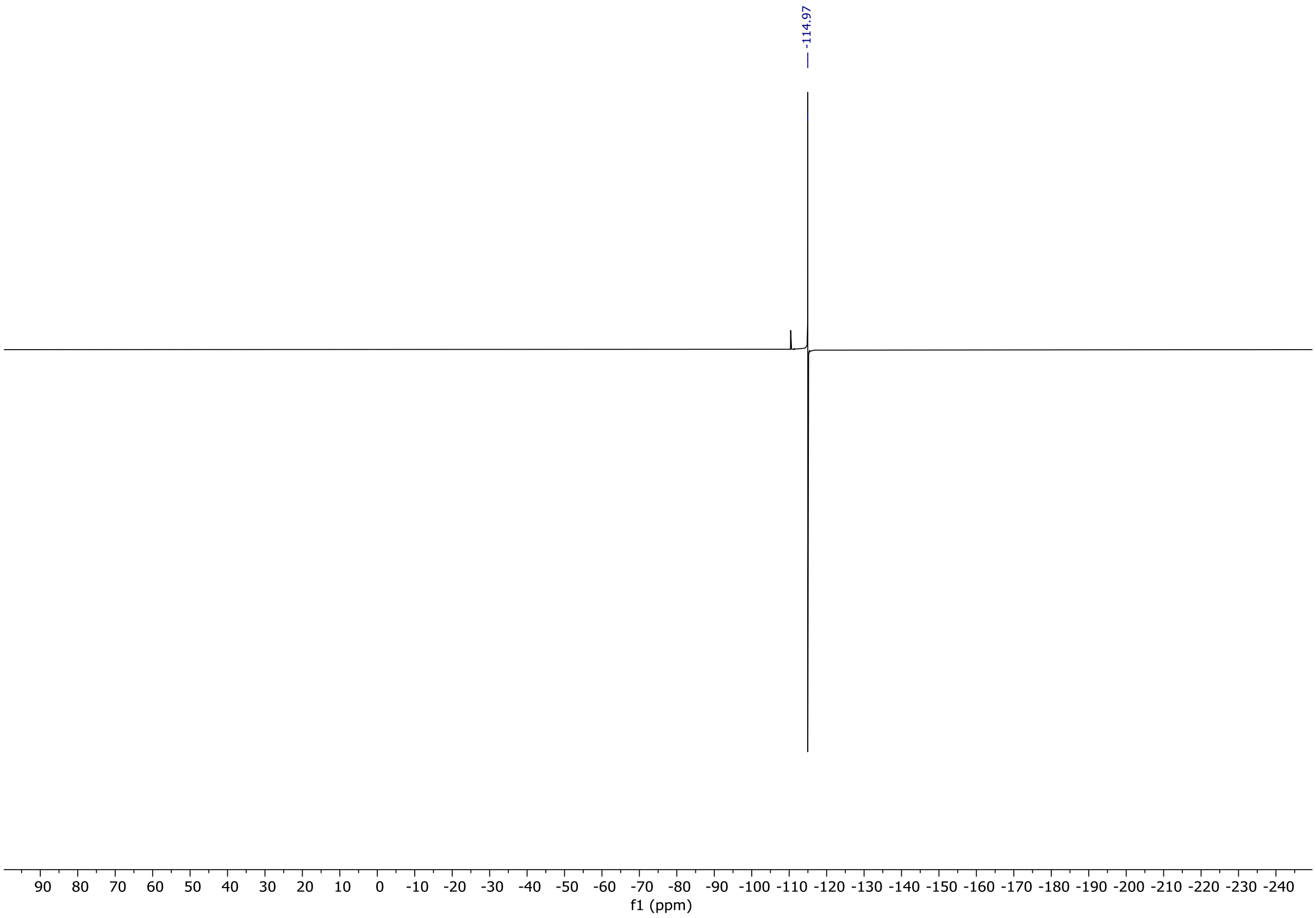

-114.97
90 80 70 60 50 40 30 20 10 0 -10 -20 -30 -40 -50 -60 -70 -80 -90 -100 -110 -120 -130 -140 -150 -160 -170 -180 -190 -200 -210 -220 -230 -240
f1 (ppm)

OX04959

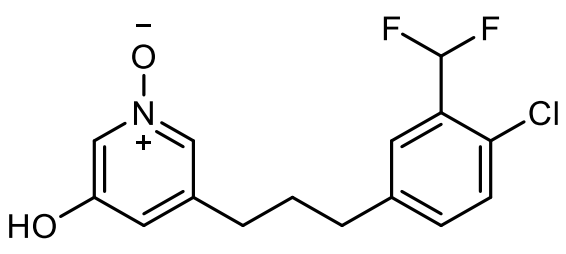

OX04959

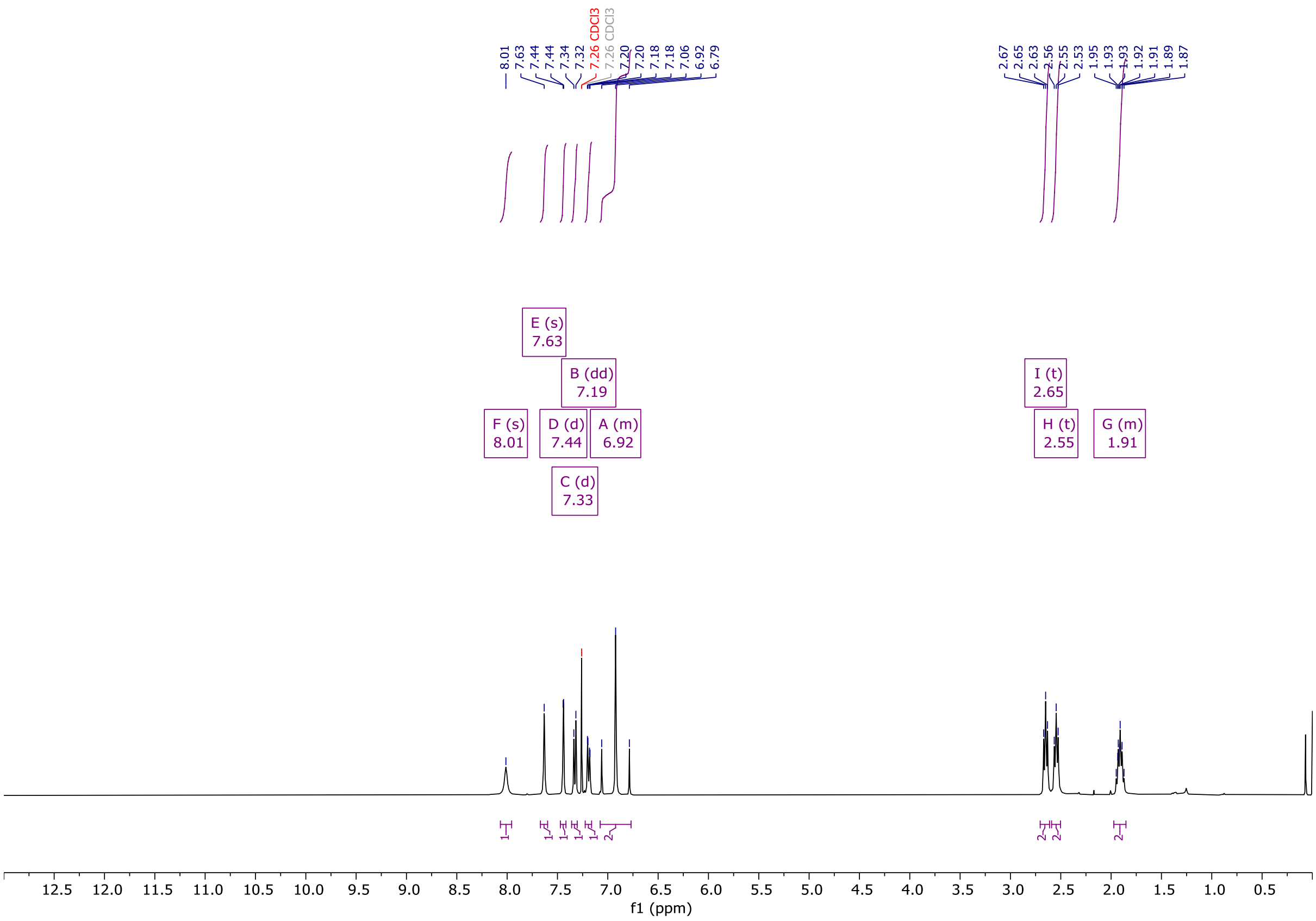

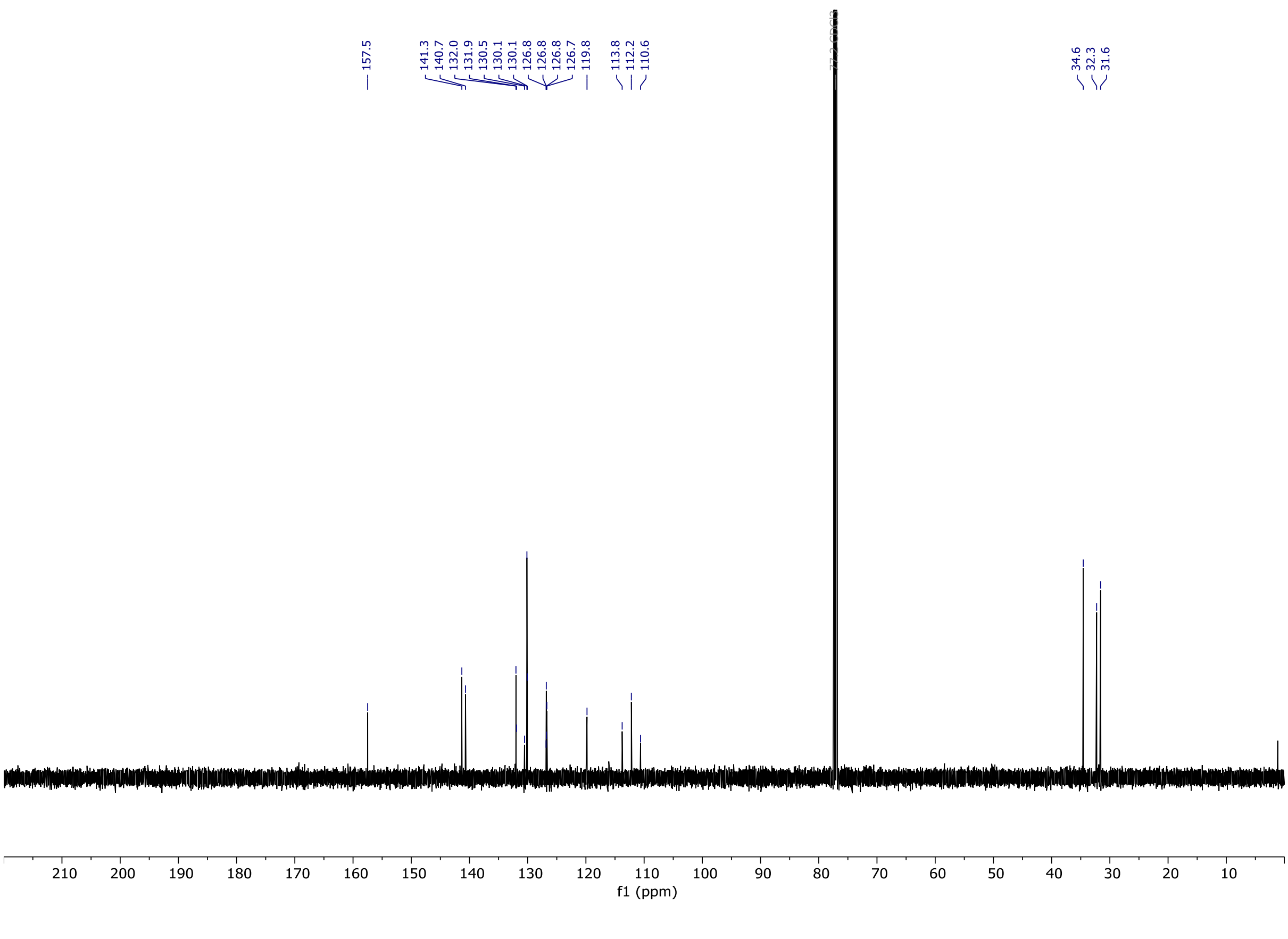

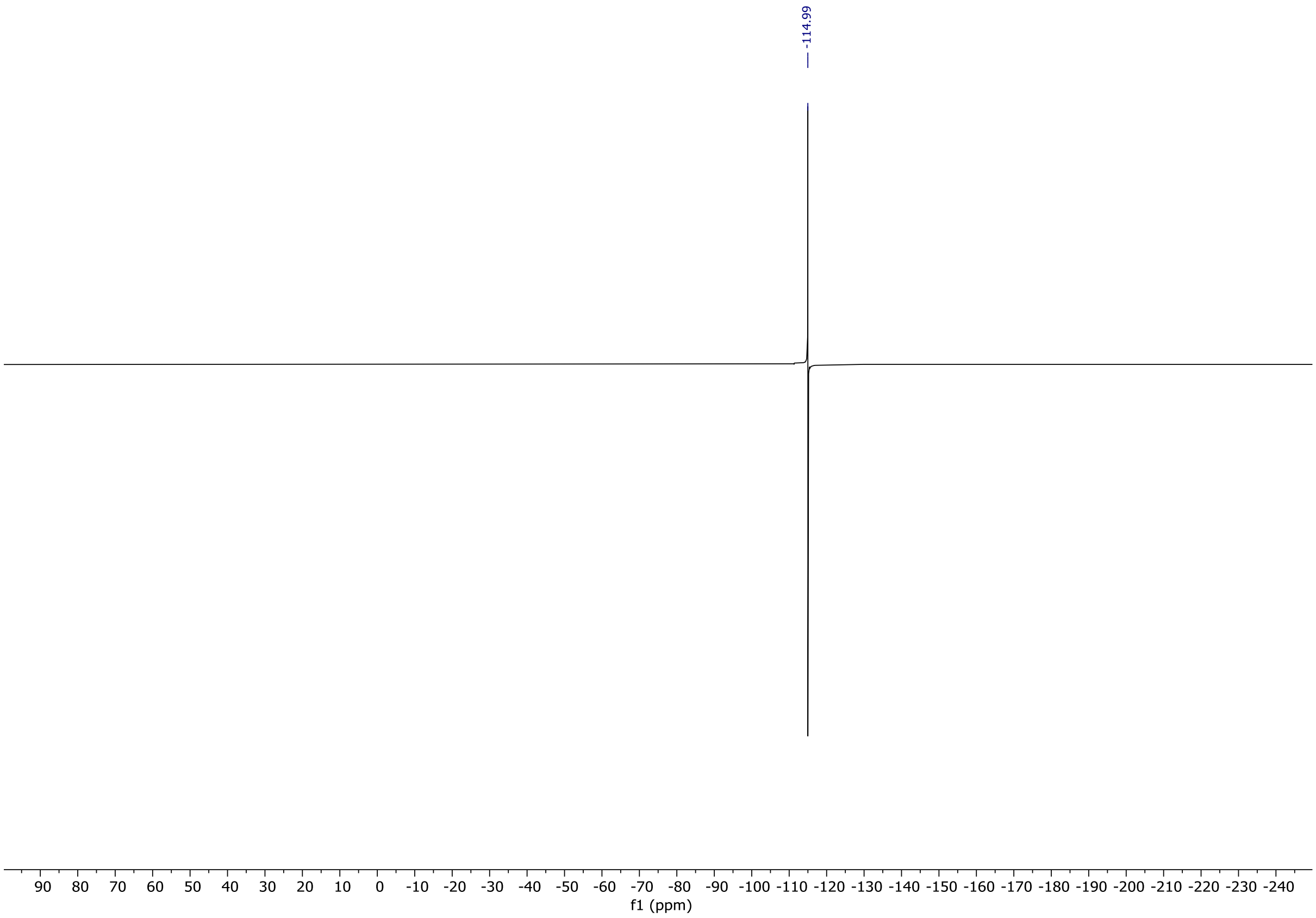
-114.99
90 80 70 60 50 40 30 20 10 0 -10 -20 -30 -40 -50 -60 -70 -80 -90 -100 -110 -120 -130 -140 -150 -160 -170 -180 -190 -200 -210 -220 -230 -240
f1 (ppm)

OX04960a

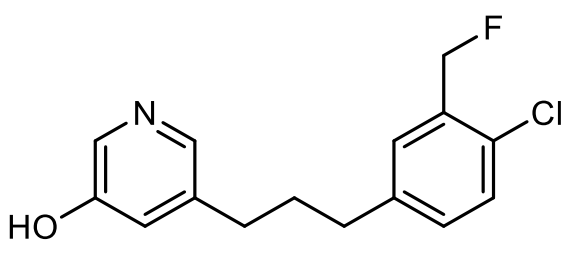

OX04960a

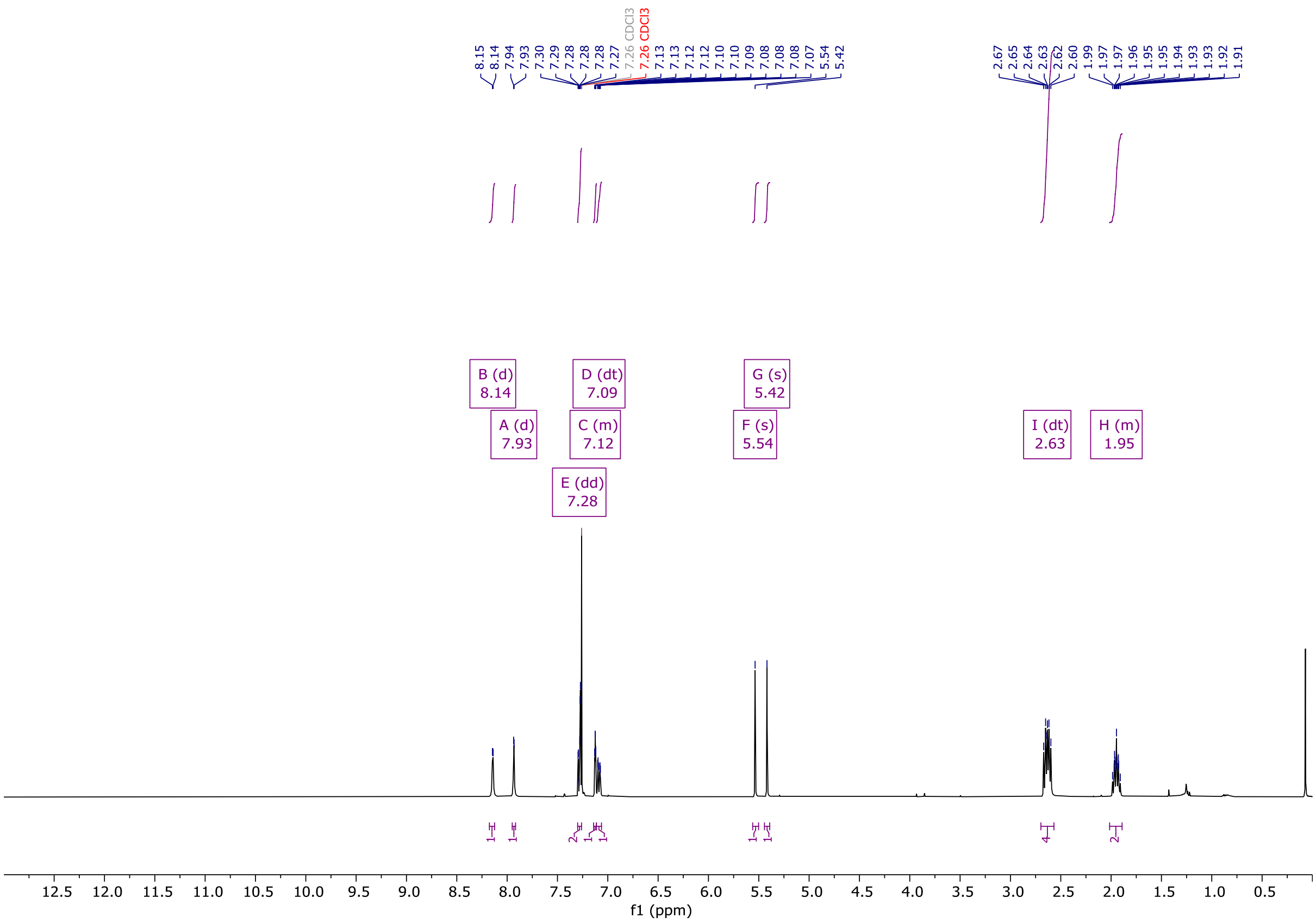

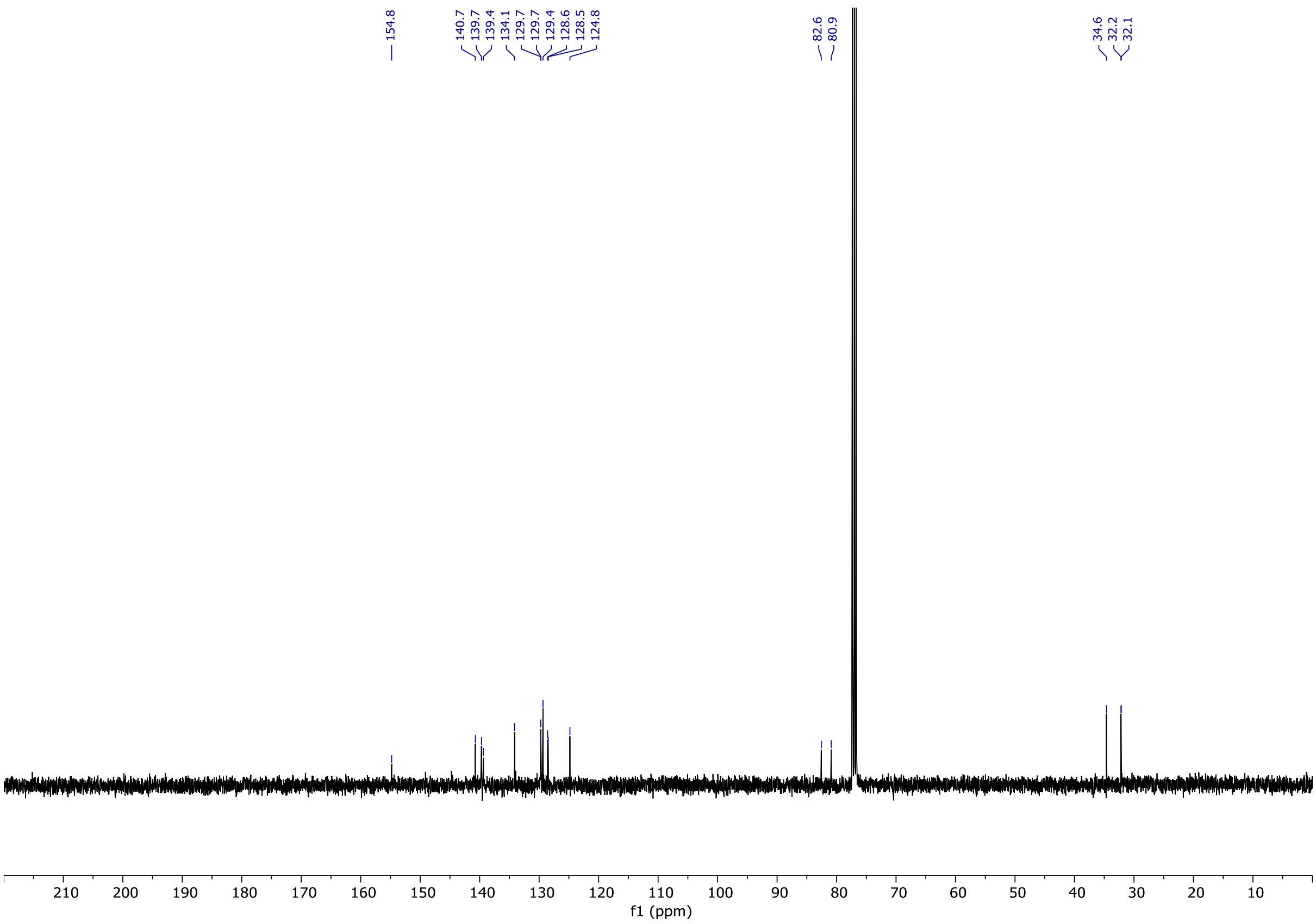

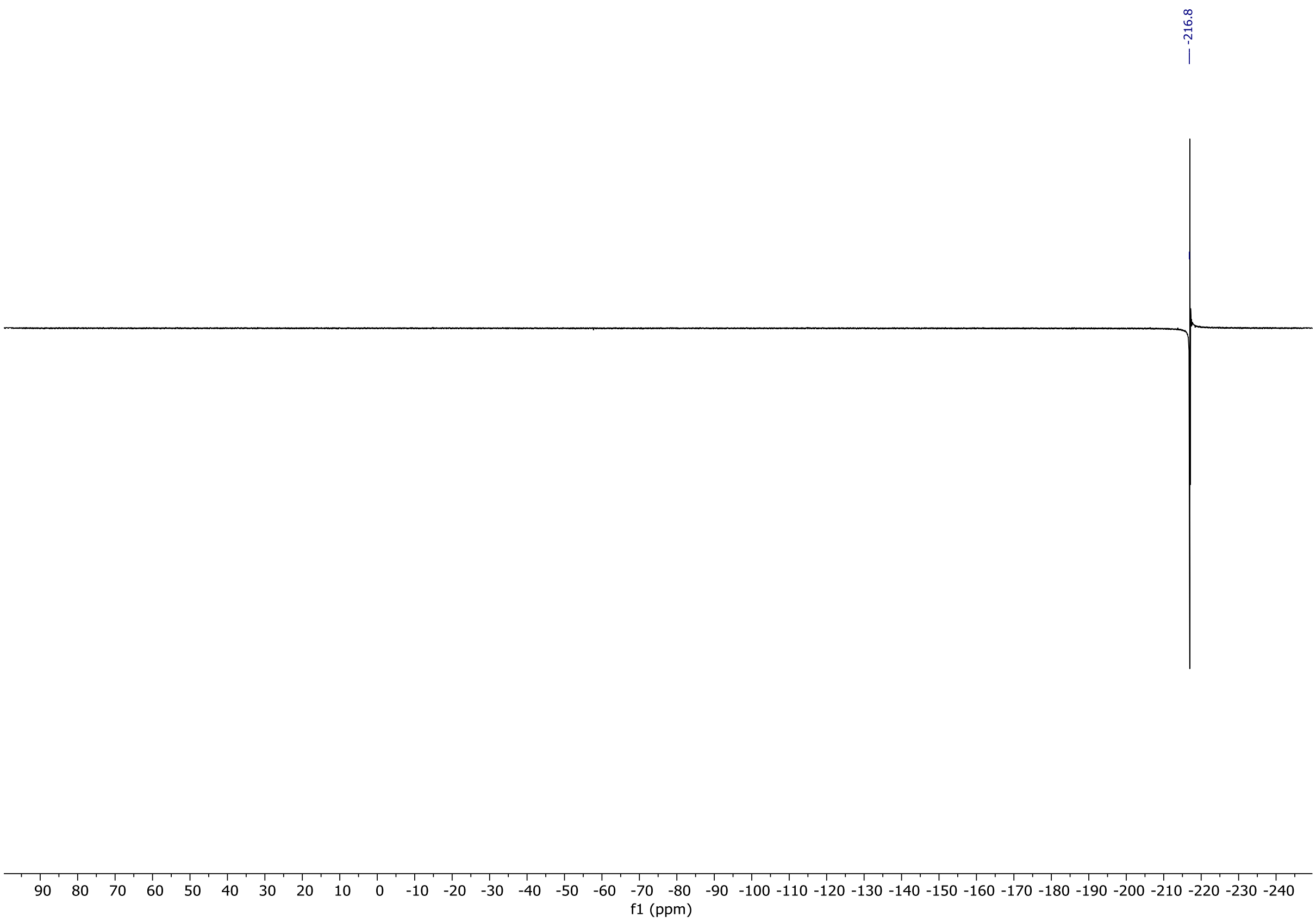

-216.8
90 80 70 60 50 40 30 20 10 0 -10 -20 -30 -40 -50 -60 -70 -80 -90 -100 -110 -120 -130 -140 -150 -160 -170 -180 -190 -200 -210 -220 -230 -240
f1 (ppm)

OX04960

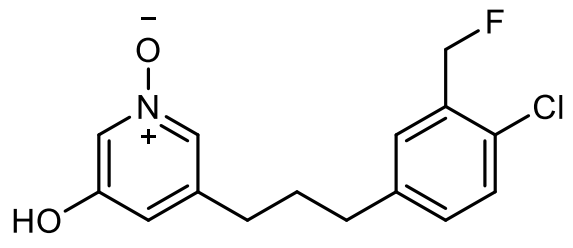

OX04960

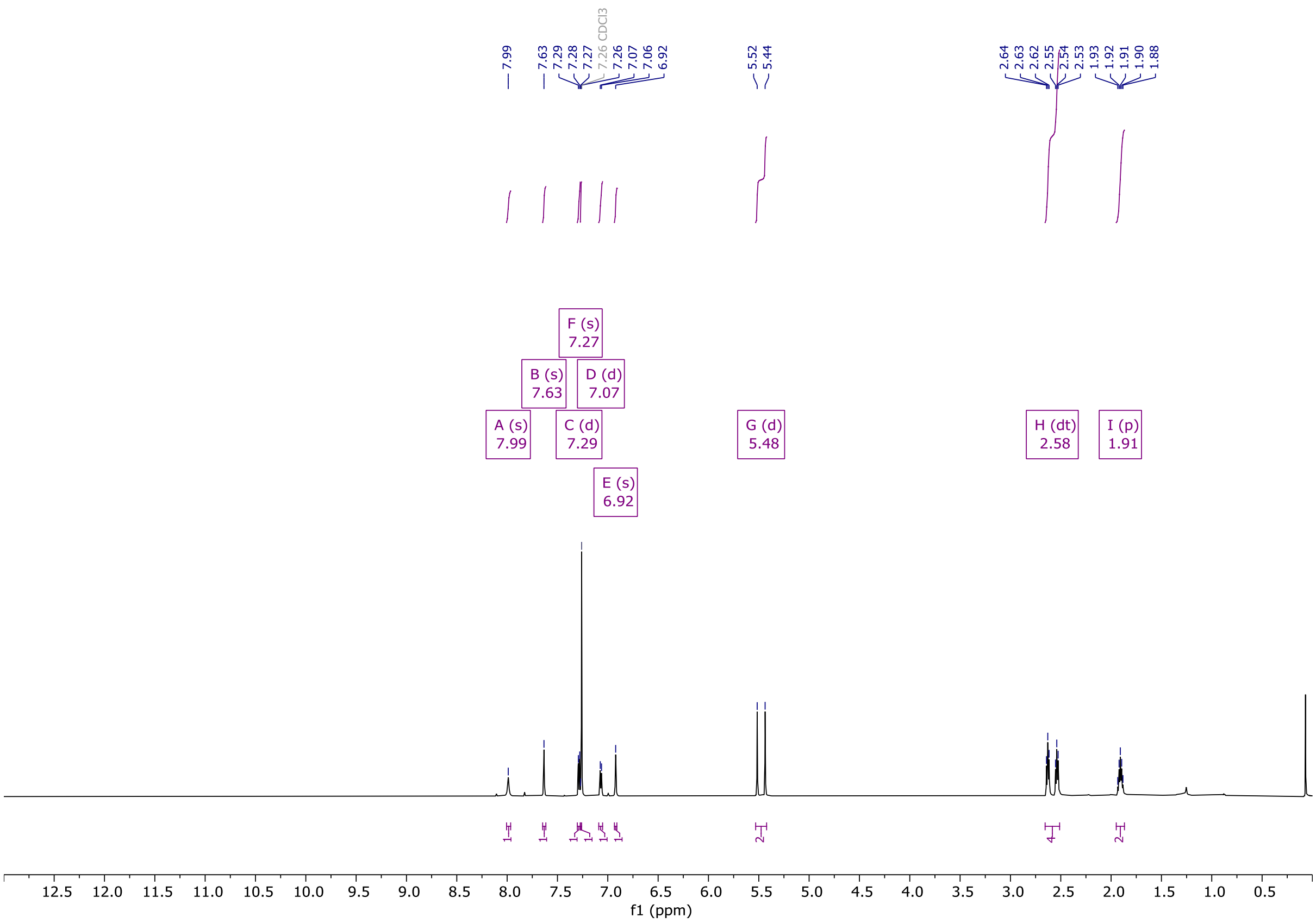

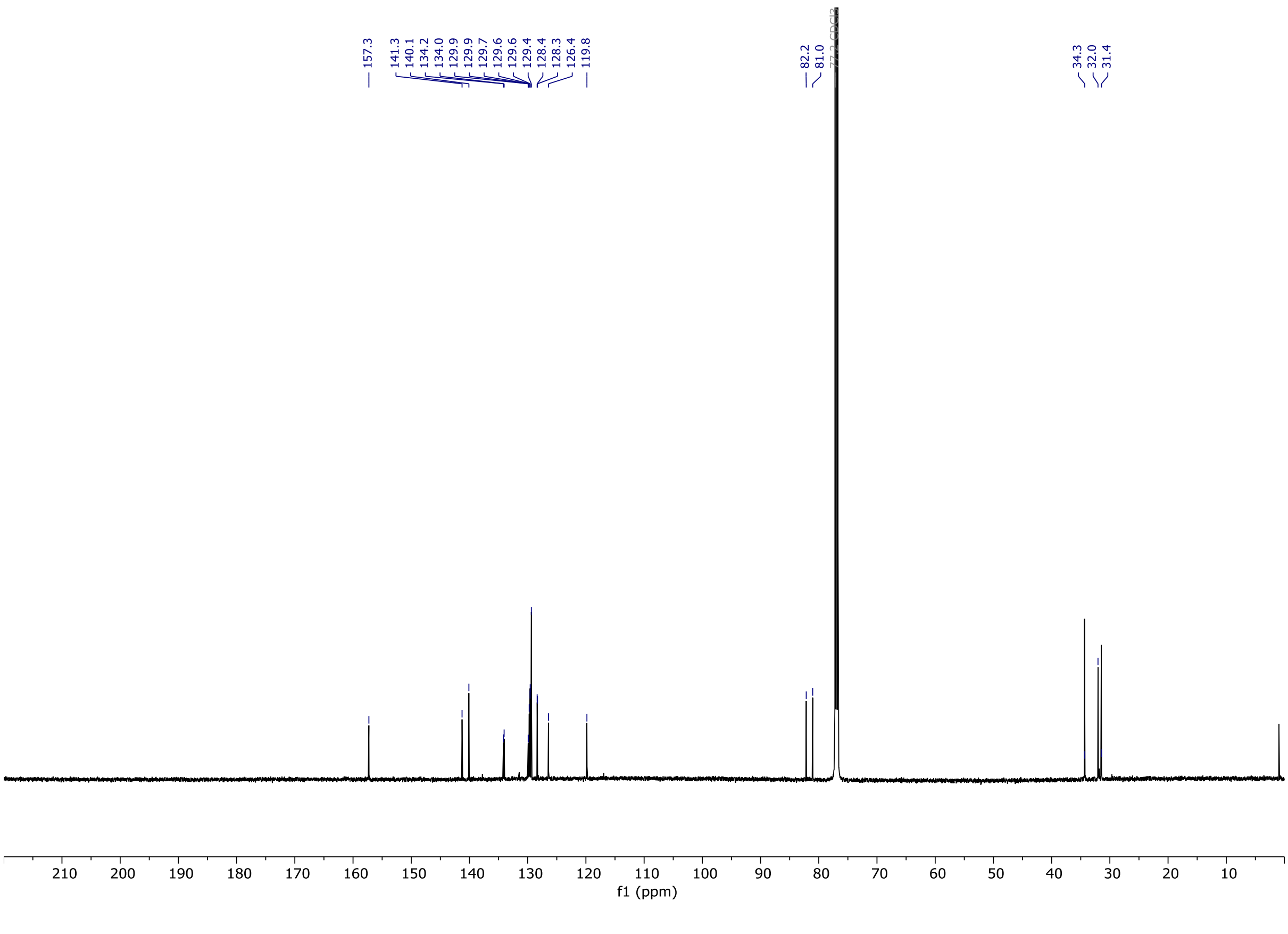

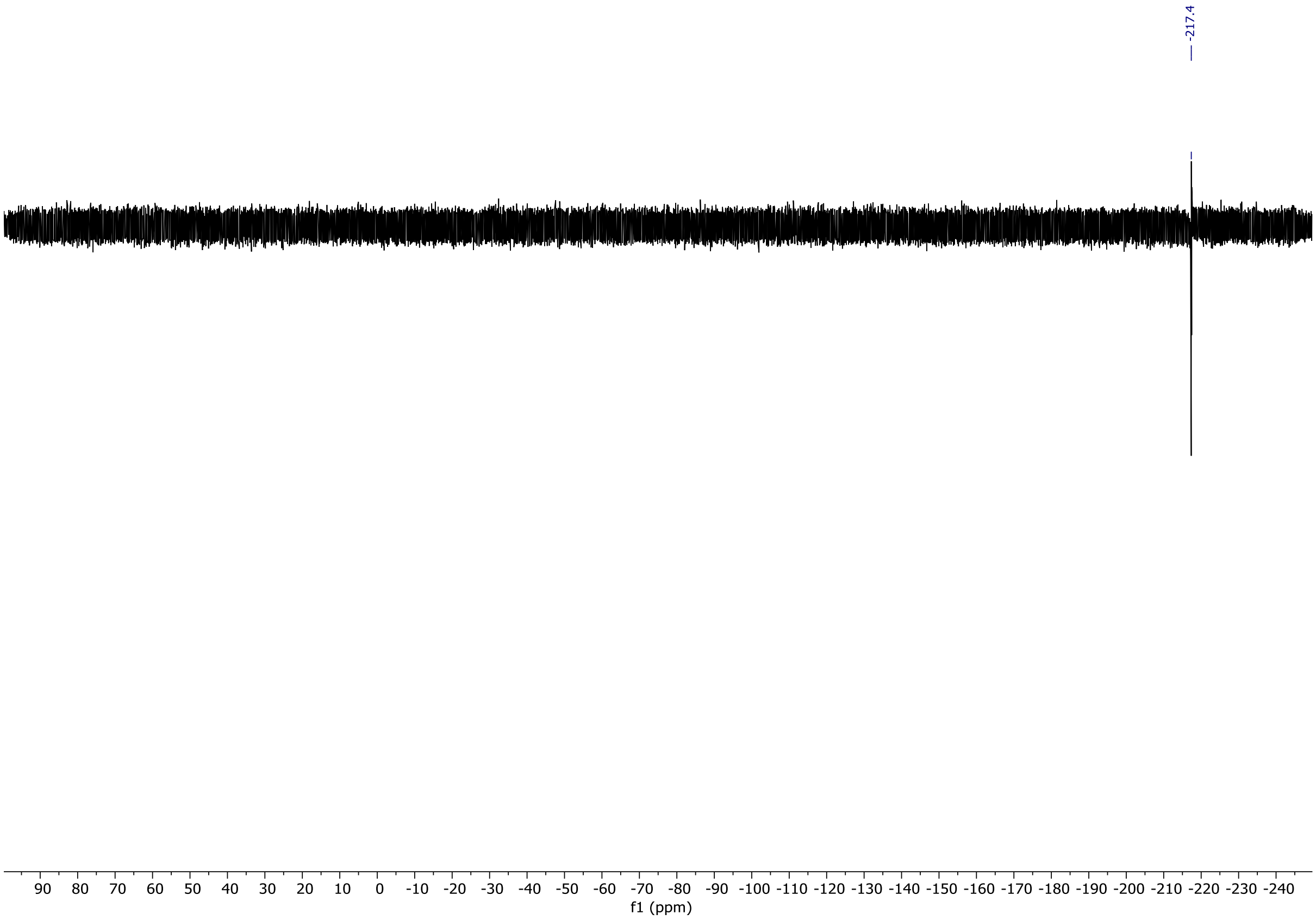

-217.4
90 80 70 60 50 40 30 20 10 0 -10 -20 -30 -40 -50 -60 -70 -80 -90 -100 -110 -120 -130 -140 -150 -160 -170 -180 -190 -200 -210 -220 -230 -240
f1 (ppm)

OX04961a

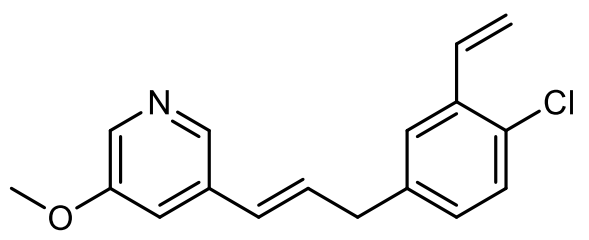

OX04961a

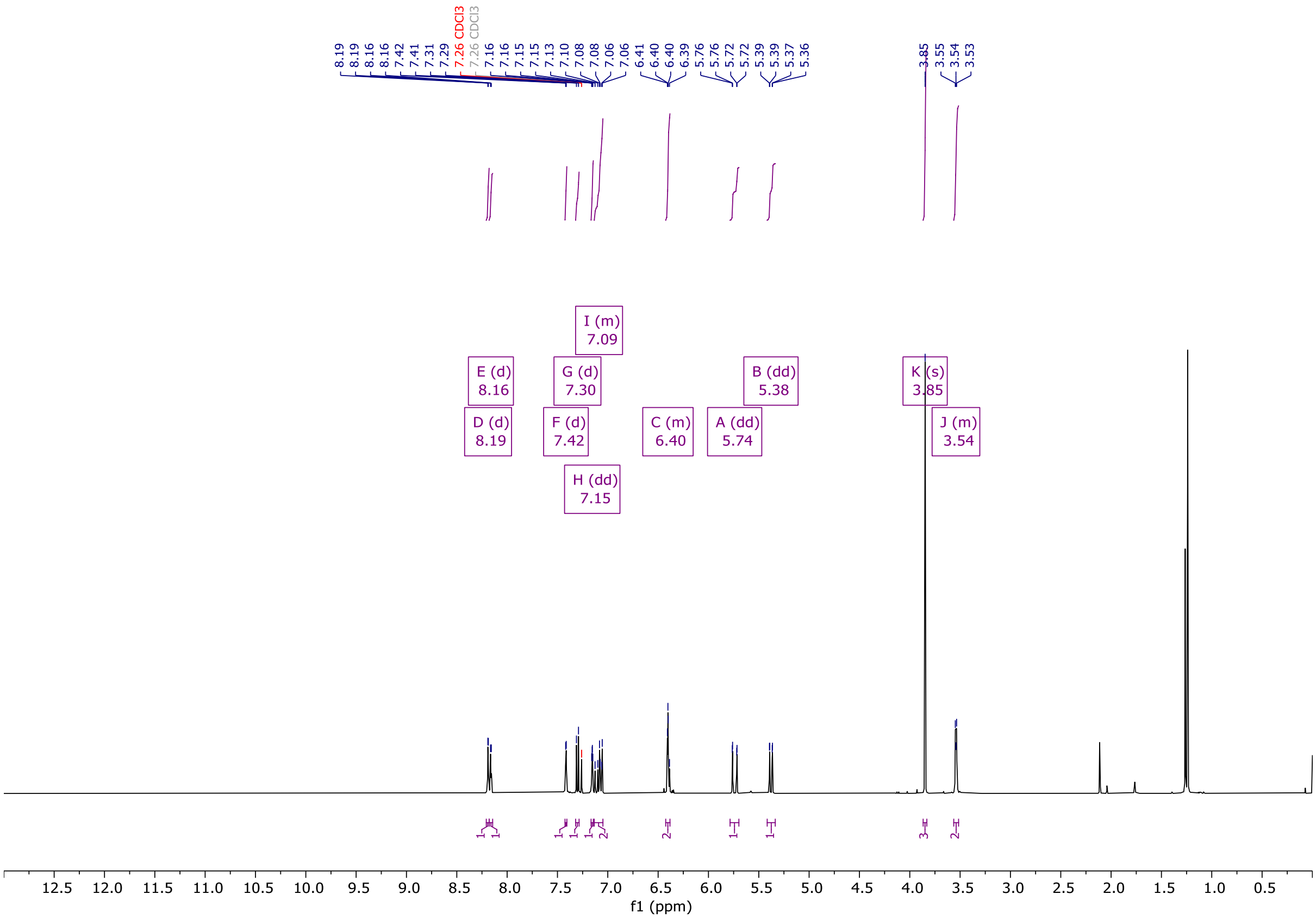

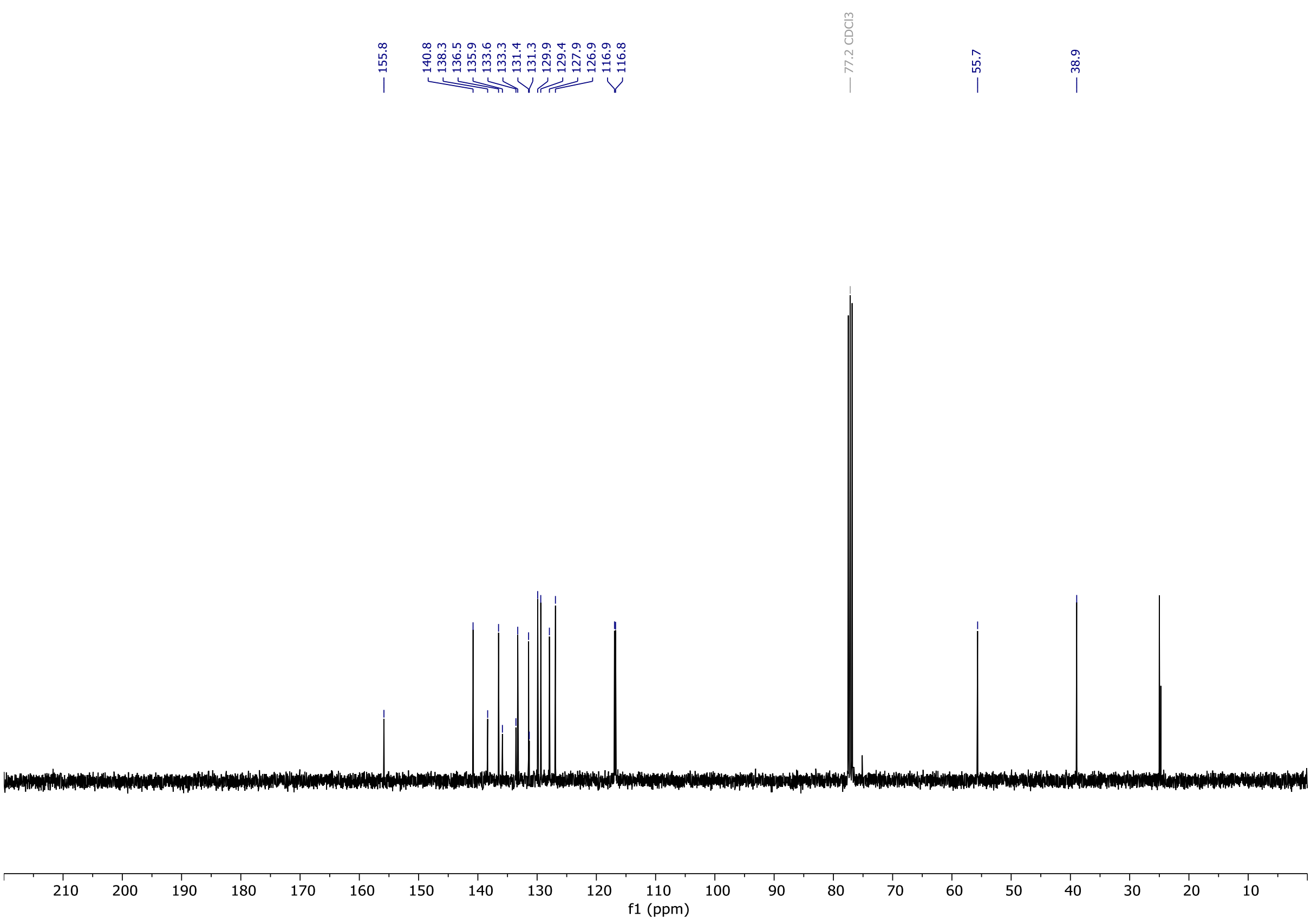

OX04961b

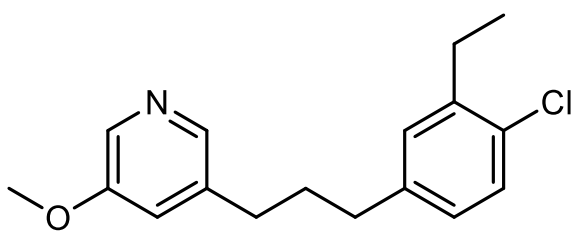

OX04961b

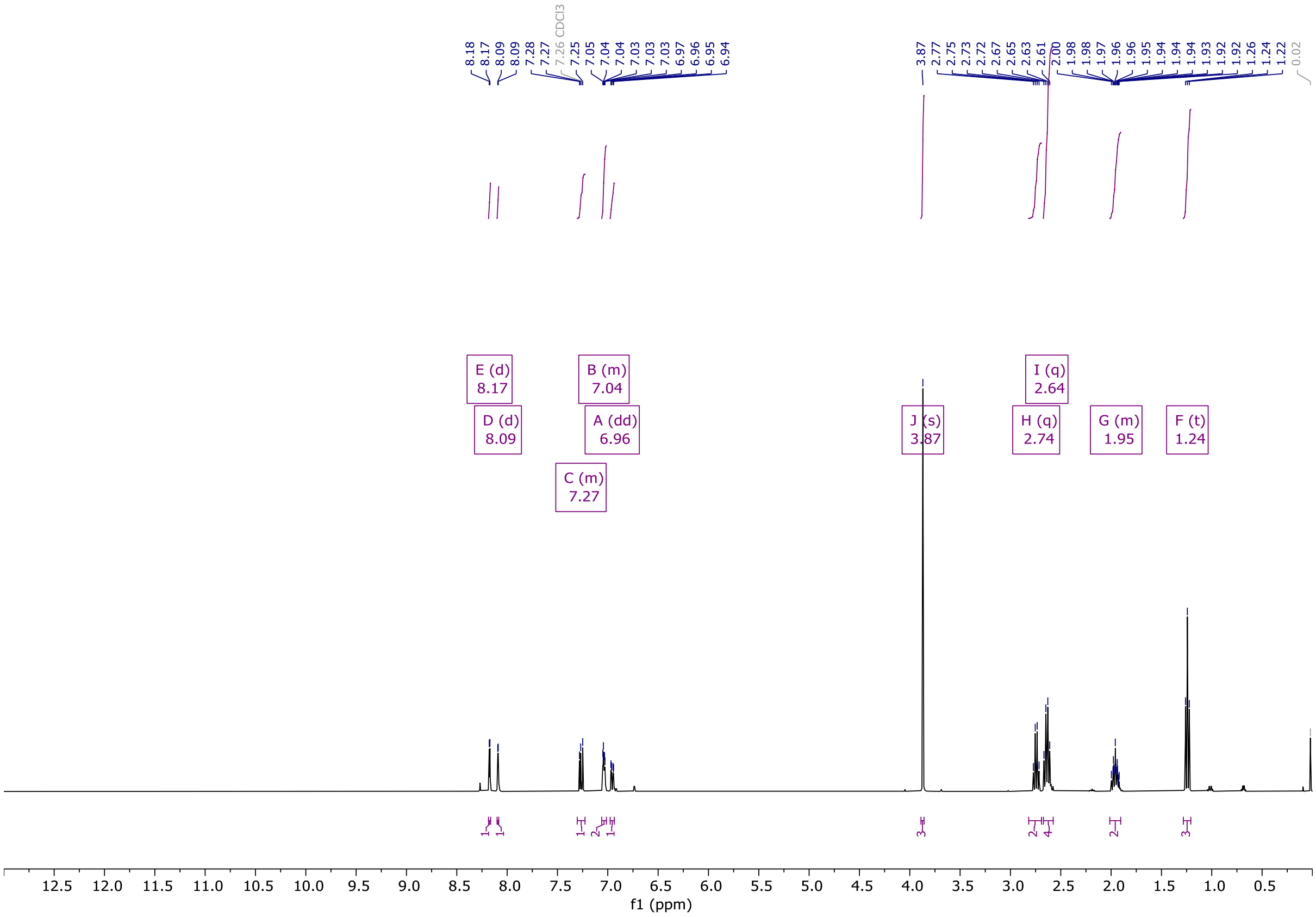

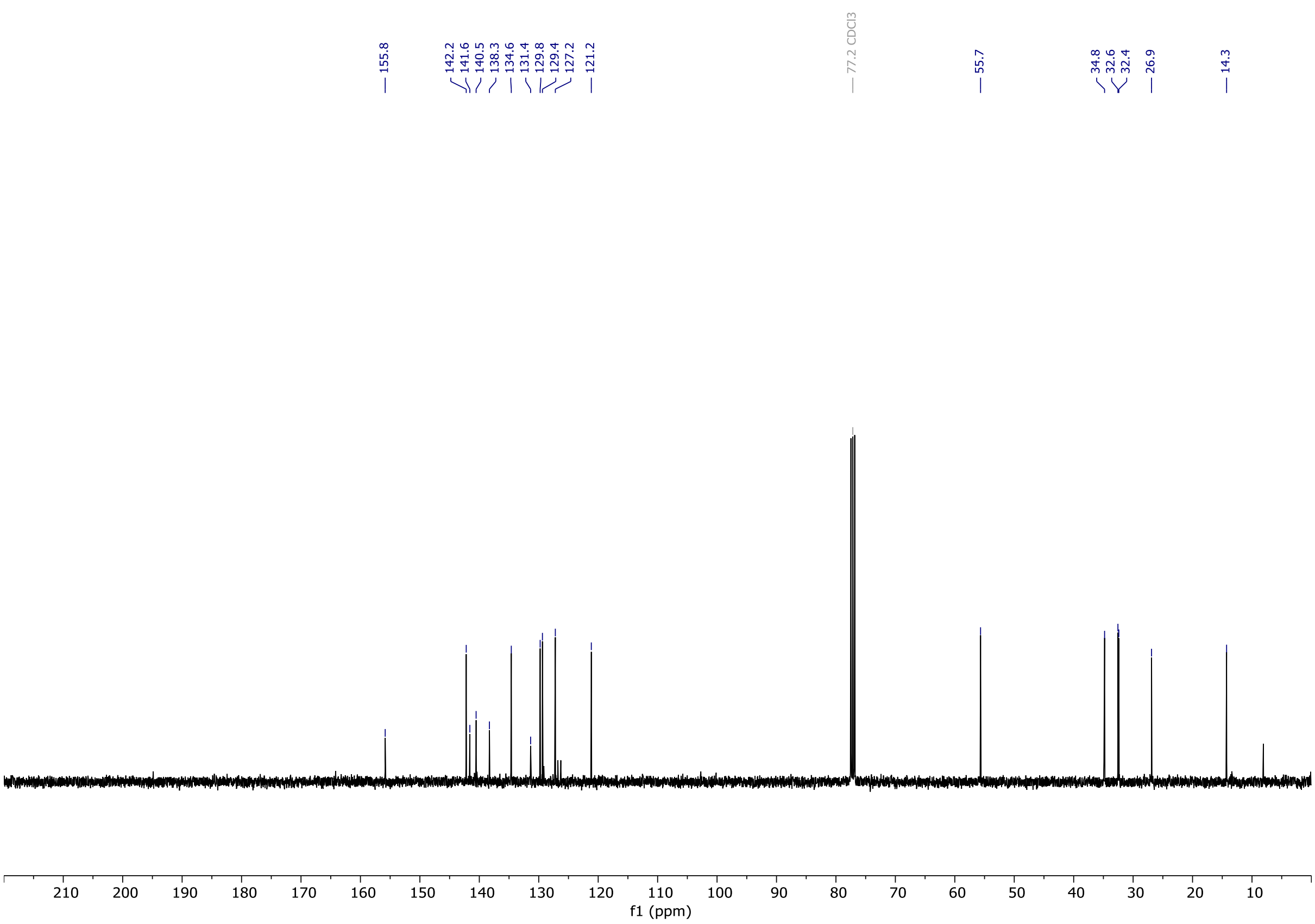

OX04961c

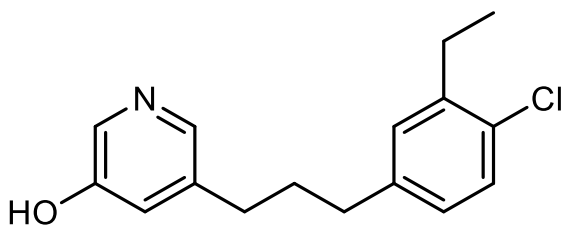

OX04961c

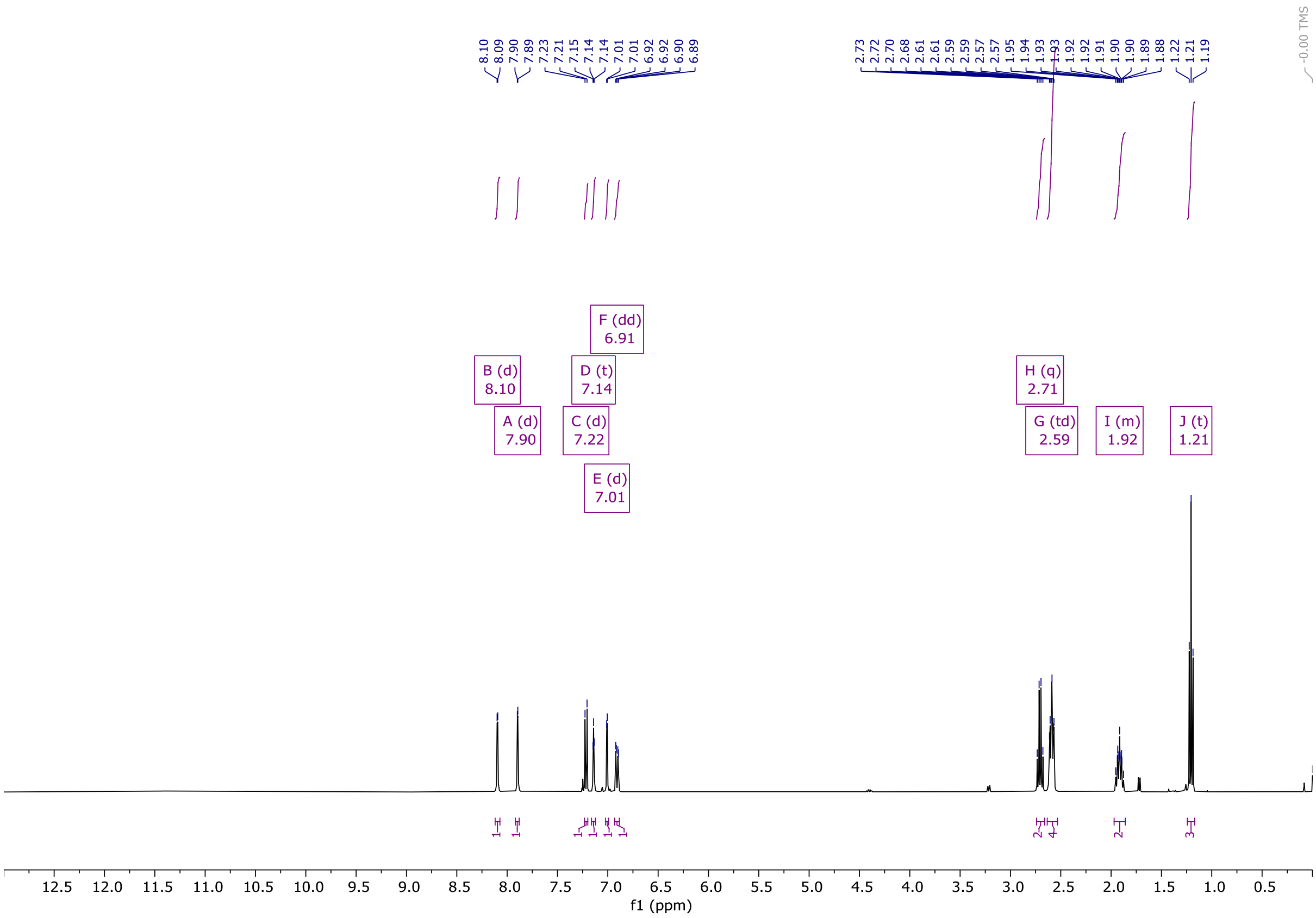

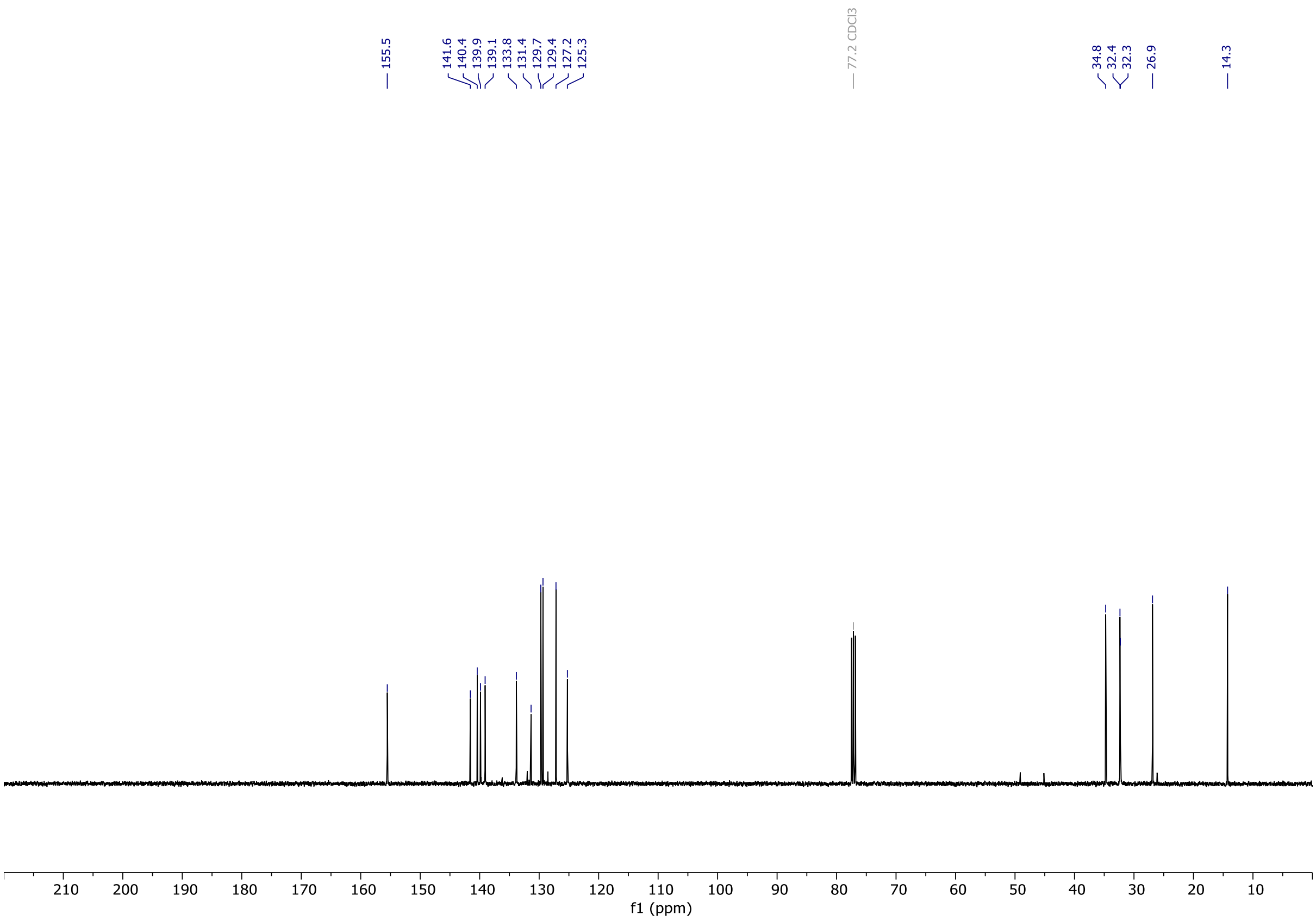

OX04961

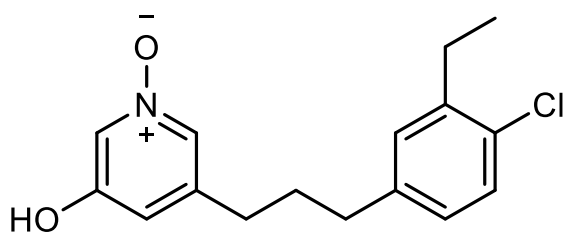

OX04961

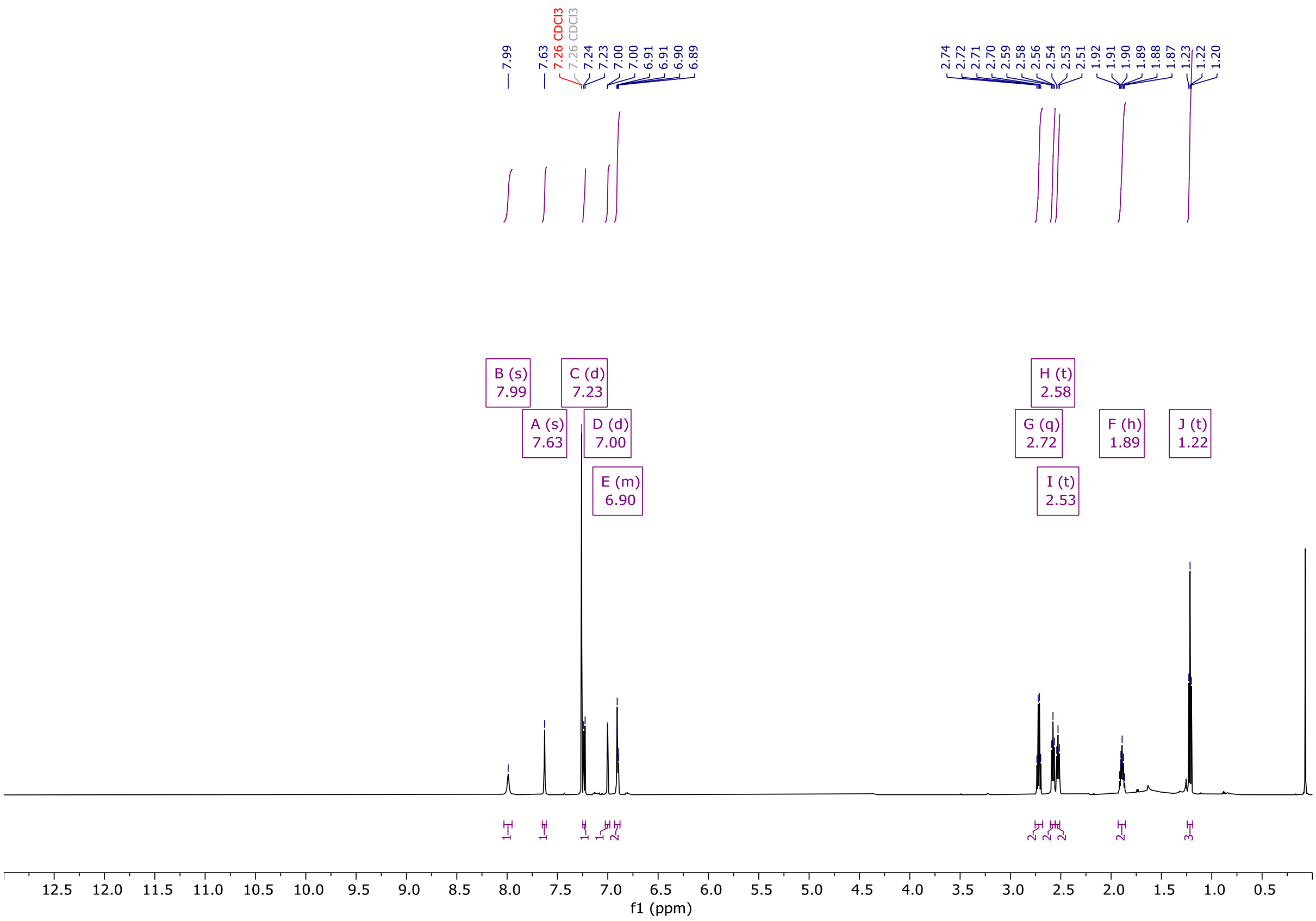

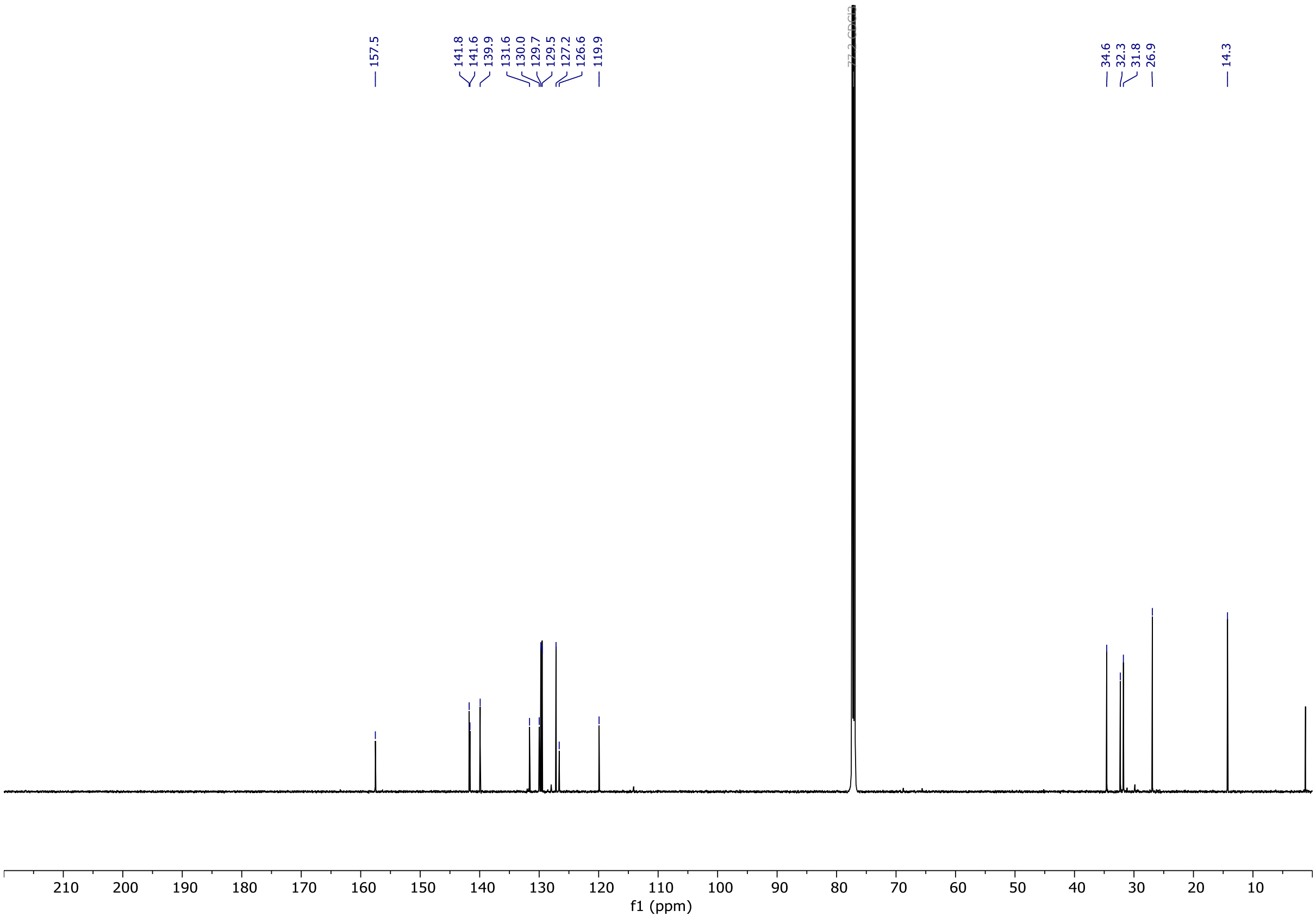

OX04962a

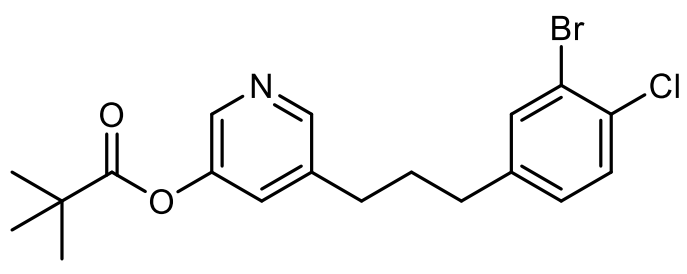

OX04962a

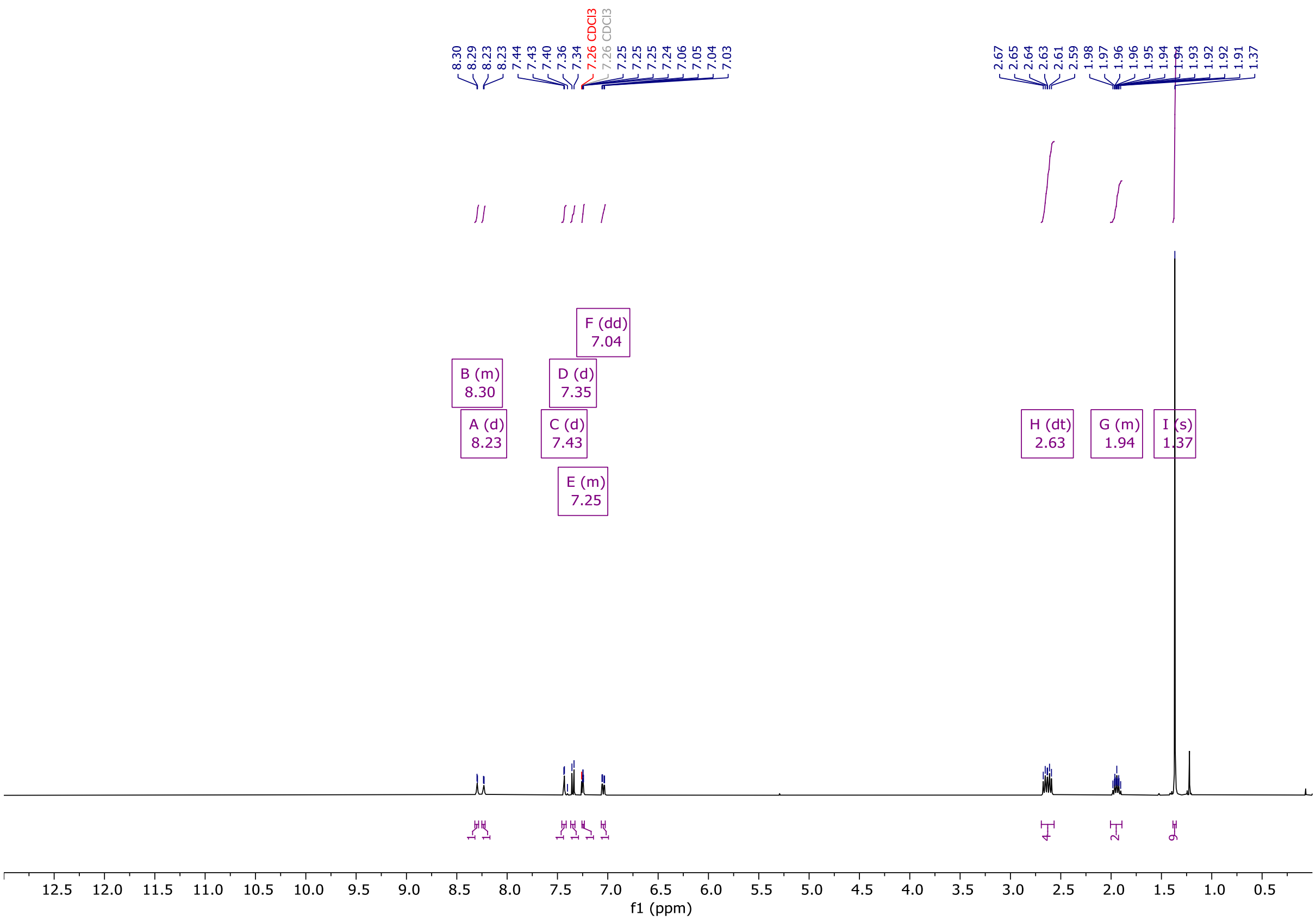

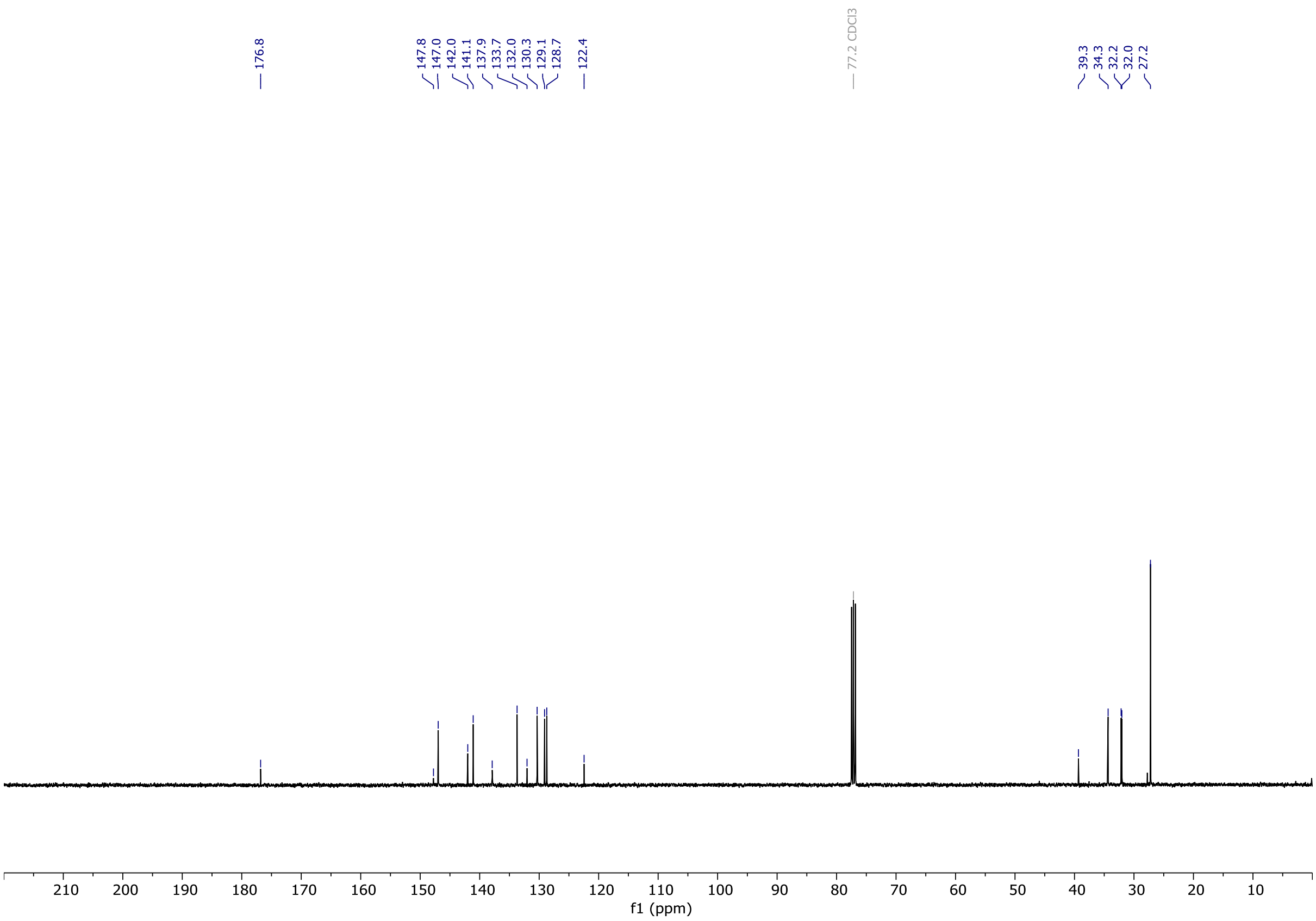

OX04962b

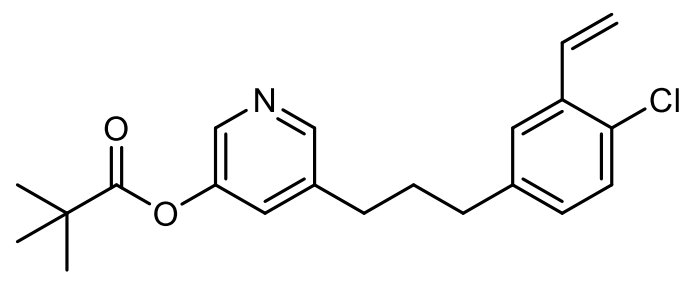

OX04962b

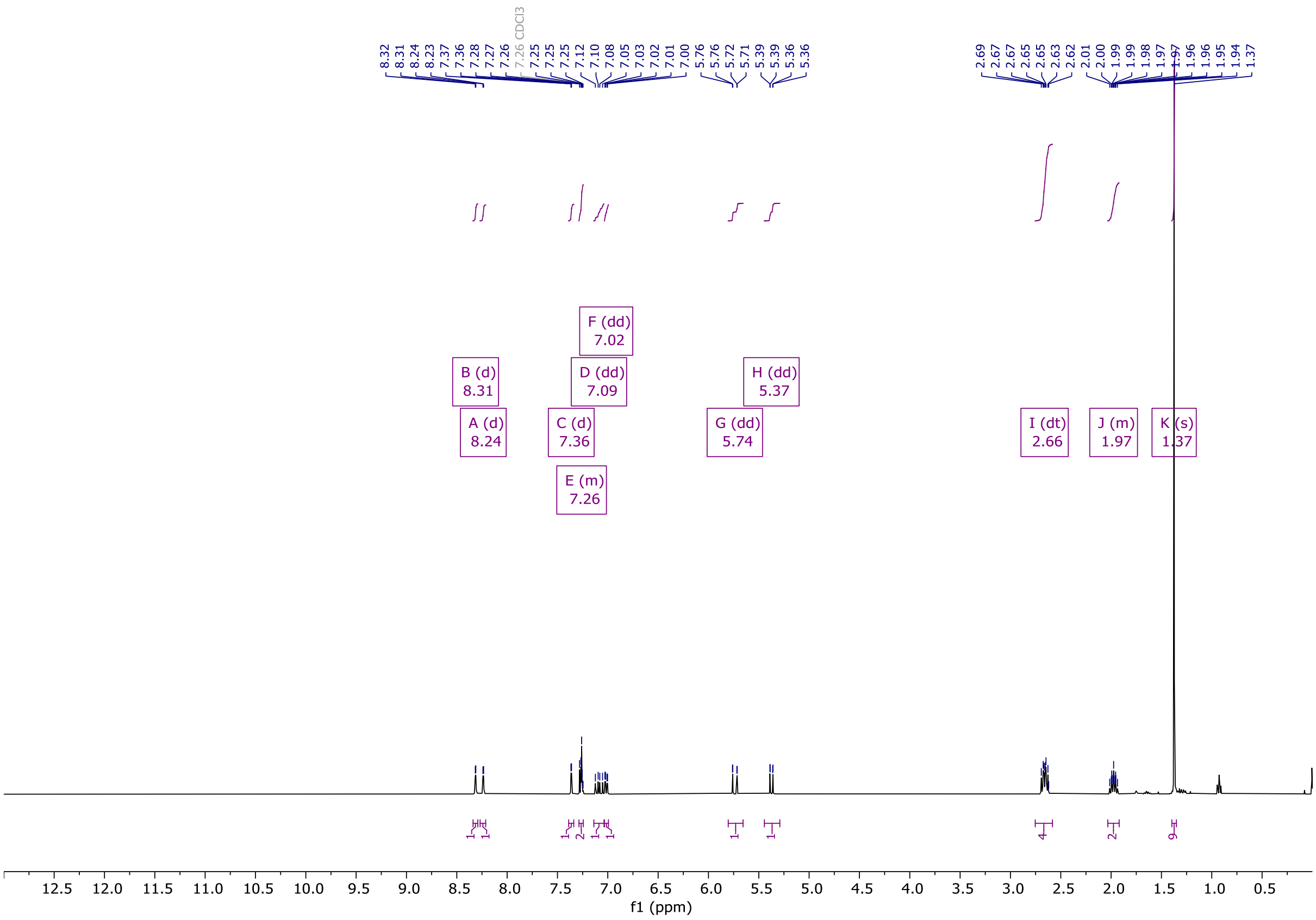

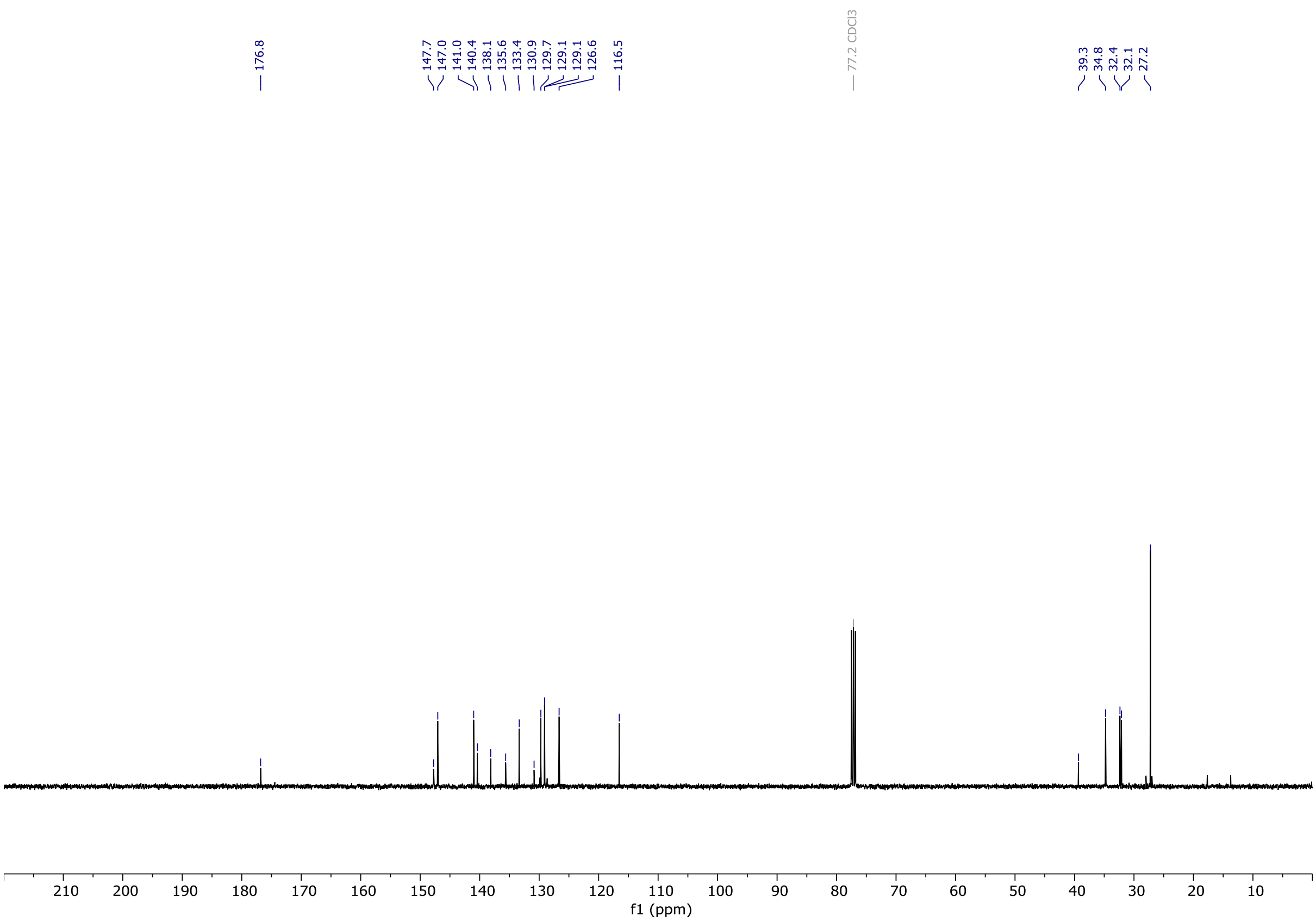

OX04962

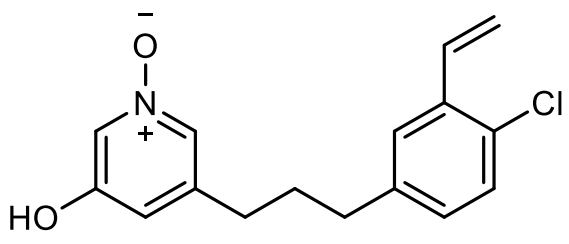

OX04962

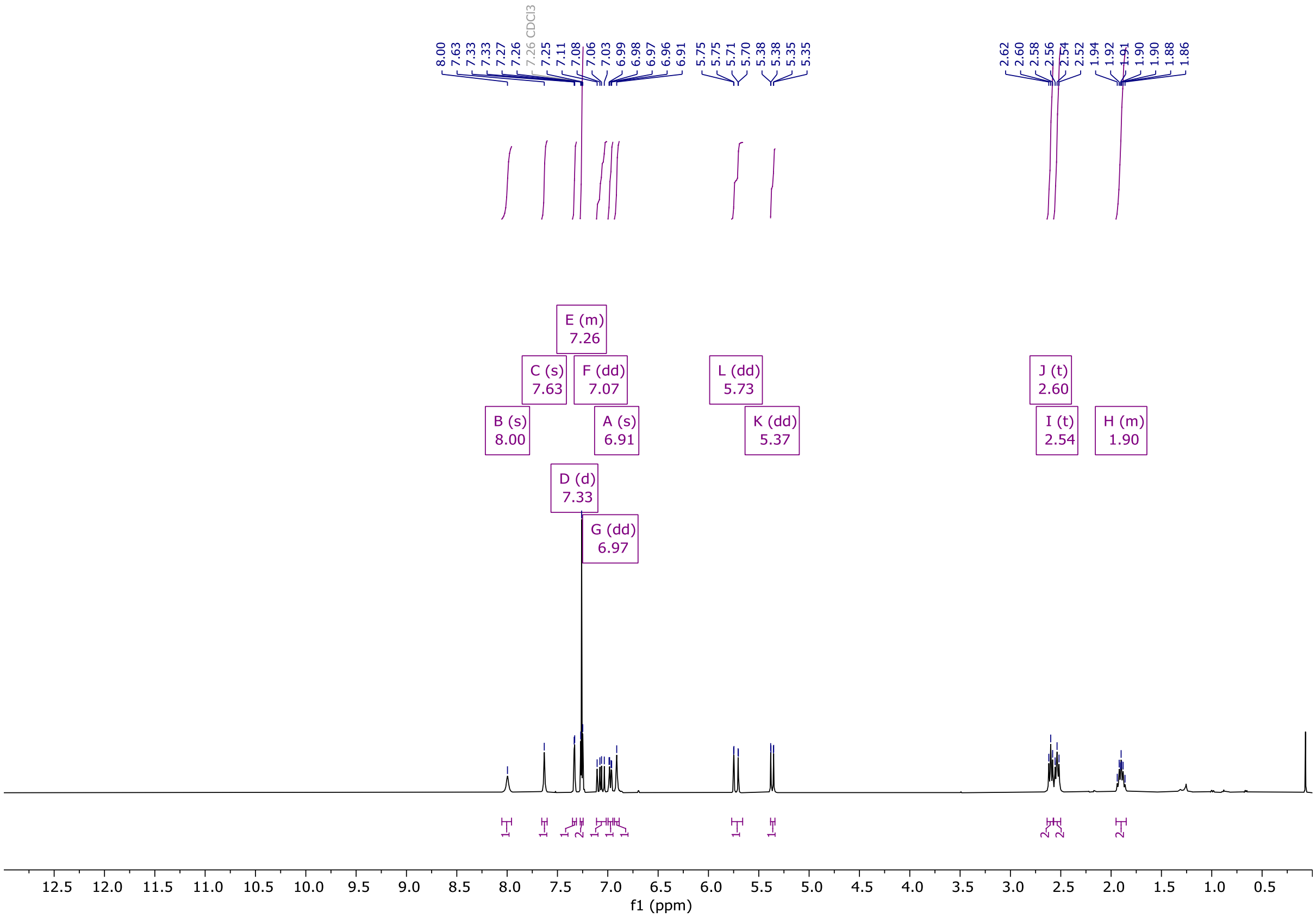

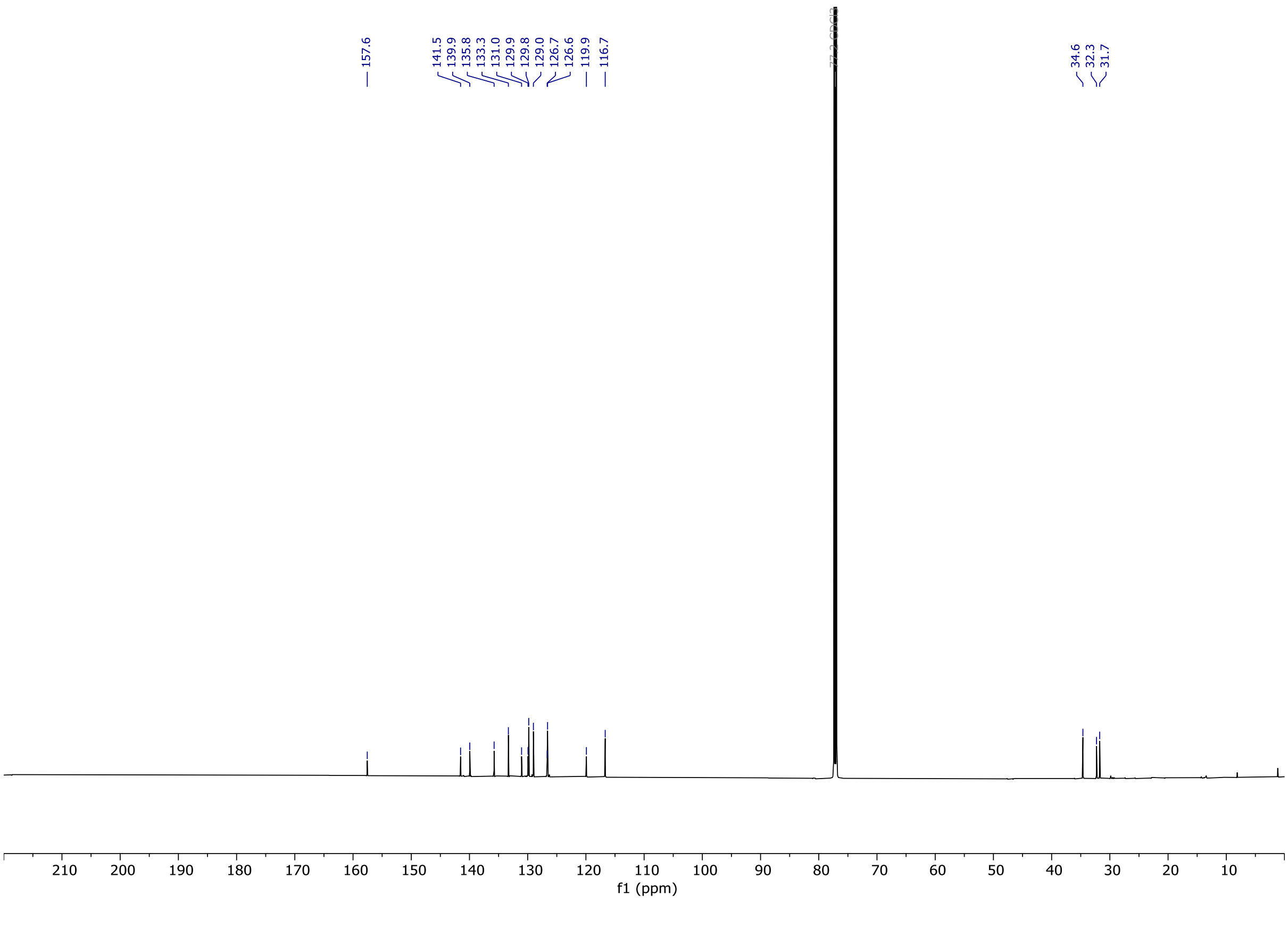

OX04963a

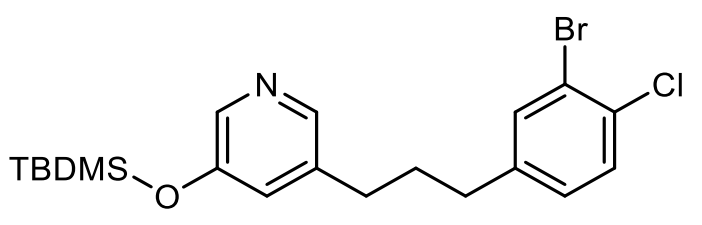

OX04963a

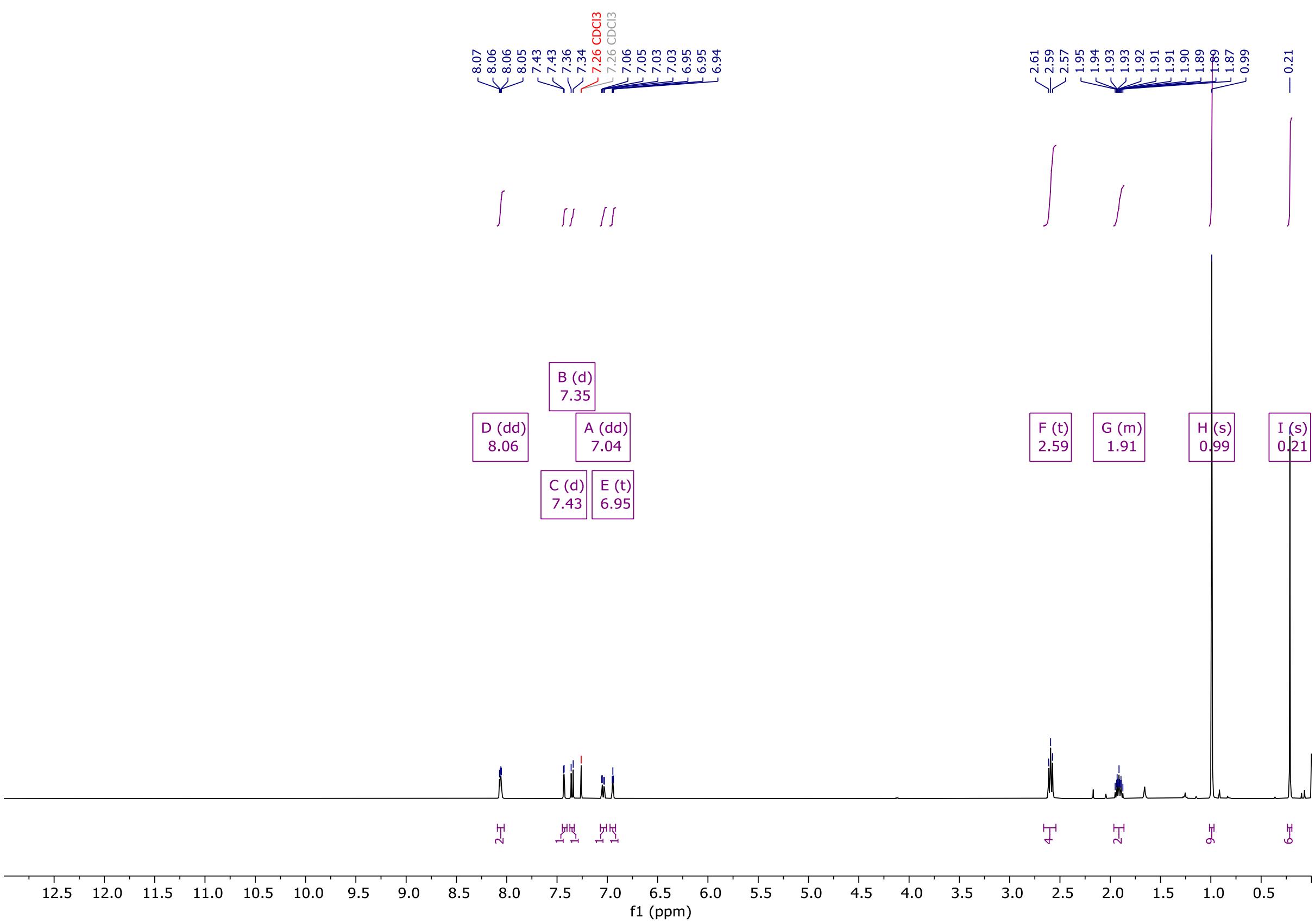

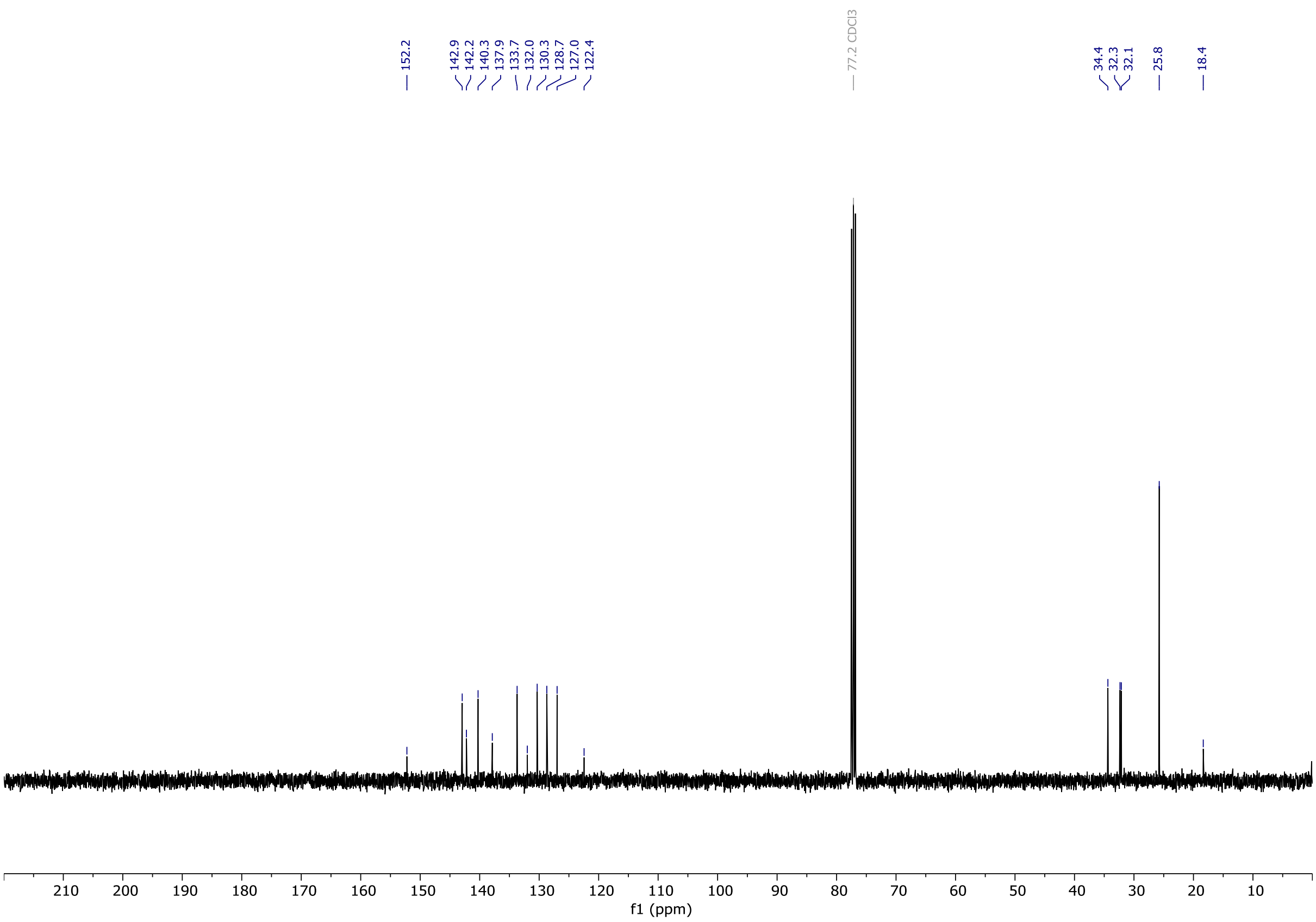

OX04963b

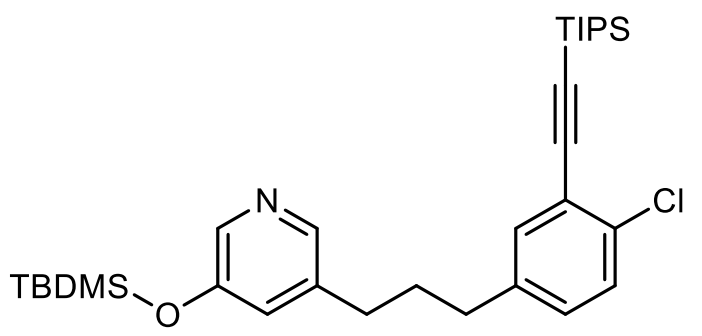

OX04963b

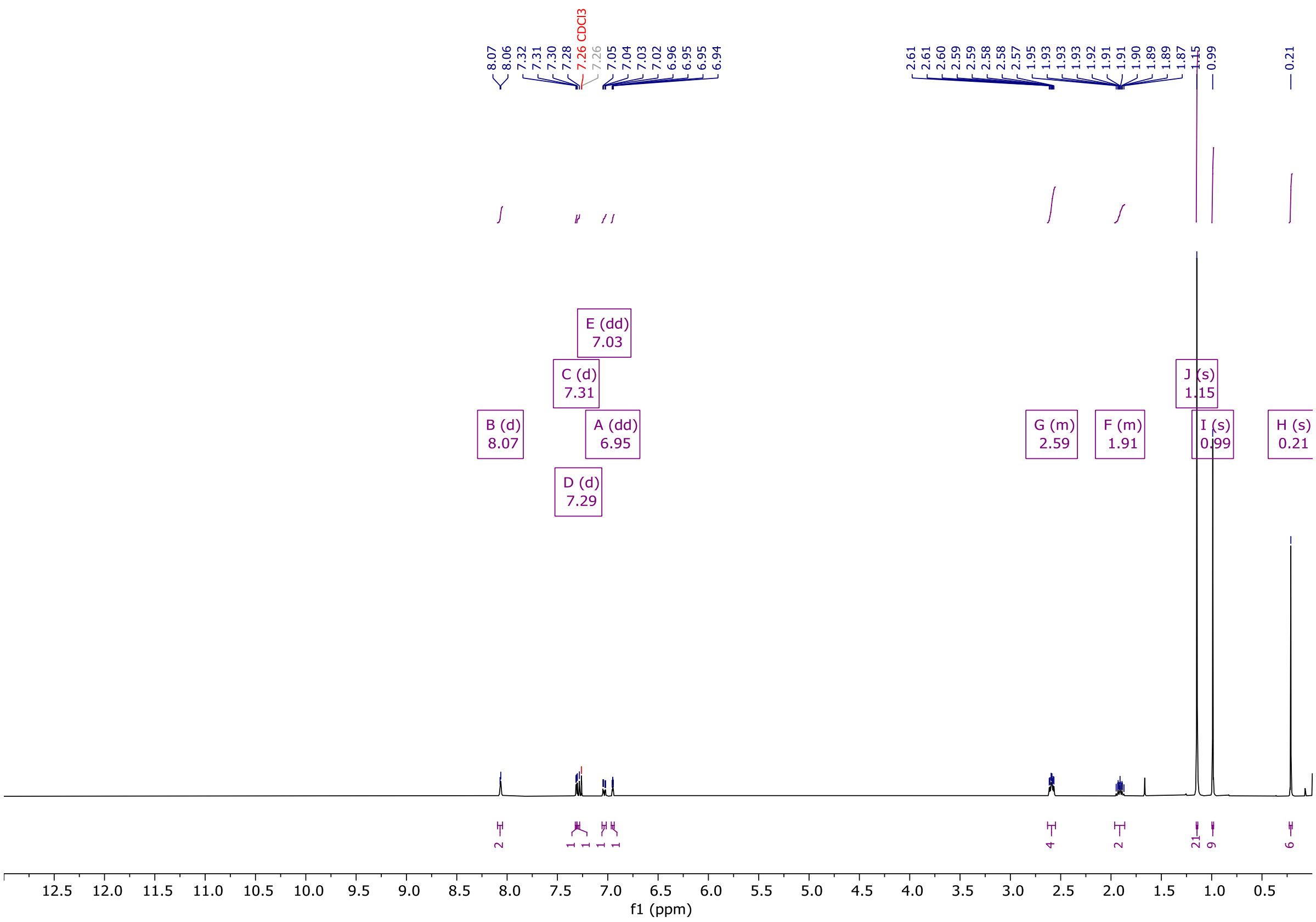

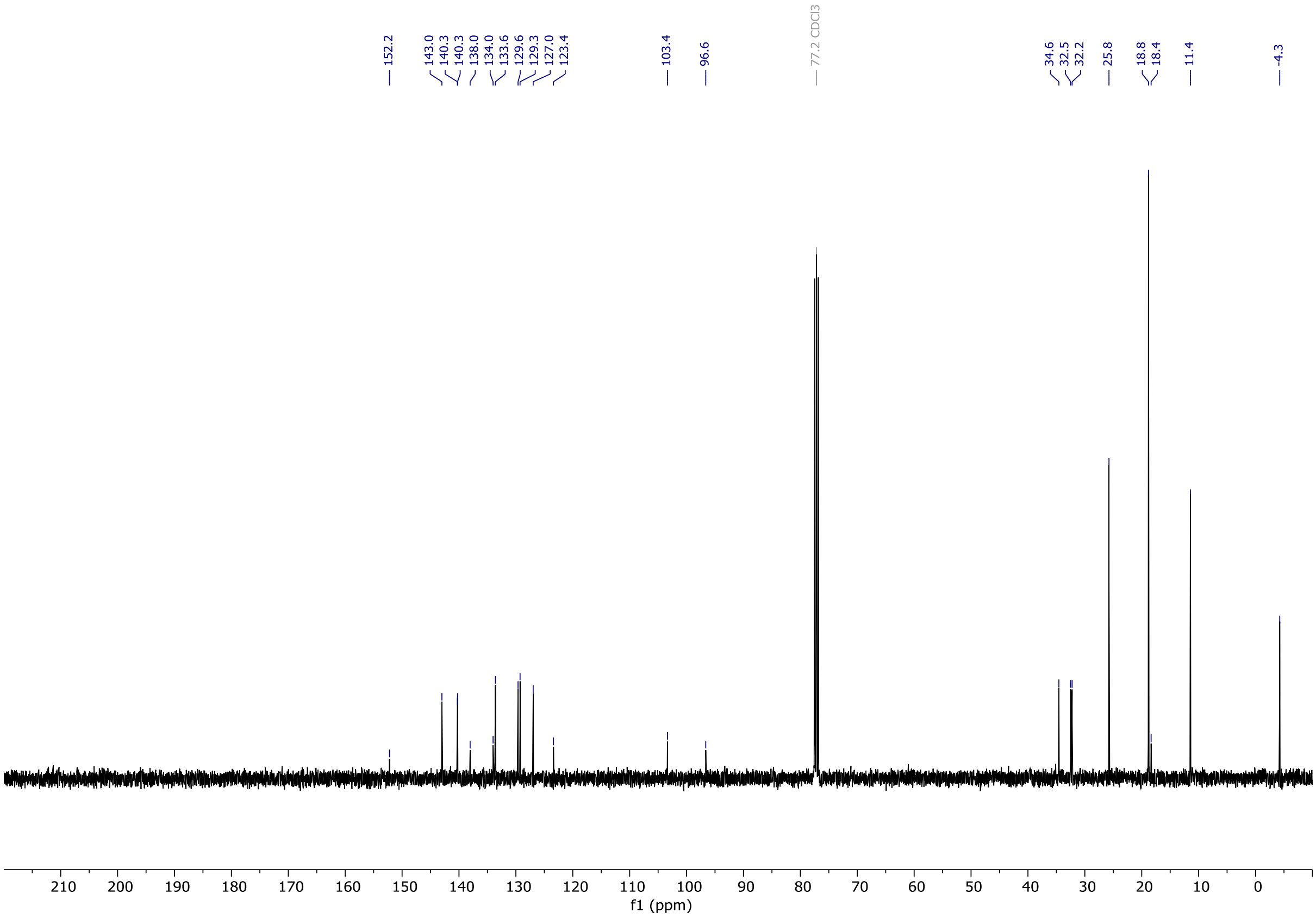

OX04963

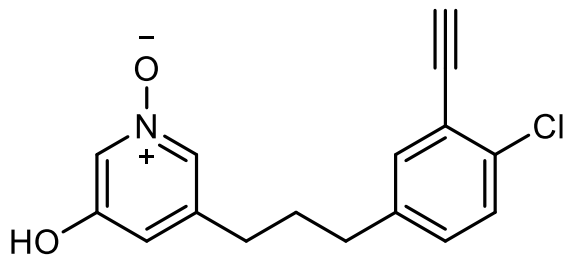

OX04963

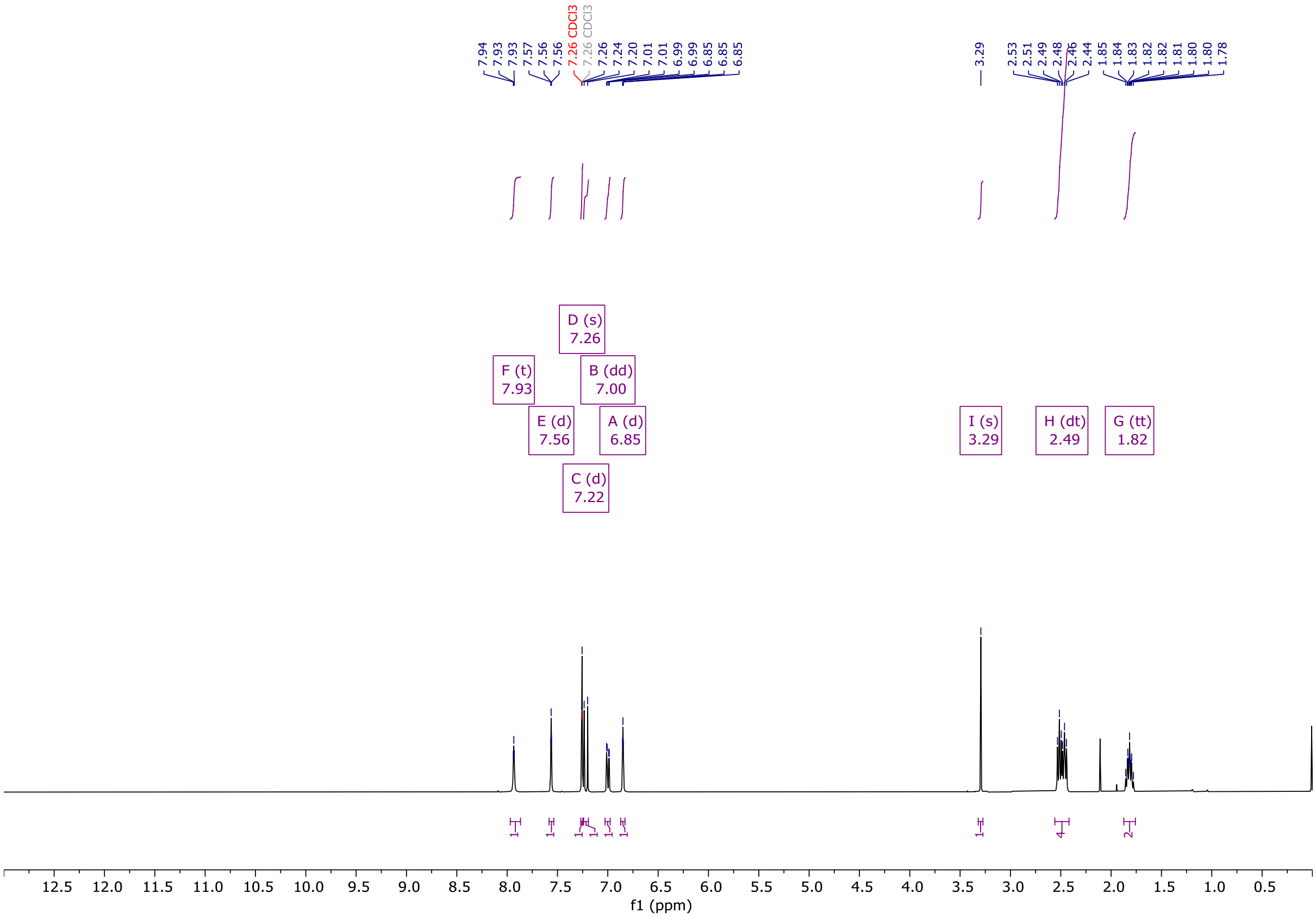

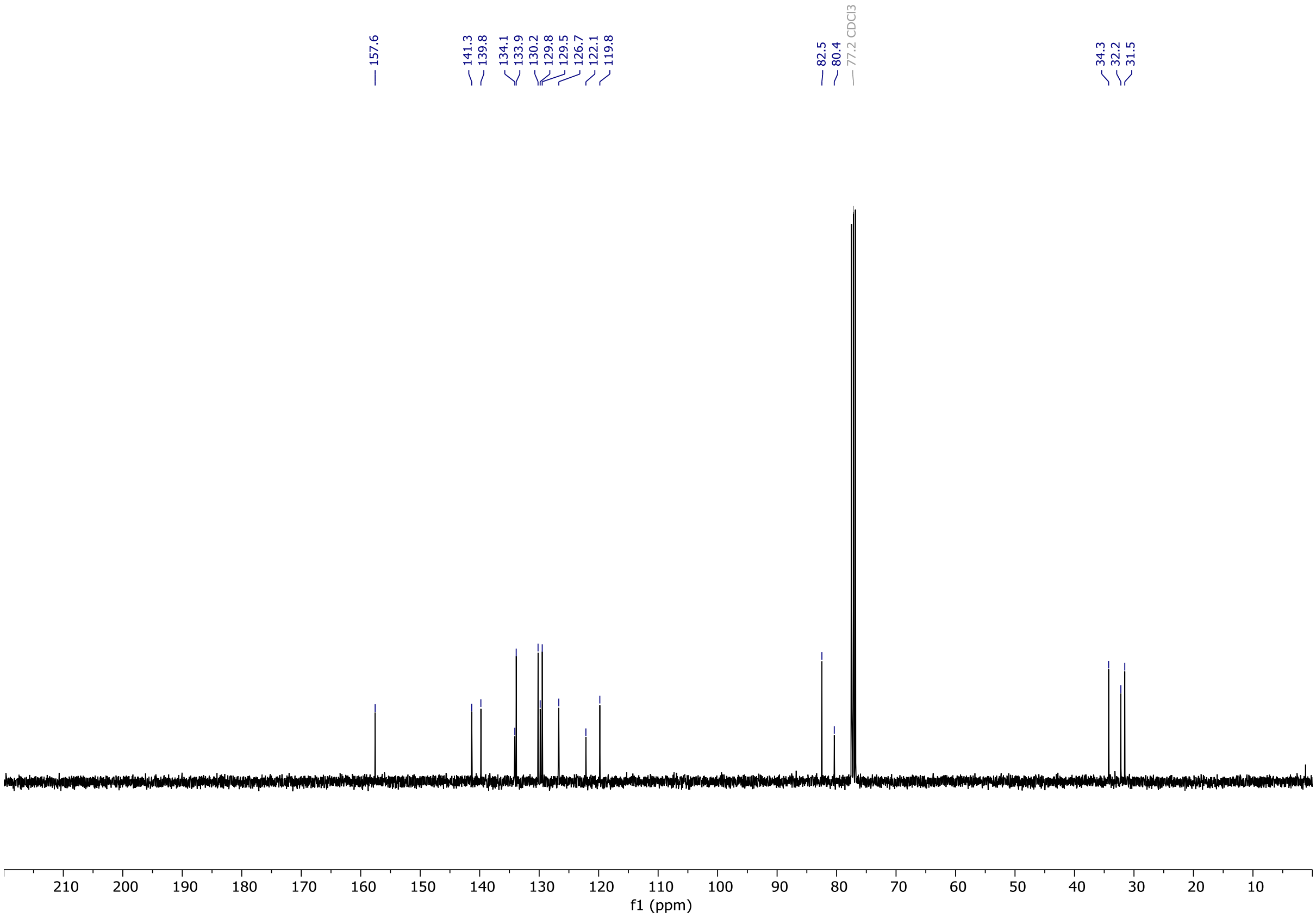

OX04964a

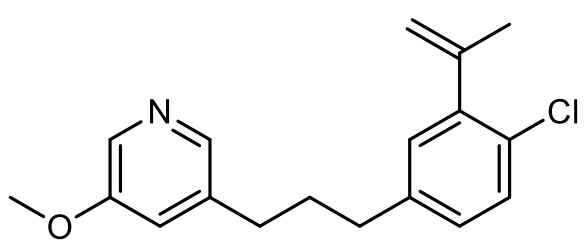

OX04964a

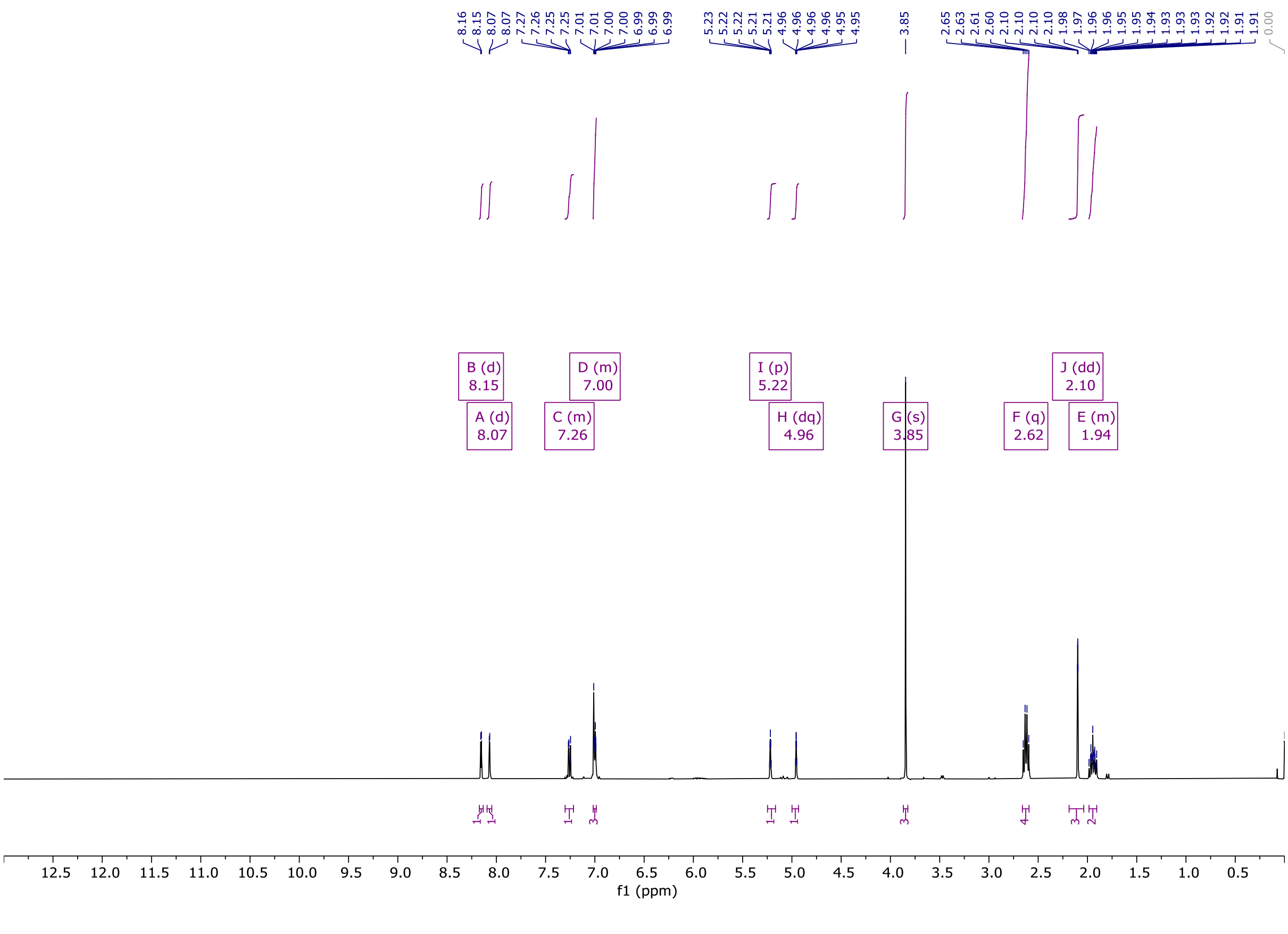

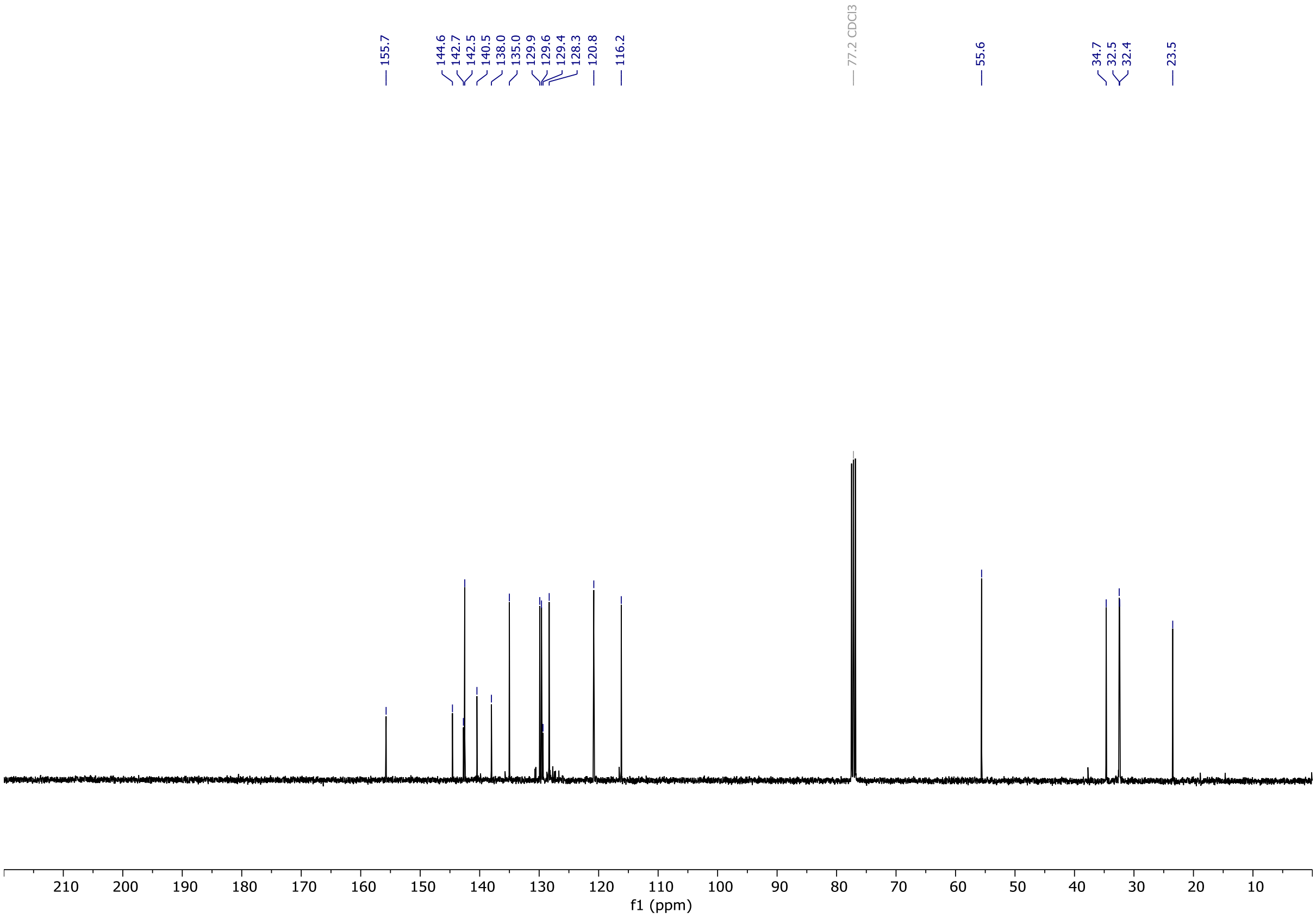

OX04964b

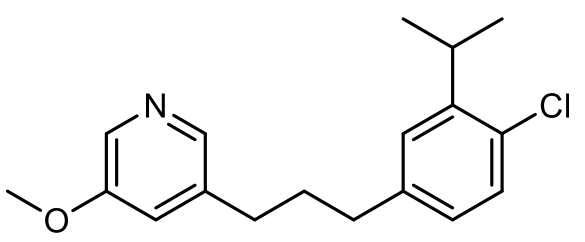

OX04964b

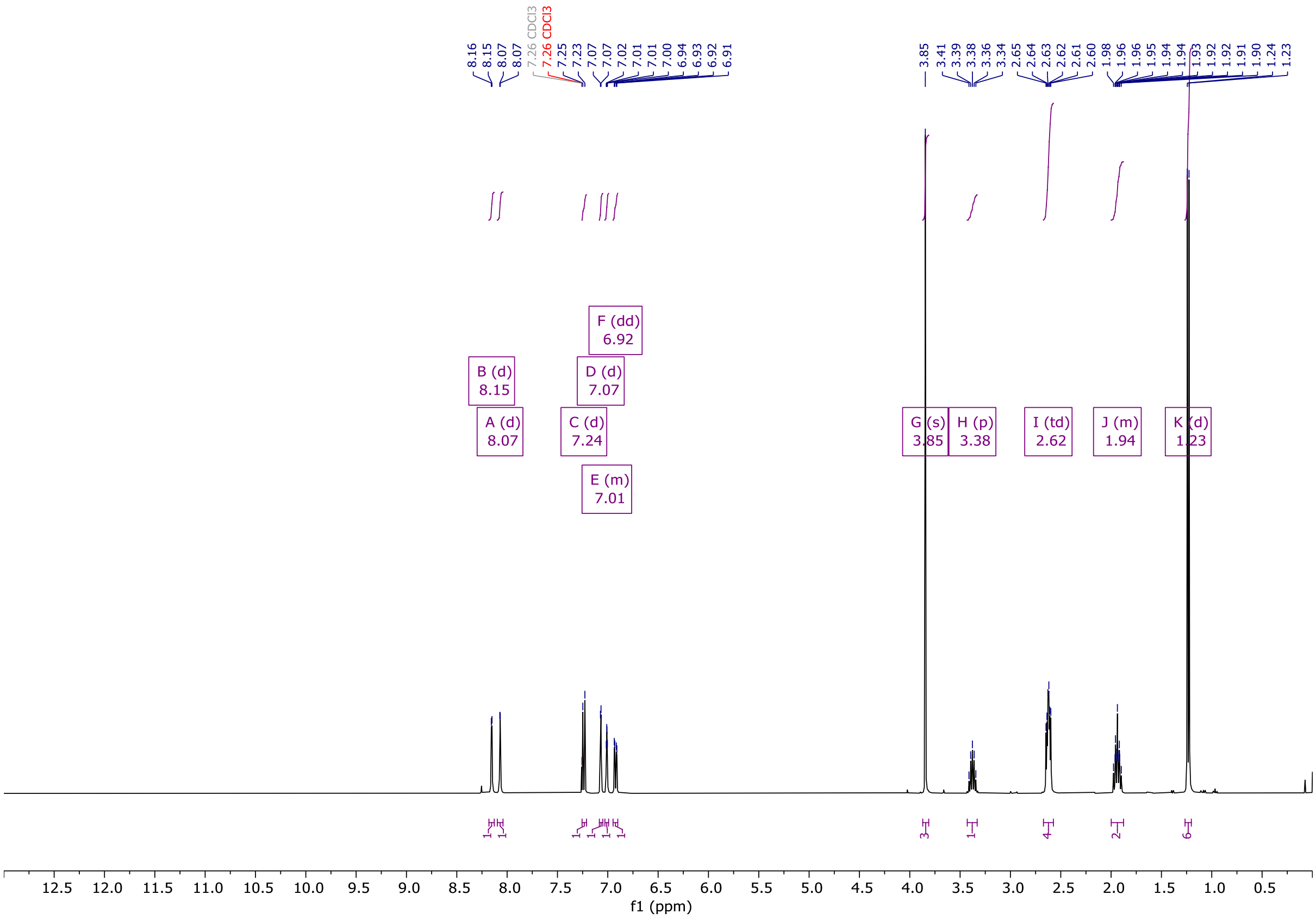

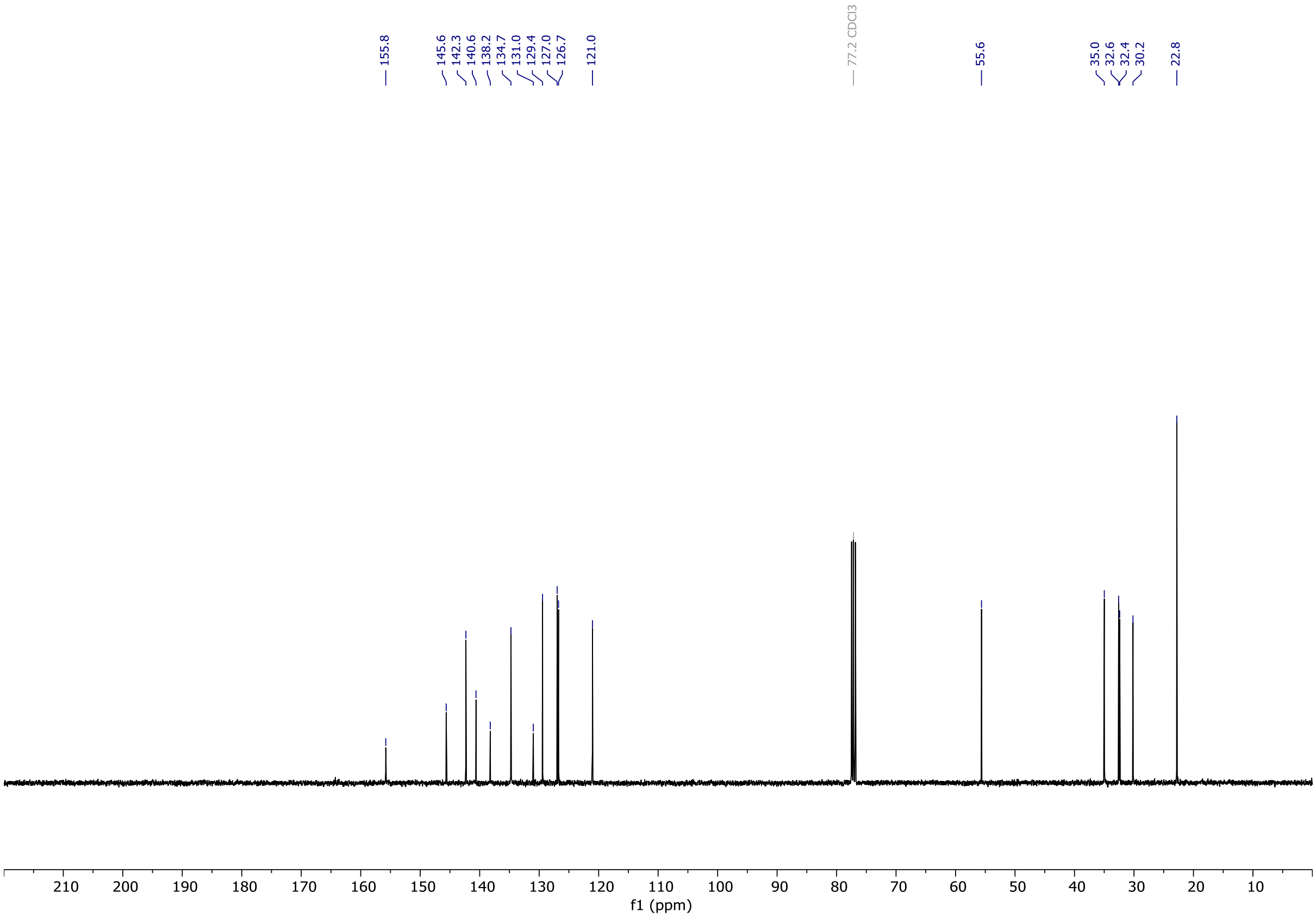

OX04964c

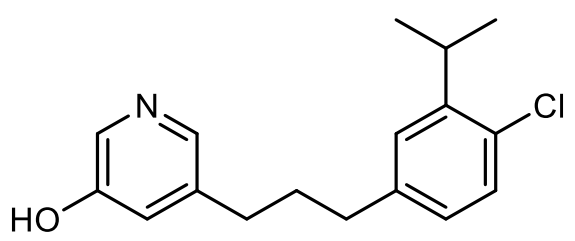

OX04964c

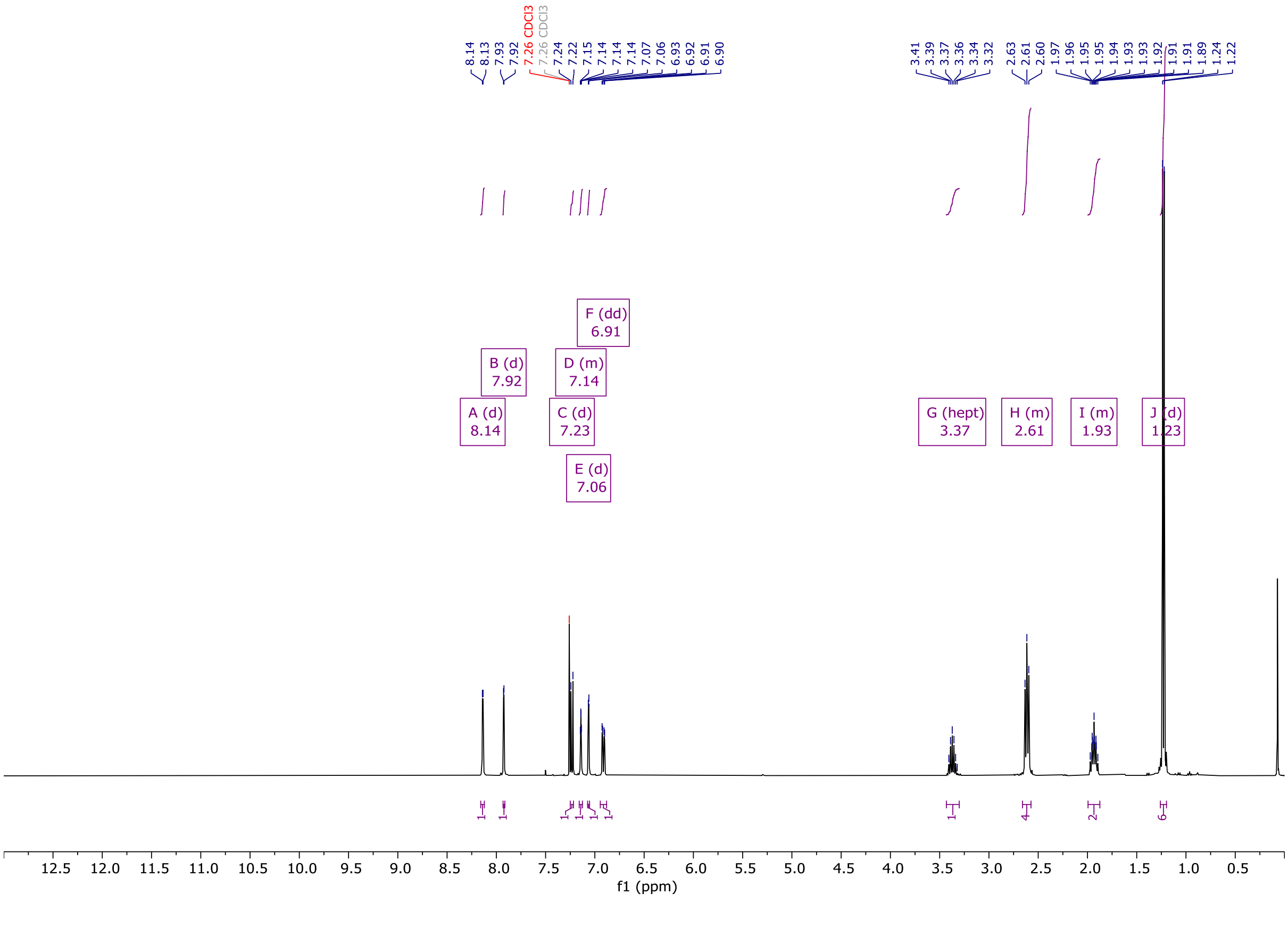

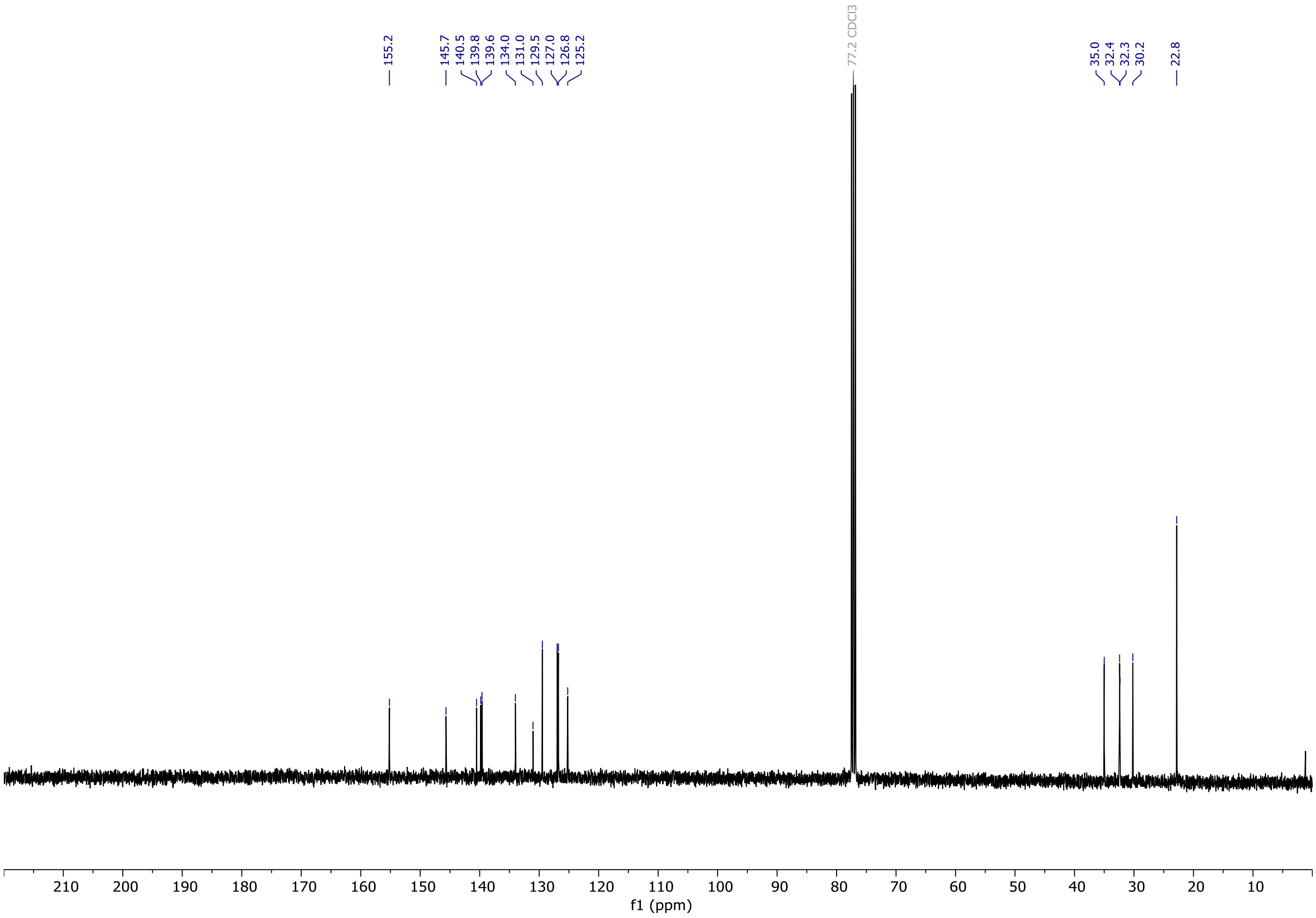

OX04964

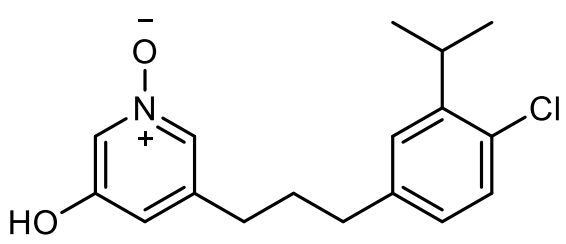

OX04964

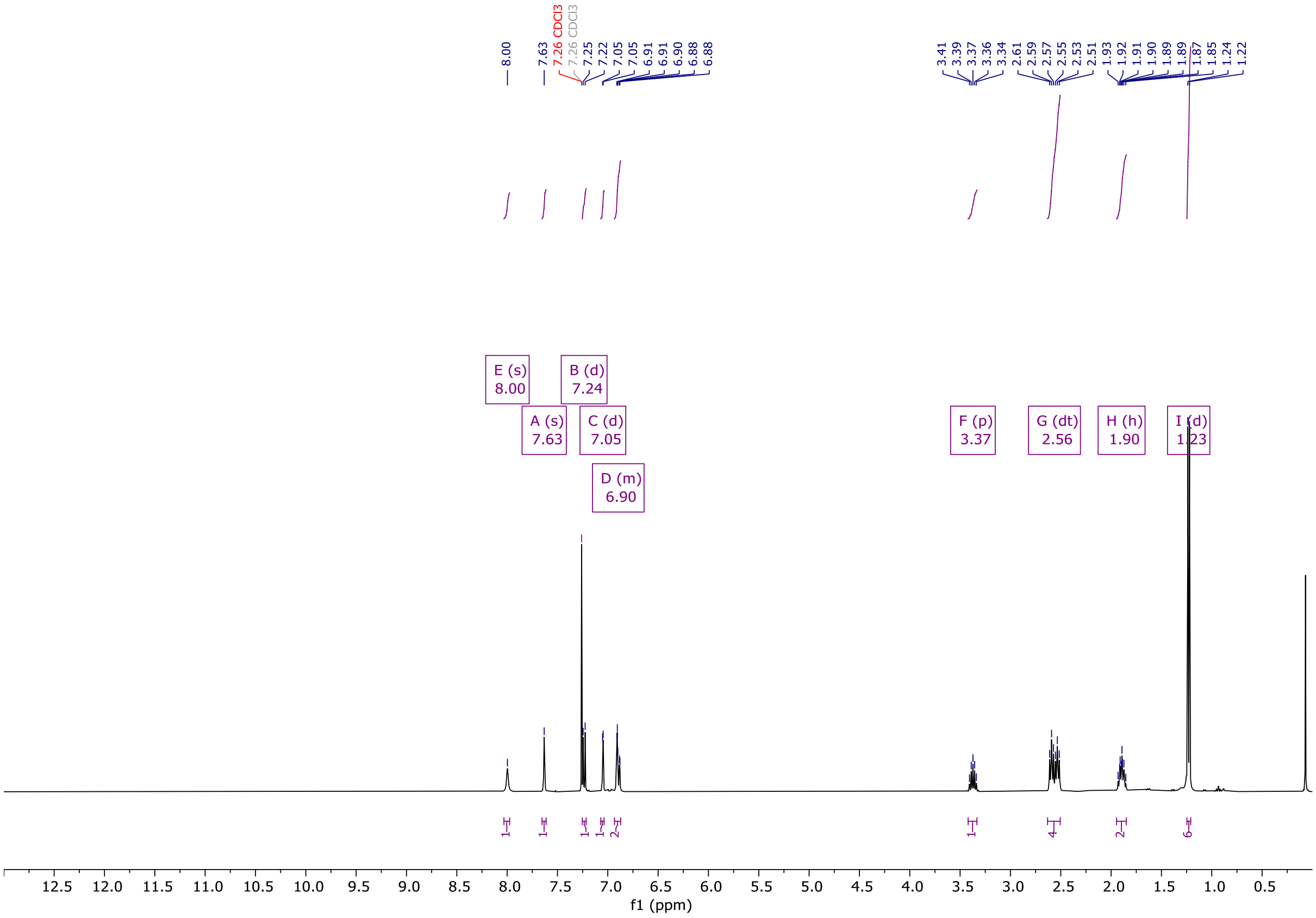

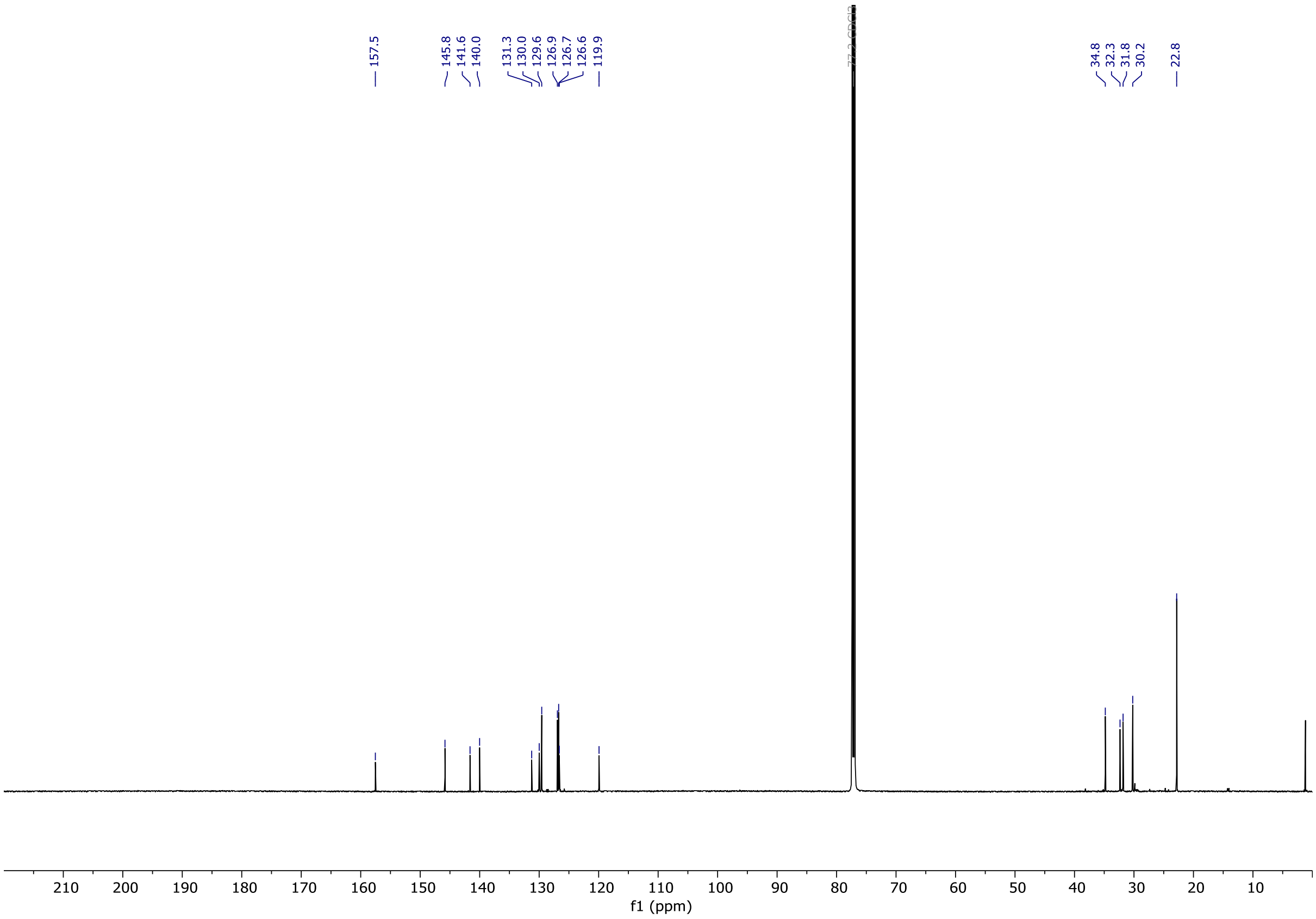

OX04965a

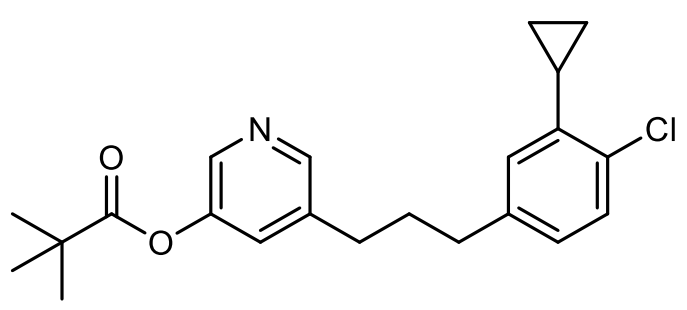

OX04965a

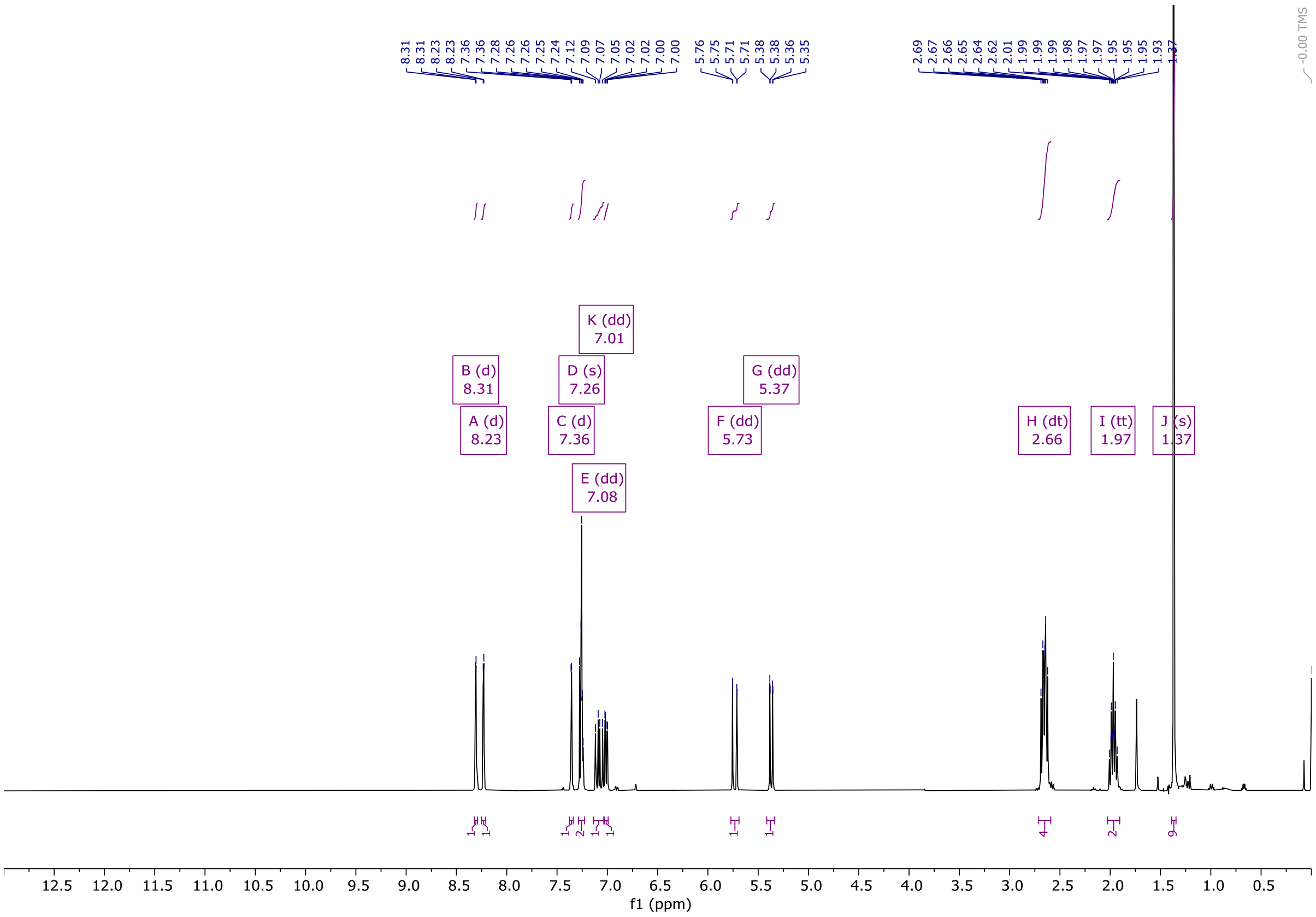
8.31 8.31 8.23 8.23 7.36 7.36 7.28 7.26 7.26 7.25 7.24 7.12 7.09 7.07 7.05 7.02 7.00 7.00
5.76 5.75 5.71 5.71 5.38 5.38 5.36 5.35
2.69 2.67 2.66 2.65 2.64 2.62 2.01 1.99 1.99 1.99 1.98 1.97 1.97 1.95 1.95 1.95 1.93 1.37
-0.00 TMS
K (dd) 7.01
B (d) 8.31
D (s) 7.26
G (dd) 5.37
A (d) 8.23
C (d) 7.36
F (dd) 5.73
H (dt) 2.66
I (tt) 1.97
J (s) 1.37
E (dd) 7.08
f1 (ppm)

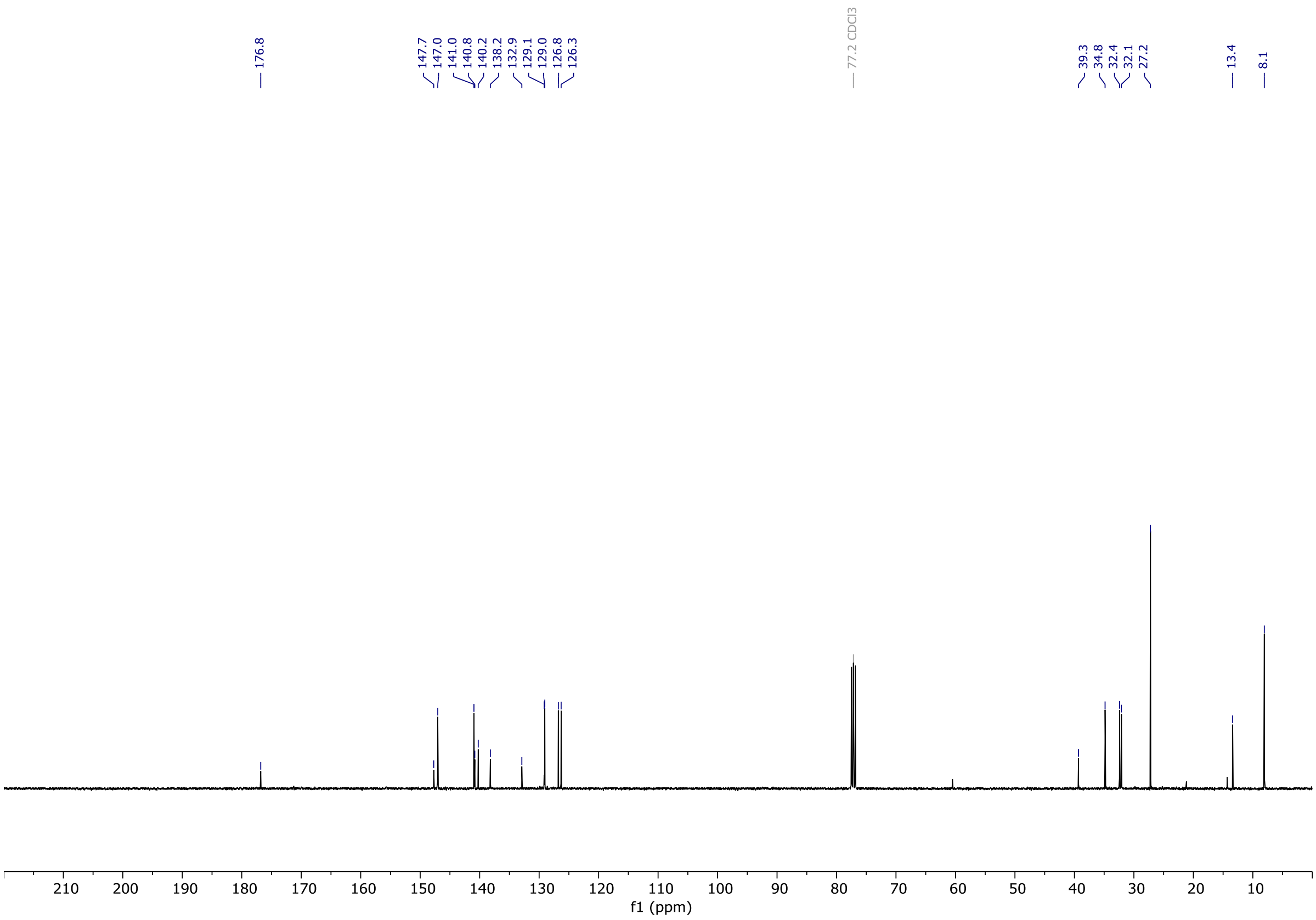
176.8
147.7 147.0 141.0 140.8 140.2 138.2 132.9 129.1 129.0 126.8 126.3
77.2 CDCl3
39.3 34.8 32.4 32.1 27.2
13.4
8.1
f1 (ppm)

OX04965

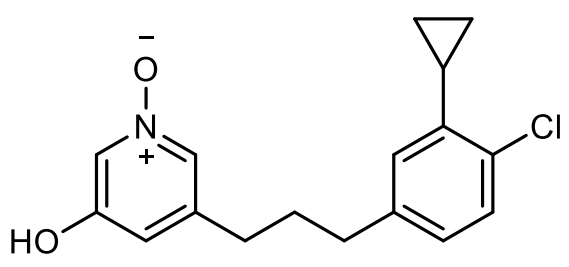

OX04965

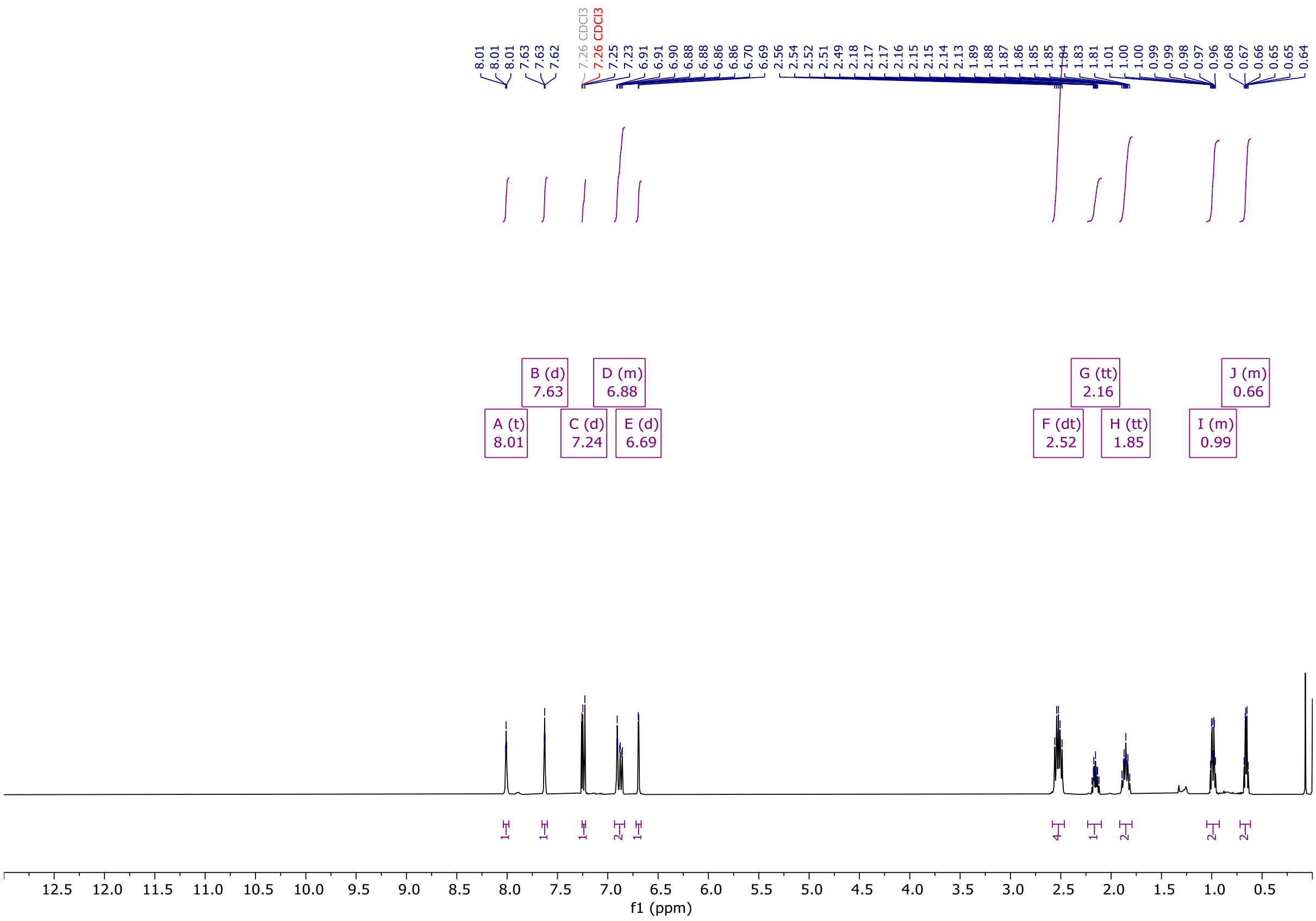

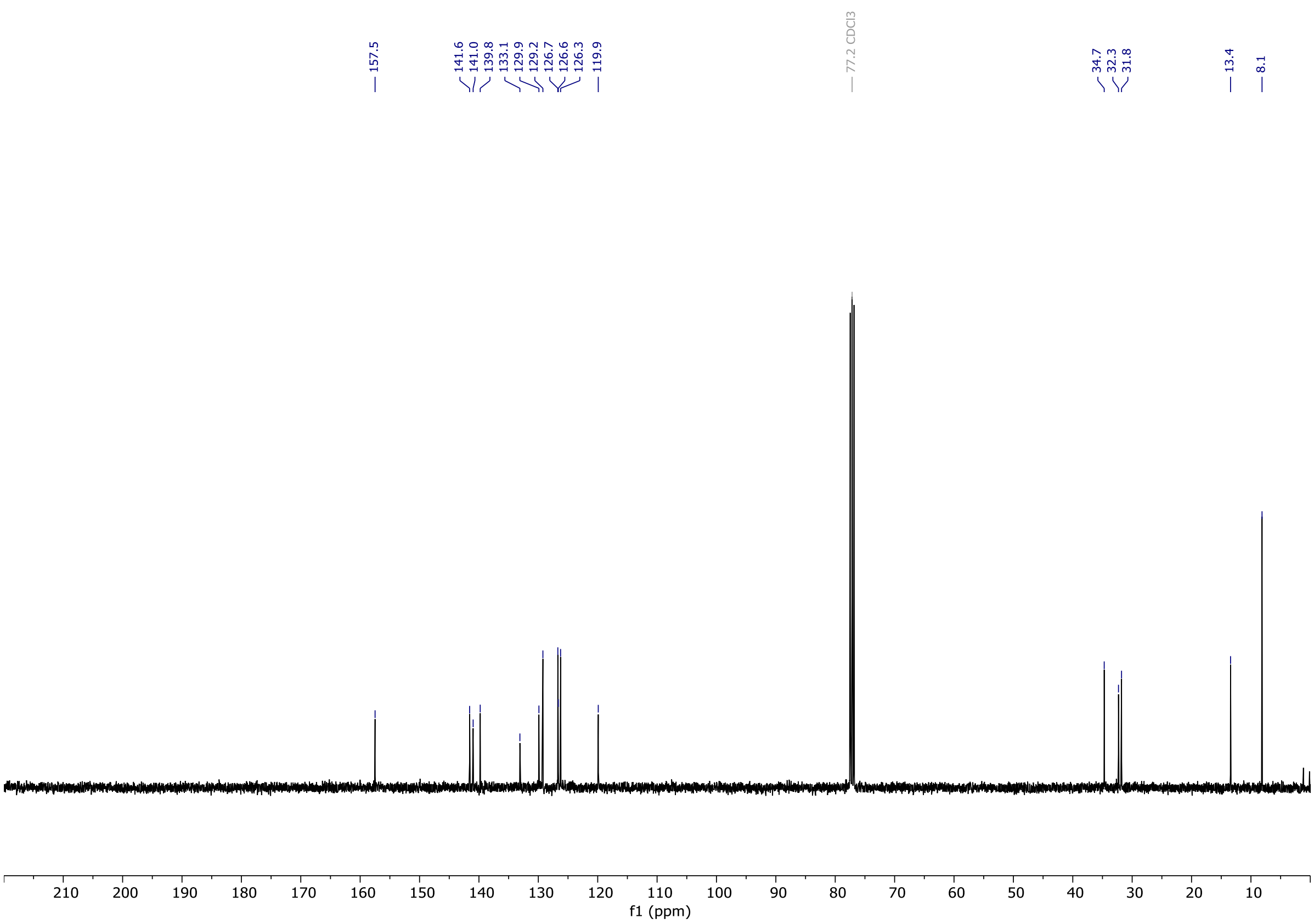

OX04966a

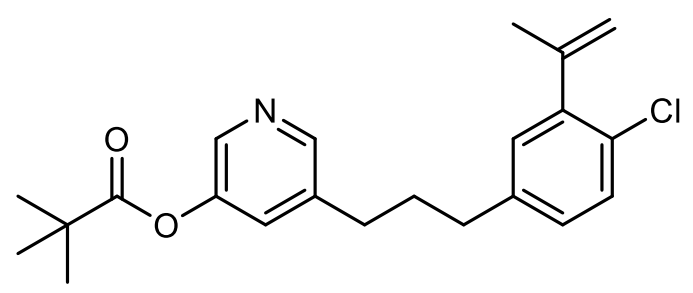
N
O
O
Cl

OX04966a

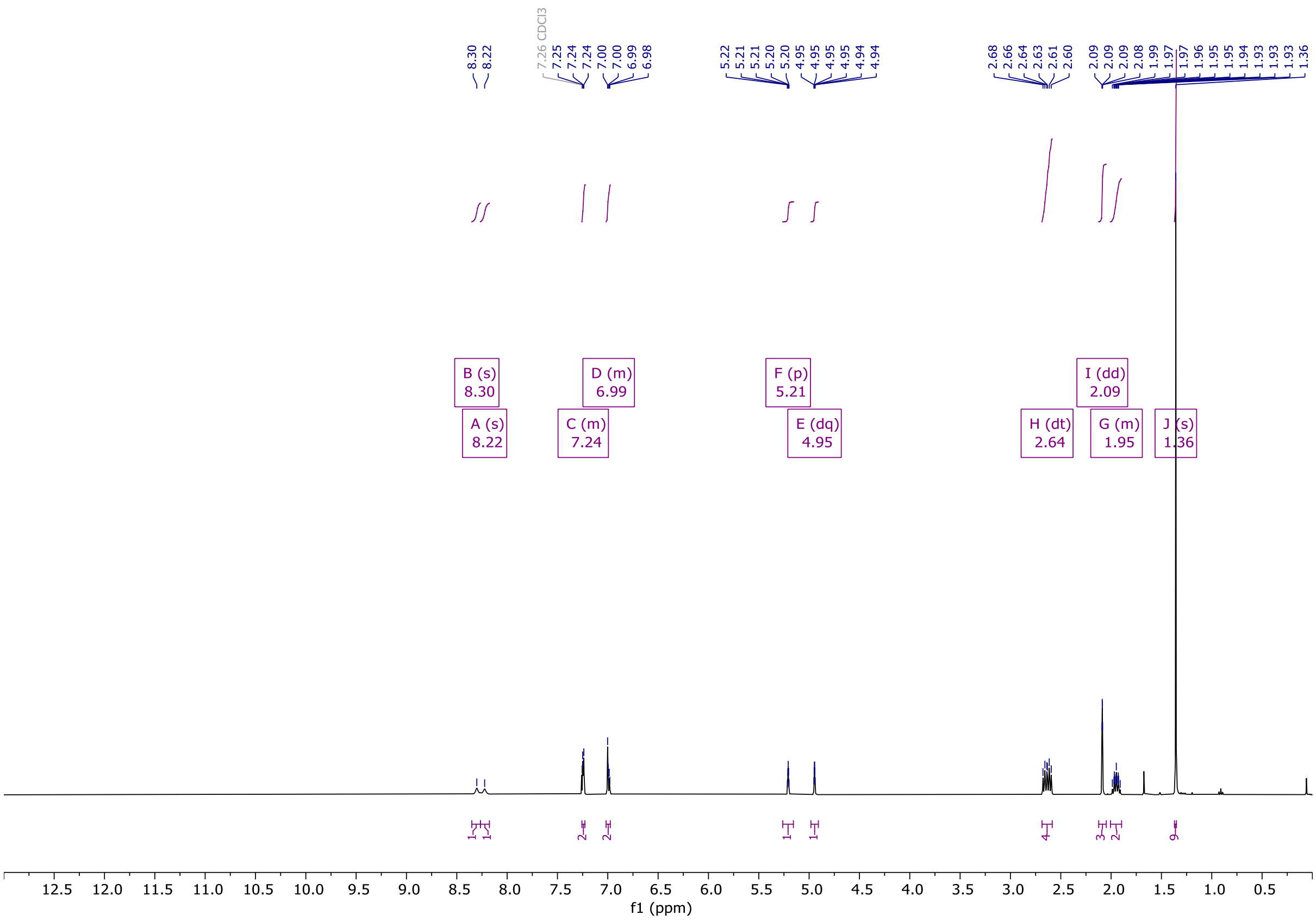
8.30
8.22
7.26 CDCl3
7.25
7.24
7.24
7.00
7.00
6.99
6.98
5.22
5.21
5.21
5.20
5.20
4.95
4.95
4.95
4.95
4.94
4.94
2.68
2.66
2.64
2.63
2.61
2.60
2.09
2.09
2.09
2.09
2.08
1.99
1.97
1.97
1.96
1.95
1.95
1.94
1.93
1.93
1.93
1.36
B (s) 8.30
A (s) 8.22
D (m) 6.99
C (m) 7.24
F (p) 5.21
E (dq) 4.95
H (dt) 2.64
I (dd) 2.09
G (m) 1.95
J (s) 1.36
1
1
2
2
1
1
4
3
2
9
12.5 12.0 11.5 11.0 10.5 10.0 9.5 9.0 8.5 8.0 7.5 7.0 6.5 6.0 5.5 5.0 4.5 4.0 3.5 3.0 2.5 2.0 1.5 1.0 0.5
f1 (ppm)

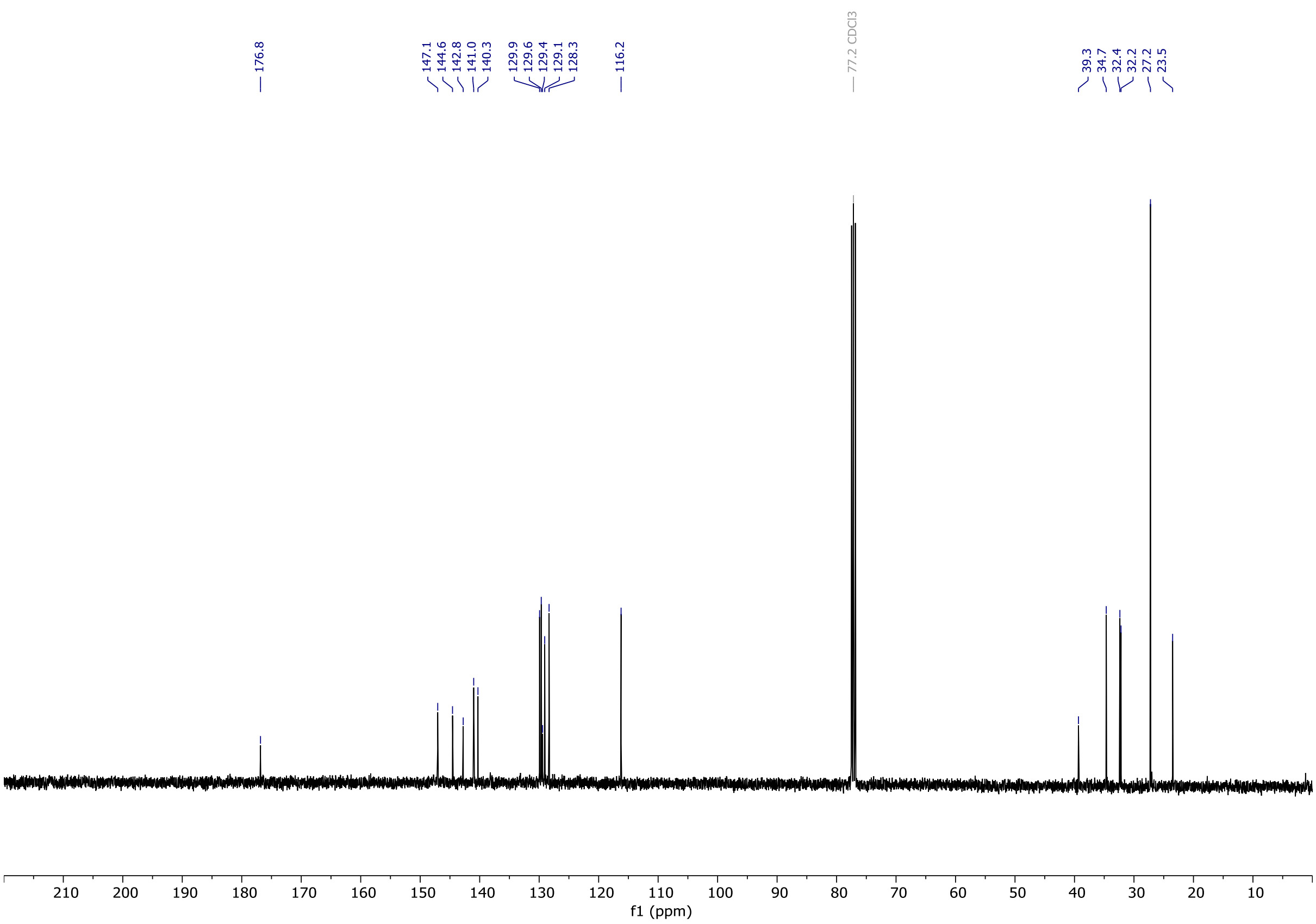
176.8
147.1
144.6
142.8
141.0
140.3
129.9
129.6
129.4
129.1
128.3
116.2
77.2 CDCl3
39.3
34.7
32.4
32.2
27.2
23.5
210 200 190 180 170 160 150 140 130 120 110 100 90 80 70 60 50 40 30 20 10
f1 (ppm)

OX04966b

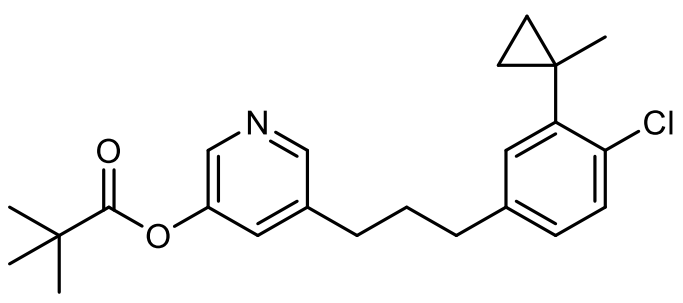

OX04966b

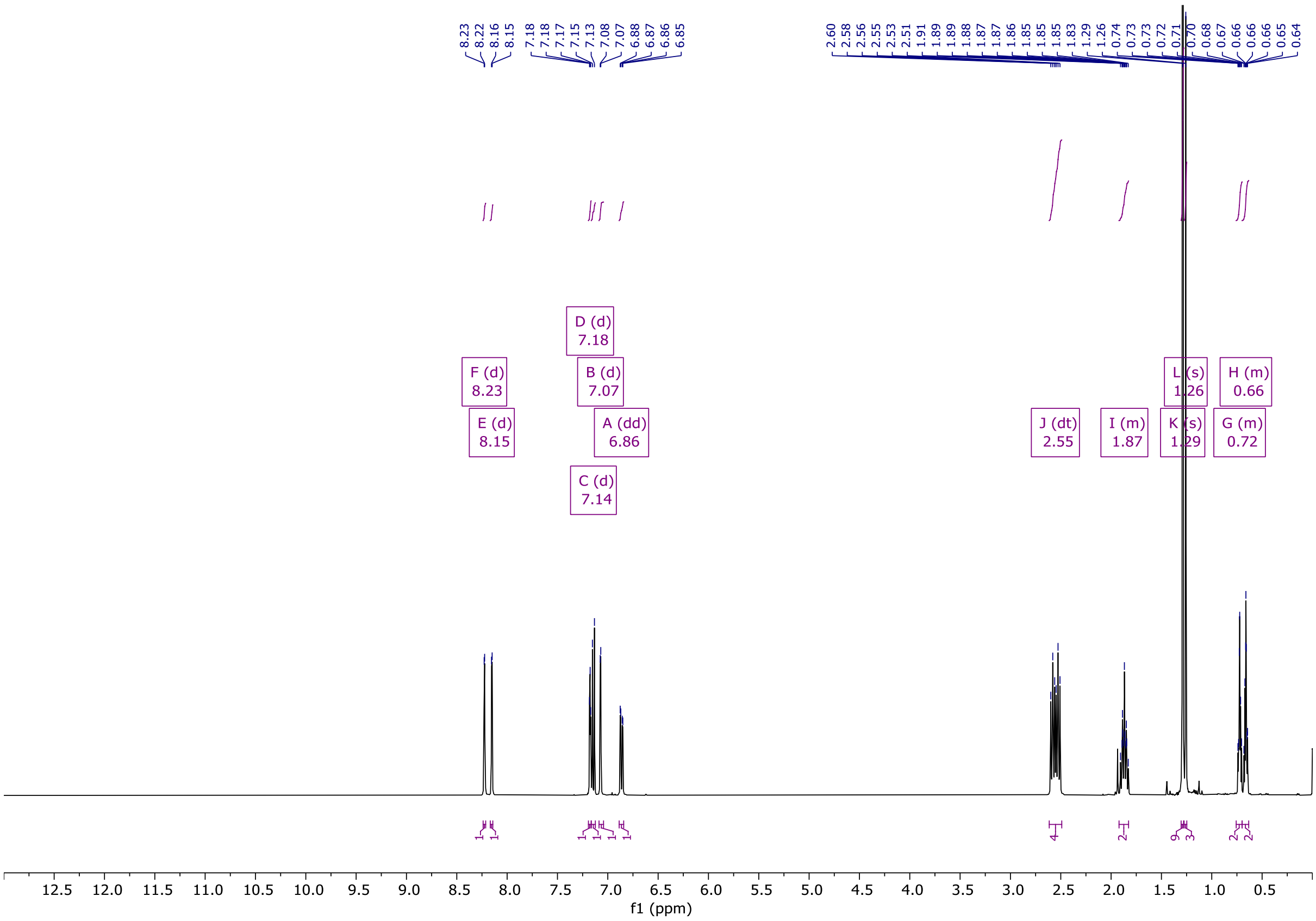

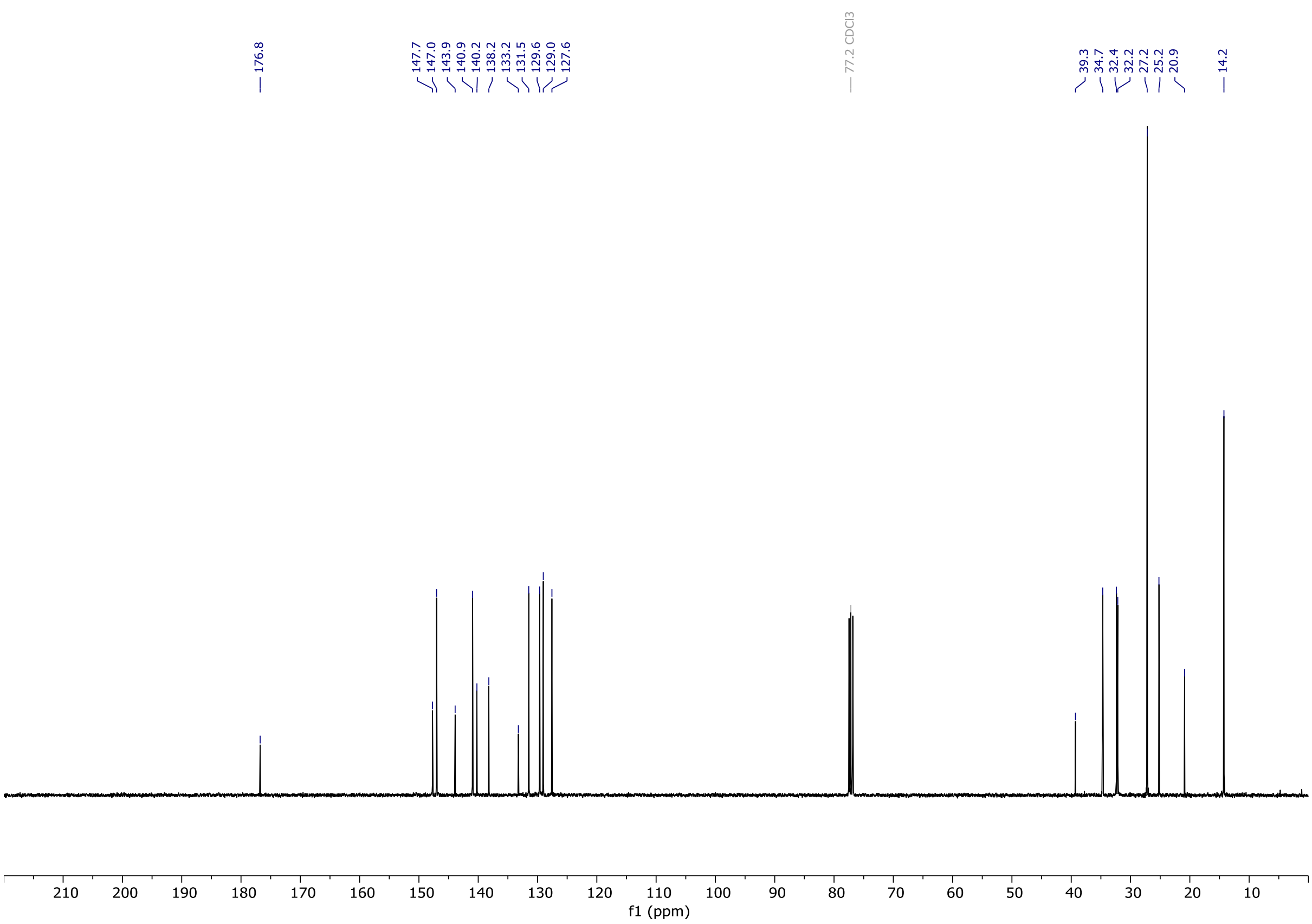

OX04966

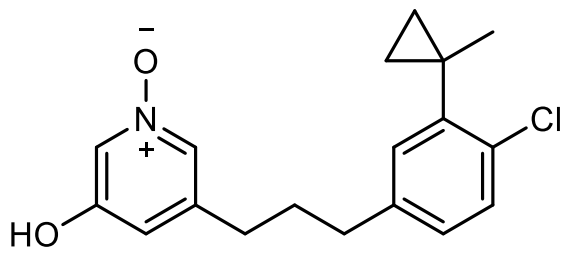

OX04966

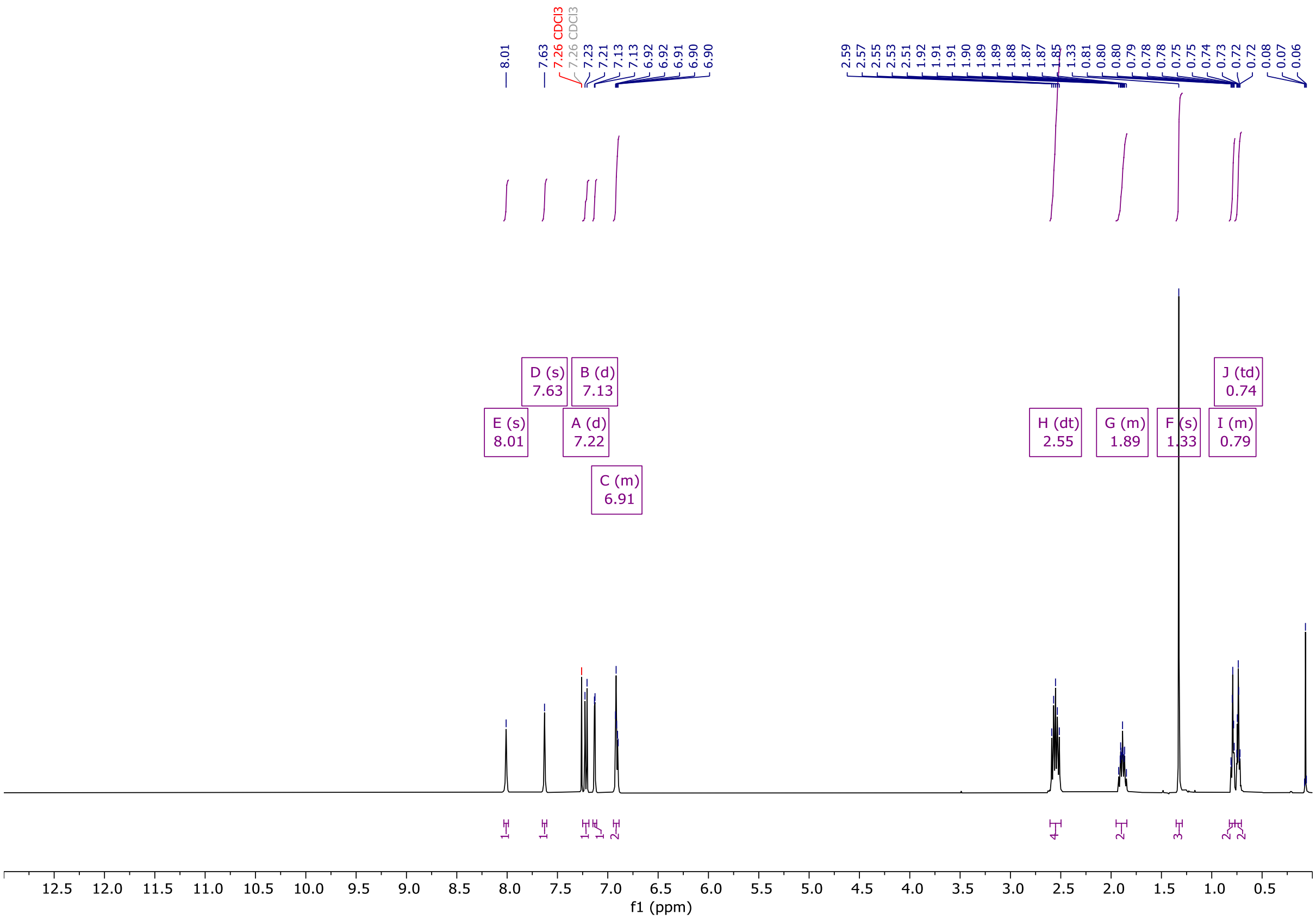

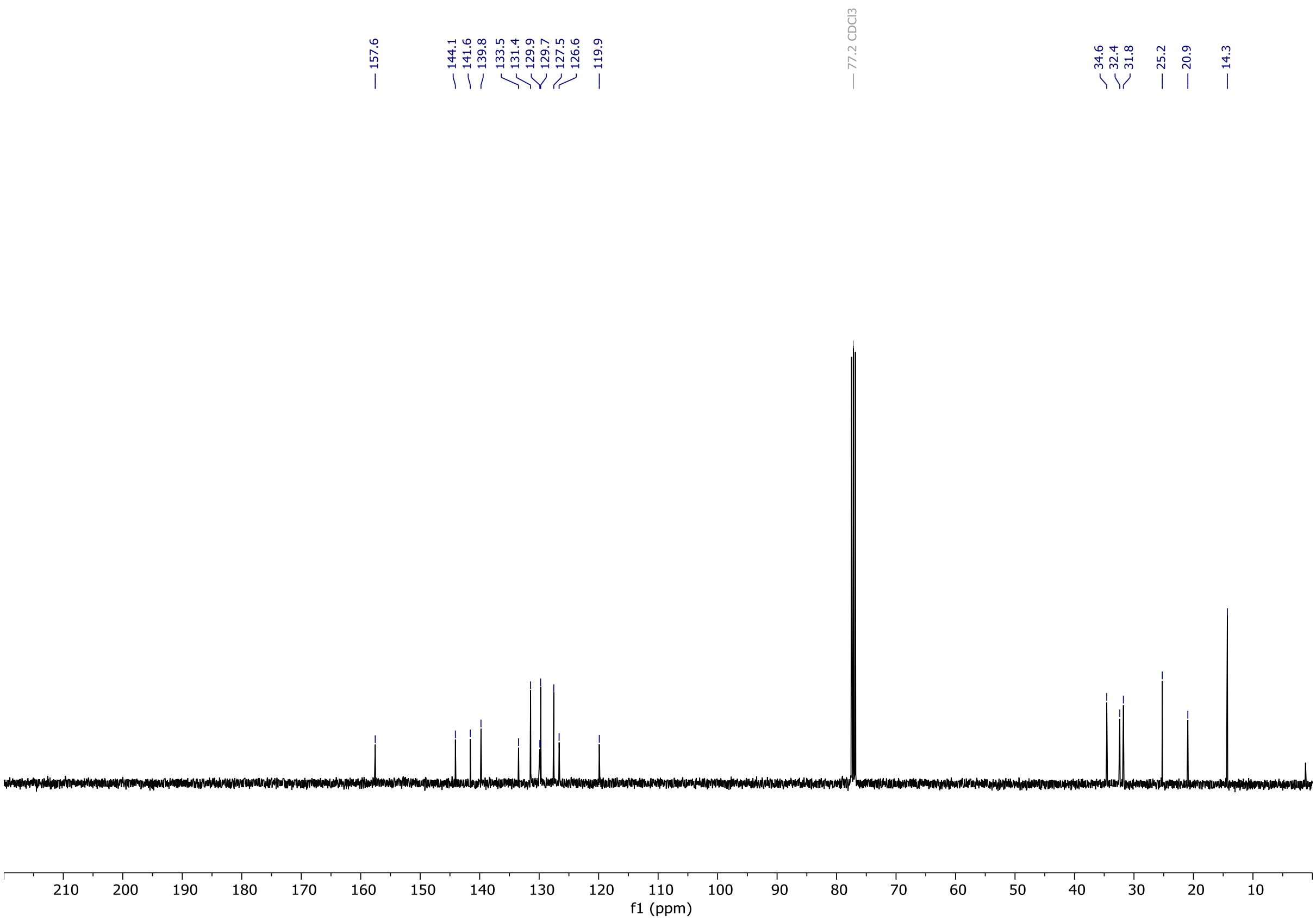

HRMS

OX04539

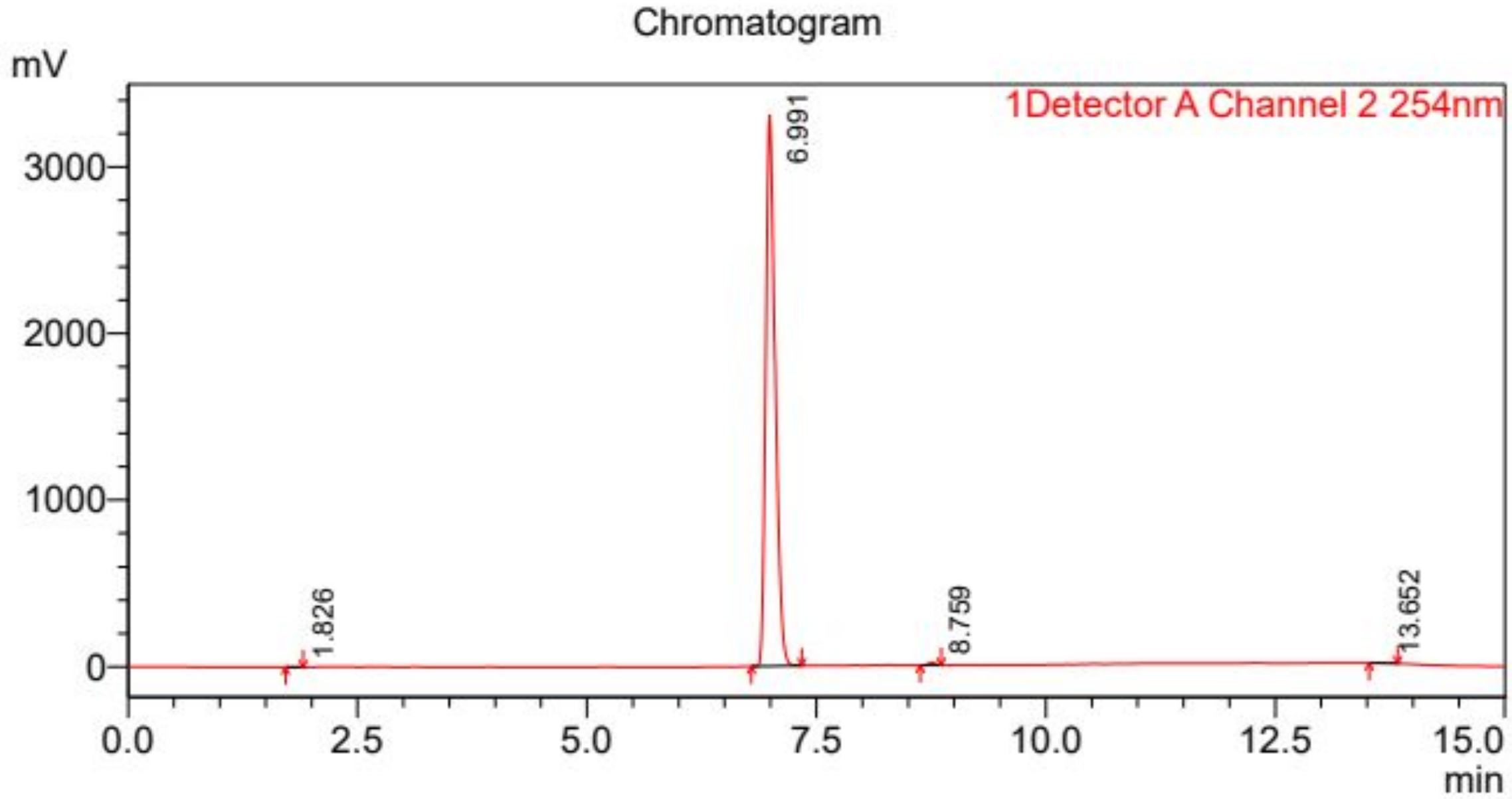

Detector A Channel 2 254nm

Peak#	Ret. Time (min)	Area%
1	1.826	0.096
2	6.991	99.377
3	8.759	0.384
4	13.652	0.143
Total		100.000

OX04540

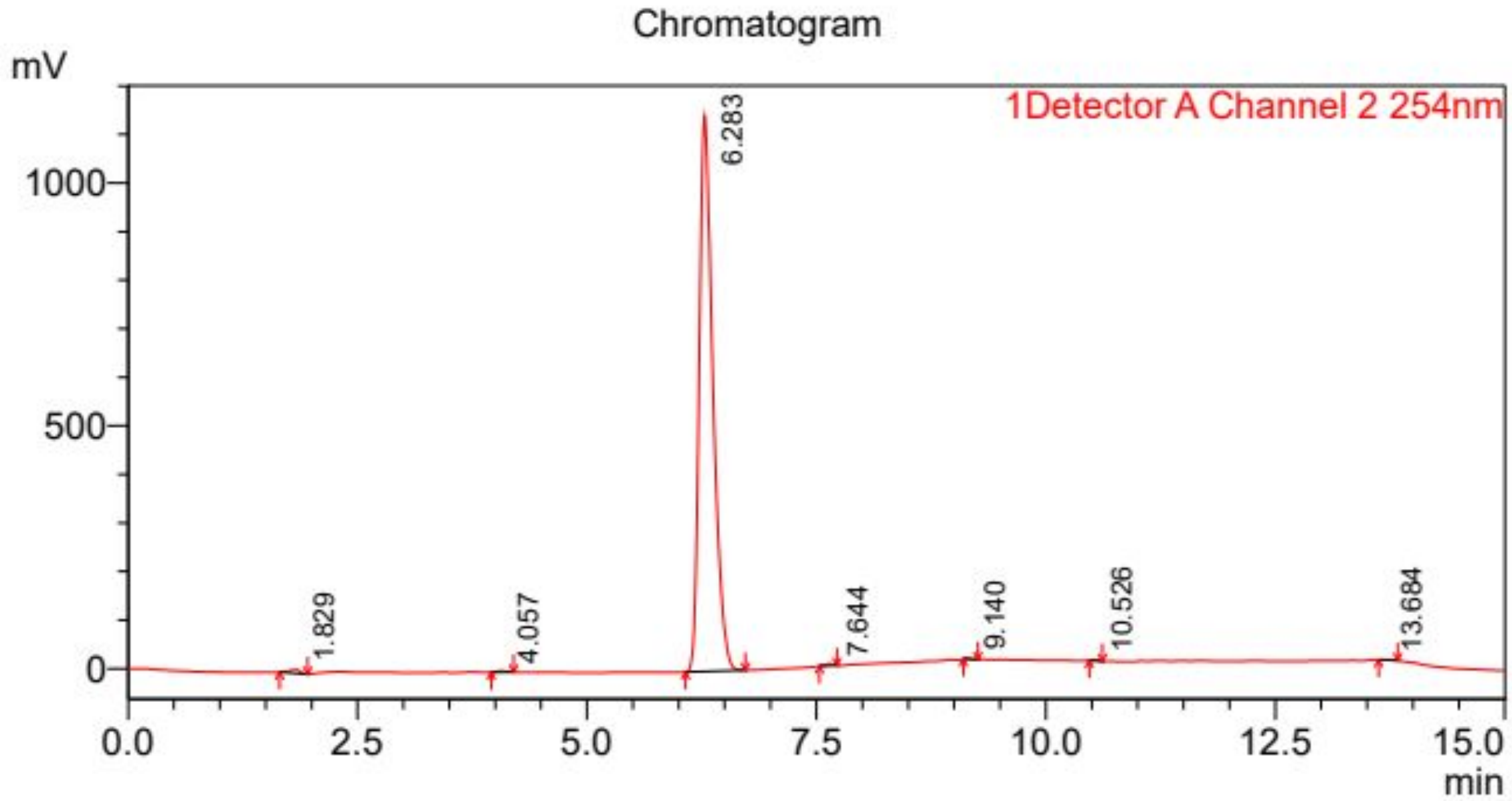

Detector A Channel 2 254nm

Peak#	Ret. Time (min)	Area%
1	1.829	0.506
2	4.057	0.189
3	6.283	98.932
4	7.644	0.178
5	9.140	0.053
6	10.526	0.074
7	13.684	0.067
Total		100.000

OX04954

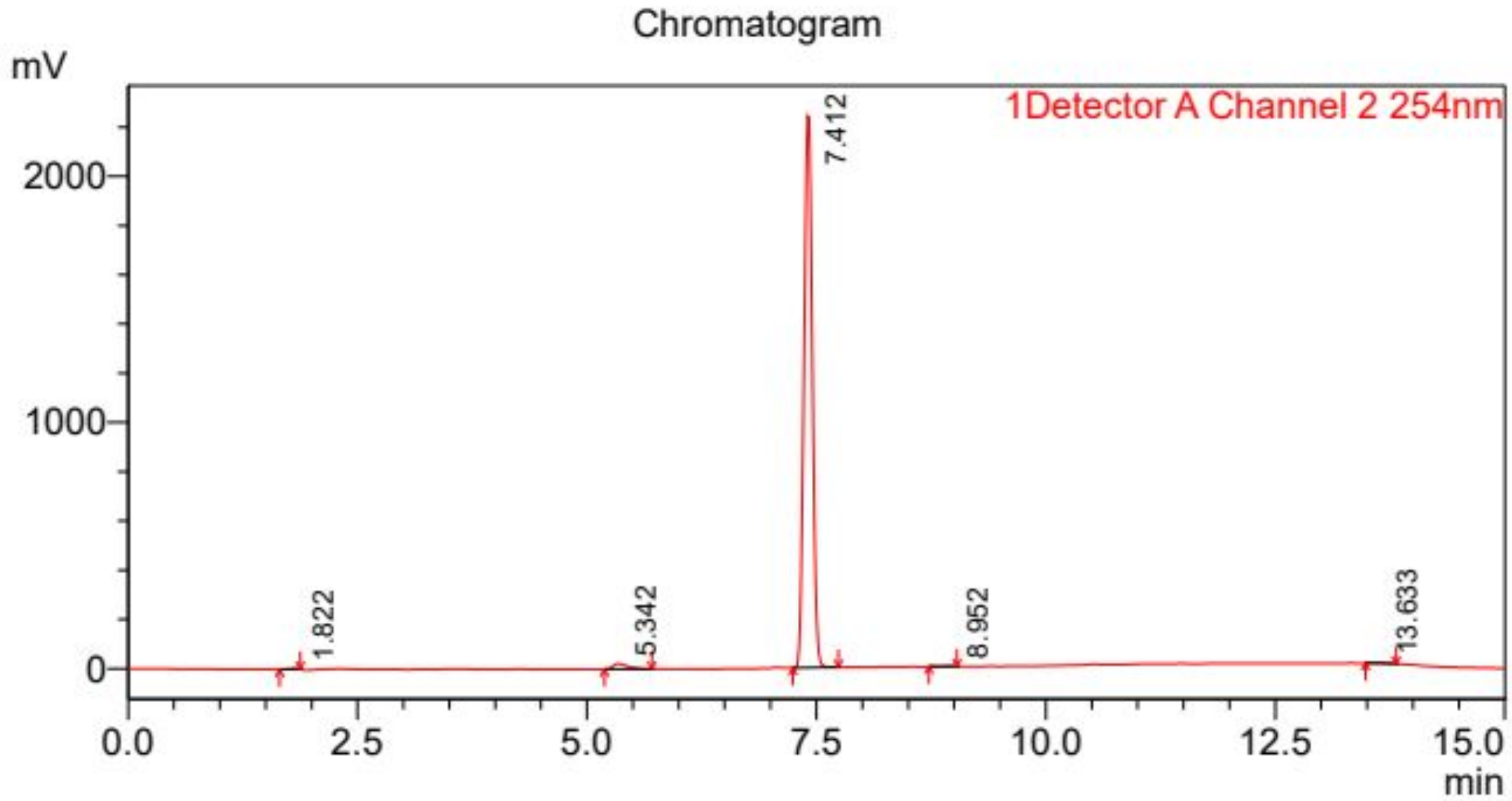

Detector A Channel 2 254nm

Peak#	Ret. Time (min)	Area%
1	1.822	0.185
2	5.342	1.771
3	7.412	97.654
4	8.952	0.150
5	13.633	0.239
Total		100.000

OX04955

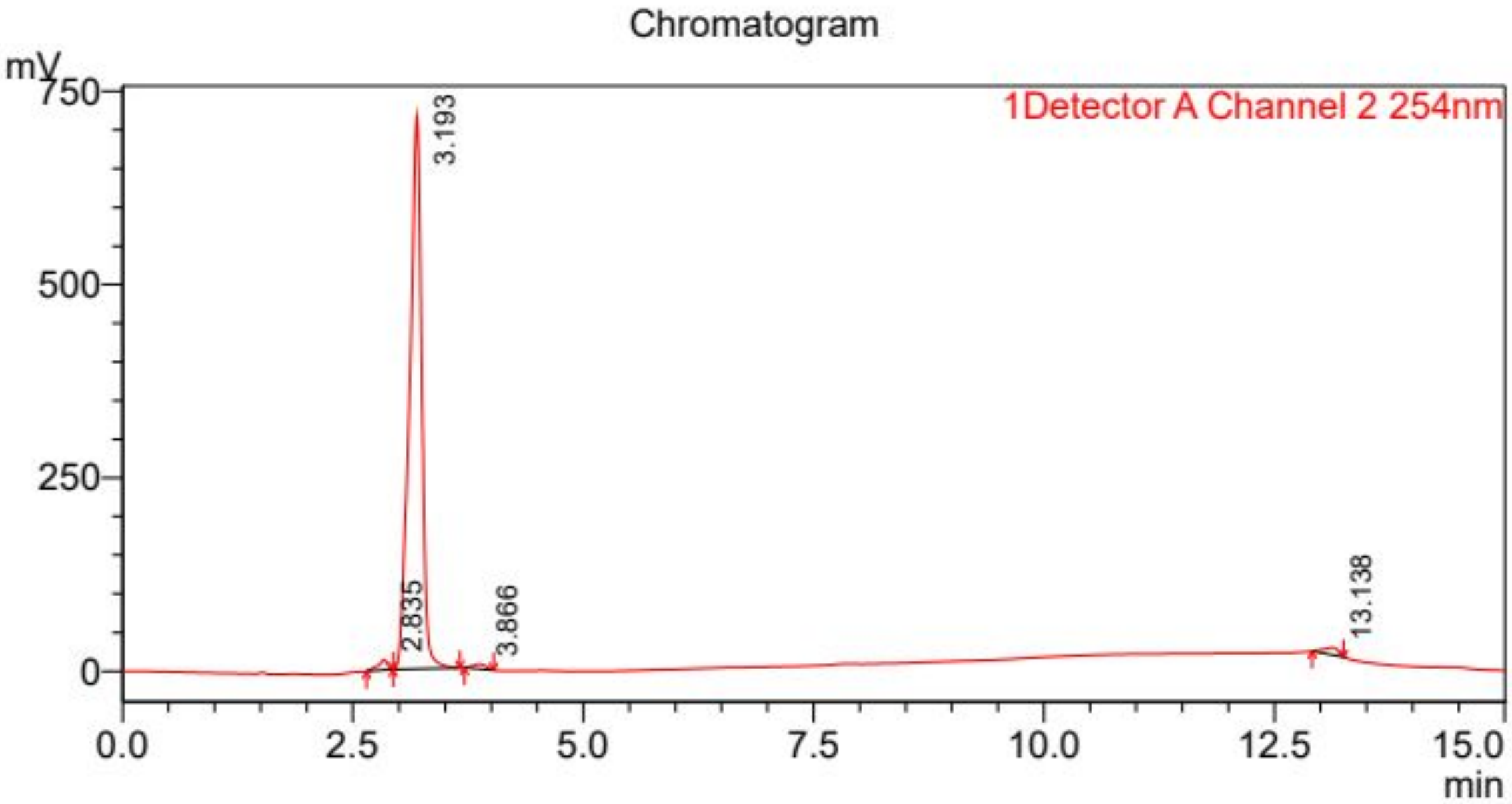

Detector A Channel 2 254nm

Peak#	Ret. Time (min)	Area%
1	2.835	1.399
2	3.193	96.196
3	3.866	0.916
4	13.138	1.489
Total		100.000

OX04956

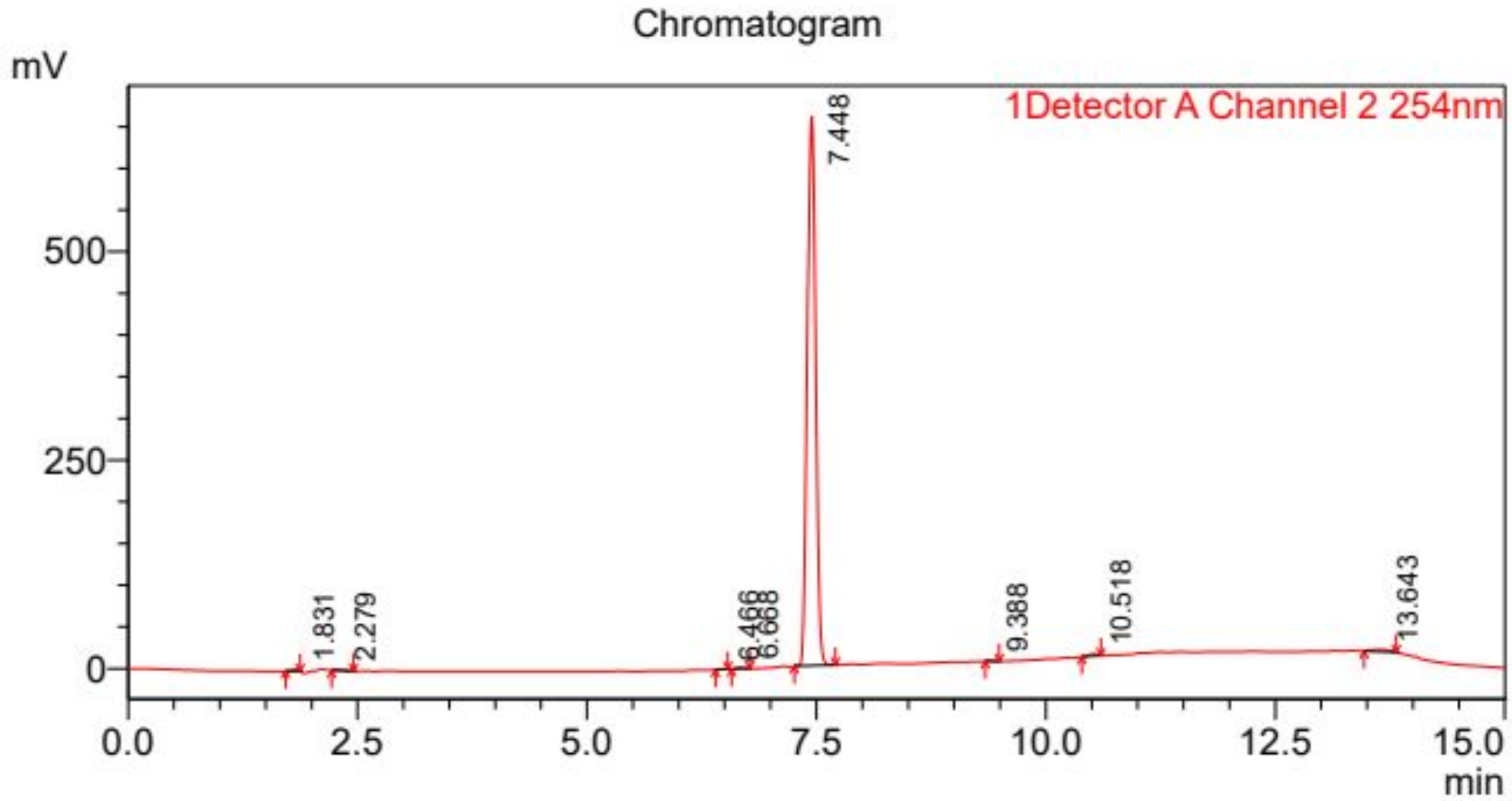

Detector A Channel 2 254nm

Peak#	Ret. Time (min)	Area%
1	1.831	0.230
2	2.279	0.205
3	6.466	0.069
4	6.668	0.234
5	7.448	98.029
6	9.388	0.059
7	10.518	0.368
8	13.643	0.807
Total		100.000

OX04957

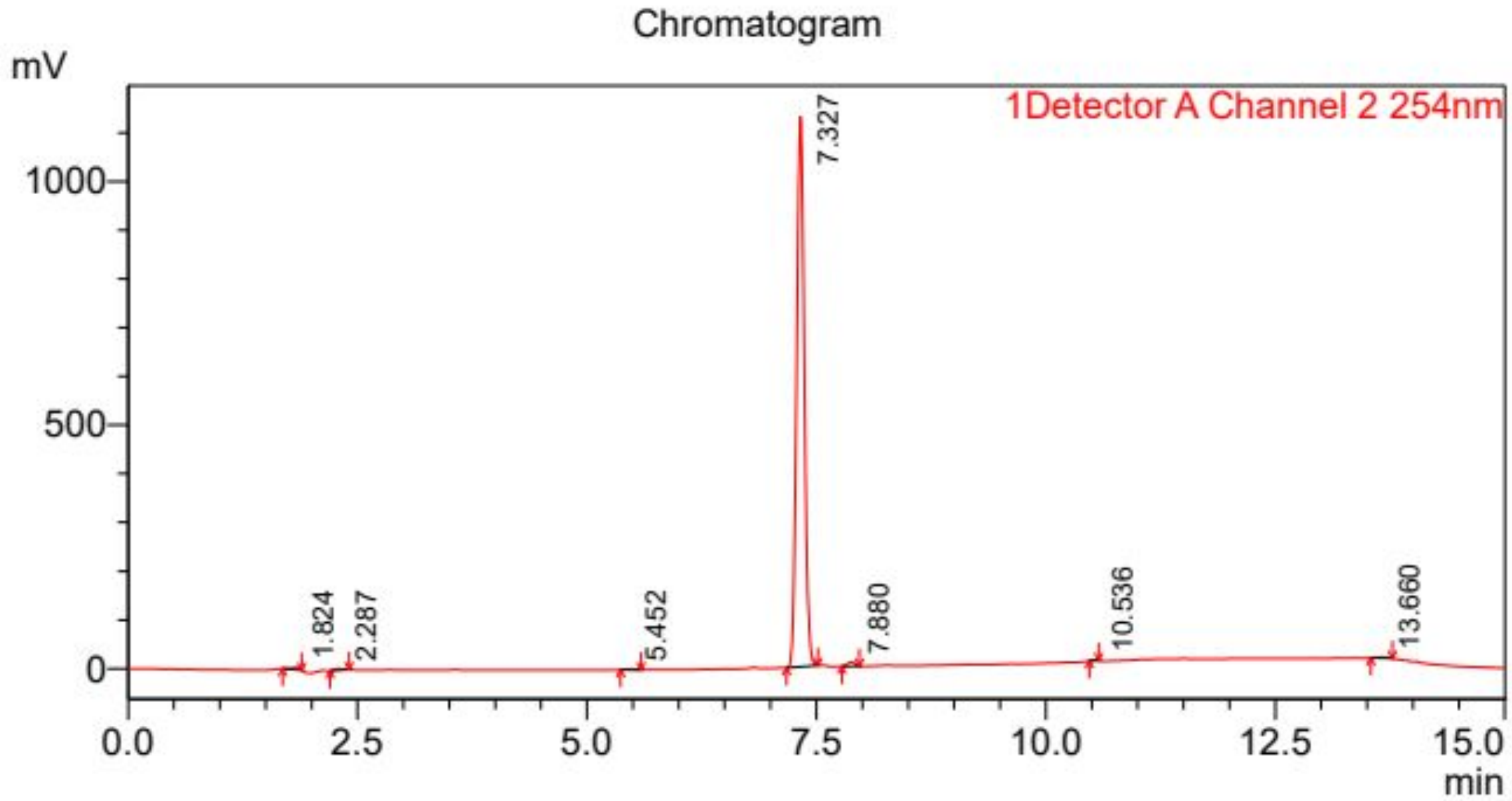

Detector A Channel 2 254nm

Peak#	Ret. Time (min)	Area%
1	1.824	0.410
2	2.287	0.381
3	5.452	0.164
4	7.327	98.053
5	7.880	0.693
6	10.536	0.082
7	13.660	0.217
Total		100.000

OX04958

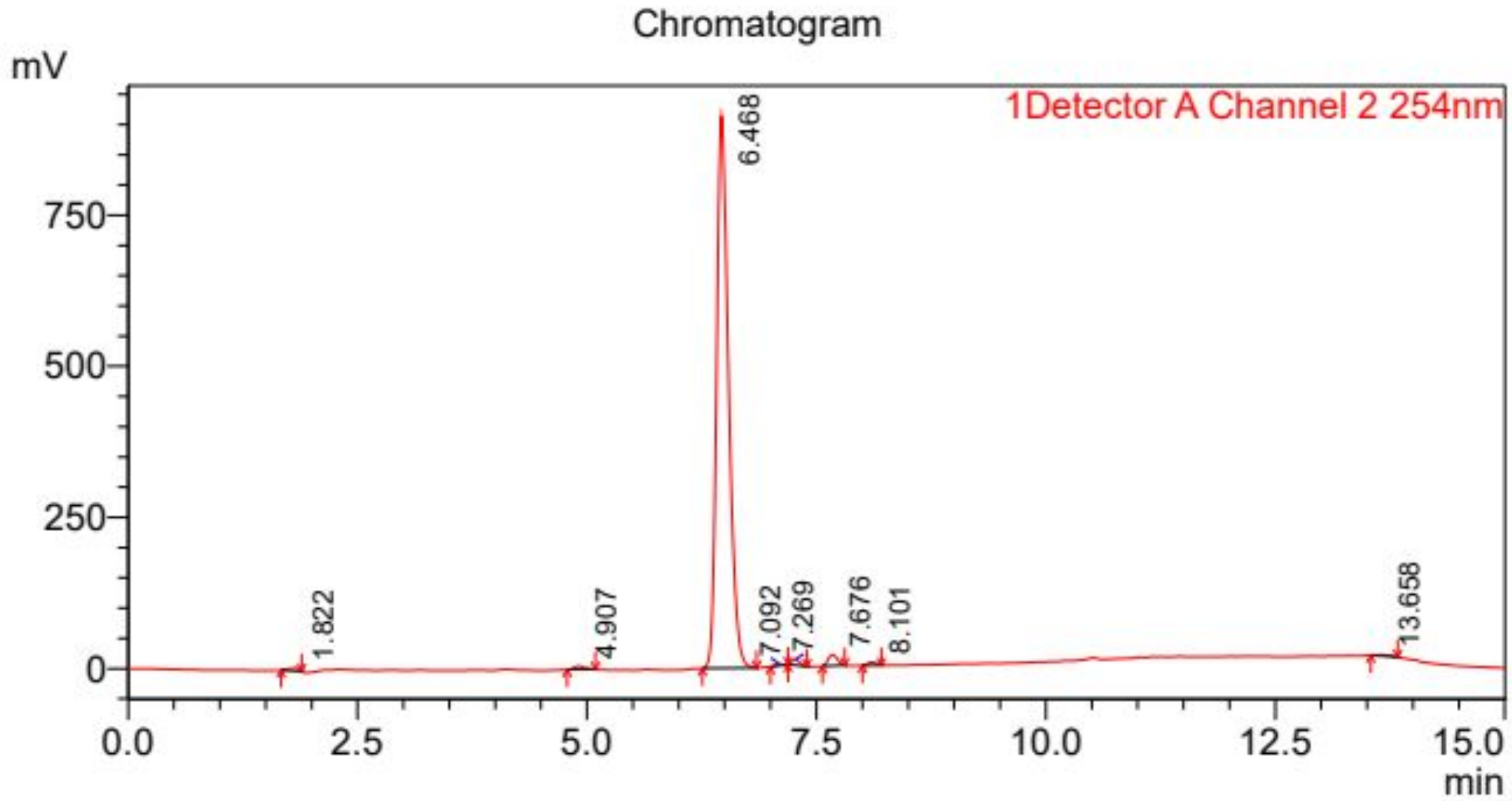

Detector A Channel 2 254nm

Peak#	Ret. Time (min)	Area%
1	1.822	0.444
2	4.907	0.558
3	6.468	96.016
4	7.092	0.192
5	7.269	0.638
6	7.676	1.503
7	8.101	0.419
8	13.658	0.231
Total		100.000

OX04959

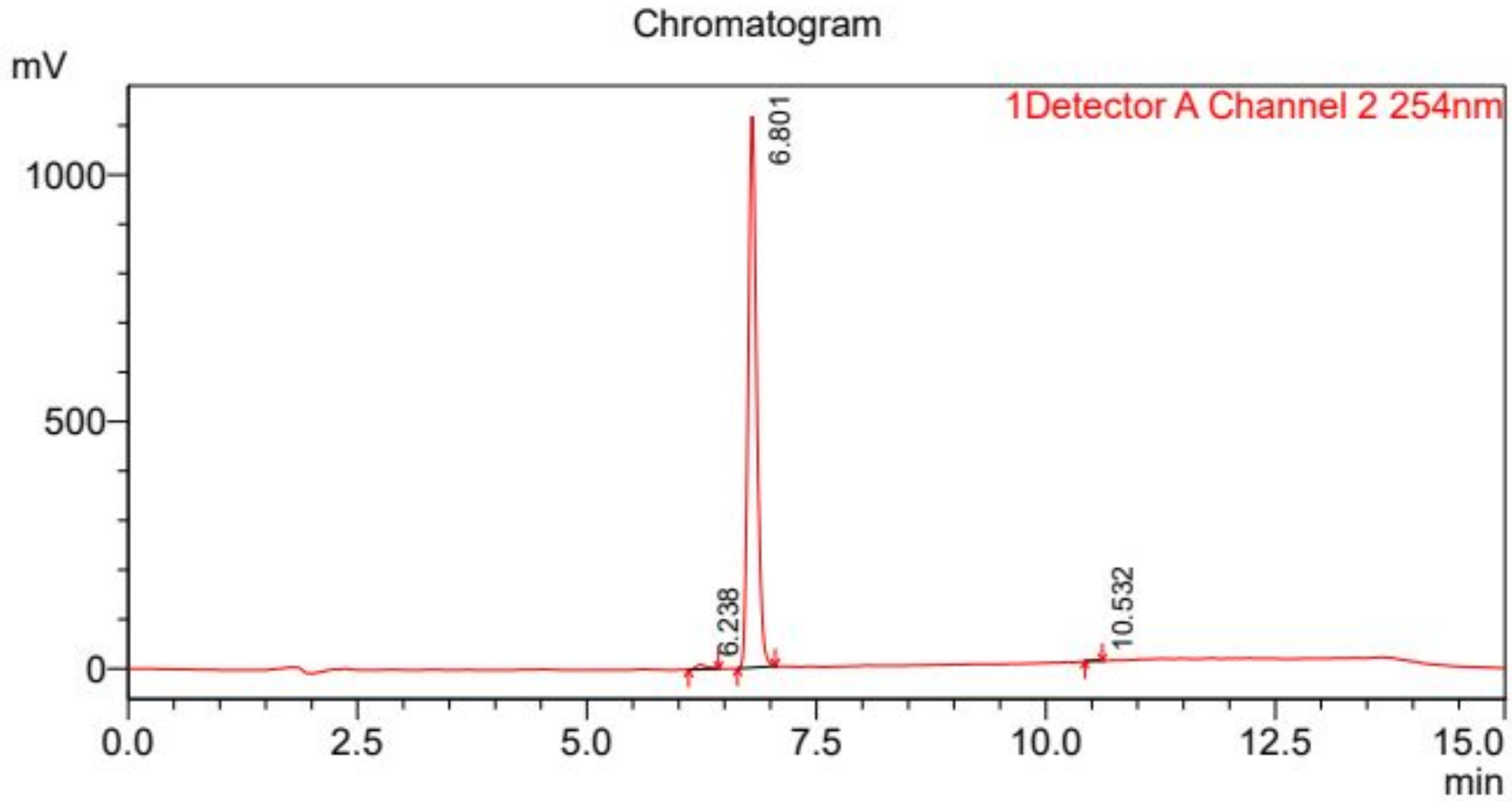

Detector A Channel 2 254nm

Peak#	Ret. Time (min)	Area%
1	6.238	1.055
2	6.801	98.719
3	10.532	0.226
Total		100.000

OX04960

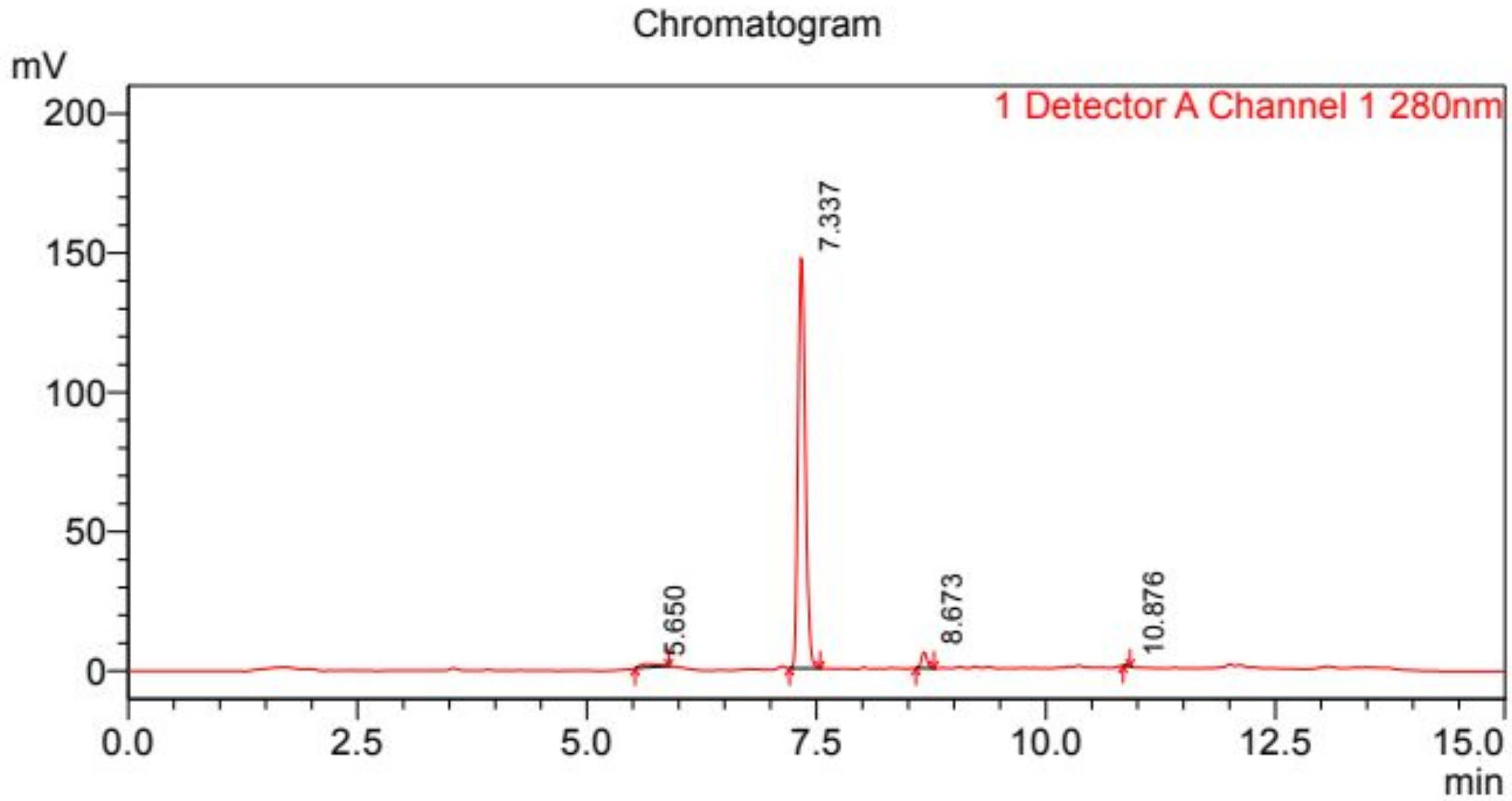

Detector A Channel 1 280nm

Peak#	Ret. Time (min)	Area%
1	5.650	1.857
2	7.337	95.238
3	8.673	2.756
4	10.876	0.148
Total		100.000

OX04961

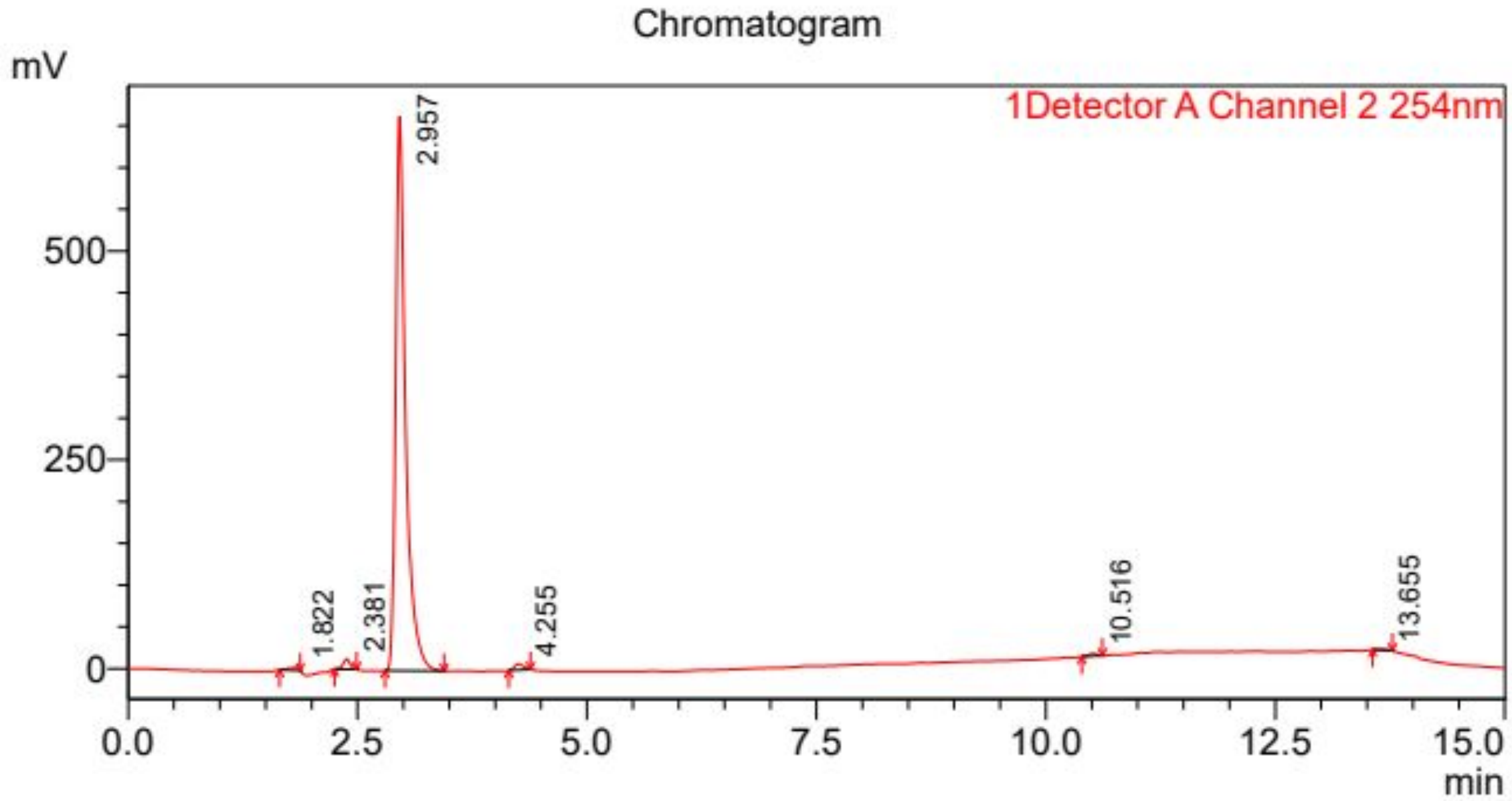

Detector A Channel 2 254nm

Peak#	Ret. Time (min)	Area%
1	1.822	0.467
2	2.381	1.245
3	2.957	96.753
4	4.255	0.961
5	10.516	0.347
6	13.655	0.227
Total		100.000

OX04962

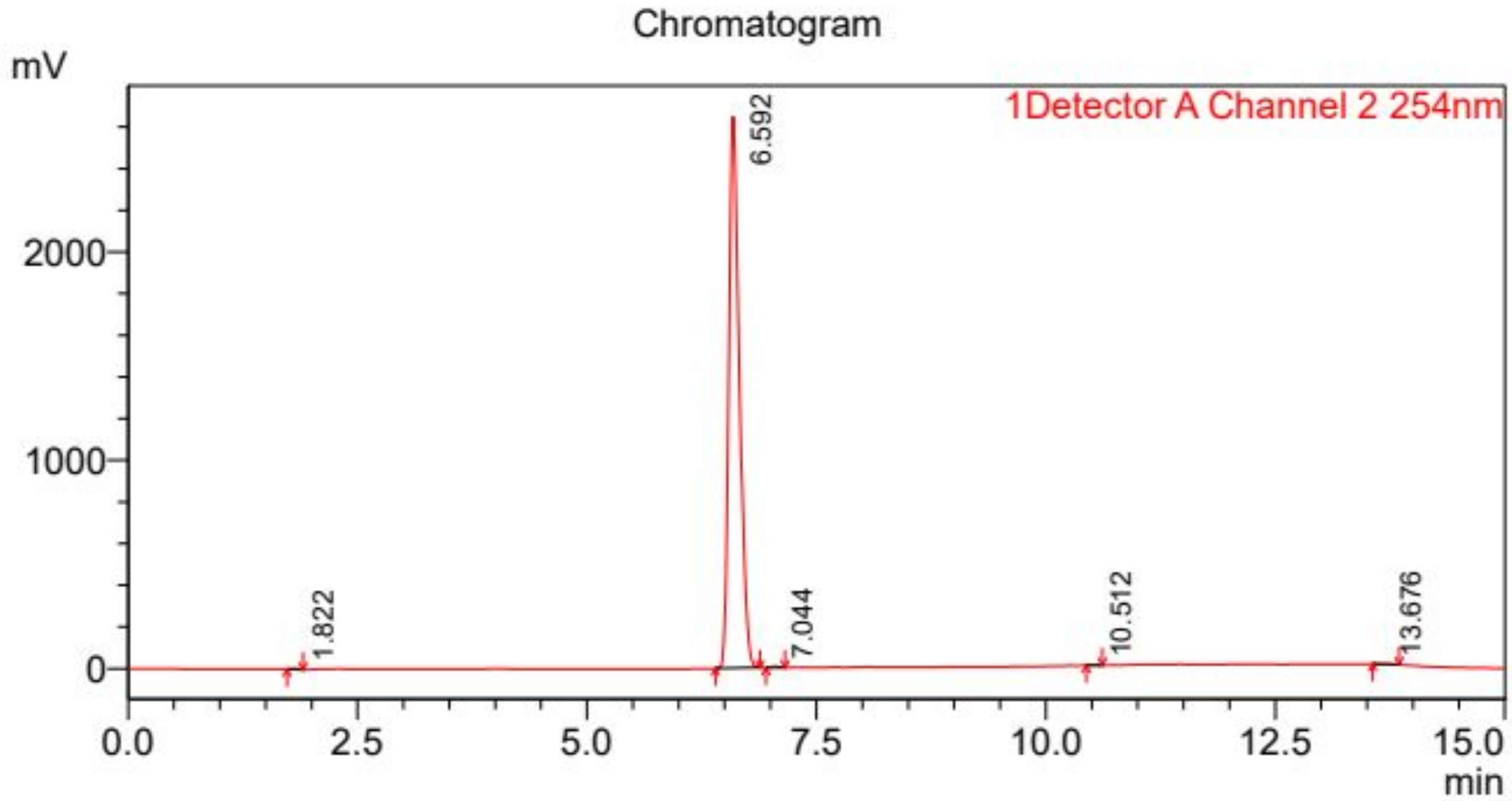

Detector A Channel 2 254nm

Peak#	Ret. Time (min)	Area%
1	1.822	0.112
2	6.592	99.524
3	7.044	0.176
4	10.512	0.056
5	13.676	0.131
Total		100.000

OX04963

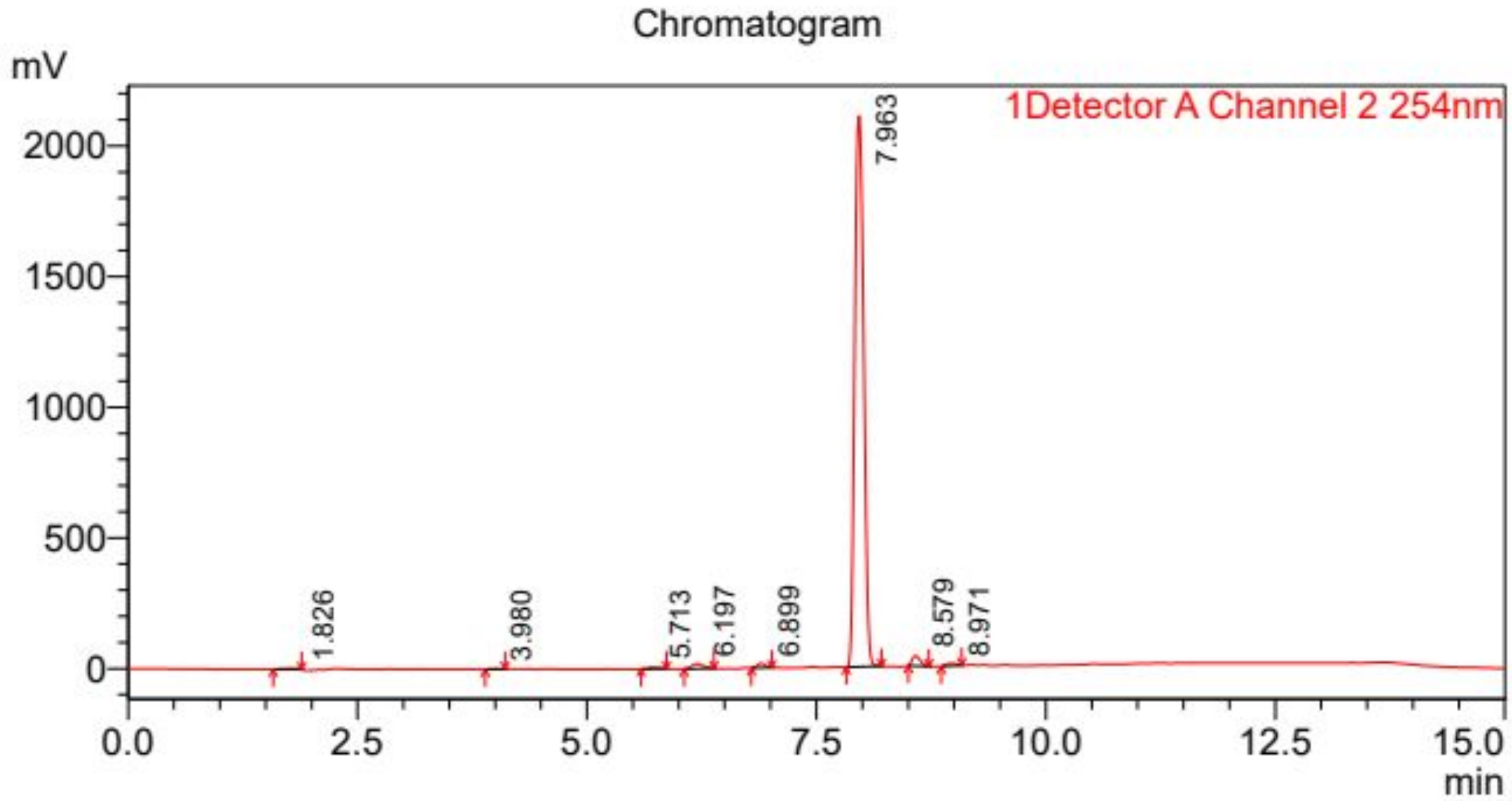

Detector A Channel 2 254nm

Peak#	Ret. Time (min)	Area%
1	1.826	0.364
2	3.980	0.109
3	5.713	0.349
4	6.197	1.096
5	6.899	0.691
6	7.963	95.281
7	8.579	1.595
8	8.971	0.515
Total		100.000

OX04964

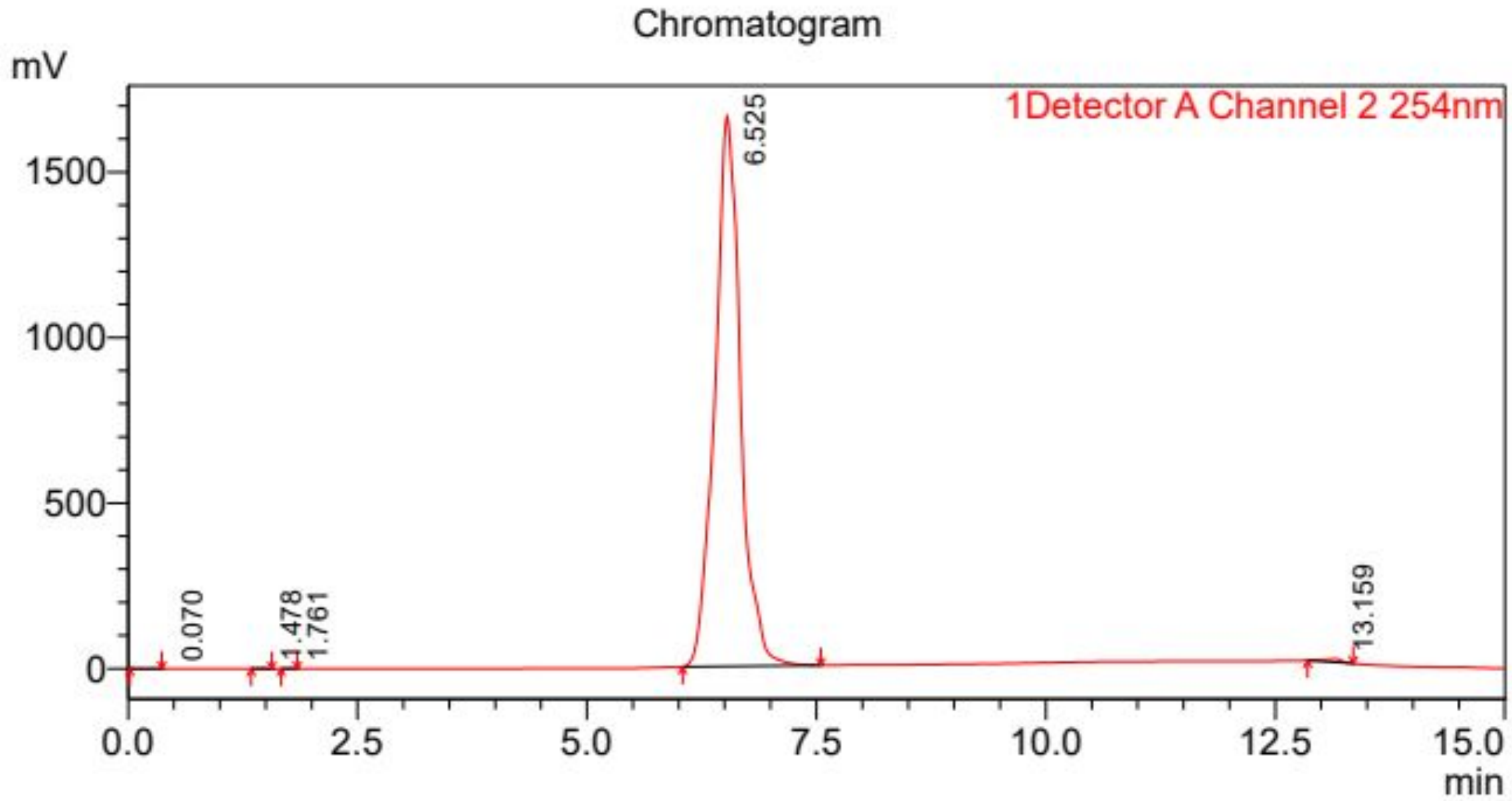

Detector A Channel 2 254nm

Peak#	Ret. Time (min)	Area%
1	0.070	0.005
2	1.478	0.067
3	1.761	0.012
4	6.525	99.482
5	13.159	0.434
Total		100.000

OX04965

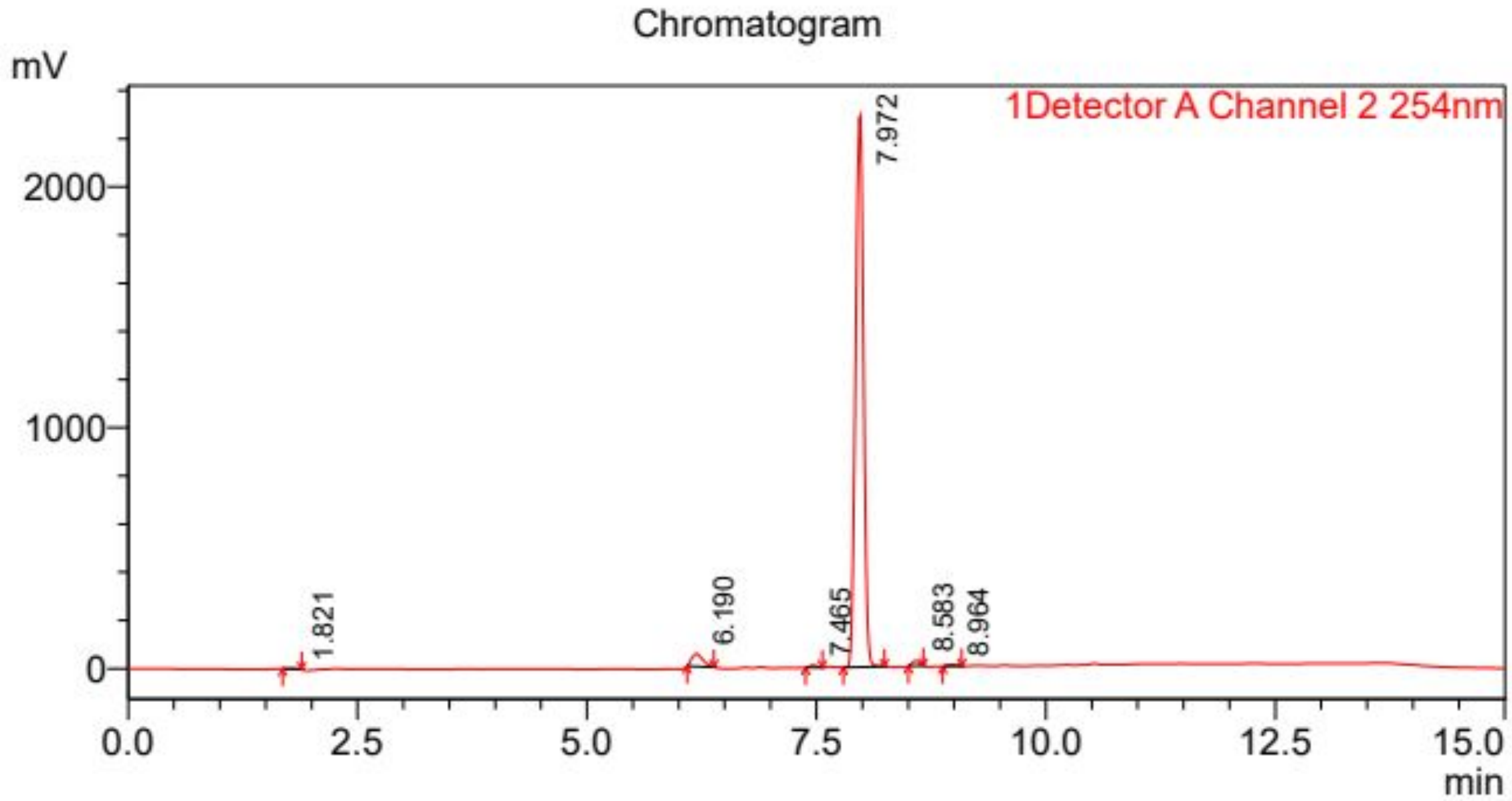

Detector A Channel 2 254nm

Peak#	Ret. Time (min)	Area%
1	1.821	0.237
2	6.190	3.121
3	7.465	0.355
4	7.972	95.148
5	8.583	0.790
6	8.964	0.349
Total		100.000

OX04966

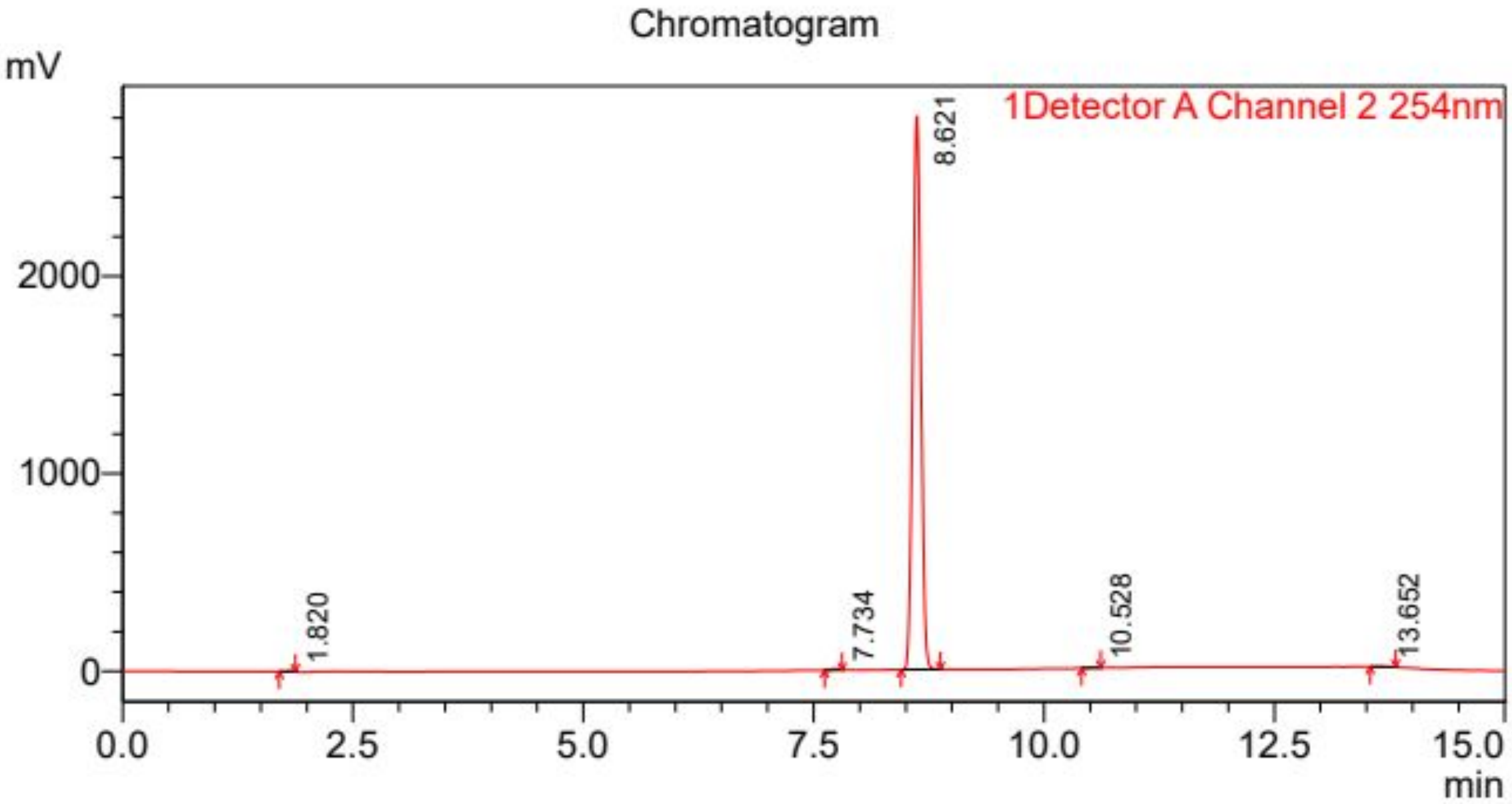

Detector A Channel 2 254nm

Peak#	Ret. Time (min)	Area%
1	1.820	0.118
2	7.734	0.234
3	8.621	99.426
4	10.528	0.094
5	13.652	0.128
Total		100.000

References

1. Makino, K. *et al.* Chemoselective Demethylation of Methoxypyridine. *Synlett* **30**, 951–954 (2019).
