# Supplementary material for: Steric control of signaling bias in the immunometabolic receptor GPR84
Source: Nat Commun. 2026 Sep 5;17:10562. doi: 10.1038/s41467-026-77312-7 (PMC13642346; doi:10.1038/s41467-026-77312-7)
Supplement: Supplementary file 2 [file 41467_2026_77312_MOESM2_ESM.pdf]

nature portfolio

Corresponding author(s): Irina G. Tikhonova, Graeme Milligan, Cheng Zhang, Angela Russell

Last updated by author(s): Nov 9, 2025

Reporting Summary

Nature Portfolio wishes to improve the reproducibility of the work that we publish. This form provides structure for consistency and transparency in reporting. For further information on Nature Portfolio policies, see our Editorial Policies and the Editorial Policy Checklist.

Statistics

For all statistical analyses, confirm that the following items are present in the figure legend, table legend, main text, or Methods section.

n/a	Confirmed	
☐	☒	The exact sample size (n) for each experimental group/condition, given as a discrete number and unit of measurement
☐	☒	A statement on whether measurements were taken from distinct samples or whether the same sample was measured repeatedly
☐	☒	The statistical test(s) used AND whether they are one- or two-sided<br>*Only common tests should be described solely by name; describe more complex techniques in the Methods section.*
☒	☐	A description of all covariates tested
☒	☐	A description of any assumptions or corrections, such as tests of normality and adjustment for multiple comparisons
☐	☒	A full description of the statistical parameters including central tendency (e.g. means) or other basic estimates (e.g. regression coefficient) AND variation (e.g. standard deviation) or associated estimates of uncertainty (e.g. confidence intervals)
☐	☒	For null hypothesis testing, the test statistic (e.g. F, t, r) with confidence intervals, effect sizes, degrees of freedom and P value noted<br>*Give P values as exact values whenever suitable.*
☒	☐	For Bayesian analysis, information on the choice of priors and Markov chain Monte Carlo settings
☒	☐	For hierarchical and complex designs, identification of the appropriate level for tests and full reporting of outcomes
☒	☐	Estimates of effect sizes (e.g. Cohen's d, Pearson's r), indicating how they were calculated

Our web collection on statistics for biologists contains articles on many of the points above.

Software and code

Policy information about availability of computer code

Data collection	SerialEM 4.0, EPU3.7 , AMBER20, Schrodinger Maestro 2021-3, MOE 2019.0102, GROMACS 2021.4, AlphaFold2
Data analysis	cryoSPARC 4.2, COOT 0.9.8.7, Phenix 1.21.2, ChimeraX-1.9, Prism 10, Schrodinger GLIDE, Schrodinger Maestro 2021-3 suite, CHARMM-GUI, AmberTools23, MDAnalysis 2.7.0, MDAnalysis 2.6.1, SciKit Learn, 1.5.1 VMD1.9.3 , Pymol 3.0.5, NumPy, 1.26.4 SciPy1.14.0, MatPlotLib 3.9.1, Seaborn 0.13.2, Python 3.12.4, ParmEd 3.4.4, MestReNova 15.0.0-34764, MOE 2019.0102, Pymol 3.1.3, DataWarrior 5.5.0, VMD 1.9.4, R 4.5.0, Jupyter-lab 4.0.11 (notebook 7.0.8)

For manuscripts utilizing custom algorithms or software that are central to the research but not yet described in published literature, software must be made available to editors and reviewers. We strongly encourage code deposition in a community repository (e.g. GitHub). See the Nature Portfolio guidelines for submitting code & software for further information.

Data

Policy information about availability of data

All manuscripts must include a data availability statement. This statement should provide the following information, where applicable:

- Accession codes, unique identifiers, or web links for publicly available datasets
- A description of any restrictions on data availability
- For clinical datasets or third party data, please ensure that the statement adheres to our policy

The 3D cryo-EM density maps of the structures of GPR84 signaling complexes have been deposited in the Electron Microscopy Data Bank under the accession

numbers EMD-71830 (https://www.ebi.ac.uk/emdb). PDB -9PT4

Simulation protocols, input and output files were uploaded to ZENODO and linked to the current publication.

Research involving human participants, their data, or biological material

Policy information about studies with human participants or human data. See also policy information about sex, gender (identity/presentation), and sexual orientation and race, ethnicity and racism.

Reporting on sex and gender	N/A
Reporting on race, ethnicity, or other socially relevant groupings	N/A
Population characteristics	N/A
Recruitment	N/A
Ethics oversight	N/A

Note that full information on the approval of the study protocol must also be provided in the manuscript.

Field-specific reporting

Please select the one below that is the best fit for your research. If you are not sure, read the appropriate sections before making your selection.

☒ Life sciences ☐ Behavioural & social sciences ☐ Ecological, evolutionary & environmental sciences

For a reference copy of the document with all sections, see nature.com/documents/nr-reporting-summary-flat.pdf

Life sciences study design

All studies must disclose on these points even when the disclosure is negative.

Sample size	For functional assays, we used data from 3-5 experiments. The rational is that three biological replicates are the minimum for inferential analysis. For cryo-EM studies, our dataset contains 10 to 20 million particles, collected from >10,000 micrographs. The sample size was determined based on the number of particles to achieve sufficient resolution of cryo-EM maps. For molecular dynamics simulations, we used 5-10 replicas of 1-2 microseconds (10000-20000 frames per replica).
Data exclusions	For cryo-EM data, particles were excluded during 2D and 3D classification based on protocols of cryoSparc to achieve high resolution.
Replication	For all signaling and binding assays, we used data from 3-5 repeated experiments . Not all attempts at replication were successful because of mistakes in sample preparation. For each cryo-EM structure, 4-6 cryo-grids prepared from the same protein sample were screened and the best grid was used from data collection. Only one cryo-EM dataset was used for the determination of each structure. Classical MD simulations included 5-10 replicates.
Randomization	All experiments follow a deterministic pattern. No probability distributions were involved in the rationale of experimental design. All the pharmacological data for replication and statistical analysis were obtained using distinct biological replicates from membrane preparations from different cell cultures. Molecular Dynamics simulations in AMBER employed randomly generated starting velocities (generated once per system) and a constantly acting random force, in both cases SEED was generated from CPU time in microseconds and the seeds were never repeated between replicates of the same simulation.
Blinding	No human or animal subjects are involved. Blinding is not applicable to the study. All experiments follow a deterministic pattern. The investigators were aware of how cells were treated before collecting data and how protein samples were prepared for cryo-EM data collection. Blinding is not applicable to the conducted molecular simulation study.

Reporting for specific materials, systems and methods

We require information from authors about some types of materials, experimental systems and methods used in many studies. Here, indicate whether each material, system or method listed is relevant to your study. If you are not sure if a list item applies to your research, read the appropriate section before selecting a response.

Materials & experimental systems

n/a	Involved in the study
☐	☒ Antibodies
☐	☒ Eukaryotic cell lines
☒	☐ Palaeontology and archaeology
☒	☐ Animals and other organisms
☒	☐ Clinical data
☒	☐ Dual use research of concern
☒	☐ Plants

Methods

n/a	Involved in the study
☒	☐ ChIP-seq
☒	☐ Flow cytometry
☒	☐ MRI-based neuroimaging

Antibodies

Antibodies used	The rabbit phospho-site-specific GPR84 antiserum pThr263/pThr264-GPR84 (7TM0120B) and the non-phospho state GPR84 antiserum (7TM0120N) were developed in collaboration with 7TMAntibodies GmbH. Both were used at 1:2000 dilution. IRDye 800CW donkey anti-rabbit IgG was from LI-COR Biosciences and used at 1:10,000 dilution. The sheep GFP antiserum was generated in-house
Validation	The scFV16 was characterised https://doi.org/10.1038/s41467-018-06002-w, anti-pThr263/pThr264 antiserum was validated https://doi.org/10.1016/j.jbc.2022.101932.

Eukaryotic cell lines

Policy information about cell lines and Sex and Gender in Research

Cell line source(s)	Flp-In™ T-REx™ 293 cells (InvitroGen), HEK293T cells (ATCC), insect Sf9 cells (ExpressionSystems), CHO-K1-hGPR84 (DiscoverX 95-0158C2), CHO-K1-hGPR84-β-arrestin-2 (DiscoverX 93-0647C2).
Authentication	All the mammalian cell lines are fully authenticated. We rely on the vendor to authenticate the insect cell line.
Mycoplasma contamination	HEK293T and Flp-In T-REx 293 cells were authenticated by Northgene (Case Number C-24809a and C-24809b). All cell lines were confirmed to be mycoplasma free and continue to be tested for mycoplasma every 3 months.
Commonly misidentified lines (See ICLAC register)	None found in the ICLAC database.

Plants

Seed stocks	N/A
Novel plant genotypes	N/A
Authentication	N/A
