# Supplementary material for: Steric control of signaling bias in the immunometabolic receptor GPR84
Source: Nat Commun. 2026 Sep 5;17:10562. doi: 10.1038/s41467-026-77312-7 (PMC13642346; doi:10.1038/s41467-026-77312-7)
Supplement: Supplementary file 3 [file 41467_2026_77312_MOESM3_ESM.pdf]

nature portfolio

# Peer Review File

# Steric control of signaling bias in the immunometabolic receptor GPR84

Corresponding Author: Professor Angela Russell

**This file contains all reviewer reports in order by version, followed by all author rebuttals in order by version.**

Version 0:

Reviewer comments:

Reviewer #1

(Remarks to the Author)
The manuscript by Wang et al. reports an interdisciplinary research project that combines cryoEM, MD simulations and rational synthesis of a novel compound series. The major aim of this study is to mechanistically understand ligand-dependent bias at GPR84 and therefore the abovementioned methodological approaches are combined in a reasonable manner. The authors describe new biased ligands (e.g. OX04529 and OX04954) and provide a rational explanation for the ligand's characteristics by means of MD simulations and mutational studies to support their findings. While I cannot fully judge on the cryoEM part, the MD part, the synthesis, and the pharmacological evaluation is methodologically sound and well-reported.

Given the interesting and relevant topic, the mechanistic insights, the valuable novel compound series and the combined methodology used, I support publication of this study in Nature Communications. However, a few issues have to be addressed before acceptance, as outlined below:

1) One of the most surprising results is the huge difference in ß-arr-2 recruitment between OX04529 and OX04954. Those compounds are highly similar and only differ in one substituent (-CF3 vs. -CH3). While the authors give some mechanistic insights on how these compounds interact within the binding site, the large functional difference of those two compounds is not well rationalized and could be analyzed in more detail.

2) There is a discrepancy in the data for OX04529 shown in Fig 1d and Fig 3f.

3) The molecular library of OX04529 derivatives is well-thought and rational. The discussion of the SAR, however, is not very detailed and misses a structural explanation (or hypothesis) of the reported effects. For example, the halogen series gave a surprising result, where a methyl, or fluorine substituent gave similar results in ß-arr-2 recruitment, which was significantly different compared to bromine or chlorine substituents. One would expect a rank order according the size of the halogen atom, but this is not the case.

4) The role of L336 is described as essential for the biased properties of reported compounds. This could be easily extended to further support the model. E.g. a L336V mutation could be interesting to look for a graded effect on bias (L336, L336V, L336A). Another possibility would be to test ligands with larger substituents on the L336A mutant (e.g. OX04964). These are some suggestions to strengthen the study, but I don't want to push for a lot of new experiments.

5) Please provide bias factors for the reported compounds.

6) The authors state that their findings on the bias mechanism for GPR84, is transferable to other GPCRs. This should be elaborated a bit more in the discussion. The observation that ligand-dependent bias could be achieved by 'extended binding modes', or more bulky substituents compared to a lead structure has been reported previously for different receptors, e.g. 10.1021/acschembio.7b00275, 10.1038/nchembio.2527, 10.1038/s41589-018-0145-x, 10.1021/acsptsci.0c00069, 10.1111/febs.15783, 10.1073/pnas.2112397119 among others).

7) In the methods section, there are two paragraphs about docking (GLIDE and MOE). It remains unclear which docking program was used for which part of the study.

Reviewer #2

(Remarks to the Author)
This manuscript investigates the mechanism of biased agonism in GPR84 using the cryo-EM structure of the GPR84–Gi complex, complemented by mutagenesis, MD simulations, and analysis of ligand analogs. GPR84 is an immunometabolic GPCR with potential relevance in inflammation and cancer immunotherapy. The topic is important, and the work provides valuable mechanistic insight. However, several points require clarification or further analysis to substantiate the conclusions.
• The cryo-EM map appears to be of high quality and shows lipid-like densities. However, only a single cholesterol molecule was modeled despite the evident density between TM4 and TM5 on the intracellular side. Lipids can modulate GPCR dynamics, and the unmodeled density may influence the ICL3 region or G-protein coupling. The authors should either model this additional lipid or at least discuss its potential structural and functional role.
• The structure was solved with a G-protein–biased ligand and unsurprisingly resembles previously reported active-state complexes. However, GPCRs are dynamic, and biased ligands often stabilize specific conformations not captured in static structures. This has been well established by NMR studies of adrenergic receptors. Although the authors used MD simulations, additional conformational states might already be present in the cryo-EM data. Could authors examine the alternative class seen in their dataset during the processing (Extended Data Figure 4)? It can also be beneficial to perform 3D variability analysis in CryoSPARC to reveal potential conformational transitions. If distinct conformations exist, refining these could offer direct experimental evidence for bias-related dynamics. Otherwise, the rationale for solving another static structure remains unclear. Complementary approaches, such as DEER or FRET, to monitor TM5–TM6 movements, or extended simulations, would further validate the suggested mechanism that uniquely stabilizes an inactive-like TM5–TM6 configuration unfavorable for arrestin binding.
• The proposed mechanism seems to be highly receptor and ligand-specific. At the current state, it is unclear how the findings extend to other GPCRs, as the authors do not articulate a broader model. Probably an additional mechanistic figure would help.
• Additionally, GPR84 lacks the canonical CWxP motif, which the authors often refer to as "conserved" throughout the text, which is misleading. The SYxP suggests a distinct activation mechanism. The authors should discuss this divergence more thoroughly and clarify how their observations could inform general principles of GPCR bias.
• In Figure 1b, ligand definitions are inconsistent. The pharmacological profiles should be clearly stated, e.g., balanced ligand without the need to state structural differences in the text below. Instead, structural differences can be effectively illustrated by visual emphasis rather than extensive textual description.

Reviewer #3

(Remarks to the Author)
The manuscript “Steric control of signaling bias in the immunometabolic receptor GPR84” elucidates a structural and molecular basis of ligand-dependent signaling bias at the immunometabolic GPCR GPR84. The authors profile three closely related orthosteric agonists that display comparable potency and efficacy for Gi-activation but divergent capacities to recruit β-arrestins. Using a NanoBiT-tethering approach, they determine a high-resolution cryo-EM structure of the GPR84–Gi complex bound to the Gi-biased agonist OX04529. Structural analysis locates the ligand in a shared orthosteric pocket, while revealing ligand-specific rearrangements of hydrophobic residues in TM5 and TM6 induced by a bulky 3-trifluoromethyl substituent. Molecular dynamics simulations and site-directed mutagenesis show that these steric perturbations indirectly promote distinct conformational changes in the NPxxY motif responsible for efficient β-arrestin recruitment. The authors conclude that these structural aspects collectively favor G-protein coupling over β-arrestin engagement. A focused SAR/SFSR library varying the aryl 3-substituent establishes a steric-occupancy model that links specific ligand substituents and local residue conformations to predictable biased signaling at GPR84.

This is an interesting mechanistic study of a pharmacologically important aspect of GPCR signaling, namely biased signaling. I appreciate the highly interdisciplinary approach undertaken by the authors and the elegance with which the structural and computational work is predictive and confirmed by pharmacological experiments. Although I do not have the expertise to assess the cryo-EM and computational data in depth, my overall impression is that the manuscript is technically sound.

However, despite these positive aspects, I am less convinced about the broader significance and impact of this work for a wider audience. In its current form, the manuscript presents a mechanism for biased agonism that seems to be a very specific case for GPR84. To enhance its impact and appeal to non-specialists, a revised version of the manuscript might consider
1) demonstrating the generalizability of the proposed structural mechanism for biased agonism; and
2) establish a physiological relevance of GPR84 biased agonism, for instance in demonstrating that the degree of biased agonism translates into gradual enhancement of macrophage phagocytosis.

I have some more technical comments:

1) Heterogeneity in the reference ligands: The authors use many different reference ligands for their signaling assays (capric acid, OX04539, PSB-16671). Please use the same reference ligand throughout the study or better explain why so many different reference ligands are needed. Different reference ligands will make quantification of ligand bias and its interpretation complicated.

2) Quantification of ligand bias: The manuscript is all about ligand bias, however, ligand bias is never quantified. This is state-of-the art in the field and such a quantification should be provided.
3) The authors should control for equal expression levels of the various GPR84 mutants and the wild-type receptors to strengthen data interpretation.
4) The GPR84 mutants L336A and F187A appear to have some degree of spontaneous activity in GTPgammaS assays. This should be mentioned and discussed.

Reviewer #4

(Remarks to the Author)
In this manuscript, the authors propose a molecular mechanism for controlling G protein versus β-arrestin signaling bias in the GPR84 receptor. The authors use a combination of cryo-EM, molecular dynamics simulations, site-directed mutagenesis, and functional assays to study how different ligands affect the structure and signaling properties of this receptor.
The cryo-EM structures of the receptor bound to OX04529 or 6-OAU are virtually identical. However, molecular dynamics simulations reveal that OX04529 increases the distance between Phe187(5.47) and Leu336(6.52) by approximately 2.5 Å. This structural feature is not observed in the cryo-EM data, which raises questions about the conformational sampling in the experimental structures. The authors report that these changes propagate to the cytoplasmic sides of TM6 and TM7, with OX04529 shifting the cytosolic end of TM7 inward while displacing TM6 outward (Figs. 4f-g, Extended Data Figs. 9-10). Unfortunately, the presentation of TM6 and TM7 movements uses different visualization methods, making direct comparison difficult. Furthermore, quantitative measurements for these helical displacements are not clearly provided; visual inspection of the figures suggests movements of approximately 2 Å. The biased ligand appears to stabilize a subtly different receptor conformation—involving rearrangement of two side chains and small helical shifts—but these differences emerge only in molecular dynamics simulations and are absent from the cryo-EM conformational ensemble. This discrepancy between experimental and computational data deserves more attention.
The authors report that OX04529 results in impaired receptor phosphorylation, which predictably leads to reduced β-arrestin recruitment. Yet the impaired GRK-mediated phosphorylation appears to be the primary event, with β-arrestin recruitment simply a downstream consequence. It would be helpful to understand how the subtle structural differences between biased and unbiased ligand-bound states actually affect GRK recognition and catalytic efficiency. Additional questions arise regarding receptor phosphorylation heterogeneity: whether β-arrestins bind to partially phosphorylated receptors, whether distinct receptor populations exist, and whether different binding modes occur. While these questions may be beyond the scope of this work, the claim of having elucidated the molecular mechanism of signaling bias at GPR84 seems too strong.
The structural and SAR data do provide valuable insights for structure-based drug design at this receptor. The molecular dynamics simulations are rigorous, though they primarily extend rather than independently reveal the structural hypotheses derived from the cryo-EM data.
The authors conclude by suggesting their findings "may also be applicable to other class A GPCRs" without providing any supporting evidence. Even a simple sequence conservation analysis of the key residues across class A GPCRs would help substantiate this claim. Without such analysis, the broader applicability remains completely speculative.
The manuscript presents solid data and is well-written, offering useful insights for GPR84-targeted drug design. However, the mechanistic understanding remains incomplete, and the generality of the proposed mechanism needs further investigation to extend beyond this specific receptor.

Version 1:

Reviewer comments:

Reviewer #1

(Remarks to the Author)
The authors have adequately addressed all points raised in the first round of review, which in my perspective renders the revised manuscript acceptable for publication in the present form.

Reviewer #2

(Remarks to the Author)
I thank the authors for their detailed responses and revisions. I particularly appreciate the extended sequence and structural analyses across other class A GPCRs. However, several concerns remain unresolved, and unfortunately, I cannot recommend publication in the current form.

• Why were only ~285 sequences analysed in the generalizability study? Were the olfactory receptors excluded? They are class A GPCRs, and their apparent exclusion should be justified or explicitly stated.
• Regarding conformational heterogeneity: the authors state they performed additional 3D classification but found nothing informative. I disagree with this assessment. Extended Data Figure 4 clearly shows that 17% of particles fall into an alternative class, and judging from the G protein conformation, this class does not appear hopeless for further refinement. I urge the authors to properly analyse this population rather than dismissing it. This is existing data that requires no additional experiments.

Minor points:

• Amino acid annotations are inconsistent across figures. For example, in Figure 2, Ballesteros-Weinstein numbering is used in some panels but absent in panels c, d, and e. BW numbering should be applied consistently throughout all the figures.
• Figure readability and inconsistent font sizes remain an issue, e.g., panels 2e and 3b are difficult to read, and Extended Figure 7 is very low resolution.
• Page 25: ECIS should be spelled out at first use.

Reviewer #3

(Remarks to the Author)
The revised manuscript "*Steric control of signaling bias in the immunometabolic receptor GPR84*" has improved significantly compared to the previous version. I appreciate the considerable effort the authors have invested in addressing my comments. Nevertheless, several issues still need to be addressed. In particular, some points raised previously require further clarification and additional experimentation, as outlined below.

*Physiological relevance of GPR84 biased agonism*:
I appreciate the authors' efforts to include additional impedance and ERK phosphorylation data, which broaden the experimental basis of the study. However, these measurements provide only indirect evidence regarding the physiological consequences of the observed signaling bias. Therefore, I maintain that it would be important to evaluate the balanced and biased agonists in phagocytosis and chemotaxis assays, as previously performed in the authors' earlier publication (10.1021/acschembio.9b00533), in order to more directly establish the physiological relevance of the reported biased agonism.

*Heterogeneity in reference ligands and quantification of ligand bias*:
Using multiple reference ligands complicates the quantification and interpretation of ligand bias. In this particular case, for quantification of ligand bias it is not appropriate to use different reference ligands for the two signaling pathways being compared. Ideally (cf. IUPHAR Review 32), multiple reference ligands should be used for both pathways; at minimum, the same reference ligand should be used for both pathways.

*Control of receptor expression levels*:
The level of receptor expression influences $EC_{50}$ values obtained from functional assays and, consequently, the resulting quantification of signaling bias. This factor needs to be taken into account in the revised version of the manuscript.

Reviewer #4

(Remarks to the Author)
The manuscript has been carefully revised and has improved. The authors have addressed several of my concerns adequately; for instance, the presentation of TM6/TM7 movement data is now more clear, and the justification for why key mechanistic features emerge only in MD is reasonable and consistent with the literature. Their decision to soften their claims is also appreciated.

Regarding the role of MD simulations, I would still encourage the authors to explicitly acknowledge in the discussion that the simulations, by design, start from the cryo-EM-derived structural framework and therefore serve to elaborate and test (rather than independently discover) the proposed mechanism. This would not diminish the work, but would help readers assess how much the MD findings depend on the cryo-EM starting point.

Regarding generalizability, the authors have made a genuine effort. Their analysis demonstrates plausibility rather than proof, but combined with the tempered language now used in the manuscript, this is a reasonable response. Further experimental validation in other receptors would strengthen the broader claim but this is beyond what can fairly be asked in revision.

In summary, the authors have made meaningful improvements, including new data and analyses, softened mechanistic claims, and improved figure presentation. Whether the scope and generality of the findings are sufficient for publication in this journal is ultimately a decision for the editor.

Version 2:

Reviewer comments:

Reviewer #2

(Remarks to the Author)
The authors made satisfactory revisions addressing the previous comments. I recommend accepting the manuscript for publication in its current form.

Reviewer #3

(Remarks to the Author)

I have no further comments to the current manuscript.

**Open Access** This Peer Review File is licensed under a Creative Commons Attribution 4.0 International License, which permits use, sharing, adaptation, distribution and reproduction in any medium or format, as long as you give appropriate credit to the original author(s) and the source, provide a link to the Creative Commons license, and indicate if changes were made.
In cases where reviewers are anonymous, credit should be given to 'Anonymous Referee' and the source.
The images or other third party material in this Peer Review File are included in the article's Creative Commons license, unless indicated otherwise in a credit line to the material. If material is not included in the article's Creative Commons license and your intended use is not permitted by statutory regulation or exceeds the permitted use, you will need to obtain permission directly from the copyright holder.
To view a copy of this license, visit https://creativecommons.org/licenses/by/4.0/

**Response to the reviewers' comments**

We thank all of the reviewers for thoughtful and constructive comments. By considering and where requested performing additional experiments suggested by these comments we believe we have strengthened the manuscript. We have responded to all of the comments in turn in blue below. Changes made to the text and figures of the manuscript are marked in red.

**Reviewer #1 (Remarks to the Author):**

The manuscript by Wang et al. reports an interdisciplinary research project that combines cryoEM, MD simulations and rational synthesis of a novel compound series. The major aim of this study is to mechanistically understand ligand-dependent bias at GPR84 and therefore the abovementioned methodological approaches are combined in a reasonable manner. The authors describe new biased ligands (e.g. OX04529 and OX04954) and provide a rational explanation for the ligand's characteristics by means of MD simulations and mutational studies to support their findings. While I cannot fully judge on the cryoEM part, the MD part, the synthesis, and the pharmacological evaluation is methodologically sound and well-reported.

Given the interesting and relevant topic, the mechanistic insights, the valuable novel compound series and the combined methodology used, I support publication of this study in Nature Communications. However, a few issues have to be addressed before acceptance, as outlined below:

One of the most surprising results is the huge difference in ß-arr-2 recruitment between OX04529 and OX04954. Those compounds are highly similar and only differ in one substituent (-CF3 vs. -CH3). While the authors give some mechanistic insights on how these compounds interact within the binding site, the large functional difference of those two compounds is not well rationalized and could be analyzed in more detail.

We thank Reviewer 1 for your positive comments on our manuscript.

We agree that the magnitude of the difference in β-arrestin-2 recruitment between OX04529 and OX04954 is striking given that they form a matched molecular pair differing only by -CF3 vs -CH3. Signalling bias reflects stabilisation of specific receptor conformational microstates, and small local perturbations arising from differences in ligand sterics and electrostatics could shift the population of microstates disproportionately. In this case, the substitution of -CF3 for -CH3 does significantly increases steric bulk (as evidenced by the difference in calculated van

der Waals radii and distance from L336), which can change the orientation of key amino acids L336 and F187. Consistent with this, our MD analysis indicates that the -CF3 group of OX04529 through changing the conformation of L336 and F187, indirectly disrupts the H-bond between Y332 and N104.

To ensure this difference was not assay-specific, **we have also included new data where we evaluated these ligands in orthogonal cellular readouts relevant to macrophage signalling to further support the functional differences between these ligands**. In ECIS assays in primary mouse BMDMs and human THP-1 derived M1 macrophages, OX04529, OX04954 and OX04539 produced distinct impedance time courses consistent with differential signalling dynamics, whereas no response was observed in BMDMs from GPR84$^{-/-}$ mice (**New Extended Data Fig. 1 – see below**). In TR-FRET ERK1/2 phosphorylation assays, OX04529 maintained a more robust concentration-response profile, whereas OX04954 and OX04539 showed reduced apparent efficacy at higher concentrations (Extended Data Fig. 1c). Together, these analyses provide a mechanistic explanation for how a -CF3 to -CH3 substitution can produce a large divergence in β-arrestin recruitment and associated receptor signalling and trafficking by altering receptor conformational sampling rather than simply changing binding affinity or binding kinetics.

The manuscript text has been revised as follows, and additional data included in Extended Figure 1 and Extended Figure 8:

In time-resolved FRET (TR-FRET)-based ERK1/2 phosphorylation assays, OX04539 and OX04954 exhibited a marked reduction in apparent efficacy at higher concentrations, whereas the strongly biased agonist OX04529 preserved a robust signalling response across the concentration range (**Extended Data Fig. 1c**). Furthermore, in a label-free electric cell–substrate impedance sensing (ECIS) assay, which records rapid, real-time alterations in cell morphology, OX04529, OX04954 and OX04539 elicited distinct impedance signals in bone marrow–derived macrophages (BMDMs) and THP-1–derived M1 macrophages, which display the highest GPR84 expression among M0, M1 and M2 subsets (**Extended Data Fig. 1d,e**). The biased agonist OX04529 consistently exhibited a pronounced delay in signal decay following the initial response, and the corresponding area under the curve (AUC) was significantly greater than that observed for the non-biased agonist OX04539 (**Extended Data Fig. 1f,g**). The moderate biased ligand OX04954 displayed an intermediate rate of decay and AUC between these two agonists, establishing a consistent trend whereby the three ligands

differentially modulate signalling dynamics in primary macrophages consistent with reduced receptor internalisation. By contrast, stimulation of $GPR84^{-/-}$ BMDM-derived M1 macrophages with these ligands failed to elicit discernible response over a 30-min recording period (**Extended Data Fig. 1h**), indicating that the differential signalling profiles are dependent on GPR84 expression and activation.

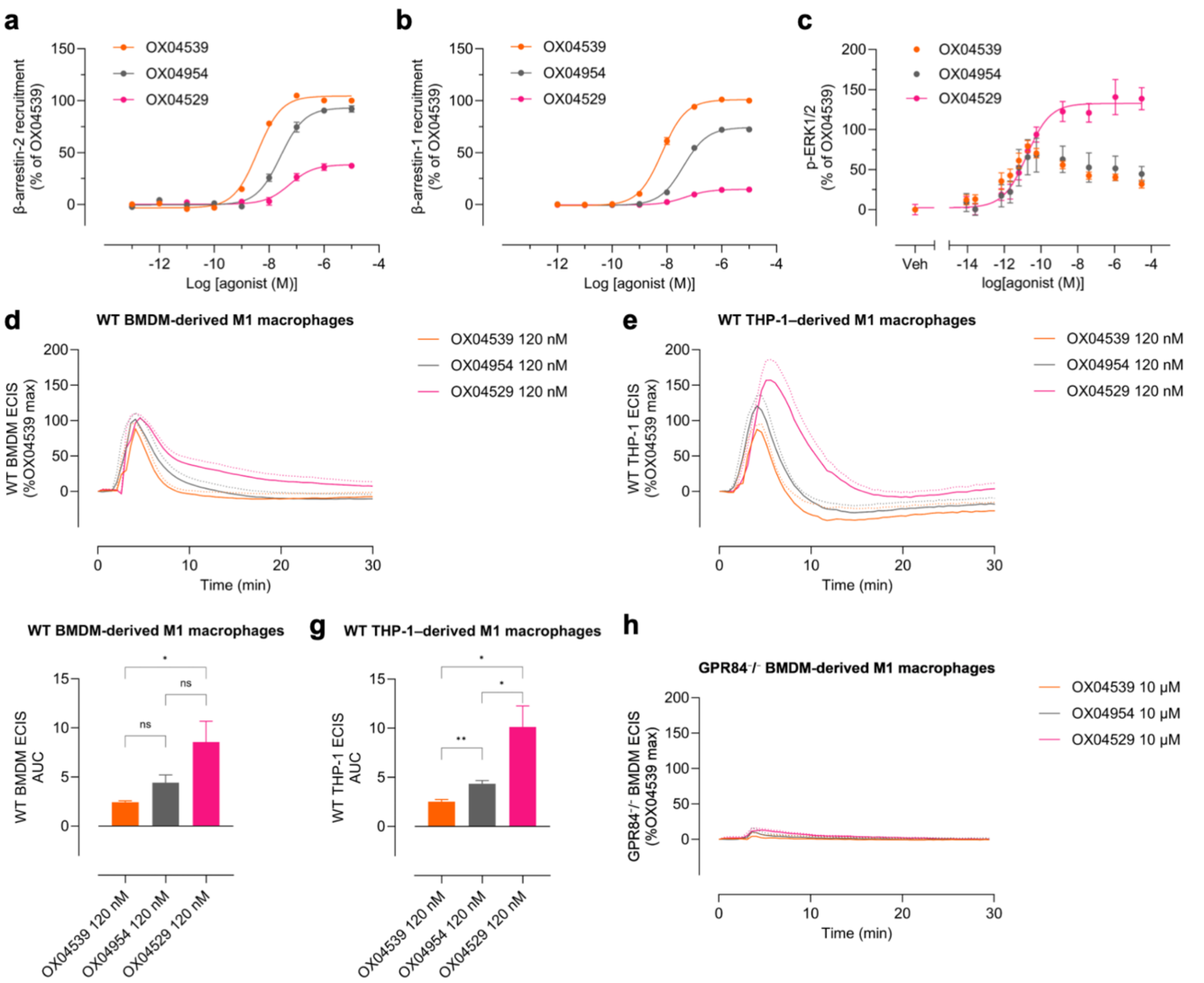


**Extended Data Fig. 1:** Interactions between GPR84 and either (a), β-arrestin-1 or (b), β-arrestin-2 produced by various concentrations of the indicated ligands are shown. Data represent means ± S.E.M. from at least three biologically independent experiments. (c), Phosphorylation of ERK detected after 5 min incubation of the indicated agonists. Data represent means ± S.E.M. from three biologically independent experiments. Electric cell-substrate impedance sensing assays at tested at 120 nM (~$EC_{90}$) (d), WT BMDM-derived M1 macrophages, (e), WT THP-1 derived M1 macrophages or (h), $GPR84^{-/-}$ BMDM-derived M1 macrophages (tested up to 10 μM). (f) and (g) compare the area under the curve between each

compound at corresponding cell lines. *p < 0.05, **p < 0.01. One-way ANOVA followed by Tukey's multiple comparison test.

In support of the β-arrestin recruitment and receptor phosphorylation assays, internalisation assays detecting the trafficking of GPR84 from the plasma membrane to the early endosomal compartment demonstrated that OX04529 behaved as a low-efficacy partial agonist at the wild type receptor, in contrast to the full agonist OX05439 (**Extended Data Fig. 8c**). By comparison, substitution of the sterically bulky leucine residue with alanine, at the Leu336Ala GPR84 mutant, relieved this constraint, thereby the efficacy of OX04529 was markedly enhanced, reaching levels comparable to those observed with OX05439 (**Extended Data Fig. 8d**). These differential phosphorylation profiles are consistent with the distinct signaling dynamics observed in ECIS assays (**Extended Data Fig. 1d-g**), where OX04529 showed prolonged impedance responses indicative of reduced receptor desensitization.

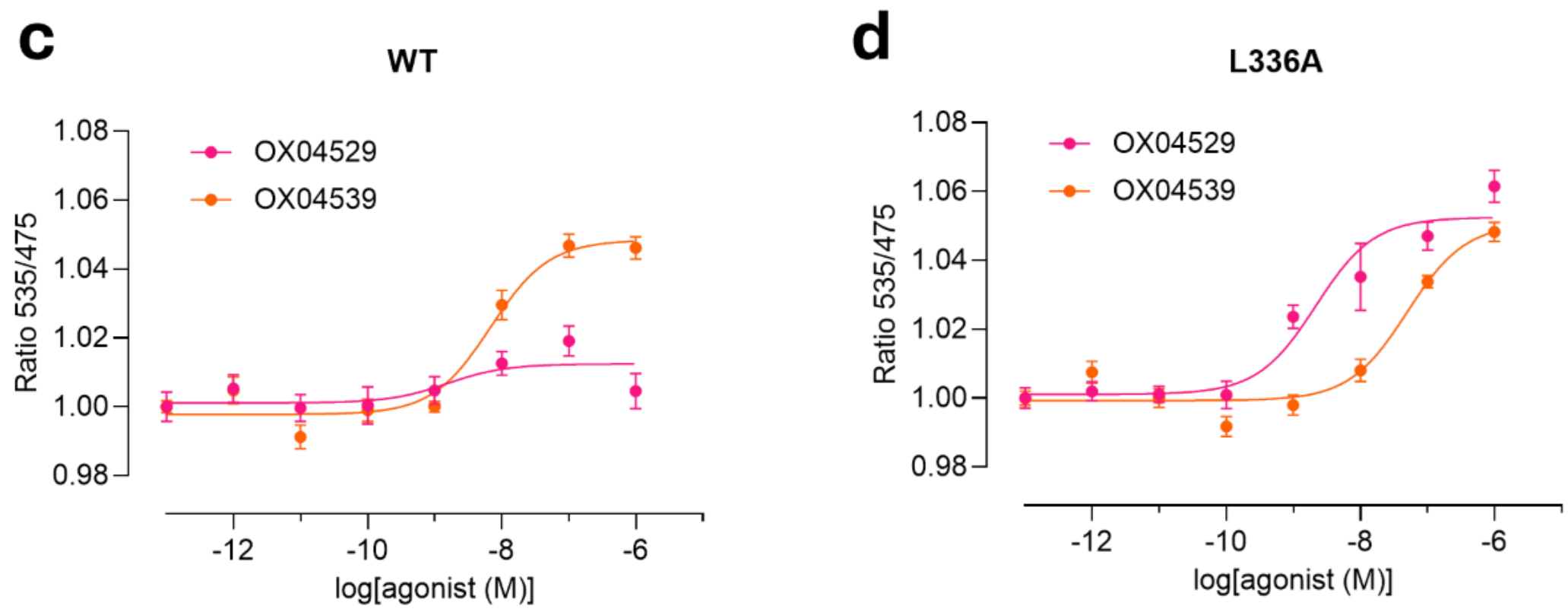


**Extended Data Fig. 8:**

**c,** OX04539 can induce internalization of wild type GPR84 receptor, while OX04529 is much less effective.

**d**, OX04529 and OX04539 induce equivalent receptor internalization at Leu336$^{6.52}$Ala GPR84.

2) There is a discrepancy in the data for OX04529 shown in Fig 1d and Fig 3f.

The assay in Fig. 1d is an enzyme complementation-based β-arrestin recruitment assay (PathHunter® β-Arrestin Assay) that integrates β-arrestin signalling over a 90-min period in CHO cells stably overexpressing hGPR84, whereas Fig. 3f depicts β-arrestin recruitment

measured at a single 5-min time point in transiently transfected HEK293T cells; this distinction in assay format and temporal integration accounts for the apparently lower efficacy observed in Fig. 1d relative to Fig. 3f. We have now added the assay details in each figure legend to specify which system was used.

It is important to note that the two sets of data were produced using very distinct methods of measuring recruitment of the arrestin to the receptor. Although the quantitative details vary to some degree the key feature, which is observed using each approach, is that the effectiveness of OX04529 to promote receptor-arrestin interactions at wild type GPR84 is much lower than for OX04539 and that the ability of OX04529 to promote interactions with arrestin proteins is markedly enhanced at the Leu336Ala (original manuscript) and also Leu336Val (new data) forms of GPR84.

3) The molecular library of OX04529 derivatives is well-thought and rational. The discussion of the SAR, however, is not very detailed and misses a structural explanation (or hypothesis) of the reported effects. For example, the halogen series gave a surprising result, where a methyl, or fluorine substituent gave similar results in ß-arr-2 recruitment, which was significantly different compared to bromine or chlorine substituents. One would expect a rank order according the size of the halogen atom, but this is not the case.

We have now added a more detailed SAR discussion, focusing mainly on the trends of ethyl, vinyl, alkynyl, of F, Br, Cl and compared them to methyl, and discussed the correlation between van der Waals volume and bias factor.

The following highlighted text was added into the revised manuscript:

Analysis of the correlation between a range of physicochemical parameters and measured cAMP inhibition and β-arrestin-2 recruitment revealed striking patterns (Fig. 5e, f, Extended Data Fig. 12). β-arrestin-2 efficacy showed a strong negative correlation with the calculated van der Waals volume of the ligand but not with ligand lipophilicity (Fig. 5e). Extending the carbon chain in the 3-substituent beyond methyl was found to be sufficient to reduce efficacy in β-arrestin-2 recruitment, where ethyl (OX04961), vinyl (OX04962), alkynyl (OX04954) and branched alkyl derivatives OX04964, OX04965 and OX04966 all showed no significant change in efficacy in β-arrestin-2 recruitment compared to OX04929. Intriguingly, the alkynyl analogue OX04954, which has a smaller van der Waals volume than the corresponding ethyl (OX04961) and vinyl (OX04962) substituents, showed lower β-arrestin-2 recruitment efficacy.

This may be because the ethyl and vinyl groups can adopt a conformation that orients the substituent away from L336$^{6.52}$ through carbon-carbon bond rotation. However, the conjugation of the vinyl group with the aromatic ring locks its conformation, in contrast to ethyl, giving a rank order ethyl > vinyl > alkynyl. Notably, there was no apparent correlation with electronegativity or electron withdrawing capacity of the substituents (Extended Data Fig. 12). With a larger van der Waals volume, Cl and Br substituted ligands showed lower β-arrestin-2 recruitment efficacy than the methyl substituted analogue (OX04954), whereas the smaller F substituted analogue, appears to recruit β-arrestin-2 with a slightly higher efficacy than methyl substituted derivative, giving a rank order F > Me > Cl > Br that negatively correlates with their calculated van der Waals volume. Successive replacement of the fluorine substituents within the trifluoromethyl group of OX04529 to yield OX04959 (CHF2), and OX04960 (CH2F) increased significantly the efficacy in β-arrestin-2 recruitment assays and showed a trend of CH3 > CH2F > CHF2 > CF3 (**Fig. 5d**) This trend can be rationalized by the decreasing conformational freedom in the substituent as successive fluorine atoms are introduced, increasing the energy barrier for the substituent to orient the fluorine atoms away from L336$^{6.52}$ and reduce steric clash.

…

Whilst displaying reducing potency in [35S]GTPγS binding assays compared with OX04529, OX04959 (CHF2), and OX04960 (CH2F) (Extended Data Table 3) these ligands did not display reduced efficacy in this assay of Gi-activation.

….

To obtain a comprehensive quantification of signalling bias across this ligand series, we determined bias factors together with ΔΔLog(Emax/EC50) for all compounds exhibiting measurable β-arrestin-2 activity. A strong, statistically significant positive correlation was observed between van der Waals volume and ΔΔLog(Emax/EC50) (**Extended Fig. 11a,c**).

4) The role of L336 is described as essential for the biased properties of reported compounds. This could be easily extended to further support the model. E.g. a L336V mutation could be interesting to look for a graded effect on bias (L336, L336V, L336A). Another possibility would be to test ligands with larger substituents on the L336A mutant (e.g. OX04964). These are some suggestions to strengthen the study, but I don’t want to push for a lot of new experiments.

We thank the reviewer for your suggestion, and we have now added more compounds into our BRET-based β-arrestin-1 assays at the L336A mutant and, additionally, we have added data using a L336V mutant.

We have added the following discussion and some figures in Extended Data Figure 11:

Consistent with our hypothesis, stimulation of wild type, L336A, and L336V GPR84 receptors with these three ligands in BRET-based β-arrestin-1 recruitment assays revealed a progressive increase in efficacy as the substituent size decreased from the sterically bulky $CF_3$ group to the smaller $CF_2H$, $CH_2F$, and $CH_3$ motifs across all three receptor backgrounds. For each ligand, a clear efficacy trend was observed, progressing from low efficacy at the wild-type receptor, to intermediate efficacy at L336V, and to enhanced efficacy at L336A, in some cases exceeding the efficacy of OX04539 (**Extended Fig. 11e.f**).

….

In addition, BRET-based β-arrestin-1 recruitment assays were performed using the bulkier ligands OX4965 and OX4966 at wild-type, L336V, and L336A GPR84 receptors. These experiments demonstrated that elimination of a single carbon atom from 1-methylcyclopropyl substituent OX04966, provided sufficient spatial accommodation to permit higher β-arrestin-1 recruitment (**Extended Fig. 11e,f**).

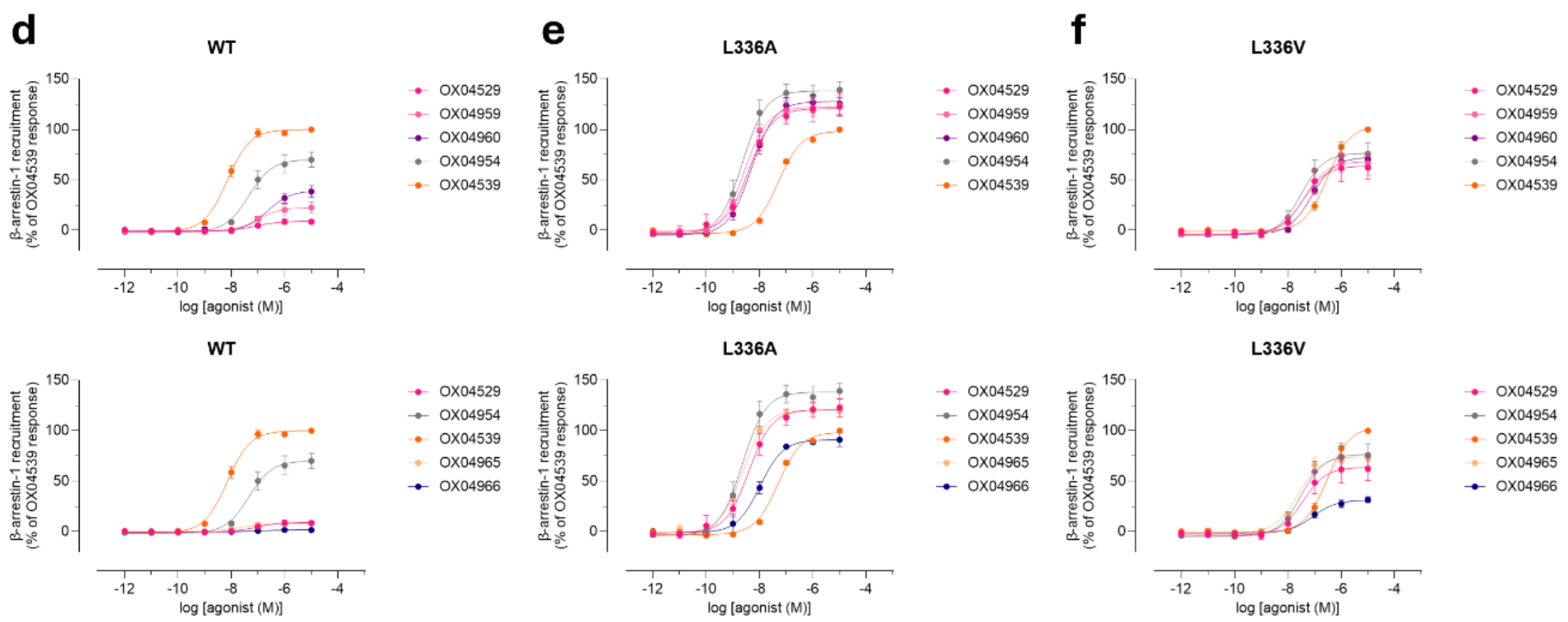


**Extended Data Fig. 11:**

BRET-based β-arrestin-1 recruitment to **d**, GPR84 wild type (WT), **e**, Leu336$^{6.52}$Ala and **f**, Leu336$^{6.52}$Val mutants in response to OX04529 analogues.

5) Please provide bias factors for the reported compounds.

We have now provided bias factors following ‘Community guidelines for GPCR ligand bias: IUPHAR review 32’ and performed a correlation analysis between bias factor and van der Waals volume (Extended Data Fig. 11).

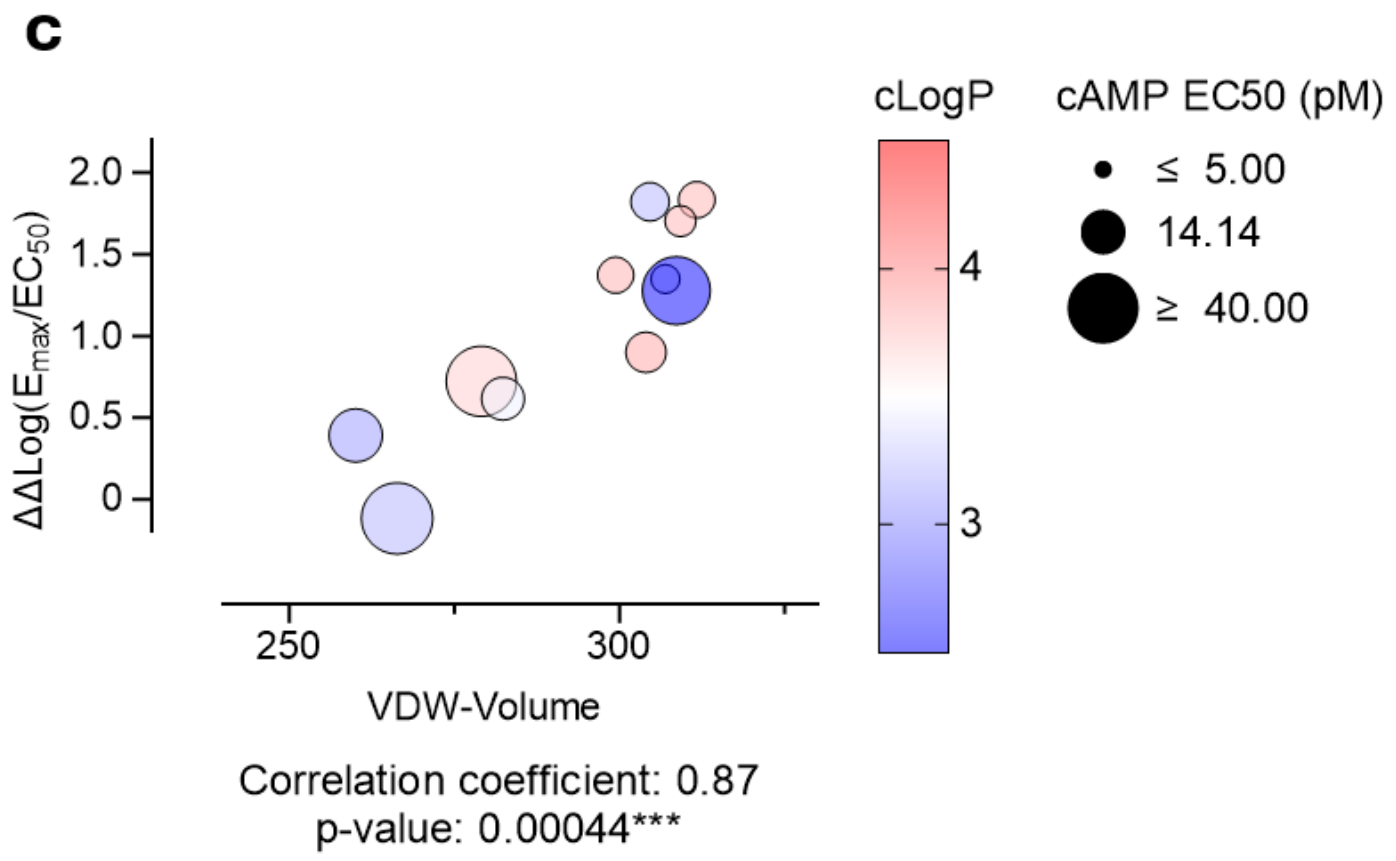


**a**

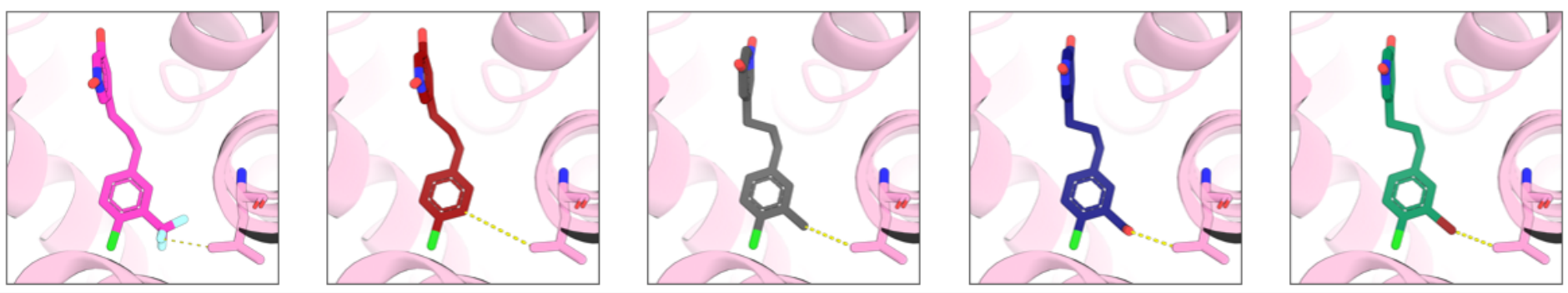

| Agonist | OX04529 | OX04540 | OX04954 | OX04955 | OX04956 |
|---|---|---|---|---|---|
| Aryl 3-substituent | $CF_3$ | H | Me | MeOH | Br |
| Distance between L336[6.52] (Å) | 3.7 (dock) | 5.8 | 4.6 | 3.7 | 4.3 |
| $pEC_{50}$ ± SEM<br>$\%E_{max}$ ± SEM | 10.92 ± 0.18<br>97 ± 0.2 | 10.69 ± 0.09<br>97 ± 0.7 | 10.90 ± 0.23<br>98 ± 1.8 | 10.45 ± 0.20<br>101 ± 1.5 | 11.04 ± 0.12<br>98 ± 2.1 |
| $pEC_{50}$ ± SEM<br>$\%E_{max}$ | ND<br>10 ± 1.5 | 6.96 ± 0.17<br>81 ± 5.3 | 7.03 ± 0.24<br>71 ± 1.3 | 6.58 ± 0.23<br>21 ± 2.9 | 6.86 ± 0.39<br>34 ± 6.0 |
| $\Delta\Delta Log(E_{max}/EC_{50})$ ± SEM | ND | 0.67 ± 0.09 | 0.86 ± 0.17 | 1.42 ± 0.15 | 1.5 ± 0.22 |
| Bias Factor | ND | 4.63 | 7.32 | 26.05 | 31.85 |
| VDM-V (docking) | 269.736 | 239.72 | 256.784 | 264.84 | 257.872 |
| VDM-V (elements) | 316.74 | 267.55 | 287.46 | 310.9 | 302.7 |
| cLogP | 3.9476 | 3.0993 | 3.4432 | 2.502 | 3.8245 |
| LLE | 6.9757 | 7.5907 | 7.4535 | 7.9447 | 7.2155 |

6) The authors state that their findings on the bias mechanism for GPR84, is transferable to other GPCRs. This should be elaborated a bit more in the discussion. The observation that ligand-dependent bias could be achieved by ‘extended binding modes’, or more bulky substituents compared to a lead structure has been reported previously for different receptors, e.g. 10.1021/acschembio.7b00275, 10.1038/nchembio.2527, 10.1038/s41589-018-0145-x, 10.1021/acsptsci.0c00069, 10.1111/febs.15783, 10.1073/pnas.2112397119 among others).

In response to the reviewer's very helpful suggestion, we have performed a sequence alignment at TM5, 5.47 and TM6, 6.52 for 286 class A GPCRs, and we have precisely investigated the AF2 models of these GPCRs with TM6, 6.52 similar (Isoleucine, Valine) or identical (Leucine) to GPR84. In the investigated 34/286 models, 73% (25/34) showed a predicted binding pocket very close to TM6, 6.52 (< 5.0 Å). We have also added the suggested references into our discussion.

An additional discussion has been added into the manuscript:

**Potential extension to other class A hGPCRs**

To further investigate the applicability of this mechanism by disrupting the side chain confirmation dynamics of amino acid residues at positions 6.52 and 5.47, we performed sequence alignment of 285 class A hGPCRs excluding GPR84. Among these, 13/285 (4.6%) receptors shared an identical residue at position 6.52, and 187/285 (65.6%) shared an identical residue at position 5.47 (**Extended Data Table 8**). At the toggle switch motif, 248/285 (87.0%) receptors carried an aromatic tryptophan, phenylalanine, or tyrosine at position 6.48, whereas the accompanying residue of the toggle switch was more variable. To gain further insights into the structural relevance of these findings, we analysed in detail the potential binding pockets of class A GPCRs with a leucine at position 6.52 using AlphaFold2 models (**Extended Data Fig. 12**). In 8/13 (61.5%) of these receptors, the leucine was located within 5 Å of the predicted orthosteric binding site and was predicted to adopt a conformation oriented inwards into the pocket, mirroring the arrangement observed for $L336^{6.52}$ in GPR84 when bound to the non-biased agonist 6-OAU. Furthermore, when we expanded the analysis to 22/285 receptors bearing an alternative branched aliphatic isoleucine or valine at position 6.52, 17/21 (81.0%) displayed a predicted orthosteric binding pocket where the side chain was predicted to also adopt a conformation oriented inwards into the pocket. These observations suggest that the residues at position 6.52 are a potentially targetable site in a subset of GPCRs and warrant further investigation through rational ligand design based on compounds with known or predicted binding modes.

**Extended Data Table 8: Sequence alignment of 285 class A GPCRs of 2 residues, 5.47 and 6.52. Boxes highlighted in orange or brown represent identical or similar residues at 5.47 respectively, boxes highlighted in sky blue or dark blue mean identical or similar residues at 6.52 respectively.**

| Gene | TM5, 5.47 | TM6, 6.52 | Gene | TM5, 5.47 | TM6, 6.52 | Gene | TM5, 5.47 | TM6, 6.52 |
|---|---|---|---|---|---|---|---|---|
| ACKR1 | L | G | GPR15 | F | N | MLNR | F | H |
| ACKR2 | F | N | GPR150 | F | F | MRGPRD | V | S |
| ACKR3 | F | H | GPR151 | F | W | MRGPRE | L | G |
| ACKR4 | F | N | GPR152 | G | Q | MRGPRF | L | G |
| ADORA1 | V | H | GPR153 | G | L | MRGPRG | V | V |
| ADORA2A | V | H | GPR160 | L | V | MRGPRX1 | L | G |
| ADORA2B | V | H | GPR161 | A | M | MRGPRX2 | L | G |
| ADORA3 | I | S | GPR162 | L | L | MRGPRX3 | L | G |
| ADRA1A | F | F | GPR17 | F | H | MRGPRX4 | L | G |
| ADRA1B | F | F | GPR171 | F | H | MTNR1A | F | N |
| ADRA1D | F | F | GPR173 | F | I | MTNR1B | F | N |
| ADRA2A | F | F | GPR174 | F | H | NMBR | F | H |
| ADRA2B | F | F | GPR176 | V | A | NMUR1 | F | H |
| ADRA2C | F | F | GPR18 | F | H | NMUR2 | Y | H |
| ADRB1 | F | F | GPR182 | F | H | NPBWR1 | F | H |
| ADRB2 | F | F | GPR183 | Y | H | NPBWR2 | F | H |
| ADRB3 | F | F | GPR19 | F | H | NPFFR1 | Y | W |
| AGTR1 | F | Q | GPR20 | F | H | NPFFR2 | Y | W |
| AGTR2 | F | H | GPR21 | L | I | NPSR1 | Y | F |
| APLNR | F | H | GPR22 | F | S | NPY1R | Y | T |
| AVPR1A | F | F | GPR25 | F | S | NPY2R | Y | H |
| AVPR1B | F | F | GPR26 | F | V | NPY4R | Y | H |
| AVPR2 | F | F | GPR27 | F | V | NPY5R | Y | H |
| BDKRB1 | F | H | GPR3 | F | T | NPY6R | Y | I |
| BDKRB2 | F | Q | GPR31 | F | F | NTSR1 | F | H |
| BRS3 | Y | H | GPR32 | F | N | NTSR2 | F | H |
| C3AR1 | F | H | GPR33 | F | H | OPN1LW | C | T |
| C5AR1 | F | Q | GPR34 | F | H | OPN1MW | C | A |
| C5AR2 | F | H | GPR35 | F | H | OPN1SW | F | A |
| CCKAR | F | F | GPR37 | F | N | OPN3 | L | I |
| CCKBR | F | Y | GPR37L1 | F | N | OPN4 | F | S |
| CCR1 | L | N | GPR39 | F | Q | OPN5 | L | A |
| CCR10 | F | S | GPR4 | F | H | OPRD1 | F | H |
| CCR2 | L | N | GPR42 | F | N | OPRK1 | F | H |
| CCR3 | L | N | GPR45 | F | S | OPRL1 | F | Q |
| CCR4 | L | N | GPR50 | F | N | OPRM1 | F | H |
| CCR5 | L | N | GPR52 | L | I | OXGR1 | F | H |
| CCR6 | F | N | GPR55 | F | H | OXTR | Y | F |
| CCR7 | F | N | GPR6 | F | A | P2RY1 | F | H |
| CCR8 | L | N | GPR61 | F | F | P2RY10 | F | H |
| CCR9 | F | N | GPR62 | F | G | P2RY11 | C | H |
| CCRL2 | L | N | GPR63 | F | T | P2RY12 | F | H |
| CHRM1 | F | N | GPR65 | Y | H | P2RY13 | F | H |
| CHRM2 | F | N | GPR68 | F | H | P2RY14 | F | H |
| CHRM3 | F | N | GPR75 | F | G | P2RY2 | F | H |
| CHRM4 | F | N | GPR78 | F | V | P2RY4 | F | H |
| CHRM5 | F | N | GPR82 | I | S | P2RY6 | F | H |
| CMKLR1 | F | H | GPR83 | Y | N | P2RY8 | F | N |
| CMKLR2 | Y | H | GPR85 | F | L | PARD3 | F | N |
| CNR1 | T | L | GPR87 | F | H | PAWR | C | N |
| CNR2 | I | L | GPR88 | L | V | PRLHR | Y | H |
| CX3CR1 | F | N | GRPR | Y | H | PROKR1 | F | Y |
| CXCR1 | F | N | HCAR1 | F | V | PROKR2 | F | Y |
| CXCR2 | F | N | HCAR2 | F | V | PTAFR | F | H |
| CXCR3 | F | H | HCAR3 | F | V | PTGDR | L | I |
| CXCR4 | L | Y | HCRTR1 | Y | S | PTGDR2 | F | H |
| CXCR5 | F | H | HCRTR2 | Y | S | PTGER1 | V | L |
| CXCR6 | F | N | HRH1 | F | F | PTGER2 | L | T |
| CYSLTR1 | F | H | HRH2 | F | F | PTGER3 | G | L |
| CYSLTR2 | C | H | HRH3 | F | T | PTGER4 | S | V |
| DRD1 | F | F | HRH4 | F | S | PTGFR | L | L |
| DRD2 | F | F | HTR1A | F | F | PTGIR | L | T |
| DRD3 | F | F | HTR1B | F | F | QRFPR | F | H |
| DRD4 | F | F | HTR1D | F | F | RHO | F | A |
| DRD5 | F | F | HTR1E | F | F | RRH | F | S |
| EDNRA | F | H | HTR1F | F | F | RXFP1 | N | F |
| EDNRB | F | H | HTR2A | F | F | RXFP2 | N | F |
| F2R | F | N | HTR2B | F | F | RXFP3 | F | Q |
| F2RL1 | F | N | HTR2C | F | F | RXFP4 | F | H |
| FFAR1 | F | N | HTR4 | F | F | S1PR1 | F | F |
| FFAR2 | F | N | HTR5A | F | F | S1PR2 | F | F |
| FFAR3 | F | N | HTR6 | F | F | S1PR3 | F | F |
| FFAR4 | F | I | HTR7 | F | F | S1PR4 | F | F |
| FPR1 | F | Q | KISS1R | Y | Q | S1PR5 | F | F |
| FPR2 | F | Q | LGR4 | N | A | SSTR1 | F | Y |
| FPR3 | F | E | LGR5 | N | A | SSTR2 | F | Y |
| FSHR | N | S | LGR6 | N | A | SSTR3 | F | Y |
| GALR1 | Y | H | LHCGR | N | S | SSTR4 | F | Y |
| GALR2 | Y | H | LPAR1 | N | L | SSTR5 | F | F |
| GALR3 | Y | H | LPAR2 | S | Q | SUCNR1 | F | H |
| GHSR | F | H | LPAR3 | N | L | TAAR1 | F | F |
| GNRHR | F | Y | LPAR4 | F | N | TAAR2 | F | F |
| GPBAR1 | L | V | LPAR5 | F | N | TAAR3P | F | F |
| GPER | F | N | LPAR6 | F | N | TAAR5 | F | T |
| GPR101 | I | C | LTB4R | F | H | TAAR6 | F | S |
| GPR119 | G | L | LTB4R2 | F | H | TAAR8 | F | T |
| GPR12 | F | T | MAS1 | V | R | TAAR9 | F | L |
| GPR132 | F | H | MAS1L | I | S | TACR1 | Y | H |
| GPR135 | Y | C | MC1R | F | F | TACR2 | Y | H |
| GPR139 | Y | I | MC2R | F | V | TACR3 | Y | H |
| GPR141 | A | Q | MC3R | F | F | TBXA2R | G | L |
| GPR142 | Y | V | MC4R | F | F | TRHR | Y | R |
| GPR146 | Y | Y | MC5R | F | F | TSHR | N | S |
| GPR148 | G | S | MCHR1 | F | Y | UTS2R | I | W |
| GPR149 | F | M | MCHR2 | F | H | XCR1 | F | N |

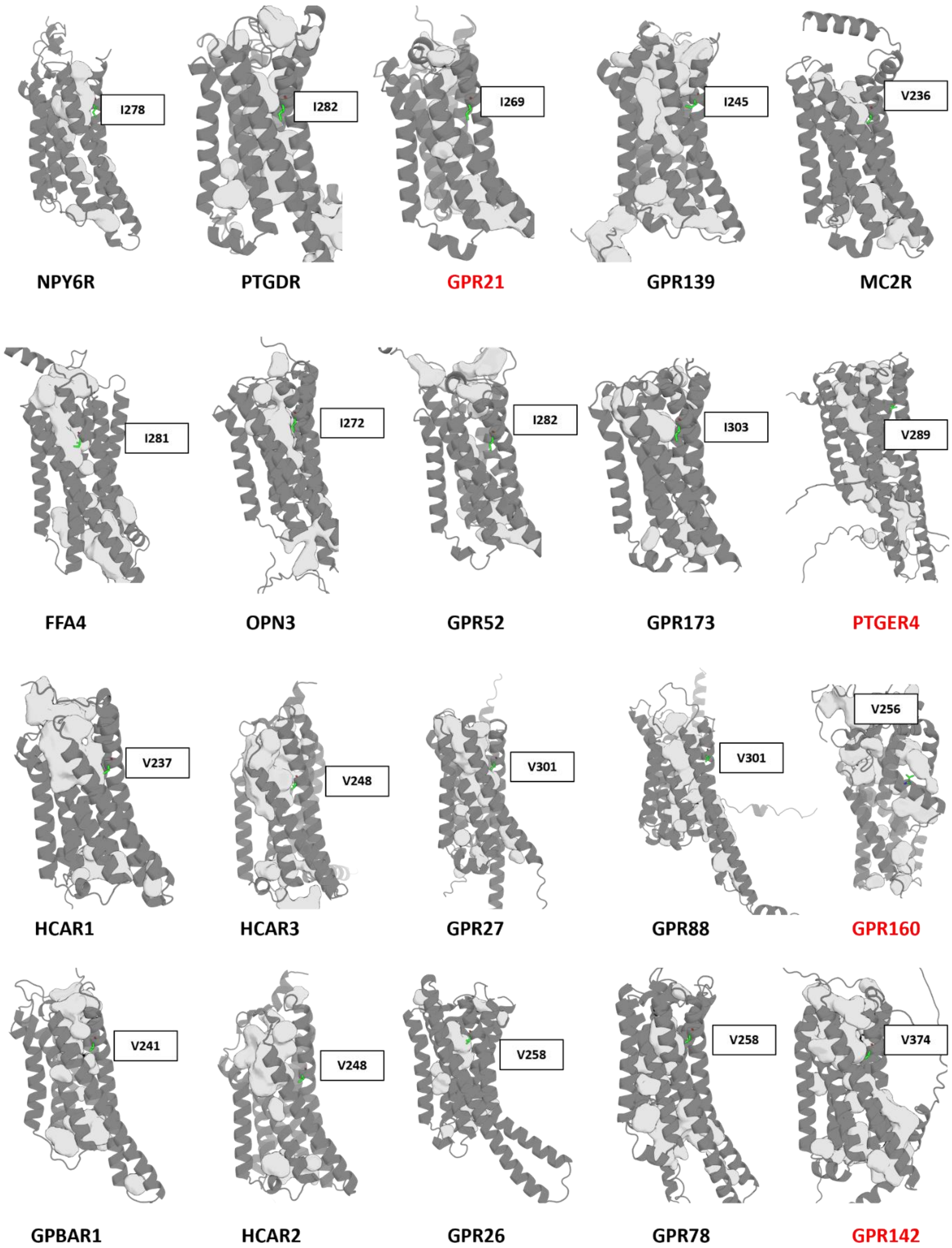

I278
NPY6R
I282
PTGDR
I269
GPR21
I245
GPR139
V236
MC2R
I281
FFA4
I272
OPN3
I282
GPR52
I303
GPR173
V289
PTGER4
V237
HCAR1
V248
HCAR3
V301
GPR27
V301
GPR88
V256
GPR160
V241
GPBAR1
V248
HCAR2
V258
GPR26
V258
GPR78
V374
GPR142

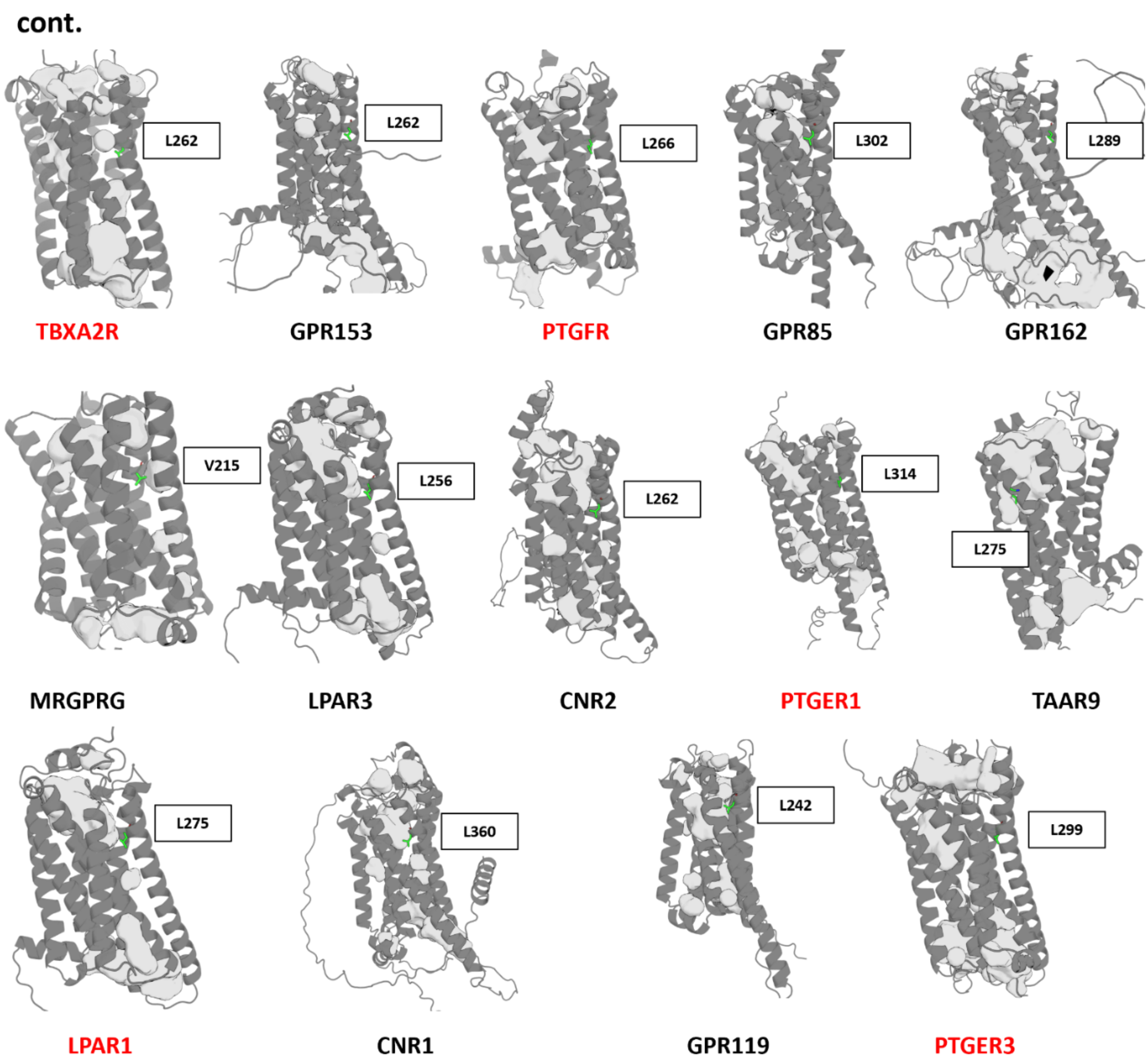


**Extended Data Fig. 12: Investigating predicted binding pockets on AF2 models across class A GPCRs with identical or similar residues at position 6.52 revealed 73% (name highlighted in black, the remainder in red) of these receptors having the 6.52 residue (green stick) close (< 5Å) to the predicted orthosteric binding pockets (grey surface).**

We also added to the discussion:

Additionally, from a sequence alignment analysis followed by binding pocket mining, we found that 81% of the searched class A GPCRs with a steric leucine, isoleucine or valine at TM6.52 have a close proximity to the pocket. In summary, we elucidate the molecular mechanism of the OX04529 series for rational design of G protein–biased GPR84 agonists, by integrating complementary functional, structural and computational dynamic analyses.

7) In the methods section, there are two paragraphs about docking (GLIDE and MOE). It remains unclear which docking program was used for which part of the study.

Docking in GLIDE was done to prepare the OX04539 bound GPR84 complex based on the cryoEM with OX04529 (for MD simulations). Docking in MOE was done to compare the 3-aryl substituent position among OX04529 analogues (for Figure 5).

**We have now added these descriptions in the paragraph and in the figure legends:**

Systems include GPR84 bound to G protein-biased ligand OX04529 (with and without G protein), partially biased OX04954, reference agonists (6-OAU and OX04539 computationally docked into the OX04529 cryo-EM structure using GLIDE, along with 6-OAU native cryo-EM structure), ligand-free receptor states derived from cryo-EM structures, and point mutants (Phe187$^{5.47}$Ala and Leu336$^{6.52}$Ala).

Furthermore, when measuring the interatomic distance between the most distal heavy atom of the aryl 3-substituent and of the Leu336$^{6.52}$ side chain from the OX04529-GPR84 cryo-EM structure and comparing this with the measured interatomic distance taken from MOE docking models of a selection of ligands, we observed a strong correlation between interatomic distance and β-arrestin-2 recruitment efficacy (**Fig. 5f**).

**Reviewer #2 (Remarks to the Author):**

This manuscript investigates the mechanism of biased agonism in GPR84 using the cryo-EM structure of the GPR84–Gi complex, complemented by mutagenesis, MD simulations, and analysis of ligand analogs. GPR84 is an immunometabolic GPCR with potential relevance in inflammation and cancer immunotherapy. The topic is important, and the work provides valuable mechanistic insight. However, several points require clarification or further analysis to substantiate the conclusions.

• The cryo-EM map appears to be of high quality and shows lipid-like densities. However, only a single cholesterol molecule was modeled despite the evident density between TM4 and TM5 on the intracellular side. Lipids can modulate GPCR dynamics, and the unmodeled density may influence the ICL3 region or G-protein coupling. The authors should either model this additional lipid or at least discuss its potential structural and functional role.

We thank the reviewer for this thoughtful suggestion. We tried to model cholesterol molecules into the cryo-EM density at the locations indicated. However, in each case the density was either not strong enough to support placement of a full cholesterol molecule or lacked clear polar interactions with the receptor that would justify confident modeling. For these reasons, we believe it is more appropriate to leave these densities unmodeled, which is consistent with good practices in GPCR structural biology. It is also possible that these features correspond to other lipids or sterols, or even detergent molecules from protein purification.

We agree with the reviewer that lipids, including cholesterol, can influence GPCR dynamics. However, defining the specific role of cholesterol or other lipids in modulating GPR84 signaling falls outside the scope of the present study.

• The structure was solved with a G-protein–biased ligand and unsurprisingly resembles previously reported active-state complexes. However, GPCRs are dynamic, and biased ligands often stabilize specific conformations not captured in static structures. This has been well established by NMR studies of adrenergic receptors. Although the authors used MD simulations, additional conformational states might already be present in the cryo-EM data. Could authors examine the alternative class seen in their dataset during the processing (Extended Data Figure 4)? It can also be beneficial to perform 3D variability analysis in CryoSPARC to reveal potential conformational transitions. If distinct conformations exist, refining these could offer direct experimental evidence for bias-related dynamics. Otherwise,

the rationale for solving another static structure remains unclear. Complementary approaches, such as DEER or FRET, to monitor TM5–TM6 movements, or extended simulations, would further validate the suggested mechanism that uniquely stabilizes an inactive-like TM5–TM6 configuration unfavorable for arrestin binding.

We thank the reviewer for this constructive suggestion. We agree that GPCRs are highly dynamic proteins and that biased agonists can stabilize conformations not readily captured in static cryo-EM structures.

**Regarding cryo-EM heterogeneity analysis:** Our structural analysis focused on the GPR84-$G_i$ signalling complex, where the receptor is stabilized by $G_i$ in a fully active conformation. In this context, 3DVA and related heterogeneity analyses are limited in their ability to resolve ligand-specific receptor states, as they typically reveal conformational variations within the G protein or the overall complex rather than distinct receptor conformations. For example, recent studies on opioid receptor-$G_i$ complexes (Nature, PMID: 41193810; bioRxiv, PMID: 40502042) identified conformational diversity in $G_i$ during the activation process rather than receptor-specific states. Similarly, our previous work on the ghrelin receptor-$G_i$ complex (PMID: 34737341) revealed two conformations differing in $G_i$ coupling orientation. Nevertheless, we performed additional heterogeneity analyses on our cryo-EM data but did not identify new 3D classes with sufficient resolution to model GPR84 in alternative conformations. We conclude that $G_i$ stabilizes GPR84 in a relatively homogeneous conformation regardless of the bound agonist.

**Rationale for our structural and computational approach:** $G_i$-bound cryo-EM structures represent a thermodynamic endpoint where the G protein stabilizes a homogeneous active conformation, explaining why our OX04529-bound structure resembles other active-state complexes. The value of our structure lies in providing an experimentally validated starting point for MD simulations. To probe ligand-specific conformational dynamics, we performed extensive MD simulations (ten 2-μs replicates) of GPR84 alone bound to either OX04529 or 6OAU. These simulations revealed distinct conformational states: OX04529 results in a unique increase in the TM5-TM6 distance at the orthosteric binding site (Fig 2B, 2C, 3A), which alters hydrogen bonding between the critical 'toggle-switch' residue Tyr332$^{6.48}$ and other key residues. This mechanism was validated through mutagenesis and pharmacological assays and prevents β-arrestin recruitment by impairing ICL3-phosphorylation.

**Experimental validation:** Our simulations identified a distinct arrangement of L336$^{6.52}$ and F187$^{5.47}$ in the binding pocket, along with differences in other critical activation motifs. We validated this mechanistic model through mutagenesis studies, and the functional data strongly support our hypothesis. Furthermore, guided by our model, we designed and validated new biased ligands with tuneable efficacy ranging from full agonism to inactive for β-arrestin signalling—a novel contribution of this study.

**Regarding additional biophysical approaches:** We agree that DEER or single-molecule FRET could provide complementary insight into receptor dynamics. However, these techniques require specialized instrumentation and extensive optimization beyond the current expertise of our team.

We believe that the combination of cryo-EM structure, extensive MD simulations, and functional validation provides a coherent and robust foundation for the mechanistic model of OX04529-induced GPR84 biased signalling.

• The proposed mechanism seems to be highly receptor and ligand-specific. At the current state, it is unclear how the findings extend to other GPCRs, as the authors do not articulate a broader model. Probably an additional mechanistic figure would help.

We agree with the reviewer that the proposed mechanism could be highly ligand- and receptor-specific. In response to this reviewer's comment and those of reviewer 1, we have now added a class A GPCR sequence alignment to gain some understanding of generalisability of this mechanism (**Extended Data Table 8**). Additionally, we analysed the AF2 model of the 285 GPCRs with an identical (leucine) or similar (valine, isoleucine) residue at 6.52 (**Extended Data Fig. 13**) and added the following discussion:

See response to reviewer 1, comment 6 above for alterations to the manuscript.

• Additionally, GPR84 lacks the canonical CWxP motif, which the authors often refer to as "conserved" throughout the text, which is misleading. The SYxP suggests a distinct activation mechanism. The authors should discuss this divergence more thoroughly and clarify how their observations could inform general principles of GPCR bias.

We thank reviewer 2 for your comment.

The tryptophan in the toggle switch CWxP motif is highly conserved across class A GPCRs that we analysed (195/285), whereas in GPR84 the tryptophan is replaced by phenylalanine,

which is a minor case (46/285). We have changed the text to SYxP and removed 'conserved' when discussing this motif in GPR84.

The canonical W6.48 (toggle switch within the CWxP motif) relating to GPCR bias has been extensively characterised in other class A GPCRs, including the μ-opioid receptor (10.1016/j.molcel.2021.07.033), κ-opioid receptor (10.1038/s41467-023-37041-7), δ-opioid receptor (10.1038/s41467-024-52601-1) and the CB1 cannabinoid receptor (10.1016/j.cell.2023.11.017). Our study further supports the concept that perturbation of a non-canonical toggle switch/CWxP micro-switch can effectively modulate receptor activation; however, in contrast to these canonical systems, we describe an indirect mechanism in which steric repulsion displaces 5.47 and 6.52, thereby propagating conformational changes that underlie GPR84 signalling.

• In Figure 1b, ligand definitions are inconsistent. The pharmacological profiles should be clearly stated, e.g., balanced ligand without the need to state structural differences in the text below. Instead, structural differences can be effectively illustrated by visual emphasis rather than extensive textual description.

We have changed the text in Fig. 1b into a bias factor number, and a descriptor e.g. Non-biased agonist/reference agonist/G-protein biased agonist.

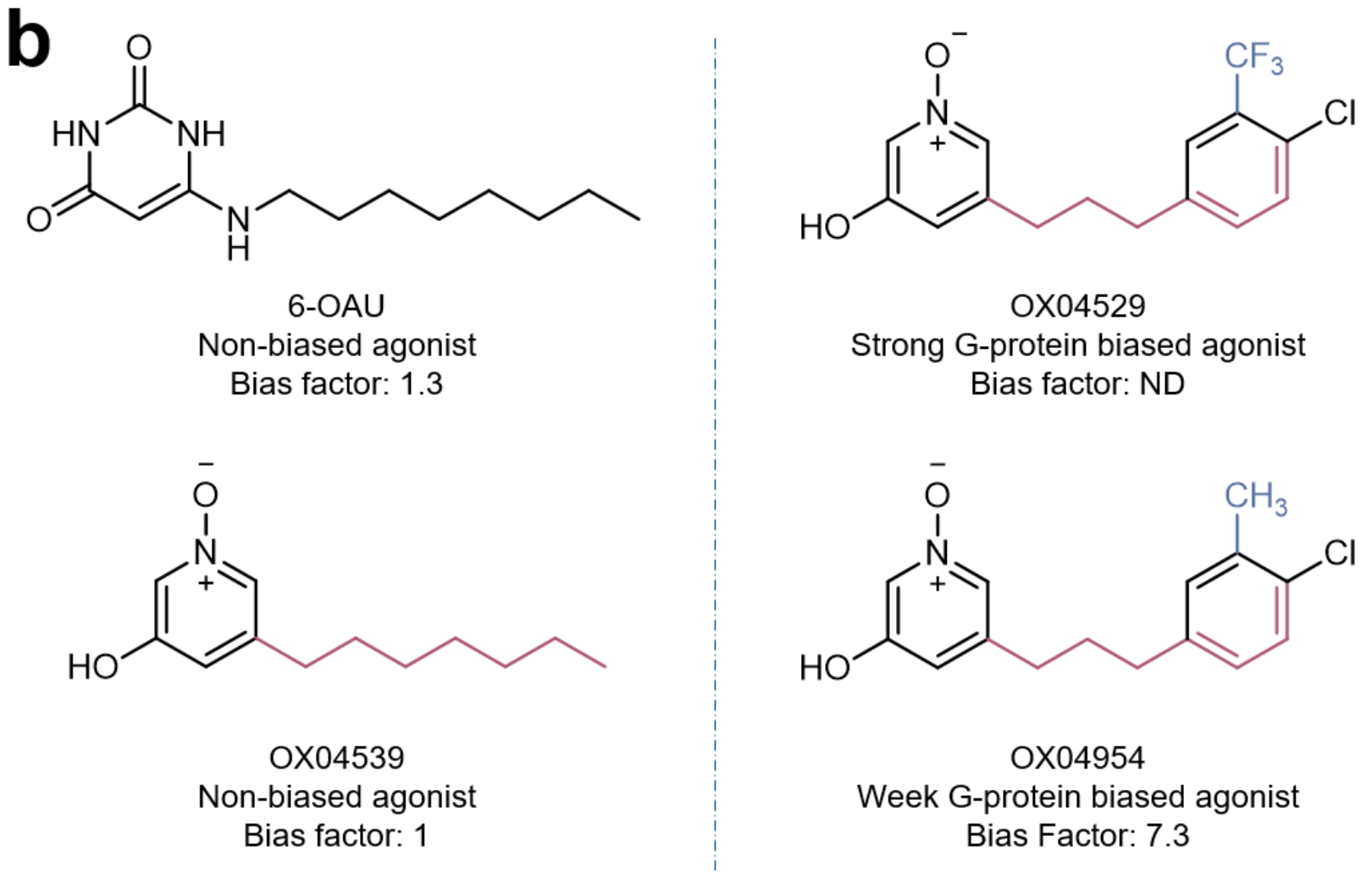

**Reviewer #3 (Remarks to the Author):**

The manuscript "Steric control of signaling bias in the immunometabolic receptor GPR84" elucidates a structural and molecular basis of ligand-dependent signaling bias at the immunometabolic GPCR GPR84. The authors profile three closely related orthosteric agonists that display comparable potency and efficacy for Gi-activation but divergent capacities to recruit β-arrestins. Using a NanoBiT-tethering approach, they determine a high-resolution cryo-EM structure of the GPR84–Gi complex bound to the Gi-biased agonist OX04529. Structural analysis locates the ligand in a shared orthosteric pocket, while revealing ligand-specific rearrangements of hydrophobic residues in TM5 and TM6 induced by a bulky 3-trifluoromethyl substituent. Molecular dynamics simulations and site-directed mutagenesis show that these steric perturbations indirectly promote distinct conformational changes in the NPxxY motif responsible for efficient β-arrestin recruitment. The authors conclude that these structural aspects collectively favor G-protein coupling over β-arrestin engagement. A focused SAR/SFSR library varying the aryl 3-substituent establishes a steric-occupancy model that links specific ligand substituents and local residue conformations to predictable biased signaling at GPR84.

This is an interesting mechanistic study of a pharmacologically important aspect of GPCR signaling, namely biased signaling. I appreciate the highly interdisciplinary approach undertaken by the authors and the elegance with which the structural and computational work is predictive and confirmed by pharmacological experiments. Although I do not have the expertise to assess the cryo-EM and computational data in depth, my overall impression is that the manuscript is technically sound.

However, despite these positive aspects, I am less convinced about the broader significance and impact of this work for a wider audience. In its current form, the manuscript presents a mechanism for biased agonism that seems to be a very specific case for GPR84. To enhance its impact and appeal to non-specialists, a revised version of the manuscript might consider

1) demonstrating the generalizability of the proposed structural mechanism for biased agonism; and

We thank Reviewer 3 for your careful reading of our manuscript and their suggestions for improving the document.

For point (1), we have now performed a sequence alignment with 286 class A hGPCRs focusing on the key residues 5.47 and 6.52, and it has shown that at 6.52 there are 34/286 GPCRs have similar size and property (valine and isoleucine) or identical (leucine) residues (**Extended Data Table 8**). With a deeper investigation by digging into AF2 model of these 34 GPCRs, 25 out of 34 showed that the predicted orthosteric binding pocket is close (< 5.0 Å) to 6.52 (**Extended Data Fig. 13**).

See response to Reviewer 1, comment 6 for alterations made to the manuscript.

2) establish a physiological relevance of GPR84 biased agonism, for instance in demonstrating that the degree of biased agonism translates into gradual enhancement of macrophage phagocytosis.

For point (2), we have previously shown that the macrophage phagocytosis is dependent on G-protein pathway (10.1021/acschembio.9b00533). To assess the difference between the strongly biased, medium biased and non-biased agonists, we firstly performed a label-free electric cell–substrate impedance sensing (ECIS) assay, which determines real-time changes in cellular morphology. In this assay, the strongly biased, medium biased and non-biased agonists showed distinct impedance response in BMDM-derived M1 macrophages and THP-1-derived M1 macrophages, which showed the highest GPR84 expression among M0, M1 and M2 macrophages. Furthermore, no visible response was detected in GPR84$^{-/-}$ BMDM-derived M1 macrophages in the same assay within a 30-min detection period, indicating that the differences in signalling are from the activation of GPR84 (**Extended Data Fig. 1**).

In addition, we performed TR-FRET-based ERK1/2 phosphorylation assays. In these assays, non-biased (OX04539) and medium biased (OX04954) agonists exhibited a marked drop of efficacy at high concentrations, whereas the highly biased agonist OX04529 maintained a robust signalling response across the concentration range (**Extended Data Fig. 1c**). We suggest that this behaviour reflects GPR84 desensitisation, with the strongly biased agonist failed to desensitise the receptor at 5 min even at a high concentration, thereby sustaining downstream ERK1/2 signalling.

See response to reviewer 1, comment 1 for alterations made to the manuscript in response to this important comment.

I have some more technical comments:

1) Heterogeneity in the reference ligands: The authors use many different reference ligands for their signaling assays (capric acid, OX04539, PSB-16671). Please use the same reference ligand throughout the study or better explain why so many different reference ligands are needed. Different reference ligands will make quantification of ligand bias and its interpretation complicated.

We are using different reference ligands for the following reasons:

Capric acid was initially thought to be the endogenous ligand for GPR84, albeit with low potency, which is why 100 uM capric acid has been used to normalise efficacy in our cAMP assays. Capric acid failed to activate β-arrestin in any assay due to its low affinity for GPR84.

For GTPγS assays and the mutagenesis studies, we use an allosteric agonist, PSB-16671, which binds to a very different site on GPR84, as the positive control to confirm whether the mutagenesis is affecting binding of the ligands or receptor function.

As for β-arrestin assays (in both CHO cells and HEK293T cells), OX04539 was used as a reference throughout as it has the highest efficacy and activity so far in our assay, and the previously used PSB-16671 (fully biased and unable to promote interactions with β-arrestin isoforms) or capric acid (low potency) failed to activate β-arrestin.

2) Quantification of ligand bias: The manuscript is all about ligand bias, however, ligand bias is never quantified. This is state-of-the art in the field and such a quantification should be provided.

We have now quantified signalling bias in accordance with the Community guidelines for GPCR ligand bias: IUPHAR Review 32, determining ΔΔLog(Emax/EC50) for each independent experiment (n = 3) in the cAMP and β-arrestin assays to obtain ΔΔLog(Emax/EC50) ± SEM, from which the final bias factors were derived.

Additionally, we conducted correlation analyses between van der Waals volume and ΔΔLog(Emax/EC50), demonstrating that the calculated bias factors exhibit a strong, positive association with van der Waals volume.

3) The authors should control for equal expression levels of the various GPR84 mutants and the wild-type receptors to strengthen data interpretation.

Simply assessing the amount of various mutated forms of a receptor, whether via immunoblotting or some type of ELISA and comparing this to wild type, does not address whether a mutation alters folding or function of the receptor. Hence, in cases where a receptor has both orthosteric and allosteric ligands available, we adopt the strategy of assessing whether the expressed mutated receptor protein is functional or not (see Nature. 2025 Jul;643(8074):1428-1438. doi: 10.1038/s41586-025-09186-6). Herein, for mutants of the orthosteric pocket we ensure that responses to an allosteric ligand (in this case PSB-16671) are unaffected or only slightly modified. This defines the expressed protein is correctly folded and expressed and then allows the degree of effect of the specific mutation on the orthosteric ligand(s) to be measured and quantified with confidence.

4) The GPR84 mutants L336A and F187A appear to have some degree of spontaneous activity in GTPgammaS assays. This should be mentioned and discussed.

We thank the reviewer for your comment and noting the modest effect that the F187A and, rather more so, the L336A mutation. We cannot provide a definitive understanding of this but as we show that these mutations clearly alter communication from the orthosteric binding pocket to functional outcomes in terms of arrestin recruitment it is not surprisingly that they also have modest effects on communication to molecular switches associated with G protein activation. We now note in the text the effects that the reviewer highlights.

**Reviewer #4 (Remarks to the Author):**

In this manuscript, the authors propose a molecular mechanism for controlling G protein versus β-arrestin signaling bias in the GPR84 receptor. The authors use a combination of cryo-EM, molecular dynamics simulations, site-directed mutagenesis, and functional assays to study how different ligands affect the structure and signaling properties of this receptor.

The cryo-EM structures of the receptor bound to OX04529 or 6-OAU are virtually identical. However, molecular dynamics simulations reveal that OX04529 increases the distance between Phe187(5.47) and Leu336(6.52) by approximately 2.5 Å. This structural feature is not observed in the cryo-EM data, which raises questions about the conformational sampling in the experimental structures.

Because it is extremely challenging to solve structures of GPR84 alone bound to different agonists, our structural analysis in this study focused on the GPR84-Gi signaling complex, with the goal of defining the molecular basis for OX04529 binding. In this complex, the receptor is

stabilized by Gi in a homogenous fully active conformation, which may not fully reflect the distinct biased conformations induced by the biased agonist. Our structure reveals subtle but meaningful structural differences in the binding of OX04529 compared to 6-OAU.

To investigate ligand-specific conformational dynamics, we performed extensive MD simulations of the receptor alone in complex with the biased agonist. These simulations generated a consistent and experimentally testable model for the mechanism underlying OX04529-induced biased signaling. Similar approaches have been used to elucidate biased signaling mechanisms for other GPCRs.

Our proposed mechanism centers on a distinct arrangement of the L336$^{6.52}$ and F187$^{5.47}$ pair within the binding pocket, together with other key structural features associated with receptor activation. We conducted functional assays by mutagenesis to evaluate this model, and the results strongly support our mechanism. Moreover, using insights from the model, we designed and experimentally validated new biased ligands with tunable efficacy, ranging from full agonism to lack of activity specifically for β-arrestin signaling. This is a particularly novel aspect of the study, since we are not aware of similar ligand design for other GPCRs.

Altogether, our cryo-EM structure, MD simulations, and functional assays provide a coherent and robust foundation for the mechanistic model of OX04529-induced biased signaling at GPR84. We believe the convergence of these complementary approaches offers compelling support for the proposed mechanism.

The authors report that these changes propagate to the cytoplasmic sides of TM6 and TM7, with OX04529 shifting the cytosolic end of TM7 inward while displacing TM6 outward (Figs. 4f-g, Extended Data Figs. 9-10). Unfortunately, the presentation of TM6 and TM7 movements uses different visualization methods, making direct comparison difficult. Furthermore, quantitative measurements for these helical displacements are not clearly provided; visual inspection of the figures suggests movements of approximately 2 Å. The biased ligand appears to stabilize a subtly different receptor conformation—involving rearrangement of two side chains and small helical shifts—but these differences emerge only in molecular dynamics simulations and are absent from the cryo-EM conformational ensemble. This discrepancy between experimental and computational data deserves more attention.

We thank the reviewer for this observation. In our simulations of GPR84-ligand complexes, we observe an increase in the F187$^{5.47}$–L336$^{6.52}$ Cα distance of approximately 2 Å upon

OX04529 binding. We have updated Extended Figure 5a to include reference lines showing the average distance for non-OX04529 conditions (~6.2 Å) versus OX04529-bound states (~8 Å), providing clear quantitative comparison (**Extended Fig. 5a**).

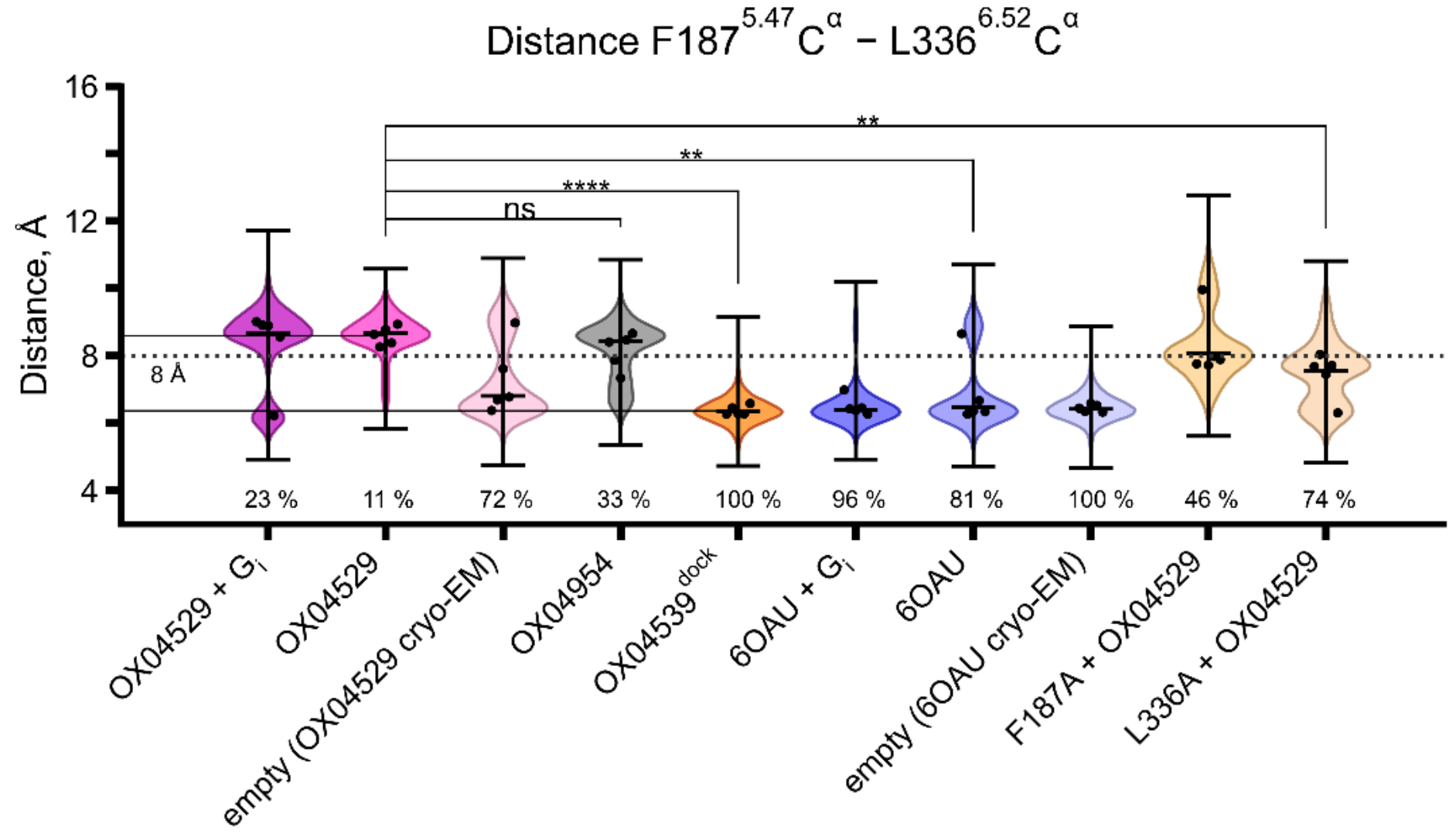


**Extended Fig. 5a**: **F187$^{5.47}$–L336$^{6.52}$ Cα distance in GPR84-ligand complexes.** Reference lines: non-OX04529 average (~6.2 Å, solid) and OX04529-bound (~8 Å, dotted). Percentages indicate frames below 8 Å. ****$p < 0.0001$, **$p < 0.01$, ns = not significant.

Regarding the helical displacements, we have now unified the visualization approach. Figure 4g has been updated to show MD simulation atom density for both TM6 and TM7, with an additional view that more clearly illustrates the inward movement of TM7 (indicated by arrows) (**Extended Fig. 10d**). Quantitatively, Figure 4f shows the distance of the TM7 cytosolic end from the 7TM bundle Cα centre of mass: 8% of the OX04529-bound receptor population adopts a conformation with reduced TM7 distance (~15 Å versus ~16–18 Å for other ligands), corresponding to an inward shift of approximately 1–2 Å (**Extended Fig. 10e**). These measurements confirm the visual observations noted by the reviewer.

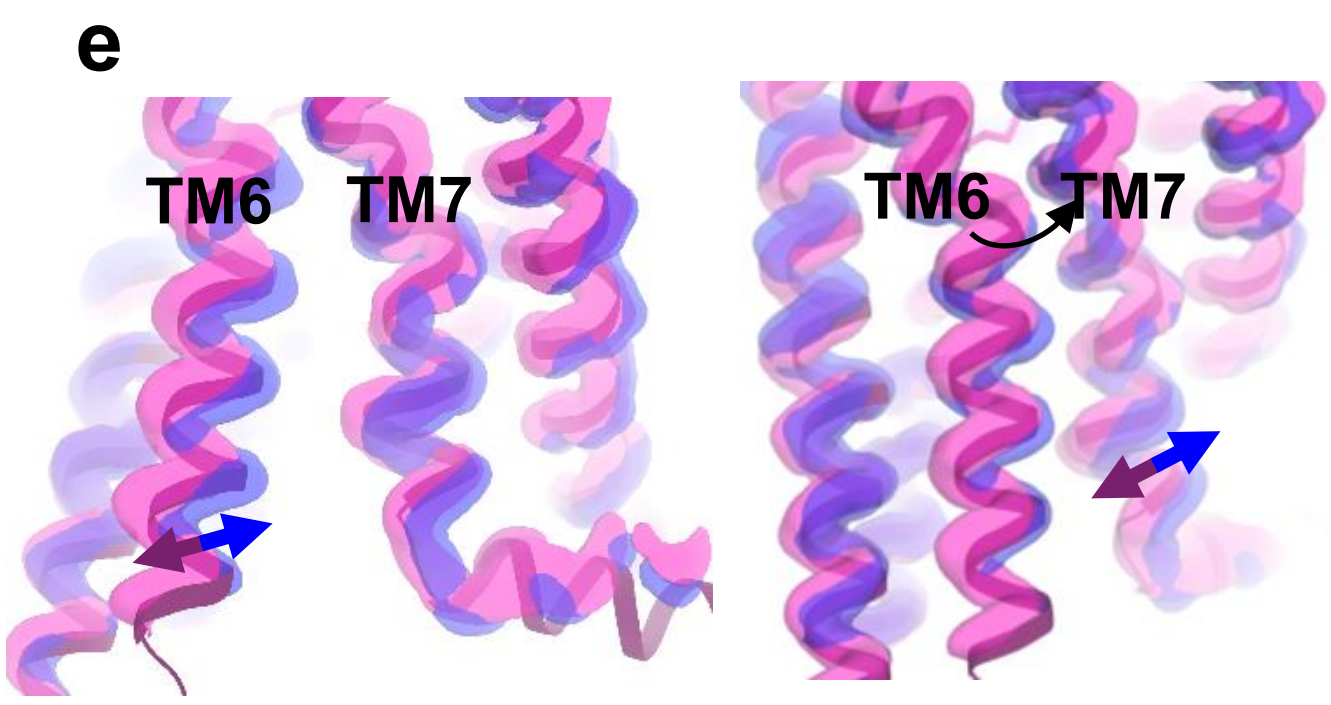

**Extended Figure 10e. MD simulation density visualization of TM6 and TM7 movements in OX04529-bound GPR84.** Arrows indicate outward displacement of TM6 and inward movement of TM7.

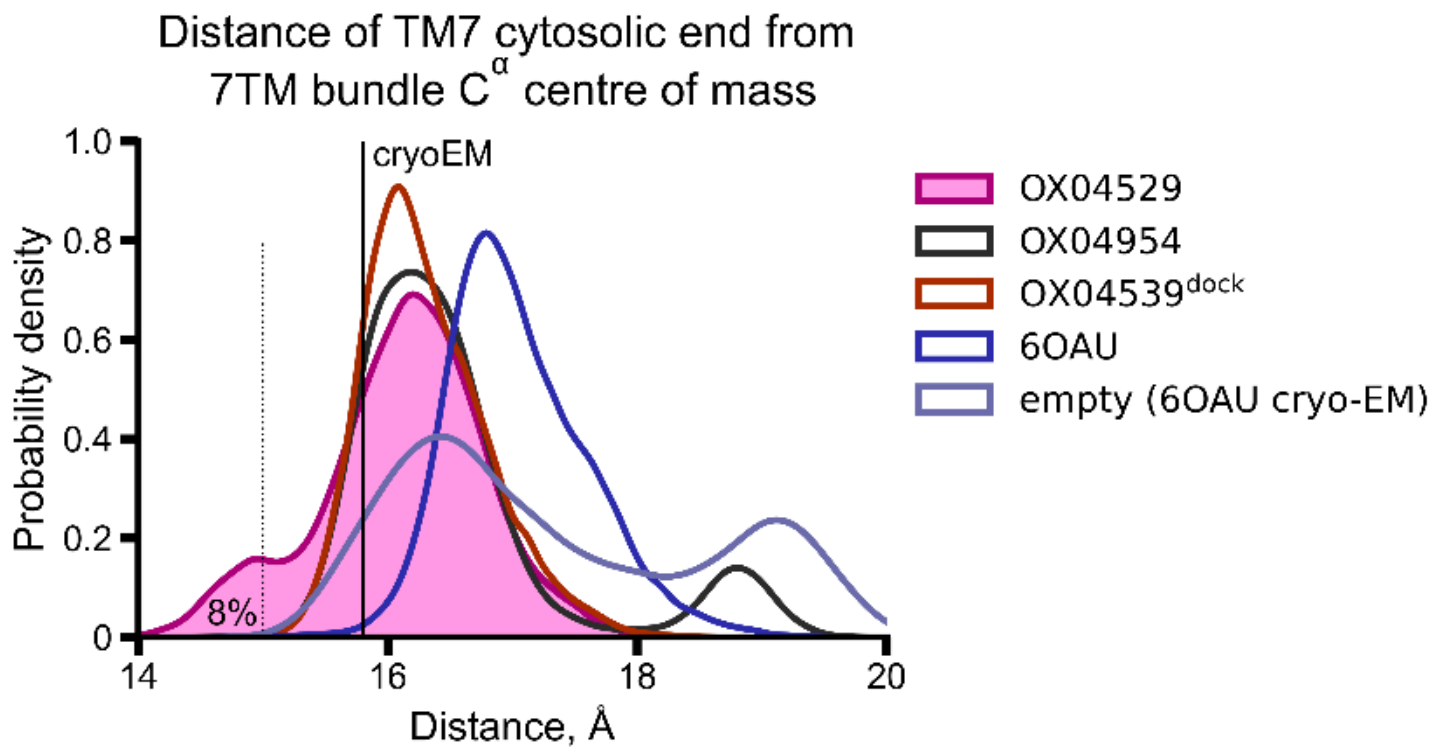


**Extended Figure 10d. TM7 cytosolic end displacement from 7TM bundle centre of mass.** OX04529-bound GPR84 shows 8% of conformations with reduced TM7 distance (~15 Å), indicating inward helix movement compared to other ligands

The authors report that OX04529 results in impaired receptor phosphorylation, which predictably leads to reduced β-arrestin recruitment. Yet the impaired GRK-mediated phosphorylation appears to be the primary event, with β-arrestin recruitment simply a downstream consequence. It would be helpful to understand how the subtle structural differences between biased and unbiased ligand-bound states actually affect GRK recognition and catalytic efficiency. Additional questions arise regarding receptor phosphorylation heterogeneity: whether β-arrestins bind to partially phosphorylated receptors, whether distinct receptor populations exist, and whether different binding modes occur. While these questions may be beyond the scope of this work, the claim of having elucidated the molecular mechanism of signaling bias at GPR84 seems too strong. The structural and SAR data do provide valuable insights for structure-based drug design at this receptor. The molecular dynamics simulations are rigorous, though they primarily extend rather than independently reveal the structural hypotheses derived from the cryo-EM data.

We thank reviewer 4 for your thorough reading of the manuscript, and your thoughtful comments.

We agree that the phrase ‘having elucidated the molecular mechanism of signaling bias at GPR84’ is perhaps too strong, as the mechanism we investigated applies to this series of compounds, and there are other series of biased ligands in the literature that may not share the same molecular mechanism. We have now changed our wording into ‘In summary, we elucidate the molecular mechanism of the OX04529 series for rational design of G protein–biased GPR84 agonists, by integrating complementary functional, structural and computational dynamic analyses.’

The authors conclude by suggesting their findings "may also be applicable to other class A GPCRs" without providing any supporting evidence. Even a simple sequence conservation analysis of the key residues across class A GPCRs would help substantiate this claim. Without such analysis, the broader applicability remains completely speculative.

The manuscript presents solid data and is well-written, offering useful insights for GPR84-targeted drug design. However, the mechanistic understanding remains incomplete, and the generality of the proposed mechanism needs further investigation to extend beyond this specific receptor.

We performed a sequence alignment of 285 class A human GPCRs excluding GPR84, focusing on the toggle switch region and the key residues identified in our data at positions 5.47 and 6.52. We also analysed AF2 models of these receptors (since most lack experimentally determined structures) that share the same (leucine) or similar (valine, isoleucine) residue at position 6.52. Notably, in over 70% of these GPCRs, the predicted binding pocket lies within 5.0 Å of residue 6.52. This indicates that a substantial number of GPCRs possess an accessible 6.52 position that could be targeted via steric clash to induce ligand bias.

See response to reviewer 1, comment 6 for changes made to the manuscript.

Response to reviewers

NCOMMS-25-67869A

**REVIEWER COMMENTS**

**Reviewer #1 (Remarks to the Author):**

*The authors have adequately addressed all points raised in the first round of review, which in my perspective renders the revised manuscript acceptable for publication in the present form.*

**Reviewer #2 (Remarks to the Author):**

*I thank the authors for their detailed responses and revisions. I particularly appreciate the extended sequence and structural analyses across other class A GPCRs. However, several concerns remain unresolved, and unfortunately, I cannot recommend publication in the current form.*

• *Why were only ~285 sequences analysed in the generalizability study? Were the olfactory receptors excluded? They are class A GPCRs, and their apparent exclusion should be justified or explicitly stated.*

We have clarified the receptor selection in the revised text: 'To assess the potential generality of this mechanism across class A GPCRs, we performed sequence alignment of 285 non-olfactory human receptors beyond GPR84.' Olfactory receptors were excluded due to their distinct evolutionary lineage and limited structural characterisation, which preclude meaningful comparison with conventional class A GPCRs. This is consistent with standard practice in class A GPCR analyses, where olfactory receptors are routinely treated as a separate subfamily.

• *Regarding conformational heterogeneity: the authors state they performed additional 3D classification but found nothing informative. I disagree with this assessment. Extended Data Figure 4 clearly shows that 17% of particles fall into an alternative class, and judging from the G protein conformation, this class does not appear hopeless for further refinement. I urge the authors to properly analyse this population rather than dismissing it. This is existing data that requires no additional experiments.*

We thank the reviewer for this insightful suggestion. We reprocessed the cryo-EM dataset using the 17.1% subset of particles shown in Extended Data Figure 2, which yielded a cryo-EM map at 4.1-Å resolution (**Response Fig. 1a**). Based on this map, we built the model of GPR84 and the Gi protein. Due to the limited resolution, most side chains could not be assigned with high confidence. Nevertheless, following real-space refinement, the overall structure of GPR84 remains largely unchanged with that observed in our 3.0-Å structure (**Response Fig. 1b**). However, we note subtle differences in the conformation of the N-terminal helix of Gαi and the scFv16 compared to the higher-resolution structure, as illustrated in **Response Fig. 1c**. Therefore, this subset of particles was excluded from the final reconstruction to improve the overall resolution of the map.

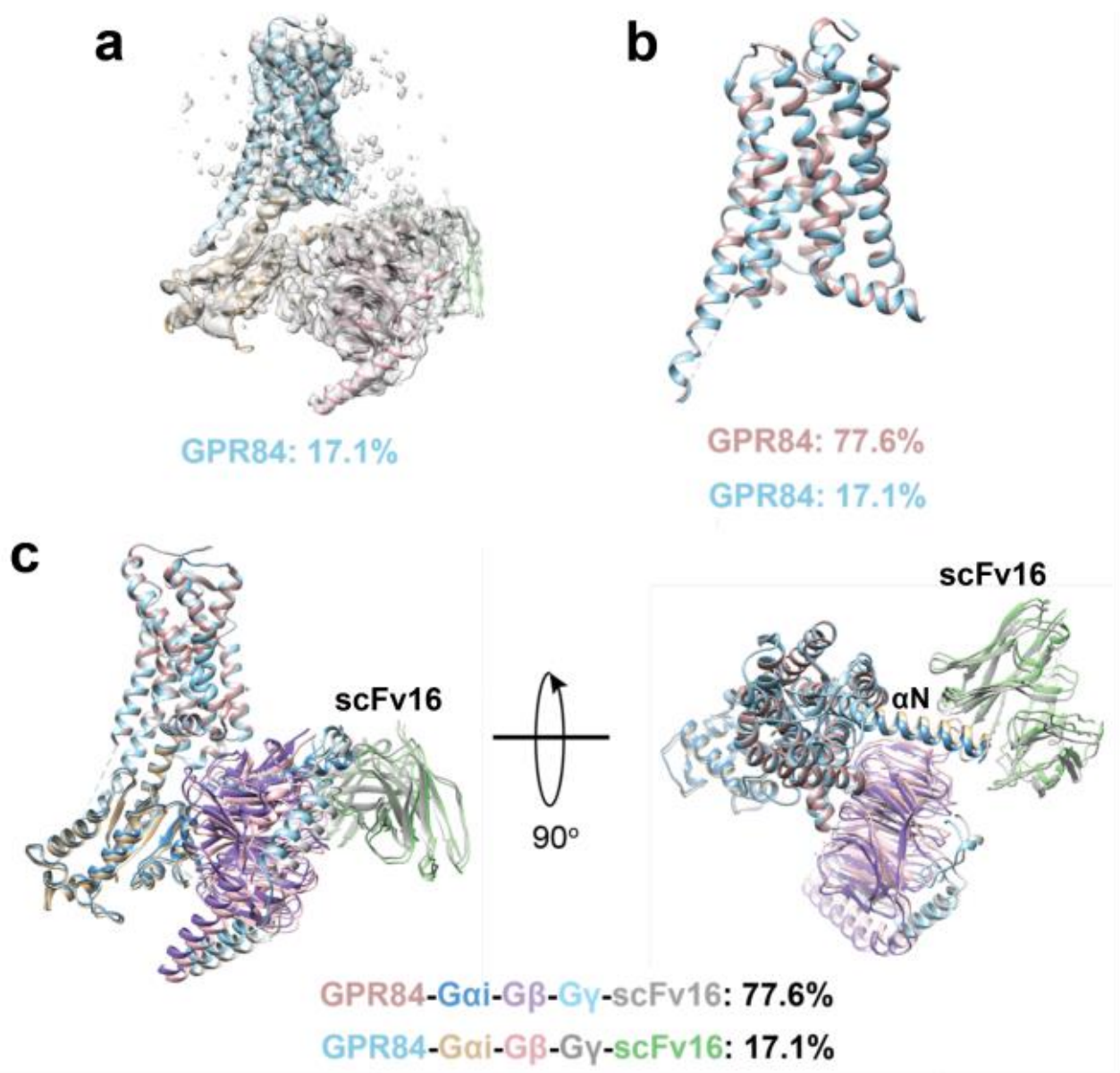


**Fig 1. (a)** Cryo-EM density map at 4.1-Å resolution reconstructed from the 17.1% particle subset that was excluded from the initial map reconstruction. **(b)** Overall structure of GPR84 based on the 4.1-Å map remains largely unchanged compared to the original GPR84 structure obtained from the 3.0-Å map calculated from the 77.6% particle subset. **(c)** Subtle differences are observed in the conformation of the N-terminal helix of Gαi and the scFv16.

As we do not observe meaningful conformational differences within the receptor itself, we have focused our structural analysis on the 3.0-Å structure. The 4.1-Å map has been included in the revised **Extended Data Figure 2 (panel c**).

*Minor points:*

*• Amino acid annotations are inconsistent across figures. For example, in Figure 2, Ballesteros-Weinstein numbering is used in some panels but absent in panels c, d, and e. BW numbering should be applied consistently throughout all the figures.*

Figure 2c, d and e were updated with BW numbering added.

*• Figure readability and inconsistent font sizes remain an issue, e.g., panels 2e and 3b are difficult to read, and Extended Figure 7 is very low resolution.*

Figures 2e, 3b and Extended Figure 7 were updated.

*• Page 25: ECIS should be spelled out at first use.*

ECIS abbreviation is spelled out on page 4.

**Reviewer #3 (Remarks to the Author):**

*The revised manuscript "Steric control of signaling bias in the immunometabolic receptor GPR84" has improved significantly compared to the previous version. I appreciate the considerable effort the authors have invested in addressing my comments. Nevertheless, several issues still need to be addressed. In particular, some points raised previously require further clarification and additional experimentation, as outlined below.*

*Physiological relevance of GPR84 biased agonism*:
*I appreciate the authors' efforts to include additional impedance and ERK phosphorylation data, which broaden the experimental basis of the study. However, these measurements provide only indirect evidence regarding the physiological consequences of the observed signaling bias. Therefore, I maintain that it would be important to evaluate the balanced and biased agonists in phagocytosis and chemotaxis assays, as previously performed in the authors' earlier publication (10.1021/acschembio.9b00533), in order to more directly establish the physiological relevance of the reported biased agonism.*

The intended focus for our manuscript is the development of a predictive, structure-based framework for rationally designing biased GPCR ligands, which in itself addresses a longstanding challenge in chemical biology and drug discovery. In the first revision of the manuscript, in response to the comments of reviewer 3, we included substantially more data confirming that ligands with varying degrees of signalling bias at the receptor translate to distinct effects on signalling and morphological responses in native cells, consistent with our previous published work (Lucy et al., *ACS Chem. Bio.* **2019**).

As reviewer 3 notes, we have already previously published data comparing G-protein-biased and β-arrestin-recruiting ligands demonstrating that these translate to distinct biological responses in immune cells (Lucy et al., *ACS Chem. Bio.* **2019**), work which we cite in our current manuscript. Our intention is to use the chemical tools we describe in this manuscript to address these important questions regarding the biological consequences of signalling bias at GPR84 in a range of detailed studies in physiological relevant models – these studies are underway. We strongly feel such studies fall outside of the scope of this manuscript, instead they will be the subject of a future manuscript submission.

To address the important point regarding highlighting physiological relevance for these biased ligands raised by the reviewer, we have added an additional paragraph to the discussion:

'We previously published data comparing G-protein-biased and b-arrestin-recruiting ligands at GPR84 demonstrating that these translate to distinct biological responses in primary immune cells in vitro in a GPR84-specific manner.[16] We are applying the chemical tools we describe in this manuscript to address these important questions regarding the biological

consequences of signalling bias at GPR84 in a range of detailed studies in physiological relevant models.'

*Heterogeneity in reference ligands and quantification of ligand bias*:
*Using multiple reference ligands complicates the quantification and interpretation of ligand bias. In this particular case, for quantification of ligand bias it is not appropriate to use different reference ligands for the two signaling pathways being compared. Ideally (cf. IUPHAR Review 32), multiple reference ligands should be used for both pathways; at minimum, the same reference ligand should be used for both pathways.*

The reviewer is correct to highlight the importance of the use of reference ligands to define the extent of 'bias'. GPR84 is officially a Class A 'orphan' receptor. Currently the IUPHAR/BPS guide to pharmacology states that 'There is some evidence to suggest that (GPR42 and) GPR84 may be free fatty acid receptors'. Whilst GPR84 can certainly be activated by relatively high concentrations of medium chain-length fatty acids this means it is difficult to achieve full concentration-effect curves to define ligand $EC_{50}$. Moreover, there are numerous such fatty acids, including various hydroxylated and other variants of the basic saturated carbon chain that can activate the receptor. Hence defining a single fatty acid as the reference ligand is ill-advised. Hence, in the manuscript, subsequent to initial studies in which we provide the basic characteristics of OX04539/OX04529/OX04954 in comparison to the previously studied 6-OAU (for which we had earlier obtained a cryo-EM structure with a GPR84-Gi complex, **8G05**), we always use OX04539 as the reference ligand based on both high potency and effective regulation of both Gi-mediated signaling and its ability to effectively promote recruitment of arrestin isoforms. Moreover, as other key ligands (OX04529, OX04954) are structurally matched to OX04539 but with varying steric profiles then this has allowed up to address specifically how 'bias' varies with this parameter. We include the sentence for clarity on the use of reference ligand: 'OX04539 was subsequently used as a high potency structure-matched reference ligand throughout the studies.' on page 4. These are the key ligands used throughout the studies and specifically in determining how 'bias' alters with alteration of residues Leu336 and Phe187 and to subsequently extend markedly the chemical series to support the initial observations.

*Control of receptor expression levels*:
*The level of receptor expression influences $EC_{50}$ values obtained from functional assays and, consequently, the resulting quantification of signaling bias. This factor needs to be taken into account in the revised version of the manuscript.*

Once again the reviewer is correct to highlight the anticipated variation in ligand $EC_{50}$ with receptor expression. Many receptor point mutants may be expressed at different levels to wild type but overlaying this, point mutants may also be expressed but folded incorrectly so that they are unable to respond correctly to ligands. This sometimes results in the basis of loss of

function of certain mutants being incorrectly attributed.  In an earlier paper (Nat Commun. 2023 Sep 14;14(1):5706. doi: 10.1038/s41467-023-41201-0) we observed exactly this for GPR84. For example, as shown in **Supplementary Figure 3** of doi: 10.1038/s41467-023-41201-0 ), for the mutation Phe[335]Ala, when we measured expression based on binding of the allosteric radioligand antagonist [$^{3}$H]38 (described in other papers as [$^{3}$H]9543) the modified receptor was expressed as well as wild type, however when using the orthosteric radioligand antagonist [$^{3}$H]140 no specific binding was detected.

Thus, to address the 'functional expression' issue, for every mutant we describe in the current manuscript we test in G protein activation assays whether the mutant responds to the allosteric activator PSB-16671, for which we recently identified the binding site (bioRxiv: 10.64898/2026.04.10.715585v1, ref. 33), as effectively as does wild type GPR84. We include the following statement to make this point clear 'To define that these mutations did not result in lack of expression or general misfolding of the receptor we performed initial studies using the GPR84 allosteric agonist, PSB-16671[11,31,32], the binding site of which we recently identified.[33]' on page 7.

As the $EC_{50}$ for PSB-16671 was highly similar even in mutants that showed altered $EC_{50}$ to OX04539/OX04529/OX04954 (as detailed on page 7) we can conclude, based on the reviewers comments about expression levels and ligand $EC_{50}$, that the mutants are expressed to similar levels and that effects on the $EC_{50}$ of the sterically matched ligand set reflect receptor attributes specially related to this ligand set.

**Reviewer #4 (Remarks to the Author):**

*The manuscript has been carefully revised and has improved. The authors have addressed several of my concerns adequately; for instance, the presentation of TM6/TM7 movement data is now more clear, and the justification for why key mechanistic features emerge only in MD is reasonable and consistent with the literature. Their decision to soften their claims is also appreciated.*

*Regarding the role of MD simulations, I would still encourage the authors to explicitly acknowledge in the discussion that the simulations, by design, start from the cryo-EM-derived structural framework and therefore serve to elaborate and test (rather than independently discover) the proposed mechanism. This would not diminish the work, but would help readers assess how much the MD findings depend on the cryo-EM starting point.*

We thank the reviewer for this suggestion. We have revised the discussion to explicitly acknowledge that the MD simulations were built upon the cryo-EM structural framework, and therefore serve to elaborate and test, rather than independently discover, the proposed mechanism. The revised text now reads: 'MD simulations, built upon the cryo-EM structural framework to elaborate rather than independently discover the proposed mechanism, revealed that OX04529 stabilises a distinctive TM6 twist at its extracellular tip, separating TM6 from TM5 at the binding site'. (page 21).

*Regarding generalizability, the authors have made a genuine effort. Their analysis demonstrates plausibility rather than proof, but combined with the tempered language now used in the manuscript, this is a reasonable response. Further experimental validation in other receptors would strengthen the broader claim but this is beyond what can fairly be asked in revision.*

We thank the reviewers for their supportive comments.

*In summary, the authors have made meaningful improvements, including new data and analyses, softened mechanistic claims, and improved figure presentation. Whether the scope and generality of the findings are sufficient for publication in this journal is ultimately a decision for the editor.*
